# A Genome-Wide Knockout Screen in Human Macrophages Identified Host Factors Modulating *Salmonella* Infection

**DOI:** 10.1128/mBio.02169-19

**Published:** 2019-10-08

**Authors:** Amy T. Y. Yeung, Yoon Ha Choi, Amy H. Y. Lee, Christine Hale, Hannes Ponstingl, Derek Pickard, David Goulding, Mark Thomas, Erin Gill, Jong Kyoung Kim, Allan Bradley, Robert E. W. Hancock, Gordon Dougan

**Affiliations:** a Wellcome Sanger Institute, Hinxton, United Kingdom; b University of Cambridge Department of Medicine, Cambridge, United Kingdom; c Centre for Microbial Diseases and Immunity Research, Vancouver, Canada; d DGIST, Department of New Biology, Daegu, Republic of Korea; Institut Pasteur; Hudson Institute of Medical Research; University of Aberdeen

**Keywords:** CRISPR, *Salmonella*, bacteria, genome-wide screen, macrophages

## Abstract

A genome-scale CRISPR knockout library screen of THP-1 human macrophages was performed to identify loss-of-function mutations conferring resistance to *Salmonella* uptake. The screen identified 183 candidate genes, from which 14 representative genes involved in actin dynamics (*ACTR3*, *ARPC4*, *CAPZB*, *TOR3A*, *CYFIP2*, *CTTN*, and *NHLRC2*), glycosaminoglycan metabolism (*B3GNT1*), receptor signaling (*PDGFB* and *CD27*), lipid raft formation (*CLTCL1*), calcium transport (*ATP2A2* and *ITPR3*), and cholesterol metabolism (*HMGCR*) were analyzed further. For some of these pathways, known chemical inhibitors could replicate the *Salmonella* resistance phenotype, indicating their potential as targets for host-directed therapy. The screen indicated a role for the relatively uncharacterized gene *NHLRC2* in both *Salmonella* invasion and macrophage differentiation. Upon differentiation, *NHLRC2* mutant macrophages were hyperinflammatory and did not exhibit characteristics typical of macrophages, including atypical morphology and inability to interact and phagocytose bacteria/particles. Immunoprecipitation confirmed an interaction of NHLRC2 with FRYL, EIF2AK2, and KLHL13.

## Abstract (precis)

*Salmonella* exploits macrophages to gain access to the lymphatic system and bloodstream to lead to local and potentially systemic infections. With an increasing number of antibiotic-resistant isolates identified in humans, *Salmonella* infections have become major threats to public health. Therefore, there is an urgent need to identify alternative approaches to anti-infective therapy, including host-directed therapies. In this study, we used a simple genome-wide screen to identify 183 candidate host factors in macrophages that can confer resistance to *Salmonella* infection. These factors may be potential therapeutic targets against *Salmonella* infections.

## INTRODUCTION

Salmonella enterica, a major cause of disease in humans and animals (1–3), normally infects via the intestinal epithelia, where entry into macrophages provides a key route toward tissue dissemination and systemic spread (4, 5). Macrophages are also key immune phagocytes that serve as a first line of defense against many bacterial pathogens, including *Salmonella*. Following bacterial engulfment, macrophages can present antigens to other immune cells and secrete molecules such as cytokines and chemokines to recruit more immune cells to the area of infection (6).

Salmonellae are facultative intracellular pathogens with a tropism for macrophages (7), and many studies have demonstrated that the outcome of an infection is significantly dependent on how *Salmonella* initially interacts with macrophages. *Salmonella* uptake by macrophages is mediated by phagocytosis, although *Salmonella* can also exploit the *Salmonella* pathogenicity island-1 type III secretion system to actively invade the macrophages (8). The various phagocytic receptors expressed on the plasma membrane of macrophages recognize structures and molecules expressed by *Salmonella*, and the phagocytic mechanisms initiated by each of these receptors employ different mechanisms and signaling pathways (9). These signaling pathways stimulate actin rearrangements, resulting in massive ruffling and extrusion of the plasma membrane to engulf *Salmonella*. Once inside the macrophage, salmonellae are enclosed within a specialized intracellular compartment termed the *Salmonella-*containing vacuole (SCV), which limits fusion with lysosomes, enabling the bacteria to thrive (10).

Despite significant progress in understanding the early interaction of *Salmonella* and macrophages, the picture is incomplete. Importantly, a comprehensive and unbiased analysis of human macrophage factors required for *Salmonella* uptake is needed. The development of CRISPR/Cas9 technology enables generation of complete loss-of-function alleles in a variety cell types, thus enabling functional genetic analyses in higher eukaryotes (11, 12). Here, we performed a genome-wide CRISPR/Cas9 knockout screen to identify host factors involved in *Salmonella* uptake in a human THP-1 macrophage model. Our screen led to the identification of 183 candidate genes that may play roles in conferring *Salmonella* resistance in macrophages, including genes involved in actin binding, receptor signaling, lipid raft formation, calcium transport, and cholesterol metabolism, as well as those of limited known function. In this article, the potential of these genes as targets for host-directed therapies and the role of one example, *NHLRC2*, are explored.

## RESULTS

### Genome-wide CRISPR screen for host factors involved in early macrophage-*Salmonella* interaction.

**FIG 1:**
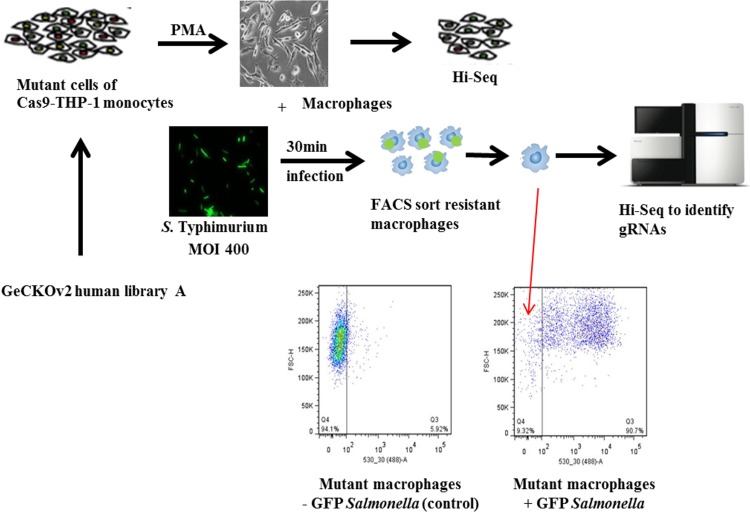
Schematic of a CRISPR/Cas9 screen setup to identify host factors involved in early *Salmonella*-macrophage interaction. Cas9-THP-1 monocytes were transduced with GeCKO human pooled gRNA library A at an MOI of 0.3 and at 100× coverage. The mutant library cells were selected in puromycin for 2 weeks. After drug selection, genomic DNA was extracted from 1 × 10^7^ mutant cells. Genomic DNA was used as the template for PCR and barcoding for Hi-Seq to evaluate sgRNA coverage in the THP-1-GeCKO library. The remaining mutant cells were differentiated into macrophages using PMA, and genomic DNAs were extracted from a subset of the mutant macrophages. *S.* Typhimurium expressing constitutive GFP was used to infect the mutant library macrophages for 30 min. The infected cells were washed and sorted on a flow cytometer for GFP-negative macrophages. Genomic DNA was extracted from the resultant sorted cells and used as the template for PCR and barcoding for Hi-Seq to identify enriched gRNAs. Three biological replicates were performed.

To identify host genes critical for early macrophage-*Salmonella* interaction, a genome-wide loss-of-function genetic screen was performed (see Fig. 1 for schematic) using a genome-scale CRISPR Knock-Out (GeCKO) v2.0 single-guide RNA (sgRNA) pooled library (13) transfected into human THP-1-derived macrophages. A Cas9-expressing THP-1 cell line (Cas9-THP-1) was demonstrated to express Cas9 activity using a Cas9 functional assay (see Fig. S1A in the supplemental material). Cas9-THP-1 monocytes were transduced with lentiviruses expressing the pooled GeCKO library A sgRNAs, followed by puromycin selection. To evaluate sgRNA target diversity in the THP-1-GeCKO library, PCR amplicons of the integrated sgRNA cassettes from all 3 transductions were sequenced. Primer sequences for PCR amplification of gRNAs are listed in Table S1 in the supplemental material.

10.1128/mBio.02169-19.2 FIG S1 Cas9 functional assay and *Salmonella* infection of Cas9-THP-1 macrophages. (A) Cas9-THP-1 and THP-1 control cells were transduced with lentivirus produced with vector containing a GFP gRNA expressed under the U6 promoter and constitutively expressing blue fluorescent protein (BFP). The ratios of BFP only and GFP-BFP double-positive cells were analyzed using the LSRFortessa flow cytometer. (Left panel) Cas9-THP-1 cells with empty vector. (Middle panel) Cas9-THP-1 cells with BFPgGFP vector. (Right panel) Control THP-1 cells with BFPgGFP vector. (B) Cas9-THP-1 macrophages were infected for 30 min with various MOI of the *S.* Typhimurium SL1344 strain constitutively expressing GFP. GFP expression was detected by flow cytometry. Download FIG S1, PDF file, 0.2 MB. Copyright © 2019 Yeung et al. 2019 Yeung et al. This content is distributed under the terms of the Creative Commons Attribution 4.0 International license.

10.1128/mBio.02169-19.6 TABLE S1 Primer or gRNA sequences used in this study. Download Table S1, DOCX file, 0.1 MB. Copyright © 2019 Yeung et al. 2019 Yeung et al. This content is distributed under the terms of the Creative Commons Attribution 4.0 International license.

The Salmonella enterica serovar Typhimurium SL1344(pGFPmut3.1) strain, constitutively expressing green fluorescent protein (GFP), was utilized for the *Salmonella*-exposed macrophage uptake screen. Cas9-THP-1 cells were infected with various multiplicities of infection (MOI) of the *S.* Typhimurium pGFPmut3.1 strain, and at an MOI of 400, a maximum macrophage infectivity of 90 to 95% was achieved without significantly increasing the number of dead macrophages (Fig. S1B). Consequently, 3 independent pools of mutant library macrophages were separately infected at an MOI of 400 for 30 min.

**FIG 2:**
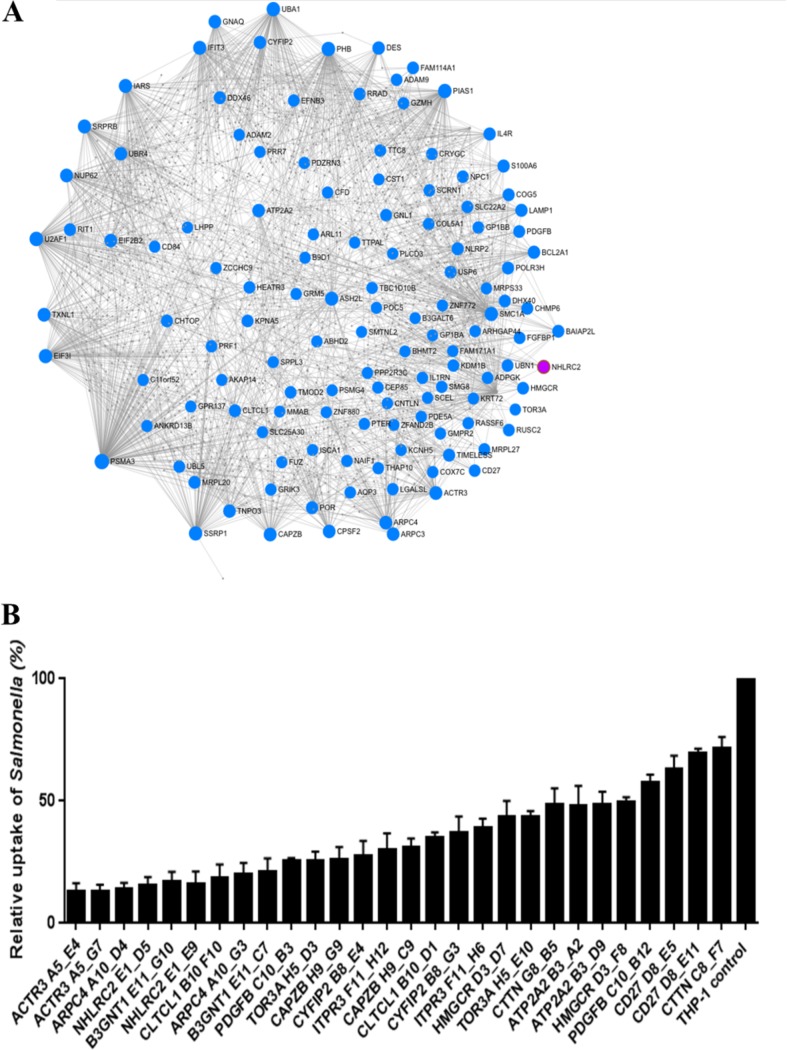
Interaction network of *Salmonella*-resistant mutants and relative uptake of *Salmonella* for mutant versus WT THP-1 macrophages. (A) One hundred eighty-three candidate genes with *Salmonella-*resistant phenotypes (nodes highlighted in blue, with *NHLRC2* highlighted in magenta) were submitted to NetworkAnalyst to identify known first-order protein-protein interactors (gray nodes) using the IMEx Interactome database. The observed protein-protein interaction network indicates functional interactions between the individual proteins that when deleted demonstrate *Salmonella* resistance. (B) Selected homozygous, compound heterozygous, or heterozygous clonal mutants, generated from the same gRNA, that displayed the strongest *Salmonella*-resistant phenotypes are shown. The mutant and WT Cas9-THP-1 macrophages were infected with *S.* Typhimurium constitutively expressing GFP for 30 min at an MOI of 400. Infected macrophages were washed, and GFP was measured using a flow cytometer. Results are averages from at least 3 independent experiments. For the labeling of each mutant cell line, the name of the gene is followed by the gRNA used (Table S3A) and the position of the clone picked.

*Salmonella*-exposed macrophages in dishes were washed, and medium was replaced with fresh medium containing 50 μg/ml gentamicin for 30 min to kill the extracellular bacteria. The infection state of the mutant macrophages was monitored at the single-cell level by flow cytometry (Fig. 1), and the viable, GFP-negative macrophages were sorted for genomic DNA (gDNA) extraction. Deep-sequencing analysis in both sorted and unsorted cells (cells with GFP-expressing *Salmonella*) showed that a subset of sgRNAs were strongly enriched in the sorted cells (see Fig. S2A in the supplemental material). Summary reports from 3 independent experiments were generated for enriched guide RNAs (gRNAs) identified from each screen using MAGeCK (14) (Fig. S2B). Specific criteria were defined to enable us to combine the 3 gRNA summary reports into a single candidate gene list. Thus, for each summary report, we first removed all gRNAs with a false-discovery rate (FDR) of >0.0001 and then generated a combined candidate gene list based on genes with at least 2 gRNA hits in at least 2 of the 3 experiments. This identified 183 candidate genes ranked by FDR (see Table S2, part A, in the supplemental material). These 183 candidate genes formed a densely connected first-order protein-protein interaction (PPI) network, indicating the potential functional importances of these resistance genes (Fig. 2A).

10.1128/mBio.02169-19.3 FIG S2 Deep sequencing analyses of sgRNAs in the THP-1-GeCKO library. Shown are read coverage plots for evaluation of the sgRNA diversity in (A) pre-PMA-treated monocytes, (B) post-PMA-treated macrophages, and (C) post-FACS-sorted (*Salmonella-*negative) macrophages. (D) Scatterplot showing enrichment of specific sgRNAs in the screen after each infection. Plots were generated with the MAGeCK statistical package v.0.5.2. Download FIG S2, PDF file, 0.1 MB. Copyright © 2019 Yeung et al. 2019 Yeung et al. This content is distributed under the terms of the Creative Commons Attribution 4.0 International license.

10.1128/mBio.02169-19.7 TABLE S2 (A) List of candidate genes identified from *Salmonella-*macrophage genome-wide CRISPR screen. (B) List of overrepresented pathways identified for the candidate genes using InnateDB. Only pathways with corrected *P* values of <0.5 are shown. Download Table S2, DOCX file, 0.2 MB. Copyright © 2019 Yeung et al. 2019 Yeung et al. This content is distributed under the terms of the Creative Commons Attribution 4.0 International license.

10.1128/mBio.02169-19.8 TABLE S3 (A) List of selected candidate genes and gRNA sequences chosen for validation. (B) Percentage of relative uptake of *Salmonella* for mutant versus WT control. (C) Zygosity of selected clonal mutants as determined by MiSeq. Download Table S3, DOCX file, 0.1 MB. Copyright © 2019 Yeung et al. 2019 Yeung et al. This content is distributed under the terms of the Creative Commons Attribution 4.0 International license.

We submitted the candidate gene list to a web database pathway analysis tool, InnateDB (15), to identify overrepresented pathways (Table S2, part B). This pathway analysis revealed a number of genes/pathways previously associated with *Salmonella* infection, including regulation of actin rearrangements/RAC1/CDC42 signaling (*ACTR3*, *ARPC3*, *ARPC4*, and *CYFIP2*), membrane trafficking (*ACTR3*, *CHRM2*, *CTTN*, *ARPC2*, *CAPZB*, *CLTCL1*, and *ARPC4*), and Fcγ receptor-dependent phagocytosis (*ACTR3*, *ARPC3*, *ARPC4*, *CYFIP2*, and *FCGR1A*). There were also a number of genes/pathways that had not been linked to *Salmonella* infections, including regulation of cellular cholesterol (*HMGCR* and *NPC1*), calcium trafficking (*ATP2A2* and *ITPR3*), glycosaminoglycan metabolism (*B3GNT1*, *B3GALT6*, and *HS6ST2*), intracellular endosomal trafficking (*CLTCL1*), and various signaling ligands/receptors (*PDGFB*, *CD27*, *TLR2*, *TLR7*, *EFNB3*, *GRM5*, *CHRM2*, and *FDG1*). Interestingly only 5 of these genes overlapped with those identified in a recent genome-wide CRISPR/Cas9 screen of genes involved in phagocytic uptake of paramagnetic particles (16).

### Validation of selected gene candidates identified from the genome-wide screen.

Of the 183 candidate genes, 19 were selected for validation by constructing targeted mutations using specific sgRNAs. Genes selected for validation were based on their rankings in our candidate gene list and their known or unknown biological functions. Five gRNA expression vectors were constructed for each gene (see Table S3, part A, in the supplemental material), and these gRNAs were individually tested to see whether they would give rise to resistant cells upon *S.* Typhimurium infection (Table S3, part B). Fourteen genes (with at least three sgRNAs) gave moderate to strong resistance to *S.* Typhimurium infections (≤75% fold decrease of GFP-positive cells for the mutant versus the wild-type [WT] control [empty vector only]), while 5 genes gave only slight or no improved resistance to the infections (>75% fold decrease of GFP-positive cells for mutant versus WT) (Table S3, part B). For these 14 genes, we chose the two sgRNAs per gene that gave the highest resistance to *Salmonella*, and single-cell sorted a total of 28 mixed-sample populations to generate clonal mutants. The clonal mutant lines were screened for resistance to *S.* Typhimurium. The gene mutations for selected clonal mutants that showed moderate to strong resistance to *S.* Typhimurium (as highlighted in Fig. 2) were determined by amplifying genomic regions harboring gRNA cutting sites and determining their sequence. The zygosities of the clonal mutants highlighted in Fig. 2 are listed in Table S3, part C. Primer sequences for PCR amplification of the sgRNAs for MiSeq are listed in Table S1.

The top-ranking candidate genes selected for validation included *ACTR3* and *ARPC4* (part of Arp2/3 complex involve in actin binding/reorganization). The role of the Arp2/3 complex in *Salmonella* infections has been somewhat conflicting (17–19), but here we demonstrated that mutants with clonal mutations generated in the *ACTR3* and *ARPC4* genes showed strong resistance to *S.* Typhimurium infections (Fig. 2B). For example, the percentage of *Salmonella* GFP-positive cells for a compound heterozygous mutant clone, ACTR3_A5_E4, versus the WT THP-1 control was reduced to only 13.1% ± 3.3%, and the percentage of a compound heterozygous mutant clone, ARPC4_A10_D4, was reduced to 14.2% ± 2.3%.

**FIG 3:**
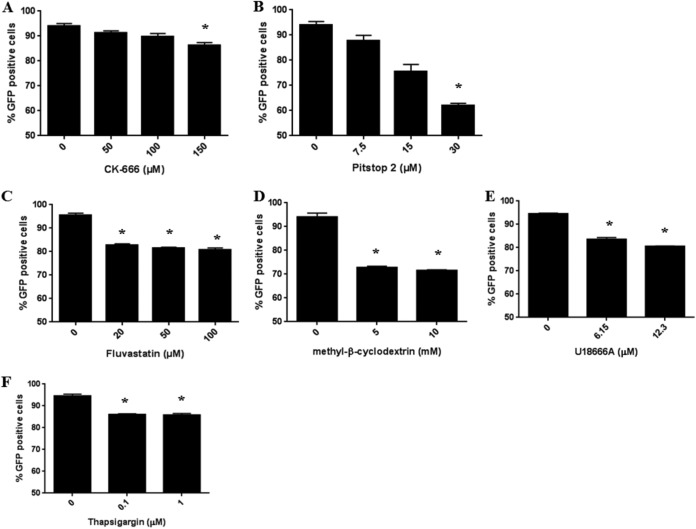
Effect of inhibitors on *S.* Typhimurium infection of THP-1 macrophages. THP-1 macrophages were infected with *S.* Typhimurium constitutively expressing GFP at an MOI of 400 in the presence or absence of the indicated inhibitor for 30 min. Postinfection, the cells were washed, and GFP was measured using a flow cytometer. Results are the average of 3 independent experiments ± standard deviation (SD). * indicates statistically significant difference (*P < *0.05) between untreated and treated as determined using Student's *t* test.

Other genes on the list that are involved in actin dynamics, but have a less-well-understood relevance to host-pathogen interactions, were investigated, including *CAPZB* (F-actin-capping protein subunit beta), *CYFIP2* (cytoplasmic FMR1 interacting protein 2), *TOR3A* (torsin family 3 member A), and *CTTN* (cortactin). Clones with mutations in each of these genes showed moderate to strong resistance to *S.* Typhimurium infection. The reductions in *Salmonella* GFP-positive cells, compared to WT, were to 26.0% ± 5.2% for the CAPZB_H9_G9 heterozygous mutant clone, 27.5% ± 6.1% for the CYFIP2_B8_E4 homozygous mutant clone, 25.3% ± 4.0% for the TOR3A_H5_D3 compound heterozygous mutant clone, and 48.4% ± 6.7% for the CTTN_G8_B5 compound heterozygous mutant clone. Furthermore, WT THP-1 control macrophages were treated with CK-666 (Arp2/3 complex inhibitor), and infection with *Salmonella* was assessed. Compared to untreated controls, only a small increase in resistance to *Salmonella* infection was observed (i.e., 91.8% ± 1.1% of *Salmonella* GFP-positive cells for macrophages treated with 150 μM CK-666 compared to untreated macrophages) (Fig. 3A).

Interestingly *CLTCL1*, which encodes the minor clathrin heavy chain, was identified from our screen. While studies have shown the role of clathrins in mediating internalization of various bacteria, including Escherichia coli and Listeria monocytogenes, into host cells (20, 21), the dependence of *Salmonella* internalization on clathrin is unclear (22, 23). Clonal mutants of *CLTCL1* showed strong resistance to *Salmonella* infection, with residual *Salmonella* GFP-positive cells being at only 18.5% ± 5.5% for the CLTCL1_B10_F10 compound heterozygous mutant clone compared to the WT (Fig. 2B). Alternatively, we infected WT control macrophages that have been treated with Pitstop 2 (clathrin inhibitor) and showed that at 30 μl, the macrophages were moderately resistant to *S.* Typhimurium infection (66.0% ± 0.9%) (Fig. 3B).

HMGCR (HMG-coenzyme A [CoA] reductase) is involved in regulating cellular cholesterol levels and cholesterol biosynthesis. Studies have shown the important role of cholesterol in *Salmonella* infections (24, 25). *HMGCR* clonal mutants showed moderate resistance to *S.* Typhimurium infection. The percentage of GFP-positive *Salmonella* taken up into the HMGCR_D3_D7 compound heterozygous mutant clone was reduced to 43.5% ± 6.5% compared to the WT (Fig. 2B). Alternatively, WT macrophages were treated with 100 μM HMG-CoA reductase inhibitor fluvastatin, and macrophage uptake of *Salmonella* was modestly reduced to 84.7% ± 0.8% compared with untreated macrophages (Fig. 3C). Treatment with other cholesterol inhibitors, including methyl-β-cyclodextrin (Mβ-CD) and U18666A (cholesterol transport inhibitor), also improved resistance of WT control macrophages to *S.* Typhimurium infection, with percentages of residual uptake of 76.0% ± 0.6% for Mβ-CD and 85.2% ± 1.3% for U18666A (Fig. 3D and E).

Another top-ranking candidate gene was *ATP2A2* (calcium ATPase), which is involved in maintaining cellular calcium homeostasis by translocation of calcium from the cytosol to the endoplasmic reticulum (ER) (26). Cytosolic calcium regulates a wide variety of global cellular processes, including cell growth and apoptosis (27), and was recently shown to play a critical role in microbial infections (28, 29). In addition to *ATP2A2*, *ITPR3* (a second messenger that mediates release of intracellular calcium) was chosen for validation. *ATP2A2* and *ITPR3* clonal mutants showed moderate resistance to *S.* Typhimurium infection. Thus, we observed decreases in *Salmonella* GFP-positive cells compared with the WT control, to 48.2% ± 8.0% for the ATP2A2_B3_A2 compound heterozygous mutant clone and 29.8% ± 7.0% for the ITPR3_F11_H12 compound heterozygous mutant clone (Fig. 2B). Consistent with this, WT macrophages treated with 1 μM thapsigargin (Ca^2+^ ATPase inhibitor) were slightly resistant to *Salmonella* infection (90.8% ± 0.9%) (Fig. 3F).

The *B3GNT1* gene encodes a β-1,4-glucuronyltransferase involved in glycosaminoglycan (GAG) metabolism. GSGs (complex linear polysaccharides) interact with various molecules to regulate several cellular processes, but in particular cell surface GAGs engage pathogens, including *Salmonella*, to promote their attachment and invasion of host cells (30–32). *B3GNT1* clonal mutants showed strong resistance to *S.* Typhimurium infection. A strong decrease in *Salmonella* GFP-positive cells to 16.8% ± 5.5% was observed for the B3GNT1_E11_G10 compound heterozygous mutant clone (Fig. 2B).

Platelet-derived growth factor (PDGF) is a growth factor with roles in cell proliferation, chemotaxis, and induction of reactive oxygen species (ROS) production (33). While it has been shown that PDGF stimulates phagocytosis of neutrophils (34), its role in *Salmonella* infection is unclear. Here, we showed that the PDGFB_C10_B3 homozygous mutant clone decreased *Salmonella* uptake to 25.6% ± 1.1% relative to the control. CD27 is a member of the tumor necrosis factor receptor (TNFR) superfamily and binds to CD70 to activate NF-κB and MAPK8/JNK. Blocking the CD27-CD70 interaction has been shown to eliminate persistence of lymphocytic choriomeningitis virus (LCMV) infection (35), while CD27^+^ B cells internalized *Salmonella* more efficiently than CD27^−^ B cells (36). Here, we showed that a homozygous mutant clone, CD27_D8_E5, showed moderate resistance to *S.* Typhimurium infection, reducing uptake to 63.0% ± 5.5% (Fig. 2B).

Another candidate, *NHLRC2* (NHL-repeat-containing protein 2), was one of the top 3 mutants identified in our genome-wide screen. A homozygous mutant clone, NHLRC2_E1_D5, showed strong resistance to *Salmonella* infection, reducing uptake to 15.4% ± 3.5% compared to the WT control cells (Fig. 2B). Intriguingly, similar results were recently observed using alternative assessment methods, including imaging and serial plating (see Fig. S3A and B in the supplemental material). NHLRC2 was very recently identified as a central player in phagocytosis of superparamagnetic substrates, acting by regulating the RhoA-Rac1 signaling cascades that control actin polymerization and filopodium formation. Intriguingly although 86 genes were found to be critical for phagocytosis of superparamagnetic substrates in U937 macrophages, only 5 overlapped with our study on uptake, including *NHLRC2*, *RIT1*, and 3 genes from the Arp2/3 complex (*ACTR3*, *ARPC3*, and *ARPC4*) (16), indicating that these two biological processes are very different. Since nothing was known about the biological function of NHLRC2 in *Salmonella* infection, it was chosen for study in greater detail.

10.1128/mBio.02169-19.4 FIG S3 *S.* Typhimurium infections and cellular expression of NHLRC2 in WT and *NHLRC2* mutant THP-1 macrophages. (A) WT and clonal *NHLRC2* mutant macrophages were infected with *S.* Typhimurium constitutively expressing GFP at an MOI of 50 for 30 min. Uninfected macrophages were used as a control. Postinfection, the macrophages were washed and lysed with 0.1% Triton X-100. Serial dilutions of the lysed cells were made and spotted onto agar plates. The plates were incubated for 16 to 18 h at 37°C, and the resultant CFU/ml were calculated. Results are the average of 3 independent experiments ± SD. * indicates statistically significant difference (*P < *0.05) between the WT and each clonal mutant as determined using Student’s *t* test. (B) WT and clonal *NHLRC2* mutant macrophages were infected with *S.* Typhimurium constitutively expressing GFP at an MOI of 400 for 30 min. Postinfection, the macrophages were washed, and GFP intensity was measured using the CellInsight NXT high-content screening platform (Thermo Fisher Scientific). Results are the average of 3 independent experiments ± SD. * indicates statistically significant difference (*P < *0.05) between the WT and each clonal mutant as determined using Student’s *t* test. (C) THP-1 macrophages were fixed, permeabilized, blocked, and stained with anti-NHLRC2 rabbit polyclonal antibody (HPA038493; Sigma) and anti-GORASP2 mouse monoclonal antibody (AMAb91016; Sigma) as the primary antibodies. Subsequently, the cells were washed and incubated with anti-rabbit AF488 (A-11008; Thermo Fisher) and anti-mouse AF647 (A-21235; Thermo Fisher) as the secondary fluorescent antibodies. Finally, the stained cells were mounted onto coverslips with Prolong Gold antifade reagent with DAPI for confocal imagining at a 40× objective. The top 2 panels (left to right) represent staining with DAPI (blue) and NHLRC2 (green), and the bottom panels (left to right) represent staining with GORASP2 (red) and a merge of all 3 stains. (D, top panel) Human iPS-derived macrophages were stained with primary conjugated anti-NHLRC2-AF488 antibody (bs-9322R-A488, Bioss Antibodies) and anti-GORASP2 mouse monoclonal antibody. Subsequently, the cells were incubated with secondary fluorescent anti-mouse AF647 antibody. Finally, the stained cells were mounted onto coverslips with DAPI for confocal imaging at a 60× objective. The first 3 panels represent individual staining with DAPI (blue), NHLRC2 (green), and GORASP2 (red), and the final panel is a merge of all 3 stains. (Bottom panel) THP-1 NHLRC2 E1_C5 mutant macrophages were stained with primary anti-NHLRC2 rabbit polyclonal antibody (HPA038493; Sigma) and secondary anti-rabbit AF488 antibody. Cells were mounted onto coverslips with DAPI for confocal imagining at a 40× objective. The first 2 panels represent individual staining with DAPI (blue) and NHLRC2 (green), and the final panel is a merge of the 2 stains. Download FIG S3, PDF file, 0.1 MB. Copyright © 2019 Yeung et al. 2019 Yeung et al. This content is distributed under the terms of the Creative Commons Attribution 4.0 International license.

### Characterization of the *NHLRC2* gene identified from the genome-wide screen.

NHLRC2 is a 79-kDa protein of 726 amino acids that harbors an N-terminal thioredoxin-like (Trx-like) domain and a C-terminal NHL repeat domain. Whole-exome sequencing (WES) of samples from children with FINCA (fibrosis neurodegeration cerebral angiomatosis) identified mutations in NHLRC2 (compound heterozygous mutations p.Asp148Tyr and p.Arg201GlyfsTer6 [37]) associated with fatality from FINCA in early childhood. The *NHLRC2* gene is present across all kingdoms of life from mammals to prokaryotes, and its sequences are highly conserved across evolution (38).

**FIG 4:**
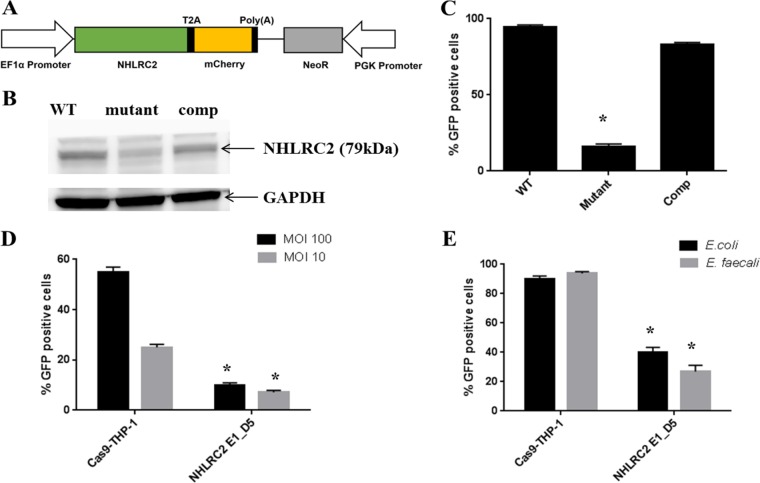
NHLRC2 complementation vector and *S. Typhimurium* infection of WT and *NHLRC2* mutant THP-1 macrophages. (A) Schematic view of NHLRC2 complementation vector comprised of EF1α promoter, human *NHLRC2* open reading frame (ORF) clone, T2A-mCherry, neomycin-resistant gene, and a PGK promoter. (B) WT, NHLRC2_E1_D5 mutant, and *NHLRC2* complemented mutant samples were probed with anti-NHLRC2 primary antibody (HPA038493) and horseradish peroxidase (HRP)-conjugated anti-rabbit IgG secondary antibody. The resultant bands were visualized on an ImageQuant Las 4000. Anti-GAPDH antibody was used as a loading control. (C and D) WT, NHLRC2_E1_D5 mutant, and *NHLRC2* complemented macrophages were infected with *S.* Typhimurium constitutively expressing GFP for 30 min at MOI of (C) 400 and (D) 100 or 10. (E) WT and NHLRC2_E1_D5 mutant macrophages were infected with carboxyfluorescein succinimidyl ester (CFSE)-stained E. coli or E. faecalis for 30 min at an MOI of 400. Uninfected macrophages were used as a control. Postinfection, the cells were washed, and GFP was measured using a flow cytometer. The results shown are the average of 3 independent experiments ± SD. * indicates a statistically significant difference (*P < *0.05) between the WT and *NHLRC2* mutant as determined using Student's *t* test.

Sequencing of the above-mentioned homozygous mutant revealed that the NHLRC2_E1_D5 clonal mutant had a biallelic single-nucleotide variation (SNV) that resulted in amino acid change from proline to histidine at amino acid position 155 within the thioredoxin domain. This mutation did not completely obliterate protein expression of NHLRC2, as shown by Western blotting (Fig. 4B). In repeated attempts, we were unsuccessful in generating a null mutant in the THP-1 cells that would result in complete abolishment of its protein expression. Transduction of the mutant cells with a *NHLRC2* cDNA (construct shown in Fig. 4A) restored the protein expression in the macrophages to the level found in unedited THP-1 cells (Fig. 4B), as well as restoring *Salmonella* infectivity of macrophages (Fig. 4C).

The response of the *NHLRC2* mutant macrophages to *S.* Typhimurium was studied at more physiologically relevant MOIs, and strong resistance to *Salmonella* infection was still observed (e.g., at an MOI of 10, uptake of *Salmonella* GFP-positive cells was decreased to just 28.8% ± 0.8% compared with the WT control cells) (Fig. 4D). This mutant had a general deficiency in bacterial uptake since the *NHLRC2* mutant macrophages also demonstrated a 60 to 70% deficiency in uptake of another Gram-negative bacterium, E. coli, and a Gram-positive bacterium, Enterococcus faecalis (Fig. 4E).

**FIG 5:**
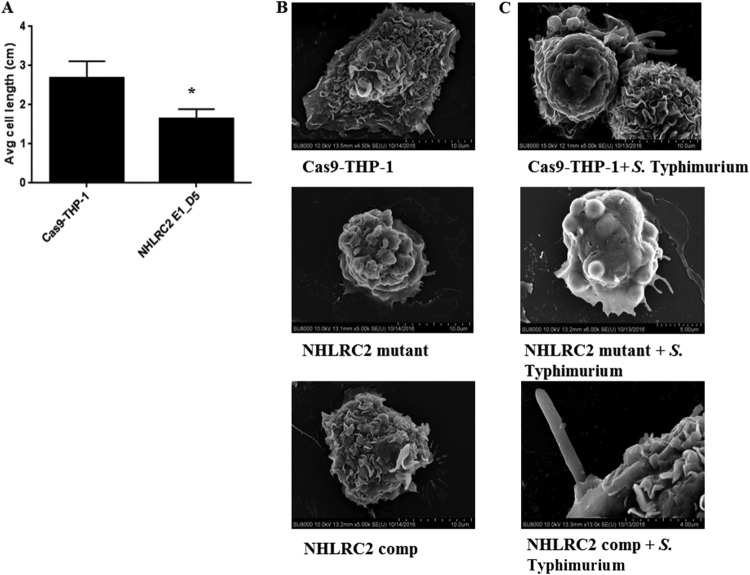
Characterization of WT and *NHLRC2* mutant macrophages. (A) Cell size comparison for WT and *NHLRC2* mutant macrophages. Phase-contrast microscopy at a resolution of ×20 magnification was used to image WT and NHLRC2_E1_D5 macrophages. Results are the average of 50 cells ± SD. * indicates statistically significant difference (*P < *0.05) between the WT and each clonal mutant as determined using Student's *t* test. (B and C) Representative SEM images of (B) naive and (C) *S.* Typhimurium-infected WT, NHLRC2 mutant, and NHLRC2 complemented (comp) macrophages.

The cell morphology of the *NHLRC2* mutant macrophages was investigated using phase-contrast microscopy and scanning electron microscopy (SEM). Phase-contrast microscopy showed that the *NHLRC2* mutant macrophages lacked filopodia and appeared smaller (cell length measurements of 50 WT and mutant THP-1 macrophages showed mutants were ∼40% shorter) (Fig. 5A). SEM showed that *NHLRC2* mutant macrophages were missing membrane ruffles (Fig. 5B), which are dynamic structures formed by actin filaments that play important roles in pathogen detection and actin-dependent internalization during phagocytosis (39, 40). While we observed the formation of the phagocytic cups required for internalization of *Salmonella* in WT THP-1 macrophages, these structures could not be observed in the *NHLRC2* mutant macrophages (Fig. 5C). While complementation of the *NHLRC2* mutant macrophages with functional NHLRC2 cDNA restored the ability of the macrophages to form the phagocytic cups in the presence of *Salmonella* (Fig. 5C).

**FIG 6:**
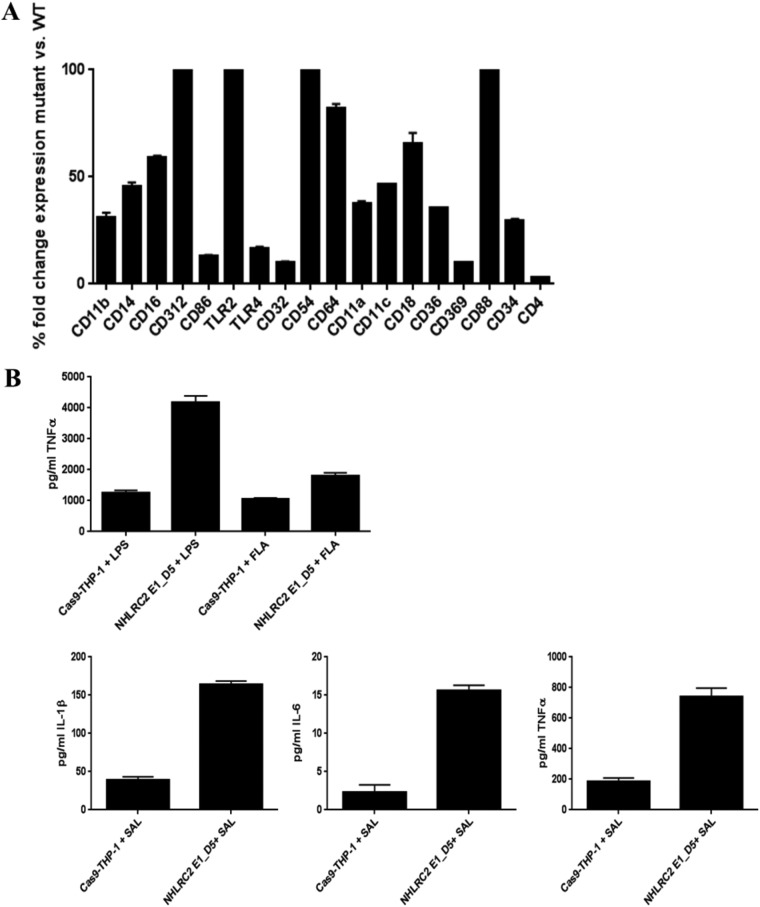
Evaluation of cell surface markers and inflammatory cytokines produced by WT and NHLRC2 mutant macrophages. (A) Expression of selected surface markers on WT and *NHLRC2* mutant macrophages was determined using a flow cytometer. Results shown are the mean percentages of fold changes from 3 independent experiments ± SD. (B) Expression of TNF-α, IL-6, and IL-1β in supernatants of WT and *NHLRC2* mutant macrophages stimulated with LPS, flagellin, or live *S.* Typhimurium cells was determined using a flow cytometer. The results shown are the averages from 3 independent experiments ± SD.

To gain insights into the inability of the *NHLRC2* mutant macrophages to interact with *Salmonella*, a transcriptome sequencing (RNA-Seq) experiment was performed to compare the RNA expression profiles of *NHLRC2* mutant and WT THP-1 macrophages. RNA-Seq results showed that more than a quarter of human protein-encoding genes were dysregulated as a result of the *NHLRC2* mutation. Of the 5,156 dysregulated genes with adjusted *P* values of <0.01, 2,101 genes were upregulated with fold changes (FCs) of ≥1.5, and 3,055 genes were downregulated with FCs of ≤1.5 (Table S4, part C). To understand the functional differences between *NHLRC2* transcriptome and the WT, we utilized Sigora (41) to perform pathway overrepresentation analyses. Prominent downregulated pathways in the mutant included various cell cycle processes, the RHO GTPase cycle, interferon alpha/beta signaling, cell death signaling via NRAGE, NRIF, and NADE, and antigen processing and presentation (Table S4, part A). Specifically, we observed that many genes encoding receptors involved in the recognition and engulfment of pathogens, signal transduction, and actin reorganization were downregulated in the *NHLRC2* mutant (Table 1). Downregulation of several cell surface receptors (i.e., Fc receptors) was confirmed by flow cytometry (Fig. 6A). Moreover, as expected we observed reduced uptake of opsonized *Salmonella* in the *NHLRC2* mutant macrophages (data not shown). Interestingly, among the upregulated pathways were several immune pathways, including regulation of nitric oxide-sensitive guanylyl cyclase, cytokine-cytokine receptor interaction, and genes involved in the proinflammatory response, such as those coding for tumor necrosis factor (TNF), interleukin-6 (IL-6), and IL-1 (Table S4, part B). Cytokine analysis after lipopolysaccharide (LPS), flagellar, or whole-bacterial stimulation confirmed that the *NHLRC2* mutant expressed ∼4- to 6-fold-higher levels of IL-1β, TNF-α, and IL-6 than WT macrophages (Fig. 6B).

**TABLE 1:** Selected genes downregulated in the *NHLRC2* mutant compared to WT THP-1 macrophages, as determined by RNA-Seq

Gene ID	Gene function(s)	FC for mutant vs WT
*FCGR1A*	Receptors	−3.58
*FCGR3A*	Receptors	−3.50
*FCGR2A*	Receptors	−3.27
*FCGR1B*	Receptors	−2.19
*ITGAM*	Receptors	−3.57
*CD36*	Receptors	−7.32
*CLEC7A*	Receptors	−7.08
*TLR4*	Receptors	−1.73
*CD47*	Recognition and engulfment	−2.90
*ELMO1*	Recognition and engulfment	−2.67
*MCOLN3*	Recognition and engulfment	−10.85
*SCARB1*	Recognition and engulfment	−3.60
*SIGLEC1*	Recognition and engulfment	−17.52
*SIGLEC11*	Recognition and engulfment	−3.28
*AXL*	Signal transduction	−11.02
*MERTK*	Signal transduction	−1.76
*SYK*	Signal transduction	−1.95
*ARPIN*	Actin reorganization	−10.40
*ARPC5*	Actin reorganization	−1.93
*ARPC1B*	Actin reorganization	−1.54
*ACTB*	Actin reorganization	−1.59

10.1128/mBio.02169-19.9 TABLE S4 (A) List of overrepresented pathways for downregulated genes using Sigora. (B) List of overrepresented pathways for upregulated genes using Sigora. (C) List of dysregulated genes expressed in *NHLRC2* mutant versus WT THP-1 macrophages by RNA-Seq. Only genes with fold changes of ≤1.5 or ≥1.5 and adjusted *P* values of <0.01 are shown. Download Table S4, DOCX file, 0.7 MB. Copyright © 2019 Yeung et al. 2019 Yeung et al. This content is distributed under the terms of the Creative Commons Attribution 4.0 International license.

Subcellular fractionation of fibroblasts previously showed that the majority of the NHLRC2 protein was localized in the cytosol (42). Here, we utilized immunofluorescence to investigate the localization of NHLRC2 to organelles by costaining the WT THP-1 macrophages with NHLRC2 antibody and various organelle antibodies, including those binding to the endoplasmic reticulum (ER), mitochondria, and peroxisome. We showed that NHLRC2 antibody stain only merged with the GORASP2 (Golgi reassembly-stacking protein 2) antibody stain (Fig. S3C). A similar staining pattern was also observed using a different NHLRC2 antibody with macrophages derived from human iPS cells (Fig. S3D). We also showed significant reduction in NHLRC2 antibody staining in *NHLRC2* mutant macrophages (Fig. S3D).

To investigate the potential thioredoxin activity of NHLRC2, given that NHLRC2 contains an N-terminal Trx-like domain, we generated a construct containing full-length human NHLRC and expressed the protein in 293FT cells. The purified protein was tested in a thioredoxin reductase (TrxR) assay but showed no significant thioredoxin activity compared to the positive control, suggesting that NHLRC2 might not be involved in thiol-disulfide exchange (see Fig. S4 in the supplemental material).

10.1128/mBio.02169-19.5 FIG S4 Purified NHLRC2 proteins did not exhibit thioredoxin activity in a thioredoxin reductase assay. (A) Map of construct for NHLRC2 protein purification from mammalian cells. (B and C) Purified NHLRC2 protein was identified by Coomassie staining (B) and immunoblotting (C) with NHLRC2 antibody. (D) Purified NHLRC2 protein was used in a thioredoxin reductase assay. A result from a positive control with or without TrxR inhibitor (Inh) is also presented. The results shown are the average absorbance at 412 nm of various amounts of purified NHLRC2 versus the positive control ± SD. Download FIG S4, PDF file, 0.1 MB. Copyright © 2019 Yeung et al. 2019 Yeung et al. This content is distributed under the terms of the Creative Commons Attribution 4.0 International license.

**FIG 7:**
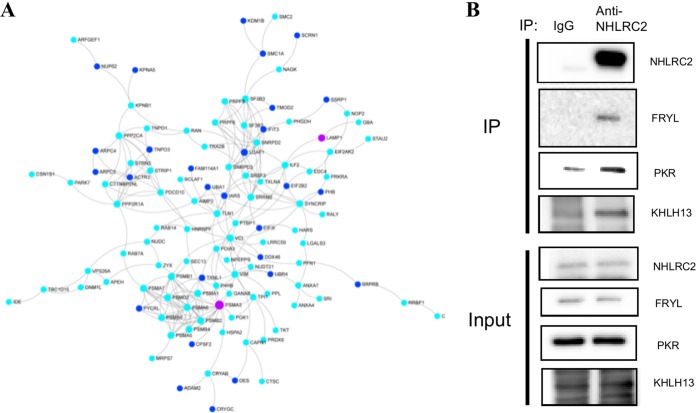
Protein-protein interaction networks and co-IP of NHLRC2 and its interactors. (A) NetworkAnalyst was utilized to construct a zero-order (direct interactors only) protein-protein interaction (based on the InnateDB database) network from 82 of the 191 proteins (turquoise nodes) identified to interact with NHLRC2 and 30 of the 183 host factors (dark blue) identified by CRISPR/Cas9 to influence *Salmonella* infection of macrophages. There were 3 overlapping proteins common to both data sets (NHLRC2, PSMA3, and LAMP1 [magenta nodes]); NHLRC2 does not appear but interacts with all turquoise nodes shown. (B) A co-IP experiment was performed to confirm protein interactors of NHLRC2 identified from the IP-MS experiment. IgG antibody was used as a control.

Recently, a study by Paakkola et al. showed in a HEK cell model that NHLRC2 interacts with proteins involved in cytosolic processes, including cell-cell adhesion, cell division, and intracellular protein transport (42). Here, we utilized immunoprecipitation coupled to mass spectrometry (IP-MS) to identify proteins that interacted with NHLRC2 in THP-1 cells. Selected protein-protein interactions (PPIs) were validated by coimmunoprecipitation (co-IP). First, we confirmed the specificity of an anti-NHLRC2 antibody in THP-1 cells using IP-MS. Subsequently, we used the anti-NHLRC2 antibody to perform IP-MS using whole-cell lysates of WT and *NHLRC2* mutant THP-1 cells. IgG antibody was used as a negative control for pulldowns. After applying a manual thresholding approach of removing all proteins with quantitative value of >0 in the IgG control samples, MS revealed 191 proteins were coimmunoprecipitated (see Table S5 in the supplemental material), none of which were known previously to interact with NHLRC2. Pathway enrichment analysis using Reactome revealed the interactors of NHLRC2 were involved in biological processes, including cell cycle, extracellular matrix organization, programmed cell death, immune system, and signal transduction. Intriguingly we were able to observe an excellent zero-order (direct interactors only) protein-protein interaction network of 110 proteins (Fig. 7A), comprising 80 NHLRC2 interactors interacting with 30 host factors for *Salmonella* uptake (from Table S2). There were 3 overlapping proteins common to both data sets (NHLRC2, PSMA3, and LAMP1). This indicates that NHLRC2 and its interactors have strong functional overlaps with *Salmonella* uptake proteins.

10.1128/mBio.02169-19.10 TABLE S5 List of proteins coprecipitated with NHLRC2 as detected by mass spectrometry. Results are presented as normalized quantitative values as determined by Scaffold proteome software. The following parameters were used for quantitative analysis: a protein threshold of 99.0%, minimum number of peptides of 2, and peptide threshold of 95%. Download Table S5, DOCX file, 0.1 MB. Copyright © 2019 Yeung et al. 2019 Yeung et al. This content is distributed under the terms of the Creative Commons Attribution 4.0 International license.

Subsequently, 3 potential interactors of NHLRC2 were validated by coimmunoprecipitation (co-IP): FRYL (FRY like transcription coactivator; involved in actin cytoskeleton regulation), EIF2AK2 (eukaryotic translation initiation factor 2 alpha kinase 2; involved in cell proliferation and differentiation), and KLHL13 (Kelch like family member 13; involved in mitotic progression) (Fig. 7B). Interestingly, MS revealed the protein expression levels for these 3 validated NHLRC2 interactors were lower in the *NHLRC2* mutant than in the WT THP-1 cells.

## DISCUSSION

In this study, we have shown that the uptake of *Salmonella* into macrophages is a complex process requiring coordination of hundreds of host genes and is substantially distinct from phagocytosis of paramagnetic beads. Moreover, the complexity of *Salmonella* uptake in macrophages is contributed by the ability of the bacteria to mediate their uptake into the macrophages via the *Salmonella* SPI-1-dependent invasion process. We discuss here some biological features of the essential genes, but importantly our broad screen captured strong biological themes based on pathway and network analysis (Fig. 2 and Fig. 7; Table S2, part B).

The seven-subunit Arp2/3 complex plays a major role in the regulation of the eukaryotic actin cytoskeleton. It facilitates the rapid growth of actin filaments to generate actin networks and functions during dynamic cellular events, such as cell motility, phagocytosis, endocytosis, chemotaxis, and membrane trafficking (43). It is known that certain pathogens, including Shigella flexneri, Listeria monocytogenes, and E. coli, exploit the Arp2/3 complex to promote attachment, internalization, or cell-cell spread (44, 45). Interestingly the role of the Arp2/3 complex in *Salmonella* invasion of host cells has been controversial. While one group showed the Arp2/3 complex played a key role in actin reorganization required for *Salmonella* internalization into polarized epithelial cells (17), another group uncovered an Arp2/3 complex-independent *Salmonella* invasion mechanism based on myosin II (19). Here, we found that the Arp2/3 complex is required for *Salmonella* invasion of human macrophages. Independent targeted mutations in 3 Arp2/3 complex subunits in THP-1 macrophages all resulted in strong resistance to *Salmonella* invasion. Consistent with this, a small-molecule inhibitor, CK-666, targeting the Arp2/3 complex led to modestly decreased *Salmonella* uptake. We suspect the milder effects of the Arp2/3 inhibitor CK-666 on *Salmonella* invasion, as opposed to the strong inhibition observed for the Arp2/3 complex knockout mutants, may be due to incomplete inhibition by CK-666.

The screen also identified an HMG-CoA reductase (HMGCR), which is involved in the rate-limiting step in cholesterol biosynthesis (46). Statins are used in the clinic to inhibit HMG-CoA reductase to reduce cholesterol levels in serum. Intriguingly, patients receiving statin therapy have reduced mortality associated with several bacterial infections (47–49). Here, we showed that HMGCR mutant macrophages exhibited significant resistance to *Salmonella* infection, while treatment of macrophages with the statin drug fluvastatin led to reduced *Salmonella* uptake. Moreover, treatment with the other cholesterol inhibitors Mβ-CD and U18666A also decreased *Salmonella* uptake. Thus, our results suggest that cholesterol inhibitors could be investigated for use in the clinic against *Salmonella* infections.

An *NHLRC2* mutant exhibited strong (>85%) resistance to *Salmonella* infection. We showed by flow cytometry and gene expression studies many of the key receptors involved in pathogen recognition and interaction, such as TLR4, ITGAM, FCGRIIA, FCGRIII, and DECTIN-1, were lacking in the *NHLRC2* mutant macrophages. As many of these receptors are involved in interaction with other bacteria, it was not surprising when we also observed strong resistance of *NHLRC2* mutant macrophages to other microbes.

A previous study by Haney et al. indicated NHLRC2 as a key regulator in paramagnetic particle phagocytosis and suggested it acted through controlling actin polymerization and filopodium formation (16), even though overall there was little overlap in the host factors required for phagocytosis and *Salmonella* uptake. Our study also indicated the involvement of NHLRC2 in actin cytoskeleton regulation, but further demonstrated an exceptionally broad perturbation of cellular functions with broad interactions between 80 NHLRC2 interactions and 30 genes involved in *Salmonella* uptake. Here, microscopy analyses showed marked changes in the cellular morphology of the *NHLRC2* mutant macrophages. Compared with WT macrophages, *NHLRC2* mutant macrophages were smaller and lacked filopodia. In the presence of *Salmonella*, WT macrophages displayed increased membrane ruffling and engaged with *Salmonella* to form phagocytic cups, but the *NHLRC2* mutant macrophage cell surface remained smooth and did not appear to engage with the bacteria in the environment. Transduction of the mutant cells with a *NHLRC2* cDNA restored the morphology (i.e., filopodia) to that of a WT macrophage. When infected with *Salmonella*, the complemented macrophages restored formation of phagocytic cups to engage with the bacteria. Moreover, RNA-Seq revealed the downregulation of several genes involved in actin cytoskeletal arrangements in the *NHLRC2* mutant, and our co-IP experiment confirmed interaction of NHLRC2 with FRYL, which is known to play a role in actin cytoskeleton regulation.

In summary, our screen enabled the identification of 183 host factors involved in *Salmonella*-macrophage interactions that can be investigated as potential therapeutic targets against *Salmonella* infections. This simple screen can be extended to other intracellular pathogens to advance our understanding of host-pathogen interactions.

## MATERIALS AND METHODS

Detailed methods are described in Text S1 in the supplemental material.

10.1128/mBio.02169-19.1 TEXT S1 Detailed descriptions of methods to transduce THP-1 cells, generate Cas9-THP-1 cells, generate genome-wide mutant libraries and screening, perform Western blots, immunoprecipitations, and mass spectrometry, complement an NHLRC2 mutation in Cas9-THP-1 cells, perform RNA-Seq analysis, purify NHLRC2 protein, and perform thioredoxin reductase assays. Download Text S1, DOCX file, 0.1 MB. Copyright © 2019 Yeung et al. 2019 Yeung et al. This content is distributed under the terms of the Creative Commons Attribution 4.0 International license.

### Bacteria and cell line culture.

*S.* Typhimurium SL1344 was transformed with pGFPmut3.1 (6039-1; Clontech), enabling the expression of GFP constitutively under the *lac* operon promoter. The transformed bacteria were routinely grown in L broth or on L agar containing 100 μg/ml ampicillin (69-53-4; Sigma).

The human monocytic-like cell line THP-1 was obtained from the European Collection of Authenticated Cell Cultures (ECACC) and was maintained mycoplasma free. The cell line was routinely cultured in RPMI 1640 supplemented with 2 mM l-glutamine and 10% heat-inactivated fetal calf serum (FCS [F7524; Sigma]). Cells were differentiated into mature macrophages by stimulation with 100 ng/ml phorbol-12-myristate-13-acetate (PMA [P1585; Sigma]) for 48 h and then replaced with fresh medium without PMA for 24 h prior to assay.

### Macrophage infections with *Salmonella.*

On the day of the assay, macrophage dishes were washed with phosphate-buffered saline (PBS), and *S.* Typhimurium SL1344 harboring pGFPmut3.1, grown to mid-log phase, was added to the macrophages at the indicated MOI and incubated at 37°C for 30 min. After incubation, the cells were washed 3× with PBS and incubated for a further 30 min with medium containing 50 μg/ml gentamicin (15710064; Thermo Fisher) to kill extracellular bacteria. For flow cytometry, the infected macrophages were washed and detached from the dish using lidocaine solution prepared as described previously (50) and analyzed on an LSRFortessa flow cytometer (BD). For cytokine analysis, the infection supernatants were collected, filtered, and assayed for a panel of selected cytokines/chemokines customized with the antihuman Milliplex magnetic bead kits (Millipore) and analyzed with Luminex FlexMap3D (Thermo Fisher).

### Generation of genome-wide mutant libraries and screening.

A total of 2.4 × 10^7^ Cas9-THP-1 cells were transduced with the GeCKO v2.0 half library A (13) at an MOI of 0.3 and selected in puromycin for 14 days. Subsequently, the mutant cells were differentiated to macrophages and infected with GFP-expressing *S.* Typhimurium. The infected macrophages were subjected to a flow cytometry cell sorter, and GFP-negative macrophages were collected and extracted for genomic DNA (gDNA). PCR was performed to amplify the gRNA regions, and subsequently sequencing adaptors and barcodes were attached to the samples under the conditions described in 2014 by both Shalem et al. and Sanjana et al. (13, 51), with some modifications. Samples were sequenced on an Illumina HiSeq2500 for 50-bp single-end sequencing. The numbers of reads for each guide were counted with an in-house script. Enrichment of guides and genes was analyzed using MAGeCK statistical package version 0.5.2 (14). See Text S1 for more detail.

### Generation of targeted sgRNA mutants in Cas9-THP-1 cells to validate.

Candidate genes selected from the mutant library screen results were validated by generating targeted mutants in Cas9-THP-1 cells using sgRNAs. Five sgRNAs per candidate gene were designed or chosen. The gRNAs were designed using the web tools at http://crispr.mit.edu and https://sanger.ac.uk/htgt/wge or chosen from the human Brunello (52) or GeCKO v2.0 (13) gRNA libraries. The sgRNAs (IDT) were cloned into the lentiviral gRNA expression vector, pKLV-U6gRNA(BbsI)-PGKpuro2ABFP (53). 293T cells were cotransfected with the pLenti constructs and ViraPower packaging mix according to the manufacturer’s instruction (K497500; Invitrogen). The medium was replaced with fresh medium 24 h after transfection. Viral supernatant was harvested 48 h after transfection and stored at –80°C. Cas9-THP-1 cells were transduced with the viral supernatants as described above and selected in 0.9 μg/ml puromycin (P9620; Sigma) for 2 weeks. Lentivirus expressing the empty lentivirus vector was also transduced into Cas9-THP-1 cells as control. The resultant mixed population of mutants generated from each gRNA was screened for resistance to *S.* Typhimurium using flow cytometry (BD LSRFortessa). Subsequently, 2 gRNAs per candidate gene were chosen, and samples were single-cell sorted into 96-well plates using the FACS Aria II fluorescence-activated cell sorter to achieve 96 clonal mutant lines per gRNA. Clonal mutant lines were further expanded and screened by flow cytometry for resistance to *S.* Typhimurium SL1344 harboring pGFPmut3.1. To confirm the gene mutation, the genomic region harboring the gRNA cutting site was amplified using primers in Table S1 and barcoded by a second PCR to generate the sequencing library (13). Sequencing libraries were submitted for Illumina MiSeq 150-bp paired-end sequencing.

### Fluorescence imagining and scanning electron microscopy.

For confocal imaging, cells were washed, fixed with 4% paraformaldehyde (PFA), permeabilized, and stained with primary antibodies, including anti-GORASP2 mouse monoclonal antibody (AMAb91016; Sigma) or anti-NHLRC2 rabbit polyclonal antibody (HPA038493; Sigma). This was followed by counterstaining with secondary antibodies, including anti-mouse AF647 (A-21235; Thermo Fisher) or anti-rabbit AF488 (A-11008; Thermo Fisher). A conjugated anti-NHLRC2-AF488 antibody (bs-9322R-A488; Bioss Antibodies) was also used. Stained cells were washed and mounted onto slides with ProLong Gold containing DAPI (4′,6-diamidino-2-phenylindole [Invitrogen]). The preparations were visualized with an LSM510 META confocal microscope (Zeiss). Alternatively for scanning electron microscopy, cells were fixed with a mixture of 2.5% glutaraldehyde and 4% PFA on ice and processed as described previously (54).

### RNA-Seq analysis.

Cas9-THP-1 and *NHLRC2* mutant THP-1 macrophages (1.0 × 10^6^) were infected with *S.* Typhimurium SL1344(pGFPmut3.1) at an MOI of 50 as described above. Three independent infections were performed. After gentamicin treatment, the cells were harvested, and total RNAs from samples of uninfected and *Salmonella-*infected macrophages were purified using the RNAeasy minikit (Qiagen) as per the manufacturer’s instructions. Preparation of the samples for Illumina sequencing was performed as previously described (55). The resultant multiplexed library was sequenced on an Illumina HiSeq2500 using a 75-bp paired-end read length. See Text S1 for more detail.

### Western blot, immunoprecipitations, and mass spectrometry.

For Western blot experiments, cell lysates from Cas9-THP-1 and *NHLRC2* mutant THP-1 macrophages were prepared under denaturing conditions and incubated with the indicated primary and secondary antibodies. See Text S1 for more detail.

For immunoprecipitation experiments, cell lysates from WT and *NHLRC2* mutant THP-1 macrophages were extracted under nondenaturing conditions and incubated with anti-NHLRC2 antibody (HPA038493; Sigma-Aldrich) or IgG isotype control antibody (ab171870; Abcam). Protein A-labeled magnetic beads were used to precipitate NHLRC2, and the bound proteins were purified by a magnetic separator. To confirm the NHLRC2-interacting proteins through coimmunoprecipitation, anti-FRYL (AB95065; AbCam), anti-PKR (AB32506; Abcam), anti-peroxiredoxin 3 (AB129206; Abcam), anti-KHLH13 (MA5-15658; Thermo Fisher Scientific), anti-NHLRC2 (HPA038493; Sigma-Aldrich), and anti-glyceraldehyde-3-phosphate dehydrogenase (anti-GAPDH) (AB9484; Abcam) antibodies were used for the primary reaction, and VeriBlot for IP detection reagent (AB131366; Abcam) was used for the secondary reaction. See Text S1 for more detail.

For mass spectrometry, the eluants obtained from the immunoprecipitation preparation described above were boiled and separated on Tris-glycine gel. The resultant bands were isolated, destained, reduced, alkylated, and digested enzymatically. After digestion, the supernatant was pipetted into a sample vial and loaded onto an autosampler for automated liquid chromatography-tandem mass spectrometry (LC-MS/MS) analysis.

Post-run, all MS/MS data were submitted to the Mascot search algorithm (Matrix Science, London, United Kingdom) and searched against the UniProt human database (71,898 sequences and 24,121,858 residues). All data were then imported into the Scaffold program (version_4.5.4; Proteome Software, Inc., Portland, OR). See Text S1 in the supplemental material for more detail.

### Statistical analyses.

Statistical significance analysis was performed with GraphPad Prism software, and the numbers of replicates are mentioned in the associated figure legends. Differences were considered significant when *P* is <0.05.

### Data availability.

The CRISPR data reported in the paper have been deposited in the European Genome-phenome Archive (http://www.ebi.ac.uk/ega) under study accession no. EGAS00001001256. The RNA-Seq data have been deposited in the European Nucleotide Archive (http://www.ebi.ac.uk/ena) under study accession no. PRJEB6662.
