# Supplementary figures and images for: A Genome-Wide Knockout Screen in Human Macrophages Identified Host Factors Modulating Salmonella Infection
Source: mBio. 2019 Oct 8;10(5):e02169-19. doi: 10.1128/mBio.02169-19 (PMC6786873; doi:10.1128/mBio.02169-19)

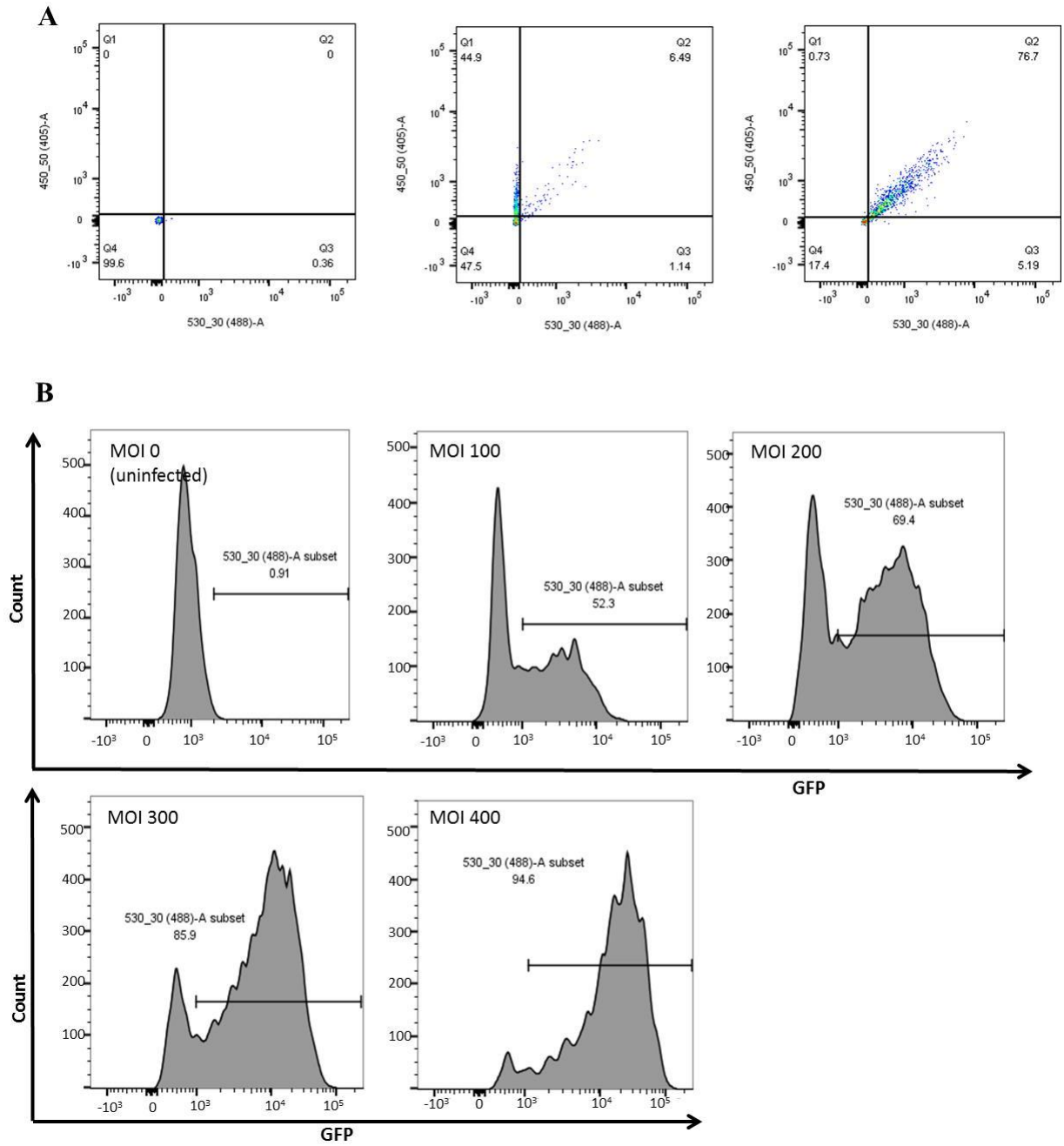

**Fig. S1: Cas9 functional assay and *Salmonella* infection of Cas9-THP-1 macrophages.**

Supplement: FIG S1 [file mBio.02169-19-sf001.pdf]

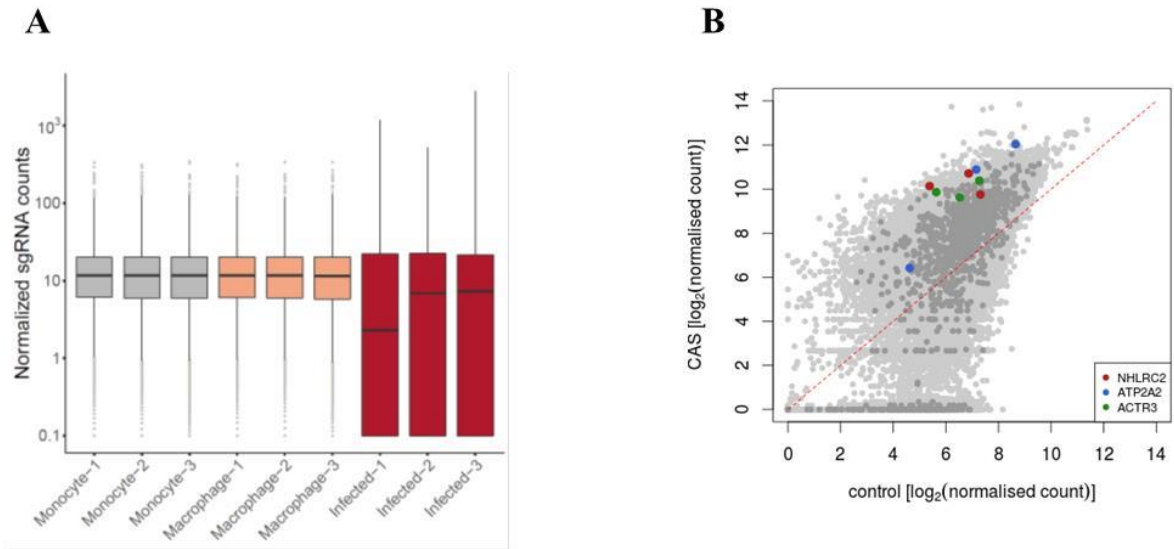

**Fig. S2. Deep sequencing analyses of sgRNAs in the THP-1-GeCKO library.**

Supplement: FIG S2 [file mBio.02169-19-sf002.pdf]
