# Supplementary material for: A Genome-Wide Knockout Screen in Human Macrophages Identified Host Factors Modulating Salmonella Infection
Source: mBio. 2019 Oct 8;10(5):e02169-19. doi: 10.1128/mBio.02169-19 (PMC6786873; doi:10.1128/mBio.02169-19)
Supplement: TABLE S1 [file mBio.02169-19-st001.docx]

| **Supplementary Table 1: Primer or gRNA sequences used in this study** | |
| --- | --- |
|  |  |
| **Primer sequences for PCR amplification for Gibson assembly to generate NHLRC2 complementation construct (5’-3’)** | |
| **Fr1-F** | cgcccagagcagcGCGGCCGTTAATGATATCTATAAC |
| **Fr1-R** | agaggcccgcaccGATCGCCCTTCCCAACAG |
| **Fr2-F** | gggaagggcgatcGGTGCGGGCCTCTTCGCT |
| **Fr2-R** | cgggcaccggagcAAGTGGCACCGAGTCGGTG |
| **Fr3-F** | ctcggtgccacttGCTCCGGTGCCCGTCAGT |
| **Fr3-R** | cgggcgccgccatTCACGACACCTGAAATGGAAGAAAAAAACTTTGAAC |
| **Fr4-F** | tcaggtgtcgtgaATGGCGGCGCCCGGAGGC |
| **Fr4-R** | ttcctctgccctcAAATACATACCTGAGCTCTACTGGAGCTATGCAACCTTGCTGTG |
| **Fr5-F** | caggtatgtatttGAGGGCAGAGGAAGTCTC |
| **Fr5-R** | ttaatgatatctaTCAGCGAGCTCTCTTGTAC |
| **Fr6-F** | gagagctcgctgaTAGATATCATTAACGGCCGCCCATAG |
| **Fr6-R** | cattaacggccgcGCTGCTCTGGGCGTGGTT |
|  |  |
| **Primer sequences for PCR confirmation of NHLRC2 complementation by Sanger sequencing (5’-3’)** | |
| **seq1-F** | CAGGGGGCAGATCAGAGATA |
| **seq2-F** | AGATATGGGCACTCCTGCTG |
| **seq3-F** | AATGAACCAGGAGGCTTGTG |
| **seq4-F** | AGGCAATTTTGTTCAGTCAGC |
| **seq5-F** | CTACGAGGGCACCCAGAC |
| **seq6-F** | AGGACGGCGAGTTCATCTAC |
| **seq7-F** | GATGCTCTTCGTCCAGATCA |
|  |  |
| **Primer sequences for PCR amplification of gRNAs from libraries or enriched mutants for Illumina Hi-Seq (5’-3’)** | |
| **GeCKOv2 F1** | aatggactatcatatgcttaccgtaacttgaaagtatttcg |
| **GeCKOv2 R1** | ctttagtttgtatgtctgttgctattatgtctactattctttcc |
|  |  |
| **Primer sequences for PCR amplification of gRNAs for Mi-Seq (5’-3’)** | |
| **ACTR3 A5 MISEQ_F** | tcttgtggaaaggacgaaacaccgCGTCCTCTCTACAAGGTATTTATAGC |
| **ACTR3 A5 MISEQ_R** | *TCTACTATTCTTTCCCCTGCACTGT*GGAAAACCAACCTTCAATCTACC |
| **ARPC4 A10 MISEQ_F** | tcttgtggaaaggacgaaacaccgCAACTTCCACACAGAGCAGATGTACAAAC |
| **ARPC4 A10 MISEQ_R** | *TCTACTATTCTTTCCCCTGCACTGT*CTAGAGAGCTCAGGAGATATAAACACACTG |
| **ATP2B2 B3 MISEQ_F** | tcttgtggaaaggacgaaacaccgAAGATATTGATGCTTAGAATTGTAGC |
| **ATP2B2 B3 MISEQ_R** | *TCTACTATTCTTTCCCCTGCACTGT*GCCAGTAATAAAATCCTAACTAGCAAG |
| **CYFIP2 B8 MISEQ_F** | tcttgtggaaaggacgaaacaccgGTGAATTGTAACTCCGTAAATCTGTTAAAA |
| **CYFIP2 B8 MISEQ_R** | *TCTACTATTCTTTCCCCTGCACTGT*ATGAGTATGACACCTGATGCTCCC |
| **CTLCL1 B10 MISEQ_F** | tcttgtggaaaggacgaaacaccgAAGATTCCTTTCTAACAATCCCATCG |
| **CTLCL1 B10 MISEQ_R** | *TCTACTATTCTTTCCCCTGCACTGT*CTACAGTCAGTCCATTTCAGAGGAA |
| **PDGFB C10 MISEQ_F** | tcttgtggaaaggacgaaacaccgGTCTGACTGTGACTTCTCCTGCAGA |
| **PDGFB C10 MISEQ_R** | *TCTACTATTCTTTCCCCTGCACTGT*GCATTTAAGGATGGTTTTACTCTCTCAG |
| **HMGCR D3 MISEQ_F** | tcttgtggaaaggacgaaacaccgGAATATTTCATGCAGTTTAATATGTGTGTG |
| **HMGCR D3 MISEQ_R** | *TCTACTATTCTTTCCCCTGCACTGT*GATAAATTATCCATCCAGTCACTCAC |
| **CD27 D8 MISEQ_F** | tcttgtggaaaggacgaaacaccgGAAGGGTTTGGAAGAGGATCACACTC |
| **CD27 D8 MISEQ_R** | *TCTACTATTCTTTCCCCTGCACTGT*CTTGAAGGTCTCCACAGGTCTGAGT |
| **NHLRC2 E1 MISEQ_F** | tcttgtggaaaggacgaaacaccgGTATGACTAGAGTTGGCCAGCAG |
| **NHLRC2 E1 MISEQ_R** | *TCTACTATTCTTTCCCCTGCACTGT*AGTTGAAGATCCCAGCGTAGTTC |
| **B3GNT1 E11 MISEQ_F** | tcttgtggaaaggacgaaacaccgTACCTCTGTAAAGTGGAGCGACATTTCTTA |
| **B3GNT1 E11 MISEQ_R** | *TCTACTATTCTTTCCCCTGCACTGT*GGGTTTGATTTTGAGGTCCTGAACGAAG |
| **ITPR3 F11 MISEQ_F** | tcttgtggaaaggacgaaacaccgCGTTCCCGCTCCTTGAGACTAGG |
| **ITPR3 F11 MISEQ_R** | *TCTACTATTCTTTCCCCTGCACTGT*CCGCAGCCATGAGTGAAATGTCC |
| **CTTN G8 MISEQ_F** | tcttgtggaaaggacgaaacaccgAGGAGAGTTAAACATGCCTTCCAAG |
| **CTTN G8 MISEQ_R** | *TCTACTATTCTTTCCCCTGCACTGT*CTCCCTCCTTCCTCTATAGTGACCA |
| **TOR3A H5 MISEQ_F** | tcttgtggaaaggacgaaacaccgGAGAAACAGAAAGATAGTCCATGGAGACTC |
| **TOR3A H5 MISEQ_R** | *TCTACTATTCTTTCCCCTGCACTGT*CACTTCCCCAGCTGCTCCCTATATC |
| **CAPZB H9 MISEQ_F** | tcttgtggaaaggacgaaacaccgGAGTCCCCATCTCTGTTGTAGTCACA |
| **CAPZB H9 MISEQ_R** | *TCTACTATTCTTTCCCCTGCACTGT*GAAAAGAAAAGAACAGGCCCATAATTTTG |
