# Supplementary material for: A Genome-Wide Knockout Screen in Human Macrophages Identified Host Factors Modulating Salmonella Infection
Source: mBio. 2019 Oct 8;10(5):e02169-19. doi: 10.1128/mBio.02169-19 (PMC6786873; doi:10.1128/mBio.02169-19)
Supplement: TABLE S2 [file mBio.02169-19-st002.docx]

| **Supplementary Tables 2A: List of candidate genes identified from *Salmonella-*macrophage genome-wide CRISPR screen.**  **Supplementary Table 2B: Table S2B: List of over-represented pathways identified for the candidate genes using InnateDB.** Only pathways with corrected p-value<0.5 are shown.    **Table S2A: List of candidate genes identified from *Salmonella-*macrophage genome-wide CRISPR screen.** | | | | | | | | | | | | | | |  | | |  |  |  |  |  |  |
| --- | --- | --- | --- | --- | --- | --- | --- | --- | --- | --- | --- | --- | --- | --- | --- | --- | --- | --- | --- | --- | --- | --- | --- |
|  |  |  |  |  | |  |  |  |  |  |  |  |  |  |  |  |  |  |  |  |  |  |  |
| **sgrna** | **Rep- licate** | **Gene** | **Rep. Count** | | **Control count** | **Treatment count** | **Control mean** | **Treat mean** | **Control var** | **Adj var** | **Score** | **P low** | **P high** | **P two- sided** | **FDR** | **High in treat-ment** |  |  |  |  |  |  |  |
| HGLibA_03608 | Rep 1 | ATP2A2 | 2 | | 228.03 | 2451.3 | 228.03 | 2451.3 | 3.17E+06 | 89055 | 7.4501 | 1 | 6.00E-14 | 1.20E-13 | 1.09E-12 | TRUE |  |  |  |  |  |  |  |
| HGLibA_03607 | Rep 1 | ATP2A2 | 2 | | 77.301 | 1200.1 | 77.301 | 1200.1 | 8.53E+05 | 9252.2 | 11.673 | 1 | 1.11E-31 | 2.22E-31 | 3.61E-30 | TRUE |  |  |  |  |  |  |  |
| HGLibA_03608 | Rep 2 | ATP2A2 | 3 | | 1346.9 | 10618 | 1346.9 | 10618 | 5.27E+07 | 8.88E+05 | 9.8405 | 1 | 4.08E-23 | 8.16E-23 | 2.17E-21 | TRUE |  |  |  |  |  |  |  |
| HGLibA_03607 | Rep 2 | ATP2A2 | 3 | | 533.87 | 4286.1 | 533.87 | 4286.1 | 8.65E+06 | 1.63E+05 | 9.2939 | 1 | 8.20E-21 | 1.64E-20 | 3.85E-19 | TRUE |  |  |  |  |  |  |  |
| HGLibA_03609 | Rep 2 | ATP2A2 | 3 | | 126.96 | 656.73 | 126.96 | 656.73 | 1.62E+05 | 11800 | 4.877 | 1 | 6.13E-07 | 1.23E-06 | 9.59E-06 | TRUE |  |  |  |  |  |  |  |
| HGLibA_03609 | Rep 3 | ATP2A2 | 3 | | 0.62217 | 36.567 | 0.62217 | 36.567 | 1013.2 | 1.0954 | 34.344 | 1 | 1.19E-258 | 2.37E-258 | 1.86E-256 | TRUE |  |  |  |  |  |  |  |
| HGLibA_03607 | Rep 3 | ATP2A2 | 3 | | 98.613 | 609.45 | 98.613 | 609.45 | 1.54E+05 | 4123.5 | 7.9552 | 1 | 9.54E-16 | 1.91E-15 | 2.99E-14 | TRUE |  |  |  |  |  |  |  |
| HGLibA_03608 | Rep 3 | ATP2A2 | 3 | | 303.31 | 1572.4 | 303.31 | 1572.4 | 9.28E+05 | 30248 | 7.2969 | 1 | 1.53E-13 | 3.07E-13 | 4.24E-12 | TRUE |  |  |  |  |  |  |  |
| HGLibA_31899 | Rep 1 | NHLRC2 | 3 | | 67.176 | 459.62 | 67.176 | 459.62 | 92364 | 6900.7 | 4.7242 | 1 | 1.46E-06 | 2.92E-06 | 1.60E-05 | TRUE |  |  |  |  |  |  |  |
| HGLibA_31900 | Rep 1 | NHLRC2 | 3 | | 65.768 | 459.62 | 65.768 | 459.62 | 93322 | 6602.1 | 4.8472 | 1 | 7.92E-07 | 1.58E-06 | 8.87E-06 | TRUE |  |  |  |  |  |  |  |
| HGLibA_31898 | Rep 1 | NHLRC2 | 3 | | 19.457 | 536.22 | 19.457 | 536.22 | 1.95E+05 | 526.81 | 22.515 | 1 | 1.86E-112 | 3.72E-112 | 1.33E-110 | TRUE |  |  |  |  |  |  |  |
| HGLibA_31898 | Rep 2 | NHLRC2 | 3 | | 119.86 | 3207.8 | 119.86 | 3207.8 | 6.95E+06 | 10624 | 29.959 | 1 | 1.93E-197 | 3.86E-197 | 1.01E-194 | TRUE |  |  |  |  |  |  |  |
| HGLibA_31900 | Rep 2 | NHLRC2 | 3 | | 431.35 | 7772.8 | 431.35 | 7772.8 | 3.73E+07 | 1.10E+05 | 22.102 | 1 | 1.66E-108 | 3.32E-108 | 4.58E-106 | TRUE |  |  |  |  |  |  |  |
| HGLibA_31899 | Rep 2 | NHLRC2 | 3 | | 643.48 | 2961.1 | 643.48 | 2961.1 | 3.04E+06 | 2.29E+05 | 4.8384 | 1 | 7.19E-07 | 1.44E-06 | 1.12E-05 | TRUE |  |  |  |  |  |  |  |
| HGLibA_31898 | Rep 3 | NHLRC2 | 2 | | 41.27 | 597.26 | 41.27 | 597.26 | 2.07E+05 | 890.52 | 18.631 | 1 | 9.75E-78 | 1.95E-77 | 1.01E-75 | TRUE |  |  |  |  |  |  |  |
| HGLibA_31900 | Rep 3 | NHLRC2 | 2 | | 76.734 | 938.55 | 76.734 | 938.55 | 4.86E+05 | 2648 | 16.748 | 1 | 3.15E-63 | 6.31E-63 | 2.79E-61 | TRUE |  |  |  |  |  |  |  |
| HGLibA_00659 | Rep 1 | ACTR3 | 3 | | 45.87 | 383.01 | 45.87 | 383.01 | 70226 | 3114.4 | 6.0413 | 1 | 9.62E-10 | 1.92E-09 | 1.37E-08 | TRUE |  |  |  |  |  |  |  |
| HGLibA_00660 | Rep 1 | ACTR3 | 3 | | 63.831 | 740.49 | 63.831 | 740.49 | 2.97E+05 | 6202.7 | 8.5918 | 1 | 5.41E-18 | 1.08E-17 | 1.18E-16 | TRUE |  |  |  |  |  |  |  |
| HGLibA_00658 | Rep 1 | ACTR3 | 3 | | 23.507 | 485.15 | 23.507 | 485.15 | 1.50E+05 | 777.98 | 16.551 | 1 | 9.85E-62 | 1.97E-61 | 4.99E-60 | TRUE |  |  |  |  |  |  |  |
| HGLibA_00658 | Rep 2 | ACTR3 | 2 | | 93.053 | 3673.1 | 93.053 | 3673.1 | 9.78E+06 | 6696.5 | 43.748 | 1 | 0 | 0 | 0 | TRUE |  |  |  |  |  |  |  |
| HGLibA_00659 | Rep 2 | ACTR3 | 2 | | 329.63 | 2017.7 | 329.63 | 2017.7 | 1.68E+06 | 67446 | 6.5002 | 1 | 4.47E-11 | 8.94E-11 | 1.09E-09 | TRUE |  |  |  |  |  |  |  |
| HGLibA_00660 | Rep 3 | ACTR3 | 2 | | 105.98 | 694.77 | 105.98 | 694.77 | 2.07E+05 | 4683.2 | 8.6038 | 1 | 4.10E-18 | 8.21E-18 | 1.45E-16 | TRUE |  |  |  |  |  |  |  |
| HGLibA_00659 | Rep 3 | ACTR3 | 2 | | 74.764 | 450.99 | 74.764 | 450.99 | 83323 | 2529.3 | 7.4809 | 1 | 3.96E-14 | 7.93E-14 | 1.14E-12 | TRUE |  |  |  |  |  |  |  |
| HGLibA_03086 | Rep 1 | ARPC4 | 3 | | 215.18 | 1506.5 | 215.18 | 1506.5 | 1.00E+06 | 78859 | 4.5985 | 1 | 2.73E-06 | 5.47E-06 | 2.90E-05 | TRUE |  |  |  |  |  |  |  |
| HGLibA_03087 | Rep 1 | ARPC4 | 3 | | 73.956 | 868.17 | 73.956 | 868.17 | 4.10E+05 | 8435.3 | 8.6474 | 1 | 3.34E-18 | 6.67E-18 | 7.32E-17 | TRUE |  |  |  |  |  |  |  |
| HGLibA_03085 | Rep 1 | ARPC4 | 3 | | 26.589 | 485.15 | 26.589 | 485.15 | 1.46E+05 | 1003.6 | 14.475 | 1 | 1.09E-47 | 2.19E-47 | 4.63E-46 | TRUE |  |  |  |  |  |  |  |
| HGLibA_03085 | Rep 2 | ARPC4 | 2 | | 496.02 | 2972.6 | 496.02 | 2972.6 | 3.61E+06 | 1.42E+05 | 6.5614 | 1 | 2.94E-11 | 5.88E-11 | 7.25E-10 | TRUE |  |  |  |  |  |  |  |
| HGLibA_03086 | Rep 2 | ARPC4 | 2 | | 1790.9 | 7699.6 | 1790.9 | 7699.6 | 1.96E+07 | 1.50E+06 | 4.8308 | 1 | 7.32E-07 | 1.47E-06 | 1.14E-05 | TRUE |  |  |  |  |  |  |  |
| HGLibA_03086 | Rep 3 | ARPC4 | 2 | | 365.83 | 1462.7 | 365.83 | 1462.7 | 6.68E+05 | 42218 | 5.3382 | 1 | 4.88E-08 | 9.75E-08 | 8.72E-07 | TRUE |  |  |  |  |  |  |  |
| HGLibA_03087 | Rep 3 | ARPC4 | 2 | | 74.038 | 316.91 | 74.038 | 316.91 | 33068 | 2486.2 | 4.871 | 1 | 5.96E-07 | 1.19E-06 | 9.42E-06 | TRUE |  |  |  |  |  |  |  |
| HGLibA_51030 | Rep 1 | TOR3A | 2 | | 20.602 | 127.67 | 20.602 | 127.67 | 6772.2 | 592.61 | 4.3983 | 1 | 6.81E-06 | 1.36E-05 | 6.98E-05 | TRUE |  |  |  |  |  |  |  |
| HGLibA_51029 | Rep 1 | TOR3A | 2 | | 30.287 | 331.95 | 30.287 | 331.95 | 58546 | 1314.4 | 8.3207 | 1 | 5.48E-17 | 1.10E-16 | 1.14E-15 | TRUE |  |  |  |  |  |  |  |
| HGLibA_51029 | Rep 2 | TOR3A | 2 | | 122.23 | 1795.4 | 122.23 | 1795.4 | 1.88E+06 | 11010 | 15.946 | 1 | 1.73E-57 | 3.46E-57 | 2.45E-55 | TRUE |  |  |  |  |  |  |  |
| HGLibA_51030 | Rep 2 | TOR3A | 2 | | 233.42 | 1390.6 | 233.42 | 1390.6 | 7.87E+05 | 35882 | 6.1088 | 1 | 5.63E-10 | 1.13E-09 | 1.23E-08 | TRUE |  |  |  |  |  |  |  |
| HGLibA_51031 | Rep 3 | TOR3A | 2 | | 17.524 | 268.16 | 17.524 | 268.16 | 42414 | 201.42 | 17.66 | 1 | 4.80E-70 | 9.59E-70 | 4.48E-68 | TRUE |  |  |  |  |  |  |  |
| HGLibA_51029 | Rep 3 | TOR3A | 2 | | 27.168 | 134.08 | 27.168 | 134.08 | 6533.9 | 429.6 | 5.1581 | 1 | 1.38E-07 | 2.76E-07 | 2.35E-06 | TRUE |  |  |  |  |  |  |  |
| HGLibA_02594 | Rep 1 | AQP3 | 3 | | 26.325 | 434.08 | 26.325 | 434.08 | 1.13E+05 | 983.11 | 13.005 | 1 | 7.19E-39 | 1.44E-38 | 2.68E-37 | TRUE |  |  |  |  |  |  |  |
| HGLibA_02593 | Rep 1 | AQP3 | 3 | | 18.577 | 306.41 | 18.577 | 306.41 | 56539 | 478.95 | 13.152 | 1 | 1.03E-39 | 2.06E-39 | 3.89E-38 | TRUE |  |  |  |  |  |  |  |
| HGLibA_02592 | Rep 1 | AQP3 | 3 | | 20.074 | 459.62 | 20.074 | 459.62 | 1.38E+05 | 561.74 | 18.545 | 1 | 5.55E-77 | 1.11E-76 | 3.24E-75 | TRUE |  |  |  |  |  |  |  |
| HGLibA_02593 | Rep 3 | AQP3 | 2 | | 63.772 | 390.05 | 63.772 | 390.05 | 62791 | 1911.4 | 7.4629 | 1 | 4.56E-14 | 9.12E-14 | 1.30E-12 | TRUE |  |  |  |  |  |  |  |
| HGLibA_02592 | Rep 3 | AQP3 | 2 | | 69.579 | 329.1 | 69.579 | 329.1 | 38282 | 2228.4 | 5.4977 | 1 | 2.07E-08 | 4.14E-08 | 3.82E-07 | TRUE |  |  |  |  |  |  |  |
| HGLibA_02927 | Rep 1 | ARL11 | 2 | | 59.693 | 434.08 | 59.693 | 434.08 | 84829 | 5393 | 5.0981 | 1 | 2.17E-07 | 4.33E-07 | 2.55E-06 | TRUE |  |  |  |  |  |  |  |
| HGLibA_02926 | Rep 1 | ARL11 | 2 | | 65.416 | 536.22 | 65.416 | 536.22 | 1.37E+05 | 6528.5 | 5.8269 | 1 | 3.57E-09 | 7.14E-09 | 4.88E-08 | TRUE |  |  |  |  |  |  |  |
| HGLibA_02928 | Rep 2 | ARL11 | 3 | | 302.03 | 9527.1 | 302.03 | 9527.1 | 6.33E+07 | 57477 | 38.479 | 1 | 0 | 0 | 0 | TRUE |  |  |  |  |  |  |  |
| HGLibA_02927 | Rep 2 | ARL11 | 3 | | 337.51 | 2190 | 337.51 | 2190 | 2.04E+06 | 70427 | 6.9803 | 1 | 1.64E-12 | 3.28E-12 | 4.49E-11 | TRUE |  |  |  |  |  |  |  |
| HGLibA_02926 | Rep 2 | ARL11 | 3 | | 518.1 | 2710.5 | 518.1 | 2710.5 | 2.77E+06 | 1.54E+05 | 5.5814 | 1 | 1.32E-08 | 2.63E-08 | 2.52E-07 | TRUE |  |  |  |  |  |  |  |
| HGLibA_05152 | Rep 1 | C11orf52 | 3 | | 12.678 | 102.14 | 12.678 | 102.14 | 4914.3 | 219.17 | 6.0427 | 1 | 9.42E-10 | 1.88E-09 | 1.34E-08 | TRUE |  |  |  |  |  |  |  |
| HGLibA_05151 | Rep 1 | C11orf52 | 3 | | 23.595 | 204.27 | 23.595 | 204.27 | 20273 | 784.01 | 6.4528 | 1 | 6.86E-11 | 1.37E-10 | 1.05E-09 | TRUE |  |  |  |  |  |  |  |
| HGLibA_05150 | Rep 1 | C11orf52 | 3 | | 59.605 | 561.75 | 59.605 | 561.75 | 1.59E+05 | 5376.4 | 6.8483 | 1 | 4.72E-12 | 9.43E-12 | 7.73E-11 | TRUE |  |  |  |  |  |  |  |
| HGLibA_05152 | Rep 3 | C11orf52 | 2 | | 28.309 | 255.97 | 28.309 | 255.97 | 32401 | 461.42 | 10.598 | 1 | 1.67E-26 | 3.35E-26 | 8.19E-25 | TRUE |  |  |  |  |  |  |  |
| HGLibA_05151 | Rep 3 | C11orf52 | 2 | | 39.3 | 182.83 | 39.3 | 182.83 | 11681 | 817.5 | 5.0201 | 1 | 2.82E-07 | 5.64E-07 | 4.63E-06 | TRUE |  |  |  |  |  |  |  |
| HGLibA_05990 | Rep 1 | C20orf144 | 2 | | 57.052 | 612.82 | 57.052 | 612.82 | 1.98E+05 | 4907.2 | 7.9338 | 1 | 1.34E-15 | 2.68E-15 | 2.64E-14 | TRUE |  |  |  |  |  |  |  |
| HGLibA_05989 | Rep 1 | C20orf144 | 2 | | 16.728 | 178.74 | 16.728 | 178.74 | 16818 | 386.18 | 8.2443 | 1 | 1.04E-16 | 2.07E-16 | 2.13E-15 | TRUE |  |  |  |  |  |  |  |
| HGLibA_05990 | Rep 2 | C20orf144 | 3 | | 210.55 | 1628.3 | 210.55 | 1628.3 | 1.23E+06 | 29718 | 8.2243 | 1 | 1.10E-16 | 2.21E-16 | 4.06E-15 | TRUE |  |  |  |  |  |  |  |
| HGLibA_05989 | Rep 2 | C20orf144 | 3 | | 205.82 | 1150.2 | 205.82 | 1150.2 | 5.19E+05 | 28509 | 5.5934 | 1 | 1.25E-08 | 2.51E-08 | 2.40E-07 | TRUE |  |  |  |  |  |  |  |
| HGLibA_05991 | Rep 2 | C20orf144 | 3 | | 715.24 | 3408.3 | 715.24 | 3408.3 | 4.13E+06 | 2.78E+05 | 5.1036 | 1 | 1.83E-07 | 3.65E-07 | 3.06E-06 | TRUE |  |  |  |  |  |  |  |
| HGLibA_08316 | Rep 1 | CD27 | 3 | | 29.934 | 229.81 | 29.934 | 229.81 | 24369 | 1282.9 | 5.5804 | 1 | 1.50E-08 | 3.01E-08 | 1.96E-07 | TRUE |  |  |  |  |  |  |  |
| HGLibA_08314 | Rep 1 | CD27 | 3 | | 48.335 | 459.62 | 48.335 | 459.62 | 1.07E+05 | 3473.2 | 6.9787 | 1 | 1.88E-12 | 3.75E-12 | 3.15E-11 | TRUE |  |  |  |  |  |  |  |
| HGLibA_08315 | Rep 1 | CD27 | 3 | | 49.92 | 510.69 | 49.92 | 510.69 | 1.35E+05 | 3714.7 | 7.5599 | 1 | 2.54E-14 | 5.08E-14 | 4.69E-13 | TRUE |  |  |  |  |  |  |  |
| HGLibA_08314 | Rep 3 | CD27 | 2 | | 97.991 | 450.99 | 97.991 | 450.99 | 70558 | 4077.6 | 5.5281 | 1 | 1.73E-08 | 3.45E-08 | 3.21E-07 | TRUE |  |  |  |  |  |  |  |
| HGLibA_08315 | Rep 3 | CD27 | 2 | | 108.67 | 487.56 | 108.67 | 487.56 | 81000 | 4896 | 5.4149 | 1 | 3.26E-08 | 6.53E-08 | 5.93E-07 | TRUE |  |  |  |  |  |  |  |
| HGLibA_10037 | Rep 1 | CLIC6 | 2 | | 36.009 | 408.55 | 36.009 | 408.55 | 89756 | 1882.5 | 8.5864 | 1 | 5.63E-18 | 1.13E-17 | 1.22E-16 | TRUE |  |  |  |  |  |  |  |
| HGLibA_10036 | Rep 1 | CLIC6 | 2 | | 29.054 | 791.56 | 29.054 | 791.56 | 4.24E+05 | 1205.9 | 21.958 | 1 | 4.58E-107 | 9.16E-107 | 3.22E-105 | TRUE |  |  |  |  |  |  |  |
| HGLibA_10036 | Rep 3 | CLIC6 | 3 | | 55.788 | 463.18 | 55.788 | 463.18 | 1.02E+05 | 1510.7 | 10.481 | 1 | 5.69E-26 | 1.14E-25 | 2.75E-24 | TRUE |  |  |  |  |  |  |  |
| HGLibA_10037 | Rep 3 | CLIC6 | 3 | | 84.407 | 377.86 | 84.407 | 377.86 | 48572 | 3132.9 | 5.2428 | 1 | 8.46E-08 | 1.69E-07 | 1.47E-06 | TRUE |  |  |  |  |  |  |  |
| HGLibA_10035 | Rep 3 | CLIC6 | 3 | | 41.581 | 182.83 | 41.581 | 182.83 | 11225 | 902.3 | 4.7024 | 1 | 1.40E-06 | 2.80E-06 | 2.11E-05 | TRUE |  |  |  |  |  |  |  |
| HGLibA_10362 | Rep 2 | CNPY1 | 2 | | 430.57 | 2516.4 | 430.57 | 2516.4 | 2.55E+06 | 1.10E+05 | 6.2902 | 1 | 1.76E-10 | 3.51E-10 | 4.03E-09 | TRUE |  |  |  |  |  |  |  |
| HGLibA_10363 | Rep 2 | CNPY1 | 2 | | 359.59 | 1762 | 359.59 | 1762 | 1.12E+06 | 79084 | 4.9869 | 1 | 3.41E-07 | 6.82E-07 | 5.52E-06 | TRUE |  |  |  |  |  |  |  |
| HGLibA_10362 | Rep 3 | CNPY1 | 3 | | 110.95 | 646.02 | 110.95 | 646.02 | 1.68E+05 | 5079.3 | 7.5077 | 1 | 3.20E-14 | 6.40E-14 | 9.29E-13 | TRUE |  |  |  |  |  |  |  |
| HGLibA_10363 | Rep 3 | CNPY1 | 3 | | 96.851 | 560.69 | 96.851 | 560.69 | 1.26E+05 | 3994.1 | 7.3394 | 1 | 1.14E-13 | 2.29E-13 | 3.19E-12 | TRUE |  |  |  |  |  |  |  |
| HGLibA_10361 | Rep 3 | CNPY1 | 3 | | 102.04 | 390.05 | 102.04 | 390.05 | 45804 | 4379.8 | 4.352 | 1 | 7.19E-06 | 1.44E-05 | 9.89E-05 | TRUE |  |  |  |  |  |  |  |
| HGLibA_12035 | Rep 1 | CYFIP2 | 2 | | 52.914 | 536.22 | 52.914 | 536.22 | 1.49E+05 | 4194.1 | 7.4628 | 1 | 5.34E-14 | 1.07E-13 | 9.71E-13 | TRUE |  |  |  |  |  |  |  |
| HGLibA_12033 | Rep 1 | CYFIP2 | 2 | | 28.086 | 331.95 | 28.086 | 331.95 | 60068 | 1124.1 | 9.0629 | 1 | 7.95E-20 | 1.59E-19 | 1.85E-18 | TRUE |  |  |  |  |  |  |  |
| HGLibA_12034 | Rep 3 | CYFIP2 | 3 | | 50.81 | 402.24 | 50.81 | 402.24 | 75696 | 1282.1 | 9.8147 | 1 | 5.28E-23 | 1.06E-22 | 2.28E-21 | TRUE |  |  |  |  |  |  |  |
| HGLibA_12035 | Rep 3 | CYFIP2 | 3 | | 103.18 | 609.45 | 103.18 | 609.45 | 1.50E+05 | 4466.7 | 7.5752 | 1 | 1.91E-14 | 3.82E-14 | 5.60E-13 | TRUE |  |  |  |  |  |  |  |
| HGLibA_12033 | Rep 3 | CYFIP2 | 3 | | 70.409 | 402.24 | 70.409 | 402.24 | 64301 | 2275.4 | 6.9564 | 1 | 1.88E-12 | 3.75E-12 | 4.81E-11 | TRUE |  |  |  |  |  |  |  |
| HGLibA_12721 | Rep 1 | DDX46 | 3 | | 72.635 | 893.7 | 72.635 | 893.7 | 4.41E+05 | 8123.7 | 9.1096 | 1 | 5.23E-20 | 1.05E-19 | 1.23E-18 | TRUE |  |  |  |  |  |  |  |
| HGLibA_12719 | Rep 1 | DDX46 | 3 | | 11.534 | 383.01 | 11.534 | 383.01 | 1.03E+05 | 180.84 | 27.624 | 1 | 3.52E-168 | 7.05E-168 | 3.09E-166 | TRUE |  |  |  |  |  |  |  |
| HGLibA_12720 | Rep 1 | DDX46 | 3 | | 3.7858 | 178.74 | 3.7858 | 178.74 | 23755 | 20.129 | 38.995 | 1 | 0 | 0 | 0 | TRUE |  |  |  |  |  |  |  |
| HGLibA_12721 | Rep 2 | DDX46 | 2 | | 470.78 | 2472.7 | 470.78 | 2472.7 | 2.31E+06 | 1.29E+05 | 5.5634 | 1 | 1.46E-08 | 2.92E-08 | 2.78E-07 | TRUE |  |  |  |  |  |  |  |
| HGLibA_12719 | Rep 2 | DDX46 | 2 | | 248.4 | 1334 | 248.4 | 1334 | 6.82E+05 | 40204 | 5.4144 | 1 | 3.45E-08 | 6.89E-08 | 6.30E-07 | TRUE |  |  |  |  |  |  |  |
| HGLibA_14739 | Rep 2 | EIF2B2 | 2 | | 382.46 | 2581.9 | 382.46 | 2581.9 | 2.90E+06 | 88529 | 7.3923 | 1 | 8.01E-14 | 1.60E-13 | 2.44E-12 | TRUE |  |  |  |  |  |  |  |
| HGLibA_14737 | Rep 2 | EIF2B2 | 2 | | 350.92 | 1555.1 | 350.92 | 1555.1 | 8.17E+05 | 75629 | 4.3786 | 1 | 6.64E-06 | 1.33E-05 | 9.08E-05 | TRUE |  |  |  |  |  |  |  |
| HGLibA_14737 | Rep 3 | EIF2B2 | 3 | | 77.978 | 475.37 | 77.978 | 475.37 | 93105 | 2724.2 | 7.6138 | 1 | 1.43E-14 | 2.85E-14 | 4.21E-13 | TRUE |  |  |  |  |  |  |  |
| HGLibA_14739 | Rep 3 | EIF2B2 | 3 | | 120.7 | 609.45 | 120.7 | 609.45 | 1.37E+05 | 5895.4 | 6.3655 | 1 | 1.03E-10 | 2.07E-10 | 2.31E-09 | TRUE |  |  |  |  |  |  |  |
| HGLibA_14738 | Rep 3 | EIF2B2 | 3 | | 7.6734 | 48.756 | 7.6734 | 48.756 | 999.71 | 49.741 | 5.8251 | 1 | 3.31E-09 | 6.63E-09 | 6.56E-08 | TRUE |  |  |  |  |  |  |  |
| HGLibA_14788 | Rep 1 | EIF3I | 3 | | 29.846 | 204.27 | 29.846 | 204.27 | 18243 | 1275.1 | 4.8848 | 1 | 6.48E-07 | 1.30E-06 | 7.34E-06 | TRUE |  |  |  |  |  |  |  |
| HGLibA_14789 | Rep 1 | EIF3I | 3 | | 18.929 | 204.27 | 18.929 | 204.27 | 22045 | 497.8 | 8.3072 | 1 | 6.11E-17 | 1.22E-16 | 1.27E-15 | TRUE |  |  |  |  |  |  |  |
| HGLibA_14790 | Rep 1 | EIF3I | 3 | | 28.35 | 434.08 | 28.35 | 434.08 | 1.11E+05 | 1146.1 | 11.985 | 1 | 2.68E-33 | 5.36E-33 | 8.98E-32 | TRUE |  |  |  |  |  |  |  |
| HGLibA_14789 | Rep 2 | EIF3I | 2 | | 117.5 | 1077 | 117.5 | 1077 | 5.77E+05 | 10245 | 9.4795 | 1 | 1.46E-21 | 2.91E-21 | 7.10E-20 | TRUE |  |  |  |  |  |  |  |
| HGLibA_14790 | Rep 2 | EIF3I | 2 | | 243.67 | 1873.8 | 243.67 | 1873.8 | 1.62E+06 | 38815 | 8.2742 | 1 | 7.25E-17 | 1.45E-16 | 2.70E-15 | TRUE |  |  |  |  |  |  |  |
| HGLibA_18817 | Rep 1 | GCNT4 | 3 | | 15.407 | 255.34 | 15.407 | 255.34 | 39308 | 326.3 | 13.283 | 1 | 1.81E-40 | 3.63E-40 | 6.93E-39 | TRUE |  |  |  |  |  |  |  |
| HGLibA_18818 | Rep 1 | GCNT4 | 3 | | 16.904 | 434.08 | 16.904 | 434.08 | 1.26E+05 | 394.56 | 21.002 | 1 | 3.91E-98 | 7.81E-98 | 2.66E-96 | TRUE |  |  |  |  |  |  |  |
| HGLibA_18819 | Rep 1 | GCNT4 | 3 | | 8.4521 | 485.15 | 8.4521 | 485.15 | 1.80E+05 | 96.628 | 48.495 | 1 | 0 | 0 | 0 | TRUE |  |  |  |  |  |  |  |
| HGLibA_18817 | Rep 2 | GCNT4 | 2 | | 129.33 | 1699 | 129.33 | 1699 | 1.63E+06 | 12204 | 14.209 | 1 | 4.58E-46 | 9.16E-46 | 5.17E-44 | TRUE |  |  |  |  |  |  |  |
| HGLibA_18819 | Rep 2 | GCNT4 | 2 | | 119.86 | 588.62 | 119.86 | 588.62 | 1.26E+05 | 10624 | 4.5477 | 1 | 3.09E-06 | 6.18E-06 | 4.42E-05 | TRUE |  |  |  |  |  |  |  |
| HGLibA_20354 | Rep 1 | GRM5 | 3 | | 92.797 | 893.7 | 92.797 | 893.7 | 4.05E+05 | 13555 | 6.8791 | 1 | 3.83E-12 | 7.65E-12 | 6.30E-11 | TRUE |  |  |  |  |  |  |  |
| HGLibA_20352 | Rep 1 | GRM5 | 3 | | 31.607 | 331.95 | 31.607 | 331.95 | 57669 | 1436 | 7.9256 | 1 | 1.42E-15 | 2.85E-15 | 2.78E-14 | TRUE |  |  |  |  |  |  |  |
| HGLibA_20353 | Rep 1 | GRM5 | 3 | | 30.903 | 1021.4 | 30.903 | 1021.4 | 7.33E+05 | 1370.4 | 26.755 | 1 | 6.70E-158 | 1.34E-157 | 5.75E-156 | TRUE |  |  |  |  |  |  |  |
| HGLibA_20354 | Rep 2 | GRM5 | 2 | | 782.27 | 9228.9 | 782.27 | 9228.9 | 4.64E+07 | 3.28E+05 | 14.746 | 1 | 1.77E-49 | 3.55E-49 | 2.19E-47 | TRUE |  |  |  |  |  |  |  |
| HGLibA_20353 | Rep 2 | GRM5 | 2 | | 411.64 | 2878.8 | 411.64 | 2878.8 | 3.66E+06 | 1.01E+05 | 7.7526 | 1 | 4.99E-15 | 9.98E-15 | 1.65E-13 | TRUE |  |  |  |  |  |  |  |
| HGLibA_23066 | Rep 1 | IL1RN | 2 | | 49.92 | 485.15 | 49.92 | 485.15 | 1.20E+05 | 3714.7 | 7.141 | 1 | 5.84E-13 | 1.17E-12 | 1.01E-11 | TRUE |  |  |  |  |  |  |  |
| HGLibA_23067 | Rep 1 | IL1RN | 2 | | 26.237 | 280.88 | 26.237 | 280.88 | 41570 | 976.32 | 8.1495 | 1 | 2.29E-16 | 4.57E-16 | 4.62E-15 | TRUE |  |  |  |  |  |  |  |
| HGLibA_23067 | Rep 3 | IL1RN | 3 | | 13.066 | 365.67 | 13.066 | 365.67 | 91000 | 121.91 | 31.935 | 1 | 4.94E-224 | 9.87E-224 | 7.52E-222 | TRUE |  |  |  |  |  |  |  |
| HGLibA_23066 | Rep 3 | IL1RN | 3 | | 88.037 | 511.94 | 88.037 | 511.94 | 1.05E+05 | 3374.6 | 7.2972 | 1 | 1.57E-13 | 3.14E-13 | 4.34E-12 | TRUE |  |  |  |  |  |  |  |
| HGLibA_23068 | Rep 3 | IL1RN | 3 | | 10.058 | 73.134 | 10.058 | 73.134 | 2405.2 | 78.276 | 7.1293 | 1 | 5.78E-13 | 1.16E-12 | 1.54E-11 | TRUE |  |  |  |  |  |  |  |
| HGLibA_26106 | Rep 2 | LCE1C | 3 | | 32.332 | 875.21 | 32.332 | 875.21 | 5.18E+05 | 983.1 | 26.882 | 1 | 2.08E-159 | 4.16E-159 | 8.55E-157 | TRUE |  |  |  |  |  |  |  |
| HGLibA_26107 | Rep 2 | LCE1C | 3 | | 125.38 | 1911.1 | 125.38 | 1911.1 | 2.15E+06 | 11534 | 16.627 | 1 | 2.51E-62 | 5.03E-62 | 3.86E-60 | TRUE |  |  |  |  |  |  |  |
| HGLibA_26105 | Rep 2 | LCE1C | 3 | | 126.17 | 993.45 | 126.17 | 993.45 | 4.61E+05 | 11666 | 8.0295 | 1 | 5.57E-16 | 1.11E-15 | 1.96E-14 | TRUE |  |  |  |  |  |  |  |
| HGLibA_26106 | Rep 3 | LCE1C | 2 | | 1.1406 | 12.189 | 1.1406 | 12.189 | 77.394 | 2.5378 | 6.9353 | 1 | 2.66E-12 | 5.31E-12 | 6.72E-11 | TRUE |  |  |  |  |  |  |  |
| HGLibA_26107 | Rep 3 | LCE1C | 2 | | 41.374 | 219.4 | 41.374 | 219.4 | 18307 | 894.44 | 5.9527 | 1 | 1.44E-09 | 2.88E-09 | 2.94E-08 | TRUE |  |  |  |  |  |  |  |
| HGLibA_26389 | Rep 2 | LGALSL | 3 | | 280.74 | 3031.8 | 280.74 | 3031.8 | 4.86E+06 | 50284 | 12.268 | 1 | 7.50E-35 | 1.50E-34 | 6.27E-33 | TRUE |  |  |  |  |  |  |  |
| HGLibA_26391 | Rep 2 | LGALSL | 3 | | 144.31 | 700.43 | 144.31 | 700.43 | 1.76E+05 | 14907 | 4.5549 | 1 | 2.97E-06 | 5.95E-06 | 4.26E-05 | TRUE |  |  |  |  |  |  |  |
| HGLibA_26390 | Rep 2 | LGALSL | 3 | | 637.17 | 2778.6 | 637.17 | 2778.6 | 2.58E+06 | 2.25E+05 | 4.5111 | 1 | 3.54E-06 | 7.09E-06 | 5.03E-05 | TRUE |  |  |  |  |  |  |  |
| HGLibA_26391 | Rep 3 | LGALSL | 2 | | 41.063 | 207.21 | 41.063 | 207.21 | 15833 | 882.7 | 5.5923 | 1 | 1.22E-08 | 2.44E-08 | 2.30E-07 | TRUE |  |  |  |  |  |  |  |
| HGLibA_26390 | Rep 3 | LGALSL | 2 | | 169.96 | 719.15 | 169.96 | 719.15 | 1.69E+05 | 10812 | 5.2818 | 1 | 6.74E-08 | 1.35E-07 | 1.18E-06 | TRUE |  |  |  |  |  |  |  |
| HGLibA_29925 | Rep 1 | MRPL20 | 2 | | 80.559 | 612.82 | 80.559 | 612.82 | 1.73E+05 | 10086 | 5.3 | 1 | 7.34E-08 | 1.47E-07 | 9.04E-07 | TRUE |  |  |  |  |  |  |  |
| HGLibA_29924 | Rep 1 | MRPL20 | 2 | | 27.117 | 434.08 | 27.117 | 434.08 | 1.13E+05 | 1045.3 | 12.587 | 1 | 1.55E-36 | 3.11E-36 | 5.51E-35 | TRUE |  |  |  |  |  |  |  |
| HGLibA_29924 | Rep 2 | MRPL20 | 3 | | 280.74 | 16797 | 280.74 | 16797 | 2.17E+08 | 50284 | 73.656 | 1 | 0 | 0 | 0 | TRUE |  |  |  |  |  |  |  |
| HGLibA_29923 | Rep 2 | MRPL20 | 3 | | 200.3 | 1576.9 | 200.3 | 1576.9 | 1.16E+06 | 27128 | 8.3581 | 1 | 3.59E-17 | 7.17E-17 | 1.36E-15 | TRUE |  |  |  |  |  |  |  |
| HGLibA_29925 | Rep 2 | MRPL20 | 3 | | 1040.9 | 4292.5 | 1040.9 | 4292.5 | 5.90E+06 | 5.54E+05 | 4.37 | 1 | 6.76E-06 | 1.35E-05 | 9.24E-05 | TRUE |  |  |  |  |  |  |  |
| HGLibA_37705 | Rep 1 | POR | 2 | | 39.795 | 408.55 | 39.795 | 408.55 | 86648 | 2317.2 | 7.6604 | 1 | 1.16E-14 | 2.33E-14 | 2.19E-13 | TRUE |  |  |  |  |  |  |  |
| HGLibA_37706 | Rep 1 | POR | 2 | | 51.505 | 1174.6 | 51.505 | 1174.6 | 9.00E+05 | 3964.7 | 17.836 | 1 | 2.33E-71 | 4.66E-71 | 1.31E-69 | TRUE |  |  |  |  |  |  |  |
| HGLibA_37704 | Rep 2 | POR | 3 | | 133.27 | 1505 | 133.27 | 1505 | 1.22E+06 | 12891 | 12.081 | 1 | 7.56E-34 | 1.51E-33 | 6.14E-32 | TRUE |  |  |  |  |  |  |  |
| HGLibA_37705 | Rep 2 | POR | 3 | | 463.69 | 2808.1 | 463.69 | 2808.1 | 3.24E+06 | 1.26E+05 | 6.6065 | 1 | 2.18E-11 | 4.35E-11 | 5.42E-10 | TRUE |  |  |  |  |  |  |  |
| HGLibA_37706 | Rep 2 | POR | 3 | | 371.42 | 2062.7 | 371.42 | 2062.7 | 1.66E+06 | 83909 | 5.8387 | 1 | 2.92E-09 | 5.84E-09 | 5.98E-08 | TRUE |  |  |  |  |  |  |  |
| HGLibA_38675 | Rep 1 | PROKR2 | 2 | | 62.158 | 638.36 | 62.158 | 638.36 | 2.12E+05 | 5868.2 | 7.5218 | 1 | 3.41E-14 | 6.83E-14 | 6.27E-13 | TRUE |  |  |  |  |  |  |  |
| HGLibA_38673 | Rep 1 | PROKR2 | 2 | | 25.268 | 306.41 | 25.268 | 306.41 | 51614 | 903.24 | 9.3546 | 1 | 5.25E-21 | 1.05E-20 | 1.28E-19 | TRUE |  |  |  |  |  |  |  |
| HGLibA_38673 | Rep 3 | PROKR2 | 3 | | 3.0071 | 60.945 | 3.0071 | 60.945 | 2348.5 | 10.9 | 17.548 | 1 | 3.73E-69 | 7.46E-69 | 3.45E-67 | TRUE |  |  |  |  |  |  |  |
| HGLibA_38675 | Rep 3 | PROKR2 | 3 | | 157.82 | 1401.7 | 157.82 | 1401.7 | 9.65E+05 | 9480.8 | 12.775 | 1 | 1.19E-37 | 2.38E-37 | 7.69E-36 | TRUE |  |  |  |  |  |  |  |
| HGLibA_38674 | Rep 3 | PROKR2 | 3 | | 63.565 | 463.18 | 63.565 | 463.18 | 96677 | 1900.5 | 9.1667 | 1 | 2.63E-20 | 5.26E-20 | 1.03E-18 | TRUE |  |  |  |  |  |  |  |
| HGLibA_39077 | Rep 1 | PSMA3 | 2 | | 23.243 | 178.74 | 23.243 | 178.74 | 14751 | 760.04 | 5.6403 | 1 | 1.06E-08 | 2.12E-08 | 1.40E-07 | TRUE |  |  |  |  |  |  |  |
| HGLibA_39075 | Rep 1 | PSMA3 | 2 | | 15.848 | 178.74 | 15.848 | 178.74 | 17137 | 345.67 | 8.7613 | 1 | 1.20E-18 | 2.40E-18 | 2.68E-17 | TRUE |  |  |  |  |  |  |  |
| HGLibA_39076 | Rep 2 | PSMA3 | 3 | | 36.275 | 944.61 | 36.275 | 944.61 | 5.99E+05 | 1210.3 | 26.109 | 1 | 1.69E-150 | 3.37E-150 | 6.60E-148 | TRUE |  |  |  |  |  |  |  |
| HGLibA_39077 | Rep 2 | PSMA3 | 3 | | 125.38 | 818.67 | 125.38 | 818.67 | 2.86E+05 | 11534 | 6.4554 | 1 | 6.14E-11 | 1.23E-10 | 1.47E-09 | TRUE |  |  |  |  |  |  |  |
| HGLibA_39075 | Rep 2 | PSMA3 | 3 | | 138 | 667.01 | 138 | 667.01 | 1.60E+05 | 13739 | 4.5133 | 1 | 3.63E-06 | 7.25E-06 | 5.14E-05 | TRUE |  |  |  |  |  |  |  |
| HGLibA_42891 | Rep 2 | SCEL | 3 | | 130.12 | 1565.4 | 130.12 | 1565.4 | 1.34E+06 | 12340 | 12.92 | 1 | 1.97E-38 | 3.94E-38 | 1.82E-36 | TRUE |  |  |  |  |  |  |  |
| HGLibA_42889 | Rep 2 | SCEL | 3 | | 253.92 | 1548.7 | 253.92 | 1548.7 | 9.88E+05 | 41852 | 6.3288 | 1 | 1.38E-10 | 2.77E-10 | 3.21E-09 | TRUE |  |  |  |  |  |  |  |
| HGLibA_42890 | Rep 2 | SCEL | 3 | | 2026.7 | 9854.8 | 2026.7 | 9854.8 | 3.50E+07 | 1.88E+06 | 5.7144 | 1 | 5.92E-09 | 1.18E-08 | 1.17E-07 | TRUE |  |  |  |  |  |  |  |
| HGLibA_42889 | Rep 3 | SCEL | 2 | | 70.305 | 377.86 | 70.305 | 377.86 | 54750 | 2269.5 | 6.4559 | 1 | 5.79E-11 | 1.16E-10 | 1.33E-09 | TRUE |  |  |  |  |  |  |  |
| HGLibA_42890 | Rep 3 | SCEL | 2 | | 471.19 | 1913.7 | 471.19 | 1913.7 | 1.16E+06 | 66243 | 5.6045 | 1 | 1.08E-08 | 2.16E-08 | 2.04E-07 | TRUE |  |  |  |  |  |  |  |
| HGLibA_51962 | Rep 2 | TSPO2 | 3 | | 55.201 | 1255.6 | 55.201 | 1255.6 | 1.03E+06 | 2590.3 | 23.586 | 1 | 3.08E-123 | 6.17E-123 | 9.71E-121 | TRUE |  |  |  |  |  |  |  |
| HGLibA_51961 | Rep 2 | TSPO2 | 3 | | 264.96 | 3090.9 | 264.96 | 3090.9 | 5.19E+06 | 45239 | 13.286 | 1 | 1.56E-40 | 3.11E-40 | 1.53E-38 | TRUE |  |  |  |  |  |  |  |
| HGLibA_51960 | Rep 2 | TSPO2 | 3 | | 415.58 | 2914.8 | 415.58 | 2914.8 | 3.76E+06 | 1.03E+05 | 7.7851 | 1 | 3.86E-15 | 7.72E-15 | 1.28E-13 | TRUE |  |  |  |  |  |  |  |
| HGLibA_51961 | Rep 3 | TSPO2 | 2 | | 66.053 | 475.37 | 66.053 | 475.37 | 1.01E+05 | 2033.4 | 9.0772 | 1 | 6.00E-20 | 1.20E-19 | 2.31E-18 | TRUE |  |  |  |  |  |  |  |
| HGLibA_51962 | Rep 3 | TSPO2 | 2 | | 24.368 | 158.46 | 24.368 | 158.46 | 10698 | 355.75 | 7.1092 | 1 | 6.47E-13 | 1.29E-12 | 1.72E-11 | TRUE |  |  |  |  |  |  |  |
| HGLibA_52157 | Rep 1 | TTC8 | 2 | | 44.373 | 383.01 | 44.373 | 383.01 | 71204 | 2906.6 | 6.2813 | 1 | 2.11E-10 | 4.22E-10 | 3.16E-09 | TRUE |  |  |  |  |  |  |  |
| HGLibA_52156 | Rep 1 | TTC8 | 2 | | 35.657 | 306.41 | 35.657 | 306.41 | 45484 | 1844.4 | 6.3044 | 1 | 1.82E-10 | 3.63E-10 | 2.72E-09 | TRUE |  |  |  |  |  |  |  |
| HGLibA_52155 | Rep 2 | TTC8 | 3 | | 334.36 | 3287.5 | 334.36 | 3287.5 | 5.52E+06 | 69228 | 11.224 | 1 | 1.73E-29 | 3.46E-29 | 1.20E-27 | TRUE |  |  |  |  |  |  |  |
| HGLibA_52157 | Rep 2 | TTC8 | 3 | | 282.31 | 2219.5 | 282.31 | 2219.5 | 2.30E+06 | 50802 | 8.5948 | 1 | 4.66E-18 | 9.32E-18 | 1.87E-16 | TRUE |  |  |  |  |  |  |  |
| HGLibA_52156 | Rep 2 | TTC8 | 3 | | 496.02 | 2466.3 | 496.02 | 2466.3 | 2.22E+06 | 1.42E+05 | 5.2199 | 1 | 9.88E-08 | 1.98E-07 | 1.71E-06 | TRUE |  |  |  |  |  |  |  |
| HGLibA_00272 | Rep 1 | ABHD2 | 2 | | 83.024 | 561.75 | 83.024 | 561.75 | 1.37E+05 | 10741 | 4.6191 | 1 | 2.44E-06 | 4.89E-06 | 2.62E-05 | TRUE |  |  |  |  |  |  |  |
| HGLibA_00273 | Rep 1 | ABHD2 | 2 | | 26.589 | 331.95 | 26.589 | 331.95 | 61150 | 1003.6 | 9.6388 | 1 | 3.43E-22 | 6.86E-22 | 8.59E-21 | TRUE |  |  |  |  |  |  |  |
| HGLibA_00273 | Rep 3 | ABHD2 | 2 | | 60.558 | 341.29 | 60.558 | 341.29 | 45932 | 1745.1 | 6.7202 | 1 | 9.80E-12 | 1.96E-11 | 2.38E-10 | TRUE |  |  |  |  |  |  |  |
| HGLibA_00271 | Rep 3 | ABHD2 | 2 | | 19.598 | 121.89 | 19.598 | 121.89 | 6184.3 | 244.16 | 6.5464 | 1 | 3.29E-11 | 6.59E-11 | 7.70E-10 | TRUE |  |  |  |  |  |  |  |
| HGLibA_00325 | Rep 1 | ABRA | 2 | | 25.62 | 459.62 | 25.62 | 459.62 | 1.30E+05 | 929.47 | 14.235 | 1 | 3.46E-46 | 6.91E-46 | 1.45E-44 | TRUE |  |  |  |  |  |  |  |
| HGLibA_00326 | Rep 1 | ABRA | 2 | | 40.059 | 893.7 | 40.059 | 893.7 | 5.18E+05 | 2349.3 | 17.612 | 1 | 1.26E-69 | 2.52E-69 | 6.86E-68 | TRUE |  |  |  |  |  |  |  |
| HGLibA_00327 | Rep 3 | ABRA | 2 | | 54.232 | 889.8 | 54.232 | 889.8 | 4.76E+05 | 1437.5 | 22.038 | 1 | 6.73E-108 | 1.35E-107 | 7.87E-106 | TRUE |  |  |  |  |  |  |  |
| HGLibA_00326 | Rep 3 | ABRA | 2 | | 58.795 | 255.97 | 58.795 | 255.97 | 21844 | 1656.8 | 4.8441 | 1 | 6.87E-07 | 1.37E-06 | 1.08E-05 | TRUE |  |  |  |  |  |  |  |
| HGLibA_00750 | Rep 2 | ADAM2 | 2 | | 129.33 | 1220.9 | 129.33 | 1220.9 | 7.50E+05 | 12204 | 9.8813 | 1 | 2.85E-23 | 5.71E-23 | 1.53E-21 | TRUE |  |  |  |  |  |  |  |
| HGLibA_00748 | Rep 2 | ADAM2 | 2 | | 369.84 | 2429 | 369.84 | 2429 | 2.53E+06 | 83258 | 7.1364 | 1 | 5.32E-13 | 1.06E-12 | 1.51E-11 | TRUE |  |  |  |  |  |  |  |
| HGLibA_00749 | Rep 3 | ADAM2 | 2 | | 23.228 | 499.75 | 23.228 | 499.75 | 1.61E+05 | 327.41 | 26.335 | 1 | 4.22E-153 | 8.45E-153 | 5.91E-151 | TRUE |  |  |  |  |  |  |  |
| HGLibA_00750 | Rep 3 | ADAM2 | 2 | | 33.286 | 195.02 | 33.286 | 195.02 | 15330 | 611.71 | 6.5394 | 1 | 3.39E-11 | 6.78E-11 | 7.92E-10 | TRUE |  |  |  |  |  |  |  |
| HGLibA_00786 | Rep 1 | ADAM9 | 2 | | 28.702 | 229.81 | 28.702 | 229.81 | 24826 | 1175.8 | 5.8648 | 1 | 2.81E-09 | 5.63E-09 | 3.88E-08 | TRUE |  |  |  |  |  |  |  |
| HGLibA_00784 | Rep 1 | ADAM9 | 2 | | 13.382 | 587.29 | 13.382 | 587.29 | 2.54E+05 | 244.68 | 36.689 | 1 | 6.76E-295 | 1.35E-294 | 7.65E-293 | TRUE |  |  |  |  |  |  |  |
| HGLibA_00784 | Rep 3 | ADAM9 | 2 | | 29.864 | 560.69 | 29.864 | 560.69 | 1.96E+05 | 506.39 | 23.589 | 1 | 2.75E-123 | 5.50E-123 | 3.54E-121 | TRUE |  |  |  |  |  |  |  |
| HGLibA_00785 | Rep 3 | ADAM9 | 2 | | 340.84 | 1267.7 | 340.84 | 1267.7 | 4.73E+05 | 37224 | 4.8037 | 1 | 8.10E-07 | 1.62E-06 | 1.26E-05 | TRUE |  |  |  |  |  |  |  |
| HGLibA_01022 | Rep 2 | ADPGK | 2 | | 539.39 | 2980.4 | 539.39 | 2980.4 | 3.46E+06 | 1.66E+05 | 5.9893 | 1 | 1.16E-09 | 2.32E-09 | 2.47E-08 | TRUE |  |  |  |  |  |  |  |
| HGLibA_01023 | Rep 2 | ADPGK | 2 | | 444.76 | 2019 | 444.76 | 2019 | 1.40E+06 | 1.17E+05 | 4.6086 | 1 | 2.24E-06 | 4.49E-06 | 3.27E-05 | TRUE |  |  |  |  |  |  |  |
| HGLibA_01023 | Rep 3 | ADPGK | 2 | | 106.39 | 1328.6 | 106.39 | 1328.6 | 9.80E+05 | 4715.7 | 17.798 | 1 | 3.89E-71 | 7.79E-71 | 3.83E-69 | TRUE |  |  |  |  |  |  |  |
| HGLibA_01021 | Rep 3 | ADPGK | 2 | | 90.111 | 438.8 | 90.111 | 438.8 | 69394 | 3516.2 | 5.8804 | 1 | 2.19E-09 | 4.37E-09 | 4.40E-08 | TRUE |  |  |  |  |  |  |  |
| HGLibA_01233 | Rep 2 | AGPAT6 | 2 | | 115.13 | 1985.6 | 115.13 | 1985.6 | 2.40E+06 | 9871.9 | 18.826 | 1 | 2.64E-79 | 5.29E-79 | 5.32E-77 | TRUE |  |  |  |  |  |  |  |
| HGLibA_01231 | Rep 2 | AGPAT6 | 2 | | 695.53 | 3479 | 695.53 | 3479 | 4.44E+06 | 2.65E+05 | 5.4116 | 1 | 3.42E-08 | 6.85E-08 | 6.26E-07 | TRUE |  |  |  |  |  |  |  |
| HGLibA_01233 | Rep 3 | AGPAT6 | 2 | | 15.554 | 414.43 | 15.554 | 414.43 | 1.16E+05 | 164.17 | 31.131 | 1 | 5.21E-213 | 1.04E-212 | 7.86E-211 | TRUE |  |  |  |  |  |  |  |
| HGLibA_01232 | Rep 3 | AGPAT6 | 2 | | 77.875 | 450.99 | 77.875 | 450.99 | 81462 | 2717.8 | 7.1571 | 1 | 4.42E-13 | 8.84E-13 | 1.19E-11 | TRUE |  |  |  |  |  |  |  |
| HGLibA_01417 | Rep 2 | AKAP14 | 2 | | 611.94 | 4189.7 | 611.94 | 4189.7 | 7.68E+06 | 2.09E+05 | 7.821 | 1 | 2.88E-15 | 5.76E-15 | 9.68E-14 | TRUE |  |  |  |  |  |  |  |
| HGLibA_01418 | Rep 2 | AKAP14 | 2 | | 307.55 | 1799.3 | 307.55 | 1799.3 | 1.30E+06 | 59413 | 6.1199 | 1 | 5.22E-10 | 1.04E-09 | 1.15E-08 | TRUE |  |  |  |  |  |  |  |
| HGLibA_01417 | Rep 3 | AKAP14 | 2 | | 152.85 | 926.36 | 152.85 | 926.36 | 3.52E+05 | 8957.1 | 8.1731 | 1 | 1.59E-16 | 3.18E-16 | 5.23E-15 | TRUE |  |  |  |  |  |  |  |
| HGLibA_01419 | Rep 3 | AKAP14 | 2 | | 46.248 | 292.54 | 46.248 | 292.54 | 35954 | 1087 | 7.47 | 1 | 4.36E-14 | 8.72E-14 | 1.25E-12 | TRUE |  |  |  |  |  |  |  |
| HGLibA_02002 | Rep 2 | ANKRD13B | 2 | | 413.22 | 3162.9 | 413.22 | 3162.9 | 4.61E+06 | 1.02E+05 | 8.61 | 1 | 4.05E-18 | 8.10E-18 | 1.63E-16 | TRUE |  |  |  |  |  |  |  |
| HGLibA_02004 | Rep 2 | ANKRD13B | 2 | | 269.69 | 1231.2 | 269.69 | 1231.2 | 5.23E+05 | 46727 | 4.4481 | 1 | 4.85E-06 | 9.69E-06 | 6.74E-05 | TRUE |  |  |  |  |  |  |  |
| HGLibA_02002 | Rep 3 | ANKRD13B | 2 | | 19.391 | 195.02 | 19.391 | 195.02 | 19589 | 239.73 | 11.343 | 1 | 4.46E-30 | 8.93E-30 | 2.43E-28 | TRUE |  |  |  |  |  |  |  |
| HGLibA_02004 | Rep 3 | ANKRD13B | 2 | | 42.411 | 304.72 | 42.411 | 304.72 | 41564 | 934.04 | 8.583 | 1 | 5.04E-18 | 1.01E-17 | 1.78E-16 | TRUE |  |  |  |  |  |  |  |
| HGLibA_02780 | Rep 1 | ARHGAP44 | 2 | | 132.42 | 1736.3 | 132.42 | 1736.3 | 1.70E+06 | 28522 | 9.4971 | 1 | 1.38E-21 | 2.76E-21 | 3.40E-20 | TRUE |  |  |  |  |  |  |  |
| HGLibA_02779 | Rep 1 | ARHGAP44 | 2 | | 22.715 | 280.88 | 22.715 | 280.88 | 43643 | 724.81 | 9.5892 | 1 | 5.55E-22 | 1.11E-21 | 1.38E-20 | TRUE |  |  |  |  |  |  |  |
| HGLibA_02779 | Rep 2 | ARHGAP44 | 2 | | 335.94 | 2154 | 335.94 | 2154 | 1.96E+06 | 69826 | 6.8801 | 1 | 3.33E-12 | 6.66E-12 | 8.89E-11 | TRUE |  |  |  |  |  |  |  |
| HGLibA_02780 | Rep 2 | ARHGAP44 | 2 | | 1115.8 | 4706.4 | 1115.8 | 4706.4 | 7.21E+06 | 6.29E+05 | 4.5279 | 1 | 3.24E-06 | 6.47E-06 | 4.61E-05 | TRUE |  |  |  |  |  |  |  |
| HGLibA_02841 | Rep 2 | ARHGEF26 | 2 | | 227.11 | 2742.6 | 227.11 | 2742.6 | 4.13E+06 | 34129 | 13.616 | 1 | 1.80E-42 | 3.60E-42 | 1.85E-40 | TRUE |  |  |  |  |  |  |  |
| HGLibA_02839 | Rep 2 | ARHGEF26 | 2 | | 473.15 | 2400.7 | 473.15 | 2400.7 | 2.13E+06 | 1.31E+05 | 5.3323 | 1 | 5.36E-08 | 1.07E-07 | 9.59E-07 | TRUE |  |  |  |  |  |  |  |
| HGLibA_02840 | Rep 3 | ARHGEF26 | 2 | | 62.217 | 450.99 | 62.217 | 450.99 | 91430 | 1830.1 | 9.0878 | 1 | 5.45E-20 | 1.09E-19 | 2.10E-18 | TRUE |  |  |  |  |  |  |  |
| HGLibA_02841 | Rep 3 | ARHGEF26 | 2 | | 25.613 | 134.08 | 25.613 | 134.08 | 6782.1 | 387.82 | 5.5078 | 1 | 2.01E-08 | 4.02E-08 | 3.72E-07 | TRUE |  |  |  |  |  |  |  |
| HGLibA_03082 | Rep 1 | ARPC3 | 2 | | 104.95 | 791.56 | 104.95 | 791.56 | 2.87E+05 | 17533 | 5.1854 | 1 | 1.37E-07 | 2.74E-07 | 1.64E-06 | TRUE |  |  |  |  |  |  |  |
| HGLibA_03083 | Rep 1 | ARPC3 | 2 | | 122.29 | 1659.7 | 122.29 | 1659.7 | 1.57E+06 | 24147 | 9.8938 | 1 | 2.82E-23 | 5.65E-23 | 7.36E-22 | TRUE |  |  |  |  |  |  |  |
| HGLibA_03082 | Rep 3 | ARPC3 | 2 | | 234.04 | 2120.9 | 234.04 | 2120.9 | 2.23E+06 | 19079 | 13.66 | 1 | 9.20E-43 | 1.84E-42 | 6.35E-41 | TRUE |  |  |  |  |  |  |  |
| HGLibA_03083 | Rep 3 | ARPC3 | 2 | | 260.27 | 1791.8 | 260.27 | 1791.8 | 1.41E+06 | 23044 | 10.089 | 1 | 3.23E-24 | 6.47E-24 | 1.48E-22 | TRUE |  |  |  |  |  |  |  |
| HGLibA_03297 | Rep 2 | ASH2L | 2 | | 1363.5 | 7680.3 | 1363.5 | 7680.3 | 2.33E+07 | 9.08E+05 | 6.63 | 1 | 1.82E-11 | 3.63E-11 | 4.56E-10 | TRUE |  |  |  |  |  |  |  |
| HGLibA_03296 | Rep 2 | ASH2L | 2 | | 478.67 | 2763.2 | 478.67 | 2763.2 | 3.05E+06 | 1.33E+05 | 6.2529 | 1 | 2.23E-10 | 4.45E-10 | 5.07E-09 | TRUE |  |  |  |  |  |  |  |
| HGLibA_03295 | Rep 3 | ASH2L | 2 | | 18.872 | 182.83 | 18.872 | 182.83 | 16977 | 228.8 | 10.84 | 1 | 1.25E-27 | 2.50E-27 | 6.28E-26 | TRUE |  |  |  |  |  |  |  |
| HGLibA_03297 | Rep 3 | ASH2L | 2 | | 258.2 | 1170.1 | 258.2 | 1170.1 | 4.70E+05 | 22718 | 6.0503 | 1 | 7.56E-10 | 1.51E-09 | 1.59E-08 | TRUE |  |  |  |  |  |  |  |
| HGLibA_03965 | Rep 2 | B3GALT6 | 2 | | 140.37 | 1407.3 | 140.37 | 1407.3 | 1.02E+06 | 14172 | 10.642 | 1 | 1.07E-26 | 2.15E-26 | 6.72E-25 | TRUE |  |  |  |  |  |  |  |
| HGLibA_03966 | Rep 2 | B3GALT6 | 2 | | 1398.9 | 6934.9 | 1398.9 | 6934.9 | 1.75E+07 | 9.52E+05 | 5.6752 | 1 | 7.49E-09 | 1.50E-08 | 1.47E-07 | TRUE |  |  |  |  |  |  |  |
| HGLibA_03967 | Rep 3 | B3GALT6 | 2 | | 49.981 | 377.86 | 49.981 | 377.86 | 65438 | 1245.6 | 9.2903 | 1 | 8.35E-21 | 1.67E-20 | 3.33E-19 | TRUE |  |  |  |  |  |  |  |
| HGLibA_03966 | Rep 3 | B3GALT6 | 2 | | 344.37 | 1572.4 | 344.37 | 1572.4 | 8.53E+05 | 37912 | 6.3069 | 1 | 1.48E-10 | 2.96E-10 | 3.27E-09 | TRUE |  |  |  |  |  |  |  |
| HGLibA_03982 | Rep 1 | B3GNT1 | 2 | | 27.998 | 178.74 | 27.998 | 178.74 | 13485 | 1116.8 | 4.5107 | 1 | 4.04E-06 | 8.09E-06 | 4.24E-05 | TRUE |  |  |  |  |  |  |  |
| HGLibA_03981 | Rep 1 | B3GNT1 | 2 | | 4.6663 | 51.069 | 4.6663 | 51.069 | 1381.7 | 30.013 | 8.4701 | 1 | 1.53E-17 | 3.05E-17 | 3.25E-16 | TRUE |  |  |  |  |  |  |  |
| HGLibA_03982 | Rep 2 | B3GNT1 | 2 | | 175.07 | 2662.9 | 175.07 | 2662.9 | 4.18E+06 | 21212 | 17.082 | 1 | 1.14E-65 | 2.29E-65 | 1.87E-63 | TRUE |  |  |  |  |  |  |  |
| HGLibA_03980 | Rep 2 | B3GNT1 | 2 | | 570.14 | 6027.5 | 570.14 | 6027.5 | 1.91E+07 | 1.84E+05 | 12.728 | 1 | 2.28E-37 | 4.55E-37 | 2.04E-35 | TRUE |  |  |  |  |  |  |  |
| HGLibA_04045 | Rep 2 | B9D1 | 2 | | 98.573 | 917.63 | 98.573 | 917.63 | 4.21E+05 | 7437.9 | 9.497 | 1 | 1.24E-21 | 2.47E-21 | 6.07E-20 | TRUE |  |  |  |  |  |  |  |
| HGLibA_04043 | Rep 2 | B9D1 | 2 | | 267.33 | 1271.1 | 267.33 | 1271.1 | 5.73E+05 | 45980 | 4.6809 | 1 | 1.60E-06 | 3.20E-06 | 2.38E-05 | TRUE |  |  |  |  |  |  |  |
| HGLibA_04045 | Rep 3 | B9D1 | 2 | | 23.953 | 195.02 | 23.953 | 195.02 | 18008 | 345.33 | 9.2058 | 1 | 1.88E-20 | 3.76E-20 | 7.38E-19 | TRUE |  |  |  |  |  |  |  |
| HGLibA_04043 | Rep 3 | B9D1 | 2 | | 100.48 | 560.69 | 100.48 | 560.69 | 1.23E+05 | 4262.4 | 7.0491 | 1 | 9.60E-13 | 1.92E-12 | 2.51E-11 | TRUE |  |  |  |  |  |  |  |
| HGLibA_04126 | Rep 2 | BAIAP2L1 | 2 | | 298.08 | 1517.8 | 298.08 | 1517.8 | 8.55E+05 | 56112 | 5.1491 | 1 | 1.46E-07 | 2.92E-07 | 2.48E-06 | TRUE |  |  |  |  |  |  |  |
| HGLibA_04124 | Rep 2 | BAIAP2L1 | 2 | | 869.81 | 4035.5 | 869.81 | 4035.5 | 5.68E+06 | 3.98E+05 | 5.0152 | 1 | 2.89E-07 | 5.78E-07 | 4.72E-06 | TRUE |  |  |  |  |  |  |  |
| HGLibA_04125 | Rep 3 | BAIAP2L1 | 2 | | 65.224 | 560.69 | 65.224 | 560.69 | 1.52E+05 | 1988.6 | 11.111 | 1 | 6.00E-29 | 1.20E-28 | 3.17E-27 | TRUE |  |  |  |  |  |  |  |
| HGLibA_04124 | Rep 3 | BAIAP2L1 | 2 | | 210.09 | 755.72 | 210.09 | 755.72 | 1.63E+05 | 15750 | 4.3477 | 1 | 7.22E-06 | 1.44E-05 | 9.93E-05 | TRUE |  |  |  |  |  |  |  |
| HGLibA_04299 | Rep 2 | BCL2A1 | 2 | | 146.68 | 1705.4 | 146.68 | 1705.4 | 1.58E+06 | 15356 | 12.579 | 1 | 1.56E-36 | 3.12E-36 | 1.37E-34 | TRUE |  |  |  |  |  |  |  |
| HGLibA_04300 | Rep 2 | BCL2A1 | 2 | | 443.18 | 2449.6 | 443.18 | 2449.6 | 2.34E+06 | 1.16E+05 | 5.8928 | 1 | 2.10E-09 | 4.20E-09 | 4.36E-08 | TRUE |  |  |  |  |  |  |  |
| HGLibA_04300 | Rep 3 | BCL2A1 | 2 | | 85.963 | 402.24 | 85.963 | 402.24 | 56770 | 3235.5 | 5.5602 | 1 | 1.44E-08 | 2.88E-08 | 2.70E-07 | TRUE |  |  |  |  |  |  |  |
| HGLibA_04298 | Rep 3 | BCL2A1 | 2 | | 89.592 | 365.67 | 89.592 | 365.67 | 42453 | 3480.6 | 4.6796 | 1 | 1.54E-06 | 3.07E-06 | 2.30E-05 | TRUE |  |  |  |  |  |  |  |
| HGLibA_04489 | Rep 1 | BHMT2 | 2 | | 46.574 | 893.7 | 46.574 | 893.7 | 5.00E+05 | 3214.8 | 14.941 | 1 | 1.13E-50 | 2.26E-50 | 5.03E-49 | TRUE |  |  |  |  |  |  |  |
| HGLibA_04487 | Rep 1 | BHMT2 | 2 | | 0.88042 | 25.534 | 0.88042 | 25.534 | 441.49 | 1.6459 | 19.217 | 1 | 1.77E-82 | 3.55E-82 | 1.07E-80 | TRUE |  |  |  |  |  |  |  |
| HGLibA_04489 | Rep 3 | BHMT2 | 2 | | 83.163 | 402.24 | 83.163 | 402.24 | 58049 | 3051.8 | 5.7758 | 1 | 4.10E-09 | 8.20E-09 | 8.06E-08 | TRUE |  |  |  |  |  |  |  |
| HGLibA_04488 | Rep 3 | BHMT2 | 2 | | 167.78 | 670.39 | 167.78 | 670.39 | 1.40E+05 | 10567 | 4.8895 | 1 | 5.33E-07 | 1.07E-06 | 8.46E-06 | TRUE |  |  |  |  |  |  |  |
| HGLibA_05064 | Rep 1 | C10orf25 | 2 | | 33.104 | 408.55 | 33.104 | 408.55 | 92295 | 1580.7 | 9.4432 | 1 | 2.27E-21 | 4.53E-21 | 5.57E-20 | TRUE |  |  |  |  |  |  |  |
| HGLibA_05065 | Rep 1 | C10orf25 | 2 | | 19.898 | 331.95 | 19.898 | 331.95 | 66561 | 551.64 | 13.286 | 1 | 1.74E-40 | 3.48E-40 | 6.66E-39 | TRUE |  |  |  |  |  |  |  |
| HGLibA_05065 | Rep 2 | C10orf25 | 2 | | 272.06 | 5085.5 | 272.06 | 5085.5 | 1.61E+07 | 47479 | 22.09 | 1 | 2.19E-108 | 4.37E-108 | 6.02E-106 | TRUE |  |  |  |  |  |  |  |
| HGLibA_05063 | Rep 2 | C10orf25 | 2 | | 330.42 | 1639.9 | 330.42 | 1639.9 | 9.81E+05 | 67742 | 5.0312 | 1 | 2.71E-07 | 5.43E-07 | 4.45E-06 | TRUE |  |  |  |  |  |  |  |
| HGLibA_06385 | Rep 1 | C5orf45 | 2 | | 65.416 | 638.36 | 65.416 | 638.36 | 2.08E+05 | 6528.5 | 7.0909 | 1 | 8.42E-13 | 1.68E-12 | 1.44E-11 | TRUE |  |  |  |  |  |  |  |
| HGLibA_06387 | Rep 1 | C5orf45 | 2 | | 50.712 | 766.03 | 50.712 | 766.03 | 3.45E+05 | 3838.6 | 11.545 | 1 | 4.91E-31 | 9.81E-31 | 1.56E-29 | TRUE |  |  |  |  |  |  |  |
| HGLibA_06385 | Rep 2 | C5orf45 | 2 | | 487.34 | 2934.1 | 487.34 | 2934.1 | 3.52E+06 | 1.38E+05 | 6.5878 | 1 | 2.47E-11 | 4.93E-11 | 6.11E-10 | TRUE |  |  |  |  |  |  |  |
| HGLibA_06387 | Rep 2 | C5orf45 | 2 | | 578.03 | 2552.4 | 578.03 | 2552.4 | 2.19E+06 | 1.89E+05 | 4.5471 | 1 | 2.99E-06 | 5.99E-06 | 4.29E-05 | TRUE |  |  |  |  |  |  |  |
| HGLibA_06618 | Rep 1 | C8orf4 | 2 | | 28.086 | 280.88 | 28.086 | 280.88 | 40556 | 1124.1 | 7.5397 | 1 | 2.95E-14 | 5.90E-14 | 5.42E-13 | TRUE |  |  |  |  |  |  |  |
| HGLibA_06617 | Rep 1 | C8orf4 | 2 | | 40.235 | 612.82 | 40.235 | 612.82 | 2.21E+05 | 2370.9 | 11.759 | 1 | 3.97E-32 | 7.94E-32 | 1.30E-30 | TRUE |  |  |  |  |  |  |  |
| HGLibA_06618 | Rep 3 | C8orf4 | 2 | | 113.23 | 2352.5 | 113.23 | 2352.5 | 3.53E+06 | 5265.5 | 30.859 | 1 | 2.26E-209 | 4.52E-209 | 3.39E-207 | TRUE |  |  |  |  |  |  |  |
| HGLibA_06617 | Rep 3 | C8orf4 | 2 | | 85.133 | 402.24 | 85.133 | 402.24 | 57146 | 3180.6 | 5.6227 | 1 | 1.01E-08 | 2.01E-08 | 1.91E-07 | TRUE |  |  |  |  |  |  |  |
| HGLibA_07215 | Rep 1 | CAPZB | 2 | | 54.322 | 587.29 | 54.322 | 587.29 | 1.82E+05 | 4430.3 | 8.0073 | 1 | 7.40E-16 | 1.48E-15 | 1.47E-14 | TRUE |  |  |  |  |  |  |  |
| HGLibA_07213 | Rep 1 | CAPZB | 2 | | 62.95 | 766.03 | 62.95 | 766.03 | 3.23E+05 | 6025.4 | 9.0575 | 1 | 8.43E-20 | 1.69E-19 | 1.96E-18 | TRUE |  |  |  |  |  |  |  |
| HGLibA_07214 | Rep 2 | CAPZB | 2 | | 106.46 | 796.82 | 106.46 | 796.82 | 2.90E+05 | 8558 | 7.4626 | 1 | 4.85E-14 | 9.70E-14 | 1.50E-12 | TRUE |  |  |  |  |  |  |  |
| HGLibA_07215 | Rep 2 | CAPZB | 2 | | 295.72 | 1544.8 | 295.72 | 1544.8 | 9.00E+05 | 55300 | 5.3116 | 1 | 6.07E-08 | 1.21E-07 | 1.08E-06 | TRUE |  |  |  |  |  |  |  |
| HGLibA_08466 | Rep 1 | CD84 | 2 | | 32.928 | 255.34 | 32.928 | 255.34 | 30222 | 1563.3 | 5.6252 | 1 | 1.16E-08 | 2.32E-08 | 1.53E-07 | TRUE |  |  |  |  |  |  |  |
| HGLibA_08465 | Rep 1 | CD84 | 2 | | 22.979 | 204.27 | 22.979 | 204.27 | 20493 | 742.31 | 6.6542 | 1 | 1.78E-11 | 3.56E-11 | 2.82E-10 | TRUE |  |  |  |  |  |  |  |
| HGLibA_08465 | Rep 2 | CD84 | 2 | | 233.42 | 1253.1 | 233.42 | 1253.1 | 6.02E+05 | 35882 | 5.3828 | 1 | 4.11E-08 | 8.23E-08 | 7.46E-07 | TRUE |  |  |  |  |  |  |  |
| HGLibA_08466 | Rep 2 | CD84 | 2 | | 339.88 | 1620.6 | 339.88 | 1620.6 | 9.33E+05 | 71333 | 4.7953 | 1 | 9.04E-07 | 1.81E-06 | 1.39E-05 | TRUE |  |  |  |  |  |  |  |
| HGLibA_09171 | Rep 2 | CEP85 | 2 | | 17.349 | 275.03 | 17.349 | 275.03 | 45059 | 321.02 | 14.382 | 1 | 4.03E-47 | 8.06E-47 | 4.68E-45 | TRUE |  |  |  |  |  |  |  |
| HGLibA_09169 | Rep 2 | CEP85 | 2 | | 319.38 | 1575.6 | 319.38 | 1575.6 | 9.02E+05 | 63659 | 4.9791 | 1 | 3.56E-07 | 7.12E-07 | 5.74E-06 | TRUE |  |  |  |  |  |  |  |
| HGLibA_09170 | Rep 3 | CEP85 | 2 | | 52.884 | 463.18 | 52.884 | 463.18 | 1.05E+05 | 1375.4 | 11.063 | 1 | 1.02E-28 | 2.04E-28 | 5.37E-27 | TRUE |  |  |  |  |  |  |  |
| HGLibA_09169 | Rep 3 | CEP85 | 2 | | 74.66 | 341.29 | 74.66 | 341.29 | 40216 | 2523.1 | 5.3082 | 1 | 5.94E-08 | 1.19E-07 | 1.06E-06 | TRUE |  |  |  |  |  |  |  |
| HGLibA_09255 | Rep 1 | CFD | 2 | | 19.986 | 178.74 | 19.986 | 178.74 | 15726 | 556.68 | 6.7286 | 1 | 1.07E-11 | 2.14E-11 | 1.72E-10 | TRUE |  |  |  |  |  |  |  |
| HGLibA_09253 | Rep 1 | CFD | 2 | | 71.05 | 995.84 | 71.05 | 995.84 | 5.71E+05 | 7757.9 | 10.5 | 1 | 5.49E-26 | 1.10E-25 | 1.54E-24 | TRUE |  |  |  |  |  |  |  |
| HGLibA_09255 | Rep 3 | CFD | 2 | | 14.414 | 109.7 | 14.414 | 109.7 | 5528.8 | 144.12 | 7.9372 | 1 | 1.17E-15 | 2.34E-15 | 3.65E-14 | TRUE |  |  |  |  |  |  |  |
| HGLibA_09253 | Rep 3 | CFD | 2 | | 181.15 | 719.15 | 181.15 | 719.15 | 1.61E+05 | 12108 | 4.8894 | 1 | 5.32E-07 | 1.06E-06 | 8.46E-06 | TRUE |  |  |  |  |  |  |  |
| HGLibA_09498 | Rep 1 | CHMP6 | 2 | | 80.735 | 536.22 | 80.735 | 536.22 | 1.24E+05 | 10132 | 4.5251 | 1 | 3.83E-06 | 7.65E-06 | 4.02E-05 | TRUE |  |  |  |  |  |  |  |
| HGLibA_09500 | Rep 1 | CHMP6 | 2 | | 11.357 | 102.14 | 11.357 | 102.14 | 5144 | 175.28 | 6.8568 | 1 | 4.38E-12 | 8.75E-12 | 7.18E-11 | TRUE |  |  |  |  |  |  |  |
| HGLibA_09499 | Rep 2 | CHMP6 | 2 | | 84.378 | 3800.3 | 84.378 | 3800.3 | 1.07E+07 | 5603.3 | 49.641 | 1 | 0 | 0 | 0 | TRUE |  |  |  |  |  |  |  |
| HGLibA_09500 | Rep 2 | CHMP6 | 2 | | 56.778 | 774.97 | 56.778 | 774.97 | 3.43E+05 | 2726.2 | 13.755 | 1 | 2.76E-43 | 5.52E-43 | 2.91E-41 | TRUE |  |  |  |  |  |  |  |
| HGLibA_09668 | Rep 1 | CHTOP | 2 | | 48.864 | 357.48 | 48.864 | 357.48 | 57691 | 3552.8 | 5.1777 | 1 | 1.42E-07 | 2.83E-07 | 1.69E-06 | TRUE |  |  |  |  |  |  |  |
| HGLibA_09667 | Rep 1 | CHTOP | 2 | | 28.35 | 791.56 | 28.35 | 791.56 | 4.26E+05 | 1146.1 | 22.544 | 1 | 9.65E-113 | 1.93E-112 | 6.93E-111 | TRUE |  |  |  |  |  |  |  |
| HGLibA_09667 | Rep 3 | CHTOP | 2 | | 38.471 | 341.29 | 38.471 | 341.29 | 57170 | 787.57 | 10.79 | 1 | 2.09E-27 | 4.17E-27 | 1.04E-25 | TRUE |  |  |  |  |  |  |  |
| HGLibA_09668 | Rep 3 | CHTOP | 2 | | 135.94 | 755.72 | 135.94 | 755.72 | 2.23E+05 | 7277.4 | 7.2652 | 1 | 1.97E-13 | 3.94E-13 | 5.41E-12 | TRUE |  |  |  |  |  |  |  |
| HGLibA_10153 | Rep 1 | CLTCL1 | 2 | | 81.791 | 561.75 | 81.791 | 561.75 | 1.38E+05 | 10411 | 4.704 | 1 | 1.62E-06 | 3.24E-06 | 1.76E-05 | TRUE |  |  |  |  |  |  |  |
| HGLibA_10152 | Rep 1 | CLTCL1 | 2 | | 56.523 | 561.75 | 56.523 | 561.75 | 1.62E+05 | 4812.9 | 7.2826 | 1 | 2.07E-13 | 4.13E-13 | 3.65E-12 | TRUE |  |  |  |  |  |  |  |
| HGLibA_10151 | Rep 3 | CLTCL1 | 2 | | 33.493 | 390.05 | 33.493 | 390.05 | 82534 | 618.37 | 14.338 | 1 | 6.90E-47 | 1.38E-46 | 5.02E-45 | TRUE |  |  |  |  |  |  |  |
| HGLibA_10153 | Rep 3 | CLTCL1 | 2 | | 150.67 | 1377.4 | 150.67 | 1377.4 | 9.43E+05 | 8732.2 | 13.127 | 1 | 1.21E-39 | 2.43E-39 | 8.07E-38 | TRUE |  |  |  |  |  |  |  |
| HGLibA_10197 | Rep 1 | CMKLR1 | 2 | | 62.246 | 485.15 | 62.246 | 485.15 | 1.09E+05 | 5885.6 | 5.5125 | 1 | 2.24E-08 | 4.47E-08 | 2.87E-07 | TRUE |  |  |  |  |  |  |  |
| HGLibA_10196 | Rep 1 | CMKLR1 | 2 | | 51.945 | 791.56 | 51.945 | 791.56 | 3.69E+05 | 4035.7 | 11.643 | 1 | 1.58E-31 | 3.16E-31 | 5.07E-30 | TRUE |  |  |  |  |  |  |  |
| HGLibA_10196 | Rep 2 | CMKLR1 | 2 | | 373.79 | 4495.6 | 373.79 | 4495.6 | 1.11E+07 | 84889 | 14.147 | 1 | 1.08E-45 | 2.17E-45 | 1.21E-43 | TRUE |  |  |  |  |  |  |  |
| HGLibA_10198 | Rep 2 | CMKLR1 | 2 | | 420.31 | 2046 | 420.31 | 2046 | 1.51E+06 | 1.05E+05 | 5.0119 | 1 | 2.99E-07 | 5.97E-07 | 4.87E-06 | TRUE |  |  |  |  |  |  |  |
| HGLibA_10398 | Rep 1 | CNTLN | 2 | | 49.128 | 357.48 | 49.128 | 357.48 | 57546 | 3592.9 | 5.1443 | 1 | 1.69E-07 | 3.38E-07 | 2.01E-06 | TRUE |  |  |  |  |  |  |  |
| HGLibA_10399 | Rep 1 | CNTLN | 2 | | 56.435 | 944.77 | 56.435 | 944.77 | 5.40E+05 | 4797.2 | 12.826 | 1 | 7.43E-38 | 1.49E-37 | 2.69E-36 | TRUE |  |  |  |  |  |  |  |
| HGLibA_10399 | Rep 2 | CNTLN | 2 | | 363.54 | 2531.8 | 363.54 | 2531.8 | 2.83E+06 | 80678 | 7.6338 | 1 | 1.27E-14 | 2.53E-14 | 4.06E-13 | TRUE |  |  |  |  |  |  |  |
| HGLibA_10398 | Rep 2 | CNTLN | 2 | | 307.55 | 1733.7 | 307.55 | 1733.7 | 1.19E+06 | 59413 | 5.851 | 1 | 2.72E-09 | 5.45E-09 | 5.60E-08 | TRUE |  |  |  |  |  |  |  |
| HGLibA_10483 | Rep 2 | COG5 | 2 | | 486.55 | 7136.7 | 486.55 | 7136.7 | 2.97E+07 | 1.38E+05 | 17.932 | 1 | 3.68E-72 | 7.36E-72 | 6.66E-70 | TRUE |  |  |  |  |  |  |  |
| HGLibA_10482 | Rep 2 | COG5 | 2 | | 238.94 | 1449.7 | 238.94 | 1449.7 | 8.64E+05 | 37448 | 6.2567 | 1 | 2.21E-10 | 4.41E-10 | 5.02E-09 | TRUE |  |  |  |  |  |  |  |
| HGLibA_10482 | Rep 3 | COG5 | 2 | | 37.226 | 195.02 | 37.226 | 195.02 | 14358 | 743.6 | 5.7867 | 1 | 3.93E-09 | 7.85E-09 | 7.74E-08 | TRUE |  |  |  |  |  |  |  |
| HGLibA_10483 | Rep 3 | COG5 | 2 | | 150.88 | 585.07 | 150.88 | 585.07 | 1.04E+05 | 8753.5 | 4.6408 | 1 | 1.83E-06 | 3.67E-06 | 2.72E-05 | TRUE |  |  |  |  |  |  |  |
| HGLibA_10586 | Rep 1 | COL5A1 | 2 | | 21.57 | 383.01 | 21.57 | 383.01 | 90085 | 651.47 | 14.161 | 1 | 9.97E-46 | 1.99E-45 | 4.14E-44 | TRUE |  |  |  |  |  |  |  |
| HGLibA_10588 | Rep 1 | COL5A1 | 2 | | 12.678 | 612.82 | 12.678 | 612.82 | 2.81E+05 | 219.17 | 40.538 | 1 | 0 | 0 | 0 | TRUE |  |  |  |  |  |  |  |
| HGLibA_10588 | Rep 2 | COL5A1 | 2 | | 210.55 | 2245.2 | 210.55 | 2245.2 | 2.65E+06 | 29718 | 11.803 | 1 | 2.13E-32 | 4.25E-32 | 1.65E-30 | TRUE |  |  |  |  |  |  |  |
| HGLibA_10586 | Rep 2 | COL5A1 | 2 | | 186.89 | 1282.6 | 186.89 | 1282.6 | 7.20E+05 | 23903 | 7.0873 | 1 | 7.71E-13 | 1.54E-12 | 2.17E-11 | TRUE |  |  |  |  |  |  |  |
| HGLibA_10872 | Rep 2 | COX7C | 2 | | 283.89 | 2596.1 | 283.89 | 2596.1 | 3.35E+06 | 51322 | 10.206 | 1 | 1.04E-24 | 2.07E-24 | 5.94E-23 | TRUE |  |  |  |  |  |  |  |
| HGLibA_10873 | Rep 2 | COX7C | 2 | | 339.09 | 1553.8 | 339.09 | 1553.8 | 8.35E+05 | 71031 | 4.5577 | 1 | 2.88E-06 | 5.76E-06 | 4.14E-05 | TRUE |  |  |  |  |  |  |  |
| HGLibA_10872 | Rep 3 | COX7C | 2 | | 36.915 | 414.43 | 36.915 | 414.43 | 92024 | 732.78 | 13.946 | 1 | 1.83E-44 | 3.65E-44 | 1.29E-42 | TRUE |  |  |  |  |  |  |  |
| HGLibA_10873 | Rep 3 | COX7C | 2 | | 79.741 | 329.1 | 79.741 | 329.1 | 34686 | 2833.8 | 4.6843 | 1 | 1.51E-06 | 3.01E-06 | 2.26E-05 | TRUE |  |  |  |  |  |  |  |
| HGLibA_10998 | Rep 2 | CPSF2 | 2 | | 676.6 | 3408.3 | 676.6 | 3408.3 | 4.28E+06 | 2.52E+05 | 5.4468 | 1 | 2.81E-08 | 5.63E-08 | 5.19E-07 | TRUE |  |  |  |  |  |  |  |
| HGLibA_10997 | Rep 2 | CPSF2 | 2 | | 600.9 | 2980.4 | 600.9 | 2980.4 | 3.24E+06 | 2.02E+05 | 5.2889 | 1 | 6.77E-08 | 1.35E-07 | 1.20E-06 | TRUE |  |  |  |  |  |  |  |
| HGLibA_10996 | Rep 3 | CPSF2 | 2 | | 95.088 | 499.75 | 95.088 | 499.75 | 94481 | 3866.5 | 6.5077 | 1 | 4.07E-11 | 8.14E-11 | 9.46E-10 | TRUE |  |  |  |  |  |  |  |
| HGLibA_10998 | Rep 3 | CPSF2 | 2 | | 212.68 | 816.66 | 212.68 | 816.66 | 2.02E+05 | 16097 | 4.7606 | 1 | 1.01E-06 | 2.03E-06 | 1.56E-05 | TRUE |  |  |  |  |  |  |  |
| HGLibA_11277 | Rep 1 | CRYGC | 2 | | 16.024 | 306.41 | 16.024 | 306.41 | 58731 | 353.58 | 15.443 | 1 | 5.23E-54 | 1.05E-53 | 2.41E-52 | TRUE |  |  |  |  |  |  |  |
| HGLibA_11276 | Rep 1 | CRYGC | 2 | | 15.848 | 485.15 | 15.848 | 485.15 | 1.63E+05 | 345.67 | 25.242 | 1 | 8.63E-141 | 1.73E-140 | 7.10E-139 | TRUE |  |  |  |  |  |  |  |
| HGLibA_11277 | Rep 2 | CRYGC | 2 | | 47.315 | 691.43 | 47.315 | 691.43 | 2.78E+05 | 1958.3 | 14.555 | 1 | 3.14E-48 | 6.29E-48 | 3.75E-46 | TRUE |  |  |  |  |  |  |  |
| HGLibA_11276 | Rep 2 | CRYGC | 2 | | 152.98 | 1245.3 | 152.98 | 1245.3 | 7.35E+05 | 16583 | 8.4828 | 1 | 1.25E-17 | 2.49E-17 | 4.85E-16 | TRUE |  |  |  |  |  |  |  |
| HGLibA_11443 | Rep 1 | CST1 | 2 | | 48.687 | 587.29 | 48.687 | 587.29 | 1.89E+05 | 3526.1 | 9.0702 | 1 | 7.48E-20 | 1.50E-19 | 1.75E-18 | TRUE |  |  |  |  |  |  |  |
| HGLibA_11444 | Rep 1 | CST1 | 2 | | 7.2195 | 229.81 | 7.2195 | 229.81 | 36814 | 70.56 | 26.499 | 1 | 6.24E-155 | 1.25E-154 | 5.33E-153 | TRUE |  |  |  |  |  |  |  |
| HGLibA_11445 | Rep 3 | CST1 | 2 | | 94.258 | 597.26 | 94.258 | 597.26 | 1.50E+05 | 3807.1 | 8.1521 | 1 | 1.91E-16 | 3.82E-16 | 6.26E-15 | TRUE |  |  |  |  |  |  |  |
| HGLibA_11444 | Rep 3 | CST1 | 2 | | 32.975 | 207.21 | 32.975 | 207.21 | 17975 | 601.77 | 7.1027 | 1 | 6.72E-13 | 1.34E-12 | 1.78E-11 | TRUE |  |  |  |  |  |  |  |
| HGLibA_11716 | Rep 1 | CTSL | 2 | | 49.744 | 383.01 | 49.744 | 383.01 | 67794 | 3687.4 | 5.4883 | 1 | 2.56E-08 | 5.11E-08 | 3.27E-07 | TRUE |  |  |  |  |  |  |  |
| HGLibA_11715 | Rep 1 | CTSL | 2 | | 18.841 | 383.01 | 18.841 | 383.01 | 93150 | 493.05 | 16.401 | 1 | 1.18E-60 | 2.36E-60 | 5.90E-59 | TRUE |  |  |  |  |  |  |  |
| HGLibA_11717 | Rep 2 | CTSL | 2 | | 67.818 | 613.04 | 67.818 | 613.04 | 1.86E+05 | 3765.2 | 8.8853 | 1 | 3.68E-19 | 7.36E-19 | 1.58E-17 | TRUE |  |  |  |  |  |  |  |
| HGLibA_11715 | Rep 2 | CTSL | 2 | | 108.04 | 837.94 | 108.04 | 837.94 | 3.26E+05 | 8790.5 | 7.785 | 1 | 3.98E-15 | 7.96E-15 | 1.32E-13 | TRUE |  |  |  |  |  |  |  |
| HGLibA_11812 | Rep 1 | CWC15 | 2 | | 57.668 | 459.62 | 57.668 | 459.62 | 99129 | 5018.4 | 5.674 | 1 | 8.80E-09 | 1.76E-08 | 1.17E-07 | TRUE |  |  |  |  |  |  |  |
| HGLibA_11813 | Rep 1 | CWC15 | 2 | | 33.632 | 510.69 | 33.632 | 510.69 | 1.54E+05 | 1633.5 | 11.803 | 1 | 2.35E-32 | 4.71E-32 | 7.78E-31 | TRUE |  |  |  |  |  |  |  |
| HGLibA_11811 | Rep 3 | CWC15 | 2 | | 12.029 | 170.65 | 12.029 | 170.65 | 16799 | 105.93 | 15.412 | 1 | 7.79E-54 | 1.56E-53 | 6.39E-52 | TRUE |  |  |  |  |  |  |  |
| HGLibA_11812 | Rep 3 | CWC15 | 2 | | 33.39 | 390.05 | 33.39 | 390.05 | 82620 | 615.03 | 14.381 | 1 | 3.71E-47 | 7.43E-47 | 2.71E-45 | TRUE |  |  |  |  |  |  |  |
| HGLibA_13003 | Rep 1 | DES | 2 | | 18.577 | 127.67 | 18.577 | 127.67 | 7138.9 | 478.95 | 4.9849 | 1 | 3.86E-07 | 7.73E-07 | 4.46E-06 | TRUE |  |  |  |  |  |  |  |
| HGLibA_13004 | Rep 1 | DES | 2 | | 27.029 | 638.36 | 27.029 | 638.36 | 2.68E+05 | 1038.3 | 18.972 | 1 | 1.83E-80 | 3.65E-80 | 1.09E-78 | TRUE |  |  |  |  |  |  |  |
| HGLibA_13005 | Rep 3 | DES | 2 | | 22.709 | 134.08 | 22.709 | 134.08 | 7275.5 | 314.87 | 6.2762 | 1 | 1.93E-10 | 3.86E-10 | 4.22E-09 | TRUE |  |  |  |  |  |  |  |
| HGLibA_13004 | Rep 3 | DES | 2 | | 82.126 | 353.48 | 82.126 | 353.48 | 41309 | 2985 | 4.9667 | 1 | 3.65E-07 | 7.30E-07 | 5.90E-06 | TRUE |  |  |  |  |  |  |  |
| HGLibA_13199 | Rep 1 | DHX40 | 2 | | 58.196 | 383.01 | 58.196 | 383.01 | 62909 | 5114.8 | 4.5418 | 1 | 3.52E-06 | 7.04E-06 | 3.71E-05 | TRUE |  |  |  |  |  |  |  |
| HGLibA_13200 | Rep 1 | DHX40 | 2 | | 79.414 | 612.82 | 79.414 | 612.82 | 1.74E+05 | 9788.5 | 5.3914 | 1 | 4.43E-08 | 8.86E-08 | 5.54E-07 | TRUE |  |  |  |  |  |  |  |
| HGLibA_13199 | Rep 2 | DHX40 | 2 | | 529.14 | 3646.1 | 529.14 | 3646.1 | 5.83E+06 | 1.60E+05 | 7.7835 | 1 | 3.89E-15 | 7.78E-15 | 1.29E-13 | TRUE |  |  |  |  |  |  |  |
| HGLibA_13200 | Rep 2 | DHX40 | 2 | | 608.78 | 2959.8 | 608.78 | 2959.8 | 3.15E+06 | 2.07E+05 | 5.1637 | 1 | 1.33E-07 | 2.66E-07 | 2.27E-06 | TRUE |  |  |  |  |  |  |  |
| HGLibA_14604 | Rep 1 | EFNB3 | 2 | | 44.373 | 357.48 | 44.373 | 357.48 | 60244 | 2906.6 | 5.8077 | 1 | 3.99E-09 | 7.97E-09 | 5.43E-08 | TRUE |  |  |  |  |  |  |  |
| HGLibA_14602 | Rep 1 | EFNB3 | 2 | | 23.948 | 357.48 | 23.948 | 357.48 | 74863 | 808.38 | 11.731 | 1 | 5.53E-32 | 1.11E-31 | 1.81E-30 | TRUE |  |  |  |  |  |  |  |
| HGLibA_14603 | Rep 3 | EFNB3 | 2 | | 14.932 | 524.13 | 14.932 | 524.13 | 1.95E+05 | 153.09 | 41.153 | 1 | 0 | 0 | 0 | TRUE |  |  |  |  |  |  |  |
| HGLibA_14604 | Rep 3 | EFNB3 | 2 | | 66.053 | 268.16 | 66.053 | 268.16 | 22734 | 2033.4 | 4.4819 | 1 | 3.98E-06 | 7.97E-06 | 5.69E-05 | TRUE |  |  |  |  |  |  |  |
| HGLibA_16029 | Rep 1 | FAM114A1 | 2 | | 20.162 | 153.21 | 20.162 | 153.21 | 10779 | 566.82 | 5.5882 | 1 | 1.43E-08 | 2.86E-08 | 1.87E-07 | TRUE |  |  |  |  |  |  |  |
| HGLibA_16030 | Rep 1 | FAM114A1 | 2 | | 18.929 | 306.41 | 18.929 | 306.41 | 56252 | 497.8 | 12.885 | 1 | 3.41E-38 | 6.82E-38 | 1.24E-36 | TRUE |  |  |  |  |  |  |  |
| HGLibA_16031 | Rep 3 | FAM114A1 | 2 | | 62.009 | 560.69 | 62.009 | 560.69 | 1.56E+05 | 1819.4 | 11.691 | 1 | 7.62E-32 | 1.52E-31 | 4.31E-30 | TRUE |  |  |  |  |  |  |  |
| HGLibA_16029 | Rep 3 | FAM114A1 | 2 | | 40.026 | 207.21 | 40.026 | 207.21 | 16090 | 844.08 | 5.7545 | 1 | 4.74E-09 | 9.49E-09 | 9.28E-08 | TRUE |  |  |  |  |  |  |  |
| HGLibA_16089 | Rep 1 | FAM127B | 2 | | 60.221 | 740.49 | 60.221 | 740.49 | 3.03E+05 | 5493.1 | 9.1786 | 1 | 2.76E-20 | 5.52E-20 | 6.50E-19 | TRUE |  |  |  |  |  |  |  |
| HGLibA_16091 | Rep 1 | FAM127B | 2 | | 24.3 | 383.01 | 24.3 | 383.01 | 87267 | 833.14 | 12.428 | 1 | 1.16E-35 | 2.31E-35 | 4.05E-34 | TRUE |  |  |  |  |  |  |  |
| HGLibA_16091 | Rep 3 | FAM127B | 2 | | 59.728 | 463.18 | 59.728 | 463.18 | 99464 | 1703.3 | 9.7757 | 1 | 7.73E-23 | 1.55E-22 | 3.33E-21 | TRUE |  |  |  |  |  |  |  |
| HGLibA_16089 | Rep 3 | FAM127B | 2 | | 65.535 | 329.1 | 65.535 | 329.1 | 39819 | 2005.4 | 5.8857 | 1 | 2.14E-09 | 4.27E-09 | 4.30E-08 | TRUE |  |  |  |  |  |  |  |
| HGLibA_16264 | Rep 2 | FAM171A1 | 2 | | 113.56 | 664.44 | 113.56 | 664.44 | 1.78E+05 | 9626.7 | 5.6147 | 1 | 1.12E-08 | 2.25E-08 | 2.17E-07 | TRUE |  |  |  |  |  |  |  |
| HGLibA_16263 | Rep 2 | FAM171A1 | 2 | | 303.6 | 1421.4 | 303.6 | 1421.4 | 7.09E+05 | 58027 | 4.6404 | 1 | 1.94E-06 | 3.88E-06 | 2.85E-05 | TRUE |  |  |  |  |  |  |  |
| HGLibA_16265 | Rep 3 | FAM171A1 | 2 | | 23.953 | 414.43 | 23.953 | 414.43 | 1.05E+05 | 345.33 | 21.012 | 1 | 2.80E-98 | 5.60E-98 | 3.16E-96 | TRUE |  |  |  |  |  |  |  |
| HGLibA_16263 | Rep 3 | FAM171A1 | 2 | | 72.275 | 329.1 | 72.275 | 329.1 | 37292 | 2382.8 | 5.2614 | 1 | 7.68E-08 | 1.54E-07 | 1.34E-06 | TRUE |  |  |  |  |  |  |  |
| HGLibA_16321 | Rep 2 | FAM181B | 2 | | 149.83 | 807.1 | 149.83 | 807.1 | 2.50E+05 | 15964 | 5.202 | 1 | 1.12E-07 | 2.23E-07 | 1.92E-06 | TRUE |  |  |  |  |  |  |  |
| HGLibA_16322 | Rep 2 | FAM181B | 2 | | 548.85 | 2578.1 | 548.85 | 2578.1 | 2.34E+06 | 1.71E+05 | 4.9005 | 1 | 5.27E-07 | 1.05E-06 | 8.33E-06 | TRUE |  |  |  |  |  |  |  |
| HGLibA_16321 | Rep 3 | FAM181B | 2 | | 23.124 | 292.54 | 23.124 | 292.54 | 47689 | 324.89 | 14.947 | 1 | 9.07E-51 | 1.81E-50 | 7.16E-49 | TRUE |  |  |  |  |  |  |  |
| HGLibA_16320 | Rep 3 | FAM181B | 2 | | 91.044 | 377.86 | 91.044 | 377.86 | 45923 | 3580.8 | 4.7931 | 1 | 8.78E-07 | 1.76E-06 | 1.36E-05 | TRUE |  |  |  |  |  |  |  |
| HGLibA_16621 | Rep 1 | FAM47E-STBD1 | 2 | | 77.83 | 638.36 | 77.83 | 638.36 | 1.94E+05 | 9384.8 | 5.7861 | 1 | 4.57E-09 | 9.13E-09 | 6.20E-08 | TRUE |  |  |  |  |  |  |  |
| HGLibA_16620 | Rep 1 | FAM47E-STBD1 | 2 | | 82.144 | 740.49 | 82.144 | 740.49 | 2.71E+05 | 10505 | 6.4234 | 1 | 8.45E-11 | 1.69E-10 | 1.29E-09 | TRUE |  |  |  |  |  |  |  |
| HGLibA_16621 | Rep 2 | FAM47E-STBD1 | 2 | | 488.92 | 5743.5 | 488.92 | 5743.5 | 1.80E+07 | 1.39E+05 | 14.106 | 1 | 1.92E-45 | 3.85E-45 | 2.14E-43 | TRUE |  |  |  |  |  |  |  |
| HGLibA_16620 | Rep 2 | FAM47E-STBD1 | 2 | | 507.06 | 3367.2 | 507.06 | 3367.2 | 4.89E+06 | 1.48E+05 | 7.4264 | 1 | 6.16E-14 | 1.23E-13 | 1.89E-12 | TRUE |  |  |  |  |  |  |  |
| HGLibA_16786 | Rep 1 | FAM89B | 2 | | 49.216 | 383.01 | 49.216 | 383.01 | 68117 | 3606.3 | 5.5584 | 1 | 1.71E-08 | 3.43E-08 | 2.23E-07 | TRUE |  |  |  |  |  |  |  |
| HGLibA_16785 | Rep 1 | FAM89B | 2 | | 26.061 | 434.08 | 26.061 | 434.08 | 1.14E+05 | 962.81 | 13.15 | 1 | 1.07E-39 | 2.14E-39 | 4.04E-38 | TRUE |  |  |  |  |  |  |  |
| HGLibA_16785 | Rep 2 | FAM89B | 2 | | 182.16 | 1913.6 | 182.16 | 1913.6 | 1.92E+06 | 22809 | 11.465 | 1 | 1.12E-30 | 2.24E-30 | 8.15E-29 | TRUE |  |  |  |  |  |  |  |
| HGLibA_16786 | Rep 2 | FAM89B | 2 | | 240.52 | 1200.4 | 240.52 | 1200.4 | 5.28E+05 | 37901 | 4.9304 | 1 | 4.60E-07 | 9.21E-07 | 7.33E-06 | TRUE |  |  |  |  |  |  |  |
| HGLibA_17427 | Rep 1 | FGFBP1 | 2 | | 144.3 | 1046.9 | 144.3 | 1046.9 | 4.93E+05 | 34146 | 4.8846 | 1 | 6.62E-07 | 1.32E-06 | 7.49E-06 | TRUE |  |  |  |  |  |  |  |
| HGLibA_17425 | Rep 1 | FGFBP1 | 2 | | 32.047 | 229.81 | 32.047 | 229.81 | 23615 | 1477.8 | 5.1443 | 1 | 1.68E-07 | 3.37E-07 | 2.00E-06 | TRUE |  |  |  |  |  |  |  |
| HGLibA_17425 | Rep 2 | FGFBP1 | 2 | | 386.4 | 2332.6 | 386.4 | 2332.6 | 2.23E+06 | 90206 | 6.48 | 1 | 5.09E-11 | 1.02E-10 | 1.23E-09 | TRUE |  |  |  |  |  |  |  |
| HGLibA_17427 | Rep 2 | FGFBP1 | 2 | | 1038.6 | 5400.4 | 1038.6 | 5400.4 | 1.10E+07 | 5.51E+05 | 5.8743 | 1 | 2.31E-09 | 4.62E-09 | 4.78E-08 | TRUE |  |  |  |  |  |  |  |
| HGLibA_17836 | Rep 1 | FOXD4 | 2 | | 13.823 | 127.67 | 13.823 | 127.67 | 8124.7 | 261.38 | 7.0419 | 1 | 1.18E-12 | 2.36E-12 | 2.00E-11 | TRUE |  |  |  |  |  |  |  |
| HGLibA_17837 | Rep 1 | FOXD4 | 2 | | 8.0119 | 153.21 | 8.0119 | 153.21 | 14671 | 86.825 | 15.582 | 1 | 5.99E-55 | 1.20E-54 | 2.84E-53 | TRUE |  |  |  |  |  |  |  |
| HGLibA_17837 | Rep 3 | FOXD4 | 2 | | 6.3254 | 146.27 | 6.3254 | 146.27 | 13974 | 36.116 | 23.286 | 1 | 3.58E-120 | 7.16E-120 | 4.57E-118 | TRUE |  |  |  |  |  |  |  |
| HGLibA_17836 | Rep 3 | FOXD4 | 2 | | 67.505 | 548.5 | 67.505 | 548.5 | 1.42E+05 | 2112.7 | 10.465 | 1 | 6.76E-26 | 1.35E-25 | 3.26E-24 | TRUE |  |  |  |  |  |  |  |
| HGLibA_18213 | Rep 1 | FUZ | 2 | | 26.501 | 383.01 | 26.501 | 383.01 | 85144 | 996.77 | 11.292 | 1 | 8.97E-30 | 1.79E-29 | 2.77E-28 | TRUE |  |  |  |  |  |  |  |
| HGLibA_18211 | Rep 1 | FUZ | 2 | | 28.614 | 663.89 | 28.614 | 663.89 | 2.89E+05 | 1168.4 | 18.586 | 1 | 2.64E-77 | 5.27E-77 | 1.54E-75 | TRUE |  |  |  |  |  |  |  |
| HGLibA_18212 | Rep 2 | FUZ | 2 | | 315.43 | 1471.5 | 315.43 | 1471.5 | 7.58E+05 | 62229 | 4.6345 | 1 | 1.99E-06 | 3.99E-06 | 2.93E-05 | TRUE |  |  |  |  |  |  |  |
| HGLibA_18211 | Rep 2 | FUZ | 2 | | 133.27 | 656.73 | 133.27 | 656.73 | 1.57E+05 | 12891 | 4.6104 | 1 | 2.28E-06 | 4.57E-06 | 3.33E-05 | TRUE |  |  |  |  |  |  |  |
| HGLibA_18433 | Rep 1 | GAGE10 | 2 | | 79.855 | 995.84 | 79.855 | 995.84 | 5.50E+05 | 9902.3 | 9.2049 | 1 | 2.17E-20 | 4.33E-20 | 5.13E-19 | TRUE |  |  |  |  |  |  |  |
| HGLibA_18434 | Rep 1 | GAGE10 | 2 | | 51.857 | 638.36 | 51.857 | 638.36 | 2.25E+05 | 4021.4 | 9.2487 | 1 | 1.43E-20 | 2.87E-20 | 3.41E-19 | TRUE |  |  |  |  |  |  |  |
| HGLibA_18433 | Rep 3 | GAGE10 | 2 | | 140.71 | 1316.4 | 140.71 | 1316.4 | 8.69E+05 | 7735.9 | 13.367 | 1 | 4.97E-41 | 9.95E-41 | 3.35E-39 | TRUE |  |  |  |  |  |  |  |
| HGLibA_18435 | Rep 3 | GAGE10 | 2 | | 54.647 | 292.54 | 54.647 | 292.54 | 32736 | 1456.9 | 6.2325 | 1 | 2.48E-10 | 4.97E-10 | 5.40E-09 | TRUE |  |  |  |  |  |  |  |
| HGLibA_18531 | Rep 2 | GALNT10 | 2 | | 115.13 | 664.44 | 115.13 | 664.44 | 1.76E+05 | 9871.9 | 5.5286 | 1 | 1.84E-08 | 3.68E-08 | 3.47E-07 | TRUE |  |  |  |  |  |  |  |
| HGLibA_18529 | Rep 2 | GALNT10 | 2 | | 532.29 | 2378.9 | 532.29 | 2378.9 | 1.92E+06 | 1.62E+05 | 4.5862 | 1 | 2.49E-06 | 4.98E-06 | 3.61E-05 | TRUE |  |  |  |  |  |  |  |
| HGLibA_18530 | Rep 3 | GALNT10 | 2 | | 122.36 | 755.72 | 122.36 | 755.72 | 2.37E+05 | 6039.6 | 8.1498 | 1 | 1.93E-16 | 3.87E-16 | 6.35E-15 | TRUE |  |  |  |  |  |  |  |
| HGLibA_18529 | Rep 3 | GALNT10 | 2 | | 148.7 | 682.58 | 148.7 | 682.58 | 1.61E+05 | 8530.8 | 5.7803 | 1 | 3.94E-09 | 7.88E-09 | 7.76E-08 | TRUE |  |  |  |  |  |  |  |
| HGLibA_18874 | Rep 1 | GDF7 | 2 | | 25.18 | 178.74 | 25.18 | 178.74 | 14215 | 896.75 | 5.1279 | 1 | 1.83E-07 | 3.66E-07 | 2.17E-06 | TRUE |  |  |  |  |  |  |  |
| HGLibA_18875 | Rep 1 | GDF7 | 2 | | 24.828 | 842.63 | 24.828 | 842.63 | 5.01E+05 | 871.01 | 27.71 | 1 | 3.30E-169 | 6.60E-169 | 2.90E-167 | TRUE |  |  |  |  |  |  |  |
| HGLibA_18874 | Rep 3 | GDF7 | 2 | | 70.512 | 450.99 | 70.512 | 450.99 | 85955 | 2281.3 | 7.966 | 1 | 8.81E-16 | 1.76E-15 | 2.77E-14 | TRUE |  |  |  |  |  |  |  |
| HGLibA_18876 | Rep 3 | GDF7 | 2 | | 161.35 | 609.45 | 161.35 | 609.45 | 1.11E+05 | 9859.6 | 4.5128 | 1 | 3.37E-06 | 6.75E-06 | 4.86E-05 | TRUE |  |  |  |  |  |  |  |
| HGLibA_19016 | Rep 1 | GGT6 | 2 | | 11.446 | 102.14 | 11.446 | 102.14 | 5128.1 | 178.05 | 6.7967 | 1 | 6.65E-12 | 1.33E-11 | 1.08E-10 | TRUE |  |  |  |  |  |  |  |
| HGLibA_19017 | Rep 1 | GGT6 | 2 | | 41.996 | 510.69 | 41.996 | 510.69 | 1.44E+05 | 2591.8 | 9.2062 | 1 | 2.12E-20 | 4.25E-20 | 5.03E-19 | TRUE |  |  |  |  |  |  |  |
| HGLibA_19015 | Rep 3 | GGT6 | 2 | | 298.23 | 1523.6 | 298.23 | 1523.6 | 8.63E+05 | 29353 | 7.1524 | 1 | 4.44E-13 | 8.89E-13 | 1.19E-11 | TRUE |  |  |  |  |  |  |  |
| HGLibA_19017 | Rep 3 | GGT6 | 2 | | 115.83 | 585.07 | 115.83 | 585.07 | 1.26E+05 | 5480.7 | 6.3384 | 1 | 1.23E-10 | 2.47E-10 | 2.74E-09 | TRUE |  |  |  |  |  |  |  |
| HGLibA_19430 | Rep 2 | GMPR2 | 2 | | 52.046 | 622.03 | 52.046 | 622.03 | 2.12E+05 | 2327.9 | 11.814 | 1 | 1.93E-32 | 3.86E-32 | 1.50E-30 | TRUE |  |  |  |  |  |  |  |
| HGLibA_19429 | Rep 2 | GMPR2 | 2 | | 1062.2 | 5046.9 | 1062.2 | 5046.9 | 9.03E+06 | 5.75E+05 | 5.2569 | 1 | 7.97E-08 | 1.59E-07 | 1.39E-06 | TRUE |  |  |  |  |  |  |  |
| HGLibA_19430 | Rep 3 | GMPR2 | 2 | | 43.863 | 572.88 | 43.863 | 572.88 | 1.85E+05 | 990.74 | 16.807 | 1 | 1.18E-63 | 2.36E-63 | 1.05E-61 | TRUE |  |  |  |  |  |  |  |
| HGLibA_19431 | Rep 3 | GMPR2 | 2 | | 105.87 | 548.5 | 105.87 | 548.5 | 1.13E+05 | 4675.1 | 6.4736 | 1 | 5.09E-11 | 1.02E-10 | 1.17E-09 | TRUE |  |  |  |  |  |  |  |
| HGLibA_19467 | Rep 2 | GNAQ | 2 | | 223.17 | 2425.2 | 223.17 | 2425.2 | 3.12E+06 | 33054 | 12.112 | 1 | 5.14E-34 | 1.03E-33 | 4.21E-32 | TRUE |  |  |  |  |  |  |  |
| HGLibA_19466 | Rep 2 | GNAQ | 2 | | 655.31 | 2912.2 | 655.31 | 2912.2 | 2.87E+06 | 2.37E+05 | 4.6338 | 1 | 1.97E-06 | 3.94E-06 | 2.90E-05 | TRUE |  |  |  |  |  |  |  |
| HGLibA_19466 | Rep 3 | GNAQ | 2 | | 151.6 | 597.26 | 151.6 | 597.26 | 1.10E+05 | 8828.3 | 4.7431 | 1 | 1.11E-06 | 2.22E-06 | 1.70E-05 | TRUE |  |  |  |  |  |  |  |
| HGLibA_19467 | Rep 3 | GNAQ | 2 | | 23.746 | 109.7 | 23.746 | 109.7 | 4184.2 | 340.16 | 4.6604 | 1 | 1.75E-06 | 3.50E-06 | 2.61E-05 | TRUE |  |  |  |  |  |  |  |
| HGLibA_19544 | Rep 1 | GNL1 | 2 | | 42.436 | 280.88 | 42.436 | 280.88 | 33925 | 2648.7 | 4.633 | 1 | 2.27E-06 | 4.53E-06 | 2.44E-05 | TRUE |  |  |  |  |  |  |  |
| HGLibA_19545 | Rep 1 | GNL1 | 2 | | 33.896 | 229.81 | 33.896 | 229.81 | 22983 | 1660.3 | 4.8081 | 1 | 9.56E-07 | 1.91E-06 | 1.06E-05 | TRUE |  |  |  |  |  |  |  |
| HGLibA_19545 | Rep 2 | GNL1 | 2 | | 157.72 | 2073 | 157.72 | 2073 | 2.42E+06 | 17531 | 14.465 | 1 | 1.13E-47 | 2.27E-47 | 1.34E-45 | TRUE |  |  |  |  |  |  |  |
| HGLibA_19543 | Rep 2 | GNL1 | 2 | | 351.71 | 1884.1 | 351.71 | 1884.1 | 1.36E+06 | 75940 | 5.5607 | 1 | 1.49E-08 | 2.99E-08 | 2.84E-07 | TRUE |  |  |  |  |  |  |  |
| HGLibA_19628 | Rep 2 | GOLGA6L6 | 2 | | 846.15 | 5708.8 | 846.15 | 5708.8 | 1.42E+07 | 3.79E+05 | 7.9006 | 1 | 1.52E-15 | 3.03E-15 | 5.19E-14 | TRUE |  |  |  |  |  |  |  |
| HGLibA_19629 | Rep 2 | GOLGA6L6 | 2 | | 600.11 | 2620.5 | 600.11 | 2620.5 | 2.29E+06 | 2.02E+05 | 4.4962 | 1 | 3.80E-06 | 7.61E-06 | 5.38E-05 | TRUE |  |  |  |  |  |  |  |
| HGLibA_19627 | Rep 3 | GOLGA6L6 | 2 | | 14.517 | 85.323 | 14.517 | 85.323 | 2938 | 145.9 | 5.8619 | 1 | 2.58E-09 | 5.17E-09 | 5.17E-08 | TRUE |  |  |  |  |  |  |  |
| HGLibA_19629 | Rep 3 | GOLGA6L6 | 2 | | 104.01 | 450.99 | 104.01 | 450.99 | 67616 | 4530.4 | 5.1552 | 1 | 1.35E-07 | 2.70E-07 | 2.30E-06 | TRUE |  |  |  |  |  |  |  |
| HGLibA_19700 | Rep 1 | GP1BA | 2 | | 25.973 | 357.48 | 25.973 | 357.48 | 73102 | 956.09 | 10.721 | 1 | 5.06E-27 | 1.01E-26 | 1.47E-25 | TRUE |  |  |  |  |  |  |  |
| HGLibA_19698 | Rep 1 | GP1BA | 2 | | 30.375 | 740.49 | 30.375 | 740.49 | 3.63E+05 | 1322.3 | 19.528 | 1 | 3.95E-85 | 7.90E-85 | 2.47E-83 | TRUE |  |  |  |  |  |  |  |
| HGLibA_19698 | Rep 2 | GP1BA | 2 | | 139.58 | 1381.6 | 139.58 | 1381.6 | 9.78E+05 | 14027 | 10.487 | 1 | 5.64E-26 | 1.13E-25 | 3.41E-24 | TRUE |  |  |  |  |  |  |  |
| HGLibA_19700 | Rep 2 | GP1BA | 2 | | 173.49 | 1642.5 | 173.49 | 1642.5 | 1.36E+06 | 20864 | 10.17 | 1 | 1.53E-24 | 3.05E-24 | 8.70E-23 | TRUE |  |  |  |  |  |  |  |
| HGLibA_19701 | Rep 1 | GP1BB | 2 | | 23.067 | 153.21 | 23.067 | 153.21 | 10109 | 748.2 | 4.7577 | 1 | 1.22E-06 | 2.45E-06 | 1.35E-05 | TRUE |  |  |  |  |  |  |  |
| HGLibA_19702 | Rep 1 | GP1BB | 2 | | 18.137 | 459.62 | 18.137 | 459.62 | 1.41E+05 | 455.91 | 20.676 | 1 | 3.53E-95 | 7.06E-95 | 2.33E-93 | TRUE |  |  |  |  |  |  |  |
| HGLibA_19701 | Rep 3 | GP1BB | 2 | | 37.641 | 438.8 | 37.641 | 438.8 | 1.05E+05 | 758.13 | 14.57 | 1 | 2.40E-48 | 4.80E-48 | 1.79E-46 | TRUE |  |  |  |  |  |  |  |
| HGLibA_19702 | Rep 3 | GP1BB | 2 | | 46.248 | 255.97 | 46.248 | 255.97 | 25564 | 1087 | 6.3609 | 1 | 1.09E-10 | 2.18E-10 | 2.43E-09 | TRUE |  |  |  |  |  |  |  |
| HGLibA_19897 | Rep 1 | GPR137 | 2 | | 41.732 | 357.48 | 41.732 | 357.48 | 61830 | 2558 | 6.2429 | 1 | 2.70E-10 | 5.40E-10 | 3.99E-09 | TRUE |  |  |  |  |  |  |  |
| HGLibA_19896 | Rep 1 | GPR137 | 2 | | 22.715 | 229.81 | 22.715 | 229.81 | 27262 | 724.81 | 7.6923 | 1 | 9.03E-15 | 1.81E-14 | 1.71E-13 | TRUE |  |  |  |  |  |  |  |
| HGLibA_19898 | Rep 3 | GPR137 | 2 | | 122.57 | 743.53 | 122.57 | 743.53 | 2.27E+05 | 6057.7 | 7.9783 | 1 | 7.87E-16 | 1.57E-15 | 2.48E-14 | TRUE |  |  |  |  |  |  |  |
| HGLibA_19896 | Rep 3 | GPR137 | 2 | | 26.131 | 146.27 | 26.131 | 146.27 | 8401 | 401.54 | 5.9953 | 1 | 1.12E-09 | 2.25E-09 | 2.32E-08 | TRUE |  |  |  |  |  |  |  |
| HGLibA_19926 | Rep 1 | GPR15 | 2 | | 86.546 | 663.89 | 86.546 | 663.89 | 2.03E+05 | 11716 | 5.3339 | 1 | 6.10E-08 | 1.22E-07 | 7.55E-07 | TRUE |  |  |  |  |  |  |  |
| HGLibA_19928 | Rep 1 | GPR15 | 2 | | 41.996 | 536.22 | 41.996 | 536.22 | 1.61E+05 | 2591.8 | 9.7078 | 1 | 1.76E-22 | 3.51E-22 | 4.47E-21 | TRUE |  |  |  |  |  |  |  |
| HGLibA_19928 | Rep 3 | GPR15 | 2 | | 47.077 | 1511.4 | 47.077 | 1511.4 | 1.60E+06 | 1121.5 | 43.728 | 1 | 0 | 0 | 0 | TRUE |  |  |  |  |  |  |  |
| HGLibA_19927 | Rep 3 | GPR15 | 2 | | 103.18 | 426.61 | 103.18 | 426.61 | 58371 | 4466.7 | 4.8395 | 1 | 6.93E-07 | 1.39E-06 | 1.09E-05 | TRUE |  |  |  |  |  |  |  |
| HGLibA_20286 | Rep 1 | GRIK3 | 2 | | 64.007 | 740.49 | 64.007 | 740.49 | 2.97E+05 | 6238.4 | 8.5649 | 1 | 6.84E-18 | 1.37E-17 | 1.48E-16 | TRUE |  |  |  |  |  |  |  |
| HGLibA_20287 | Rep 1 | GRIK3 | 2 | | 26.325 | 357.48 | 26.325 | 357.48 | 72805 | 983.11 | 10.562 | 1 | 2.81E-26 | 5.61E-26 | 7.91E-25 | TRUE |  |  |  |  |  |  |  |
| HGLibA_20287 | Rep 3 | GRIK3 | 2 | | 66.676 | 390.05 | 66.676 | 390.05 | 61277 | 2067.2 | 7.1123 | 1 | 6.15E-13 | 1.23E-12 | 1.64E-11 | TRUE |  |  |  |  |  |  |  |
| HGLibA_20286 | Rep 3 | GRIK3 | 2 | | 169.44 | 731.34 | 169.44 | 731.34 | 1.77E+05 | 10753 | 5.4187 | 1 | 3.16E-08 | 6.33E-08 | 5.75E-07 | TRUE |  |  |  |  |  |  |  |
| HGLibA_20724 | Rep 1 | GZMH | 2 | | 27.645 | 178.74 | 27.645 | 178.74 | 13573 | 1087.9 | 4.5809 | 1 | 2.90E-06 | 5.80E-06 | 3.07E-05 | TRUE |  |  |  |  |  |  |  |
| HGLibA_20723 | Rep 1 | GZMH | 2 | | 29.582 | 229.81 | 29.582 | 229.81 | 24498 | 1251.8 | 5.6592 | 1 | 9.52E-09 | 1.90E-08 | 1.26E-07 | TRUE |  |  |  |  |  |  |  |
| HGLibA_20724 | Rep 2 | GZMH | 2 | | 161.66 | 1037.1 | 161.66 | 1037.1 | 4.55E+05 | 18340 | 6.4648 | 1 | 5.74E-11 | 1.15E-10 | 1.38E-09 | TRUE |  |  |  |  |  |  |  |
| HGLibA_20723 | Rep 2 | GZMH | 2 | | 356.44 | 1789 | 356.44 | 1789 | 1.18E+06 | 77820 | 5.1353 | 1 | 1.57E-07 | 3.13E-07 | 2.65E-06 | TRUE |  |  |  |  |  |  |  |
| HGLibA_21071 | Rep 2 | HEATR3 | 2 | | 104.88 | 882.93 | 104.88 | 882.93 | 3.75E+05 | 8328.3 | 8.5256 | 1 | 8.69E-18 | 1.74E-17 | 3.43E-16 | TRUE |  |  |  |  |  |  |  |
| HGLibA_21072 | Rep 2 | HEATR3 | 2 | | 670.29 | 3779.7 | 670.29 | 3779.7 | 5.64E+06 | 2.47E+05 | 6.2534 | 1 | 2.20E-10 | 4.41E-10 | 5.02E-09 | TRUE |  |  |  |  |  |  |  |
| HGLibA_21071 | Rep 3 | HEATR3 | 2 | | 43.448 | 353.48 | 43.448 | 353.48 | 59154 | 974.39 | 9.9321 | 1 | 1.64E-23 | 3.29E-23 | 7.36E-22 | TRUE |  |  |  |  |  |  |  |
| HGLibA_21072 | Rep 3 | HEATR3 | 2 | | 104.63 | 450.99 | 104.63 | 450.99 | 67319 | 4578.4 | 5.1189 | 1 | 1.64E-07 | 3.27E-07 | 2.76E-06 | TRUE |  |  |  |  |  |  |  |
| HGLibA_21496 | Rep 1 | HIST1H4D | 2 | | 31.959 | 204.27 | 31.959 | 204.27 | 17625 | 1469.4 | 4.4952 | 1 | 4.36E-06 | 8.71E-06 | 4.55E-05 | TRUE |  |  |  |  |  |  |  |
| HGLibA_21494 | Rep 1 | HIST1H4D | 2 | | 62.95 | 561.75 | 62.95 | 561.75 | 1.55E+05 | 6025.4 | 6.4259 | 1 | 8.28E-11 | 1.66E-10 | 1.26E-09 | TRUE |  |  |  |  |  |  |  |
| HGLibA_21495 | Rep 3 | HIST1H4D | 2 | | 83.578 | 621.64 | 83.578 | 621.64 | 1.76E+05 | 3078.7 | 9.6972 | 1 | 1.66E-22 | 3.32E-22 | 7.04E-21 | TRUE |  |  |  |  |  |  |  |
| HGLibA_21494 | Rep 3 | HIST1H4D | 2 | | 76.215 | 329.1 | 76.215 | 329.1 | 35893 | 2616.5 | 4.9438 | 1 | 4.11E-07 | 8.22E-07 | 6.62E-06 | TRUE |  |  |  |  |  |  |  |
| HGLibA_21707 | Rep 2 | HMGCR | 2 | | 354.07 | 4346.5 | 354.07 | 4346.5 | 1.04E+07 | 76877 | 14.399 | 1 | 2.91E-47 | 5.82E-47 | 3.39E-45 | TRUE |  |  |  |  |  |  |  |
| HGLibA_21708 | Rep 2 | HMGCR | 2 | | 475.51 | 3612.7 | 475.51 | 3612.7 | 6.00E+06 | 1.32E+05 | 8.6388 | 1 | 3.14E-18 | 6.28E-18 | 1.27E-16 | TRUE |  |  |  |  |  |  |  |
| HGLibA_21707 | Rep 3 | HMGCR | 2 | | 45.211 | 243.78 | 45.211 | 243.78 | 22831 | 1044.7 | 6.1435 | 1 | 4.39E-10 | 8.78E-10 | 9.38E-09 | TRUE |  |  |  |  |  |  |  |
| HGLibA_21708 | Rep 3 | HMGCR | 2 | | 106.91 | 414.43 | 106.91 | 414.43 | 52314 | 4756.4 | 4.4589 | 1 | 4.38E-06 | 8.77E-06 | 6.21E-05 | TRUE |  |  |  |  |  |  |  |
| HGLibA_22146 | Rep 1 | HS6ST2 | 2 | | 36.89 | 255.34 | 36.89 | 255.34 | 28665 | 1979.4 | 4.9102 | 1 | 5.71E-07 | 1.14E-06 | 6.49E-06 | TRUE |  |  |  |  |  |  |  |
| HGLibA_22144 | Rep 1 | HS6ST2 | 2 | | 37.858 | 663.89 | 37.858 | 663.89 | 2.70E+05 | 2088.9 | 13.697 | 1 | 6.60E-43 | 1.32E-42 | 2.61E-41 | TRUE |  |  |  |  |  |  |  |
| HGLibA_22144 | Rep 3 | HS6ST2 | 2 | | 65.12 | 487.56 | 65.12 | 487.56 | 1.08E+05 | 1983.1 | 9.4862 | 1 | 1.29E-21 | 2.58E-21 | 5.30E-20 | TRUE |  |  |  |  |  |  |  |
| HGLibA_22146 | Rep 3 | HS6ST2 | 2 | | 62.32 | 463.18 | 62.32 | 463.18 | 97568 | 1835.5 | 9.3566 | 1 | 4.44E-21 | 8.88E-21 | 1.79E-19 | TRUE |  |  |  |  |  |  |  |
| HGLibA_22473 | Rep 1 | IARS | 2 | | 32.312 | 229.81 | 32.312 | 229.81 | 23523 | 1503.2 | 5.0939 | 1 | 2.20E-07 | 4.40E-07 | 2.58E-06 | TRUE |  |  |  |  |  |  |  |
| HGLibA_22474 | Rep 1 | IARS | 2 | | 41.116 | 383.01 | 41.116 | 383.01 | 73414 | 2480.1 | 6.8654 | 1 | 4.17E-12 | 8.34E-12 | 6.85E-11 | TRUE |  |  |  |  |  |  |  |
| HGLibA_22473 | Rep 3 | IARS | 2 | | 26.857 | 219.4 | 26.857 | 219.4 | 22825 | 421.1 | 9.383 | 1 | 3.54E-21 | 7.09E-21 | 1.44E-19 | TRUE |  |  |  |  |  |  |  |
| HGLibA_22474 | Rep 3 | IARS | 2 | | 80.882 | 341.29 | 80.882 | 341.29 | 37941 | 2905.7 | 4.831 | 1 | 7.28E-07 | 1.46E-06 | 1.14E-05 | TRUE |  |  |  |  |  |  |  |
| HGLibA_22632 | Rep 1 | IFIT3 | 2 | | 85.489 | 587.29 | 85.489 | 587.29 | 1.51E+05 | 11419 | 4.6959 | 1 | 1.68E-06 | 3.37E-06 | 1.83E-05 | TRUE |  |  |  |  |  |  |  |
| HGLibA_22633 | Rep 1 | IFIT3 | 2 | | 68.585 | 536.22 | 68.585 | 536.22 | 1.34E+05 | 7206.4 | 5.5087 | 1 | 2.29E-08 | 4.57E-08 | 2.93E-07 | TRUE |  |  |  |  |  |  |  |
| HGLibA_22633 | Rep 2 | IFIT3 | 2 | | 506.27 | 2453.4 | 506.27 | 2453.4 | 2.16E+06 | 1.48E+05 | 5.063 | 1 | 2.28E-07 | 4.56E-07 | 3.77E-06 | TRUE |  |  |  |  |  |  |  |
| HGLibA_22631 | Rep 2 | IFIT3 | 2 | | 575.66 | 2605.1 | 575.66 | 2605.1 | 2.33E+06 | 1.87E+05 | 4.6915 | 1 | 1.49E-06 | 2.99E-06 | 2.23E-05 | TRUE |  |  |  |  |  |  |  |
| HGLibA_23172 | Rep 1 | IL4R | 2 | | 17.168 | 153.21 | 17.168 | 153.21 | 11543 | 407.32 | 6.7405 | 1 | 9.83E-12 | 1.97E-11 | 1.59E-10 | TRUE |  |  |  |  |  |  |  |
| HGLibA_23171 | Rep 1 | IL4R | 2 | | 64.271 | 689.43 | 64.271 | 689.43 | 2.51E+05 | 6292.3 | 7.881 | 1 | 2.05E-15 | 4.10E-15 | 3.98E-14 | TRUE |  |  |  |  |  |  |  |
| HGLibA_23173 | Rep 3 | IL4R | 2 | | 60.35 | 719.15 | 60.35 | 719.15 | 2.83E+05 | 1734.6 | 15.818 | 1 | 1.26E-56 | 2.53E-56 | 1.06E-54 | TRUE |  |  |  |  |  |  |  |
| HGLibA_23171 | Rep 3 | IL4R | 2 | | 88.348 | 767.91 | 88.348 | 767.91 | 2.87E+05 | 3395.7 | 11.662 | 1 | 1.07E-31 | 2.14E-31 | 6.02E-30 | TRUE |  |  |  |  |  |  |  |
| HGLibA_23642 | Rep 1 | IRX6 | 2 | | 132.68 | 1123.5 | 132.68 | 1123.5 | 6.08E+05 | 28641 | 5.8547 | 1 | 3.05E-09 | 6.10E-09 | 4.19E-08 | TRUE |  |  |  |  |  |  |  |
| HGLibA_23643 | Rep 1 | IRX6 | 2 | | 25.444 | 280.88 | 25.444 | 280.88 | 42019 | 916.31 | 8.4383 | 1 | 2.01E-17 | 4.03E-17 | 4.26E-16 | TRUE |  |  |  |  |  |  |  |
| HGLibA_23643 | Rep 2 | IRX6 | 2 | | 240.52 | 1831.4 | 240.52 | 1831.4 | 1.54E+06 | 37901 | 8.1717 | 1 | 1.71E-16 | 3.41E-16 | 6.19E-15 | TRUE |  |  |  |  |  |  |  |
| HGLibA_23642 | Rep 2 | IRX6 | 2 | | 696.32 | 3098.6 | 696.32 | 3098.6 | 3.25E+06 | 2.65E+05 | 4.6656 | 1 | 1.69E-06 | 3.37E-06 | 2.50E-05 | TRUE |  |  |  |  |  |  |  |
| HGLibA_23646 | Rep 2 | ISCA1 | 2 | | 208.19 | 1091.1 | 208.19 | 1091.1 | 4.50E+05 | 29111 | 5.1749 | 1 | 1.28E-07 | 2.57E-07 | 2.19E-06 | TRUE |  |  |  |  |  |  |  |
| HGLibA_23645 | Rep 2 | ISCA1 | 2 | | 951.03 | 4184.6 | 951.03 | 4184.6 | 5.88E+06 | 4.69E+05 | 4.7205 | 1 | 1.28E-06 | 2.56E-06 | 1.93E-05 | TRUE |  |  |  |  |  |  |  |
| HGLibA_23647 | Rep 3 | ISCA1 | 2 | | 4.9773 | 158.46 | 4.9773 | 158.46 | 17490 | 24.393 | 31.075 | 1 | 3.08E-212 | 6.15E-212 | 4.63E-210 | TRUE |  |  |  |  |  |  |  |
| HGLibA_23646 | Rep 3 | ISCA1 | 2 | | 48.736 | 280.35 | 48.736 | 280.35 | 31355 | 1191.7 | 6.7093 | 1 | 1.06E-11 | 2.12E-11 | 2.57E-10 | TRUE |  |  |  |  |  |  |  |
| HGLibA_24222 | Rep 1 | KCNH5 | 2 | | 68.321 | 510.69 | 68.321 | 510.69 | 1.19E+05 | 7148.5 | 5.2321 | 1 | 1.06E-07 | 2.12E-07 | 1.28E-06 | TRUE |  |  |  |  |  |  |  |
| HGLibA_24221 | Rep 1 | KCNH5 | 2 | | 33.896 | 536.22 | 33.896 | 536.22 | 1.71E+05 | 1660.3 | 12.328 | 1 | 4.01E-35 | 8.03E-35 | 1.39E-33 | TRUE |  |  |  |  |  |  |  |
| HGLibA_24223 | Rep 2 | KCNH5 | 2 | | 372.21 | 3752.8 | 372.21 | 3752.8 | 7.26E+06 | 84235 | 11.648 | 1 | 1.31E-31 | 2.62E-31 | 9.88E-30 | TRUE |  |  |  |  |  |  |  |
| HGLibA_24221 | Rep 2 | KCNH5 | 2 | | 161.66 | 1050 | 161.66 | 1050 | 4.70E+05 | 18340 | 6.5597 | 1 | 3.05E-11 | 6.10E-11 | 7.51E-10 | TRUE |  |  |  |  |  |  |  |
| HGLibA_24492 | Rep 1 | KDM1B | 2 | | 125.72 | 1072.4 | 125.72 | 1072.4 | 5.56E+05 | 25588 | 5.9183 | 1 | 2.07E-09 | 4.15E-09 | 2.89E-08 | TRUE |  |  |  |  |  |  |  |
| HGLibA_24491 | Rep 1 | KDM1B | 2 | | 27.557 | 280.88 | 27.557 | 280.88 | 40841 | 1080.8 | 7.7055 | 1 | 8.16E-15 | 1.63E-14 | 1.55E-13 | TRUE |  |  |  |  |  |  |  |
| HGLibA_24491 | Rep 3 | KDM1B | 2 | | 32.249 | 158.46 | 32.249 | 158.46 | 9100.4 | 578.88 | 5.2456 | 1 | 8.56E-08 | 1.71E-07 | 1.49E-06 | TRUE |  |  |  |  |  |  |  |
| HGLibA_24492 | Rep 3 | KDM1B | 2 | | 210.29 | 865.42 | 210.29 | 865.42 | 2.39E+05 | 15777 | 5.2156 | 1 | 9.61E-08 | 1.92E-07 | 1.67E-06 | TRUE |  |  |  |  |  |  |  |
| HGLibA_25294 | Rep 1 | KLRC4 | 2 | | 50.448 | 383.01 | 50.448 | 383.01 | 67365 | 3797.1 | 5.397 | 1 | 4.27E-08 | 8.54E-08 | 5.34E-07 | TRUE |  |  |  |  |  |  |  |
| HGLibA_25295 | Rep 1 | KLRC4 | 2 | | 71.755 | 740.49 | 71.755 | 740.49 | 2.85E+05 | 7919.4 | 7.5147 | 1 | 3.61E-14 | 7.22E-14 | 6.62E-13 | TRUE |  |  |  |  |  |  |  |
| HGLibA_25293 | Rep 2 | KLRC4 | 2 | | 885.58 | 4810.5 | 885.58 | 4810.5 | 8.93E+06 | 4.12E+05 | 6.1165 | 1 | 5.22E-10 | 1.04E-09 | 1.15E-08 | TRUE |  |  |  |  |  |  |  |
| HGLibA_25295 | Rep 2 | KLRC4 | 2 | | 492.07 | 2389.2 | 492.07 | 2389.2 | 2.05E+06 | 1.40E+05 | 5.0629 | 1 | 2.28E-07 | 4.56E-07 | 3.77E-06 | TRUE |  |  |  |  |  |  |  |
| HGLibA_25365 | Rep 2 | KPNA5 | 2 | | 159.29 | 1949.6 | 159.29 | 1949.6 | 2.10E+06 | 17853 | 13.399 | 1 | 3.45E-41 | 6.90E-41 | 3.44E-39 | TRUE |  |  |  |  |  |  |  |
| HGLibA_25367 | Rep 2 | KPNA5 | 2 | | 211.34 | 972.89 | 211.34 | 972.89 | 3.28E+05 | 29922 | 4.4025 | 1 | 6.02E-06 | 1.20E-05 | 8.27E-05 | TRUE |  |  |  |  |  |  |  |
| HGLibA_25365 | Rep 3 | KPNA5 | 2 | | 85.859 | 609.45 | 85.859 | 609.45 | 1.65E+05 | 3228.6 | 9.2148 | 1 | 1.67E-20 | 3.34E-20 | 6.56E-19 | TRUE |  |  |  |  |  |  |  |
| HGLibA_25367 | Rep 3 | KPNA5 | 2 | | 53.817 | 316.91 | 53.817 | 316.91 | 40600 | 1418.3 | 6.9861 | 1 | 1.53E-12 | 3.06E-12 | 3.94E-11 | TRUE |  |  |  |  |  |  |  |
| HGLibA_25528 | Rep 2 | KRT72 | 2 | | 830.38 | 4638.2 | 830.38 | 4638.2 | 8.44E+06 | 3.66E+05 | 6.2944 | 1 | 1.69E-10 | 3.37E-10 | 3.88E-09 | TRUE |  |  |  |  |  |  |  |
| HGLibA_25529 | Rep 2 | KRT72 | 2 | | 394.29 | 2278.6 | 394.29 | 2278.6 | 2.08E+06 | 93603 | 6.1591 | 1 | 4.06E-10 | 8.12E-10 | 9.01E-09 | TRUE |  |  |  |  |  |  |  |
| HGLibA_25529 | Rep 3 | KRT72 | 2 | | 97.888 | 633.83 | 97.888 | 633.83 | 1.71E+05 | 4070 | 8.4008 | 1 | 2.37E-17 | 4.73E-17 | 8.09E-16 | TRUE |  |  |  |  |  |  |  |
| HGLibA_25527 | Rep 3 | KRT72 | 2 | | 109.92 | 560.69 | 109.92 | 560.69 | 1.17E+05 | 4995.6 | 6.3778 | 1 | 9.56E-11 | 1.91E-10 | 2.14E-09 | TRUE |  |  |  |  |  |  |  |
| HGLibA_25762 | Rep 1 | KRTAP4-3 | 2 | | 48.071 | 612.82 | 48.071 | 612.82 | 2.10E+05 | 3433.8 | 9.6376 | 1 | 3.49E-22 | 6.98E-22 | 8.74E-21 | TRUE |  |  |  |  |  |  |  |
| HGLibA_25761 | Rep 1 | KRTAP4-3 | 2 | | 15.848 | 383.01 | 15.848 | 383.01 | 96865 | 345.67 | 19.748 | 1 | 5.15E-87 | 1.03E-86 | 3.26E-85 | TRUE |  |  |  |  |  |  |  |
| HGLibA_25760 | Rep 2 | KRTAP4-3 | 2 | | 259.44 | 2331.3 | 259.44 | 2331.3 | 2.68E+06 | 43530 | 9.9305 | 1 | 1.72E-23 | 3.43E-23 | 9.31E-22 | TRUE |  |  |  |  |  |  |  |
| HGLibA_25761 | Rep 2 | KRTAP4-3 | 2 | | 262.6 | 1280 | 262.6 | 1280 | 5.91E+05 | 44503 | 4.823 | 1 | 7.91E-07 | 1.58E-06 | 1.22E-05 | TRUE |  |  |  |  |  |  |  |
| HGLibA_25976 | Rep 1 | LAMP1 | 2 | | 60.661 | 510.69 | 60.661 | 510.69 | 1.25E+05 | 5577.2 | 6.026 | 1 | 1.06E-09 | 2.12E-09 | 1.51E-08 | TRUE |  |  |  |  |  |  |  |
| HGLibA_25978 | Rep 1 | LAMP1 | 2 | | 12.062 | 510.69 | 12.062 | 510.69 | 1.91E+05 | 198.05 | 35.431 | 1 | 3.50E-275 | 7.00E-275 | 3.87E-273 | TRUE |  |  |  |  |  |  |  |
| HGLibA_25978 | Rep 2 | LAMP1 | 2 | | 146.68 | 2208 | 146.68 | 2208 | 2.86E+06 | 15356 | 16.634 | 1 | 2.23E-62 | 4.47E-62 | 3.44E-60 | TRUE |  |  |  |  |  |  |  |
| HGLibA_25977 | Rep 2 | LAMP1 | 2 | | 205.03 | 1385.4 | 205.03 | 1385.4 | 8.34E+05 | 28310 | 7.0155 | 1 | 1.29E-12 | 2.58E-12 | 3.56E-11 | TRUE |  |  |  |  |  |  |  |
| HGLibA_26279 | Rep 2 | LEKR1 | 2 | | 233.42 | 1679.7 | 233.42 | 1679.7 | 1.26E+06 | 35882 | 7.6354 | 1 | 1.26E-14 | 2.53E-14 | 4.05E-13 | TRUE |  |  |  |  |  |  |  |
| HGLibA_26280 | Rep 2 | LEKR1 | 2 | | 843.78 | 4229.6 | 843.78 | 4229.6 | 6.57E+06 | 3.77E+05 | 5.5152 | 1 | 1.90E-08 | 3.81E-08 | 3.58E-07 | TRUE |  |  |  |  |  |  |  |
| HGLibA_26281 | Rep 3 | LEKR1 | 2 | | 22.709 | 170.65 | 22.709 | 170.65 | 13305 | 314.87 | 8.337 | 1 | 4.24E-17 | 8.47E-17 | 1.43E-15 | TRUE |  |  |  |  |  |  |  |
| HGLibA_26280 | Rep 3 | LEKR1 | 2 | | 120.39 | 511.94 | 120.39 | 511.94 | 85870 | 5868.5 | 5.1112 | 1 | 1.70E-07 | 3.40E-07 | 2.86E-06 | TRUE |  |  |  |  |  |  |  |
| HGLibA_26444 | Rep 1 | LHPP | 2 | | 27.117 | 178.74 | 27.117 | 178.74 | 13707 | 1045.3 | 4.6896 | 1 | 1.71E-06 | 3.43E-06 | 1.86E-05 | TRUE |  |  |  |  |  |  |  |
| HGLibA_26443 | Rep 1 | LHPP | 2 | | 40.059 | 638.36 | 40.059 | 638.36 | 2.43E+05 | 2349.3 | 12.344 | 1 | 3.31E-35 | 6.62E-35 | 1.15E-33 | TRUE |  |  |  |  |  |  |  |
| HGLibA_26444 | Rep 2 | LHPP | 2 | | 308.34 | 1759.4 | 308.34 | 1759.4 | 1.23E+06 | 59692 | 5.9393 | 1 | 1.60E-09 | 3.19E-09 | 3.35E-08 | TRUE |  |  |  |  |  |  |  |
| HGLibA_26443 | Rep 2 | LHPP | 2 | | 134.85 | 758.26 | 134.85 | 758.26 | 2.26E+05 | 13171 | 5.4321 | 1 | 3.17E-08 | 6.33E-08 | 5.82E-07 | TRUE |  |  |  |  |  |  |  |
| HGLibA_27382 | Rep 2 | LRRC4C | 2 | | 111.19 | 1177.2 | 111.19 | 1177.2 | 7.28E+05 | 9264.1 | 11.076 | 1 | 9.39E-29 | 1.88E-28 | 6.36E-27 | TRUE |  |  |  |  |  |  |  |
| HGLibA_27384 | Rep 2 | LRRC4C | 2 | | 637.96 | 3670.5 | 637.96 | 3670.5 | 5.38E+06 | 2.26E+05 | 6.3811 | 1 | 9.66E-11 | 1.93E-10 | 2.27E-09 | TRUE |  |  |  |  |  |  |  |
| HGLibA_27382 | Rep 3 | LRRC4C | 2 | | 75.179 | 365.67 | 75.179 | 365.67 | 48152 | 2554.1 | 5.748 | 1 | 4.85E-09 | 9.70E-09 | 9.47E-08 | TRUE |  |  |  |  |  |  |  |
| HGLibA_27383 | Rep 3 | LRRC4C | 2 | | 196.6 | 780.1 | 196.6 | 780.1 | 1.89E+05 | 14000 | 4.9313 | 1 | 4.29E-07 | 8.58E-07 | 6.90E-06 | TRUE |  |  |  |  |  |  |  |
| HGLibA_29454 | Rep 1 | MMAB | 2 | | 68.057 | 689.43 | 68.057 | 689.43 | 2.46E+05 | 7090.9 | 7.379 | 1 | 1.01E-13 | 2.02E-13 | 1.82E-12 | TRUE |  |  |  |  |  |  |  |
| HGLibA_29452 | Rep 1 | MMAB | 2 | | 25.444 | 408.55 | 25.444 | 408.55 | 99801 | 916.31 | 12.656 | 1 | 6.48E-37 | 1.30E-36 | 2.31E-35 | TRUE |  |  |  |  |  |  |  |
| HGLibA_29452 | Rep 3 | MMAB | 2 | | 67.09 | 536.32 | 67.09 | 536.32 | 1.35E+05 | 2089.9 | 10.264 | 1 | 5.51E-25 | 1.10E-24 | 2.58E-23 | TRUE |  |  |  |  |  |  |  |
| HGLibA_29454 | Rep 3 | MMAB | 2 | | 70.409 | 304.72 | 70.409 | 304.72 | 30824 | 2275.4 | 4.9122 | 1 | 4.84E-07 | 9.69E-07 | 7.73E-06 | TRUE |  |  |  |  |  |  |  |
| HGLibA_29938 | Rep 1 | MRPL27 | 2 | | 22.099 | 153.21 | 22.099 | 153.21 | 10325 | 684.81 | 5.01 | 1 | 3.40E-07 | 6.80E-07 | 3.94E-06 | TRUE |  |  |  |  |  |  |  |
| HGLibA_29940 | Rep 1 | MRPL27 | 2 | | 65.328 | 510.69 | 65.328 | 510.69 | 1.21E+05 | 6510.2 | 5.5197 | 1 | 2.15E-08 | 4.29E-08 | 2.76E-07 | TRUE |  |  |  |  |  |  |  |
| HGLibA_29939 | Rep 2 | MRPL27 | 2 | | 119.08 | 1209.4 | 119.08 | 1209.4 | 7.56E+05 | 10497 | 10.642 | 1 | 1.09E-26 | 2.18E-26 | 6.80E-25 | TRUE |  |  |  |  |  |  |  |
| HGLibA_29938 | Rep 2 | MRPL27 | 2 | | 216.86 | 1245.3 | 216.86 | 1245.3 | 6.18E+05 | 31366 | 5.8073 | 1 | 3.57E-09 | 7.14E-09 | 7.26E-08 | TRUE |  |  |  |  |  |  |  |
| HGLibA_30092 | Rep 1 | MRPS33 | 2 | | 50.272 | 331.95 | 50.272 | 331.95 | 47328 | 3769.5 | 4.5878 | 1 | 2.82E-06 | 5.65E-06 | 2.99E-05 | TRUE |  |  |  |  |  |  |  |
| HGLibA_30093 | Rep 1 | MRPS33 | 2 | | 62.422 | 612.82 | 62.422 | 612.82 | 1.92E+05 | 5920.4 | 7.1533 | 1 | 5.35E-13 | 1.07E-12 | 9.28E-12 | TRUE |  |  |  |  |  |  |  |
| HGLibA_30093 | Rep 3 | MRPS33 | 2 | | 125.57 | 901.99 | 125.57 | 901.99 | 3.64E+05 | 6323.4 | 9.7638 | 1 | 8.54E-23 | 1.71E-22 | 3.67E-21 | TRUE |  |  |  |  |  |  |  |
| HGLibA_30092 | Rep 3 | MRPS33 | 2 | | 146.62 | 792.28 | 146.62 | 792.28 | 2.42E+05 | 8321 | 7.0781 | 1 | 7.73E-13 | 1.55E-12 | 2.04E-11 | TRUE |  |  |  |  |  |  |  |
| HGLibA_30151 | Rep 1 | MS4A4A | 2 | | 56.611 | 434.08 | 56.611 | 434.08 | 86917 | 4828.5 | 5.4322 | 1 | 3.51E-08 | 7.02E-08 | 4.45E-07 | TRUE |  |  |  |  |  |  |  |
| HGLibA_30150 | Rep 1 | MS4A4A | 2 | | 33.808 | 714.96 | 33.808 | 714.96 | 3.28E+05 | 1651.3 | 16.762 | 1 | 2.90E-63 | 5.80E-63 | 1.51E-61 | TRUE |  |  |  |  |  |  |  |
| HGLibA_30152 | Rep 3 | MS4A4A | 2 | | 107.32 | 633.83 | 107.32 | 633.83 | 1.63E+05 | 4789.1 | 7.6081 | 1 | 1.48E-14 | 2.96E-14 | 4.37E-13 | TRUE |  |  |  |  |  |  |  |
| HGLibA_30150 | Rep 3 | MS4A4A | 2 | | 62.735 | 316.91 | 62.735 | 316.91 | 37064 | 1857 | 5.8983 | 1 | 1.98E-09 | 3.96E-09 | 4.00E-08 | TRUE |  |  |  |  |  |  |  |
| HGLibA_31078 | Rep 1 | NAIF1 | 2 | | 27.117 | 255.34 | 27.117 | 255.34 | 32760 | 1045.3 | 7.0589 | 1 | 1.05E-12 | 2.10E-12 | 1.78E-11 | TRUE |  |  |  |  |  |  |  |
| HGLibA_31079 | Rep 1 | NAIF1 | 2 | | 16.728 | 306.41 | 16.728 | 306.41 | 58104 | 386.18 | 14.741 | 1 | 2.19E-49 | 4.38E-49 | 9.62E-48 | TRUE |  |  |  |  |  |  |  |
| HGLibA_31079 | Rep 3 | NAIF1 | 2 | | 18.25 | 609.45 | 18.25 | 609.45 | 2.61E+05 | 215.97 | 40.228 | 1 | 0 | 0 | 0 | TRUE |  |  |  |  |  |  |  |
| HGLibA_31077 | Rep 3 | NAIF1 | 2 | | 84.718 | 536.32 | 84.718 | 536.32 | 1.21E+05 | 3153.3 | 8.0421 | 1 | 4.73E-16 | 9.45E-16 | 1.51E-14 | TRUE |  |  |  |  |  |  |  |
| HGLibA_31220 | Rep 1 | NBPF1 | 2 | | 55.203 | 408.55 | 55.203 | 408.55 | 75758 | 4581.3 | 5.2204 | 1 | 1.13E-07 | 2.25E-07 | 1.36E-06 | TRUE |  |  |  |  |  |  |  |
| HGLibA_31221 | Rep 1 | NBPF1 | 2 | | 72.635 | 587.29 | 72.635 | 587.29 | 1.63E+05 | 8123.7 | 5.71 | 1 | 7.15E-09 | 1.43E-08 | 9.55E-08 | TRUE |  |  |  |  |  |  |  |
| HGLibA_31220 | Rep 2 | NBPF1 | 2 | | 524.41 | 4043.2 | 524.41 | 4043.2 | 7.56E+06 | 1.58E+05 | 8.8595 | 1 | 4.43E-19 | 8.86E-19 | 1.89E-17 | TRUE |  |  |  |  |  |  |  |
| HGLibA_31222 | Rep 2 | NBPF1 | 2 | | 1142.7 | 7161.1 | 1142.7 | 7161.1 | 2.14E+07 | 6.57E+05 | 7.4264 | 1 | 6.06E-14 | 1.21E-13 | 1.86E-12 | TRUE |  |  |  |  |  |  |  |
| HGLibA_32116 | Rep 1 | NLRP2 | 2 | | 52.473 | 434.08 | 52.473 | 434.08 | 89849 | 4121.7 | 5.944 | 1 | 1.75E-09 | 3.51E-09 | 2.46E-08 | TRUE |  |  |  |  |  |  |  |
| HGLibA_32115 | Rep 1 | NLRP2 | 2 | | 21.218 | 255.34 | 21.218 | 255.34 | 35750 | 629.73 | 9.3298 | 1 | 6.62E-21 | 1.32E-20 | 1.61E-19 | TRUE |  |  |  |  |  |  |  |
| HGLibA_32114 | Rep 2 | NLRP2 | 2 | | 346.19 | 1891.8 | 346.19 | 1891.8 | 1.39E+06 | 73774 | 5.6905 | 1 | 7.05E-09 | 1.41E-08 | 1.38E-07 | TRUE |  |  |  |  |  |  |  |
| HGLibA_32115 | Rep 2 | NLRP2 | 2 | | 213.71 | 1167 | 213.71 | 1167 | 5.27E+05 | 30537 | 5.455 | 1 | 2.75E-08 | 5.51E-08 | 5.09E-07 | TRUE |  |  |  |  |  |  |  |
| HGLibA_32418 | Rep 2 | NPC1 | 2 | | 155.35 | 1186.2 | 155.35 | 1186.2 | 6.48E+05 | 17054 | 7.894 | 1 | 1.66E-15 | 3.32E-15 | 5.66E-14 | TRUE |  |  |  |  |  |  |  |
| HGLibA_32419 | Rep 2 | NPC1 | 2 | | 14.194 | 107.96 | 14.194 | 107.96 | 5352.5 | 224.39 | 6.2592 | 1 | 2.34E-10 | 4.67E-10 | 5.30E-09 | TRUE |  |  |  |  |  |  |  |
| HGLibA_32418 | Rep 3 | NPC1 | 2 | | 33.597 | 207.21 | 33.597 | 207.21 | 17798 | 621.71 | 6.963 | 1 | 1.83E-12 | 3.66E-12 | 4.69E-11 | TRUE |  |  |  |  |  |  |  |
| HGLibA_32417 | Rep 3 | NPC1 | 2 | | 98.095 | 487.56 | 98.095 | 487.56 | 86823 | 4085.2 | 6.0934 | 1 | 5.89E-10 | 1.18E-09 | 1.25E-08 | TRUE |  |  |  |  |  |  |  |
| HGLibA_33064 | Rep 2 | NUP62 | 2 | | 255.5 | 2093.6 | 255.5 | 2093.6 | 2.08E+06 | 42328 | 8.934 | 1 | 2.30E-19 | 4.60E-19 | 1.00E-17 | TRUE |  |  |  |  |  |  |  |
| HGLibA_33062 | Rep 2 | NUP62 | 2 | | 557.53 | 2638.5 | 557.53 | 2638.5 | 2.46E+06 | 1.76E+05 | 4.9538 | 1 | 4.01E-07 | 8.02E-07 | 6.43E-06 | TRUE |  |  |  |  |  |  |  |
| HGLibA_33064 | Rep 3 | NUP62 | 2 | | 38.782 | 231.59 | 38.782 | 231.59 | 21848 | 798.74 | 6.8222 | 1 | 4.90E-12 | 9.80E-12 | 1.22E-10 | TRUE |  |  |  |  |  |  |  |
| HGLibA_33062 | Rep 3 | NUP62 | 2 | | 123.19 | 609.45 | 123.19 | 609.45 | 1.35E+05 | 6112.3 | 6.2197 | 1 | 2.64E-10 | 5.29E-10 | 5.74E-09 | TRUE |  |  |  |  |  |  |  |
| HGLibA_33504 | Rep 1 | OR10G9 | 2 | | 174.68 | 1404.4 | 174.68 | 1404.4 | 9.29E+05 | 50945 | 5.4482 | 1 | 3.26E-08 | 6.52E-08 | 4.14E-07 | TRUE |  |  |  |  |  |  |  |
| HGLibA_33503 | Rep 1 | OR10G9 | 2 | | 33.632 | 714.96 | 33.632 | 714.96 | 3.28E+05 | 1633.5 | 16.857 | 1 | 5.80E-64 | 1.16E-63 | 3.03E-62 | TRUE |  |  |  |  |  |  |  |
| HGLibA_33502 | Rep 2 | OR10G9 | 2 | | 690.01 | 3531.7 | 690.01 | 3531.7 | 4.64E+06 | 2.61E+05 | 5.5652 | 1 | 1.44E-08 | 2.87E-08 | 2.74E-07 | TRUE |  |  |  |  |  |  |  |
| HGLibA_33504 | Rep 2 | OR10G9 | 2 | | 1611.1 | 7500.4 | 1611.1 | 7500.4 | 1.97E+07 | 1.23E+06 | 5.305 | 1 | 6.08E-08 | 1.22E-07 | 1.08E-06 | TRUE |  |  |  |  |  |  |  |
| HGLibA_35803 | Rep 1 | PDE5A | 2 | | 33.016 | 357.48 | 33.016 | 357.48 | 67613 | 1572 | 8.1835 | 1 | 1.73E-16 | 3.46E-16 | 3.51E-15 | TRUE |  |  |  |  |  |  |  |
| HGLibA_35802 | Rep 1 | PDE5A | 2 | | 14.351 | 204.27 | 14.351 | 204.27 | 24100 | 282.18 | 11.306 | 1 | 7.61E-30 | 1.52E-29 | 2.35E-28 | TRUE |  |  |  |  |  |  |  |
| HGLibA_35803 | Rep 2 | PDE5A | 2 | | 249.98 | 1592.3 | 249.98 | 1592.3 | 1.07E+06 | 40671 | 6.6562 | 1 | 1.57E-11 | 3.15E-11 | 3.98E-10 | TRUE |  |  |  |  |  |  |  |
| HGLibA_35801 | Rep 2 | PDE5A | 2 | | 501.54 | 2912.2 | 501.54 | 2912.2 | 3.40E+06 | 1.45E+05 | 6.3224 | 1 | 1.42E-10 | 2.84E-10 | 3.29E-09 | TRUE |  |  |  |  |  |  |  |
| HGLibA_35843 | Rep 1 | PDGFB | 2 | | 39.795 | 306.41 | 39.795 | 306.41 | 43385 | 2317.2 | 5.5386 | 1 | 1.92E-08 | 3.83E-08 | 2.47E-07 | TRUE |  |  |  |  |  |  |  |
| HGLibA_35845 | Rep 1 | PDGFB | 2 | | 22.539 | 331.95 | 22.539 | 331.95 | 64298 | 713.26 | 11.585 | 1 | 3.06E-31 | 6.11E-31 | 9.75E-30 | TRUE |  |  |  |  |  |  |  |
| HGLibA_35843 | Rep 2 | PDGFB | 2 | | 297.3 | 2028 | 297.3 | 2028 | 1.80E+06 | 55841 | 7.3241 | 1 | 1.34E-13 | 2.68E-13 | 4.00E-12 | TRUE |  |  |  |  |  |  |  |
| HGLibA_35844 | Rep 2 | PDGFB | 2 | | 186.11 | 1263.3 | 186.11 | 1263.3 | 6.95E+05 | 23719 | 6.9946 | 1 | 1.50E-12 | 3.00E-12 | 4.12E-11 | TRUE |  |  |  |  |  |  |  |
| HGLibA_35998 | Rep 1 | PDZRN3 | 2 | | 102.13 | 740.49 | 102.13 | 740.49 | 2.47E+05 | 16563 | 4.9602 | 1 | 4.48E-07 | 8.96E-07 | 5.12E-06 | TRUE |  |  |  |  |  |  |  |
| HGLibA_35997 | Rep 1 | PDZRN3 | 2 | | 10.565 | 178.74 | 10.565 | 178.74 | 19360 | 151.41 | 13.668 | 1 | 9.86E-43 | 1.97E-42 | 3.88E-41 | TRUE |  |  |  |  |  |  |  |
| HGLibA_35997 | Rep 2 | PDZRN3 | 2 | | 51.258 | 3335.1 | 51.258 | 3335.1 | 8.67E+06 | 2264.3 | 69.01 | 1 | 0 | 0 | 0 | TRUE |  |  |  |  |  |  |  |
| HGLibA_35998 | Rep 2 | PDZRN3 | 2 | | 1038.6 | 4796.3 | 1038.6 | 4796.3 | 8.00E+06 | 5.51E+05 | 5.0608 | 1 | 2.27E-07 | 4.54E-07 | 3.76E-06 | TRUE |  |  |  |  |  |  |  |
| HGLibA_36311 | Rep 1 | PHB | 2 | | 61.806 | 434.08 | 61.806 | 434.08 | 83440 | 5799.1 | 4.8886 | 1 | 6.41E-07 | 1.28E-06 | 7.27E-06 | TRUE |  |  |  |  |  |  |  |
| HGLibA_36313 | Rep 1 | PHB | 2 | | 140.25 | 2732.2 | 140.25 | 2732.2 | 4.69E+06 | 32171 | 14.451 | 1 | 1.58E-47 | 3.16E-47 | 6.69E-46 | TRUE |  |  |  |  |  |  |  |
| HGLibA_36311 | Rep 2 | PHB | 2 | | 247.61 | 2263.2 | 247.61 | 2263.2 | 2.54E+06 | 39971 | 10.082 | 1 | 3.73E-24 | 7.46E-24 | 2.08E-22 | TRUE |  |  |  |  |  |  |  |
| HGLibA_36313 | Rep 2 | PHB | 2 | | 1040.9 | 4382.5 | 1040.9 | 4382.5 | 6.25E+06 | 5.54E+05 | 4.4909 | 1 | 3.86E-06 | 7.72E-06 | 5.45E-05 | TRUE |  |  |  |  |  |  |  |
| HGLibA_36507 | Rep 1 | PIAS1 | 2 | | 19.545 | 408.55 | 19.545 | 408.55 | 1.07E+05 | 531.73 | 16.87 | 1 | 4.68E-64 | 9.37E-64 | 2.45E-62 | TRUE |  |  |  |  |  |  |  |
| HGLibA_36506 | Rep 1 | PIAS1 | 2 | | 22.451 | 612.82 | 22.451 | 612.82 | 2.54E+05 | 707.52 | 22.195 | 1 | 2.39E-109 | 4.79E-109 | 1.70E-107 | TRUE |  |  |  |  |  |  |  |
| HGLibA_36506 | Rep 2 | PIAS1 | 2 | | 316.22 | 1686.2 | 316.22 | 1686.2 | 1.09E+06 | 62514 | 5.4792 | 1 | 2.38E-08 | 4.76E-08 | 4.43E-07 | TRUE |  |  |  |  |  |  |  |
| HGLibA_36507 | Rep 2 | PIAS1 | 2 | | 135.64 | 686.29 | 135.64 | 686.29 | 1.74E+05 | 13312 | 4.7726 | 1 | 1.03E-06 | 2.07E-06 | 1.58E-05 | TRUE |  |  |  |  |  |  |  |
| HGLibA_36992 | Rep 1 | PLCD3 | 2 | | 44.549 | 510.69 | 44.549 | 510.69 | 1.41E+05 | 2930.6 | 8.6106 | 1 | 4.57E-18 | 9.15E-18 | 9.97E-17 | TRUE |  |  |  |  |  |  |  |
| HGLibA_36993 | Rep 1 | PLCD3 | 2 | | 12.766 | 229.81 | 12.766 | 229.81 | 32532 | 222.28 | 14.558 | 1 | 3.24E-48 | 6.48E-48 | 1.38E-46 | TRUE |  |  |  |  |  |  |  |
| HGLibA_36993 | Rep 2 | PLCD3 | 2 | | 150.62 | 1994.6 | 150.62 | 1994.6 | 2.25E+06 | 16118 | 14.525 | 1 | 4.78E-48 | 9.57E-48 | 5.68E-46 | TRUE |  |  |  |  |  |  |  |
| HGLibA_36992 | Rep 2 | PLCD3 | 2 | | 409.27 | 4846.4 | 409.27 | 4846.4 | 1.28E+07 | 1.00E+05 | 14.017 | 1 | 6.84E-45 | 1.37E-44 | 7.52E-43 | TRUE |  |  |  |  |  |  |  |
| HGLibA_37216 | Rep 1 | PLP1 | 2 | | 128.37 | 1149 | 128.37 | 1149 | 6.51E+05 | 26726 | 6.2433 | 1 | 2.73E-10 | 5.46E-10 | 4.03E-09 | TRUE |  |  |  |  |  |  |  |
| HGLibA_37215 | Rep 1 | PLP1 | 2 | | 35.305 | 408.55 | 35.305 | 408.55 | 90359 | 1806.8 | 8.7809 | 1 | 1.02E-18 | 2.03E-18 | 2.27E-17 | TRUE |  |  |  |  |  |  |  |
| HGLibA_37215 | Rep 2 | PLP1 | 2 | | 313.86 | 2427.7 | 313.86 | 2427.7 | 2.73E+06 | 61661 | 8.5128 | 1 | 9.46E-18 | 1.89E-17 | 3.72E-16 | TRUE |  |  |  |  |  |  |  |
| HGLibA_37217 | Rep 2 | PLP1 | 2 | | 640.33 | 2966.2 | 640.33 | 2966.2 | 3.07E+06 | 2.27E+05 | 4.8776 | 1 | 5.90E-07 | 1.18E-06 | 9.26E-06 | TRUE |  |  |  |  |  |  |  |
| HGLibA_37449 | Rep 1 | POC5 | 2 | | 150.29 | 1098 | 150.29 | 1098 | 5.44E+05 | 37181 | 4.9148 | 1 | 5.68E-07 | 1.14E-06 | 6.46E-06 | TRUE |  |  |  |  |  |  |  |
| HGLibA_37450 | Rep 1 | POC5 | 2 | | 16.992 | 689.43 | 16.992 | 689.43 | 3.46E+05 | 398.79 | 33.673 | 1 | 9.08E-249 | 1.82E-248 | 9.71E-247 | TRUE |  |  |  |  |  |  |  |
| HGLibA_37451 | Rep 2 | POC5 | 2 | | 586.7 | 2866 | 586.7 | 2866 | 2.97E+06 | 1.94E+05 | 5.1783 | 1 | 1.23E-07 | 2.46E-07 | 2.11E-06 | TRUE |  |  |  |  |  |  |  |
| HGLibA_37450 | Rep 2 | POC5 | 2 | | 239.73 | 1179.8 | 239.73 | 1179.8 | 5.05E+05 | 37674 | 4.8433 | 1 | 7.16E-07 | 1.43E-06 | 1.11E-05 | TRUE |  |  |  |  |  |  |  |
| HGLibA_37632 | Rep 2 | POLR3H | 2 | | 189.26 | 1751.7 | 189.26 | 1751.7 | 1.53E+06 | 24458 | 9.9907 | 1 | 9.44E-24 | 1.89E-23 | 5.17E-22 | TRUE |  |  |  |  |  |  |  |
| HGLibA_37634 | Rep 2 | POLR3H | 2 | | 479.46 | 3299.1 | 479.46 | 3299.1 | 4.77E+06 | 1.34E+05 | 7.706 | 1 | 7.17E-15 | 1.43E-14 | 2.34E-13 | TRUE |  |  |  |  |  |  |  |
| HGLibA_37632 | Rep 3 | POLR3H | 2 | | 35.567 | 450.99 | 35.567 | 450.99 | 1.13E+05 | 686.7 | 15.853 | 1 | 7.34E-57 | 1.47E-56 | 6.19E-55 | TRUE |  |  |  |  |  |  |  |
| HGLibA_37634 | Rep 3 | POLR3H | 2 | | 137.6 | 609.45 | 137.6 | 609.45 | 1.25E+05 | 7435.5 | 5.472 | 1 | 2.36E-08 | 4.71E-08 | 4.33E-07 | TRUE |  |  |  |  |  |  |  |
| HGLibA_37906 | Rep 1 | PPIAL4D | 2 | | 84.081 | 791.56 | 84.081 | 791.56 | 3.15E+05 | 11029 | 6.7367 | 1 | 1.03E-11 | 2.06E-11 | 1.66E-10 | TRUE |  |  |  |  |  |  |  |
| HGLibA_37905 | Rep 1 | PPIAL4D | 2 | | 47.543 | 1098 | 47.543 | 1098 | 7.89E+05 | 3355.6 | 18.133 | 1 | 1.09E-73 | 2.19E-73 | 6.25E-72 | TRUE |  |  |  |  |  |  |  |
| HGLibA_37905 | Rep 2 | PPIAL4D | 2 | | 327.26 | 4408.2 | 327.26 | 4408.2 | 1.10E+07 | 66564 | 15.818 | 1 | 1.31E-56 | 2.62E-56 | 1.82E-54 | TRUE |  |  |  |  |  |  |  |
| HGLibA_37906 | Rep 2 | PPIAL4D | 2 | | 413.22 | 1923.9 | 413.22 | 1923.9 | 1.29E+06 | 1.02E+05 | 4.7305 | 1 | 1.24E-06 | 2.48E-06 | 1.87E-05 | TRUE |  |  |  |  |  |  |  |
| HGLibA_38165 | Rep 1 | PPP2R3C | 2 | | 82.672 | 561.75 | 82.672 | 561.75 | 1.38E+05 | 10646 | 4.6431 | 1 | 2.18E-06 | 4.35E-06 | 2.34E-05 | TRUE |  |  |  |  |  |  |  |
| HGLibA_38163 | Rep 1 | PPP2R3C | 2 | | 34.601 | 280.88 | 34.601 | 280.88 | 37311 | 1732.7 | 5.9164 | 1 | 2.06E-09 | 4.13E-09 | 2.88E-08 | TRUE |  |  |  |  |  |  |  |
| HGLibA_38164 | Rep 2 | PPP2R3C | 2 | | 373 | 2539.5 | 373 | 2539.5 | 2.81E+06 | 84562 | 7.4504 | 1 | 5.17E-14 | 1.03E-13 | 1.59E-12 | TRUE |  |  |  |  |  |  |  |
| HGLibA_38163 | Rep 2 | PPP2R3C | 2 | | 300.45 | 1471.5 | 300.45 | 1471.5 | 7.83E+05 | 56929 | 4.9082 | 1 | 5.13E-07 | 1.03E-06 | 8.12E-06 | TRUE |  |  |  |  |  |  |  |
| HGLibA_38380 | Rep 1 | PRDM15 | 2 | | 47.543 | 459.62 | 47.543 | 459.62 | 1.07E+05 | 3355.6 | 7.1136 | 1 | 7.12E-13 | 1.42E-12 | 1.22E-11 | TRUE |  |  |  |  |  |  |  |
| HGLibA_38379 | Rep 1 | PRDM15 | 2 | | 31.167 | 357.48 | 31.167 | 357.48 | 68970 | 1394.8 | 8.7372 | 1 | 1.50E-18 | 2.99E-18 | 3.32E-17 | TRUE |  |  |  |  |  |  |  |
| HGLibA_38380 | Rep 2 | PRDM15 | 2 | | 286.26 | 2734.9 | 286.26 | 2734.9 | 3.78E+06 | 52107 | 10.727 | 1 | 4.25E-27 | 8.50E-27 | 2.71E-25 | TRUE |  |  |  |  |  |  |  |
| HGLibA_38381 | Rep 2 | PRDM15 | 2 | | 93.841 | 890.64 | 93.841 | 890.64 | 4.00E+05 | 6800.3 | 9.6624 | 1 | 2.50E-22 | 4.99E-22 | 1.27E-20 | TRUE |  |  |  |  |  |  |  |
| HGLibA_38449 | Rep 2 | PRF1 | 2 | | 40.218 | 758.26 | 40.218 | 758.26 | 3.59E+05 | 1458.8 | 18.8 | 1 | 4.44E-79 | 8.87E-79 | 8.90E-77 | TRUE |  |  |  |  |  |  |  |
| HGLibA_38450 | Rep 2 | PRF1 | 2 | | 730.23 | 3439.2 | 730.23 | 3439.2 | 4.17E+06 | 2.89E+05 | 5.0371 | 1 | 2.59E-07 | 5.18E-07 | 4.25E-06 | TRUE |  |  |  |  |  |  |  |
| HGLibA_38450 | Rep 3 | PRF1 | 2 | | 90.94 | 767.91 | 90.94 | 767.91 | 2.84E+05 | 3573.6 | 11.324 | 1 | 5.31E-30 | 1.06E-29 | 2.88E-28 | TRUE |  |  |  |  |  |  |  |
| HGLibA_38448 | Rep 3 | PRF1 | 2 | | 88.866 | 524.13 | 88.866 | 524.13 | 1.11E+05 | 3430.9 | 7.4309 | 1 | 5.76E-14 | 1.15E-13 | 1.64E-12 | TRUE |  |  |  |  |  |  |  |
| HGLibA_38697 | Rep 2 | PROSER2 | 2 | | 671.87 | 4851.6 | 671.87 | 4851.6 | 1.06E+07 | 2.48E+05 | 8.3878 | 1 | 2.72E-17 | 5.44E-17 | 1.04E-15 | TRUE |  |  |  |  |  |  |  |
| HGLibA_38698 | Rep 2 | PROSER2 | 2 | | 537.02 | 2770.9 | 537.02 | 2770.9 | 2.87E+06 | 1.65E+05 | 5.5032 | 1 | 2.06E-08 | 4.11E-08 | 3.85E-07 | TRUE |  |  |  |  |  |  |  |
| HGLibA_38697 | Rep 3 | PROSER2 | 2 | | 199.82 | 3924.9 | 199.82 | 3924.9 | 9.71E+06 | 14409 | 31.032 | 1 | 1.05E-211 | 2.10E-211 | 1.58E-209 | TRUE |  |  |  |  |  |  |  |
| HGLibA_38699 | Rep 3 | PROSER2 | 2 | | 148.28 | 780.1 | 148.28 | 780.1 | 2.30E+05 | 8488.7 | 6.8575 | 1 | 3.70E-12 | 7.40E-12 | 9.27E-11 | TRUE |  |  |  |  |  |  |  |
| HGLibA_38851 | Rep 1 | PRR7 | 2 | | 117.54 | 944.77 | 117.54 | 944.77 | 4.20E+05 | 22224 | 5.549 | 1 | 1.83E-08 | 3.66E-08 | 2.36E-07 | TRUE |  |  |  |  |  |  |  |
| HGLibA_38852 | Rep 1 | PRR7 | 2 | | 25.18 | 587.29 | 25.18 | 587.29 | 2.26E+05 | 896.75 | 18.771 | 1 | 8.16E-79 | 1.63E-78 | 4.82E-77 | TRUE |  |  |  |  |  |  |  |
| HGLibA_38851 | Rep 2 | PRR7 | 2 | | 837.47 | 3706.5 | 837.47 | 3706.5 | 4.64E+06 | 3.72E+05 | 4.7056 | 1 | 1.38E-06 | 2.77E-06 | 2.07E-05 | TRUE |  |  |  |  |  |  |  |
| HGLibA_38852 | Rep 2 | PRR7 | 2 | | 313.07 | 1420.1 | 313.07 | 1420.1 | 6.93E+05 | 61378 | 4.4686 | 1 | 4.39E-06 | 8.78E-06 | 6.15E-05 | TRUE |  |  |  |  |  |  |  |
| HGLibA_39215 | Rep 1 | PSMG4 | 2 | | 44.197 | 331.95 | 44.197 | 331.95 | 50349 | 2882.6 | 5.3595 | 1 | 5.25E-08 | 1.05E-07 | 6.53E-07 | TRUE |  |  |  |  |  |  |  |
| HGLibA_39213 | Rep 1 | PSMG4 | 2 | | 26.237 | 408.55 | 26.237 | 408.55 | 98960 | 976.32 | 12.236 | 1 | 1.26E-34 | 2.51E-34 | 4.29E-33 | TRUE |  |  |  |  |  |  |  |
| HGLibA_39213 | Rep 2 | PSMG4 | 2 | | 210.55 | 2127 | 210.55 | 2127 | 2.34E+06 | 29718 | 11.117 | 1 | 5.84E-29 | 1.17E-28 | 3.99E-27 | TRUE |  |  |  |  |  |  |  |
| HGLibA_39215 | Rep 2 | PSMG4 | 2 | | 410.06 | 2395.6 | 410.06 | 2395.6 | 2.31E+06 | 1.01E+05 | 6.2611 | 1 | 2.12E-10 | 4.24E-10 | 4.84E-09 | TRUE |  |  |  |  |  |  |  |
| HGLibA_39297 | Rep 1 | PTER | 2 | | 41.556 | 306.41 | 41.556 | 306.41 | 42538 | 2535.6 | 5.2598 | 1 | 9.07E-08 | 1.81E-07 | 1.11E-06 | TRUE |  |  |  |  |  |  |  |
| HGLibA_39299 | Rep 1 | PTER | 2 | | 25.268 | 434.08 | 25.268 | 434.08 | 1.15E+05 | 903.24 | 13.603 | 1 | 2.41E-42 | 4.83E-42 | 9.44E-41 | TRUE |  |  |  |  |  |  |  |
| HGLibA_39299 | Rep 2 | PTER | 2 | | 629.29 | 3892.8 | 629.29 | 3892.8 | 6.29E+06 | 2.20E+05 | 6.9538 | 1 | 1.95E-12 | 3.91E-12 | 5.31E-11 | TRUE |  |  |  |  |  |  |  |
| HGLibA_39298 | Rep 2 | PTER | 2 | | 826.43 | 4626.7 | 826.43 | 4626.7 | 8.41E+06 | 3.63E+05 | 6.3092 | 1 | 1.53E-10 | 3.07E-10 | 3.54E-09 | TRUE |  |  |  |  |  |  |  |
| HGLibA_39679 | Rep 1 | PYCRL | 2 | | 75.717 | 485.15 | 75.717 | 485.15 | 99563 | 8860.3 | 4.3497 | 1 | 8.63E-06 | 1.73E-05 | 8.73E-05 | TRUE |  |  |  |  |  |  |  |
| HGLibA_39680 | Rep 1 | PYCRL | 2 | | 21.13 | 229.81 | 21.13 | 229.81 | 27979 | 624.35 | 8.3514 | 1 | 4.21E-17 | 8.42E-17 | 8.80E-16 | TRUE |  |  |  |  |  |  |  |
| HGLibA_39679 | Rep 3 | PYCRL | 2 | | 105.04 | 767.91 | 105.04 | 767.91 | 2.66E+05 | 4610.6 | 9.7622 | 1 | 8.71E-23 | 1.74E-22 | 3.74E-21 | TRUE |  |  |  |  |  |  |  |
| HGLibA_39680 | Rep 3 | PYCRL | 2 | | 17.732 | 97.512 | 17.732 | 97.512 | 3694.5 | 205.53 | 5.5648 | 1 | 1.47E-08 | 2.94E-08 | 2.75E-07 | TRUE |  |  |  |  |  |  |  |
| HGLibA_40390 | Rep 1 | RASSF6 | 2 | | 85.753 | 638.36 | 85.753 | 638.36 | 1.85E+05 | 11493 | 5.1547 | 1 | 1.61E-07 | 3.22E-07 | 1.92E-06 | TRUE |  |  |  |  |  |  |  |
| HGLibA_40388 | Rep 1 | RASSF6 | 2 | | 17.168 | 204.27 | 17.168 | 204.27 | 22791 | 407.32 | 9.2709 | 1 | 1.15E-20 | 2.30E-20 | 2.75E-19 | TRUE |  |  |  |  |  |  |  |
| HGLibA_40388 | Rep 2 | RASSF6 | 2 | | 175.07 | 1061.6 | 175.07 | 1061.6 | 4.63E+05 | 21212 | 6.0868 | 1 | 6.50E-10 | 1.30E-09 | 1.41E-08 | TRUE |  |  |  |  |  |  |  |
| HGLibA_40390 | Rep 2 | RASSF6 | 2 | | 334.36 | 1664.3 | 334.36 | 1664.3 | 1.01E+06 | 69228 | 5.0547 | 1 | 2.40E-07 | 4.80E-07 | 3.96E-06 | TRUE |  |  |  |  |  |  |  |
| HGLibA_41336 | Rep 1 | RIT1 | 2 | | 110.76 | 740.49 | 110.76 | 740.49 | 2.37E+05 | 19626 | 4.4951 | 1 | 4.43E-06 | 8.85E-06 | 4.61E-05 | TRUE |  |  |  |  |  |  |  |
| HGLibA_41338 | Rep 1 | RIT1 | 2 | | 34.16 | 459.62 | 34.16 | 459.62 | 1.20E+05 | 1687.2 | 10.358 | 1 | 2.42E-25 | 4.84E-25 | 6.66E-24 | TRUE |  |  |  |  |  |  |  |
| HGLibA_41337 | Rep 3 | RIT1 | 2 | | 87.414 | 475.37 | 87.414 | 475.37 | 87269 | 3332.6 | 6.7204 | 1 | 9.69E-12 | 1.94E-11 | 2.36E-10 | TRUE |  |  |  |  |  |  |  |
| HGLibA_41336 | Rep 3 | RIT1 | 2 | | 209.57 | 755.72 | 209.57 | 755.72 | 1.63E+05 | 15681 | 4.3614 | 1 | 6.78E-06 | 1.36E-05 | 9.35E-05 | TRUE |  |  |  |  |  |  |  |
| HGLibA_41762 | Rep 1 | ROPN1B | 2 | | 76.509 | 791.56 | 76.509 | 791.56 | 3.26E+05 | 9055.1 | 7.5143 | 1 | 3.62E-14 | 7.25E-14 | 6.64E-13 | TRUE |  |  |  |  |  |  |  |
| HGLibA_41764 | Rep 1 | ROPN1B | 2 | | 44.021 | 510.69 | 44.021 | 510.69 | 1.41E+05 | 2858.7 | 8.7281 | 1 | 1.63E-18 | 3.26E-18 | 3.61E-17 | TRUE |  |  |  |  |  |  |  |
| HGLibA_41764 | Rep 3 | ROPN1B | 2 | | 74.245 | 402.24 | 74.245 | 402.24 | 62341 | 2498.5 | 6.5619 | 1 | 2.85E-11 | 5.71E-11 | 6.69E-10 | TRUE |  |  |  |  |  |  |  |
| HGLibA_41763 | Rep 3 | ROPN1B | 2 | | 50.084 | 231.59 | 50.084 | 231.59 | 18665 | 1250.1 | 5.1335 | 1 | 1.54E-07 | 3.08E-07 | 2.61E-06 | TRUE |  |  |  |  |  |  |  |
| HGLibA_42220 | Rep 2 | RRAD | 2 | | 89.11 | 1935.5 | 89.11 | 1935.5 | 2.42E+06 | 6188.7 | 23.471 | 1 | 4.67E-122 | 9.35E-122 | 1.45E-119 | TRUE |  |  |  |  |  |  |  |
| HGLibA_42221 | Rep 2 | RRAD | 2 | | 167.18 | 1096.3 | 167.18 | 1096.3 | 5.14E+05 | 19499 | 6.6535 | 1 | 1.62E-11 | 3.24E-11 | 4.08E-10 | TRUE |  |  |  |  |  |  |  |
| HGLibA_42221 | Rep 3 | RRAD | 2 | | 76.63 | 682.58 | 76.63 | 682.58 | 2.29E+05 | 2641.7 | 11.79 | 1 | 2.37E-32 | 4.74E-32 | 1.36E-30 | TRUE |  |  |  |  |  |  |  |
| HGLibA_42222 | Rep 3 | RRAD | 2 | | 85.652 | 463.18 | 85.652 | 463.18 | 82574 | 3214.9 | 6.6584 | 1 | 1.48E-11 | 2.96E-11 | 3.56E-10 | TRUE |  |  |  |  |  |  |  |
| HGLibA_42470 | Rep 1 | RUSC2 | 2 | | 80.735 | 1021.4 | 80.735 | 1021.4 | 5.82E+05 | 10132 | 9.345 | 1 | 5.83E-21 | 1.17E-20 | 1.42E-19 | TRUE |  |  |  |  |  |  |  |
| HGLibA_42469 | Rep 1 | RUSC2 | 2 | | 13.471 | 357.48 | 13.471 | 357.48 | 86044 | 247.98 | 21.846 | 1 | 5.32E-106 | 1.06E-105 | 3.73E-104 | TRUE |  |  |  |  |  |  |  |
| HGLibA_42470 | Rep 3 | RUSC2 | 2 | | 183.33 | 950.74 | 183.33 | 950.74 | 3.39E+05 | 12367 | 6.9007 | 1 | 2.72E-12 | 5.44E-12 | 6.87E-11 | TRUE |  |  |  |  |  |  |  |
| HGLibA_42469 | Rep 3 | RUSC2 | 2 | | 17.421 | 109.7 | 17.421 | 109.7 | 5041.9 | 199.38 | 6.5354 | 1 | 3.56E-11 | 7.12E-11 | 8.30E-10 | TRUE |  |  |  |  |  |  |  |
| HGLibA_42564 | Rep 1 | S100A6 | 2 | | 22.979 | 178.74 | 22.979 | 178.74 | 14827 | 742.31 | 5.717 | 1 | 6.77E-09 | 1.35E-08 | 9.05E-08 | TRUE |  |  |  |  |  |  |  |
| HGLibA_42563 | Rep 1 | S100A6 | 2 | | 33.544 | 510.69 | 33.544 | 510.69 | 1.54E+05 | 1624.7 | 11.838 | 1 | 1.56E-32 | 3.13E-32 | 5.19E-31 | TRUE |  |  |  |  |  |  |  |
| HGLibA_42562 | Rep 2 | S100A6 | 2 | | 287.83 | 2066.6 | 287.83 | 2066.6 | 1.91E+06 | 52633 | 7.7533 | 1 | 5.00E-15 | 1.00E-14 | 1.65E-13 | TRUE |  |  |  |  |  |  |  |
| HGLibA_42564 | Rep 2 | S100A6 | 2 | | 156.14 | 888.07 | 156.14 | 888.07 | 3.13E+05 | 17212 | 5.5789 | 1 | 1.37E-08 | 2.74E-08 | 2.62E-07 | TRUE |  |  |  |  |  |  |  |
| HGLibA_43048 | Rep 1 | SCRN1 | 2 | | 18.313 | 306.41 | 18.313 | 306.41 | 56756 | 465.05 | 13.36 | 1 | 6.50E-41 | 1.30E-40 | 2.50E-39 | TRUE |  |  |  |  |  |  |  |
| HGLibA_43050 | Rep 1 | SCRN1 | 2 | | 16.112 | 331.95 | 16.112 | 331.95 | 70181 | 357.58 | 16.702 | 1 | 7.86E-63 | 1.57E-62 | 4.08E-61 | TRUE |  |  |  |  |  |  |  |
| HGLibA_43048 | Rep 2 | SCRN1 | 2 | | 195.57 | 2589.7 | 195.57 | 2589.7 | 3.79E+06 | 25968 | 14.857 | 1 | 3.55E-50 | 7.11E-50 | 4.47E-48 | TRUE |  |  |  |  |  |  |  |
| HGLibA_43050 | Rep 2 | SCRN1 | 2 | | 152.2 | 1025.6 | 152.2 | 1025.6 | 4.56E+05 | 16427 | 6.8144 | 1 | 5.36E-12 | 1.07E-11 | 1.41E-10 | TRUE |  |  |  |  |  |  |  |
| HGLibA_44017 | Rep 1 | SHD | 2 | | 134.35 | 1251.2 | 134.35 | 1251.2 | 7.83E+05 | 29403 | 6.5132 | 1 | 4.70E-11 | 9.39E-11 | 7.31E-10 | TRUE |  |  |  |  |  |  |  |
| HGLibA_44019 | Rep 1 | SHD | 2 | | 14.351 | 306.41 | 14.351 | 306.41 | 60294 | 282.18 | 17.386 | 1 | 6.51E-68 | 1.30E-67 | 3.50E-66 | TRUE |  |  |  |  |  |  |  |
| HGLibA_44018 | Rep 3 | SHD | 2 | | 100.27 | 597.26 | 100.27 | 597.26 | 1.45E+05 | 4246.9 | 7.6262 | 1 | 1.29E-14 | 2.58E-14 | 3.81E-13 | TRUE |  |  |  |  |  |  |  |
| HGLibA_44017 | Rep 3 | SHD | 2 | | 189.04 | 938.55 | 189.04 | 938.55 | 3.22E+05 | 13058 | 6.5591 | 1 | 2.85E-11 | 5.69E-11 | 6.68E-10 | TRUE |  |  |  |  |  |  |  |
| HGLibA_44052 | Rep 1 | SHISA8 | 2 | | 88.747 | 689.43 | 88.747 | 689.43 | 2.21E+05 | 12348 | 5.4057 | 1 | 4.10E-08 | 8.19E-08 | 5.16E-07 | TRUE |  |  |  |  |  |  |  |
| HGLibA_44050 | Rep 1 | SHISA8 | 2 | | 31.519 | 255.34 | 31.519 | 255.34 | 30807 | 1427.7 | 5.9236 | 1 | 1.97E-09 | 3.95E-09 | 2.76E-08 | TRUE |  |  |  |  |  |  |  |
| HGLibA_44052 | Rep 2 | SHISA8 | 2 | | 755.46 | 6066.1 | 755.46 | 6066.1 | 1.73E+07 | 3.08E+05 | 9.5723 | 1 | 5.72E-22 | 1.14E-21 | 2.86E-20 | TRUE |  |  |  |  |  |  |  |
| HGLibA_44050 | Rep 2 | SHISA8 | 2 | | 320.16 | 1728.6 | 320.16 | 1728.6 | 1.15E+06 | 63947 | 5.5696 | 1 | 1.42E-08 | 2.85E-08 | 2.71E-07 | TRUE |  |  |  |  |  |  |  |
| HGLibA_44248 | Rep 2 | SIX3 | 2 | | 279.95 | 2952.1 | 279.95 | 2952.1 | 4.57E+06 | 50026 | 11.947 | 1 | 3.76E-33 | 7.52E-33 | 2.98E-31 | TRUE |  |  |  |  |  |  |  |
| HGLibA_44250 | Rep 2 | SIX3 | 2 | | 145.89 | 1421.4 | 145.89 | 1421.4 | 1.03E+06 | 15205 | 10.344 | 1 | 2.52E-25 | 5.05E-25 | 1.48E-23 | TRUE |  |  |  |  |  |  |  |
| HGLibA_44248 | Rep 3 | SIX3 | 2 | | 42.204 | 487.56 | 42.204 | 487.56 | 1.29E+05 | 926.06 | 14.635 | 1 | 9.18E-49 | 1.84E-48 | 6.88E-47 | TRUE |  |  |  |  |  |  |  |
| HGLibA_44250 | Rep 3 | SIX3 | 2 | | 25.198 | 219.4 | 25.198 | 219.4 | 23443 | 377 | 10.002 | 1 | 8.27E-24 | 1.65E-23 | 3.72E-22 | TRUE |  |  |  |  |  |  |  |
| HGLibA_44568 | Rep 1 | SLC22A2 | 2 | | 14.791 | 204.27 | 14.791 | 204.27 | 23885 | 300.15 | 10.937 | 1 | 4.77E-28 | 9.55E-28 | 1.41E-26 | TRUE |  |  |  |  |  |  |  |
| HGLibA_44567 | Rep 1 | SLC22A2 | 2 | | 14.527 | 204.27 | 14.527 | 204.27 | 24014 | 289.3 | 11.156 | 1 | 4.17E-29 | 8.35E-29 | 1.27E-27 | TRUE |  |  |  |  |  |  |  |
| HGLibA_44566 | Rep 2 | SLC22A2 | 2 | | 78.858 | 652.88 | 78.858 | 652.88 | 2.03E+05 | 4954 | 8.1555 | 1 | 2.00E-16 | 4.00E-16 | 7.24E-15 | TRUE |  |  |  |  |  |  |  |
| HGLibA_44567 | Rep 2 | SLC22A2 | 2 | | 130.12 | 967.75 | 130.12 | 967.75 | 4.26E+05 | 12340 | 7.5404 | 1 | 2.66E-14 | 5.33E-14 | 8.38E-13 | TRUE |  |  |  |  |  |  |  |
| HGLibA_44701 | Rep 2 | SLC25A30 | 2 | | 345.4 | 2080.7 | 345.4 | 2080.7 | 1.77E+06 | 73467 | 6.4023 | 1 | 8.52E-11 | 1.70E-10 | 2.02E-09 | TRUE |  |  |  |  |  |  |  |
| HGLibA_44702 | Rep 2 | SLC25A30 | 2 | | 552.01 | 3186 | 552.01 | 3186 | 4.06E+06 | 1.73E+05 | 6.3276 | 1 | 1.37E-10 | 2.74E-10 | 3.18E-09 | TRUE |  |  |  |  |  |  |  |
| HGLibA_44702 | Rep 3 | SLC25A30 | 2 | | 113.86 | 585.07 | 113.86 | 585.07 | 1.28E+05 | 5316.8 | 6.4624 | 1 | 5.48E-11 | 1.10E-10 | 1.26E-09 | TRUE |  |  |  |  |  |  |  |
| HGLibA_44703 | Rep 3 | SLC25A30 | 2 | | 114.69 | 536.32 | 114.69 | 536.32 | 1.01E+05 | 5385.5 | 5.7454 | 1 | 4.87E-09 | 9.75E-09 | 9.52E-08 | TRUE |  |  |  |  |  |  |  |
| HGLibA_45442 | Rep 1 | SLC9C2 | 2 | | 18.665 | 408.55 | 18.665 | 408.55 | 1.08E+05 | 483.62 | 17.729 | 1 | 1.57E-70 | 3.13E-70 | 8.58E-69 | TRUE |  |  |  |  |  |  |  |
| HGLibA_45441 | Rep 1 | SLC9C2 | 2 | | 17.344 | 383.01 | 17.344 | 383.01 | 94955 | 415.94 | 17.93 | 1 | 4.31E-72 | 8.61E-72 | 2.43E-70 | TRUE |  |  |  |  |  |  |  |
| HGLibA_45441 | Rep 3 | SLC9C2 | 2 | | 40.856 | 463.18 | 40.856 | 463.18 | 1.15E+05 | 874.92 | 14.278 | 1 | 1.64E-46 | 3.28E-46 | 1.19E-44 | TRUE |  |  |  |  |  |  |  |
| HGLibA_45443 | Rep 3 | SLC9C2 | 2 | | 14.828 | 97.512 | 14.828 | 97.512 | 4073.3 | 151.28 | 6.7224 | 1 | 1.01E-11 | 2.02E-11 | 2.45E-10 | TRUE |  |  |  |  |  |  |  |
| HGLibA_45640 | Rep 2 | SMC1A | 2 | | 265.75 | 2396.9 | 265.75 | 2396.9 | 2.84E+06 | 45485 | 9.9925 | 1 | 9.20E-24 | 1.84E-23 | 5.05E-22 | TRUE |  |  |  |  |  |  |  |
| HGLibA_45642 | Rep 2 | SMC1A | 2 | | 812.24 | 3536.8 | 812.24 | 3536.8 | 4.17E+06 | 3.51E+05 | 4.5957 | 1 | 2.36E-06 | 4.71E-06 | 3.43E-05 | TRUE |  |  |  |  |  |  |  |
| HGLibA_45641 | Rep 3 | SMC1A | 2 | | 238.29 | 1133.6 | 238.29 | 1133.6 | 4.56E+05 | 19699 | 6.3787 | 1 | 9.35E-11 | 1.87E-10 | 2.10E-09 | TRUE |  |  |  |  |  |  |  |
| HGLibA_45642 | Rep 3 | SMC1A | 2 | | 130.34 | 511.94 | 130.34 | 511.94 | 80694 | 6754.9 | 4.6429 | 1 | 1.82E-06 | 3.64E-06 | 2.71E-05 | TRUE |  |  |  |  |  |  |  |
| HGLibA_45711 | Rep 1 | SMG8 | 2 | | 45.958 | 306.41 | 45.958 | 306.41 | 40523 | 3126.9 | 4.6577 | 1 | 2.01E-06 | 4.02E-06 | 2.17E-05 | TRUE |  |  |  |  |  |  |  |
| HGLibA_45710 | Rep 1 | SMG8 | 2 | | 77.742 | 689.43 | 77.742 | 689.43 | 2.33E+05 | 9362.6 | 6.3216 | 1 | 1.64E-10 | 3.28E-10 | 2.46E-09 | TRUE |  |  |  |  |  |  |  |
| HGLibA_45710 | Rep 3 | SMG8 | 2 | | 184.37 | 3156.9 | 184.37 | 3156.9 | 6.07E+06 | 12491 | 26.597 | 1 | 3.90E-156 | 7.79E-156 | 5.48E-154 | TRUE |  |  |  |  |  |  |  |
| HGLibA_45711 | Rep 3 | SMG8 | 2 | | 57.758 | 719.15 | 57.758 | 719.15 | 2.87E+05 | 1605.8 | 16.505 | 1 | 1.82E-61 | 3.64E-61 | 1.59E-59 | TRUE |  |  |  |  |  |  |  |
| HGLibA_45837 | Rep 1 | SMTNL2 | 2 | | 71.49 | 740.49 | 71.49 | 740.49 | 2.86E+05 | 7858.6 | 7.5467 | 1 | 2.83E-14 | 5.65E-14 | 5.20E-13 | TRUE |  |  |  |  |  |  |  |
| HGLibA_45835 | Rep 1 | SMTNL2 | 2 | | 17.961 | 306.41 | 17.961 | 306.41 | 57049 | 446.86 | 13.645 | 1 | 1.34E-42 | 2.68E-42 | 5.26E-41 | TRUE |  |  |  |  |  |  |  |
| HGLibA_45836 | Rep 2 | SMTNL2 | 2 | | 751.52 | 3689.8 | 751.52 | 3689.8 | 4.93E+06 | 3.05E+05 | 5.3216 | 1 | 5.63E-08 | 1.13E-07 | 1.01E-06 | TRUE |  |  |  |  |  |  |  |
| HGLibA_45837 | Rep 2 | SMTNL2 | 2 | | 406.91 | 1971.5 | 406.91 | 1971.5 | 1.40E+06 | 99157 | 4.9686 | 1 | 3.74E-07 | 7.48E-07 | 6.02E-06 | TRUE |  |  |  |  |  |  |  |
| HGLibA_46150 | Rep 1 | SOD3 | 2 | | 19.193 | 127.67 | 19.193 | 127.67 | 7024.5 | 512.2 | 4.7932 | 1 | 1.02E-06 | 2.05E-06 | 1.14E-05 | TRUE |  |  |  |  |  |  |  |
| HGLibA_46151 | Rep 1 | SOD3 | 2 | | 50.272 | 383.01 | 50.272 | 383.01 | 67472 | 3769.5 | 5.4196 | 1 | 3.76E-08 | 7.53E-08 | 4.75E-07 | TRUE |  |  |  |  |  |  |  |
| HGLibA_46150 | Rep 3 | SOD3 | 2 | | 41.581 | 390.05 | 41.581 | 390.05 | 76341 | 902.3 | 11.601 | 1 | 2.23E-31 | 4.46E-31 | 1.25E-29 | TRUE |  |  |  |  |  |  |  |
| HGLibA_46151 | Rep 3 | SOD3 | 2 | | 79.119 | 316.91 | 79.119 | 316.91 | 31416 | 2794.9 | 4.498 | 1 | 3.68E-06 | 7.35E-06 | 5.27E-05 | TRUE |  |  |  |  |  |  |  |
| HGLibA_46783 | Rep 2 | SPPL3 | 2 | | 285.47 | 2213.1 | 285.47 | 2213.1 | 2.27E+06 | 51844 | 8.4659 | 1 | 1.42E-17 | 2.84E-17 | 5.52E-16 | TRUE |  |  |  |  |  |  |  |
| HGLibA_46784 | Rep 2 | SPPL3 | 2 | | 547.28 | 3764.3 | 547.28 | 3764.3 | 6.21E+06 | 1.71E+05 | 7.7894 | 1 | 3.71E-15 | 7.42E-15 | 1.23E-13 | TRUE |  |  |  |  |  |  |  |
| HGLibA_46783 | Rep 3 | SPPL3 | 2 | | 64.083 | 572.88 | 64.083 | 572.88 | 1.62E+05 | 1927.8 | 11.588 | 1 | 2.55E-31 | 5.10E-31 | 1.43E-29 | TRUE |  |  |  |  |  |  |  |
| HGLibA_46784 | Rep 3 | SPPL3 | 2 | | 70.201 | 572.88 | 70.201 | 572.88 | 1.56E+05 | 2263.6 | 10.566 | 1 | 2.32E-26 | 4.63E-26 | 1.13E-24 | TRUE |  |  |  |  |  |  |  |
| HGLibA_47029 | Rep 1 | SRPRB | 2 | | 46.751 | 408.55 | 46.751 | 408.55 | 81427 | 3240.2 | 6.3559 | 1 | 1.30E-10 | 2.61E-10 | 1.97E-09 | TRUE |  |  |  |  |  |  |  |
| HGLibA_47031 | Rep 1 | SRPRB | 2 | | 17.52 | 357.48 | 17.52 | 357.48 | 81211 | 424.66 | 16.497 | 1 | 2.40E-61 | 4.79E-61 | 1.21E-59 | TRUE |  |  |  |  |  |  |  |
| HGLibA_47030 | Rep 3 | SRPRB | 2 | | 100.48 | 816.66 | 100.48 | 816.66 | 3.16E+05 | 4262.4 | 10.97 | 1 | 2.85E-28 | 5.70E-28 | 1.46E-26 | TRUE |  |  |  |  |  |  |  |
| HGLibA_47029 | Rep 3 | SRPRB | 2 | | 82.437 | 609.45 | 82.437 | 609.45 | 1.69E+05 | 3005 | 9.6139 | 1 | 3.74E-22 | 7.48E-22 | 1.57E-20 | TRUE |  |  |  |  |  |  |  |
| HGLibA_47055 | Rep 1 | SRRM5 | 2 | | 18.137 | 127.67 | 18.137 | 127.67 | 7222.3 | 455.91 | 5.1299 | 1 | 1.81E-07 | 3.61E-07 | 2.15E-06 | TRUE |  |  |  |  |  |  |  |
| HGLibA_47054 | Rep 1 | SRRM5 | 2 | | 45.43 | 357.48 | 45.43 | 357.48 | 59628 | 3052.5 | 5.648 | 1 | 1.02E-08 | 2.04E-08 | 1.35E-07 | TRUE |  |  |  |  |  |  |  |
| HGLibA_47055 | Rep 2 | SRRM5 | 2 | | 165.6 | 1819.8 | 165.6 | 1819.8 | 1.76E+06 | 19165 | 11.949 | 1 | 3.70E-33 | 7.40E-33 | 2.93E-31 | TRUE |  |  |  |  |  |  |  |
| HGLibA_47054 | Rep 2 | SRRM5 | 2 | | 469.99 | 3258 | 469.99 | 3258 | 4.67E+06 | 1.29E+05 | 7.7597 | 1 | 4.71E-15 | 9.41E-15 | 1.55E-13 | TRUE |  |  |  |  |  |  |  |
| HGLibA_47164 | Rep 1 | SSRP1 | 2 | | 13.382 | 153.21 | 13.382 | 153.21 | 12653 | 244.68 | 8.9387 | 1 | 2.45E-19 | 4.90E-19 | 5.63E-18 | TRUE |  |  |  |  |  |  |  |
| HGLibA_47165 | Rep 1 | SSRP1 | 2 | | 48.423 | 587.29 | 48.423 | 587.29 | 1.90E+05 | 3486.4 | 9.1262 | 1 | 4.47E-20 | 8.93E-20 | 1.05E-18 | TRUE |  |  |  |  |  |  |  |
| HGLibA_47164 | Rep 2 | SSRP1 | 2 | | 258.65 | 1976.6 | 258.65 | 1976.6 | 1.80E+06 | 43289 | 8.2571 | 1 | 8.36E-17 | 1.67E-16 | 3.10E-15 | TRUE |  |  |  |  |  |  |  |
| HGLibA_47166 | Rep 2 | SSRP1 | 2 | | 231.05 | 1298 | 231.05 | 1298 | 6.63E+05 | 35220 | 5.6855 | 1 | 7.32E-09 | 1.46E-08 | 1.44E-07 | TRUE |  |  |  |  |  |  |  |
| HGLibA_47969 | Rep 2 | SYNDIG1 | 2 | | 154.56 | 929.19 | 154.56 | 929.19 | 3.53E+05 | 16896 | 5.9594 | 1 | 1.43E-09 | 2.87E-09 | 3.02E-08 | TRUE |  |  |  |  |  |  |  |
| HGLibA_47971 | Rep 2 | SYNDIG1 | 2 | | 245.25 | 1319.9 | 245.25 | 1319.9 | 6.69E+05 | 39275 | 5.4226 | 1 | 3.29E-08 | 6.59E-08 | 6.04E-07 | TRUE |  |  |  |  |  |  |  |
| HGLibA_47969 | Rep 3 | SYNDIG1 | 2 | | 59.935 | 341.29 | 59.935 | 341.29 | 46205 | 1713.7 | 6.7965 | 1 | 5.79E-12 | 1.16E-11 | 1.43E-10 | TRUE |  |  |  |  |  |  |  |
| HGLibA_47971 | Rep 3 | SYNDIG1 | 2 | | 88.866 | 414.43 | 88.866 | 414.43 | 60124 | 3430.9 | 5.5581 | 1 | 1.46E-08 | 2.92E-08 | 2.73E-07 | TRUE |  |  |  |  |  |  |  |
| HGLibA_48468 | Rep 2 | TBC1D10B | 2 | | 346.98 | 2689.9 | 346.98 | 2689.9 | 3.35E+06 | 74082 | 8.608 | 1 | 4.14E-18 | 8.27E-18 | 1.66E-16 | TRUE |  |  |  |  |  |  |  |
| HGLibA_48467 | Rep 2 | TBC1D10B | 2 | | 128.54 | 778.82 | 128.54 | 778.82 | 2.49E+05 | 12069 | 5.9194 | 1 | 1.84E-09 | 3.68E-09 | 3.84E-08 | TRUE |  |  |  |  |  |  |  |
| HGLibA_48466 | Rep 3 | TBC1D10B | 2 | | 143.82 | 670.39 | 143.82 | 670.39 | 1.57E+05 | 8041.5 | 5.872 | 1 | 2.28E-09 | 4.55E-09 | 4.57E-08 | TRUE |  |  |  |  |  |  |  |
| HGLibA_48468 | Rep 3 | TBC1D10B | 2 | | 79.741 | 329.1 | 79.741 | 329.1 | 34686 | 2833.8 | 4.6843 | 1 | 1.51E-06 | 3.01E-06 | 2.26E-05 | TRUE |  |  |  |  |  |  |  |
| HGLibA_49289 | Rep 1 | THAP10 | 2 | | 43.141 | 306.41 | 43.141 | 306.41 | 41796 | 2741 | 5.0286 | 1 | 3.11E-07 | 6.21E-07 | 3.61E-06 | TRUE |  |  |  |  |  |  |  |
| HGLibA_49288 | Rep 1 | THAP10 | 2 | | 53.178 | 510.69 | 53.178 | 510.69 | 1.32E+05 | 4237.9 | 7.0279 | 1 | 1.32E-12 | 2.64E-12 | 2.24E-11 | TRUE |  |  |  |  |  |  |  |
| HGLibA_49288 | Rep 3 | THAP10 | 2 | | 96.021 | 816.66 | 96.021 | 816.66 | 3.22E+05 | 3933.8 | 11.49 | 1 | 7.93E-31 | 1.59E-30 | 4.38E-29 | TRUE |  |  |  |  |  |  |  |
| HGLibA_49289 | Rep 3 | THAP10 | 2 | | 88.866 | 633.83 | 88.866 | 633.83 | 1.79E+05 | 3430.9 | 9.3038 | 1 | 7.24E-21 | 1.45E-20 | 2.90E-19 | TRUE |  |  |  |  |  |  |  |
| HGLibA_49461 | Rep 1 | TIFAB | 2 | | 16.992 | 127.67 | 16.992 | 127.67 | 7445.9 | 398.79 | 5.5423 | 1 | 1.86E-08 | 3.72E-08 | 2.40E-07 | TRUE |  |  |  |  |  |  |  |
| HGLibA_49460 | Rep 1 | TIFAB | 2 | | 17.344 | 561.75 | 17.344 | 561.75 | 2.21E+05 | 415.94 | 26.694 | 1 | 3.46E-157 | 6.92E-157 | 2.96E-155 | TRUE |  |  |  |  |  |  |  |
| HGLibA_49461 | Rep 3 | TIFAB | 2 | | 8.814 | 97.512 | 8.814 | 97.512 | 5063.2 | 62.697 | 11.202 | 1 | 2.30E-29 | 4.60E-29 | 1.23E-27 | TRUE |  |  |  |  |  |  |  |
| HGLibA_49459 | Rep 3 | TIFAB | 2 | | 50.707 | 280.35 | 50.707 | 280.35 | 30647 | 1277.5 | 6.4249 | 1 | 7.15E-11 | 1.43E-10 | 1.62E-09 | TRUE |  |  |  |  |  |  |  |
| HGLibA_49491 | Rep 2 | TIMELESS | 2 | | 353.28 | 1939.4 | 353.28 | 1939.4 | 1.46E+06 | 76564 | 5.732 | 1 | 5.52E-09 | 1.10E-08 | 1.10E-07 | TRUE |  |  |  |  |  |  |  |
| HGLibA_49490 | Rep 2 | TIMELESS | 2 | | 454.22 | 2082 | 454.22 | 2082 | 1.50E+06 | 1.21E+05 | 4.6744 | 1 | 1.63E-06 | 3.26E-06 | 2.42E-05 | TRUE |  |  |  |  |  |  |  |
| HGLibA_49491 | Rep 3 | TIMELESS | 2 | | 46.87 | 548.5 | 46.87 | 548.5 | 1.64E+05 | 1112.8 | 15.038 | 1 | 2.26E-51 | 4.53E-51 | 1.80E-49 | TRUE |  |  |  |  |  |  |  |
| HGLibA_49490 | Rep 3 | TIMELESS | 2 | | 129.2 | 767.91 | 129.2 | 767.91 | 2.40E+05 | 6650.6 | 7.8319 | 1 | 2.55E-15 | 5.09E-15 | 7.81E-14 | TRUE |  |  |  |  |  |  |  |
| HGLibA_49662 | Rep 1 | TLR7 | 2 | | 24.828 | 178.74 | 24.828 | 178.74 | 14311 | 871.01 | 5.2151 | 1 | 1.15E-07 | 2.30E-07 | 1.39E-06 | TRUE |  |  |  |  |  |  |  |
| HGLibA_49661 | Rep 1 | TLR7 | 2 | | 23.948 | 459.62 | 23.948 | 459.62 | 1.32E+05 | 808.38 | 15.323 | 1 | 3.34E-53 | 6.69E-53 | 1.53E-51 | TRUE |  |  |  |  |  |  |  |
| HGLibA_49661 | Rep 2 | TLR7 | 2 | | 119.08 | 1110.4 | 119.08 | 1110.4 | 6.17E+05 | 10497 | 9.6757 | 1 | 2.18E-22 | 4.36E-22 | 1.12E-20 | TRUE |  |  |  |  |  |  |  |
| HGLibA_49662 | Rep 2 | TLR7 | 2 | | 114.34 | 1030.7 | 114.34 | 1030.7 | 5.25E+05 | 9748.9 | 9.281 | 1 | 9.57E-21 | 1.91E-20 | 4.48E-19 | TRUE |  |  |  |  |  |  |  |
| HGLibA_50096 | Rep 2 | TMEM180 | 2 | | 96.996 | 2255.5 | 96.996 | 2255.5 | 3.33E+06 | 7222.5 | 25.399 | 1 | 1.49E-142 | 2.99E-142 | 5.52E-140 | TRUE |  |  |  |  |  |  |  |
| HGLibA_50097 | Rep 2 | TMEM180 | 2 | | 292.56 | 1430.4 | 292.56 | 1430.4 | 7.39E+05 | 54226 | 4.8863 | 1 | 5.74E-07 | 1.15E-06 | 9.01E-06 | TRUE |  |  |  |  |  |  |  |
| HGLibA_50095 | Rep 3 | TMEM180 | 2 | | 160 | 1023.9 | 160 | 1023.9 | 4.43E+05 | 9714 | 8.765 | 1 | 9.85E-19 | 1.97E-18 | 3.57E-17 | TRUE |  |  |  |  |  |  |  |
| HGLibA_50096 | Rep 3 | TMEM180 | 2 | | 11.717 | 97.512 | 11.717 | 97.512 | 4541.6 | 101.32 | 8.5233 | 1 | 8.84E-18 | 1.77E-17 | 3.07E-16 | TRUE |  |  |  |  |  |  |  |
| HGLibA_50598 | Rep 1 | TMOD2 | 2 | | 67.088 | 434.08 | 67.088 | 434.08 | 80106 | 6881.9 | 4.4239 | 1 | 6.13E-06 | 1.23E-05 | 6.31E-05 | TRUE |  |  |  |  |  |  |  |
| HGLibA_50596 | Rep 1 | TMOD2 | 2 | | 37.066 | 434.08 | 37.066 | 434.08 | 1.02E+05 | 1999 | 8.8797 | 1 | 4.21E-19 | 8.42E-19 | 9.55E-18 | TRUE |  |  |  |  |  |  |  |
| HGLibA_50597 | Rep 3 | TMOD2 | 2 | | 134.18 | 658.21 | 134.18 | 658.21 | 1.57E+05 | 7111.1 | 6.2142 | 1 | 2.73E-10 | 5.46E-10 | 5.92E-09 | TRUE |  |  |  |  |  |  |  |
| HGLibA_50596 | Rep 3 | TMOD2 | 2 | | 49.359 | 207.21 | 49.359 | 207.21 | 13931 | 1218.5 | 4.5221 | 1 | 3.32E-06 | 6.64E-06 | 4.79E-05 | TRUE |  |  |  |  |  |  |  |
| HGLibA_50904 | Rep 2 | TNPO3 | 2 | | 13.406 | 376.56 | 13.406 | 376.56 | 96573 | 202.69 | 25.508 | 1 | 9.73E-144 | 1.95E-143 | 3.63E-141 | TRUE |  |  |  |  |  |  |  |
| HGLibA_50903 | Rep 2 | TNPO3 | 2 | | 807.51 | 6770.4 | 807.51 | 6770.4 | 2.20E+07 | 3.48E+05 | 10.112 | 1 | 2.68E-24 | 5.35E-24 | 1.51E-22 | TRUE |  |  |  |  |  |  |  |
| HGLibA_50904 | Rep 3 | TNPO3 | 2 | | 7.466 | 73.134 | 7.466 | 73.134 | 2724.7 | 47.524 | 9.5257 | 1 | 9.53E-22 | 1.91E-21 | 3.93E-20 | TRUE |  |  |  |  |  |  |  |
| HGLibA_50905 | Rep 3 | TNPO3 | 2 | | 76.319 | 487.56 | 76.319 | 487.56 | 1.00E+05 | 2622.8 | 8.0299 | 1 | 5.23E-16 | 1.05E-15 | 1.66E-14 | TRUE |  |  |  |  |  |  |  |
| HGLibA_51854 | Rep 1 | TSGA13 | 2 | | 33.28 | 306.41 | 33.28 | 306.41 | 46766 | 1598.2 | 6.8321 | 1 | 5.25E-12 | 1.05E-11 | 8.57E-11 | TRUE |  |  |  |  |  |  |  |
| HGLibA_51853 | Rep 1 | TSGA13 | 2 | | 38.651 | 1174.6 | 38.651 | 1174.6 | 9.55E+05 | 2180.8 | 24.324 | 1 | 6.80E-131 | 1.36E-130 | 5.27E-129 | TRUE |  |  |  |  |  |  |  |
| HGLibA_51852 | Rep 3 | TSGA13 | 2 | | 16.28 | 231.59 | 16.28 | 231.59 | 30975 | 177.51 | 16.161 | 1 | 5.38E-59 | 1.08E-58 | 4.62E-57 | TRUE |  |  |  |  |  |  |  |
| HGLibA_51853 | Rep 3 | TSGA13 | 2 | | 45.522 | 280.35 | 45.522 | 280.35 | 32556 | 1057.3 | 7.2217 | 1 | 2.79E-13 | 5.58E-13 | 7.60E-12 | TRUE |  |  |  |  |  |  |  |
| HGLibA_52224 | Rep 1 | TTPAL | 2 | | 36.626 | 331.95 | 36.626 | 331.95 | 54546 | 1950 | 6.6877 | 1 | 1.42E-11 | 2.85E-11 | 2.28E-10 | TRUE |  |  |  |  |  |  |  |
| HGLibA_52226 | Rep 1 | TTPAL | 2 | | 58.196 | 612.82 | 58.196 | 612.82 | 1.97E+05 | 5114.8 | 7.7551 | 1 | 5.57E-15 | 1.11E-14 | 1.07E-13 | TRUE |  |  |  |  |  |  |  |
| HGLibA_52224 | Rep 3 | TTPAL | 2 | | 48.218 | 450.99 | 48.218 | 450.99 | 1.02E+05 | 1169.5 | 11.778 | 1 | 2.77E-32 | 5.53E-32 | 1.58E-30 | TRUE |  |  |  |  |  |  |  |
| HGLibA_52226 | Rep 3 | TTPAL | 2 | | 73.83 | 572.88 | 73.83 | 572.88 | 1.52E+05 | 2473.9 | 10.034 | 1 | 5.83E-24 | 1.17E-23 | 2.64E-22 | TRUE |  |  |  |  |  |  |  |
| HGLibA_52436 | Rep 1 | TXNL1 | 2 | | 65.239 | 714.96 | 65.239 | 714.96 | 2.72E+05 | 6491.8 | 8.0639 | 1 | 4.67E-16 | 9.35E-16 | 9.34E-15 | TRUE |  |  |  |  |  |  |  |
| HGLibA_52434 | Rep 1 | TXNL1 | 2 | | 74.044 | 944.77 | 74.044 | 944.77 | 4.99E+05 | 8456.3 | 9.4687 | 1 | 1.79E-21 | 3.59E-21 | 4.42E-20 | TRUE |  |  |  |  |  |  |  |
| HGLibA_52436 | Rep 2 | TXNL1 | 2 | | 449.49 | 3620.4 | 449.49 | 3620.4 | 6.18E+06 | 1.19E+05 | 9.1932 | 1 | 2.11E-20 | 4.22E-20 | 9.71E-19 | TRUE |  |  |  |  |  |  |  |
| HGLibA_52434 | Rep 2 | TXNL1 | 2 | | 577.24 | 2569.1 | 577.24 | 2569.1 | 2.24E+06 | 1.88E+05 | 4.5932 | 1 | 2.40E-06 | 4.81E-06 | 3.49E-05 | TRUE |  |  |  |  |  |  |  |
| HGLibA_52493 | Rep 2 | U2AF1 | 2 | | 126.17 | 1238.9 | 126.17 | 1238.9 | 7.84E+05 | 11666 | 10.302 | 1 | 3.92E-25 | 7.84E-25 | 2.29E-23 | TRUE |  |  |  |  |  |  |  |
| HGLibA_52491 | Rep 2 | U2AF1 | 2 | | 127.75 | 1046.1 | 127.75 | 1046.1 | 5.20E+05 | 11934 | 8.407 | 1 | 2.39E-17 | 4.79E-17 | 9.17E-16 | TRUE |  |  |  |  |  |  |  |
| HGLibA_52493 | Rep 3 | U2AF1 | 2 | | 39.611 | 207.21 | 39.611 | 207.21 | 16194 | 828.85 | 5.8216 | 1 | 3.18E-09 | 6.37E-09 | 6.31E-08 | TRUE |  |  |  |  |  |  |  |
| HGLibA_52491 | Rep 3 | U2AF1 | 2 | | 29.553 | 134.08 | 29.553 | 134.08 | 6172.6 | 497.25 | 4.6874 | 1 | 1.52E-06 | 3.05E-06 | 2.29E-05 | TRUE |  |  |  |  |  |  |  |
| HGLibA_52514 | Rep 1 | UBA1 | 2 | | 28.526 | 229.81 | 28.526 | 229.81 | 24893 | 1160.9 | 5.9075 | 1 | 2.17E-09 | 4.35E-09 | 3.03E-08 | TRUE |  |  |  |  |  |  |  |
| HGLibA_52513 | Rep 1 | UBA1 | 2 | | 32.224 | 561.75 | 32.224 | 561.75 | 1.93E+05 | 1494.7 | 13.697 | 1 | 6.67E-43 | 1.33E-42 | 2.63E-41 | TRUE |  |  |  |  |  |  |  |
| HGLibA_52513 | Rep 3 | UBA1 | 2 | | 37.019 | 463.18 | 37.019 | 463.18 | 1.19E+05 | 736.38 | 15.705 | 1 | 7.71E-56 | 1.54E-55 | 6.45E-54 | TRUE |  |  |  |  |  |  |  |
| HGLibA_52514 | Rep 3 | UBA1 | 2 | | 58.276 | 548.5 | 58.276 | 548.5 | 1.51E+05 | 1631.2 | 12.138 | 1 | 3.59E-34 | 7.18E-34 | 2.14E-32 | TRUE |  |  |  |  |  |  |  |
| HGLibA_52712 | Rep 1 | UBL5 | 2 | | 10.477 | 76.603 | 10.477 | 76.603 | 2645.8 | 148.87 | 5.4197 | 1 | 3.71E-08 | 7.42E-08 | 4.69E-07 | TRUE |  |  |  |  |  |  |  |
| HGLibA_52710 | Rep 1 | UBL5 | 2 | | 11.622 | 459.62 | 11.622 | 459.62 | 1.53E+05 | 183.65 | 33.058 | 1 | 7.36E-240 | 1.47E-239 | 7.82E-238 | TRUE |  |  |  |  |  |  |  |
| HGLibA_52712 | Rep 2 | UBL5 | 2 | | 62.298 | 1666.9 | 62.298 | 1666.9 | 1.88E+06 | 3226.7 | 28.248 | 1 | 8.80E-176 | 1.76E-175 | 3.98E-173 | TRUE |  |  |  |  |  |  |  |
| HGLibA_52711 | Rep 2 | UBL5 | 2 | | 508.64 | 3428.9 | 508.64 | 3428.9 | 5.10E+06 | 1.49E+05 | 7.5609 | 1 | 2.21E-14 | 4.42E-14 | 6.98E-13 | TRUE |  |  |  |  |  |  |  |
| HGLibA_52721 | Rep 2 | UBN1 | 2 | | 656.1 | 2918.7 | 656.1 | 2918.7 | 2.89E+06 | 2.38E+05 | 4.6403 | 1 | 1.91E-06 | 3.82E-06 | 2.81E-05 | TRUE |  |  |  |  |  |  |  |
| HGLibA_52720 | Rep 2 | UBN1 | 2 | | 344.61 | 1551.2 | 344.61 | 1551.2 | 8.22E+05 | 73160 | 4.461 | 1 | 4.54E-06 | 9.08E-06 | 6.34E-05 | TRUE |  |  |  |  |  |  |  |
| HGLibA_52720 | Rep 3 | UBN1 | 2 | | 38.885 | 450.99 | 38.885 | 450.99 | 1.10E+05 | 802.47 | 14.548 | 1 | 3.30E-48 | 6.60E-48 | 2.45E-46 | TRUE |  |  |  |  |  |  |  |
| HGLibA_52721 | Rep 3 | UBN1 | 2 | | 144.24 | 585.07 | 144.24 | 585.07 | 1.08E+05 | 8082.7 | 4.9034 | 1 | 4.98E-07 | 9.96E-07 | 7.94E-06 | TRUE |  |  |  |  |  |  |  |
| HGLibA_52755 | Rep 1 | UBR4 | 2 | | 44.99 | 331.95 | 44.99 | 331.95 | 49939 | 2991.2 | 5.2467 | 1 | 9.74E-08 | 1.95E-07 | 1.18E-06 | TRUE |  |  |  |  |  |  |  |
| HGLibA_52756 | Rep 1 | UBR4 | 2 | | 62.246 | 510.69 | 62.246 | 510.69 | 1.24E+05 | 5885.6 | 5.8453 | 1 | 3.19E-09 | 6.39E-09 | 4.38E-08 | TRUE |  |  |  |  |  |  |  |
| HGLibA_52756 | Rep 2 | UBR4 | 2 | | 436.87 | 3588.2 | 436.87 | 3588.2 | 6.12E+06 | 1.13E+05 | 9.3778 | 1 | 3.73E-21 | 7.46E-21 | 1.78E-19 | TRUE |  |  |  |  |  |  |  |
| HGLibA_52757 | Rep 2 | UBR4 | 2 | | 189.26 | 1571.8 | 189.26 | 1571.8 | 1.18E+06 | 24458 | 8.8402 | 1 | 5.39E-19 | 1.08E-18 | 2.28E-17 | TRUE |  |  |  |  |  |  |  |
| HGLibA_53403 | Rep 1 | USP6 | 2 | | 121.5 | 995.84 | 121.5 | 995.84 | 4.71E+05 | 23821 | 5.665 | 1 | 9.37E-09 | 1.87E-08 | 1.24E-07 | TRUE |  |  |  |  |  |  |  |
| HGLibA_53405 | Rep 1 | USP6 | 2 | | 46.663 | 485.15 | 46.663 | 485.15 | 1.23E+05 | 3227.5 | 7.7184 | 1 | 7.42E-15 | 1.48E-14 | 1.41E-13 | TRUE |  |  |  |  |  |  |  |
| HGLibA_53405 | Rep 3 | USP6 | 2 | | 121.53 | 706.96 | 121.53 | 706.96 | 2.01E+05 | 5967.3 | 7.5786 | 1 | 1.85E-14 | 3.71E-14 | 5.44E-13 | TRUE |  |  |  |  |  |  |  |
| HGLibA_53403 | Rep 3 | USP6 | 2 | | 194.12 | 719.15 | 194.12 | 719.15 | 1.52E+05 | 13687 | 4.4877 | 1 | 3.78E-06 | 7.57E-06 | 5.42E-05 | TRUE |  |  |  |  |  |  |  |
| HGLibA_55052 | Rep 1 | ZCCHC9 | 2 | | 66.824 | 536.22 | 66.824 | 536.22 | 1.35E+05 | 6825.4 | 5.6816 | 1 | 8.44E-09 | 1.69E-08 | 1.12E-07 | TRUE |  |  |  |  |  |  |  |
| HGLibA_55051 | Rep 1 | ZCCHC9 | 2 | | 19.369 | 178.74 | 19.369 | 178.74 | 15923 | 521.91 | 6.976 | 1 | 1.89E-12 | 3.79E-12 | 3.18E-11 | TRUE |  |  |  |  |  |  |  |
| HGLibA_55051 | Rep 2 | ZCCHC9 | 2 | | 201.09 | 1569.2 | 201.09 | 1569.2 | 1.15E+06 | 27323 | 8.2768 | 1 | 7.13E-17 | 1.43E-16 | 2.65E-15 | TRUE |  |  |  |  |  |  |  |
| HGLibA_55050 | Rep 2 | ZCCHC9 | 2 | | 652.16 | 4099.8 | 652.16 | 4099.8 | 7.04E+06 | 2.35E+05 | 7.1098 | 1 | 6.38E-13 | 1.28E-12 | 1.80E-11 | TRUE |  |  |  |  |  |  |  |
| HGLibA_55083 | Rep 2 | ZDHHC16 | 2 | | 324.9 | 3551 | 324.9 | 3551 | 6.70E+06 | 65686 | 12.587 | 1 | 1.38E-36 | 2.76E-36 | 1.21E-34 | TRUE |  |  |  |  |  |  |  |
| HGLibA_55085 | Rep 2 | ZDHHC16 | 2 | | 428.2 | 1871.2 | 428.2 | 1871.2 | 1.17E+06 | 1.09E+05 | 4.3737 | 1 | 6.77E-06 | 1.35E-05 | 9.24E-05 | TRUE |  |  |  |  |  |  |  |
| HGLibA_55084 | Rep 3 | ZDHHC16 | 2 | | 20.013 | 341.29 | 20.013 | 341.29 | 70774 | 253.14 | 20.193 | 1 | 6.28E-91 | 1.26E-90 | 6.91E-89 | TRUE |  |  |  |  |  |  |  |
| HGLibA_55083 | Rep 3 | ZDHHC16 | 2 | | 79.534 | 487.56 | 79.534 | 487.56 | 98236 | 2820.8 | 7.6825 | 1 | 8.36E-15 | 1.67E-14 | 2.49E-13 | TRUE |  |  |  |  |  |  |  |
| HGLibA_55151 | Rep 2 | ZFAND2B | 2 | | 842.21 | 10438 | 842.21 | 10438 | 6.04E+07 | 3.76E+05 | 15.658 | 1 | 1.60E-55 | 3.20E-55 | 2.18E-53 | TRUE |  |  |  |  |  |  |  |
| HGLibA_55149 | Rep 2 | ZFAND2B | 2 | | 108.82 | 1318.6 | 108.82 | 1318.6 | 9.56E+05 | 8907.9 | 12.818 | 1 | 7.43E-38 | 1.49E-37 | 6.74E-36 | TRUE |  |  |  |  |  |  |  |
| HGLibA_55149 | Rep 3 | ZFAND2B | 2 | | 27.272 | 731.34 | 27.272 | 731.34 | 3.61E+05 | 432.45 | 33.857 | 1 | 1.60E-251 | 3.19E-251 | 2.47E-249 | TRUE |  |  |  |  |  |  |  |
| HGLibA_55151 | Rep 3 | ZFAND2B | 2 | | 140.09 | 658.21 | 140.09 | 658.21 | 1.52E+05 | 7675.4 | 5.9139 | 1 | 1.77E-09 | 3.53E-09 | 3.58E-08 | TRUE |  |  |  |  |  |  |  |
| HGLibA_56711 | Rep 1 | ZNF772 | 2 | | 90.244 | 944.77 | 90.244 | 944.77 | 4.67E+05 | 12787 | 7.5569 | 1 | 2.62E-14 | 5.24E-14 | 4.83E-13 | TRUE |  |  |  |  |  |  |  |
| HGLibA_56709 | Rep 1 | ZNF772 | 2 | | 33.72 | 408.55 | 33.72 | 408.55 | 91744 | 1642.4 | 9.2489 | 1 | 1.42E-20 | 2.84E-20 | 3.39E-19 | TRUE |  |  |  |  |  |  |  |
| HGLibA_56709 | Rep 2 | ZNF772 | 2 | | 247.61 | 2141.1 | 247.61 | 2141.1 | 2.23E+06 | 39971 | 9.471 | 1 | 1.55E-21 | 3.11E-21 | 7.56E-20 | TRUE |  |  |  |  |  |  |  |
| HGLibA_56710 | Rep 2 | ZNF772 | 2 | | 503.12 | 3106.3 | 503.12 | 3106.3 | 4.00E+06 | 1.46E+05 | 6.8077 | 1 | 5.48E-12 | 1.10E-11 | 1.44E-10 | TRUE |  |  |  |  |  |  |  |
| HGLibA_56893 | Rep 1 | ZNF880 | 2 | | 38.387 | 485.15 | 38.387 | 485.15 | 1.31E+05 | 2149.9 | 9.6353 | 1 | 3.56E-22 | 7.12E-22 | 8.91E-21 | TRUE |  |  |  |  |  |  |  |
| HGLibA_56892 | Rep 1 | ZNF880 | 2 | | 48.247 | 740.49 | 48.247 | 740.49 | 3.24E+05 | 3460 | 11.768 | 1 | 3.57E-32 | 7.15E-32 | 1.18E-30 | TRUE |  |  |  |  |  |  |  |
| HGLibA_56892 | Rep 2 | ZNF880 | 2 | | 327.26 | 2234.9 | 327.26 | 2234.9 | 2.18E+06 | 66564 | 7.3941 | 1 | 7.93E-14 | 1.59E-13 | 2.41E-12 | TRUE |  |  |  |  |  |  |  |
| HGLibA_56894 | Rep 2 | ZNF880 | 2 | | 514.16 | 3273.4 | 514.16 | 3273.4 | 4.52E+06 | 1.52E+05 | 7.0738 | 1 | 8.32E-13 | 1.66E-12 | 2.33E-11 | TRUE |  |  |  |  |  |  |  |

**Table S2B: List of over-represented pathways identified for the candidate genes using InnateDB.**

| **Pathway Name** | **Pathway Id** | **Source Name** | **Organism** | **Pathway uploaded gene count** | **Genes in InnateDB for this entity** | **Pathway p-value** | **Pathway p-value (corrected)** | **Gene Symbols** |
| --- | --- | --- | --- | --- | --- | --- | --- | --- |
| Role of pi3k subunit p85 in regulation of actin organization and cell migration | 4121 | PID BIOCARTA | 9606 | 3 | 17 | 0.00 | 0.02 | ACTR3; ARPC3; ARPC4; |
| Y branching of actin filaments | 3933 | PID BIOCARTA | 9606 | 3 | 17 | 0.00 | 0.02 | ACTR3; ARPC3; ARPC4; |
| EPHB-mediated forward signalling | 17191 | REACTOME | 9606 | 4 | 39 | 0.00 | 0.03 | ACTR3; ARPC3; ARPC4; EFNB3; |
| How does salmonella hijack a cell | 4051 | PID BIOCARTA | 9606 | 3 | 12 | 0.00 | 0.03 | ACTR3; ARPC3; ARPC4; |
| EPH-Ephrin signalling | 19189 | REACTOME | 9606 | 5 | 91 | 0.00 | 0.03 | ACTR3; ARPC3; ARPC4; CLTCL1; EFNB3; |
| Fcgamma receptor (FCGR) dependent phagocytosis | 18888 | REACTOME | 9606 | 5 | 89 | 0.00 | 0.03 | ACTR3; ARPC3; ARPC4; CYFIP2; FCGR1A; |
| Regulation of actin dynamics for phagocytic cup formation | 13005 | REACTOME | 9606 | 5 | 66 | 0.00 | 0.03 | ACTR3; ARPC3; ARPC4; CYFIP2; FCGR1A; |
| PDGFR-beta signalling pathway | 15816 | PID NCI | 9606 | 5 | 85 | 0.00 | 0.03 | ACTR3; ARPC3; ARPC4; CYFIP2; PDGFB; |
| RAC1 signalling pathway | 15344 | PID NCI | 9606 | 4 | 52 | 0.00 | 0.03 | ACTR3; ARPC3; ARPC4; CYFIP2; |
| Collagen degradation | 13392 | REACTOME | 9606 | 4 | 61 | 0.00 | 0.04 | ADAM9; COL4A6; COL5A1; CTSL1; |
| Interferon Signalling | 18059 | REACTOME | 9606 | 6 | 158 | 0.00 | 0.06 | FCGR1A; FCGR1B; IFIT3; KPNA5; NUP62; PIAS1; |
| Antigen processing-Cross presentation | 18220 | REACTOME | 9606 | 4 | 80 | 0.01 | 0.08 | CTSL1; FCGR1A; FCGR1B; PSMA3; |
| Trafficking and processing of endosomal TLR | 13047 | REACTOME | 9606 | 2 | 13 | 0.01 | 0.09 | CTSL1; TLR7; |
| Sumoylation by ranbp2 regulates transcriptional repression | 3990 | PID BIOCARTA | 9606 | 2 | 14 | 0.01 | 0.09 | NUP62; PIAS1; |
| ErbB1 downstream signalling | 15233 | PID NCI | 9606 | 4 | 85 | 0.01 | 0.09 | ACTR3; ARPC3; ARPC4; CYFIP2; |
| Immune System | 18444 | REACTOME | 9606 | 19 | 1127 | 0.01 | 0.09 | ACTR3; ARPC3; ARPC4; CFD; CTSL1; CYFIP2; FCGR1A; FCGR1B; IFIT3; IL1RN; KPNA5; NUP62; PDGFB; PIAS1; POLR3H; PSMA3; TLR7; UBA1; UBR4; |
| Cross-presentation of soluble exogenous antigens (endosomes) | 12985 | REACTOME | 9606 | 3 | 49 | 0.01 | 0.11 | FCGR1A; FCGR1B; PSMA3; |
| Cytokine Signalling in Immune system | 17418 | REACTOME | 9606 | 7 | 267 | 0.01 | 0.12 | FCGR1A; FCGR1B; IFIT3; IL1RN; KPNA5; NUP62; PIAS1; |
| Assembly of collagen fibrils and other multimeric structures | 13386 | REACTOME | 9606 | 3 | 54 | 0.01 | 0.13 | COL4A6; COL5A1; CTSL1; |
| Non-integrin membrane-ECM interactions | 18527 | REACTOME | 9606 | 3 | 59 | 0.02 | 0.16 | COL4A6; COL5A1; PDGFB; |
| Proteasome complex | 4043 | PID BIOCARTA | 9606 | 2 | 23 | 0.02 | 0.16 | PSMA3; UBA1; |
| Interferon gamma signalling | 13077 | REACTOME | 9606 | 3 | 63 | 0.02 | 0.17 | FCGR1A; FCGR1B; PIAS1; |
| CDC42 signalling events | 15467 | PID NCI | 9606 | 3 | 61 | 0.02 | 0.17 | ACTR3; ARPC3; ARPC4; |
| Degradation of the extracellular matrix | 17498 | REACTOME | 9606 | 4 | 116 | 0.02 | 0.17 | ADAM9; COL4A6; COL5A1; CTSL1; |
| Glycosaminoglycan biosynthesis | 458 | KEGG | 9606 | 2 | 26 | 0.02 | 0.18 | B3GALT6; HS6ST2; |
| GPCR GroupI metabotropic glutamate receptor signalling pathway | 16198 | INOH | 9606 | 2 | 28 | 0.03 | 0.19 | GNAQ; GRM5; |
| Lysosome | 4356 | KEGG | 9606 | 4 | 123 | 0.03 | 0.19 | CLTCL1; CTSL1; LAMP1; NPC1; |
| Bacterial invasion of epithelial cells | 10364 | KEGG | 9606 | 3 | 77 | 0.03 | 0.20 | ARPC3; ARPC4; CLTCL1; |
| Arf6 signalling events | 15819 | PID NCI | 9606 | 2 | 31 | 0.03 | 0.20 | GNAQ; USP6; |
| Mucin type O-Glycan biosynthesis | 508 | KEGG | 9606 | 2 | 31 | 0.03 | 0.20 | GALNT10; GCNT4; |
| Adherens junctions interactions | 13856 | REACTOME | 9606 | 2 | 30 | 0.03 | 0.20 | CDH7; CDH8; |
| Class I MHC mediated antigen processing & presentation | 19282 | REACTOME | 9606 | 6 | 256 | 0.03 | 0.20 | CTSL1; FCGR1A; FCGR1B; PSMA3; UBA1; UBR4; |
| Innate Immune System | 17476 | REACTOME | 9606 | 10 | 563 | 0.03 | 0.21 | ACTR3; ARPC3; ARPC4; CFD; CTSL1; CYFIP2; FCGR1A; PDGFB; POLR3H; TLR7; |
| Mitochondrial translation elongation | 19088 | REACTOME | 9606 | 3 | 82 | 0.04 | 0.22 | MRPL20; MRPL27; MRPS33; |
| Mitochondrial translation initiation | 17759 | REACTOME | 9606 | 3 | 82 | 0.04 | 0.22 | MRPL20; MRPL27; MRPS33; |
| Mitochondrial translation termination | 17858 | REACTOME | 9606 | 3 | 83 | 0.04 | 0.22 | MRPL20; MRPL27; MRPS33; |
| Collagen formation | 19746 | REACTOME | 9606 | 3 | 85 | 0.04 | 0.22 | COL4A6; COL5A1; CTSL1; |
| Defective B3GAT3 causes JDSSDHD | 18524 | REACTOME | 9606 | 3 | 120 | 0.10 | 0.22 | B3GALT6; B3GNT1; HS6ST2; |
| Defective B4GALT1 causes B4GALT1-CDG (CDG-2d) | 18861 | REACTOME | 9606 | 3 | 120 | 0.10 | 0.22 | B3GALT6; B3GNT1; HS6ST2; |
| Defective B4GALT7 causes EDS, progeroid type | 17098 | REACTOME | 9606 | 3 | 120 | 0.10 | 0.22 | B3GALT6; B3GNT1; HS6ST2; |
| Defective CHST14 causes EDS, musculocontractural type | 18372 | REACTOME | 9606 | 3 | 120 | 0.10 | 0.22 | B3GALT6; B3GNT1; HS6ST2; |
| Defective CHST3 causes SEDCJD | 18611 | REACTOME | 9606 | 3 | 120 | 0.10 | 0.22 | B3GALT6; B3GNT1; HS6ST2; |
| Defective CHST6 causes MCDC1 | 17102 | REACTOME | 9606 | 3 | 120 | 0.10 | 0.22 | B3GALT6; B3GNT1; HS6ST2; |
| Defective CHSY1 causes TPBS | 19354 | REACTOME | 9606 | 3 | 120 | 0.10 | 0.22 | B3GALT6; B3GNT1; HS6ST2; |
| Defective EXT1 causes exostoses 1, TRPS2 and CHDS | 18206 | REACTOME | 9606 | 3 | 120 | 0.10 | 0.22 | B3GALT6; B3GNT1; HS6ST2; |
| Defective EXT2 causes exostoses 2 | 18636 | REACTOME | 9606 | 3 | 120 | 0.10 | 0.22 | B3GALT6; B3GNT1; HS6ST2; |
| Defective PAPSS2 causes SEMD-PA | 17298 | REACTOME | 9606 | 3 | 120 | 0.10 | 0.22 | B3GALT6; B3GNT1; HS6ST2; |
| Defective SLC26A2 causes chondrodysplasias | 17794 | REACTOME | 9606 | 3 | 120 | 0.10 | 0.22 | B3GALT6; B3GNT1; HS6ST2; |
| Diseases associated with glycosaminoglycan metabolism | 18653 | REACTOME | 9606 | 3 | 120 | 0.10 | 0.22 | B3GALT6; B3GNT1; HS6ST2; |
| Diseases of glycosylation | 19039 | REACTOME | 9606 | 3 | 120 | 0.10 | 0.22 | B3GALT6; B3GNT1; HS6ST2; |
| Glycosaminoglycan metabolism | 18895 | REACTOME | 9606 | 3 | 120 | 0.10 | 0.22 | B3GALT6; B3GNT1; HS6ST2; |
| Late Phase of HIV Life Cycle | 13791 | REACTOME | 9606 | 3 | 120 | 0.10 | 0.22 | CHMP6; NUP62; SSRP1; |
| MPS I - Hurler syndrome | 19525 | REACTOME | 9606 | 3 | 120 | 0.10 | 0.22 | B3GALT6; B3GNT1; HS6ST2; |
| MPS II - Hunter syndrome | 18749 | REACTOME | 9606 | 3 | 120 | 0.10 | 0.22 | B3GALT6; B3GNT1; HS6ST2; |
| MPS IIIA - Sanfilippo syndrome A | 19535 | REACTOME | 9606 | 3 | 120 | 0.10 | 0.22 | B3GALT6; B3GNT1; HS6ST2; |
| MPS IIIB - Sanfilippo syndrome B | 19886 | REACTOME | 9606 | 3 | 120 | 0.10 | 0.22 | B3GALT6; B3GNT1; HS6ST2; |
| MPS IIIC - Sanfilippo syndrome C | 19585 | REACTOME | 9606 | 3 | 120 | 0.10 | 0.22 | B3GALT6; B3GNT1; HS6ST2; |
| MPS IIID - Sanfilippo syndrome D | 17539 | REACTOME | 9606 | 3 | 120 | 0.10 | 0.22 | B3GALT6; B3GNT1; HS6ST2; |
| MPS IV - Morquio syndrome A | 18634 | REACTOME | 9606 | 3 | 120 | 0.10 | 0.22 | B3GALT6; B3GNT1; HS6ST2; |
| MPS IV - Morquio syndrome B | 18458 | REACTOME | 9606 | 3 | 120 | 0.10 | 0.22 | B3GALT6; B3GNT1; HS6ST2; |
| MPS IX - Natowicz syndrome | 18165 | REACTOME | 9606 | 3 | 120 | 0.10 | 0.22 | B3GALT6; B3GNT1; HS6ST2; |
| MPS VI - Maroteaux-Lamy syndrome | 17269 | REACTOME | 9606 | 3 | 120 | 0.10 | 0.22 | B3GALT6; B3GNT1; HS6ST2; |
| MPS VII - Sly syndrome | 19185 | REACTOME | 9606 | 3 | 120 | 0.10 | 0.22 | B3GALT6; B3GNT1; HS6ST2; |
| Mucopolysaccharidoses | 16934 | REACTOME | 9606 | 3 | 120 | 0.10 | 0.22 | B3GALT6; B3GNT1; HS6ST2; |
| O-linked glycosylation of mucins | 13368 | REACTOME | 9606 | 2 | 58 | 0.10 | 0.22 | GALNT10; GCNT4; |
| Aurora B signalling | 15698 | PID NCI | 9606 | 2 | 39 | 0.05 | 0.22 | DES; PSMA3; |
| Signalling events mediated by TCPTP | 15420 | PID NCI | 9606 | 2 | 36 | 0.04 | 0.22 | PDGFB; PIAS1; |
| ECM-receptor interaction | 468 | KEGG | 9606 | 3 | 88 | 0.05 | 0.23 | COL4A6; COL5A1; GP1BA; |
| Hematopoietic cell lineage | 415 | KEGG | 9606 | 3 | 88 | 0.05 | 0.23 | FCGR1A; GP1BA; IL4R; |
| Mitochondrial translation | 16906 | REACTOME | 9606 | 3 | 89 | 0.05 | 0.23 | MRPL20; MRPL27; MRPS33; |
| Gap junction | 436 | KEGG | 9606 | 3 | 90 | 0.05 | 0.23 | GNAQ; GRM5; PDGFB; |
| Cell-cell junction organization | 19252 | REACTOME | 9606 | 2 | 60 | 0.10 | 0.23 | CDH7; CDH8; |
| Stabilization and expansion of the E-cadherin adherens junction | 15724 | PID NCI | 9606 | 2 | 41 | 0.05 | 0.23 | AQP3; CYFIP2; |
| Huntington's disease | 4392 | KEGG | 9606 | 4 | 198 | 0.11 | 0.23 | CLTCL1; COX7C; GNAQ; GRM5; |
| Fc gamma R-mediated phagocytosis | 4359 | KEGG | 9606 | 3 | 93 | 0.05 | 0.23 | ARPC3; ARPC4; FCGR1A; |
| Shigellosis | 10374 | KEGG | 9606 | 2 | 62 | 0.11 | 0.24 | ARPC3; ARPC4; |
| Asymmetric localization of PCP proteins | 18870 | REACTOME | 9606 | 2 | 63 | 0.11 | 0.24 | FZD2; PSMA3; |
| Collagen biosynthesis and modifying enzymes | 13387 | REACTOME | 9606 | 2 | 64 | 0.12 | 0.24 | COL4A6; COL5A1; |
| HIV Life Cycle | 18642 | REACTOME | 9606 | 3 | 131 | 0.12 | 0.25 | CHMP6; NUP62; SSRP1; |
| Long-term potentiation | 507 | KEGG | 9606 | 2 | 67 | 0.12 | 0.26 | GNAQ; GRM5; |
| Antiviral mechanism by IFN-stimulated genes | 17226 | REACTOME | 9606 | 2 | 68 | 0.13 | 0.26 | KPNA5; NUP62; |
| ISG15 antiviral mechanism | 13075 | REACTOME | 9606 | 2 | 68 | 0.13 | 0.26 | KPNA5; NUP62; |
| HIV Infection | 19164 | REACTOME | 9606 | 4 | 209 | 0.12 | 0.26 | CHMP6; NUP62; PSMA3; SSRP1; |
| GPCR signalling | 16218 | INOH | 9606 | 5 | 293 | 0.13 | 0.26 | FZD2; GDF7; GNAQ; GRM5; PDGFB; |
| Antigen processing and presentation | 493 | KEGG | 9606 | 2 | 71 | 0.14 | 0.27 | CTSL1; KLRC4; |
| Organelle biogenesis and maintenance | 16766 | REACTOME | 9606 | 3 | 142 | 0.14 | 0.27 | MRPL20; MRPL27; MRPS33; |
| Regulation of actin cytoskeleton | 404 | KEGG | 9606 | 4 | 217 | 0.14 | 0.27 | ARPC3; ARPC4; CYFIP2; PDGFB; |
| Platelet activation, signalling and aggregation | 17662 | REACTOME | 9606 | 4 | 219 | 0.14 | 0.27 | CFD; GNAQ; GP1BA; PDGFB; |
| Translation | 17867 | REACTOME | 9606 | 3 | 145 | 0.15 | 0.27 | EIF2B2; EIF3I; SRPRB; |
| Arrhythmogenic right ventricular cardiomyopathy (ARVC) | 4353 | KEGG | 9606 | 2 | 74 | 0.15 | 0.27 | ATP2A2; DES; |
| ECM proteoglycans | 18499 | REACTOME | 9606 | 2 | 74 | 0.15 | 0.27 | COL4A6; COL5A1; |
| Platelet homeostasis | 13108 | REACTOME | 9606 | 2 | 76 | 0.15 | 0.28 | ATP2A2; PDE5A; |
| EPH-ephrin mediated repulsion of cells | 19071 | REACTOME | 9606 | 2 | 48 | 0.07 | 0.29 | CLTCL1; EFNB3; |
| RNA transport | 10361 | KEGG | 9606 | 4 | 170 | 0.07 | 0.29 | CYFIP2; EIF2B2; EIF3I; NUP62; |
| Validated transcriptional targets of deltaNp63 isoforms | 15671 | PID NCI | 9606 | 2 | 47 | 0.07 | 0.29 | COL5A1; RRAD; |
| Platelet degranulation | 13130 | REACTOME | 9606 | 2 | 78 | 0.16 | 0.29 | CFD; PDGFB; |
| Cardiac muscle contraction | 4399 | KEGG | 9606 | 2 | 79 | 0.16 | 0.29 | ATP2A2; COX7C; |
| Phagosome | 10394 | KEGG | 9606 | 3 | 152 | 0.16 | 0.29 | CTSL1; FCGR1A; LAMP1; |
| Hypertrophic cardiomyopathy (HCM) | 4395 | KEGG | 9606 | 2 | 83 | 0.18 | 0.30 | ATP2A2; DES; |
| Integrin cell surface interactions | 13303 | REACTOME | 9606 | 2 | 83 | 0.18 | 0.30 | COL4A6; COL5A1; |
| Response to elevated platelet cytosolic Ca2+ | 13131 | REACTOME | 9606 | 2 | 83 | 0.18 | 0.30 | CFD; PDGFB; |
| Processing of Capped Intron-Containing Pre-mRNA | 13751 | REACTOME | 9606 | 3 | 115 | 0.09 | 0.30 | CPSF2; CWC15; SMC1A; |
| Heparan sulfate/heparin (HS-GAG) metabolism | 18404 | REACTOME | 9606 | 2 | 54 | 0.09 | 0.31 | B3GALT6; HS6ST2; |
| Amoebiasis | 10358 | KEGG | 9606 | 3 | 109 | 0.08 | 0.31 | COL4A6; COL5A1; GNAQ; |
| mRNA Splicing | 19346 | REACTOME | 9606 | 3 | 111 | 0.08 | 0.31 | CPSF2; CWC15; SMC1A; |
| mRNA Splicing - Major Pathway | 13745 | REACTOME | 9606 | 3 | 111 | 0.08 | 0.31 | CPSF2; CWC15; SMC1A; |
| Pathogenic Escherichia coli infection | 434 | KEGG | 9606 | 2 | 56 | 0.09 | 0.31 | ARPC3; ARPC4; |
| Axon guidance | 17789 | REACTOME | 9606 | 6 | 336 | 0.09 | 0.31 | ACTR3; ARPC3; ARPC4; CLTCL1; COL5A1; EFNB3; |
| Hemostasis | 19856 | REACTOME | 9606 | 8 | 508 | 0.09 | 0.31 | ATP2A2; CAPZB; CD84; CFD; GNAQ; GP1BA; PDE5A; PDGFB; |
| Staphylococcus aureus infection | 10357 | KEGG | 9606 | 2 | 53 | 0.08 | 0.31 | CFD; FCGR1A; |
| Extracellular matrix organization | 17095 | REACTOME | 9606 | 5 | 266 | 0.10 | 0.32 | ADAM9; COL4A6; COL5A1; CTSL1; PDGFB; |
| Calcium signalling pathway | 594 | KEGG | 9606 | 4 | 183 | 0.09 | 0.32 | ATP2A2; GNAQ; GRM5; PLCD3; |
| Salivary secretion | 10375 | KEGG | 9606 | 2 | 91 | 0.20 | 0.32 | CST1; GNAQ; |
| Cell junction organization | 17130 | REACTOME | 9606 | 2 | 87 | 0.19 | 0.32 | CDH7; CDH8; |
| Glycogen storage diseases | 19851 | REACTOME | 9606 | 4 | 253 | 0.20 | 0.32 | B3GALT6; B3GNT1; HS6ST2; NUP62; |
| Metabolism of carbohydrates | 17445 | REACTOME | 9606 | 4 | 253 | 0.20 | 0.32 | B3GALT6; B3GNT1; HS6ST2; NUP62; |
| Myoclonic epilepsy of Lafora | 17077 | REACTOME | 9606 | 4 | 253 | 0.20 | 0.32 | B3GALT6; B3GNT1; HS6ST2; NUP62; |
| Dilated cardiomyopathy | 5714 | KEGG | 9606 | 2 | 90 | 0.20 | 0.33 | ATP2A2; DES; |
| PCP/CE pathway | 16659 | REACTOME | 9606 | 2 | 90 | 0.20 | 0.33 | FZD2; PSMA3; |
| mRNA surveillance pathway | 10376 | KEGG | 9606 | 2 | 90 | 0.20 | 0.33 | CPSF2; PPP2R3C; |
| Alzheimer's disease | 521 | KEGG | 9606 | 3 | 172 | 0.21 | 0.33 | ATP2A2; COX7C; GNAQ; |
| Protein digestion and absorption | 10383 | KEGG | 9606 | 2 | 89 | 0.20 | 0.33 | COL4A6; COL5A1; |
| Purine metabolism | 503 | KEGG | 9606 | 3 | 174 | 0.21 | 0.34 | GMPR2; PDE5A; POLR3H; |
| O-linked glycosylation | 18011 | REACTOME | 9606 | 2 | 96 | 0.22 | 0.34 | GALNT10; GCNT4; |
| Pancreatic secretion | 10370 | KEGG | 9606 | 2 | 96 | 0.22 | 0.34 | ATP2A2; GNAQ; |
| Gene Expression | 19770 | REACTOME | 9606 | 13 | 1118 | 0.22 | 0.34 | CPSF2; CWC15; EIF2B2; EIF3I; IARS; NUP62; POLR3H; PSMA3; SMC1A; SMG8; SRPRB; SSRP1; ZNF772; |
| Purine nucleotides nucleosides metabolism | 16184 | INOH | 9606 | 2 | 102 | 0.24 | 0.36 | GMPR2; PDE5A; |
| RNA Polymerase II Transcription | 18434 | REACTOME | 9606 | 2 | 102 | 0.24 | 0.36 | CPSF2; SSRP1; |
| Melanogenesis | 595 | KEGG | 9606 | 2 | 101 | 0.24 | 0.36 | FZD2; GNAQ; |
| G alpha (q) signalling events | 13217 | REACTOME | 9606 | 3 | 186 | 0.24 | 0.36 | GNAQ; GRM5; PROKR2; |
| Cap-dependent Translation Initiation | 18804 | REACTOME | 9606 | 2 | 112 | 0.27 | 0.39 | EIF2B2; EIF3I; |
| Eukaryotic Translation Initiation | 16696 | REACTOME | 9606 | 2 | 112 | 0.27 | 0.39 | EIF2B2; EIF3I; |
| Hedgehog 'off' state | 18085 | REACTOME | 9606 | 2 | 112 | 0.27 | 0.39 | FUZ; PSMA3; |
| RNF mutants show enhanced WNT signalling and proliferation | 19131 | REACTOME | 9606 | 3 | 197 | 0.27 | 0.39 | ASH2L; FZD2; PSMA3; |
| TCF dependent signalling in response to WNT | 18732 | REACTOME | 9606 | 3 | 197 | 0.27 | 0.39 | ASH2L; FZD2; PSMA3; |
| XAV939 inhibits tankyrase, stabilizing AXIN | 19487 | REACTOME | 9606 | 3 | 197 | 0.27 | 0.39 | ASH2L; FZD2; PSMA3; |
| Misspliced LRP5 mutants have enhanced beta-catenin-dependent signalling | 16943 | REACTOME | 9606 | 3 | 197 | 0.27 | 0.39 | ASH2L; FZD2; PSMA3; |
| Antigen processing: Ubiquitination & Proteasome degradation | 12981 | REACTOME | 9606 | 3 | 212 | 0.31 | 0.42 | PSMA3; UBA1; UBR4; |
| Gastrin-CREB signalling pathway via PKC and MAPK | 13219 | REACTOME | 9606 | 3 | 212 | 0.31 | 0.42 | GNAQ; GRM5; PROKR2; |
| Focal adhesion | 546 | KEGG | 9606 | 3 | 210 | 0.30 | 0.42 | COL4A6; COL5A1; PDGFB; |
| Developmental Biology | 16794 | REACTOME | 9606 | 6 | 502 | 0.31 | 0.42 | ACTR3; ARPC3; ARPC4; CLTCL1; COL5A1; EFNB3; |
| Adaptive Immune System | 18371 | REACTOME | 9606 | 7 | 604 | 0.31 | 0.42 | CTSL1; FCGR1A; FCGR1B; PDGFB; PSMA3; UBA1; UBR4; |
| Host Interactions of HIV factors | 19514 | REACTOME | 9606 | 2 | 121 | 0.30 | 0.42 | NUP62; PSMA3; |
| TGF-beta signalling | 16255 | INOH | 9606 | 2 | 120 | 0.30 | 0.42 | GDF7; PSMA3; |
| S Phase | 17420 | REACTOME | 9606 | 2 | 124 | 0.31 | 0.42 | PSMA3; SMC1A; |
| Integrin signalling pathway | 16138 | INOH | 9606 | 2 | 126 | 0.32 | 0.43 | COL4A6; COL5A1; |
| Axon guidance | 494 | KEGG | 9606 | 2 | 128 | 0.33 | 0.43 | EFNB3; LRRC4C; |
| Signalling by Hedgehog | 16836 | REACTOME | 9606 | 2 | 128 | 0.33 | 0.43 | FUZ; PSMA3; |
| Signalling by WNT in cancer | 17000 | REACTOME | 9606 | 3 | 219 | 0.32 | 0.43 | ASH2L; FZD2; PSMA3; |
| Cell-Cell communication | 17071 | REACTOME | 9606 | 2 | 132 | 0.34 | 0.44 | CDH7; CDH8; |
| Spliceosome | 8120 | KEGG | 9606 | 2 | 133 | 0.34 | 0.44 | CWC15; DDX46; |
| Oxidative phosphorylation | 576 | KEGG | 9606 | 2 | 137 | 0.36 | 0.45 | COX7C; LHPP; |
| Ribosome | 474 | KEGG | 9606 | 2 | 137 | 0.36 | 0.45 | MRPL20; MRPL27; |
| Toll-Like Receptors Cascades | 17767 | REACTOME | 9606 | 2 | 140 | 0.37 | 0.46 | CTSL1; TLR7; |
| Ubiquitin mediated proteolysis | 519 | KEGG | 9606 | 2 | 140 | 0.37 | 0.46 | PIAS1; UBA1; |
| Beta-catenin independent WNT signalling | 17081 | REACTOME | 9606 | 2 | 143 | 0.38 | 0.47 | FZD2; PSMA3; |
