# Supplementary material for: A Genome-Wide Knockout Screen in Human Macrophages Identified Host Factors Modulating Salmonella Infection
Source: mBio. 2019 Oct 8;10(5):e02169-19. doi: 10.1128/mBio.02169-19 (PMC6786873; doi:10.1128/mBio.02169-19)
Supplement: TABLE S3 [file mBio.02169-19-st003.docx]

| **Supplementary Table 3A: List of selected candidate genes and gRNA sequences chosen for validation.**  **Supplementary Table 3B: Percent relative uptake of *Salmonella* for mutant vs. WT control.**  **Table S3A: List of selected candidate genes and gRNA sequences chosen for validation.** | | | |
| --- | --- | --- | --- |
|  |  |  |  |
| **Gene** | **Ensembl ID** | **gRNA (5’-3’)** | **CRISPR ID** |
| **ACTR3** | **ENSG00000115091** | TATACAAAACTAGGATATGC | 936602624 |
|  |  | TTTTCTGGAGTATTCAATGG | 936604121 |
|  |  | TGTCAGACGTCCTCTCTACA | HGLibA_00658 |
|  |  | AAGTGGGTGATCAAGCTCAA | HGLibA_00659 |
|  |  | CACTAGAATATTGTCCTCTC | HGLibA_00660 |
| **ARPC4** | **ENSG00000241553** | TCATGAAGCGCATGAACTTG | 950022976 |
|  |  | GATGTACAAACACAAGTTGG | 950023298 |
|  |  | GCCCCTACCTGAGTGCCGTG | 950022479 |
|  |  | TACAACCTGTGACCATCAGC | 950022778 |
|  |  | CAATGCCCGTGCCCGCATTG | HGLibA_03087 |
| **ATP2A2** | **ENSG00000174437** | GAACGCGCACACCAAGACGG | 1102101527 |
|  |  | ATTAGTAGCCAATGCAATTG | 1102102595 |
|  |  | CTCAATCACAAGTTCCAGCA | HGLibA_03607 |
|  |  | CCATACACCCACAATTGCAT | HGLibA_03608 |
|  |  | ACAGAGTTACCGGCTGAAGA | HGLibA_03609 |
| **NHLRC2** | **ENSG00000196865** | TCAGTACCCGAGTTTCCGGA | 1074483980 |
|  |  | GGAGCAGTGAAGCACCTCGT | 1074487616 |
|  |  | AAACAATGCCTATCCTCACA | 1074487599 |
|  |  | TGTAAATTTCAGGATTAGAA | 1074484332 |
|  |  | TGCATCATTAACCATAGGGT | 1074485710 |
| **CLTCL1** | **ENSG00000070371** | AAGTGCTCCTGAAAGCGAAC | 1180891393 |
|  |  | GGCCGTCGGATCGGAGCCAT | 1180889456 |
|  |  | TTACGAGGAGGGAATGTACG | 1180880655 |
|  |  | TTCCGCTTCGCACAGCTGTG | 1180880454 |
|  |  | TTTGCTGTACGTAATCCCAC | HGLibA_10153 |
| **EFNB3** | **ENSG00000108947** | GCCCCGACTCGCACGCCCCC | 1143562881 |
|  |  | TTCGCCGAGTTCCAGTAGAC | 1143562898 |
|  |  | TCTAGCCGGTCCCCGATCTG | 1143563366 |
|  |  | CACATCGGATGGGACCCGGG | 1143563471 |
|  |  | CTCCCGGGTCCCATCCGATG | HGLibA_14604 |
| **PDGFB** | **ENSG00000100311** | CATCAAAGGAGCGGATCGAG | 1183663782 |
|  |  | GGTCTCCGTGCAGCAGGCGT | 1183663778 |
|  |  | CTCCAGCTCGCCTCCAGAGT | 1183663181 |
|  |  | ATGACCCGCTCCCACTCTGG | 1183663185 |
|  |  | GAGTCGGCATGAATCGCTGC | HGLibA_35843 |
| **HMGCR** | **ENSG00000113161** | TCAAGACTTTTTCGAATGCA | 992297704 |
|  |  | ATCCAAGTTGACGTAAATTC | 992297845 |
|  |  | TCTATCCAATTTATAGCAGC | 992299036 |
|  |  | ATGCAGATTCTAGCCGTTAG | HGLibA_21707 |
|  |  | TTCCAGAATTTACGTCAACT | HGLibA_21708 |
| **CD27** | **ENSG00000139193** | GAGCCCAGTAGTGCCTCTCT | 1092180759 |
|  |  | GTGTGATCCTTGCATACCGG | 1092180816 |
|  |  | ACGACCGAGCGGTCAGCGAA | 1092181543 |
|  |  | GCACTGCCAGCCATTGCGAC | HGLibA_08314 |
|  |  | CCCAGGAACATTCCTCGTGA | HGLibA_08315 |
| **CYFIP2** | **ENSG00000055163** | ATCTTCCAGGGTGACGTGCG TGG | 1000016820 |
|  |  | CATTGCAAGGTACATTGAGC AGG | 1000017016 |
|  |  | CTCATAGATCTCTACTCGGT TGG | 1000018176 |
|  |  | CTATCTGCATGTCGCCGAAA | HGLibA_12033 |
|  |  | CACCTACCACCTCCATGACG | HGLibA_12035 |
| **NPC1** | **ENSG00000141458** | GCGCTGGACACAGTAGCAGC | 1155209164 |
|  |  | AGGTACAATTGCGAATATTC | 1155207510 |
|  |  | CGCGATATTTCTGGTGACCA | 1155203229 |
|  |  | GAGAAATATTAATCCGTGAG | 1155202920 |
|  |  | AAAGAGTTACAATACTACGT | HGLibA_32418 |
| **B3GNT1** | **ENSG00000174684** | CGAAAGCGCTCGTCGAAGGT | 1084010619 |
|  |  | GAAGGCGCACCGGATGGCGT | 1084010833 |
|  |  | GGTCCTGAACGAAGGTTTCT | 1084010550 |
|  |  | CAGCAGCGTCGGGGAGAGTT | 1084010534 |
|  |  | GAACCAAGAAACCTTCGTTC | HGLibA_03982 |
| **FGD1** | **ENSG00000102302** | TGTCCGAGGGTCCGACAAAC | 1190881410 |
|  |  | GCATGGCCACCGAGCCCCGG | 1190881463 |
|  |  | CGAAGCCGCTGGGGACCTTC | 1190877719 |
|  |  | GCCGACCCCCGAGTGGCCAA | 1190877572 |
|  |  | CTATCCGAGGCGACAATCAC | 1190877520 |
| **CHRM2** | **ENSG00000181072** | GACTAGGATGTTCCCGATAA | 1032959459 |
|  |  | GTAACCAATCACAGTGTAGA | 1032959474 |
|  |  | CGCTTGACTGGGTAGGTCAG | 1032959490 |
|  |  | GGCTGCAATAGCCGTACCAA | 1032959519 |
|  |  | TATAAGGACTTGTAAGAGCC | 1032959447 |
| **ITPR3** | **ENSG00000096433** | GAAGCCATTGACGGAGCCCT | 1006366993 |
|  |  | GTCCAGCTTTCTTCACATCG | 1006366986 |
|  |  | GCTGGTGGATGACCGCTGTG | 1006370368 |
|  |  | ACTTCTTAGGGGGGTTGTCC | 1006370379 |
|  |  | GGGCCGAGTAGCGGTTCATG | 1006372801 |
| **CTTN** | **ENSG00000085733** | CCCCGCGTCATCCTGGGCGA | 1084638179 |
|  |  | CCTGGGCGATGGACACAGCG | 1084638175 |
|  |  | ATGGGGTGCCAAGACGGTGC | 1084638214 |
|  |  | TCAAGAGCATCAGACCCTTA | 1084638499 |
|  |  | CCGCTCCTTGGCCATCCGCT | 1084641475 |
| **TLR2** | **ENSG00000137462** | TACAAGTTCCGTGGAATGTT | 982282872 |
|  |  | CACCGATGAAAGTTTGTTTC | 982282878 |
|  |  | GTTAACGTTTCCACTTTACC | 982282887 |
|  |  | GACCGCAATGGTATCTGCAA | 982282819 |
|  |  | TCTTTAAACTCCATTCCCTC | 982282822 |
| **TOR3A** | **ENSG00000186283** | GCGCCACGGACCGCGAAGCA | 917853125 |
|  |  | TCTGTTGGGCCAGCGGTACC | 917853325 |
|  |  | GGCCATGCAGCCGCACATTC | 917853745 |
|  |  | CCGCTTCATCGAAGATGAAC | HGLibA_51029 |
|  |  | TTGGGCCACACTTAGAACGC | HGLibA_51030 |
| **CAPZB** | **ENSG00000077549** | CAGGCGCCTCATTAGGTCCA | 902887884 |
|  |  | TGAGGTTTTTCTCGATTTGC | 902887878 |
|  |  | CAGGAGATCCTCACATAGAC | 902883061 |
|  |  | GGAGATCCTCACATAGACTG | 902883063 |
|  |  | CCAGGTCCCCAGTCTATGTG | HGLibA_07215 |

| **Table S3B: Percent relative uptake of *Salmonella* for mutant vs. WT control.** | | | |
| --- | --- | --- | --- |
|  |  |  |  |
| **Gene** | **gRNA (5’-3’)** | **Well in 96 well plate** | **Relative uptake of *Salmonella*  for mutant vs. WT control (%)** |
| **ACTR3** | TATACAAAACTAGGATATGC | A1 | 16.3 ± 5.3 |
|  | TTTTCTGGAGTATTCAATGG | A2 | 47.4 ±2.4 |
|  | TGTCAGACGTCCTCTCTACA | A3 | unable to differentiate into macrophages |
|  | AAGTGGGTGATCAAGCTCAA | A4 | 54.8 ±6.2 |
|  | CACTAGAATATTGTCCTCTC | A5 | 27.0 ±3.1 |
| **ARPC4** | TCATGAAGCGCATGAACTTG | A6 | 29.2 ±2.4 |
|  | GATGTACAAACACAAGTTGG | A7 | 38.4 ±3.9 |
|  | GCCCCTACCTGAGTGCCGTG | A8 | 22.3 ±3.7 |
|  | TACAACCTGTGACCATCAGC | A9 | 30.5 ±2.0 |
|  | CAATGCCCGTGCCCGCATTG | A10 | 17.8 ±2.7 |
| **ATP2A2** | GAACGCGCACACCAAGACGG | A11 | 65.3 ±6.9 |
|  | ATTAGTAGCCAATGCAATTG | A12 | 62.3 ±2.9 |
|  | CTCAATCACAAGTTCCAGCA | B1 | 53.5 ±3.4 |
|  | CCATACACCCACAATTGCAT | B2 | 59.6 ±5.7 |
|  | ACAGAGTTACCGGCTGAAGA | B3 | 51.0 ±3.9 |
| **CYFIP2** | ATCTTCCAGGGTGACGTGCG | B4 | 75.1 ± 3.9 |
|  | CATTGCAAGGTACATTGAGC | B5 | 76.4 ±6.7 |
|  | CTCATAGATCTCTACTCGGT | B6 | 73.3 ±2.0 |
|  | CTATCTGCATGTCGCCGAAA | B7 | 74.6 ±1.7 |
|  | CACCTACCACCTCCATGACG | B8 | 70.2 ±5.3 |
| **CLTCL1** | AAGTGCTCCTGAAAGCGAAC | B9 | 29.5 ±3.4 |
|  | GGCCGTCGGATCGGAGCCAT | B10 | 21.2 ± 1.9 |
|  | TTACGAGGAGGGAATGTACG | B11 | 47.6 ±3.5 |
|  | TTCCGCTTCGCACAGCTGTG | B12 | 56.5 ±4.7 |
|  | TTTGCTGTACGTAATCCCAC | C1 | 57.7 ±4.9 |
| **EFNB3** | GCCCCGACTCGCACGCCCCC | C2 | 80.6 ±4.2 |
|  | TTCGCCGAGTTCCAGTAGAC | C3 | 78.4 ±5.6 |
|  | TCTAGCCGGTCCCCGATCTG | C4 | 76.0 ±2.3 |
|  | CACATCGGATGGGACCCGGG | C5 | 76.5 ±6.7 |
|  | CTCCCGGGTCCCATCCGATG | C6 | 78.1 ±5.5 |
| **PDGFB** | CATCAAAGGAGCGGATCGAG | C7 | 59.5 ±3.4 |
|  | GGTCTCCGTGCAGCAGGCGT | C8 | 63.0 ±2.9 |
|  | CTCCAGCTCGCCTCCAGAGT | C9 | 58.5 ±5.7 |
|  | ATGACCCGCTCCCACTCTGG | C10 | 56.2 ±4.1 |
|  | GAGTCGGCATGAATCGCTGC | C11 | 58.5 ±3.8 |
| **HMGCR** | TCAAGACTTTTTCGAATGCA | C12 | 47.8 ±3.6 |
|  | ATCCAAGTTGACGTAAATTC | D1 | 48.4 ±4.4 |
|  | TCTATCCAATTTATAGCAGC | D2 | 44.5 ±5.4 |
|  | ATGCAGATTCTAGCCGTTAG | D3 | 43.7 ±1.7 |
|  | TTCCAGAATTTACGTCAACT | D4 | 49.0 ± 3.0 |
| **CD27** | GAGCCCAGTAGTGCCTCTCT | D5 | 71.8 ±1.3 |
|  | GTGTGATCCTTGCATACCGG | D6 | 72.2 ±1.9 |
|  | ACGACCGAGCGGTCAGCGAA | D7 | 72.8 ±1.7 |
|  | GCACTGCCAGCCATTGCGAC | D8 | 67.1 ±1.2 |
|  | CCCAGGAACATTCCTCGTGA | D9 | 70.3 ±6.8 |
| **NHLRC2** | TCAGTACCCGAGTTTCCGGA | D10 | 28.1 ±3.9 |
|  | GGAGCAGTGAAGCACCTCGT | D11 | 38.0 ± 4.7 |
|  | AAACAATGCCTATCCTCACA | D12 | 32.2 ± 2.0 |
|  | TGTAAATTTCAGGATTAGAA | E1 | 25.6 ±1.8 |
|  | TGCATCATTAACCATAGGGT | E2 | 37.8 ± 5.6 |
| **NPC1** | GCGCTGGACACAGTAGCAGC | E3 | 75.1 ±2.3 |
|  | AGGTACAATTGCGAATATTC | E4 | 75.7 ±4.3 |
|  | CGCGATATTTCTGGTGACCA | E5 | 76.8 ±4.4 |
|  | GAGAAATATTAATCCGTGAG | E6 | 74.8 ±3.1 |
|  | AAAGAGTTACAATACTACGT | E7 | 69.7 ±7.8 |
| **B3GNT1** | CGAAAGCGCTCGTCGAAGGT | E8 | 31.9 ±6.7 |
|  | GAAGGCGCACCGGATGGCGT | E9 | 23.3 ±4.5 |
|  | GGTCCTGAACGAAGGTTTCT | E10 | 26.3 ±6.7 |
|  | CAGCAGCGTCGGGGAGAGTT | E11 | 21.2 ±4.9 |
|  | GAACCAAGAAACCTTCGTTC | E12 | 25.8 ±4.0 |
| **FGD1** | TGTCCGAGGGTCCGACAAAC | F1 | 73.2 ±6.7 |
|  | GCATGGCCACCGAGCCCCGG | F2 | 82.5 ±5.6 |
|  | CGAAGCCGCTGGGGACCTTC | F3 | 81.2 ±2.0 |
|  | GCCGACCCCCGAGTGGCCAA | F4 | 82.5 ±8.9 |
|  | CTATCCGAGGCGACAATCAC | F5 | 76.9 ±6.5 |
| **CHRM2** | GACTAGGATGTTCCCGATAA | F6 | 76.8 ±3.5 |
|  | GTAACCAATCACAGTGTAGA | F7 | 74.8 ±4.9 |
|  | CGCTTGACTGGGTAGGTCAG | F8 | 83.9 ±4.4 |
|  | GGCTGCAATAGCCGTACCAA | F9 | 86.2 ±3.0 |
|  | TATAAGGACTTGTAAGAGCC | F10 | 83.4 ±8.1 |
| **ITPR3** | GAAGCCATTGACGGAGCCCT | F11 | 27.1 ±3.1 |
|  | GTCCAGCTTTCTTCACATCG | F12 | 34.8 ±5.1 |
|  | GCTGGTGGATGACCGCTGTG | G1 | 32.4 ±3.7 |
|  | ACTTCTTAGGGGGGTTGTCC | G2 | 28.4 ±2.4 |
|  | GGGCCGAGTAGCGGTTCATG | G3 | 33.3 ±6.9 |
| **CTTN** | CCCCGCGTCATCCTGGGCGA | G4 | 52.4 ±7.1 |
|  | CCTGGGCGATGGACACAGCG | G5 | 54.2 ±2.4 |
|  | ATGGGGTGCCAAGACGGTGC | G6 | 52.6 ±2.6 |
|  | TCAAGAGCATCAGACCCTTA | G7 | 54.2 ±3.1 |
|  | CCGCTCCTTGGCCATCCGCT | G8 | 41.7 ±3.7 |
| **TLR2** | TACAAGTTCCGTGGAATGTT | G9 | 85.1 ±2.9 |
|  | CACCGATGAAAGTTTGTTTC | G10 | 83.0 ±6.3 |
|  | GTTAACGTTTCCACTTTACC | G11 | 84.8 ±4.0 |
|  | GACCGCAATGGTATCTGCAA | G12 | 79.4 ±5.6 |
|  | TCTTTAAACTCCATTCCCTC | H1 | 79.4 ±2.7 |
| **TOR3A** | GCGCCACGGACCGCGAAGCA | H2 | 50.9 ±1.7 |
|  | TCTGTTGGGCCAGCGGTACC | H3 | 49.4 ±4.9 |
|  | GGCCATGCAGCCGCACATTC | H4 | 51.0 ±2.3 |
|  | CCGCTTCATCGAAGATGAAC | H5 | 25.4 ±3.9 |
|  | TTGGGCCACACTTAGAACGC | H6 | 28.8 ±3.3 |
| **CAPZB** | CAGGCGCCTCATTAGGTCCA | H7 | 72.6 ±2.1 |
|  | TGAGGTTTTTCTCGATTTGC | H8 | 33.9 ±5.7 |
|  | CAGGAGATCCTCACATAGAC | H9 | 30.1 ±2.7 |
|  | GGAGATCCTCACATAGACTG | H10 | 40.2 ±6.9 |
|  | CCAGGTCCCCAGTCTATGTG | H11 | 47.9 ±4.7 |

**Table S3C: Zygosity of selected clonal mutants as determined by MiSeq.**

| **Gene** | **Mutant clone ID** | **Zygosity** |
| --- | --- | --- |
| **ACTR3** | A5_E4 | compound heterozygous |
|  | A5_G7 | compound heterozygous |
| **ARPC4** | A10_D4 | compound heterozygous |
|  | A10_G3 | compound heterozygous |
| **NHLRC2** | E1_D5 | homozygous |
|  | E1_E9 | homozygous |
| **B3GNT1** | E11_G10 | compound heterozygous |
|  | E11_C7 | compound heterozygous |
| **CLTCL1** | B10_F10 | compound heterozygous |
|  | B10_D1 | compound heterozygous |
| **PDGFB** | C10_B3 | homozygous |
|  | C10_B12 | homozygous |
| **TOR3A** | H5_D3 | compound heterozygous |
|  | H5_E10 | compound heterozygous |
| **CAPZB** | H9_G9 | heterozygous |
|  | H9_C9 | heterozygous |
| **CYFIP2** | B8_E4 | homozygous |
|  | B8_G3 | homozygous |
| **ITPR3** | F11_H12 | compound heterozygous |
|  | F11_H6 | compound heterozygous |
| **HMGCR** | D3_D7 | compound heterozygous |
|  | D3_F8 | compound heterozygous |
| **CTTN** | G8_B5 | compound heterozygous |
|  | G8_F7 | compound heterozygous |
| **ATP2A2** | B3_A2 | compound heterozygous |
|  | B3_D9 | compound heterozygous |
| **CD27** | D8_E5 | homozygous |
|  | D8_E11 | homozygous |
