# Supplementary material for: A Genome-Wide Knockout Screen in Human Macrophages Identified Host Factors Modulating Salmonella Infection
Source: mBio. 2019 Oct 8;10(5):e02169-19. doi: 10.1128/mBio.02169-19 (PMC6786873; doi:10.1128/mBio.02169-19)
Supplement: TABLE S4 [file mBio.02169-19-st004.docx]

**Supplementary Table 4A: List of over-represented pathways for downregulated genes using Sigora.**

**Supplementary Table 4B: List of over-represented pathways for upregulated genes using Sigora.**

**Supplementary Table 4C: List of dysregulated genes expressed in *NHLRC2* mutant vs. WT THP-1 macrophages based on RNA-Seq.** Only genes with fold changes ≤ or ≥ 1.5 and p-adjusted values < 0.01 are shown.

| **Table S4A: List of over-represented pathways for downregulated genes using Sigora.** | | | |
| --- | --- | --- | --- |
|  |  |  |  |
| **PathwayID** | **Description** | **pvalues** | **Bonferroni** |
| R-HSA-194315 | Signaling by Rho GTPases | 9.4E-164 | 9.73E-161 |
| R-HSA-194840 | Rho GTPase cycle | 1.83E-84 | 1.89E-81 |
| R-HSA-195258 | RHO GTPase Effectors | 1.21E-78 | 1.26E-75 |
| R-HSA-909733 | Interferon alpha/beta signaling | 1.16E-61 | 1.21E-58 |
| R-HSA-68877 | Mitotic Prometaphase | 6.51E-60 | 6.75E-57 |
| R-HSA-69620 | Cell Cycle Checkpoints | 2.64E-55 | 2.74E-52 |
| R-HSA-1474244 | Extracellular matrix organization | 2.36E-47 | 2.45E-44 |
| R-HSA-2467813 | Separation of Sister Chromatids | 2.32E-37 | 2.41E-34 |
| R-HSA-1474228 | Degradation of the extracellular matrix | 2.96E-37 | 3.07E-34 |
| R-HSA-5083635 | Defective B3GALTL causes Peters-plus syndrome (PpS) | 3.56E-35 | 3.70E-32 |
| R-HSA-176974 | Unwinding of DNA | 4.96E-35 | 5.15E-32 |
| R-HSA-114608 | Platelet degranulation | 4.25E-34 | 4.41E-31 |
| R-HSA-73894 | DNA Repair | 3.57E-33 | 3.70E-30 |
| R-HSA-69206 | G1/S Transition | 2.62E-31 | 2.72E-28 |
| R-HSA-5663220 | RHO GTPases Activate Formins | 1.80E-28 | 1.87E-25 |
| R-HSA-373076 | Class A/1 (Rhodopsin-like receptors) | 1.41E-27 | 1.46E-24 |
| R-HSA-983189 | Kinesins | 1.76E-27 | 1.83E-24 |
| R-HSA-8957275 | Post-translational protein phosphorylation | 5.05E-26 | 5.24E-23 |
| R-HSA-69190 | DNA strand elongation | 1.19E-24 | 1.23E-21 |
| R-HSA-196757 | Metabolism of folate and pterines | 1.26E-24 | 1.30E-21 |
| R-HSA-193648 | NRAGE signals death through JNK | 1.35E-21 | 1.40E-18 |
| R-HSA-69481 | G2/M Checkpoints | 1.52E-20 | 1.58E-17 |
| R-HSA-4420097 | VEGFA-VEGFR2 Pathway | 6.71E-20 | 6.96E-17 |
| R-HSA-69242 | S Phase | 1.12E-19 | 1.16E-16 |
| R-HSA-68886 | M Phase | 1.94E-19 | 2.01E-16 |
| R-HSA-499943 | Interconversion of nucleotide di- and triphosphates | 4.67E-19 | 4.85E-16 |
| R-HSA-77289 | Mitochondrial Fatty Acid Beta-Oxidation | 2.63E-17 | 2.73E-14 |
| R-HSA-216083 | Integrin cell surface interactions | 4.95E-17 | 5.13E-14 |
| R-HSA-913531 | Interferon Signaling | 1.35E-16 | 1.40E-13 |
| R-HSA-437239 | Recycling pathway of L1 | 1.41E-16 | 1.47E-13 |
| R-HSA-3299685 | Detoxification of Reactive Oxygen Species | 6.47E-16 | 6.71E-13 |
| R-HSA-196849 | Metabolism of water-soluble vitamins and cofactors | 1.45E-15 | 1.50E-12 |
| R-HSA-8978868 | Fatty acid metabolism | 5.75E-15 | 5.97E-12 |
| R-HSA-6783310 | Fanconi Anemia Pathway | 2.69E-14 | 2.79E-11 |
| R-HSA-73886 | Chromosome Maintenance | 5.57E-14 | 5.78E-11 |
| R-HSA-453279 | Mitotic G1-G1/S phases | 1.21E-13 | 1.25E-10 |
| R-HSA-156590 | Glutathione conjugation | 2.59E-13 | 2.69E-10 |
| R-HSA-69239 | Synthesis of DNA | 1.04E-12 | 1.08E-09 |
| R-HSA-9006934 | Signaling by Receptor Tyrosine Kinases | 5.57E-12 | 5.78E-09 |
| R-HSA-1660662 | Glycosphingolipid metabolism | 7.82E-12 | 8.12E-09 |
| R-HSA-2500257 | Resolution of Sister Chromatid Cohesion | 1.04E-11 | 1.08E-08 |
| R-HSA-2514853 | Condensation of Prometaphase Chromosomes | 1.05E-11 | 1.09E-08 |
| R-HSA-445355 | Smooth Muscle Contraction | 2.04E-11 | 2.12E-08 |
| R-HSA-8964043 | Plasma lipoprotein clearance | 1.21E-10 | 1.26E-07 |
| R-HSA-1660499 | Synthesis of PIPs at the plasma membrane | 3.06E-10 | 3.18E-07 |
| R-HSA-166016 | Toll Like Receptor 4 (TLR4) Cascade | 4.78E-10 | 4.96E-07 |
| R-HSA-5693538 | Homology Directed Repair | 5.81E-10 | 6.03E-07 |
| R-HSA-204998 | Cell death signalling via NRAGE, NRIF and NADE | 1.33E-09 | 1.38E-06 |
| R-HSA-211945 | Phase I - Functionalization of compounds | 1.89E-09 | 1.96E-06 |
| R-HSA-5625900 | RHO GTPases activate CIT | 2.28E-09 | 2.37E-06 |
| R-HSA-192105 | Synthesis of bile acids and bile salts | 3.31E-09 | 3.44E-06 |
| R-HSA-2022090 | Assembly of collagen fibrils and other multimeric structures | 3.58E-09 | 3.71E-06 |
| R-HSA-68882 | Mitotic Anaphase | 3.66E-09 | 3.80E-06 |
| R-HSA-1592389 | Activation of Matrix Metalloproteinases | 5.87E-09 | 6.10E-06 |
| R-HSA-912446 | Meiotic recombination | 5.92E-09 | 6.15E-06 |
| R-HSA-15869 | Metabolism of nucleotides | 1.41E-08 | 1.47E-05 |
| R-HSA-1236978 | Cross-presentation of soluble exogenous antigens (endosomes) | 1.88E-08 | 1.96E-05 |
| R-HSA-168898 | Toll-Like Receptors Cascades | 2.86E-08 | 2.97E-05 |
| R-HSA-8874081 | MET activates PTK2 signaling | 3.04E-08 | 3.15E-05 |
| R-HSA-390918 | Peroxisomal lipid metabolism | 9.93E-08 | 1.03E-04 |
| R-HSA-5656169 | Termination of translesion DNA synthesis | 1.14E-07 | 1.18E-04 |
| R-HSA-189445 | Metabolism of porphyrins | 1.16E-07 | 1.21E-04 |
| R-HSA-2022870 | Chondroitin sulfate biosynthesis | 1.52E-07 | 1.58E-04 |
| R-HSA-9033241 | Peroxisomal protein import | 2.36E-07 | 2.45E-04 |
| R-HSA-380108 | Chemokine receptors bind chemokines | 2.37E-07 | 2.46E-04 |
| R-HSA-1236975 | Antigen processing-Cross presentation | 3.27E-07 | 3.39E-04 |
| R-HSA-381426 | Regulation of Insulin-like Growth Factor (IGF) transport and uptake by Insulin-like Growth Factor Binding Proteins (IGFBPs) | 3.30E-07 | 3.43E-04 |
| R-HSA-76002 | Platelet activation, signaling and aggregation | 6.70E-07 | 6.96E-04 |
| R-HSA-5673001 | RAF/MAP kinase cascade | 7.70E-07 | 7.99E-04 |
| R-HSA-8948216 | Collagen chain trimerization | 1.09E-06 | 1.13E-03 |
| R-HSA-71406 | Pyruvate metabolism and Citric Acid (TCA) cycle | 1.62E-06 | 1.68E-03 |
| R-HSA-1483257 | Phospholipid metabolism | 2.32E-06 | 2.41E-03 |
| R-HSA-110314 | Recognition of DNA damage by PCNA-containing replication complex | 4.72E-06 | 4.90E-03 |
| R-HSA-69275 | G2/M Transition | 4.89E-06 | 5.07E-03 |
| R-HSA-70614 | Amino acid synthesis and interconversion (transamination) | 4.94E-06 | 5.12E-03 |

| **Table S4B: List of over-represented pathways for upregulated genes using Sigora.** | | |  |
| --- | --- | --- | --- |
|  |  |  |  |
| **PathwayID** | **Description** | **p values** | **Bonferroni** |
| R-HSA-9006934 | Signaling by Receptor Tyrosine Kinases | 1.87E-48 | 1.94E-45 |
| R-HSA-9020702 | Interleukin-1 signaling | 5.28E-38 | 5.48E-35 |
| R-HSA-6783783 | Interleukin-10 signaling | 7.21E-32 | 7.49E-29 |
| R-HSA-216083 | Integrin cell surface interactions | 9.70E-26 | 1.01E-22 |
| R-HSA-425393 | Transport of inorganic cations/anions and amino acids/oligopeptides | 1.42E-22 | 1.48E-19 |
| R-HSA-446652 | Interleukin-1 family signaling | 4.09E-20 | 4.25E-17 |
| R-HSA-8948216 | Collagen chain trimerization | 7.77E-20 | 8.07E-17 |
| R-HSA-6788467 | IL-6-type cytokine receptor ligand interactions | 3.12E-19 | 3.23E-16 |
| R-HSA-8957275 | Post-translational protein phosphorylation | 5.20E-18 | 5.39E-15 |
| R-HSA-5673001 | RAF/MAP kinase cascade | 1.55E-17 | 1.61E-14 |
| R-HSA-1632852 | Macroautophagy | 5.39E-17 | 5.60E-14 |
| R-HSA-416476 | G alpha (q) signalling events | 2.77E-16 | 2.87E-13 |
| R-HSA-201681 | TCF dependent signaling in response to WNT | 9.85E-15 | 1.02E-11 |
| R-HSA-373076 | Class A/1 (Rhodopsin-like receptors) | 1.07E-14 | 1.11E-11 |
| R-HSA-6785807 | Interleukin-4 and Interleukin-13 signaling | 5.13E-14 | 5.33E-11 |
| R-HSA-2122948 | Activated NOTCH1 Transmits Signal to the Nucleus | 1.23E-13 | 1.28E-10 |
| R-HSA-392154 | Nitric oxide stimulates guanylate cyclase | 1.27E-13 | 1.32E-10 |
| R-HSA-6811558 | PI5P, PP2A and IER3 Regulate PI3K/AKT Signaling | 5.42E-13 | 5.63E-10 |
| R-HSA-380108 | Chemokine receptors bind chemokines | 1.94E-12 | 2.01E-09 |
| R-HSA-418346 | Platelet homeostasis | 2.95E-12 | 3.06E-09 |
| R-HSA-381119 | Unfolded Protein Response (UPR) | 5.05E-12 | 5.25E-09 |
| R-HSA-5663202 | Diseases of signal transduction | 8.76E-12 | 9.09E-09 |
| R-HSA-1660499 | Synthesis of PIPs at the plasma membrane | 7.28E-11 | 7.56E-08 |
| R-HSA-373752 | Netrin-1 signaling | 2.21E-10 | 2.29E-07 |
| R-HSA-5578775 | Ion homeostasis | 2.22E-10 | 2.30E-07 |
| R-HSA-1650814 | Collagen biosynthesis and modifying enzymes | 2.39E-10 | 2.48E-07 |
| R-HSA-381340 | Transcriptional regulation of white adipocyte differentiation | 5.47E-10 | 5.68E-07 |
| R-HSA-983168 | Antigen processing: Ubiquitination & Proteasome degradation | 8.03E-10 | 8.33E-07 |
| R-HSA-936837 | Ion transport by P-type ATPases | 8.32E-10 | 8.64E-07 |
| R-HSA-195721 | Signaling by WNT | 9.52E-10 | 9.88E-07 |
| R-HSA-350054 | Notch-HLH transcription pathway | 9.53E-10 | 9.89E-07 |
| R-HSA-5661231 | Metallothioneins bind metals | 1.99E-09 | 2.06E-06 |
| R-HSA-4420097 | VEGFA-VEGFR2 Pathway | 2.45E-09 | 2.54E-06 |
| R-HSA-112316 | Neuronal System | 2.59E-09 | 2.68E-06 |
| R-HSA-425407 | SLC-mediated transmembrane transport | 3.72E-09 | 3.86E-06 |
| R-HSA-1474244 | Extracellular matrix organization | 3.88E-09 | 4.03E-06 |
| R-HSA-1912420 | Pre-NOTCH Processing in Golgi | 7.26E-09 | 7.53E-06 |
| R-HSA-8876198 | RAB GEFs exchange GTP for GDP on RABs | 1.70E-08 | 1.77E-05 |
| R-HSA-1368108 | BMAL1:CLOCK,NPAS2 activates circadian gene expression | 3.34E-08 | 3.46E-05 |
| R-HSA-391903 | Eicosanoid ligand-binding receptors | 3.69E-08 | 3.83E-05 |
| R-HSA-381038 | XBP1(S) activates chaperone genes | 4.51E-08 | 4.68E-05 |
| R-HSA-6804759 | Regulation of TP53 Activity through Association with Co-factors | 1.14E-07 | 1.19E-04 |
| R-HSA-5675221 | Negative regulation of MAPK pathway | 1.49E-07 | 1.54E-04 |
| R-HSA-71240 | Tryptophan catabolism | 1.55E-07 | 1.61E-04 |
| R-HSA-5676594 | TNF receptor superfamily (TNFSF) members mediating non-canonical NF-kB pathway | 3.04E-07 | 3.15E-04 |
| R-HSA-422356 | Regulation of insulin secretion | 4.67E-07 | 4.85E-04 |
| R-HSA-210500 | Glutamate Neurotransmitter Release Cycle | 4.73E-07 | 4.91E-04 |
| R-HSA-111885 | Opioid Signalling | 5.88E-07 | 6.10E-04 |
| R-HSA-2559582 | Senescence-Associated Secretory Phenotype (SASP) | 6.68E-07 | 6.94E-04 |
| R-HSA-2022090 | Assembly of collagen fibrils and other multimeric structures | 7.69E-07 | 7.98E-04 |
| R-HSA-388844 | Receptor-type tyrosine-protein phosphatases | 1.02E-06 | 1.06E-03 |
| R-HSA-2022928 | HS-GAG biosynthesis | 1.10E-06 | 1.14E-03 |
| R-HSA-2559583 | Cellular Senescence | 1.13E-06 | 1.17E-03 |
| R-HSA-6794361 | Neurexins and neuroligins | 1.17E-06 | 1.22E-03 |
| R-HSA-1442490 | Collagen degradation | 1.18E-06 | 1.23E-03 |
| R-HSA-166016 | Toll Like Receptor 4 (TLR4) Cascade | 1.30E-06 | 1.35E-03 |
| R-HSA-5660526 | Response to metal ions | 1.82E-06 | 1.89E-03 |
| R-HSA-4641263 | Regulation of FZD by ubiquitination | 3.69E-06 | 3.83E-03 |
| R-HSA-6804756 | Regulation of TP53 Activity through Phosphorylation | 4.97E-06 | 5.16E-03 |
| R-HSA-1433557 | Signaling by SCF-KIT | 5.44E-06 | 5.64E-03 |
| R-HSA-5218921 | VEGFR2 mediated cell proliferation | 9.24E-06 | 9.59E-03 |

| **Table S4C: List of dysregulated genes expressed in *NHLRC2* mutant vs. WT THP-1 macrophages based on RNA-Seq.**   \| **ENSEMBL** \| **SYMBOL** \| **log_2_ FC** \| **p value** \| **p adj** \| \| --- \| --- \| --- \| --- \| --- \| \| ENSG00000010610 \| CD4 \| -6.26 \| 0.00E+00 \| 0.00E+00 \| \| ENSG00000011422 \| PLAUR \| 1.56 \| 0.00E+00 \| 0.00E+00 \| \| ENSG00000012124 \| CD22 \| -3.10 \| 0.00E+00 \| 0.00E+00 \| \| ENSG00000018280 \| SLC11A1 \| 2.17 \| 0.00E+00 \| 0.00E+00 \| \| ENSG00000027697 \| IFNGR1 \| -1.59 \| 0.00E+00 \| 0.00E+00 \| \| ENSG00000028277 \| POU2F2 \| 2.03 \| 0.00E+00 \| 0.00E+00 \| \| ENSG00000030582 \| GRN \| -1.39 \| 0.00E+00 \| 0.00E+00 \| \| ENSG00000035403 \| VCL \| -2.46 \| 0.00E+00 \| 0.00E+00 \| \| ENSG00000035862 \| TIMP2 \| -2.27 \| 0.00E+00 \| 0.00E+00 \| \| ENSG00000039560 \| RAI14 \| 1.84 \| 0.00E+00 \| 0.00E+00 \| \| ENSG00000049089 \| COL9A2 \| -3.07 \| 0.00E+00 \| 0.00E+00 \| \| ENSG00000049759 \| NEDD4L \| 2.77 \| 0.00E+00 \| 0.00E+00 \| \| ENSG00000064195 \| DLX3 \| -2.85 \| 0.00E+00 \| 0.00E+00 \| \| ENSG00000064666 \| CNN2 \| -1.95 \| 0.00E+00 \| 0.00E+00 \| \| ENSG00000067057 \| PFKP \| -1.90 \| 0.00E+00 \| 0.00E+00 \| \| ENSG00000070540 \| WIPI1 \| 2.53 \| 0.00E+00 \| 0.00E+00 \| \| ENSG00000070614 \| NDST1 \| 1.44 \| 0.00E+00 \| 0.00E+00 \| \| ENSG00000070961 \| ATP2B1 \| 3.32 \| 0.00E+00 \| 0.00E+00 \| \| ENSG00000073060 \| SCARB1 \| -1.85 \| 0.00E+00 \| 0.00E+00 \| \| ENSG00000074416 \| MGLL \| 3.01 \| 0.00E+00 \| 0.00E+00 \| \| ENSG00000077420 \| APBB1IP \| -2.29 \| 0.00E+00 \| 0.00E+00 \| \| ENSG00000081237 \| PTPRC \| -1.87 \| 0.00E+00 \| 0.00E+00 \| \| ENSG00000085117 \| CD82 \| 2.56 \| 0.00E+00 \| 0.00E+00 \| \| ENSG00000090530 \| P3H2 \| 3.85 \| 0.00E+00 \| 0.00E+00 \| \| ENSG00000092068 \| SLC7A8 \| -2.26 \| 0.00E+00 \| 0.00E+00 \| \| ENSG00000101335 \| MYL9 \| -2.54 \| 0.00E+00 \| 0.00E+00 \| \| ENSG00000102265 \| TIMP1 \| 1.63 \| 0.00E+00 \| 0.00E+00 \| \| ENSG00000103489 \| XYLT1 \| 1.88 \| 0.00E+00 \| 0.00E+00 \| \| ENSG00000104419 \| NDRG1 \| 2.31 \| 0.00E+00 \| 0.00E+00 \| \| ENSG00000104549 \| SQLE \| 1.26 \| 0.00E+00 \| 0.00E+00 \| \| ENSG00000104635 \| SLC39A14 \| 2.43 \| 0.00E+00 \| 0.00E+00 \| \| ENSG00000104894 \| CD37 \| -2.46 \| 0.00E+00 \| 0.00E+00 \| \| ENSG00000105835 \| NAMPT \| 2.07 \| 0.00E+00 \| 0.00E+00 \| \| ENSG00000105976 \| MET \| 3.75 \| 0.00E+00 \| 0.00E+00 \| \| ENSG00000106211 \| HSPB1 \| -2.23 \| 0.00E+00 \| 0.00E+00 \| \| ENSG00000107798 \| LIPA \| -2.03 \| 0.00E+00 \| 0.00E+00 \| \| ENSG00000108932 \| SLC16A6 \| 3.29 \| 0.00E+00 \| 0.00E+00 \| \| ENSG00000109321 \| AREG \| 2.40 \| 0.00E+00 \| 0.00E+00 \| \| ENSG00000110092 \| CCND1 \| 1.38 \| 0.00E+00 \| 0.00E+00 \| \| ENSG00000111252 \| SH2B3 \| 1.27 \| 0.00E+00 \| 0.00E+00 \| \| ENSG00000112715 \| VEGFA \| 3.82 \| 0.00E+00 \| 0.00E+00 \| \| ENSG00000112773 \| TENT5A \| -3.12 \| 0.00E+00 \| 0.00E+00 \| \| ENSG00000113070 \| HBEGF \| 2.08 \| 0.00E+00 \| 0.00E+00 \| \| ENSG00000114268 \| PFKFB4 \| 1.72 \| 0.00E+00 \| 0.00E+00 \| \| ENSG00000114626 \| ABTB1 \| 1.77 \| 0.00E+00 \| 0.00E+00 \| \| ENSG00000115414 \| FN1 \| 3.89 \| 0.00E+00 \| 0.00E+00 \| \| ENSG00000115523 \| GNLY \| 5.78 \| 0.00E+00 \| 0.00E+00 \| \| ENSG00000115594 \| IL1R1 \| 2.14 \| 0.00E+00 \| 0.00E+00 \| \| ENSG00000115738 \| ID2 \| -1.51 \| 0.00E+00 \| 0.00E+00 \| \| ENSG00000115884 \| SDC1 \| 3.33 \| 0.00E+00 \| 0.00E+00 \| \| ENSG00000115919 \| KYNU \| 2.52 \| 0.00E+00 \| 0.00E+00 \| \| ENSG00000115963 \| RND3 \| 2.11 \| 0.00E+00 \| 0.00E+00 \| \| ENSG00000117399 \| CDC20 \| -2.90 \| 0.00E+00 \| 0.00E+00 \| \| ENSG00000117632 \| STMN1 \| -2.85 \| 0.00E+00 \| 0.00E+00 \| \| ENSG00000118985 \| ELL2 \| 1.79 \| 0.00E+00 \| 0.00E+00 \| \| ENSG00000120875 \| DUSP4 \| 1.93 \| 0.00E+00 \| 0.00E+00 \| \| ENSG00000122691 \| TWIST1 \| 2.39 \| 0.00E+00 \| 0.00E+00 \| \| ENSG00000123095 \| BHLHE41 \| 3.56 \| 0.00E+00 \| 0.00E+00 \| \| ENSG00000125430 \| HS3ST3B1 \| 3.17 \| 0.00E+00 \| 0.00E+00 \| \| ENSG00000125538 \| IL1B \| 4.43 \| 0.00E+00 \| 0.00E+00 \| \| ENSG00000125730 \| C3 \| 1.80 \| 0.00E+00 \| 0.00E+00 \| \| ENSG00000127920 \| GNG11 \| 3.47 \| 0.00E+00 \| 0.00E+00 \| \| ENSG00000130303 \| BST2 \| -3.41 \| 0.00E+00 \| 0.00E+00 \| \| ENSG00000130340 \| SNX9 \| 1.77 \| 0.00E+00 \| 0.00E+00 \| \| ENSG00000132182 \| NUP210 \| -1.56 \| 0.00E+00 \| 0.00E+00 \| \| ENSG00000132688 \| NES \| -2.14 \| 0.00E+00 \| 0.00E+00 \| \| ENSG00000132718 \| SYT11 \| 2.98 \| 0.00E+00 \| 0.00E+00 \| \| ENSG00000133961 \| NUMB \| 1.44 \| 0.00E+00 \| 0.00E+00 \| \| ENSG00000134871 \| COL4A2 \| -4.02 \| 0.00E+00 \| 0.00E+00 \| \| ENSG00000135218 \| CD36 \| -2.90 \| 0.00E+00 \| 0.00E+00 \| \| ENSG00000135318 \| NT5E \| 2.56 \| 0.00E+00 \| 0.00E+00 \| \| ENSG00000135678 \| CPM \| 2.59 \| 0.00E+00 \| 0.00E+00 \| \| ENSG00000136026 \| CKAP4 \| 2.63 \| 0.00E+00 \| 0.00E+00 \| \| ENSG00000136235 \| GPNMB \| -3.34 \| 0.00E+00 \| 0.00E+00 \| \| ENSG00000136689 \| IL1RN \| 1.92 \| 0.00E+00 \| 0.00E+00 \| \| ENSG00000137491 \| SLCO2B1 \| -3.45 \| 0.00E+00 \| 0.00E+00 \| \| ENSG00000137642 \| SORL1 \| -3.14 \| 0.00E+00 \| 0.00E+00 \| \| ENSG00000137801 \| THBS1 \| 2.31 \| 0.00E+00 \| 0.00E+00 \| \| ENSG00000137841 \| PLCB2 \| -1.97 \| 0.00E+00 \| 0.00E+00 \| \| ENSG00000138061 \| CYP1B1 \| 1.67 \| 0.00E+00 \| 0.00E+00 \| \| ENSG00000138623 \| SEMA7A \| 1.55 \| 0.00E+00 \| 0.00E+00 \| \| ENSG00000139679 \| LPAR6 \| -3.71 \| 0.00E+00 \| 0.00E+00 \| \| ENSG00000140379 \| BCL2A1 \| 2.61 \| 0.00E+00 \| 0.00E+00 \| \| ENSG00000141664 \| ZCCHC2 \| 2.06 \| 0.00E+00 \| 0.00E+00 \| \| ENSG00000141753 \| IGFBP4 \| -2.14 \| 0.00E+00 \| 0.00E+00 \| \| ENSG00000142156 \| COL6A1 \| 1.63 \| 0.00E+00 \| 0.00E+00 \| \| ENSG00000143369 \| ECM1 \| -2.17 \| 0.00E+00 \| 0.00E+00 \| \| ENSG00000143537 \| ADAM15 \| -1.83 \| 0.00E+00 \| 0.00E+00 \| \| ENSG00000143799 \| PARP1 \| -1.50 \| 0.00E+00 \| 0.00E+00 \| \| ENSG00000145040 \| UCN2 \| 3.22 \| 0.00E+00 \| 0.00E+00 \| \| ENSG00000145623 \| OSMR \| 3.21 \| 0.00E+00 \| 0.00E+00 \| \| ENSG00000145936 \| KCNMB1 \| -2.37 \| 0.00E+00 \| 0.00E+00 \| \| ENSG00000148773 \| MKI67 \| -2.84 \| 0.00E+00 \| 0.00E+00 \| \| ENSG00000149948 \| HMGA2 \| 1.70 \| 0.00E+00 \| 0.00E+00 \| \| ENSG00000150630 \| VEGFC \| 2.95 \| 0.00E+00 \| 0.00E+00 \| \| ENSG00000157168 \| NRG1 \| 2.67 \| 0.00E+00 \| 0.00E+00 \| \| ENSG00000159167 \| STC1 \| 5.55 \| 0.00E+00 \| 0.00E+00 \| \| ENSG00000159200 \| RCAN1 \| 1.62 \| 0.00E+00 \| 0.00E+00 \| \| ENSG00000159399 \| HK2 \| 2.11 \| 0.00E+00 \| 0.00E+00 \| \| ENSG00000160683 \| CXCR5 \| 3.26 \| 0.00E+00 \| 0.00E+00 \| \| ENSG00000164111 \| ANXA5 \| 1.47 \| 0.00E+00 \| 0.00E+00 \| \| ENSG00000164687 \| FABP5 \| -1.68 \| 0.00E+00 \| 0.00E+00 \| \| ENSG00000165168 \| CYBB \| -2.35 \| 0.00E+00 \| 0.00E+00 \| \| ENSG00000165617 \| DACT1 \| 3.62 \| 0.00E+00 \| 0.00E+00 \| \| ENSG00000166825 \| ANPEP \| 1.84 \| 0.00E+00 \| 0.00E+00 \| \| ENSG00000168389 \| MFSD2A \| 2.19 \| 0.00E+00 \| 0.00E+00 \| \| ENSG00000169116 \| PARM1 \| 2.38 \| 0.00E+00 \| 0.00E+00 \| \| ENSG00000169385 \| RNASE2 \| -3.70 \| 0.00E+00 \| 0.00E+00 \| \| ENSG00000169436 \| COL22A1 \| 2.39 \| 0.00E+00 \| 0.00E+00 \| \| ENSG00000169442 \| CD52 \| -3.37 \| 0.00E+00 \| 0.00E+00 \| \| ENSG00000169499 \| PLEKHA2 \| -2.41 \| 0.00E+00 \| 0.00E+00 \| \| ENSG00000169896 \| ITGAM \| -1.83 \| 0.00E+00 \| 0.00E+00 \| \| ENSG00000170017 \| ALCAM \| 1.58 \| 0.00E+00 \| 0.00E+00 \| \| ENSG00000170323 \| FABP4 \| -5.35 \| 0.00E+00 \| 0.00E+00 \| \| ENSG00000170873 \| MTSS1 \| 1.29 \| 0.00E+00 \| 0.00E+00 \| \| ENSG00000171115 \| GIMAP8 \| 2.75 \| 0.00E+00 \| 0.00E+00 \| \| ENSG00000172216 \| CEBPB \| 2.51 \| 0.00E+00 \| 0.00E+00 \| \| ENSG00000172232 \| AZU1 \| -6.27 \| 0.00E+00 \| 0.00E+00 \| \| ENSG00000174136 \| RGMB \| 2.79 \| 0.00E+00 \| 0.00E+00 \| \| ENSG00000176788 \| BASP1 \| 2.48 \| 0.00E+00 \| 0.00E+00 \| \| ENSG00000177666 \| PNPLA2 \| 1.86 \| 0.00E+00 \| 0.00E+00 \| \| ENSG00000177707 \| NECTIN3 \| 2.61 \| 0.00E+00 \| 0.00E+00 \| \| ENSG00000182179 \| UBA7 \| -3.14 \| 0.00E+00 \| 0.00E+00 \| \| ENSG00000182534 \| MXRA7 \| 2.45 \| 0.00E+00 \| 0.00E+00 \| \| ENSG00000182957 \| SPATA13 \| 3.13 \| 0.00E+00 \| 0.00E+00 \| \| ENSG00000183696 \| UPP1 \| 2.45 \| 0.00E+00 \| 0.00E+00 \| \| ENSG00000183762 \| KREMEN1 \| 2.19 \| 0.00E+00 \| 0.00E+00 \| \| ENSG00000184014 \| DENND5A \| 2.38 \| 0.00E+00 \| 0.00E+00 \| \| ENSG00000184226 \| PCDH9 \| 4.80 \| 0.00E+00 \| 0.00E+00 \| \| ENSG00000184371 \| CSF1 \| 2.66 \| 0.00E+00 \| 0.00E+00 \| \| ENSG00000187498 \| COL4A1 \| -4.05 \| 0.00E+00 \| 0.00E+00 \| \| ENSG00000196154 \| S100A4 \| -3.03 \| 0.00E+00 \| 0.00E+00 \| \| ENSG00000197043 \| ANXA6 \| -2.62 \| 0.00E+00 \| 0.00E+00 \| \| ENSG00000197405 \| C5AR1 \| 1.87 \| 0.00E+00 \| 0.00E+00 \| \| ENSG00000198959 \| TGM2 \| 2.34 \| 0.00E+00 \| 0.00E+00 \| \| ENSG00000205542 \| TMSB4X \| -1.65 \| 0.00E+00 \| 0.00E+00 \| \| ENSG00000211455 \| STK38L \| 2.40 \| 0.00E+00 \| 0.00E+00 \| \| ENSG00000213949 \| ITGA1 \| 3.21 \| 0.00E+00 \| 0.00E+00 \| \| ENSG00000221968 \| FADS3 \| 1.55 \| 0.00E+00 \| 0.00E+00 \| \| ENSG00000225697 \| SLC26A6 \| 1.79 \| 0.00E+00 \| 0.00E+00 \| \| ENSG00000227706 \|  \| -2.29 \| 0.00E+00 \| 0.00E+00 \| \| ENSG00000258183 \| LINC02392 \| 4.09 \| 0.00E+00 \| 0.00E+00 \| \| ENSG00000272405 \|  \| -3.35 \| 0.00E+00 \| 0.00E+00 \| \| ENSG00000135111 \| TBX3 \| 3.13 \| 8.48E-308 \| 8.91E-306 \| \| ENSG00000169429 \| CXCL8 \| 4.79 \| 1.88E-307 \| 1.96E-305 \| \| ENSG00000105223 \| PLD3 \| -1.66 \| 4.12E-306 \| 4.27E-304 \| \| ENSG00000127824 \| TUBA4A \| 1.47 \| 8.69E-306 \| 8.95E-304 \| \| ENSG00000182871 \| COL18A1 \| -3.10 \| 1.25E-304 \| 1.28E-302 \| \| ENSG00000135077 \| HAVCR2 \| -1.74 \| 2.69E-304 \| 2.73E-302 \| \| ENSG00000196924 \| FLNA \| -3.16 \| 7.80E-304 \| 7.87E-302 \| \| ENSG00000117318 \| ID3 \| -3.57 \| 3.20E-302 \| 3.21E-300 \| \| ENSG00000023892 \| DEF6 \| -2.74 \| 9.87E-302 \| 9.83E-300 \| \| ENSG00000122641 \| INHBA \| 5.52 \| 3.76E-301 \| 3.72E-299 \| \| ENSG00000136068 \| FLNB \| -2.63 \| 2.61E-300 \| 2.57E-298 \| \| ENSG00000107249 \| GLIS3 \| 1.58 \| 4.68E-300 \| 4.57E-298 \| \| ENSG00000168071 \| CCDC88B \| -3.18 \| 1.56E-297 \| 1.52E-295 \| \| ENSG00000113810 \| SMC4 \| -1.76 \| 5.01E-297 \| 4.83E-295 \| \| ENSG00000115009 \| CCL20 \| 4.72 \| 1.24E-296 \| 1.19E-294 \| \| ENSG00000118495 \| PLAGL1 \| 2.08 \| 2.60E-294 \| 2.48E-292 \| \| ENSG00000132386 \| SERPINF1 \| -3.82 \| 4.16E-293 \| 3.94E-291 \| \| ENSG00000132003 \| ZSWIM4 \| 1.40 \| 3.19E-292 \| 3.00E-290 \| \| ENSG00000173801 \| JUP \| -4.19 \| 1.00E-291 \| 9.39E-290 \| \| ENSG00000185551 \| NR2F2 \| 1.92 \| 2.24E-291 \| 2.08E-289 \| \| ENSG00000183023 \| SLC8A1 \| -2.04 \| 1.13E-289 \| 1.05E-287 \| \| ENSG00000145390 \| USP53 \| 2.05 \| 2.55E-286 \| 2.34E-284 \| \| ENSG00000151790 \| TDO2 \| 1.67 \| 5.07E-285 \| 4.63E-283 \| \| ENSG00000129667 \| RHBDF2 \| 1.14 \| 1.28E-284 \| 1.16E-282 \| \| ENSG00000173641 \| HSPB7 \| -3.45 \| 2.48E-284 \| 2.24E-282 \| \| ENSG00000151615 \| POU4F2 \| 2.25 \| 4.36E-284 \| 3.90E-282 \| \| ENSG00000186350 \| RXRA \| -1.45 \| 1.76E-283 \| 1.57E-281 \| \| ENSG00000101057 \| MYBL2 \| -2.30 \| 1.14E-282 \| 1.01E-280 \| \| ENSG00000153976 \| HS3ST3A1 \| 2.81 \| 2.38E-282 \| 2.09E-280 \| \| ENSG00000175274 \| TP53I11 \| -2.17 \| 1.00E-280 \| 8.79E-279 \| \| ENSG00000131747 \| TOP2A \| -2.70 \| 2.15E-280 \| 1.87E-278 \| \| ENSG00000178726 \| THBD \| 1.64 \| 3.00E-278 \| 2.60E-276 \| \| ENSG00000184489 \| PTP4A3 \| -2.15 \| 5.33E-278 \| 4.59E-276 \| \| ENSG00000154380 \| ENAH \| -2.15 \| 6.06E-276 \| 5.18E-274 \| \| ENSG00000169220 \| RGS14 \| -2.68 \| 9.60E-273 \| 8.16E-271 \| \| ENSG00000179855 \| GIPC3 \| -1.59 \| 1.35E-271 \| 1.14E-269 \| \| ENSG00000123329 \| ARHGAP9 \| -2.23 \| 4.51E-271 \| 3.79E-269 \| \| ENSG00000139641 \| ESYT1 \| -1.56 \| 1.00E-270 \| 8.37E-269 \| \| ENSG00000168734 \| PKIG \| 2.91 \| 3.44E-270 \| 2.86E-268 \| \| ENSG00000121281 \| ADCY7 \| -4.10 \| 3.03E-268 \| 2.50E-266 \| \| ENSG00000130203 \| APOE \| -1.95 \| 8.24E-267 \| 6.78E-265 \| \| ENSG00000105419 \| MEIS3 \| 3.17 \| 4.20E-266 \| 3.44E-264 \| \| ENSG00000174705 \| SH3PXD2B \| 1.66 \| 5.02E-265 \| 4.09E-263 \| \| ENSG00000156535 \| CD109 \| -1.57 \| 1.98E-263 \| 1.61E-261 \| \| ENSG00000135535 \| CD164 \| -1.23 \| 2.77E-263 \| 2.23E-261 \| \| ENSG00000161791 \| FMNL3 \| 1.28 \| 3.66E-263 \| 2.93E-261 \| \| ENSG00000088826 \| SMOX \| 1.40 \| 7.65E-263 \| 6.10E-261 \| \| ENSG00000128567 \| PODXL \| -1.96 \| 4.82E-262 \| 3.82E-260 \| \| ENSG00000014257 \| ACPP \| -2.39 \| 5.42E-262 \| 4.27E-260 \| \| ENSG00000163870 \| TPRA1 \| 1.44 \| 1.16E-261 \| 9.08E-260 \| \| ENSG00000114270 \| COL7A1 \| 1.91 \| 1.20E-261 \| 9.39E-260 \| \| ENSG00000101439 \| CST3 \| -1.44 \| 2.18E-260 \| 1.69E-258 \| \| ENSG00000123094 \| RASSF8 \| 2.23 \| 4.08E-259 \| 3.16E-257 \| \| ENSG00000152492 \| CCDC50 \| 1.69 \| 6.51E-259 \| 5.00E-257 \| \| ENSG00000129422 \| MTUS1 \| -2.68 \| 1.26E-258 \| 9.63E-257 \| \| ENSG00000235531 \| MSC-AS1 \| 1.89 \| 4.09E-258 \| 3.11E-256 \| \| ENSG00000043355 \| ZIC2 \| 1.71 \| 1.71E-257 \| 1.30E-255 \| \| ENSG00000131238 \| PPT1 \| -0.91 \| 1.02E-255 \| 7.71E-254 \| \| ENSG00000003402 \| CFLAR \| 1.64 \| 6.80E-255 \| 5.10E-253 \| \| ENSG00000153317 \| ASAP1 \| 1.22 \| 3.41E-253 \| 2.54E-251 \| \| ENSG00000026508 \| CD44 \| 1.17 \| 1.11E-252 \| 8.25E-251 \| \| ENSG00000142089 \| IFITM3 \| -2.46 \| 6.16E-251 \| 4.55E-249 \| \| ENSG00000169902 \| TPST1 \| 1.61 \| 3.98E-249 \| 2.92E-247 \| \| ENSG00000184584 \| TMEM173 \| -2.96 \| 2.41E-248 \| 1.76E-246 \| \| ENSG00000186907 \| RTN4RL2 \| 1.65 \| 2.69E-248 \| 1.96E-246 \| \| ENSG00000154734 \| ADAMTS1 \| 2.07 \| 3.01E-248 \| 2.18E-246 \| \| ENSG00000137309 \| HMGA1 \| 1.15 \| 1.98E-247 \| 1.43E-245 \| \| ENSG00000196405 \| EVL \| -1.86 \| 1.69E-246 \| 1.21E-244 \| \| ENSG00000164330 \| EBF1 \| 3.10 \| 1.37E-245 \| 9.77E-244 \| \| ENSG00000176170 \| SPHK1 \| 1.60 \| 3.44E-244 \| 2.44E-242 \| \| ENSG00000075223 \| SEMA3C \| 3.14 \| 7.53E-244 \| 5.33E-242 \| \| ENSG00000008083 \| JARID2 \| 1.35 \| 8.52E-244 \| 6.00E-242 \| \| ENSG00000133687 \| TMTC1 \| 1.49 \| 4.76E-243 \| 3.33E-241 \| \| ENSG00000112033 \| PPARD \| 1.24 \| 1.86E-242 \| 1.30E-240 \| \| ENSG00000205744 \| DENND1C \| -2.60 \| 2.53E-242 \| 1.76E-240 \| \| ENSG00000198561 \| CTNND1 \| -1.85 \| 4.60E-241 \| 3.18E-239 \| \| ENSG00000198561 \| TMX2-CTNND1 \| -1.85 \| 4.60E-241 \| 3.18E-239 \| \| ENSG00000135636 \| DYSF \| -4.23 \| 8.07E-240 \| 5.55E-238 \| \| ENSG00000198910 \| L1CAM \| 2.90 \| 1.53E-239 \| 1.05E-237 \| \| ENSG00000104267 \| CA2 \| -2.23 \| 3.56E-239 \| 2.43E-237 \| \| ENSG00000135363 \| LMO2 \| -2.30 \| 4.11E-239 \| 2.79E-237 \| \| ENSG00000111144 \| LTA4H \| 1.40 \| 6.17E-238 \| 4.17E-236 \| \| ENSG00000122574 \| WIPF3 \| -2.56 \| 1.51E-237 \| 1.02E-235 \| \| ENSG00000125384 \| PTGER2 \| 3.76 \| 1.56E-237 \| 1.05E-235 \| \| ENSG00000105974 \| CAV1 \| 2.36 \| 2.41E-237 \| 1.61E-235 \| \| ENSG00000123342 \| MMP19 \| 1.87 \| 2.58E-235 \| 1.71E-233 \| \| ENSG00000138131 \| LOXL4 \| -3.69 \| 5.37E-235 \| 3.55E-233 \| \| ENSG00000107819 \| SFXN3 \| -1.50 \| 1.53E-233 \| 1.01E-231 \| \| ENSG00000149557 \| FEZ1 \| 1.72 \| 1.56E-233 \| 1.03E-231 \| \| ENSG00000149557 \| STT3A-AS1 \| 1.72 \| 1.56E-233 \| 1.03E-231 \| \| ENSG00000134250 \| NOTCH2 \| 1.37 \| 4.98E-233 \| 3.25E-231 \| \| ENSG00000103942 \| HOMER2 \| -2.63 \| 8.99E-233 \| 5.84E-231 \| \| ENSG00000168461 \| RAB31 \| 1.05 \| 6.88E-231 \| 4.45E-229 \| \| ENSG00000185950 \| IRS2 \| 1.74 \| 3.21E-229 \| 2.07E-227 \| \| ENSG00000122952 \| ZWINT \| -2.79 \| 5.01E-229 \| 3.21E-227 \| \| ENSG00000185022 \| MAFF \| 1.88 \| 9.54E-229 \| 6.09E-227 \| \| ENSG00000117013 \| KCNQ4 \| -3.03 \| 3.59E-228 \| 2.28E-226 \| \| ENSG00000147443 \| DOK2 \| -2.02 \| 7.81E-227 \| 4.95E-225 \| \| ENSG00000127507 \| ADGRE2 \| 1.27 \| 4.23E-226 \| 2.67E-224 \| \| ENSG00000105612 \| DNASE2 \| -2.51 \| 2.32E-225 \| 1.46E-223 \| \| ENSG00000198853 \| RUSC2 \| 1.15 \| 2.34E-225 \| 1.46E-223 \| \| ENSG00000136826 \| KLF4 \| -2.15 \| 4.80E-225 \| 2.99E-223 \| \| ENSG00000108679 \| LGALS3BP \| -4.44 \| 1.77E-224 \| 1.10E-222 \| \| ENSG00000174718 \| KIAA1551 \| 1.37 \| 1.92E-224 \| 1.19E-222 \| \| ENSG00000130066 \| SAT1 \| 2.02 \| 7.27E-224 \| 4.47E-222 \| \| ENSG00000103066 \| PLA2G15 \| -1.45 \| 3.54E-223 \| 2.17E-221 \| \| ENSG00000143772 \| ITPKB \| -2.50 \| 3.79E-223 \| 2.31E-221 \| \| ENSG00000123146 \| ADGRE5 \| -2.11 \| 4.21E-223 \| 2.56E-221 \| \| ENSG00000141506 \| PIK3R5 \| 1.35 \| 4.51E-222 \| 2.73E-220 \| \| ENSG00000152127 \| MGAT5 \| -1.55 \| 6.23E-222 \| 3.76E-220 \| \| ENSG00000145715 \| RASA1 \| -1.28 \| 9.88E-222 \| 5.94E-220 \| \| ENSG00000138964 \| PARVG \| -1.45 \| 1.22E-221 \| 7.33E-220 \| \| ENSG00000110031 \| LPXN \| 1.64 \| 1.97E-220 \| 1.17E-218 \| \| ENSG00000114423 \| CBLB \| 1.80 \| 4.55E-220 \| 2.70E-218 \| \| ENSG00000182162 \| P2RY8 \| -1.81 \| 4.59E-220 \| 2.72E-218 \| \| ENSG00000005884 \| ITGA3 \| -1.40 \| 2.39E-219 \| 1.41E-217 \| \| ENSG00000197766 \| CFD \| -3.31 \| 3.24E-219 \| 1.90E-217 \| \| ENSG00000173846 \| PLK3 \| 1.64 \| 4.76E-219 \| 2.78E-217 \| \| ENSG00000185201 \| IFITM2 \| -1.97 \| 2.17E-218 \| 1.27E-216 \| \| ENSG00000182568 \| SATB1 \| -1.70 \| 1.44E-217 \| 8.38E-216 \| \| ENSG00000147257 \| GPC3 \| -3.62 \| 2.75E-216 \| 1.59E-214 \| \| ENSG00000068366 \| ACSL4 \| 1.33 \| 1.58E-215 \| 9.10E-214 \| \| ENSG00000266709 \| MGC12916 \| 3.21 \| 1.80E-215 \| 1.03E-213 \| \| ENSG00000143067 \| ZNF697 \| 1.50 \| 4.21E-215 \| 2.40E-213 \| \| ENSG00000136717 \| BIN1 \| -2.36 \| 6.22E-215 \| 3.54E-213 \| \| ENSG00000105472 \| CLEC11A \| -1.47 \| 7.39E-215 \| 4.19E-213 \| \| ENSG00000131378 \| RFTN1 \| 1.32 \| 2.94E-213 \| 1.66E-211 \| \| ENSG00000164442 \| CITED2 \| 1.35 \| 1.16E-212 \| 6.51E-211 \| \| ENSG00000065320 \| NTN1 \| 3.94 \| 1.95E-211 \| 1.09E-209 \| \| ENSG00000138326 \| RPS24 \| -1.01 \| 4.22E-211 \| 2.36E-209 \| \| ENSG00000258227 \| CLEC5A \| 2.50 \| 4.95E-210 \| 2.76E-208 \| \| ENSG00000154645 \| CHODL \| -4.29 \| 1.27E-209 \| 7.06E-208 \| \| ENSG00000136854 \| STXBP1 \| 1.72 \| 1.14E-208 \| 6.27E-207 \| \| ENSG00000145632 \| PLK2 \| -2.86 \| 6.25E-207 \| 3.44E-205 \| \| ENSG00000141458 \| NPC1 \| 1.55 \| 6.46E-207 \| 3.54E-205 \| \| ENSG00000107816 \| LZTS2 \| -1.99 \| 8.43E-207 \| 4.61E-205 \| \| ENSG00000167851 \| CD300A \| -2.55 \| 1.06E-206 \| 5.76E-205 \| \| ENSG00000205302 \| SNX2 \| -1.59 \| 1.74E-206 \| 9.44E-205 \| \| ENSG00000185989 \| RASA3 \| 0.99 \| 1.34E-205 \| 7.24E-204 \| \| ENSG00000137767 \| SQOR \| 1.45 \| 8.25E-205 \| 4.44E-203 \| \| ENSG00000175352 \| NRIP3 \| 1.84 \| 3.51E-204 \| 1.88E-202 \| \| ENSG00000101384 \| JAG1 \| 1.67 \| 1.29E-202 \| 6.92E-201 \| \| ENSG00000057608 \| GDI2 \| -1.06 \| 2.61E-202 \| 1.39E-200 \| \| ENSG00000155629 \| PIK3AP1 \| -2.11 \| 4.09E-201 \| 2.17E-199 \| \| ENSG00000137804 \| NUSAP1 \| -2.41 \| 7.81E-201 \| 4.14E-199 \| \| ENSG00000158869 \| FCER1G \| -1.25 \| 1.40E-200 \| 7.39E-199 \| \| ENSG00000127418 \| FGFRL1 \| 1.11 \| 1.17E-198 \| 6.14E-197 \| \| ENSG00000121691 \| CAT \| -1.82 \| 1.38E-198 \| 7.25E-197 \| \| ENSG00000161638 \| ITGA5 \| 0.93 \| 3.46E-198 \| 1.81E-196 \| \| ENSG00000134070 \| IRAK2 \| 1.74 \| 4.06E-198 \| 2.11E-196 \| \| ENSG00000229953 \|  \| -3.39 \| 6.34E-198 \| 3.29E-196 \| \| ENSG00000074590 \| NUAK1 \| 2.67 \| 2.59E-197 \| 1.34E-195 \| \| ENSG00000120708 \| TGFBI \| -1.31 \| 2.71E-197 \| 1.40E-195 \| \| ENSG00000101347 \| SAMHD1 \| -1.55 \| 5.70E-197 \| 2.93E-195 \| \| ENSG00000117676 \| RPS6KA1 \| 0.96 \| 7.85E-197 \| 4.01E-195 \| \| ENSG00000134686 \| PHC2 \| 0.99 \| 1.17E-195 \| 5.97E-194 \| \| ENSG00000166428 \| PLD4 \| -2.16 \| 1.22E-195 \| 6.20E-194 \| \| ENSG00000110324 \| IL10RA \| -2.33 \| 3.02E-195 \| 1.53E-193 \| \| ENSG00000116962 \| NID1 \| -2.04 \| 6.38E-195 \| 3.22E-193 \| \| ENSG00000213694 \| S1PR3 \| 2.05 \| 8.25E-194 \| 4.15E-192 \| \| ENSG00000213694 \| C9orf47 \| 2.05 \| 8.25E-194 \| 4.15E-192 \| \| ENSG00000007350 \| TKTL1 \| -3.46 \| 3.14E-193 \| 1.58E-191 \| \| ENSG00000169554 \| ZEB2 \| 1.42 \| 4.19E-193 \| 2.09E-191 \| \| ENSG00000103888 \| CEMIP \| 2.10 \| 1.67E-192 \| 8.30E-191 \| \| ENSG00000148798 \| INA \| -3.71 \| 3.79E-192 \| 1.88E-190 \| \| ENSG00000170571 \| EMB \| -2.00 \| 5.25E-192 \| 2.60E-190 \| \| ENSG00000064393 \| HIPK2 \| 1.63 \| 5.05E-190 \| 2.49E-188 \| \| ENSG00000088367 \| EPB41L1 \| -3.37 \| 5.26E-190 \| 2.58E-188 \| \| ENSG00000148400 \| NOTCH1 \| 1.39 \| 1.42E-189 \| 6.97E-188 \| \| ENSG00000105825 \| TFPI2 \| 2.69 \| 1.67E-189 \| 8.13E-188 \| \| ENSG00000100139 \| MICALL1 \| 1.09 \| 1.64E-188 \| 7.97E-187 \| \| ENSG00000078674 \| PCM1 \| -1.15 \| 4.73E-188 \| 2.30E-186 \| \| ENSG00000168958 \| MFF \| 1.17 \| 7.18E-188 \| 3.47E-186 \| \| ENSG00000136830 \| FAM129B \| 0.91 \| 1.91E-187 \| 9.21E-186 \| \| ENSG00000183856 \| IQGAP3 \| -1.67 \| 4.16E-187 \| 2.00E-185 \| \| ENSG00000162704 \| ARPC5 \| -0.95 \| 1.33E-186 \| 6.39E-185 \| \| ENSG00000176890 \| TYMS \| -2.19 \| 3.03E-186 \| 1.45E-184 \| \| ENSG00000198369 \| SPRED2 \| 1.14 \| 6.09E-186 \| 2.90E-184 \| \| ENSG00000160932 \| LY6E \| -1.68 \| 1.26E-185 \| 6.00E-184 \| \| ENSG00000141524 \| TMC6 \| -1.35 \| 2.86E-185 \| 1.35E-183 \| \| ENSG00000131236 \| CAP1 \| -0.75 \| 3.06E-185 \| 1.44E-183 \| \| ENSG00000123080 \| CDKN2C \| -1.78 \| 9.98E-185 \| 4.69E-183 \| \| ENSG00000137331 \| IER3 \| 3.23 \| 2.81E-184 \| 1.32E-182 \| \| ENSG00000049618 \| ARID1B \| 1.29 \| 6.30E-184 \| 2.94E-182 \| \| ENSG00000160796 \| NBEAL2 \| -1.55 \| 6.77E-184 \| 3.15E-182 \| \| ENSG00000171298 \| GAA \| -1.28 \| 7.33E-184 \| 3.40E-182 \| \| ENSG00000120694 \| HSPH1 \| 1.17 \| 1.17E-183 \| 5.44E-182 \| \| ENSG00000105246 \| EBI3 \| 1.73 \| 3.87E-183 \| 1.79E-181 \| \| ENSG00000102359 \| SRPX2 \| 1.81 \| 4.26E-183 \| 1.96E-181 \| \| ENSG00000172379 \| ARNT2 \| 1.19 \| 6.63E-183 \| 3.04E-181 \| \| ENSG00000173334 \| TRIB1 \| 1.18 \| 9.70E-183 \| 4.44E-181 \| \| ENSG00000142173 \| COL6A2 \| 1.01 \| 1.63E-182 \| 7.43E-181 \| \| ENSG00000184678 \| HIST2H2BE \| 1.53 \| 6.95E-182 \| 3.16E-180 \| \| ENSG00000104765 \| BNIP3L \| 1.58 \| 2.97E-181 \| 1.35E-179 \| \| ENSG00000198216 \| CACNA1E \| 2.06 \| 3.75E-181 \| 1.69E-179 \| \| ENSG00000171208 \| NETO2 \| 1.47 \| 2.25E-180 \| 1.01E-178 \| \| ENSG00000271614 \| ATP2B1-AS1 \| 2.99 \| 7.13E-180 \| 3.20E-178 \| \| ENSG00000173852 \| DPY19L1 \| 1.72 \| 2.30E-179 \| 1.03E-177 \| \| ENSG00000107317 \| PTGDS \| -3.31 \| 7.92E-179 \| 3.54E-177 \| \| ENSG00000079215 \| SLC1A3 \| 1.59 \| 1.81E-178 \| 8.05E-177 \| \| ENSG00000162104 \| ADCY9 \| -1.86 \| 1.99E-178 \| 8.84E-177 \| \| ENSG00000105855 \| ITGB8 \| 2.48 \| 3.26E-178 \| 1.44E-176 \| \| ENSG00000113916 \| BCL6 \| 1.38 \| 3.07E-177 \| 1.35E-175 \| \| ENSG00000175445 \| LPL \| -1.16 \| 3.58E-177 \| 1.57E-175 \| \| ENSG00000198668 \| CALM1 \| -1.22 \| 8.28E-177 \| 3.64E-175 \| \| ENSG00000198668 \| CALM3 \| -1.22 \| 8.28E-177 \| 3.64E-175 \| \| ENSG00000175567 \| UCP2 \| -1.38 \| 9.44E-177 \| 4.13E-175 \| \| ENSG00000100292 \| HMOX1 \| -1.62 \| 8.51E-176 \| 3.71E-174 \| \| ENSG00000088325 \| TPX2 \| -2.48 \| 1.06E-175 \| 4.63E-174 \| \| ENSG00000006327 \| TNFRSF12A \| 1.53 \| 3.06E-175 \| 1.33E-173 \| \| ENSG00000131370 \| SH3BP5 \| 1.73 \| 9.56E-175 \| 4.13E-173 \| \| ENSG00000162073 \| PAQR4 \| -1.76 \| 8.30E-174 \| 3.58E-172 \| \| ENSG00000071051 \| NCK2 \| -1.05 \| 8.64E-174 \| 3.72E-172 \| \| ENSG00000124126 \| PREX1 \| 0.93 \| 2.17E-173 \| 9.32E-172 \| \| ENSG00000124762 \| CDKN1A \| 0.92 \| 2.34E-173 \| 1.00E-171 \| \| ENSG00000169567 \| HINT1 \| -1.44 \| 2.42E-173 \| 1.03E-171 \| \| ENSG00000110013 \| SIAE \| -1.49 \| 3.15E-173 \| 1.34E-171 \| \| ENSG00000065989 \| PDE4A \| 1.28 \| 3.91E-173 \| 1.66E-171 \| \| ENSG00000178573 \| MAF \| -1.78 \| 5.84E-173 \| 2.47E-171 \| \| ENSG00000135744 \| AGT \| -1.79 \| 9.63E-173 \| 4.06E-171 \| \| ENSG00000168329 \| CX3CR1 \| -2.96 \| 3.47E-172 \| 1.46E-170 \| \| ENSG00000012061 \| ERCC1 \| 1.20 \| 8.52E-172 \| 3.57E-170 \| \| ENSG00000172508 \| CARNS1 \| -3.56 \| 1.23E-171 \| 5.16E-170 \| \| ENSG00000139318 \| DUSP6 \| 0.82 \| 1.44E-170 \| 6.01E-169 \| \| ENSG00000177189 \| RPS6KA3 \| 1.12 \| 5.15E-170 \| 2.14E-168 \| \| ENSG00000085511 \| MAP3K4 \| 1.31 \| 7.70E-170 \| 3.19E-168 \| \| ENSG00000167850 \| CD300C \| -2.28 \| 1.55E-169 \| 6.39E-168 \| \| ENSG00000013810 \| TACC3 \| -1.92 \| 2.25E-169 \| 9.27E-168 \| \| ENSG00000160584 \| SIK3 \| 1.30 \| 4.75E-169 \| 1.95E-167 \| \| ENSG00000147650 \| LRP12 \| 1.26 \| 6.65E-169 \| 2.73E-167 \| \| ENSG00000020633 \| RUNX3 \| 0.87 \| 1.21E-168 \| 4.94E-167 \| \| ENSG00000170458 \| CD14 \| 1.05 \| 1.36E-168 \| 5.56E-167 \| \| ENSG00000151746 \| BICD1 \| 1.64 \| 3.78E-168 \| 1.54E-166 \| \| ENSG00000145901 \| TNIP1 \| 1.05 \| 4.65E-168 \| 1.89E-166 \| \| ENSG00000143153 \| ATP1B1 \| -2.46 \| 7.15E-167 \| 2.90E-165 \| \| ENSG00000133657 \| ATP13A3 \| 1.05 \| 1.15E-166 \| 4.62E-165 \| \| ENSG00000140678 \| ITGAX \| 0.98 \| 2.15E-166 \| 8.64E-165 \| \| ENSG00000123096 \| SSPN \| 3.75 \| 3.41E-166 \| 1.37E-164 \| \| ENSG00000118113 \| MMP8 \| 2.23 \| 1.24E-165 \| 4.97E-164 \| \| ENSG00000184232 \| OAF \| -1.35 \| 2.22E-165 \| 8.89E-164 \| \| ENSG00000158828 \| PINK1 \| 1.34 \| 2.96E-165 \| 1.18E-163 \| \| ENSG00000119280 \| C1orf198 \| 1.13 \| 5.61E-165 \| 2.23E-163 \| \| ENSG00000197122 \| SRC \| 1.73 \| 1.30E-164 \| 5.16E-163 \| \| ENSG00000090659 \| CD209 \| -2.12 \| 1.54E-164 \| 6.07E-163 \| \| ENSG00000010404 \| IDS \| 0.90 \| 5.55E-164 \| 2.19E-162 \| \| ENSG00000244509 \| APOBEC3C \| -1.76 \| 1.42E-163 \| 5.60E-162 \| \| ENSG00000198901 \| PRC1 \| -2.31 \| 5.48E-163 \| 2.15E-161 \| \| ENSG00000159176 \| CSRP1 \| -0.95 \| 6.45E-163 \| 2.52E-161 \| \| ENSG00000178860 \| MSC \| 1.58 \| 9.68E-163 \| 3.78E-161 \| \| ENSG00000140526 \| ABHD2 \| 0.95 \| 1.44E-162 \| 5.59E-161 \| \| ENSG00000162894 \| FCMR \| -3.67 \| 3.36E-162 \| 1.30E-160 \| \| ENSG00000181656 \| GPR88 \| 3.07 \| 5.22E-162 \| 2.02E-160 \| \| ENSG00000182578 \| CSF1R \| 0.78 \| 7.44E-161 \| 2.87E-159 \| \| ENSG00000109861 \| CTSC \| -0.90 \| 1.10E-160 \| 4.25E-159 \| \| ENSG00000133142 \| TCEAL4 \| -2.05 \| 1.19E-159 \| 4.57E-158 \| \| ENSG00000060237 \| WNK1 \| -0.88 \| 1.65E-159 \| 6.31E-158 \| \| ENSG00000064932 \| SBNO2 \| 1.02 \| 2.28E-159 \| 8.73E-158 \| \| ENSG00000237649 \| KIFC1 \| -2.41 \| 3.61E-159 \| 1.38E-157 \| \| ENSG00000172081 \| MOB3A \| 0.87 \| 1.28E-158 \| 4.87E-157 \| \| ENSG00000175130 \| MARCKSL1 \| 0.98 \| 3.14E-158 \| 1.19E-156 \| \| ENSG00000235568 \| NFAM1 \| -1.66 \| 1.04E-156 \| 3.92E-155 \| \| ENSG00000164484 \| TMEM200A \| 1.91 \| 1.82E-156 \| 6.87E-155 \| \| ENSG00000198467 \| TPM2 \| -1.45 \| 3.45E-156 \| 1.30E-154 \| \| ENSG00000163814 \| CDCP1 \| 1.95 \| 5.50E-156 \| 2.07E-154 \| \| ENSG00000135916 \| ITM2C \| -2.50 \| 6.05E-156 \| 2.27E-154 \| \| ENSG00000183963 \| SMTN \| 1.62 \| 6.51E-156 \| 2.43E-154 \| \| ENSG00000143870 \| PDIA6 \| -0.87 \| 1.71E-155 \| 6.36E-154 \| \| ENSG00000156802 \| ATAD2 \| -1.63 \| 7.38E-155 \| 2.75E-153 \| \| ENSG00000104763 \| ASAH1 \| -1.10 \| 1.12E-154 \| 4.15E-153 \| \| ENSG00000198715 \| GLMP \| -1.23 \| 1.44E-154 \| 5.33E-153 \| \| ENSG00000187079 \| TEAD1 \| 1.46 \| 1.75E-154 \| 6.46E-153 \| \| ENSG00000116574 \| RHOU \| -1.86 \| 3.28E-154 \| 1.21E-152 \| \| ENSG00000116337 \| AMPD2 \| 1.16 \| 3.33E-154 \| 1.22E-152 \| \| ENSG00000164647 \| STEAP1 \| 2.05 \| 6.87E-154 \| 2.52E-152 \| \| ENSG00000112335 \| SNX3 \| -0.92 \| 7.10E-154 \| 2.60E-152 \| \| ENSG00000160013 \| PTGIR \| 3.16 \| 7.30E-154 \| 2.66E-152 \| \| ENSG00000256235 \| SMIM3 \| 1.58 \| 4.20E-153 \| 1.53E-151 \| \| ENSG00000143320 \| CRABP2 \| -3.61 \| 4.85E-153 \| 1.76E-151 \| \| ENSG00000172889 \| EGFL7 \| -1.72 \| 8.18E-153 \| 2.96E-151 \| \| ENSG00000152270 \| PDE3B \| 1.61 \| 1.49E-151 \| 5.39E-150 \| \| ENSG00000150867 \| PIP4K2A \| -1.34 \| 2.71E-151 \| 9.76E-150 \| \| ENSG00000163683 \| SMIM14 \| 1.88 \| 3.04E-151 \| 1.09E-149 \| \| ENSG00000132205 \| EMILIN2 \| 0.86 \| 4.21E-151 \| 1.51E-149 \| \| ENSG00000104885 \| DOT1L \| 0.84 \| 5.67E-151 \| 2.03E-149 \| \| ENSG00000100266 \| PACSIN2 \| -1.05 \| 6.89E-151 \| 2.46E-149 \| \| ENSG00000075618 \| FSCN1 \| -1.04 \| 1.99E-150 \| 7.10E-149 \| \| ENSG00000213614 \| HEXA \| -1.61 \| 2.42E-150 \| 8.59E-149 \| \| ENSG00000166681 \| BEX3 \| -1.97 \| 2.62E-150 \| 9.28E-149 \| \| ENSG00000066294 \| CD84 \| -4.37 \| 4.61E-150 \| 1.63E-148 \| \| ENSG00000116260 \| QSOX1 \| -0.99 \| 1.23E-149 \| 4.34E-148 \| \| ENSG00000111331 \| OAS3 \| -1.92 \| 2.63E-149 \| 9.27E-148 \| \| ENSG00000089820 \| ARHGAP4 \| -1.11 \| 2.75E-149 \| 9.65E-148 \| \| ENSG00000162676 \| GFI1 \| -1.78 \| 4.45E-149 \| 1.56E-147 \| \| ENSG00000092841 \| MYL6 \| -0.94 \| 9.81E-149 \| 3.43E-147 \| \| ENSG00000149798 \| CDC42EP2 \| 2.71 \| 1.28E-148 \| 4.46E-147 \| \| ENSG00000130479 \| MAP1S \| 0.81 \| 2.45E-148 \| 8.53E-147 \| \| ENSG00000167378 \| IRGQ \| 1.39 \| 1.18E-147 \| 4.10E-146 \| \| ENSG00000079277 \| MKNK1 \| -1.60 \| 6.80E-147 \| 2.35E-145 \| \| ENSG00000256894 \|  \| 4.01 \| 1.15E-146 \| 3.98E-145 \| \| ENSG00000124151 \| NCOA3 \| 1.17 \| 1.75E-146 \| 6.03E-145 \| \| ENSG00000142945 \| KIF2C \| -2.41 \| 5.62E-146 \| 1.93E-144 \| \| ENSG00000196776 \| CD47 \| -1.53 \| 1.06E-145 \| 3.64E-144 \| \| ENSG00000064601 \| CTSA \| -0.94 \| 1.57E-145 \| 5.39E-144 \| \| ENSG00000133805 \| AMPD3 \| 1.14 \| 1.83E-145 \| 6.25E-144 \| \| ENSG00000081923 \| ATP8B1 \| -3.09 \| 2.21E-145 \| 7.55E-144 \| \| ENSG00000134986 \| NREP \| 1.30 \| 4.14E-145 \| 1.41E-143 \| \| ENSG00000074317 \| SNCB \| 3.35 \| 7.74E-145 \| 2.63E-143 \| \| ENSG00000217236 \| SP9 \| 1.30 \| 2.33E-144 \| 7.91E-143 \| \| ENSG00000170396 \| ZNF804A \| 1.47 \| 5.44E-144 \| 1.84E-142 \| \| ENSG00000197746 \| PSAP \| -0.66 \| 1.70E-143 \| 5.73E-142 \| \| ENSG00000266094 \| RASSF5 \| -1.28 \| 2.22E-143 \| 7.46E-142 \| \| ENSG00000121966 \| CXCR4 \| 2.07 \| 2.23E-143 \| 7.49E-142 \| \| ENSG00000140262 \| TCF12 \| -0.99 \| 8.72E-143 \| 2.92E-141 \| \| ENSG00000163346 \| PBXIP1 \| -1.56 \| 1.11E-142 \| 3.71E-141 \| \| ENSG00000111424 \| VDR \| 1.80 \| 3.68E-142 \| 1.23E-140 \| \| ENSG00000104805 \| NUCB1 \| -0.96 \| 5.01E-142 \| 1.67E-140 \| \| ENSG00000198826 \| ARHGAP11A \| -2.19 \| 6.01E-142 \| 1.99E-140 \| \| ENSG00000131724 \| IL13RA1 \| 1.04 \| 1.19E-140 \| 3.95E-139 \| \| ENSG00000119714 \| GPR68 \| 1.27 \| 3.92E-140 \| 1.29E-138 \| \| ENSG00000137496 \| IL18BP \| -2.19 \| 5.66E-140 \| 1.87E-138 \| \| ENSG00000196126 \| HLA-DRB1 \| -2.79 \| 2.12E-139 \| 6.99E-138 \| \| ENSG00000106546 \| AHR \| 1.95 \| 2.64E-139 \| 8.67E-138 \| \| ENSG00000106089 \| STX1A \| 1.20 \| 3.28E-139 \| 1.08E-137 \| \| ENSG00000167900 \| TK1 \| -2.54 \| 3.98E-139 \| 1.30E-137 \| \| ENSG00000102032 \| RENBP \| -1.95 \| 4.94E-139 \| 1.61E-137 \| \| ENSG00000110047 \| EHD1 \| 1.40 \| 6.16E-139 \| 2.00E-137 \| \| ENSG00000127947 \| PTPN12 \| 0.84 \| 6.36E-139 \| 2.07E-137 \| \| ENSG00000142871 \| CYR61 \| 3.79 \| 1.27E-138 \| 4.10E-137 \| \| ENSG00000116731 \| PRDM2 \| 1.28 \| 3.42E-138 \| 1.10E-136 \| \| ENSG00000198794 \| SCAMP5 \| -2.24 \| 3.50E-138 \| 1.13E-136 \| \| ENSG00000112118 \| MCM3 \| -1.22 \| 4.41E-138 \| 1.42E-136 \| \| ENSG00000152894 \| PTPRK \| 1.29 \| 6.08E-138 \| 1.96E-136 \| \| ENSG00000182511 \| FES \| -2.77 \| 7.43E-138 \| 2.38E-136 \| \| ENSG00000106348 \| IMPDH1 \| -1.63 \| 3.79E-137 \| 1.21E-135 \| \| ENSG00000133612 \| AGAP3 \| 0.87 \| 4.34E-137 \| 1.39E-135 \| \| ENSG00000175592 \| FOSL1 \| 1.31 \| 6.76E-137 \| 2.16E-135 \| \| ENSG00000145555 \| MYO10 \| 1.42 \| 7.68E-137 \| 2.44E-135 \| \| ENSG00000164181 \| ELOVL7 \| 2.65 \| 9.88E-137 \| 3.14E-135 \| \| ENSG00000163734 \| CXCL3 \| 4.14 \| 4.03E-136 \| 1.28E-134 \| \| ENSG00000110057 \| UNC93B1 \| 1.11 \| 4.11E-136 \| 1.30E-134 \| \| ENSG00000078804 \| TP53INP2 \| 1.41 \| 5.02E-136 \| 1.58E-134 \| \| ENSG00000122694 \| GLIPR2 \| -1.78 \| 1.48E-135 \| 4.66E-134 \| \| ENSG00000100985 \| MMP9 \| -1.04 \| 2.80E-135 \| 8.80E-134 \| \| ENSG00000125354 \| 06-Sep \| -2.71 \| 7.77E-135 \| 2.43E-133 \| \| ENSG00000108846 \| ABCC3 \| 1.01 \| 1.34E-134 \| 4.19E-133 \| \| ENSG00000168710 \| AHCYL1 \| 0.88 \| 1.53E-134 \| 4.77E-133 \| \| ENSG00000178695 \| KCTD12 \| 1.10 \| 2.92E-134 \| 9.08E-133 \| \| ENSG00000167703 \| SLC43A2 \| 1.84 \| 3.90E-134 \| 1.21E-132 \| \| ENSG00000233276 \| GPX1 \| -0.85 \| 5.04E-134 \| 1.56E-132 \| \| ENSG00000122042 \| UBL3 \| 1.28 \| 7.50E-134 \| 2.32E-132 \| \| ENSG00000117724 \| CENPF \| -2.43 \| 9.50E-134 \| 2.94E-132 \| \| ENSG00000156587 \| UBE2L6 \| -2.05 \| 1.13E-133 \| 3.50E-132 \| \| ENSG00000120896 \| SORBS3 \| -1.49 \| 1.90E-133 \| 5.86E-132 \| \| ENSG00000166851 \| PLK1 \| -2.90 \| 2.89E-133 \| 8.87E-132 \| \| ENSG00000123908 \| AGO2 \| 1.10 \| 8.57E-133 \| 2.63E-131 \| \| ENSG00000107719 \| PALD1 \| -2.97 \| 9.37E-133 \| 2.87E-131 \| \| ENSG00000196415 \| PRTN3 \| -6.96 \| 1.16E-132 \| 3.53E-131 \| \| ENSG00000163297 \| ANTXR2 \| 0.98 \| 1.42E-132 \| 4.33E-131 \| \| ENSG00000033170 \| FUT8 \| 1.28 \| 1.53E-131 \| 4.64E-130 \| \| ENSG00000137203 \| TFAP2A \| 2.16 \| 1.65E-131 \| 5.01E-130 \| \| ENSG00000171522 \| PTGER4 \| 1.36 \| 3.79E-131 \| 1.15E-129 \| \| ENSG00000164307 \| ERAP1 \| -1.22 \| 6.16E-131 \| 1.86E-129 \| \| ENSG00000225968 \| ELFN1 \| 2.20 \| 6.52E-131 \| 1.97E-129 \| \| ENSG00000068489 \| PRR11 \| -2.63 \| 1.56E-130 \| 4.69E-129 \| \| ENSG00000034053 \| APBA2 \| 1.26 \| 1.71E-130 \| 5.14E-129 \| \| ENSG00000132170 \| PPARG \| -1.51 \| 2.00E-130 \| 5.99E-129 \| \| ENSG00000203875 \| SNHG5 \| -1.99 \| 3.42E-130 \| 1.02E-128 \| \| ENSG00000125266 \| EFNB2 \| 3.09 \| 3.64E-130 \| 1.09E-128 \| \| ENSG00000174307 \| PHLDA3 \| -2.21 \| 9.98E-130 \| 2.97E-128 \| \| ENSG00000105122 \| RASAL3 \| -1.59 \| 1.20E-129 \| 3.58E-128 \| \| ENSG00000119655 \| NPC2 \| -1.08 \| 1.42E-129 \| 4.21E-128 \| \| ENSG00000090020 \| SLC9A1 \| 1.01 \| 1.45E-129 \| 4.30E-128 \| \| ENSG00000133943 \| DGLUCY \| -1.78 \| 1.83E-129 \| 5.42E-128 \| \| ENSG00000157551 \| KCNJ15 \| 4.53 \| 2.59E-129 \| 7.64E-128 \| \| ENSG00000198848 \| CES1 \| -3.24 \| 2.60E-129 \| 7.67E-128 \| \| ENSG00000166780 \| C16orf45 \| 1.75 \| 3.43E-129 \| 1.01E-127 \| \| ENSG00000205927 \| OLIG2 \| 2.53 \| 3.47E-129 \| 1.02E-127 \| \| ENSG00000198598 \| MMP17 \| -2.31 \| 9.93E-129 \| 2.91E-127 \| \| ENSG00000167291 \| TBC1D16 \| 1.02 \| 1.22E-128 \| 3.56E-127 \| \| ENSG00000180044 \| C3orf80 \| -1.87 \| 1.37E-128 \| 4.00E-127 \| \| ENSG00000095303 \| PTGS1 \| -0.96 \| 1.63E-128 \| 4.73E-127 \| \| ENSG00000234465 \| PINLYP \| -0.79 \| 2.08E-128 \| 6.06E-127 \| \| ENSG00000128342 \| LIF \| 5.24 \| 3.58E-128 \| 1.04E-126 \| \| ENSG00000172270 \| BSG \| -0.81 \| 8.09E-128 \| 2.34E-126 \| \| ENSG00000137193 \| PIM1 \| 1.09 \| 1.05E-127 \| 3.03E-126 \| \| ENSG00000171988 \| JMJD1C \| -1.15 \| 1.43E-127 \| 4.13E-126 \| \| ENSG00000151575 \| TEX9 \| 1.50 \| 2.02E-127 \| 5.83E-126 \| \| ENSG00000092820 \| EZR \| -1.23 \| 2.99E-127 \| 8.59E-126 \| \| ENSG00000065534 \| MYLK \| -1.13 \| 4.25E-127 \| 1.22E-125 \| \| ENSG00000189319 \| FAM53B \| -2.34 \| 4.28E-127 \| 1.22E-125 \| \| ENSG00000175040 \| CHST2 \| 1.78 \| 6.14E-127 \| 1.76E-125 \| \| ENSG00000095752 \| IL11 \| 4.92 \| 8.65E-127 \| 2.47E-125 \| \| ENSG00000087495 \| PHACTR3 \| -2.67 \| 3.68E-126 \| 1.05E-124 \| \| ENSG00000242247 \| ARFGAP3 \| 1.33 \| 7.44E-126 \| 2.11E-124 \| \| ENSG00000086666 \| ZFAND6 \| 1.07 \| 8.16E-126 \| 2.31E-124 \| \| ENSG00000148730 \| EIF4EBP2 \| -0.90 \| 1.18E-125 \| 3.33E-124 \| \| ENSG00000250657 \|  \| 2.80 \| 1.19E-125 \| 3.36E-124 \| \| ENSG00000010278 \| CD9 \| -1.70 \| 1.35E-125 \| 3.80E-124 \| \| ENSG00000134294 \| SLC38A2 \| 1.23 \| 1.83E-125 \| 5.14E-124 \| \| ENSG00000020181 \| ADGRA2 \| -2.39 \| 2.58E-125 \| 7.24E-124 \| \| ENSG00000163131 \| CTSS \| -1.02 \| 9.59E-125 \| 2.69E-123 \| \| ENSG00000204438 \| GPANK1 \| 1.35 \| 1.72E-124 \| 4.81E-123 \| \| ENSG00000188786 \| MTF1 \| 1.31 \| 5.80E-124 \| 1.62E-122 \| \| ENSG00000125089 \| SH3TC1 \| -1.26 \| 6.34E-124 \| 1.77E-122 \| \| ENSG00000159212 \| CLIC6 \| 2.54 \| 1.19E-123 \| 3.30E-122 \| \| ENSG00000196935 \| SRGAP1 \| 1.72 \| 2.99E-123 \| 8.32E-122 \| \| ENSG00000033867 \| SLC4A7 \| 1.31 \| 3.80E-123 \| 1.05E-121 \| \| ENSG00000122122 \| SASH3 \| -1.97 \| 4.14E-123 \| 1.15E-121 \| \| ENSG00000113721 \| PDGFRB \| 4.02 \| 3.40E-122 \| 9.39E-121 \| \| ENSG00000167895 \| TMC8 \| -2.08 \| 6.76E-122 \| 1.87E-120 \| \| ENSG00000206560 \| ANKRD28 \| 1.11 \| 7.70E-122 \| 2.12E-120 \| \| ENSG00000153029 \| MR1 \| -1.40 \| 1.35E-121 \| 3.72E-120 \| \| ENSG00000185420 \| SMYD3 \| -2.78 \| 1.56E-121 \| 4.27E-120 \| \| ENSG00000104964 \| AES \| -1.00 \| 6.36E-121 \| 1.74E-119 \| \| ENSG00000101412 \| E2F1 \| -1.86 \| 6.45E-121 \| 1.76E-119 \| \| ENSG00000137673 \| MMP7 \| 3.54 \| 7.94E-121 \| 2.17E-119 \| \| ENSG00000169439 \| SDC2 \| 0.65 \| 9.85E-121 \| 2.68E-119 \| \| ENSG00000240849 \| TMEM189 \| 1.09 \| 2.79E-120 \| 7.57E-119 \| \| ENSG00000123485 \| HJURP \| -2.73 \| 8.88E-120 \| 2.41E-118 \| \| ENSG00000167470 \| MIDN \| 0.90 \| 2.83E-119 \| 7.65E-118 \| \| ENSG00000198814 \| GK \| 1.72 \| 3.26E-119 \| 8.80E-118 \| \| ENSG00000074706 \| IPCEF1 \| -1.19 \| 6.93E-119 \| 1.87E-117 \| \| ENSG00000164104 \| HMGB2 \| -1.34 \| 8.28E-119 \| 2.23E-117 \| \| ENSG00000263528 \| IKBKE \| -1.40 \| 1.38E-118 \| 3.72E-117 \| \| ENSG00000113368 \| LMNB1 \| -1.46 \| 1.55E-118 \| 4.16E-117 \| \| ENSG00000181104 \| F2R \| -2.15 \| 2.99E-118 \| 8.02E-117 \| \| ENSG00000113758 \| DBN1 \| -1.19 \| 5.41E-118 \| 1.45E-116 \| \| ENSG00000140199 \| SLC12A6 \| 0.93 \| 7.74E-118 \| 2.07E-116 \| \| ENSG00000159307 \| SCUBE1 \| -1.98 \| 9.11E-118 \| 2.43E-116 \| \| ENSG00000082074 \| FYB1 \| -2.09 \| 1.76E-117 \| 4.68E-116 \| \| ENSG00000151012 \| SLC7A11 \| 1.79 \| 5.04E-117 \| 1.34E-115 \| \| ENSG00000146535 \| GNA12 \| 0.83 \| 6.11E-117 \| 1.62E-115 \| \| ENSG00000168487 \| BMP1 \| 1.07 \| 7.06E-117 \| 1.87E-115 \| \| ENSG00000151490 \| PTPRO \| -2.89 \| 7.50E-117 \| 1.98E-115 \| \| ENSG00000196396 \| PTPN1 \| 0.98 \| 1.67E-116 \| 4.41E-115 \| \| ENSG00000170312 \| CDK1 \| -3.19 \| 1.68E-116 \| 4.41E-115 \| \| ENSG00000107281 \| NPDC1 \| -1.31 \| 1.86E-116 \| 4.90E-115 \| \| ENSG00000173227 \| SYT12 \| 2.11 \| 2.85E-116 \| 7.48E-115 \| \| ENSG00000146457 \| WTAP \| 1.03 \| 2.92E-116 \| 7.66E-115 \| \| ENSG00000138801 \| PAPSS1 \| 0.92 \| 3.01E-116 \| 7.88E-115 \| \| ENSG00000156011 \| PSD3 \| 1.27 \| 8.97E-116 \| 2.34E-114 \| \| ENSG00000163739 \| CXCL1 \| 3.84 \| 1.83E-115 \| 4.77E-114 \| \| ENSG00000089248 \| ERP29 \| -1.19 \| 1.92E-115 \| 5.00E-114 \| \| ENSG00000231925 \| TAPBP \| -0.73 \| 5.85E-115 \| 1.52E-113 \| \| ENSG00000103202 \| NME4 \| -1.11 \| 6.84E-115 \| 1.77E-113 \| \| ENSG00000107372 \| ZFAND5 \| 0.77 \| 8.81E-115 \| 2.28E-113 \| \| ENSG00000117592 \| PRDX6 \| -0.92 \| 1.07E-114 \| 2.78E-113 \| \| ENSG00000240065 \| PSMB9 \| -2.47 \| 1.12E-114 \| 2.90E-113 \| \| ENSG00000091129 \| NRCAM \| 2.21 \| 1.58E-114 \| 4.08E-113 \| \| ENSG00000196187 \| TMEM63A \| -1.06 \| 2.15E-114 \| 5.52E-113 \| \| ENSG00000158106 \| RHPN1 \| -2.22 \| 2.46E-114 \| 6.32E-113 \| \| ENSG00000196743 \| GM2A \| -1.52 \| 2.60E-114 \| 6.66E-113 \| \| ENSG00000177791 \| MYOZ1 \| -2.90 \| 3.55E-114 \| 9.07E-113 \| \| ENSG00000167994 \| RAB3IL1 \| -1.86 \| 3.72E-114 \| 9.51E-113 \| \| ENSG00000157557 \| ETS2 \| 1.26 \| 1.31E-113 \| 3.33E-112 \| \| ENSG00000168938 \| PPIC \| -4.50 \| 1.50E-113 \| 3.82E-112 \| \| ENSG00000166592 \| RRAD \| 2.45 \| 1.66E-113 \| 4.22E-112 \| \| ENSG00000095970 \| TREM2 \| -1.05 \| 2.71E-113 \| 6.87E-112 \| \| ENSG00000111206 \| FOXM1 \| -2.45 \| 5.45E-113 \| 1.38E-111 \| \| ENSG00000156313 \| RPGR \| 1.55 \| 9.64E-113 \| 2.44E-111 \| \| ENSG00000105048 \| TNNT1 \| -1.72 \| 1.32E-112 \| 3.33E-111 \| \| ENSG00000198951 \| NAGA \| -0.96 \| 1.45E-112 \| 3.64E-111 \| \| ENSG00000102144 \| PGK1 \| 0.81 \| 2.39E-112 \| 6.00E-111 \| \| ENSG00000228794 \| LINC01128 \| 1.83 \| 2.39E-112 \| 6.00E-111 \| \| ENSG00000165025 \| SYK \| -0.96 \| 2.62E-112 \| 6.57E-111 \| \| ENSG00000148248 \| SURF4 \| 0.72 \| 3.42E-112 \| 8.55E-111 \| \| ENSG00000091409 \| ITGA6 \| 1.12 \| 5.80E-112 \| 1.45E-110 \| \| ENSG00000102554 \| KLF5 \| 1.90 \| 6.22E-112 \| 1.55E-110 \| \| ENSG00000112096 \| SOD2 \| 2.80 \| 8.57E-112 \| 2.13E-110 \| \| ENSG00000143013 \| LMO4 \| 1.06 \| 9.67E-112 \| 2.40E-110 \| \| ENSG00000115652 \| UXS1 \| 0.95 \| 1.19E-111 \| 2.95E-110 \| \| ENSG00000163219 \| ARHGAP25 \| -1.74 \| 2.27E-111 \| 5.63E-110 \| \| ENSG00000140525 \| FANCI \| -1.67 \| 2.60E-111 \| 6.43E-110 \| \| ENSG00000007237 \| GAS7 \| -1.08 \| 3.03E-111 \| 7.48E-110 \| \| ENSG00000143476 \| DTL \| -2.25 \| 7.49E-111 \| 1.85E-109 \| \| ENSG00000164935 \| DCSTAMP \| 1.35 \| 7.63E-111 \| 1.88E-109 \| \| ENSG00000111348 \| ARHGDIB \| -0.83 \| 8.93E-111 \| 2.20E-109 \| \| ENSG00000010256 \| UQCRC1 \| 0.83 \| 1.20E-110 \| 2.95E-109 \| \| ENSG00000248360 \| LINC00504 \| -2.02 \| 1.34E-110 \| 3.28E-109 \| \| ENSG00000182247 \| UBE2E2 \| 1.28 \| 1.65E-110 \| 4.04E-109 \| \| ENSG00000136867 \| SLC31A2 \| 1.15 \| 1.86E-110 \| 4.54E-109 \| \| ENSG00000100365 \| NCF4 \| -1.37 \| 2.06E-110 \| 5.03E-109 \| \| ENSG00000050767 \| COL23A1 \| 1.43 \| 2.59E-110 \| 6.30E-109 \| \| ENSG00000166401 \| SERPINB8 \| 1.04 \| 6.25E-110 \| 1.52E-108 \| \| ENSG00000070882 \| OSBPL3 \| -1.21 \| 7.39E-110 \| 1.79E-108 \| \| ENSG00000162736 \| NCSTN \| -0.71 \| 8.61E-110 \| 2.08E-108 \| \| ENSG00000170275 \| CRTAP \| -1.02 \| 8.80E-110 \| 2.13E-108 \| \| ENSG00000123104 \| ITPR2 \| 1.49 \| 9.10E-110 \| 2.20E-108 \| \| ENSG00000107521 \| HPS1 \| -0.99 \| 1.38E-109 \| 3.34E-108 \| \| ENSG00000152377 \| SPOCK1 \| 1.18 \| 2.67E-109 \| 6.43E-108 \| \| ENSG00000144724 \| PTPRG \| 1.00 \| 2.83E-109 \| 6.81E-108 \| \| ENSG00000165629 \| ATP5F1C \| -1.06 \| 3.52E-109 \| 8.45E-108 \| \| ENSG00000139572 \| GPR84 \| 1.22 \| 3.54E-109 \| 8.48E-108 \| \| ENSG00000196730 \| DAPK1 \| -1.47 \| 4.41E-109 \| 1.06E-107 \| \| ENSG00000168374 \| ARF4 \| 0.87 \| 5.51E-109 \| 1.32E-107 \| \| ENSG00000122705 \| CLTA \| -0.81 \| 6.24E-109 \| 1.49E-107 \| \| ENSG00000100297 \| MCM5 \| -0.99 \| 2.22E-108 \| 5.29E-107 \| \| ENSG00000113555 \| PCDH12 \| -2.19 \| 3.23E-108 \| 7.67E-107 \| \| ENSG00000103485 \| QPRT \| -1.49 \| 3.27E-108 \| 7.76E-107 \| \| ENSG00000196230 \| TUBB \| -0.67 \| 5.28E-108 \| 1.25E-106 \| \| ENSG00000172716 \| SLFN11 \| -2.67 \| 8.06E-108 \| 1.91E-106 \| \| ENSG00000221955 \| SLC12A8 \| -1.92 \| 8.15E-108 \| 1.92E-106 \| \| ENSG00000259207 \| ITGB3 \| 2.50 \| 8.82E-108 \| 2.08E-106 \| \| ENSG00000185070 \| FLRT2 \| 1.86 \| 1.18E-107 \| 2.77E-106 \| \| ENSG00000120049 \| KCNIP2 \| -2.97 \| 1.65E-107 \| 3.87E-106 \| \| ENSG00000145386 \| CCNA2 \| -2.40 \| 1.80E-107 \| 4.22E-106 \| \| ENSG00000186318 \| BACE1 \| -1.14 \| 2.29E-107 \| 5.37E-106 \| \| ENSG00000073111 \| MCM2 \| -1.18 \| 3.49E-107 \| 8.16E-106 \| \| ENSG00000134602 \| STK26 \| 1.53 \| 3.54E-107 \| 8.28E-106 \| \| ENSG00000169679 \| BUB1 \| -2.64 \| 4.24E-107 \| 9.90E-106 \| \| ENSG00000153551 \| CMTM7 \| -1.38 \| 4.96E-107 \| 1.16E-105 \| \| ENSG00000128268 \| MGAT3 \| 2.37 \| 6.77E-107 \| 1.57E-105 \| \| ENSG00000132510 \| KDM6B \| 1.41 \| 9.31E-107 \| 2.16E-105 \| \| ENSG00000081052 \| COL4A4 \| 1.74 \| 1.24E-106 \| 2.88E-105 \| \| ENSG00000144476 \| ACKR3 \| 1.71 \| 1.27E-106 \| 2.94E-105 \| \| ENSG00000179630 \| LACC1 \| 0.94 \| 1.32E-106 \| 3.05E-105 \| \| ENSG00000166224 \| SGPL1 \| -1.63 \| 1.76E-106 \| 4.06E-105 \| \| ENSG00000160803 \| UBQLN4 \| 0.91 \| 1.94E-106 \| 4.46E-105 \| \| ENSG00000144655 \| CSRNP1 \| 1.26 \| 2.09E-106 \| 4.80E-105 \| \| ENSG00000099219 \| ERMP1 \| -1.05 \| 2.32E-106 \| 5.32E-105 \| \| ENSG00000156508 \| EEF1A1 \| -0.64 \| 2.40E-106 \| 5.50E-105 \| \| ENSG00000178685 \| PARP10 \| -1.31 \| 2.53E-106 \| 5.79E-105 \| \| ENSG00000181804 \| SLC9A9 \| -1.76 \| 3.82E-106 \| 8.73E-105 \| \| ENSG00000131435 \| PDLIM4 \| -2.30 \| 4.05E-106 \| 9.24E-105 \| \| ENSG00000159335 \| PTMS \| -1.24 \| 5.34E-106 \| 1.22E-104 \| \| ENSG00000138160 \| KIF11 \| -3.15 \| 6.21E-106 \| 1.41E-104 \| \| ENSG00000142192 \| APP \| -0.72 \| 7.13E-106 \| 1.62E-104 \| \| ENSG00000142583 \| SLC2A5 \| -2.74 \| 9.93E-106 \| 2.26E-104 \| \| ENSG00000134061 \| CD180 \| -2.87 \| 1.30E-105 \| 2.96E-104 \| \| ENSG00000102024 \| PLS3 \| -2.45 \| 1.34E-105 \| 3.03E-104 \| \| ENSG00000177311 \| ZBTB38 \| 1.25 \| 1.73E-105 \| 3.92E-104 \| \| ENSG00000107551 \| RASSF4 \| -1.86 \| 6.88E-105 \| 1.55E-103 \| \| ENSG00000132329 \| RAMP1 \| -1.99 \| 8.03E-105 \| 1.81E-103 \| \| ENSG00000244026 \| FAM86DP \| 1.45 \| 1.46E-104 \| 3.27E-103 \| \| ENSG00000109814 \| UGDH \| 1.36 \| 1.62E-104 \| 3.64E-103 \| \| ENSG00000160957 \| RECQL4 \| -1.47 \| 1.81E-104 \| 4.07E-103 \| \| ENSG00000072201 \| LNX1 \| 3.47 \| 2.67E-104 \| 5.97E-103 \| \| ENSG00000185686 \| PRAME \| -1.16 \| 4.13E-104 \| 9.24E-103 \| \| ENSG00000171848 \| RRM2 \| -1.48 \| 4.24E-104 \| 9.47E-103 \| \| ENSG00000182389 \| CACNB4 \| -3.46 \| 5.22E-104 \| 1.16E-102 \| \| ENSG00000272825 \|  \| 1.79 \| 5.41E-104 \| 1.20E-102 \| \| ENSG00000196586 \| MYO6 \| -0.94 \| 9.00E-104 \| 2.00E-102 \| \| ENSG00000147813 \| NAPRT \| -1.14 \| 1.37E-103 \| 3.04E-102 \| \| ENSG00000162458 \| FBLIM1 \| -3.08 \| 1.66E-103 \| 3.67E-102 \| \| ENSG00000151694 \| ADAM17 \| 0.71 \| 2.09E-103 \| 4.64E-102 \| \| ENSG00000117394 \| SLC2A1 \| 0.71 \| 2.79E-103 \| 6.16E-102 \| \| ENSG00000139977 \| NAA30 \| 1.30 \| 3.44E-103 \| 7.59E-102 \| \| ENSG00000106948 \| AKNA \| -0.96 \| 3.52E-103 \| 7.75E-102 \| \| ENSG00000131979 \| GCH1 \| 2.26 \| 3.60E-103 \| 7.91E-102 \| \| ENSG00000139438 \| FAM222A \| 1.90 \| 6.14E-103 \| 1.35E-101 \| \| ENSG00000259642 \|  \| 1.98 \| 9.61E-103 \| 2.11E-101 \| \| ENSG00000163191 \| S100A11 \| 0.73 \| 1.32E-102 \| 2.90E-101 \| \| ENSG00000162722 \| TRIM58 \| -2.04 \| 3.29E-102 \| 7.19E-101 \| \| ENSG00000107331 \| ABCA2 \| -0.88 \| 3.67E-102 \| 8.02E-101 \| \| ENSG00000109320 \| NFKB1 \| 0.89 \| 4.24E-102 \| 9.24E-101 \| \| ENSG00000010810 \| FYN \| 0.83 \| 6.39E-102 \| 1.39E-100 \| \| ENSG00000165731 \| RET \| -1.64 \| 6.77E-102 \| 1.47E-100 \| \| ENSG00000042493 \| CAPG \| -1.04 \| 6.99E-102 \| 1.52E-100 \| \| ENSG00000089685 \| BIRC5 \| -2.57 \| 1.47E-101 \| 3.20E-100 \| \| ENSG00000198517 \| MAFK \| 1.08 \| 1.64E-101 \| 3.56E-100 \| \| ENSG00000213585 \| VDAC1 \| -0.85 \| 3.27E-101 \| 7.07E-100 \| \| ENSG00000074964 \| ARHGEF10L \| -1.89 \| 4.09E-101 \| 8.82E-100 \| \| ENSG00000155254 \| MARVELD1 \| -0.88 \| 4.16E-101 \| 8.98E-100 \| \| ENSG00000106785 \| TRIM14 \| -1.45 \| 4.51E-101 \| 9.71E-100 \| \| ENSG00000143545 \| RAB13 \| 0.74 \| 5.21E-101 \| 1.12E-99 \| \| ENSG00000115590 \| IL1R2 \| 4.47 \| 5.90E-101 \| 1.27E-99 \| \| ENSG00000135776 \| ABCB10 \| -1.39 \| 7.38E-101 \| 1.58E-99 \| \| ENSG00000136770 \| DNAJC1 \| -1.78 \| 9.29E-101 \| 1.99E-99 \| \| ENSG00000228716 \| DHFR \| -1.62 \| 1.16E-100 \| 2.48E-99 \| \| ENSG00000026297 \| RNASET2 \| -1.27 \| 1.22E-100 \| 2.60E-99 \| \| ENSG00000066279 \| ASPM \| -3.27 \| 2.38E-100 \| 5.07E-99 \| \| ENSG00000157303 \| SUSD3 \| -4.67 \| 2.47E-100 \| 5.26E-99 \| \| ENSG00000182287 \| AP1S2 \| 0.80 \| 2.89E-100 \| 6.14E-99 \| \| ENSG00000166272 \| WBP1L \| -1.14 \| 4.40E-100 \| 9.33E-99 \| \| ENSG00000166272 \| CYP17A1-AS1 \| -1.14 \| 4.40E-100 \| 9.33E-99 \| \| ENSG00000119943 \| PYROXD2 \| -3.42 \| 4.61E-100 \| 9.77E-99 \| \| ENSG00000170776 \| AKAP13 \| 1.11 \| 4.89E-100 \| 1.04E-98 \| \| ENSG00000179218 \| CALR \| -0.77 \| 5.41E-100 \| 1.14E-98 \| \| ENSG00000087266 \| SH3BP2 \| 0.72 \| 6.73E-100 \| 1.42E-98 \| \| ENSG00000164211 \| STARD4 \| 1.26 \| 9.90E-100 \| 2.09E-98 \| \| ENSG00000168016 \| TRANK1 \| -2.01 \| 1.11E-99 \| 2.33E-98 \| \| ENSG00000197696 \| NMB \| -1.97 \| 5.31E-99 \| 1.12E-97 \| \| ENSG00000138172 \| CALHM2 \| -1.16 \| 9.15E-99 \| 1.92E-97 \| \| ENSG00000105011 \| ASF1B \| -2.09 \| 9.40E-99 \| 1.97E-97 \| \| ENSG00000047597 \| XK \| -3.16 \| 9.56E-99 \| 2.00E-97 \| \| ENSG00000164023 \| SGMS2 \| 2.38 \| 1.45E-98 \| 3.04E-97 \| \| ENSG00000166106 \| ADAMTS15 \| -1.32 \| 1.52E-98 \| 3.18E-97 \| \| ENSG00000167815 \| PRDX2 \| -1.26 \| 1.99E-98 \| 4.14E-97 \| \| ENSG00000159842 \| ABR \| 0.65 \| 2.65E-98 \| 5.53E-97 \| \| ENSG00000124006 \| OBSL1 \| -1.91 \| 4.09E-98 \| 8.51E-97 \| \| ENSG00000105357 \| MYH14 \| 1.70 \| 1.22E-97 \| 2.53E-96 \| \| ENSG00000131323 \| TRAF3 \| 0.93 \| 1.42E-97 \| 2.95E-96 \| \| ENSG00000156970 \| BUB1B \| -2.37 \| 2.53E-97 \| 5.24E-96 \| \| ENSG00000090382 \| LYZ \| -3.26 \| 2.67E-97 \| 5.52E-96 \| \| ENSG00000118418 \| HMGN3 \| -1.44 \| 3.11E-97 \| 6.43E-96 \| \| ENSG00000112406 \| HECA \| 0.82 \| 3.81E-97 \| 7.85E-96 \| \| ENSG00000069275 \| NUCKS1 \| -0.71 \| 3.96E-97 \| 8.17E-96 \| \| ENSG00000185896 \| LAMP1 \| -0.72 \| 7.85E-97 \| 1.61E-95 \| \| ENSG00000134853 \| PDGFRA \| 1.90 \| 1.12E-96 \| 2.30E-95 \| \| ENSG00000173166 \| RAPH1 \| 1.17 \| 1.33E-96 \| 2.73E-95 \| \| ENSG00000127564 \| PKMYT1 \| -2.13 \| 2.21E-96 \| 4.53E-95 \| \| ENSG00000143774 \| GUK1 \| -0.87 \| 2.36E-96 \| 4.83E-95 \| \| ENSG00000145730 \| PAM \| 0.95 \| 2.36E-96 \| 4.83E-95 \| \| ENSG00000067798 \| NAV3 \| 2.21 \| 2.55E-96 \| 5.21E-95 \| \| ENSG00000137869 \| CYP19A1 \| -1.24 \| 1.00E-95 \| 2.04E-94 \| \| ENSG00000134917 \| ADAMTS8 \| -2.54 \| 1.20E-95 \| 2.45E-94 \| \| ENSG00000135766 \| EGLN1 \| -1.15 \| 1.92E-95 \| 3.91E-94 \| \| ENSG00000183160 \| TMEM119 \| 2.13 \| 1.97E-95 \| 4.00E-94 \| \| ENSG00000100300 \| TSPO \| -1.36 \| 2.14E-95 \| 4.35E-94 \| \| ENSG00000147381 \| MAGEA4 \| -3.95 \| 2.26E-95 \| 4.58E-94 \| \| ENSG00000198408 \| OGA \| -1.03 \| 2.30E-95 \| 4.64E-94 \| \| ENSG00000171603 \| CLSTN1 \| -0.71 \| 5.48E-95 \| 1.11E-93 \| \| ENSG00000103642 \| LACTB \| 1.15 \| 7.43E-95 \| 1.50E-93 \| \| ENSG00000113657 \| DPYSL3 \| 2.23 \| 1.23E-94 \| 2.47E-93 \| \| ENSG00000127838 \| PNKD \| 0.74 \| 1.32E-94 \| 2.66E-93 \| \| ENSG00000181444 \| ZNF467 \| -3.23 \| 2.08E-94 \| 4.17E-93 \| \| ENSG00000096433 \| ITPR3 \| 1.70 \| 2.78E-94 \| 5.58E-93 \| \| ENSG00000131759 \| RARA \| 0.90 \| 2.87E-94 \| 5.74E-93 \| \| ENSG00000159216 \| RUNX1 \| 0.80 \| 3.10E-94 \| 6.19E-93 \| \| ENSG00000159216 \| LOC100506403 \| 0.80 \| 3.10E-94 \| 6.19E-93 \| \| ENSG00000006025 \| OSBPL7 \| -2.45 \| 3.89E-94 \| 7.77E-93 \| \| ENSG00000058091 \| CDK14 \| 1.44 \| 4.06E-94 \| 8.10E-93 \| \| ENSG00000213753 \| CENPBD1P1 \| 0.99 \| 7.22E-94 \| 1.44E-92 \| \| ENSG00000038945 \| MSR1 \| -0.71 \| 7.90E-94 \| 1.57E-92 \| \| ENSG00000179869 \| ABCA13 \| 2.59 \| 9.09E-94 \| 1.81E-92 \| \| ENSG00000162430 \| SELENON \| -0.76 \| 9.18E-94 \| 1.82E-92 \| \| ENSG00000146918 \| NCAPG2 \| -1.56 \| 1.06E-93 \| 2.10E-92 \| \| ENSG00000022267 \| FHL1 \| -2.01 \| 1.52E-93 \| 3.01E-92 \| \| ENSG00000130592 \| LSP1 \| -2.75 \| 1.65E-93 \| 3.26E-92 \| \| ENSG00000189067 \| LITAF \| -1.25 \| 1.87E-93 \| 3.69E-92 \| \| ENSG00000140564 \| FURIN \| 0.67 \| 2.13E-93 \| 4.20E-92 \| \| ENSG00000103335 \| PIEZO1 \| -0.89 \| 2.54E-93 \| 4.99E-92 \| \| ENSG00000126759 \| CFP \| -2.76 \| 2.66E-93 \| 5.22E-92 \| \| ENSG00000170500 \| LONRF2 \| -3.97 \| 3.16E-93 \| 6.21E-92 \| \| ENSG00000183337 \| BCOR \| 0.68 \| 3.28E-93 \| 6.44E-92 \| \| ENSG00000014641 \| MDH1 \| -1.01 \| 3.51E-93 \| 6.87E-92 \| \| ENSG00000120594 \| PLXDC2 \| -0.89 \| 1.10E-92 \| 2.16E-91 \| \| ENSG00000168439 \| STIP1 \| 0.61 \| 1.26E-92 \| 2.47E-91 \| \| ENSG00000170627 \| GTSF1 \| -1.48 \| 1.67E-92 \| 3.26E-91 \| \| ENSG00000146072 \| TNFRSF21 \| -0.94 \| 2.46E-92 \| 4.79E-91 \| \| ENSG00000134028 \| ADAMDEC1 \| 1.22 \| 2.74E-92 \| 5.33E-91 \| \| ENSG00000119508 \| NR4A3 \| 3.39 \| 3.85E-92 \| 7.47E-91 \| \| ENSG00000143801 \| PSEN2 \| -1.08 \| 7.00E-92 \| 1.36E-90 \| \| ENSG00000089127 \| OAS1 \| -2.78 \| 1.24E-91 \| 2.41E-90 \| \| ENSG00000221963 \| APOL6 \| -1.82 \| 2.16E-91 \| 4.18E-90 \| \| ENSG00000166228 \| PCBD1 \| -1.50 \| 2.21E-91 \| 4.26E-90 \| \| ENSG00000095139 \| ARCN1 \| 0.63 \| 2.48E-91 \| 4.80E-90 \| \| ENSG00000085871 \| MGST2 \| -1.42 \| 3.83E-91 \| 7.39E-90 \| \| ENSG00000178951 \| ZBTB7A \| 0.74 \| 6.39E-91 \| 1.23E-89 \| \| ENSG00000203667 \| COX20 \| -1.24 \| 8.61E-91 \| 1.66E-89 \| \| ENSG00000151135 \| TMEM263 \| 1.82 \| 9.32E-91 \| 1.79E-89 \| \| ENSG00000165868 \| HSPA12A \| 1.90 \| 4.57E-90 \| 8.77E-89 \| \| ENSG00000169919 \| GUSB \| -1.04 \| 7.00E-90 \| 1.34E-88 \| \| ENSG00000163359 \| COL6A3 \| -2.74 \| 7.26E-90 \| 1.39E-88 \| \| ENSG00000158517 \| NCF1 \| 2.07 \| 8.41E-90 \| 1.61E-88 \| \| ENSG00000145349 \| CAMK2D \| -1.28 \| 1.20E-89 \| 2.30E-88 \| \| ENSG00000100342 \| APOL1 \| -4.18 \| 1.33E-89 \| 2.54E-88 \| \| ENSG00000038382 \| TRIO \| 1.62 \| 2.37E-89 \| 4.51E-88 \| \| ENSG00000085563 \| ABCB1 \| 1.36 \| 2.38E-89 \| 4.53E-88 \| \| ENSG00000130309 \| COLGALT1 \| 0.60 \| 2.72E-89 \| 5.16E-88 \| \| ENSG00000152760 \| TCTEX1D1 \| 0.95 \| 4.80E-89 \| 9.11E-88 \| \| ENSG00000104998 \| IL27RA \| -2.59 \| 6.59E-89 \| 1.25E-87 \| \| ENSG00000072571 \| HMMR \| -3.08 \| 7.28E-89 \| 1.38E-87 \| \| ENSG00000128594 \| LRRC4 \| -1.24 \| 7.66E-89 \| 1.45E-87 \| \| ENSG00000007384 \| RHBDF1 \| 0.83 \| 7.91E-89 \| 1.49E-87 \| \| ENSG00000197321 \| SVIL \| 1.27 \| 1.27E-88 \| 2.39E-87 \| \| ENSG00000159314 \| ARHGAP27 \| -0.86 \| 2.40E-88 \| 4.52E-87 \| \| ENSG00000169926 \| KLF13 \| 0.63 \| 6.01E-88 \| 1.13E-86 \| \| ENSG00000169926 \| LOC105370939 \| 0.63 \| 6.01E-88 \| 1.13E-86 \| \| ENSG00000145287 \| PLAC8 \| -3.90 \| 6.16E-88 \| 1.16E-86 \| \| ENSG00000171617 \| ENC1 \| -1.70 \| 8.13E-88 \| 1.53E-86 \| \| ENSG00000120899 \| PTK2B \| -0.67 \| 8.94E-88 \| 1.68E-86 \| \| ENSG00000052802 \| MSMO1 \| 1.16 \| 1.90E-87 \| 3.56E-86 \| \| ENSG00000002586 \| CD99 \| -0.75 \| 1.92E-87 \| 3.59E-86 \| \| ENSG00000102226 \| USP11 \| 0.94 \| 2.18E-87 \| 4.07E-86 \| \| ENSG00000143815 \| LBR \| -1.03 \| 2.23E-87 \| 4.15E-86 \| \| ENSG00000175505 \| CLCF1 \| 2.40 \| 2.94E-87 \| 5.47E-86 \| \| ENSG00000184384 \| MAML2 \| 0.82 \| 3.15E-87 \| 5.85E-86 \| \| ENSG00000055208 \| TAB2 \| 0.65 \| 3.26E-87 \| 6.05E-86 \| \| ENSG00000141526 \| SLC16A3 \| 0.67 \| 8.59E-87 \| 1.59E-85 \| \| ENSG00000239998 \| LILRA2 \| -3.85 \| 1.72E-86 \| 3.19E-85 \| \| ENSG00000186480 \| INSIG1 \| 0.94 \| 2.00E-86 \| 3.70E-85 \| \| ENSG00000110455 \| ACCS \| -1.55 \| 2.17E-86 \| 4.00E-85 \| \| ENSG00000173744 \| AGFG1 \| 0.84 \| 2.91E-86 \| 5.36E-85 \| \| ENSG00000112697 \| TMEM30A \| -0.77 \| 3.38E-86 \| 6.22E-85 \| \| ENSG00000161011 \| SQSTM1 \| 0.77 \| 3.52E-86 \| 6.47E-85 \| \| ENSG00000188486 \| H2AFX \| -0.97 \| 4.86E-86 \| 8.94E-85 \| \| ENSG00000143222 \| UFC1 \| -0.88 \| 5.69E-86 \| 1.04E-84 \| \| ENSG00000184922 \| FMNL1 \| 0.75 \| 6.49E-86 \| 1.19E-84 \| \| ENSG00000005844 \| ITGAL \| -0.84 \| 9.02E-86 \| 1.65E-84 \| \| ENSG00000131981 \| LGALS3 \| -1.24 \| 1.25E-85 \| 2.28E-84 \| \| ENSG00000183853 \| KIRREL1 \| -1.54 \| 1.93E-85 \| 3.52E-84 \| \| ENSG00000155849 \| ELMO1 \| -1.42 \| 2.51E-85 \| 4.57E-84 \| \| ENSG00000106066 \| CPVL \| -4.72 \| 2.71E-85 \| 4.93E-84 \| \| ENSG00000170006 \| TMEM154 \| -3.08 \| 2.82E-85 \| 5.12E-84 \| \| ENSG00000119900 \| OGFRL1 \| 0.87 \| 5.38E-85 \| 9.76E-84 \| \| ENSG00000131389 \| SLC6A6 \| 0.73 \| 5.58E-85 \| 1.01E-83 \| \| ENSG00000171813 \| PWWP2B \| -2.57 \| 5.91E-85 \| 1.07E-83 \| \| ENSG00000179761 \| PIPOX \| -2.52 \| 9.41E-85 \| 1.70E-83 \| \| ENSG00000123684 \| LPGAT1 \| -1.13 \| 1.06E-84 \| 1.92E-83 \| \| ENSG00000144481 \| TRPM8 \| -1.80 \| 1.08E-84 \| 1.95E-83 \| \| ENSG00000140479 \| PCSK6 \| -0.81 \| 1.21E-84 \| 2.19E-83 \| \| ENSG00000152484 \| USP12 \| 1.02 \| 1.31E-84 \| 2.36E-83 \| \| ENSG00000204482 \| LST1 \| 0.89 \| 1.38E-84 \| 2.48E-83 \| \| ENSG00000164951 \| PDP1 \| 1.09 \| 1.45E-84 \| 2.61E-83 \| \| ENSG00000092969 \| TGFB2 \| 2.17 \| 1.67E-84 \| 2.99E-83 \| \| ENSG00000132669 \| RIN2 \| -1.21 \| 1.72E-84 \| 3.07E-83 \| \| ENSG00000100714 \| MTHFD1 \| -1.01 \| 2.04E-84 \| 3.65E-83 \| \| ENSG00000143507 \| DUSP10 \| 0.90 \| 3.08E-84 \| 5.51E-83 \| \| ENSG00000153823 \| PID1 \| 2.17 \| 4.28E-84 \| 7.63E-83 \| \| ENSG00000104783 \| KCNN4 \| 0.60 \| 6.96E-84 \| 1.24E-82 \| \| ENSG00000182985 \| CADM1 \| -1.13 \| 7.71E-84 \| 1.37E-82 \| \| ENSG00000160014 \| CALM3 \| -0.61 \| 9.05E-84 \| 1.61E-82 \| \| ENSG00000168081 \| PNOC \| -3.37 \| 1.18E-83 \| 2.10E-82 \| \| ENSG00000011426 \| ANLN \| -1.52 \| 1.66E-83 \| 2.94E-82 \| \| ENSG00000129353 \| SLC44A2 \| 1.29 \| 1.72E-83 \| 3.04E-82 \| \| ENSG00000167513 \| CDT1 \| -1.83 \| 1.89E-83 \| 3.35E-82 \| \| ENSG00000147724 \| FAM135B \| 1.10 \| 1.99E-83 \| 3.52E-82 \| \| ENSG00000146670 \| CDCA5 \| -2.13 \| 2.53E-83 \| 4.46E-82 \| \| ENSG00000233695 \|  \| -4.79 \| 2.98E-83 \| 5.25E-82 \| \| ENSG00000128340 \| RAC2 \| 0.63 \| 4.15E-83 \| 7.31E-82 \| \| ENSG00000185033 \| SEMA4B \| -1.47 \| 5.40E-83 \| 9.49E-82 \| \| ENSG00000099290 \| WASHC2A \| -1.34 \| 7.17E-83 \| 1.26E-81 \| \| ENSG00000148288 \| GBGT1 \| -2.61 \| 8.08E-83 \| 1.42E-81 \| \| ENSG00000184156 \| KCNQ3 \| -1.84 \| 1.17E-82 \| 2.04E-81 \| \| ENSG00000011028 \| MRC2 \| -0.82 \| 1.39E-82 \| 2.43E-81 \| \| ENSG00000196502 \| SULT1A1 \| -2.82 \| 1.55E-82 \| 2.71E-81 \| \| ENSG00000043591 \| ADRB1 \| -3.26 \| 2.13E-82 \| 3.72E-81 \| \| ENSG00000126457 \| PRMT1 \| 0.77 \| 2.19E-82 \| 3.82E-81 \| \| ENSG00000069020 \| MAST4 \| 1.54 \| 2.31E-82 \| 4.03E-81 \| \| ENSG00000010818 \| HIVEP2 \| 2.64 \| 2.94E-82 \| 5.12E-81 \| \| ENSG00000168779 \| SHOX2 \| -1.06 \| 3.33E-82 \| 5.79E-81 \| \| ENSG00000165891 \| E2F7 \| 1.31 \| 4.00E-82 \| 6.94E-81 \| \| ENSG00000172164 \| SNTB1 \| 0.69 \| 5.03E-82 \| 8.72E-81 \| \| ENSG00000012660 \| ELOVL5 \| 0.78 \| 1.25E-81 \| 2.16E-80 \| \| ENSG00000103647 \| CORO2B \| 1.11 \| 1.26E-81 \| 2.18E-80 \| \| ENSG00000123360 \| PDE1B \| -1.45 \| 1.31E-81 \| 2.27E-80 \| \| ENSG00000187231 \| SESTD1 \| 1.21 \| 1.44E-81 \| 2.49E-80 \| \| ENSG00000087589 \| CASS4 \| -1.44 \| 1.85E-81 \| 3.19E-80 \| \| ENSG00000143891 \| GALM \| -1.58 \| 1.88E-81 \| 3.24E-80 \| \| ENSG00000164402 \| 08-Sep \| -1.51 \| 2.00E-81 \| 3.44E-80 \| \| ENSG00000182054 \| IDH2 \| -0.77 \| 2.05E-81 \| 3.52E-80 \| \| ENSG00000185432 \| METTL7A \| 0.95 \| 3.55E-81 \| 6.09E-80 \| \| ENSG00000148660 \| CAMK2G \| -2.03 \| 4.44E-81 \| 7.62E-80 \| \| ENSG00000276023 \| DUSP14 \| 0.98 \| 4.95E-81 \| 8.48E-80 \| \| ENSG00000151702 \| FLI1 \| -0.85 \| 5.01E-81 \| 8.58E-80 \| \| ENSG00000109805 \| NCAPG \| -1.87 \| 5.44E-81 \| 9.30E-80 \| \| ENSG00000135424 \| ITGA7 \| -2.09 \| 5.61E-81 \| 9.57E-80 \| \| ENSG00000234741 \| GAS5 \| -1.10 \| 5.97E-81 \| 1.02E-79 \| \| ENSG00000185482 \| STAC3 \| -2.10 \| 8.26E-81 \| 1.41E-79 \| \| ENSG00000137936 \| BCAR3 \| -2.01 \| 8.45E-81 \| 1.44E-79 \| \| ENSG00000137936 \| MIG7 \| -2.01 \| 8.45E-81 \| 1.44E-79 \| \| ENSG00000139645 \| ANKRD52 \| 0.66 \| 8.96E-81 \| 1.52E-79 \| \| ENSG00000198363 \| ASPH \| 0.83 \| 2.65E-80 \| 4.50E-79 \| \| ENSG00000156860 \| FBRS \| 0.76 \| 2.75E-80 \| 4.66E-79 \| \| ENSG00000019582 \| CD74 \| -1.03 \| 3.15E-80 \| 5.34E-79 \| \| ENSG00000196937 \| FAM3C \| 1.12 \| 4.49E-80 \| 7.59E-79 \| \| ENSG00000151503 \| NCAPD3 \| 0.72 \| 4.69E-80 \| 7.93E-79 \| \| ENSG00000116016 \| EPAS1 \| -1.81 \| 5.69E-80 \| 9.60E-79 \| \| ENSG00000250479 \| CHCHD10 \| -1.02 \| 6.02E-80 \| 1.02E-78 \| \| ENSG00000187210 \| GCNT1 \| -1.90 \| 7.00E-80 \| 1.18E-78 \| \| ENSG00000143882 \| ATP6V1C2 \| -1.24 \| 8.95E-80 \| 1.51E-78 \| \| ENSG00000132589 \| FLOT2 \| -0.89 \| 9.76E-80 \| 1.64E-78 \| \| ENSG00000171467 \| ZNF318 \| 0.88 \| 1.01E-79 \| 1.70E-78 \| \| ENSG00000196159 \| FAT4 \| 4.87 \| 1.05E-79 \| 1.76E-78 \| \| ENSG00000122966 \| CIT \| -2.52 \| 1.44E-79 \| 2.42E-78 \| \| ENSG00000112531 \| QKI \| 0.62 \| 1.89E-79 \| 3.17E-78 \| \| ENSG00000164684 \| ZNF704 \| -2.83 \| 2.16E-79 \| 3.62E-78 \| \| ENSG00000197448 \| GSTK1 \| -0.88 \| 2.44E-79 \| 4.08E-78 \| \| ENSG00000117266 \| CDK18 \| -2.26 \| 4.05E-79 \| 6.75E-78 \| \| ENSG00000274286 \| ADRA2B \| -3.75 \| 4.72E-79 \| 7.86E-78 \| \| ENSG00000094804 \| CDC6 \| -1.83 \| 5.20E-79 \| 8.65E-78 \| \| ENSG00000229619 \| MBNL1-AS1 \| -1.66 \| 5.21E-79 \| 8.66E-78 \| \| ENSG00000075624 \| ACTB \| -0.66 \| 5.89E-79 \| 9.77E-78 \| \| ENSG00000082458 \| DLG3 \| -1.75 \| 5.96E-79 \| 9.89E-78 \| \| ENSG00000158473 \| CD1D \| -1.92 \| 7.14E-79 \| 1.18E-77 \| \| ENSG00000070495 \| JMJD6 \| 1.15 \| 9.54E-79 \| 1.58E-77 \| \| ENSG00000155465 \| SLC7A7 \| -0.93 \| 1.17E-78 \| 1.93E-77 \| \| ENSG00000173193 \| PARP14 \| -1.57 \| 2.30E-78 \| 3.80E-77 \| \| ENSG00000137693 \| YAP1 \| 1.47 \| 2.40E-78 \| 3.96E-77 \| \| ENSG00000074181 \| NOTCH3 \| 3.65 \| 2.81E-78 \| 4.63E-77 \| \| ENSG00000134072 \| CAMK1 \| -0.75 \| 3.04E-78 \| 5.00E-77 \| \| ENSG00000124702 \| KLHDC3 \| -0.73 \| 3.06E-78 \| 5.02E-77 \| \| ENSG00000183726 \| TMEM50A \| 0.69 \| 3.24E-78 \| 5.32E-77 \| \| ENSG00000111726 \| CMAS \| 1.00 \| 3.53E-78 \| 5.78E-77 \| \| ENSG00000166922 \| SCG5 \| 1.90 \| 3.66E-78 \| 5.99E-77 \| \| ENSG00000110628 \| SLC22A18 \| -1.47 \| 5.24E-78 \| 8.58E-77 \| \| ENSG00000165915 \| SLC39A13 \| 0.78 \| 6.42E-78 \| 1.05E-76 \| \| ENSG00000135362 \| PRR5L \| -1.54 \| 6.48E-78 \| 1.06E-76 \| \| ENSG00000166750 \| SLFN5 \| 1.01 \| 7.31E-78 \| 1.19E-76 \| \| ENSG00000000938 \| FGR \| 0.85 \| 8.05E-78 \| 1.31E-76 \| \| ENSG00000120910 \| PPP3CC \| 1.10 \| 9.62E-78 \| 1.57E-76 \| \| ENSG00000109685 \| NSD2 \| -0.97 \| 1.18E-77 \| 1.92E-76 \| \| ENSG00000153250 \| RBMS1 \| 0.83 \| 1.41E-77 \| 2.29E-76 \| \| ENSG00000179542 \| SLITRK4 \| 1.06 \| 2.72E-77 \| 4.41E-76 \| \| ENSG00000099875 \| MKNK2 \| 0.70 \| 3.25E-77 \| 5.27E-76 \| \| ENSG00000177409 \| SAMD9L \| -2.91 \| 4.41E-77 \| 7.14E-76 \| \| ENSG00000166128 \| RAB8B \| 0.89 \| 5.23E-77 \| 8.46E-76 \| \| ENSG00000198113 \| TOR4A \| -0.91 \| 5.49E-77 \| 8.86E-76 \| \| ENSG00000186431 \| FCAR \| 1.57 \| 5.61E-77 \| 9.05E-76 \| \| ENSG00000074370 \| ATP2A3 \| 0.98 \| 5.99E-77 \| 9.65E-76 \| \| ENSG00000160216 \| AGPAT3 \| -0.84 \| 6.77E-77 \| 1.09E-75 \| \| ENSG00000272841 \|  \| 1.48 \| 7.45E-77 \| 1.20E-75 \| \| ENSG00000005108 \| THSD7A \| -1.86 \| 7.62E-77 \| 1.23E-75 \| \| ENSG00000169258 \| GPRIN1 \| 1.35 \| 9.07E-77 \| 1.46E-75 \| \| ENSG00000158186 \| MRAS \| 0.86 \| 9.89E-77 \| 1.59E-75 \| \| ENSG00000198829 \| SUCNR1 \| -2.37 \| 1.08E-76 \| 1.74E-75 \| \| ENSG00000180773 \| SLC36A4 \| 1.20 \| 1.23E-76 \| 1.96E-75 \| \| ENSG00000159202 \| UBE2Z \| 0.69 \| 1.41E-76 \| 2.26E-75 \| \| ENSG00000160703 \| NLRX1 \| -1.27 \| 1.90E-76 \| 3.04E-75 \| \| ENSG00000110719 \| TCIRG1 \| 0.70 \| 1.99E-76 \| 3.17E-75 \| \| ENSG00000179889 \| PDXDC1 \| 0.79 \| 2.06E-76 \| 3.28E-75 \| \| ENSG00000179889 \| LOC102724985 \| 0.79 \| 2.06E-76 \| 3.28E-75 \| \| ENSG00000268858 \|  \| 1.07 \| 2.27E-76 \| 3.61E-75 \| \| ENSG00000182831 \| C16orf72 \| 0.81 \| 2.58E-76 \| 4.10E-75 \| \| ENSG00000110934 \| BIN2 \| -0.85 \| 3.33E-76 \| 5.29E-75 \| \| ENSG00000171246 \| NPTX1 \| -3.16 \| 3.46E-76 \| 5.49E-75 \| \| ENSG00000165175 \| MID1IP1 \| 0.83 \| 6.86E-76 \| 1.09E-74 \| \| ENSG00000054277 \| OPN3 \| 1.65 \| 8.65E-76 \| 1.37E-74 \| \| ENSG00000153208 \| MERTK \| -0.82 \| 1.05E-75 \| 1.67E-74 \| \| ENSG00000131149 \| GSE1 \| -1.43 \| 1.25E-75 \| 1.97E-74 \| \| ENSG00000091490 \| SEL1L3 \| -1.32 \| 1.36E-75 \| 2.14E-74 \| \| ENSG00000112297 \| CRYBG1 \| -3.63 \| 1.36E-75 \| 2.15E-74 \| \| ENSG00000198743 \| SLC5A3 \| 1.56 \| 1.56E-75 \| 2.45E-74 \| \| ENSG00000166068 \| SPRED1 \| -1.74 \| 1.63E-75 \| 2.56E-74 \| \| ENSG00000142185 \| TRPM2 \| -0.76 \| 2.01E-75 \| 3.16E-74 \| \| ENSG00000133835 \| HSD17B4 \| -1.04 \| 2.11E-75 \| 3.31E-74 \| \| ENSG00000136560 \| TANK \| 0.93 \| 2.55E-75 \| 4.00E-74 \| \| ENSG00000153046 \| CDYL \| 1.18 \| 2.84E-75 \| 4.45E-74 \| \| ENSG00000099991 \| CABIN1 \| -0.99 \| 3.90E-75 \| 6.11E-74 \| \| ENSG00000126767 \| ELK1 \| -2.17 \| 6.49E-75 \| 1.01E-73 \| \| ENSG00000186185 \| KIF18B \| -2.57 \| 6.90E-75 \| 1.08E-73 \| \| ENSG00000113716 \| HMGXB3 \| 0.73 \| 7.28E-75 \| 1.14E-73 \| \| ENSG00000254470 \| AP5B1 \| 0.87 \| 7.96E-75 \| 1.24E-73 \| \| ENSG00000125726 \| CD70 \| -3.38 \| 1.06E-74 \| 1.66E-73 \| \| ENSG00000104903 \| LYL1 \| -1.90 \| 1.09E-74 \| 1.69E-73 \| \| ENSG00000154146 \| NRGN \| -1.36 \| 1.46E-74 \| 2.27E-73 \| \| ENSG00000127334 \| DYRK2 \| 0.91 \| 3.06E-74 \| 4.76E-73 \| \| ENSG00000155368 \| DBI \| -0.77 \| 3.08E-74 \| 4.78E-73 \| \| ENSG00000204525 \| HLA-C \| -0.75 \| 4.61E-74 \| 7.15E-73 \| \| ENSG00000171488 \| LRRC8C \| -1.08 \| 5.37E-74 \| 8.31E-73 \| \| ENSG00000080503 \| SMARCA2 \| 0.78 \| 5.62E-74 \| 8.70E-73 \| \| ENSG00000115170 \| ACVR1 \| 1.30 \| 5.78E-74 \| 8.93E-73 \| \| ENSG00000116514 \| RNF19B \| 1.12 \| 6.75E-74 \| 1.04E-72 \| \| ENSG00000136379 \| ABHD17C \| 1.08 \| 7.34E-74 \| 1.13E-72 \| \| ENSG00000075826 \| SEC31B \| -1.94 \| 7.38E-74 \| 1.14E-72 \| \| ENSG00000155380 \| SLC16A1 \| -1.10 \| 8.82E-74 \| 1.36E-72 \| \| ENSG00000119681 \| LTBP2 \| 1.03 \| 1.18E-73 \| 1.82E-72 \| \| ENSG00000146094 \| DOK3 \| 0.76 \| 1.47E-73 \| 2.26E-72 \| \| ENSG00000134668 \| SPOCD1 \| -0.78 \| 1.75E-73 \| 2.69E-72 \| \| ENSG00000247774 \| PCED1B-AS1 \| -2.09 \| 1.78E-73 \| 2.73E-72 \| \| ENSG00000213551 \| DNAJC9 \| -1.91 \| 1.87E-73 \| 2.87E-72 \| \| ENSG00000054523 \| KIF1B \| 0.96 \| 2.17E-73 \| 3.33E-72 \| \| ENSG00000163565 \| IFI16 \| -1.04 \| 3.08E-73 \| 4.70E-72 \| \| ENSG00000010671 \| BTK \| -1.06 \| 3.38E-73 \| 5.16E-72 \| \| ENSG00000166165 \| CKB \| 1.57 \| 3.42E-73 \| 5.22E-72 \| \| ENSG00000148484 \| RSU1 \| -0.85 \| 5.88E-73 \| 8.96E-72 \| \| ENSG00000107736 \| CDH23 \| -1.35 \| 1.01E-72 \| 1.53E-71 \| \| ENSG00000119669 \| IRF2BPL \| 0.75 \| 1.15E-72 \| 1.74E-71 \| \| ENSG00000142197 \| DOP1B \| -0.91 \| 1.72E-72 \| 2.61E-71 \| \| ENSG00000112742 \| TTK \| -2.58 \| 2.67E-72 \| 4.05E-71 \| \| ENSG00000103187 \| COTL1 \| -0.63 \| 2.71E-72 \| 4.10E-71 \| \| ENSG00000123473 \| STIL \| -1.81 \| 3.23E-72 \| 4.88E-71 \| \| ENSG00000140396 \| NCOA2 \| -0.87 \| 3.68E-72 \| 5.56E-71 \| \| ENSG00000271605 \| MILR1 \| -1.17 \| 4.21E-72 \| 6.36E-71 \| \| ENSG00000136193 \| SCRN1 \| -3.76 \| 4.75E-72 \| 7.16E-71 \| \| ENSG00000011201 \| ANOS1 \| -2.26 \| 5.43E-72 \| 8.19E-71 \| \| ENSG00000177426 \| TGIF1 \| 1.03 \| 5.87E-72 \| 8.84E-71 \| \| ENSG00000137497 \| NUMA1 \| -0.93 \| 7.56E-72 \| 1.14E-70 \| \| ENSG00000156113 \| KCNMA1 \| -1.88 \| 7.91E-72 \| 1.19E-70 \| \| ENSG00000137494 \| ANKRD42 \| 1.26 \| 1.45E-71 \| 2.18E-70 \| \| ENSG00000025039 \| RRAGD \| -1.37 \| 3.39E-71 \| 5.07E-70 \| \| ENSG00000054793 \| ATP9A \| 0.89 \| 4.06E-71 \| 6.07E-70 \| \| ENSG00000109756 \| RAPGEF2 \| 1.18 \| 5.98E-71 \| 8.94E-70 \| \| ENSG00000138180 \| CEP55 \| -3.17 \| 6.17E-71 \| 9.22E-70 \| \| ENSG00000171724 \| VAT1L \| -2.53 \| 6.67E-71 \| 9.95E-70 \| \| ENSG00000104738 \| MCM4 \| -0.86 \| 8.17E-71 \| 1.22E-69 \| \| ENSG00000126432 \| PRDX5 \| -0.97 \| 8.32E-71 \| 1.24E-69 \| \| ENSG00000197249 \| SERPINA1 \| -1.24 \| 9.11E-71 \| 1.36E-69 \| \| ENSG00000112511 \| PHF1 \| 0.89 \| 1.54E-70 \| 2.29E-69 \| \| ENSG00000105355 \| PLIN3 \| 0.64 \| 1.62E-70 \| 2.41E-69 \| \| ENSG00000168918 \| INPP5D \| -0.65 \| 1.67E-70 \| 2.48E-69 \| \| ENSG00000244242 \| IFITM10 \| 2.81 \| 1.71E-70 \| 2.53E-69 \| \| ENSG00000161642 \| ZNF385A \| 0.70 \| 1.86E-70 \| 2.75E-69 \| \| ENSG00000102290 \| PCDH11X \| 3.02 \| 2.30E-70 \| 3.40E-69 \| \| ENSG00000186283 \| TOR3A \| -1.03 \| 2.30E-70 \| 3.40E-69 \| \| ENSG00000111716 \| LDHB \| -0.71 \| 2.73E-70 \| 4.03E-69 \| \| ENSG00000135334 \| AKIRIN2 \| 0.67 \| 2.96E-70 \| 4.37E-69 \| \| ENSG00000121152 \| NCAPH \| -2.40 \| 3.27E-70 \| 4.81E-69 \| \| ENSG00000137161 \| CNPY3 \| -0.78 \| 3.82E-70 \| 5.62E-69 \| \| ENSG00000114757 \| PEX5L \| 2.74 \| 4.20E-70 \| 6.17E-69 \| \| ENSG00000171314 \| PGAM1 \| -0.90 \| 4.44E-70 \| 6.51E-69 \| \| ENSG00000081014 \| AP4E1 \| 1.18 \| 4.58E-70 \| 6.72E-69 \| \| ENSG00000068697 \| LAPTM4A \| 0.74 \| 4.86E-70 \| 7.11E-69 \| \| ENSG00000198680 \| TUSC1 \| 1.08 \| 6.27E-70 \| 9.17E-69 \| \| ENSG00000138135 \| CH25H \| 2.58 \| 6.32E-70 \| 9.24E-69 \| \| ENSG00000136141 \| LRCH1 \| 0.97 \| 6.55E-70 \| 9.57E-69 \| \| ENSG00000065054 \| SLC9A3R2 \| -2.75 \| 9.55E-70 \| 1.39E-68 \| \| ENSG00000064201 \| TSPAN32 \| -2.61 \| 1.16E-69 \| 1.69E-68 \| \| ENSG00000175455 \| CCDC14 \| -1.21 \| 1.26E-69 \| 1.84E-68 \| \| ENSG00000196781 \| TLE1 \| 1.68 \| 1.41E-69 \| 2.05E-68 \| \| ENSG00000091436 \| MAP3K20 \| 0.80 \| 1.57E-69 \| 2.29E-68 \| \| ENSG00000163975 \| MELTF \| -1.66 \| 1.64E-69 \| 2.37E-68 \| \| ENSG00000112473 \| SLC39A7 \| 0.67 \| 1.67E-69 \| 2.43E-68 \| \| ENSG00000117226 \| GBP3 \| -3.47 \| 2.05E-69 \| 2.97E-68 \| \| ENSG00000187555 \| USP7 \| 0.65 \| 2.08E-69 \| 3.01E-68 \| \| ENSG00000115935 \| WIPF1 \| -0.76 \| 2.20E-69 \| 3.19E-68 \| \| ENSG00000136108 \| CKAP2 \| -1.32 \| 2.30E-69 \| 3.32E-68 \| \| ENSG00000196182 \| STK40 \| 0.69 \| 2.35E-69 \| 3.39E-68 \| \| ENSG00000188613 \| NANOS1 \| 2.05 \| 2.40E-69 \| 3.46E-68 \| \| ENSG00000104325 \| DECR1 \| -1.02 \| 2.58E-69 \| 3.72E-68 \| \| ENSG00000172661 \| WASHC2C \| -0.96 \| 2.74E-69 \| 3.95E-68 \| \| ENSG00000169223 \| LMAN2 \| -0.72 \| 3.14E-69 \| 4.52E-68 \| \| ENSG00000162493 \| PDPN \| 0.96 \| 3.46E-69 \| 4.97E-68 \| \| ENSG00000150471 \| ADGRL3 \| -2.12 \| 4.44E-69 \| 6.37E-68 \| \| ENSG00000154473 \| BUB3 \| -1.00 \| 7.48E-69 \| 1.07E-67 \| \| ENSG00000184445 \| KNTC1 \| -1.60 \| 8.20E-69 \| 1.18E-67 \| \| ENSG00000117859 \| OSBPL9 \| -0.76 \| 1.31E-68 \| 1.88E-67 \| \| ENSG00000180066 \| C10orf91 \| -2.94 \| 1.52E-68 \| 2.18E-67 \| \| ENSG00000149418 \| ST14 \| -0.77 \| 1.52E-68 \| 2.18E-67 \| \| ENSG00000198053 \| SIRPA \| 0.62 \| 1.62E-68 \| 2.31E-67 \| \| ENSG00000002726 \| AOC1 \| -4.16 \| 1.63E-68 \| 2.32E-67 \| \| ENSG00000177614 \| PGBD5 \| 1.25 \| 1.80E-68 \| 2.56E-67 \| \| ENSG00000073756 \| PTGS2 \| 4.48 \| 2.18E-68 \| 3.10E-67 \| \| ENSG00000106366 \| SERPINE1 \| 2.22 \| 2.21E-68 \| 3.15E-67 \| \| ENSG00000170345 \| FOS \| 0.80 \| 3.88E-68 \| 5.50E-67 \| \| ENSG00000012048 \| BRCA1 \| -1.35 \| 5.05E-68 \| 7.16E-67 \| \| ENSG00000151725 \| CENPU \| -3.07 \| 5.52E-68 \| 7.82E-67 \| \| ENSG00000127948 \| POR \| -1.08 \| 1.19E-67 \| 1.68E-66 \| \| ENSG00000154122 \| ANKH \| 0.67 \| 1.63E-67 \| 2.30E-66 \| \| ENSG00000121310 \| ECHDC2 \| -1.29 \| 1.66E-67 \| 2.34E-66 \| \| ENSG00000217801 \| LOC100288175 \| 0.85 \| 1.87E-67 \| 2.65E-66 \| \| ENSG00000104164 \| BLOC1S6 \| 0.86 \| 2.23E-67 \| 3.14E-66 \| \| ENSG00000135299 \| ANKRD6 \| -2.43 \| 2.54E-67 \| 3.58E-66 \| \| ENSG00000165288 \| BRWD3 \| 1.28 \| 2.66E-67 \| 3.74E-66 \| \| ENSG00000073792 \| IGF2BP2 \| 0.75 \| 2.72E-67 \| 3.82E-66 \| \| ENSG00000082397 \| EPB41L3 \| 1.21 \| 5.68E-67 \| 7.98E-66 \| \| ENSG00000090889 \| KIF4A \| -2.64 \| 5.84E-67 \| 8.21E-66 \| \| ENSG00000171159 \| C9orf16 \| -1.11 \| 7.46E-67 \| 1.05E-65 \| \| ENSG00000232810 \| TNF \| 3.63 \| 7.52E-67 \| 1.05E-65 \| \| ENSG00000146433 \| TMEM181 \| 1.05 \| 9.07E-67 \| 1.27E-65 \| \| ENSG00000251493 \| FOXD1 \| -1.58 \| 9.54E-67 \| 1.34E-65 \| \| ENSG00000152601 \| MBNL1 \| -0.63 \| 1.22E-66 \| 1.71E-65 \| \| ENSG00000130429 \| ARPC1B \| -0.63 \| 1.60E-66 \| 2.23E-65 \| \| ENSG00000197982 \| C1orf122 \| 0.98 \| 1.70E-66 \| 2.38E-65 \| \| ENSG00000156273 \| BACH1 \| 1.00 \| 1.81E-66 \| 2.52E-65 \| \| ENSG00000156273 \| GRIK1-AS2 \| 1.00 \| 1.81E-66 \| 2.52E-65 \| \| ENSG00000168421 \| RHOH \| 2.50 \| 1.90E-66 \| 2.65E-65 \| \| ENSG00000158715 \| SLC45A3 \| -1.19 \| 2.06E-66 \| 2.87E-65 \| \| ENSG00000173848 \| NET1 \| -1.97 \| 2.36E-66 \| 3.28E-65 \| \| ENSG00000132824 \| SERINC3 \| 0.60 \| 2.95E-66 \| 4.10E-65 \| \| ENSG00000174791 \| RIN1 \| 0.95 \| 3.14E-66 \| 4.36E-65 \| \| ENSG00000163823 \| CCR1 \| -1.00 \| 3.20E-66 \| 4.44E-65 \| \| ENSG00000105851 \| PIK3CG \| -1.19 \| 3.21E-66 \| 4.45E-65 \| \| ENSG00000109220 \| CHIC2 \| 1.37 \| 4.76E-66 \| 6.58E-65 \| \| ENSG00000075702 \| WDR62 \| -1.71 \| 4.76E-66 \| 6.59E-65 \| \| ENSG00000178773 \| CPNE7 \| -1.87 \| 4.94E-66 \| 6.83E-65 \| \| ENSG00000113369 \| ARRDC3 \| 0.96 \| 7.39E-66 \| 1.02E-64 \| \| ENSG00000149635 \| OCSTAMP \| 1.46 \| 1.16E-65 \| 1.60E-64 \| \| ENSG00000165678 \| GHITM \| -0.82 \| 1.17E-65 \| 1.62E-64 \| \| ENSG00000168214 \| RBPJ \| 0.76 \| 1.19E-65 \| 1.63E-64 \| \| ENSG00000242498 \| ARPIN \| -3.48 \| 1.23E-65 \| 1.69E-64 \| \| ENSG00000071575 \| TRIB2 \| 1.40 \| 1.23E-65 \| 1.69E-64 \| \| ENSG00000023171 \| GRAMD1B \| -1.42 \| 1.36E-65 \| 1.87E-64 \| \| ENSG00000023171 \| LOC100128242 \| -1.42 \| 1.36E-65 \| 1.87E-64 \| \| ENSG00000110046 \| ATG2A \| 1.01 \| 1.73E-65 \| 2.37E-64 \| \| ENSG00000108518 \| PFN1 \| -0.69 \| 1.76E-65 \| 2.41E-64 \| \| ENSG00000102471 \| NDFIP2 \| 0.95 \| 1.81E-65 \| 2.47E-64 \| \| ENSG00000111052 \| LIN7A \| -4.20 \| 1.81E-65 \| 2.47E-64 \| \| ENSG00000134291 \| TMEM106C \| -2.42 \| 2.61E-65 \| 3.56E-64 \| \| ENSG00000175061 \| LRRC75A-AS1 \| -0.88 \| 2.65E-65 \| 3.61E-64 \| \| ENSG00000137076 \| TLN1 \| 0.67 \| 3.42E-65 \| 4.67E-64 \| \| ENSG00000075218 \| GTSE1 \| -2.56 \| 3.53E-65 \| 4.81E-64 \| \| ENSG00000169762 \| TAPT1 \| 1.09 \| 3.66E-65 \| 4.98E-64 \| \| ENSG00000168264 \| IRF2BP2 \| 0.61 \| 3.94E-65 \| 5.36E-64 \| \| ENSG00000011600 \| TYROBP \| -0.62 \| 4.14E-65 \| 5.63E-64 \| \| ENSG00000171241 \| SHCBP1 \| -2.05 \| 4.79E-65 \| 6.51E-64 \| \| ENSG00000168758 \| SEMA4C \| 0.99 \| 5.16E-65 \| 7.00E-64 \| \| ENSG00000104518 \| GSDMD \| -1.16 \| 5.52E-65 \| 7.49E-64 \| \| ENSG00000255398 \| HCAR3 \| 4.47 \| 6.10E-65 \| 8.26E-64 \| \| ENSG00000173599 \| PC \| 0.92 \| 6.38E-65 \| 8.63E-64 \| \| ENSG00000150961 \| SEC24D \| 0.70 \| 6.80E-65 \| 9.20E-64 \| \| ENSG00000015133 \| CCDC88C \| -1.29 \| 7.10E-65 \| 9.60E-64 \| \| ENSG00000137310 \| TCF19 \| -1.78 \| 9.30E-65 \| 1.25E-63 \| \| ENSG00000023287 \| RB1CC1 \| 0.86 \| 1.36E-64 \| 1.84E-63 \| \| ENSG00000214078 \| CPNE1 \| 0.73 \| 1.47E-64 \| 1.98E-63 \| \| ENSG00000130005 \| GAMT \| -1.39 \| 1.47E-64 \| 1.98E-63 \| \| ENSG00000196562 \| SULF2 \| 1.77 \| 1.54E-64 \| 2.07E-63 \| \| ENSG00000139626 \| ITGB7 \| -0.77 \| 1.82E-64 \| 2.44E-63 \| \| ENSG00000112306 \| RPS12 \| -0.67 \| 2.22E-64 \| 2.98E-63 \| \| ENSG00000165304 \| MELK \| -2.15 \| 2.68E-64 \| 3.59E-63 \| \| ENSG00000115520 \| COQ10B \| 1.12 \| 2.76E-64 \| 3.69E-63 \| \| ENSG00000110717 \| NDUFS8 \| 0.73 \| 2.98E-64 \| 3.98E-63 \| \| ENSG00000116132 \| PRRX1 \| 4.82 \| 3.41E-64 \| 4.57E-63 \| \| ENSG00000162512 \| SDC3 \| -0.76 \| 3.48E-64 \| 4.65E-63 \| \| ENSG00000172845 \| SP3 \| 0.80 \| 3.74E-64 \| 4.99E-63 \| \| ENSG00000125355 \| TMEM255A \| -1.14 \| 4.46E-64 \| 5.95E-63 \| \| ENSG00000111859 \| NEDD9 \| -1.48 \| 4.92E-64 \| 6.55E-63 \| \| ENSG00000117036 \| ETV3 \| 0.91 \| 5.60E-64 \| 7.45E-63 \| \| ENSG00000101972 \| STAG2 \| 0.73 \| 6.11E-64 \| 8.13E-63 \| \| ENSG00000153814 \| JAZF1 \| -1.58 \| 6.15E-64 \| 8.17E-63 \| \| ENSG00000039319 \| ZFYVE16 \| 1.17 \| 6.21E-64 \| 8.25E-63 \| \| ENSG00000175471 \| MCTP1 \| 1.55 \| 7.25E-64 \| 9.61E-63 \| \| ENSG00000117335 \| CD46 \| -0.77 \| 8.71E-64 \| 1.15E-62 \| \| ENSG00000253368 \| TRNP1 \| -3.12 \| 9.26E-64 \| 1.23E-62 \| \| ENSG00000093072 \| ADA2 \| -2.32 \| 9.44E-64 \| 1.25E-62 \| \| ENSG00000180957 \| PITPNB \| 0.81 \| 1.00E-63 \| 1.32E-62 \| \| ENSG00000164164 \| OTUD4 \| 0.72 \| 1.16E-63 \| 1.54E-62 \| \| ENSG00000104043 \| ATP8B4 \| 0.86 \| 1.21E-63 \| 1.60E-62 \| \| ENSG00000069399 \| BCL3 \| 1.08 \| 1.33E-63 \| 1.75E-62 \| \| ENSG00000149269 \| PAK1 \| -1.39 \| 1.35E-63 \| 1.78E-62 \| \| ENSG00000080546 \| SESN1 \| -0.98 \| 1.35E-63 \| 1.78E-62 \| \| ENSG00000118513 \| MYB \| -2.76 \| 1.45E-63 \| 1.91E-62 \| \| ENSG00000116717 \| GADD45A \| -1.37 \| 1.58E-63 \| 2.08E-62 \| \| ENSG00000137094 \| DNAJB5 \| 1.10 \| 1.70E-63 \| 2.22E-62 \| \| ENSG00000169398 \| PTK2 \| 0.77 \| 2.47E-63 \| 3.24E-62 \| \| ENSG00000105376 \| ICAM5 \| 1.27 \| 3.90E-63 \| 5.10E-62 \| \| ENSG00000138182 \| KIF20B \| -2.33 \| 5.07E-63 \| 6.63E-62 \| \| ENSG00000156642 \| NPTN \| 0.60 \| 7.43E-63 \| 9.70E-62 \| \| ENSG00000064652 \| SNX24 \| -2.94 \| 8.28E-63 \| 1.08E-61 \| \| ENSG00000251322 \| SHANK3 \| -1.16 \| 8.32E-63 \| 1.08E-61 \| \| ENSG00000026652 \| AGPAT4 \| 1.03 \| 9.17E-63 \| 1.19E-61 \| \| ENSG00000064687 \| ABCA7 \| -1.95 \| 1.45E-62 \| 1.88E-61 \| \| ENSG00000143228 \| NUF2 \| -2.98 \| 1.46E-62 \| 1.89E-61 \| \| ENSG00000119772 \| DNMT3A \| -1.46 \| 1.87E-62 \| 2.42E-61 \| \| ENSG00000178104 \| PDE4DIP \| 1.73 \| 2.16E-62 \| 2.80E-61 \| \| ENSG00000178104 \| LOC653513 \| 1.73 \| 2.16E-62 \| 2.80E-61 \| \| ENSG00000168495 \| POLR3D \| 0.87 \| 2.81E-62 \| 3.65E-61 \| \| ENSG00000106070 \| GRB10 \| 1.78 \| 2.93E-62 \| 3.79E-61 \| \| ENSG00000172380 \| GNG12 \| 1.14 \| 3.32E-62 \| 4.29E-61 \| \| ENSG00000123643 \| SLC36A1 \| 0.66 \| 3.35E-62 \| 4.33E-61 \| \| ENSG00000134057 \| CCNB1 \| -2.36 \| 3.56E-62 \| 4.60E-61 \| \| ENSG00000043143 \| JADE2 \| -2.93 \| 3.95E-62 \| 5.10E-61 \| \| ENSG00000130940 \| CASZ1 \| -2.24 \| 4.14E-62 \| 5.33E-61 \| \| ENSG00000175463 \| TBC1D10C \| -3.79 \| 4.24E-62 \| 5.46E-61 \| \| ENSG00000171951 \| SCG2 \| 1.83 \| 4.62E-62 \| 5.94E-61 \| \| ENSG00000244405 \| ETV5 \| -0.97 \| 4.77E-62 \| 6.13E-61 \| \| ENSG00000138685 \| FGF2 \| 1.69 \| 4.90E-62 \| 6.29E-61 \| \| ENSG00000176903 \| PNMA1 \| 0.84 \| 5.58E-62 \| 7.16E-61 \| \| ENSG00000107929 \| LARP4B \| -0.78 \| 6.05E-62 \| 7.76E-61 \| \| ENSG00000117407 \| ARTN \| 3.00 \| 6.51E-62 \| 8.33E-61 \| \| ENSG00000057252 \| SOAT1 \| -1.31 \| 6.59E-62 \| 8.42E-61 \| \| ENSG00000173660 \| UQCRH \| -0.66 \| 7.53E-62 \| 9.62E-61 \| \| ENSG00000081985 \| IL12RB2 \| -1.45 \| 8.13E-62 \| 1.04E-60 \| \| ENSG00000204642 \| HLA-F \| -4.61 \| 8.72E-62 \| 1.11E-60 \| \| ENSG00000090097 \| PCBP4 \| -1.11 \| 9.51E-62 \| 1.21E-60 \| \| ENSG00000115507 \| OTX1 \| -1.09 \| 9.69E-62 \| 1.23E-60 \| \| ENSG00000143333 \| RGS16 \| 1.57 \| 9.87E-62 \| 1.25E-60 \| \| ENSG00000164296 \| TIGD6 \| 1.23 \| 1.14E-61 \| 1.45E-60 \| \| ENSG00000102096 \| PIM2 \| 1.35 \| 1.15E-61 \| 1.46E-60 \| \| ENSG00000112852 \| PCDHB2 \| -1.46 \| 1.31E-61 \| 1.66E-60 \| \| ENSG00000112701 \| SENP6 \| -1.12 \| 1.42E-61 \| 1.79E-60 \| \| ENSG00000100284 \| TOM1 \| 0.83 \| 1.42E-61 \| 1.80E-60 \| \| ENSG00000144857 \| BOC \| 2.32 \| 1.46E-61 \| 1.84E-60 \| \| ENSG00000050628 \| PTGER3 \| 3.72 \| 1.54E-61 \| 1.94E-60 \| \| ENSG00000142949 \| PTPRF \| -2.12 \| 1.62E-61 \| 2.05E-60 \| \| ENSG00000006283 \| CACNA1G \| 2.12 \| 2.01E-61 \| 2.53E-60 \| \| ENSG00000063438 \| AHRR \| 0.62 \| 2.72E-61 \| 3.43E-60 \| \| ENSG00000151883 \| PARP8 \| -1.11 \| 3.07E-61 \| 3.86E-60 \| \| ENSG00000038427 \| VCAN \| -1.95 \| 4.25E-61 \| 5.34E-60 \| \| ENSG00000111335 \| OAS2 \| -2.56 \| 4.59E-61 \| 5.77E-60 \| \| ENSG00000060718 \| COL11A1 \| -4.78 \| 4.78E-61 \| 6.00E-60 \| \| ENSG00000108405 \| P2RX1 \| 1.53 \| 4.87E-61 \| 6.11E-60 \| \| ENSG00000102316 \| MAGED2 \| 0.82 \| 6.35E-61 \| 7.96E-60 \| \| ENSG00000161800 \| RACGAP1 \| -2.26 \| 6.67E-61 \| 8.35E-60 \| \| ENSG00000165480 \| SKA3 \| -2.40 \| 8.23E-61 \| 1.03E-59 \| \| ENSG00000101665 \| SMAD7 \| 0.80 \| 8.65E-61 \| 1.08E-59 \| \| ENSG00000112419 \| PHACTR2 \| 1.08 \| 1.01E-60 \| 1.26E-59 \| \| ENSG00000170606 \| HSPA4 \| -0.70 \| 1.19E-60 \| 1.48E-59 \| \| ENSG00000131018 \| SYNE1 \| 0.94 \| 1.29E-60 \| 1.61E-59 \| \| ENSG00000205730 \| ITPRIPL2 \| 1.01 \| 1.47E-60 \| 1.83E-59 \| \| ENSG00000204839 \| MROH6 \| -1.68 \| 1.56E-60 \| 1.94E-59 \| \| ENSG00000165244 \| ZNF367 \| -1.81 \| 1.63E-60 \| 2.03E-59 \| \| ENSG00000197471 \| SPN \| -0.85 \| 1.67E-60 \| 2.06E-59 \| \| ENSG00000140497 \| SCAMP2 \| -0.61 \| 1.69E-60 \| 2.10E-59 \| \| ENSG00000166927 \| MS4A7 \| -1.39 \| 1.92E-60 \| 2.38E-59 \| \| ENSG00000009790 \| TRAF3IP3 \| -1.26 \| 2.03E-60 \| 2.51E-59 \| \| ENSG00000096060 \| FKBP5 \| -1.82 \| 2.47E-60 \| 3.05E-59 \| \| ENSG00000205593 \| DENND6B \| -1.25 \| 2.63E-60 \| 3.25E-59 \| \| ENSG00000262406 \| MMP12 \| -2.45 \| 2.70E-60 \| 3.33E-59 \| \| ENSG00000164292 \| RHOBTB3 \| 1.20 \| 2.98E-60 \| 3.68E-59 \| \| ENSG00000197217 \| ENTPD4 \| 0.85 \| 3.09E-60 \| 3.81E-59 \| \| ENSG00000119977 \| TCTN3 \| -1.18 \| 3.17E-60 \| 3.90E-59 \| \| ENSG00000091073 \| DTX2 \| 1.15 \| 3.97E-60 \| 4.89E-59 \| \| ENSG00000236453 \|  \| 2.59 \| 4.55E-60 \| 5.59E-59 \| \| ENSG00000150337 \| FCGR1A \| -1.85 \| 4.67E-60 \| 5.74E-59 \| \| ENSG00000065328 \| MCM10 \| -2.59 \| 5.57E-60 \| 6.83E-59 \| \| ENSG00000152804 \| HHEX \| -1.91 \| 5.77E-60 \| 7.06E-59 \| \| ENSG00000241839 \| PLEKHO2 \| 0.67 \| 7.09E-60 \| 8.67E-59 \| \| ENSG00000151693 \| ASAP2 \| -0.96 \| 9.27E-60 \| 1.13E-58 \| \| ENSG00000167601 \| AXL \| -3.62 \| 1.01E-59 \| 1.23E-58 \| \| ENSG00000135269 \| TES \| -0.95 \| 1.05E-59 \| 1.27E-58 \| \| ENSG00000143382 \| ADAMTSL4 \| -2.06 \| 1.10E-59 \| 1.34E-58 \| \| ENSG00000118922 \| KLF12 \| 1.76 \| 1.48E-59 \| 1.80E-58 \| \| ENSG00000176393 \| RNPEP \| -0.71 \| 1.65E-59 \| 2.00E-58 \| \| ENSG00000112984 \| KIF20A \| -3.76 \| 1.81E-59 \| 2.20E-58 \| \| ENSG00000100330 \| MTMR3 \| 0.83 \| 2.35E-59 \| 2.85E-58 \| \| ENSG00000053918 \| KCNQ1 \| -1.04 \| 3.77E-59 \| 4.57E-58 \| \| ENSG00000143819 \| EPHX1 \| -1.43 \| 4.15E-59 \| 5.03E-58 \| \| ENSG00000108828 \| VAT1 \| -0.70 \| 5.98E-59 \| 7.23E-58 \| \| ENSG00000168283 \| BMI1 \| -2.24 \| 6.28E-59 \| 7.60E-58 \| \| ENSG00000106003 \| LFNG \| 1.00 \| 6.68E-59 \| 8.07E-58 \| \| ENSG00000131459 \| GFPT2 \| 3.31 \| 9.62E-59 \| 1.16E-57 \| \| ENSG00000160326 \| SLC2A6 \| 0.87 \| 1.18E-58 \| 1.42E-57 \| \| ENSG00000177084 \| POLE \| -1.00 \| 1.19E-58 \| 1.43E-57 \| \| ENSG00000105486 \| LIG1 \| -1.25 \| 1.32E-58 \| 1.59E-57 \| \| ENSG00000109452 \| INPP4B \| -1.32 \| 2.63E-58 \| 3.16E-57 \| \| ENSG00000185800 \| DMWD \| 0.87 \| 3.58E-58 \| 4.31E-57 \| \| ENSG00000101336 \| HCK \| 1.09 \| 3.59E-58 \| 4.31E-57 \| \| ENSG00000154229 \| PRKCA \| 0.65 \| 3.61E-58 \| 4.33E-57 \| \| ENSG00000162337 \| LRP5 \| -1.64 \| 3.65E-58 \| 4.37E-57 \| \| ENSG00000147168 \| IL2RG \| -6.35 \| 4.43E-58 \| 5.31E-57 \| \| ENSG00000133246 \| PRAM1 \| -1.67 \| 4.84E-58 \| 5.79E-57 \| \| ENSG00000125378 \| BMP4 \| -2.68 \| 5.02E-58 \| 6.00E-57 \| \| ENSG00000124496 \| TRERF1 \| 0.65 \| 6.99E-58 \| 8.35E-57 \| \| ENSG00000158717 \| RNF166 \| -0.72 \| 7.83E-58 \| 9.34E-57 \| \| ENSG00000182541 \| LIMK2 \| -1.48 \| 1.01E-57 \| 1.20E-56 \| \| ENSG00000078269 \| SYNJ2 \| 0.78 \| 1.09E-57 \| 1.29E-56 \| \| ENSG00000078900 \| TP73 \| -1.87 \| 1.14E-57 \| 1.35E-56 \| \| ENSG00000162302 \| RPS6KA4 \| -1.15 \| 1.33E-57 \| 1.59E-56 \| \| ENSG00000203799 \| CCDC162P \| -2.69 \| 1.42E-57 \| 1.69E-56 \| \| ENSG00000145907 \| G3BP1 \| 0.59 \| 1.69E-57 \| 2.01E-56 \| \| ENSG00000144554 \| FANCD2 \| -1.55 \| 1.85E-57 \| 2.20E-56 \| \| ENSG00000154654 \| NCAM2 \| -3.70 \| 1.94E-57 \| 2.30E-56 \| \| ENSG00000118640 \| VAMP8 \| -0.71 \| 1.98E-57 \| 2.35E-56 \| \| ENSG00000153443 \| UBALD1 \| 0.85 \| 2.19E-57 \| 2.59E-56 \| \| ENSG00000160949 \| TONSL \| -1.18 \| 2.29E-57 \| 2.71E-56 \| \| ENSG00000132334 \| PTPRE \| 0.62 \| 2.39E-57 \| 2.82E-56 \| \| ENSG00000198837 \| DENND4B \| -0.72 \| 2.46E-57 \| 2.90E-56 \| \| ENSG00000265972 \| TXNIP \| -0.69 \| 2.52E-57 \| 2.97E-56 \| \| ENSG00000196083 \| IL1RAP \| 1.44 \| 2.64E-57 \| 3.12E-56 \| \| ENSG00000184661 \| CDCA2 \| -2.79 \| 2.79E-57 \| 3.28E-56 \| \| ENSG00000105829 \| BET1 \| 1.36 \| 2.97E-57 \| 3.49E-56 \| \| ENSG00000087074 \| PPP1R15A \| 1.48 \| 3.00E-57 \| 3.53E-56 \| \| ENSG00000109790 \| KLHL5 \| 0.76 \| 3.42E-57 \| 4.02E-56 \| \| ENSG00000113580 \| NR3C1 \| 0.71 \| 4.16E-57 \| 4.89E-56 \| \| ENSG00000117054 \| ACADM \| -0.93 \| 4.28E-57 \| 5.02E-56 \| \| ENSG00000162896 \| PIGR \| -3.44 \| 4.69E-57 \| 5.49E-56 \| \| ENSG00000117593 \| DARS2 \| -1.28 \| 4.78E-57 \| 5.60E-56 \| \| ENSG00000163171 \| CDC42EP3 \| 0.65 \| 5.86E-57 \| 6.85E-56 \| \| ENSG00000175305 \| CCNE2 \| -1.66 \| 6.91E-57 \| 8.07E-56 \| \| ENSG00000145147 \| SLIT2 \| 2.40 \| 7.32E-57 \| 8.55E-56 \| \| ENSG00000137807 \| KIF23 \| -1.77 \| 8.79E-57 \| 1.02E-55 \| \| ENSG00000078403 \| MLLT10 \| -1.14 \| 9.84E-57 \| 1.15E-55 \| \| ENSG00000085840 \| ORC1 \| -1.83 \| 1.02E-56 \| 1.19E-55 \| \| ENSG00000158864 \| NDUFS2 \| -0.64 \| 1.04E-56 \| 1.21E-55 \| \| ENSG00000198728 \| LDB1 \| -0.81 \| 1.05E-56 \| 1.22E-55 \| \| ENSG00000167110 \| GOLGA2 \| 0.83 \| 1.09E-56 \| 1.27E-55 \| \| ENSG00000090238 \| YPEL3 \| 0.99 \| 1.24E-56 \| 1.44E-55 \| \| ENSG00000197253 \| TPSB2 \| -3.79 \| 1.38E-56 \| 1.60E-55 \| \| ENSG00000129173 \| E2F8 \| -2.41 \| 1.46E-56 \| 1.69E-55 \| \| ENSG00000121933 \| TMIGD3 \| -2.02 \| 1.49E-56 \| 1.72E-55 \| \| ENSG00000086300 \| SNX10 \| -1.77 \| 1.61E-56 \| 1.87E-55 \| \| ENSG00000173812 \| EIF1 \| 0.59 \| 1.64E-56 \| 1.89E-55 \| \| ENSG00000078401 \| EDN1 \| 2.20 \| 1.67E-56 \| 1.93E-55 \| \| ENSG00000119535 \| CSF3R \| -3.13 \| 1.97E-56 \| 2.27E-55 \| \| ENSG00000102760 \| RGCC \| -0.66 \| 2.11E-56 \| 2.43E-55 \| \| ENSG00000021762 \| OSBPL5 \| -1.28 \| 2.28E-56 \| 2.63E-55 \| \| ENSG00000134690 \| CDCA8 \| -2.02 \| 2.42E-56 \| 2.78E-55 \| \| ENSG00000112941 \| TENT4A \| 0.90 \| 2.67E-56 \| 3.07E-55 \| \| ENSG00000092470 \| WDR76 \| -1.98 \| 3.19E-56 \| 3.67E-55 \| \| ENSG00000107099 \| DOCK8 \| -0.60 \| 3.23E-56 \| 3.71E-55 \| \| ENSG00000197561 \| ELANE \| -6.71 \| 3.75E-56 \| 4.30E-55 \| \| ENSG00000109171 \| SLAIN2 \| 0.71 \| 4.43E-56 \| 5.07E-55 \| \| ENSG00000126787 \| DLGAP5 \| -3.29 \| 5.54E-56 \| 6.34E-55 \| \| ENSG00000089057 \| SLC23A2 \| -0.70 \| 6.70E-56 \| 7.66E-55 \| \| ENSG00000142227 \| EMP3 \| 0.69 \| 7.30E-56 \| 8.33E-55 \| \| ENSG00000165476 \| REEP3 \| -0.92 \| 7.53E-56 \| 8.59E-55 \| \| ENSG00000090013 \| BLVRB \| -1.35 \| 9.23E-56 \| 1.05E-54 \| \| ENSG00000160991 \| ORAI2 \| 0.66 \| 9.75E-56 \| 1.11E-54 \| \| ENSG00000115525 \| ST3GAL5 \| -1.04 \| 1.07E-55 \| 1.21E-54 \| \| ENSG00000277632 \| CCL3 \| 2.45 \| 1.15E-55 \| 1.31E-54 \| \| ENSG00000093009 \| CDC45 \| -1.71 \| 1.16E-55 \| 1.32E-54 \| \| ENSG00000153012 \| LGI2 \| -0.91 \| 1.24E-55 \| 1.41E-54 \| \| ENSG00000148803 \| FUOM \| -1.67 \| 1.27E-55 \| 1.44E-54 \| \| ENSG00000086065 \| CHMP5 \| 0.80 \| 1.28E-55 \| 1.45E-54 \| \| ENSG00000175224 \| ATG13 \| 0.67 \| 1.40E-55 \| 1.58E-54 \| \| ENSG00000166278 \| C2 \| -4.82 \| 1.42E-55 \| 1.61E-54 \| \| ENSG00000186716 \| BCR \| 0.62 \| 1.45E-55 \| 1.63E-54 \| \| ENSG00000117691 \| NENF \| -1.07 \| 1.52E-55 \| 1.72E-54 \| \| ENSG00000119326 \| CTNNAL1 \| 1.59 \| 1.73E-55 \| 1.95E-54 \| \| ENSG00000197702 \| PARVA \| 0.77 \| 1.88E-55 \| 2.12E-54 \| \| ENSG00000137877 \| SPTBN5 \| -1.90 \| 2.00E-55 \| 2.25E-54 \| \| ENSG00000133561 \| GIMAP6 \| -1.24 \| 2.01E-55 \| 2.26E-54 \| \| ENSG00000088899 \| LZTS3 \| 2.08 \| 2.02E-55 \| 2.28E-54 \| \| ENSG00000104447 \| TRPS1 \| 0.91 \| 2.17E-55 \| 2.44E-54 \| \| ENSG00000154429 \| CCSAP \| -1.49 \| 2.42E-55 \| 2.72E-54 \| \| ENSG00000100219 \| XBP1 \| 0.87 \| 2.68E-55 \| 3.01E-54 \| \| ENSG00000237441 \| RGL2 \| -0.82 \| 3.28E-55 \| 3.68E-54 \| \| ENSG00000147481 \| SNTG1 \| 3.86 \| 4.08E-55 \| 4.57E-54 \| \| ENSG00000117643 \| MAN1C1 \| -1.10 \| 5.05E-55 \| 5.65E-54 \| \| ENSG00000080986 \| NDC80 \| -2.55 \| 6.81E-55 \| 7.62E-54 \| \| ENSG00000166595 \| CIAO2B \| 0.89 \| 8.48E-55 \| 9.47E-54 \| \| ENSG00000142731 \| PLK4 \| -1.93 \| 1.02E-54 \| 1.14E-53 \| \| ENSG00000166250 \| CLMP \| -0.79 \| 1.04E-54 \| 1.16E-53 \| \| ENSG00000057704 \| TMCC3 \| 2.08 \| 1.06E-54 \| 1.18E-53 \| \| ENSG00000136732 \| GYPC \| -0.63 \| 1.09E-54 \| 1.22E-53 \| \| ENSG00000049449 \| RCN1 \| 0.69 \| 1.12E-54 \| 1.24E-53 \| \| ENSG00000113522 \| RAD50 \| -1.01 \| 1.27E-54 \| 1.41E-53 \| \| ENSG00000090924 \| PLEKHG2 \| -0.63 \| 1.60E-54 \| 1.77E-53 \| \| ENSG00000005882 \| PDK2 \| -1.08 \| 2.42E-54 \| 2.68E-53 \| \| ENSG00000185947 \| ZNF267 \| 0.84 \| 2.48E-54 \| 2.75E-53 \| \| ENSG00000100154 \| TTC28 \| 1.41 \| 2.58E-54 \| 2.85E-53 \| \| ENSG00000010017 \| RANBP9 \| 0.80 \| 2.76E-54 \| 3.05E-53 \| \| ENSG00000084754 \| HADHA \| -0.61 \| 3.18E-54 \| 3.52E-53 \| \| ENSG00000095321 \| CRAT \| 0.74 \| 4.26E-54 \| 4.71E-53 \| \| ENSG00000113209 \| PCDHB5 \| -5.63 \| 4.32E-54 \| 4.76E-53 \| \| ENSG00000141441 \| GAREM1 \| -2.35 \| 4.53E-54 \| 4.99E-53 \| \| ENSG00000017260 \| ATP2C1 \| 0.80 \| 4.69E-54 \| 5.16E-53 \| \| ENSG00000168393 \| DTYMK \| -1.77 \| 6.99E-54 \| 7.69E-53 \| \| ENSG00000095209 \| TMEM38B \| 1.47 \| 7.23E-54 \| 7.94E-53 \| \| ENSG00000185262 \| UBALD2 \| 0.62 \| 7.96E-54 \| 8.74E-53 \| \| ENSG00000245848 \| CEBPA \| -0.95 \| 8.73E-54 \| 9.58E-53 \| \| ENSG00000142798 \| HSPG2 \| -1.55 \| 8.80E-54 \| 9.65E-53 \| \| ENSG00000175063 \| UBE2C \| -2.36 \| 9.15E-54 \| 1.00E-52 \| \| ENSG00000267034 \|  \| 2.12 \| 9.66E-54 \| 1.06E-52 \| \| ENSG00000140931 \| CMTM3 \| -0.64 \| 1.11E-53 \| 1.21E-52 \| \| ENSG00000233452 \| STXBP5-AS1 \| 1.16 \| 1.25E-53 \| 1.36E-52 \| \| ENSG00000272068 \|  \| -3.11 \| 1.32E-53 \| 1.45E-52 \| \| ENSG00000104856 \| RELB \| 0.80 \| 1.33E-53 \| 1.45E-52 \| \| ENSG00000137872 \| SEMA6D \| 2.22 \| 1.48E-53 \| 1.61E-52 \| \| ENSG00000183020 \| AP2A2 \| -0.61 \| 1.69E-53 \| 1.84E-52 \| \| ENSG00000120686 \| UFM1 \| 0.90 \| 1.73E-53 \| 1.89E-52 \| \| ENSG00000101210 \| EEF1A2 \| -1.80 \| 1.85E-53 \| 2.01E-52 \| \| ENSG00000198133 \| TMEM229B \| -2.07 \| 1.99E-53 \| 2.17E-52 \| \| ENSG00000119042 \| SATB2 \| 0.76 \| 2.05E-53 \| 2.23E-52 \| \| ENSG00000106415 \| GLCCI1 \| -1.43 \| 2.13E-53 \| 2.31E-52 \| \| ENSG00000123240 \| OPTN \| -1.17 \| 2.23E-53 \| 2.42E-52 \| \| ENSG00000176845 \| METRNL \| 0.67 \| 2.30E-53 \| 2.49E-52 \| \| ENSG00000235750 \| KIAA0040 \| -2.32 \| 2.58E-53 \| 2.79E-52 \| \| ENSG00000156675 \| RAB11FIP1 \| -0.75 \| 2.84E-53 \| 3.08E-52 \| \| ENSG00000162413 \| KLHL21 \| 0.96 \| 2.86E-53 \| 3.09E-52 \| \| ENSG00000149968 \| MMP3 \| 1.87 \| 3.06E-53 \| 3.31E-52 \| \| ENSG00000140612 \| SEC11A \| -0.82 \| 3.16E-53 \| 3.41E-52 \| \| ENSG00000156110 \| ADK \| -1.22 \| 3.48E-53 \| 3.75E-52 \| \| ENSG00000178802 \| MPI \| -1.17 \| 3.98E-53 \| 4.30E-52 \| \| ENSG00000174371 \| EXO1 \| -1.71 \| 5.81E-53 \| 6.25E-52 \| \| ENSG00000265808 \| SEC22B \| 0.72 \| 6.20E-53 \| 6.67E-52 \| \| ENSG00000265808 \| SEC22B3 \| 0.72 \| 6.20E-53 \| 6.67E-52 \| \| ENSG00000265808 \| SEC22B2 \| 0.72 \| 6.20E-53 \| 6.67E-52 \| \| ENSG00000171700 \| RGS19 \| -0.79 \| 6.86E-53 \| 7.38E-52 \| \| ENSG00000143110 \| C1orf162 \| -2.60 \| 7.63E-53 \| 8.19E-52 \| \| ENSG00000006459 \| KDM7A \| 1.39 \| 8.31E-53 \| 8.92E-52 \| \| ENSG00000119729 \| RHOQ \| 0.80 \| 9.83E-53 \| 1.05E-51 \| \| ENSG00000007968 \| E2F2 \| -2.64 \| 9.95E-53 \| 1.07E-51 \| \| ENSG00000112992 \| NNT \| -0.94 \| 1.28E-52 \| 1.37E-51 \| \| ENSG00000065802 \| ASB1 \| 0.74 \| 1.28E-52 \| 1.37E-51 \| \| ENSG00000064225 \| ST3GAL6 \| -1.73 \| 1.34E-52 \| 1.43E-51 \| \| ENSG00000253159 \| PCDHGA12 \| -4.89 \| 1.79E-52 \| 1.91E-51 \| \| ENSG00000110422 \| HIPK3 \| 0.65 \| 1.84E-52 \| 1.97E-51 \| \| ENSG00000174744 \| BRMS1 \| 0.74 \| 1.97E-52 \| 2.10E-51 \| \| ENSG00000155363 \| MOV10 \| -0.75 \| 2.05E-52 \| 2.18E-51 \| \| ENSG00000116044 \| NFE2L2 \| 1.38 \| 2.13E-52 \| 2.26E-51 \| \| ENSG00000130396 \| AFDN \| 0.86 \| 2.22E-52 \| 2.37E-51 \| \| ENSG00000141858 \| SAMD1 \| -0.97 \| 2.62E-52 \| 2.79E-51 \| \| ENSG00000133985 \| TTC9 \| 2.77 \| 2.74E-52 \| 2.91E-51 \| \| ENSG00000101460 \| MAP1LC3A \| 1.17 \| 2.93E-52 \| 3.12E-51 \| \| ENSG00000087510 \| TFAP2C \| 1.67 \| 3.32E-52 \| 3.52E-51 \| \| ENSG00000036054 \| TBC1D23 \| 0.91 \| 3.40E-52 \| 3.60E-51 \| \| ENSG00000053254 \| FOXN3 \| -0.90 \| 3.41E-52 \| 3.62E-51 \| \| ENSG00000175354 \| PTPN2 \| 0.90 \| 3.70E-52 \| 3.92E-51 \| \| ENSG00000134184 \| GSTM1 \| -1.81 \| 4.02E-52 \| 4.26E-51 \| \| ENSG00000078399 \| HOXA9 \| -1.41 \| 4.18E-52 \| 4.42E-51 \| \| ENSG00000172348 \| RCAN2 \| 2.78 \| 4.35E-52 \| 4.60E-51 \| \| ENSG00000157224 \| CLDN12 \| 1.29 \| 4.43E-52 \| 4.68E-51 \| \| ENSG00000157224 \| LOC102723899 \| 1.29 \| 4.43E-52 \| 4.68E-51 \| \| ENSG00000183955 \| KMT5A \| -0.83 \| 4.48E-52 \| 4.73E-51 \| \| ENSG00000125827 \| TMX4 \| 0.91 \| 5.05E-52 \| 5.32E-51 \| \| ENSG00000008394 \| MGST1 \| -1.42 \| 5.52E-52 \| 5.82E-51 \| \| ENSG00000162490 \| DRAXIN \| 2.50 \| 5.63E-52 \| 5.93E-51 \| \| ENSG00000181381 \| DDX60L \| -0.83 \| 6.56E-52 \| 6.91E-51 \| \| ENSG00000187634 \| SAMD11 \| -4.17 \| 6.78E-52 \| 7.13E-51 \| \| ENSG00000160877 \| NACC1 \| 0.63 \| 6.97E-52 \| 7.32E-51 \| \| ENSG00000185811 \| IKZF1 \| -0.92 \| 7.03E-52 \| 7.38E-51 \| \| ENSG00000080493 \| SLC4A4 \| 2.03 \| 7.23E-52 \| 7.59E-51 \| \| ENSG00000186111 \| PIP5K1C \| 0.59 \| 9.38E-52 \| 9.82E-51 \| \| ENSG00000116157 \| GPX7 \| -2.29 \| 9.70E-52 \| 1.02E-50 \| \| ENSG00000197208 \| SLC22A4 \| 2.27 \| 9.89E-52 \| 1.03E-50 \| \| ENSG00000197312 \| DDI2 \| 0.91 \| 1.19E-51 \| 1.24E-50 \| \| ENSG00000076356 \| PLXNA2 \| 1.89 \| 1.45E-51 \| 1.52E-50 \| \| ENSG00000160883 \| HK3 \| -2.99 \| 1.51E-51 \| 1.57E-50 \| \| ENSG00000131788 \| PIAS3 \| 0.76 \| 1.57E-51 \| 1.63E-50 \| \| ENSG00000135862 \| LAMC1 \| 0.80 \| 1.66E-51 \| 1.73E-50 \| \| ENSG00000076382 \| SPAG5 \| -1.43 \| 1.68E-51 \| 1.75E-50 \| \| ENSG00000116161 \| CACYBP \| -0.82 \| 1.89E-51 \| 1.97E-50 \| \| ENSG00000179295 \| PTPN11 \| 0.60 \| 2.03E-51 \| 2.11E-50 \| \| ENSG00000148841 \| ITPRIP \| 0.61 \| 2.04E-51 \| 2.12E-50 \| \| ENSG00000187676 \| B3GLCT \| 1.23 \| 2.23E-51 \| 2.32E-50 \| \| ENSG00000027075 \| PRKCH \| -2.94 \| 2.32E-51 \| 2.41E-50 \| \| ENSG00000102158 \| MAGT1 \| 0.74 \| 2.33E-51 \| 2.41E-50 \| \| ENSG00000187325 \| TAF9B \| 1.12 \| 2.42E-51 \| 2.50E-50 \| \| ENSG00000167772 \| ANGPTL4 \| 2.83 \| 2.47E-51 \| 2.55E-50 \| \| ENSG00000176697 \| BDNF \| 2.16 \| 2.68E-51 \| 2.77E-50 \| \| ENSG00000127586 \| CHTF18 \| -1.35 \| 2.74E-51 \| 2.83E-50 \| \| ENSG00000130363 \| RSPH3 \| 0.89 \| 2.80E-51 \| 2.88E-50 \| \| ENSG00000278948 \|  \| 1.49 \| 2.85E-51 \| 2.94E-50 \| \| ENSG00000227039 \| ITGB2-AS1 \| -0.96 \| 2.86E-51 \| 2.95E-50 \| \| ENSG00000235884 \| LINC00941 \| 1.24 \| 2.90E-51 \| 2.98E-50 \| \| ENSG00000105856 \| HBP1 \| 0.73 \| 3.11E-51 \| 3.20E-50 \| \| ENSG00000168496 \| FEN1 \| -1.07 \| 3.58E-51 \| 3.68E-50 \| \| ENSG00000162714 \| ZNF496 \| -0.97 \| 3.60E-51 \| 3.70E-50 \| \| ENSG00000173530 \| TNFRSF10D \| 0.86 \| 3.63E-51 \| 3.73E-50 \| \| ENSG00000166848 \| TERF2IP \| 0.60 \| 3.70E-51 \| 3.79E-50 \| \| ENSG00000166848 \| LOC105371348 \| 0.60 \| 3.70E-51 \| 3.79E-50 \| \| ENSG00000100731 \| PCNX1 \| 0.85 \| 3.77E-51 \| 3.87E-50 \| \| ENSG00000185669 \| SNAI3 \| -1.38 \| 3.90E-51 \| 4.00E-50 \| \| ENSG00000181827 \| RFX7 \| 0.79 \| 3.94E-51 \| 4.04E-50 \| \| ENSG00000130881 \| LRP3 \| -1.08 \| 4.83E-51 \| 4.94E-50 \| \| ENSG00000188690 \| UROS \| -1.15 \| 5.23E-51 \| 5.35E-50 \| \| ENSG00000184545 \| DUSP8 \| 1.42 \| 5.55E-51 \| 5.67E-50 \| \| ENSG00000186174 \| BCL9L \| 0.63 \| 7.38E-51 \| 7.53E-50 \| \| ENSG00000130830 \| MPP1 \| -1.17 \| 7.94E-51 \| 8.10E-50 \| \| ENSG00000136861 \| CDK5RAP2 \| 0.78 \| 9.34E-51 \| 9.52E-50 \| \| ENSG00000126524 \| SBDS \| 0.68 \| 9.76E-51 \| 9.95E-50 \| \| ENSG00000198900 \| TOP1 \| 0.62 \| 1.02E-50 \| 1.04E-49 \| \| ENSG00000257932 \|  \| 2.13 \| 1.04E-50 \| 1.06E-49 \| \| ENSG00000196739 \| COL27A1 \| -0.97 \| 1.21E-50 \| 1.23E-49 \| \| ENSG00000165030 \| NFIL3 \| 0.74 \| 1.22E-50 \| 1.24E-49 \| \| ENSG00000101966 \| XIAP \| 0.78 \| 1.23E-50 \| 1.25E-49 \| \| ENSG00000070214 \| SLC44A1 \| 0.77 \| 1.24E-50 \| 1.26E-49 \| \| ENSG00000158850 \| B4GALT3 \| -0.74 \| 1.33E-50 \| 1.34E-49 \| \| ENSG00000146112 \| PPP1R18 \| 0.68 \| 1.44E-50 \| 1.46E-49 \| \| ENSG00000146112 \| LOC107987457 \| 0.68 \| 1.44E-50 \| 1.46E-49 \| \| ENSG00000144893 \| MED12L \| 2.74 \| 1.72E-50 \| 1.74E-49 \| \| ENSG00000099860 \| GADD45B \| 1.09 \| 2.03E-50 \| 2.05E-49 \| \| ENSG00000198246 \| SLC29A3 \| -1.54 \| 2.28E-50 \| 2.30E-49 \| \| ENSG00000165795 \| NDRG2 \| -1.14 \| 2.53E-50 \| 2.55E-49 \| \| ENSG00000179862 \| CITED4 \| 0.68 \| 2.53E-50 \| 2.55E-49 \| \| ENSG00000178922 \| HYI \| -1.13 \| 2.74E-50 \| 2.77E-49 \| \| ENSG00000070814 \| TCOF1 \| 0.69 \| 2.94E-50 \| 2.97E-49 \| \| ENSG00000147454 \| SLC25A37 \| 1.24 \| 2.97E-50 \| 2.99E-49 \| \| ENSG00000117245 \| KIF17 \| -0.85 \| 3.58E-50 \| 3.61E-49 \| \| ENSG00000176974 \| SHMT1 \| -2.70 \| 4.15E-50 \| 4.18E-49 \| \| ENSG00000171509 \| RXFP1 \| -3.29 \| 4.57E-50 \| 4.60E-49 \| \| ENSG00000157020 \| SEC13 \| 0.60 \| 4.73E-50 \| 4.75E-49 \| \| ENSG00000165379 \| LRFN5 \| 1.51 \| 4.74E-50 \| 4.76E-49 \| \| ENSG00000153944 \| MSI2 \| 0.87 \| 5.52E-50 \| 5.54E-49 \| \| ENSG00000188064 \| WNT7B \| 1.47 \| 7.23E-50 \| 7.25E-49 \| \| ENSG00000140943 \| MBTPS1 \| 0.62 \| 7.61E-50 \| 7.62E-49 \| \| ENSG00000143653 \| SCCPDH \| -1.18 \| 7.97E-50 \| 7.98E-49 \| \| ENSG00000141574 \| SECTM1 \| -3.89 \| 8.54E-50 \| 8.54E-49 \| \| ENSG00000186340 \| THBS2 \| -2.75 \| 1.04E-49 \| 1.04E-48 \| \| ENSG00000147036 \| LANCL3 \| -1.85 \| 1.13E-49 \| 1.12E-48 \| \| ENSG00000147231 \| RADX \| -1.48 \| 1.17E-49 \| 1.17E-48 \| \| ENSG00000013563 \| DNASE1L1 \| -0.77 \| 1.20E-49 \| 1.20E-48 \| \| ENSG00000260920 \|  \| 0.77 \| 1.25E-49 \| 1.24E-48 \| \| ENSG00000159640 \| ACE \| -2.16 \| 1.48E-49 \| 1.48E-48 \| \| ENSG00000162298 \| SYVN1 \| 0.60 \| 1.51E-49 \| 1.51E-48 \| \| ENSG00000141580 \| WDR45B \| 0.63 \| 1.56E-49 \| 1.55E-48 \| \| ENSG00000068308 \| OTUD5 \| 0.76 \| 1.75E-49 \| 1.74E-48 \| \| ENSG00000177628 \| GBA \| -0.61 \| 2.47E-49 \| 2.45E-48 \| \| ENSG00000160447 \| PKN3 \| -1.88 \| 2.80E-49 \| 2.77E-48 \| \| ENSG00000185009 \| AP3M1 \| -0.83 \| 3.26E-49 \| 3.23E-48 \| \| ENSG00000126709 \| IFI6 \| -1.92 \| 3.40E-49 \| 3.37E-48 \| \| ENSG00000148175 \| STOM \| 0.61 \| 3.63E-49 \| 3.59E-48 \| \| ENSG00000136758 \| YME1L1 \| -0.63 \| 4.41E-49 \| 4.35E-48 \| \| ENSG00000072422 \| RHOBTB1 \| -2.16 \| 4.88E-49 \| 4.81E-48 \| \| ENSG00000115461 \| IGFBP5 \| -2.56 \| 5.32E-49 \| 5.25E-48 \| \| ENSG00000183578 \| TNFAIP8L3 \| -0.76 \| 6.59E-49 \| 6.49E-48 \| \| ENSG00000138835 \| RGS3 \| 0.82 \| 6.95E-49 \| 6.83E-48 \| \| ENSG00000182628 \| SKA2 \| -1.38 \| 7.52E-49 \| 7.39E-48 \| \| ENSG00000135241 \| PNPLA8 \| 1.20 \| 7.74E-49 \| 7.61E-48 \| \| ENSG00000066455 \| GOLGA5 \| 0.73 \| 9.28E-49 \| 9.11E-48 \| \| ENSG00000145545 \| SRD5A1 \| 1.04 \| 9.51E-49 \| 9.34E-48 \| \| ENSG00000257923 \| CUX1 \| 0.62 \| 1.09E-48 \| 1.07E-47 \| \| ENSG00000169057 \| MECP2 \| 0.75 \| 1.18E-48 \| 1.15E-47 \| \| ENSG00000174004 \| NRROS \| -0.66 \| 1.24E-48 \| 1.21E-47 \| \| ENSG00000139496 \| NUP58 \| 0.75 \| 1.42E-48 \| 1.38E-47 \| \| ENSG00000198125 \| MB \| -3.40 \| 1.55E-48 \| 1.51E-47 \| \| ENSG00000176542 \| USF3 \| 0.93 \| 1.56E-48 \| 1.52E-47 \| \| ENSG00000113328 \| CCNG1 \| -0.71 \| 1.63E-48 \| 1.59E-47 \| \| ENSG00000134352 \| IL6ST \| 0.87 \| 1.79E-48 \| 1.74E-47 \| \| ENSG00000006652 \| IFRD1 \| 0.92 \| 1.93E-48 \| 1.88E-47 \| \| ENSG00000126016 \| AMOT \| 1.72 \| 2.65E-48 \| 2.58E-47 \| \| ENSG00000070081 \| NUCB2 \| -1.16 \| 2.90E-48 \| 2.82E-47 \| \| ENSG00000163808 \| KIF15 \| -2.29 \| 3.12E-48 \| 3.03E-47 \| \| ENSG00000095370 \| SH2D3C \| -1.75 \| 3.55E-48 \| 3.45E-47 \| \| ENSG00000170439 \| METTL7B \| -1.46 \| 3.60E-48 \| 3.50E-47 \| \| ENSG00000132356 \| PRKAA1 \| 0.61 \| 4.85E-48 \| 4.70E-47 \| \| ENSG00000134755 \| DSC2 \| -1.83 \| 5.35E-48 \| 5.19E-47 \| \| ENSG00000006747 \| SCIN \| -4.14 \| 5.46E-48 \| 5.29E-47 \| \| ENSG00000156265 \| MAP3K7CL \| 0.78 \| 5.81E-48 \| 5.62E-47 \| \| ENSG00000183826 \| BTBD9 \| 0.96 \| 6.01E-48 \| 5.81E-47 \| \| ENSG00000100122 \| CRYBB1 \| -1.99 \| 6.03E-48 \| 5.82E-47 \| \| ENSG00000163512 \| AZI2 \| 0.97 \| 6.52E-48 \| 6.30E-47 \| \| ENSG00000067840 \| PDZD4 \| 2.49 \| 6.69E-48 \| 6.45E-47 \| \| ENSG00000145358 \| DDIT4L \| -0.68 \| 7.45E-48 \| 7.19E-47 \| \| ENSG00000163050 \| COQ8A \| -0.74 \| 7.56E-48 \| 7.28E-47 \| \| ENSG00000112514 \| CUTA \| -0.80 \| 1.05E-47 \| 1.01E-46 \| \| ENSG00000102908 \| NFAT5 \| 1.00 \| 1.13E-47 \| 1.08E-46 \| \| ENSG00000197959 \| DNM3 \| 1.16 \| 1.21E-47 \| 1.16E-46 \| \| ENSG00000120913 \| PDLIM2 \| -0.91 \| 1.36E-47 \| 1.31E-46 \| \| ENSG00000187741 \| FANCA \| -1.29 \| 1.41E-47 \| 1.36E-46 \| \| ENSG00000186074 \| CD300LF \| -2.80 \| 1.58E-47 \| 1.52E-46 \| \| ENSG00000178980 \| SELENOW \| -0.99 \| 1.63E-47 \| 1.56E-46 \| \| ENSG00000092345 \| DAZL \| 4.09 \| 1.69E-47 \| 1.62E-46 \| \| ENSG00000204248 \| COL11A2 \| -2.25 \| 1.85E-47 \| 1.77E-46 \| \| ENSG00000130770 \| ATP5IF1 \| -0.89 \| 1.88E-47 \| 1.80E-46 \| \| ENSG00000186522 \| 10-Sep \| 1.32 \| 1.90E-47 \| 1.82E-46 \| \| ENSG00000177706 \| FAM20C \| 0.64 \| 1.93E-47 \| 1.85E-46 \| \| ENSG00000175573 \| C11orf68 \| 0.92 \| 2.23E-47 \| 2.13E-46 \| \| ENSG00000075426 \| FOSL2 \| 0.76 \| 2.44E-47 \| 2.33E-46 \| \| ENSG00000166801 \| FAM111A \| -0.94 \| 2.55E-47 \| 2.43E-46 \| \| ENSG00000196449 \| YRDC \| 0.90 \| 2.83E-47 \| 2.70E-46 \| \| ENSG00000105520 \| PLPPR2 \| 0.83 \| 3.03E-47 \| 2.88E-46 \| \| ENSG00000130511 \| SSBP4 \| -0.60 \| 3.96E-47 \| 3.77E-46 \| \| ENSG00000137700 \| SLC37A4 \| -1.51 \| 3.99E-47 \| 3.79E-46 \| \| ENSG00000142892 \| PIGK \| -2.06 \| 4.13E-47 \| 3.92E-46 \| \| ENSG00000185104 \| FAF1 \| -0.92 \| 5.90E-47 \| 5.61E-46 \| \| ENSG00000119801 \| YPEL5 \| 0.79 \| 8.25E-47 \| 7.83E-46 \| \| ENSG00000104972 \| LILRB1 \| -1.61 \| 8.98E-47 \| 8.52E-46 \| \| ENSG00000104972 \| LOC107987461 \| -1.61 \| 8.98E-47 \| 8.52E-46 \| \| ENSG00000029153 \| ARNTL2 \| 0.98 \| 9.18E-47 \| 8.70E-46 \| \| ENSG00000119537 \| KDSR \| 0.79 \| 9.50E-47 \| 9.00E-46 \| \| ENSG00000123728 \| RAP2C \| 0.79 \| 1.02E-46 \| 9.61E-46 \| \| ENSG00000112759 \| SLC29A1 \| -0.86 \| 1.30E-46 \| 1.23E-45 \| \| ENSG00000157637 \| SLC38A10 \| -0.65 \| 1.55E-46 \| 1.46E-45 \| \| ENSG00000182667 \| NTM \| 1.91 \| 1.58E-46 \| 1.49E-45 \| \| ENSG00000196456 \| ZNF775 \| 1.45 \| 1.62E-46 \| 1.53E-45 \| \| ENSG00000186088 \| GSAP \| 1.09 \| 1.66E-46 \| 1.56E-45 \| \| ENSG00000157625 \| TAB3 \| 0.82 \| 2.04E-46 \| 1.92E-45 \| \| ENSG00000196975 \| ANXA4 \| -0.76 \| 2.39E-46 \| 2.25E-45 \| \| ENSG00000253821 \|  \| 1.60 \| 2.76E-46 \| 2.59E-45 \| \| ENSG00000118432 \| CNR1 \| 0.82 \| 2.96E-46 \| 2.78E-45 \| \| ENSG00000139182 \| CLSTN3 \| -2.33 \| 2.98E-46 \| 2.79E-45 \| \| ENSG00000068650 \| ATP11A \| 0.62 \| 3.09E-46 \| 2.90E-45 \| \| ENSG00000131016 \| AKAP12 \| 2.86 \| 3.15E-46 \| 2.95E-45 \| \| ENSG00000104825 \| NFKBIB \| 0.89 \| 3.33E-46 \| 3.12E-45 \| \| ENSG00000198108 \| CHSY3 \| -2.31 \| 3.85E-46 \| 3.60E-45 \| \| ENSG00000163659 \| TIPARP \| 0.79 \| 4.02E-46 \| 3.77E-45 \| \| ENSG00000064012 \| CASP8 \| -0.89 \| 4.19E-46 \| 3.91E-45 \| \| ENSG00000084207 \| GSTP1 \| -0.65 \| 4.21E-46 \| 3.93E-45 \| \| ENSG00000151376 \| ME3 \| -1.29 \| 4.21E-46 \| 3.93E-45 \| \| ENSG00000169093 \| ASMTL \| 0.95 \| 4.62E-46 \| 4.31E-45 \| \| ENSG00000117479 \| SLC19A2 \| 1.79 \| 4.65E-46 \| 4.33E-45 \| \| ENSG00000162702 \| ZNF281 \| 0.64 \| 4.85E-46 \| 4.52E-45 \| \| ENSG00000136840 \| ST6GALNAC4 \| 0.60 \| 5.39E-46 \| 5.01E-45 \| \| ENSG00000167325 \| RRM1 \| -0.87 \| 5.58E-46 \| 5.19E-45 \| \| ENSG00000138496 \| PARP9 \| -1.62 \| 6.89E-46 \| 6.41E-45 \| \| ENSG00000146083 \| RNF44 \| 0.62 \| 8.62E-46 \| 8.00E-45 \| \| ENSG00000111961 \| SASH1 \| 0.69 \| 1.08E-45 \| 1.00E-44 \| \| ENSG00000133639 \| BTG1 \| 0.59 \| 1.08E-45 \| 1.00E-44 \| \| ENSG00000141560 \| FN3KRP \| -1.07 \| 1.16E-45 \| 1.08E-44 \| \| ENSG00000129245 \| FXR2 \| 0.87 \| 1.50E-45 \| 1.39E-44 \| \| ENSG00000160298 \| C21orf58 \| -1.96 \| 1.52E-45 \| 1.40E-44 \| \| ENSG00000044090 \| CUL7 \| 0.86 \| 1.66E-45 \| 1.53E-44 \| \| ENSG00000179300 \| RTL3 \| 1.66 \| 2.17E-45 \| 2.00E-44 \| \| ENSG00000178752 \| ERFE \| -1.06 \| 2.19E-45 \| 2.02E-44 \| \| ENSG00000077152 \| UBE2T \| -1.92 \| 2.27E-45 \| 2.10E-44 \| \| ENSG00000123472 \| ATPAF1 \| -1.26 \| 2.50E-45 \| 2.30E-44 \| \| ENSG00000125733 \| TRIP10 \| 0.73 \| 2.51E-45 \| 2.31E-44 \| \| ENSG00000146386 \| ABRACL \| -0.88 \| 3.54E-45 \| 3.25E-44 \| \| ENSG00000158716 \| DUSP23 \| -0.87 \| 3.70E-45 \| 3.40E-44 \| \| ENSG00000141424 \| SLC39A6 \| 0.73 \| 3.77E-45 \| 3.46E-44 \| \| ENSG00000225339 \|  \| 1.53 \| 4.65E-45 \| 4.26E-44 \| \| ENSG00000161643 \| SIGLEC16 \| -1.99 \| 6.31E-45 \| 5.79E-44 \| \| ENSG00000101003 \| GINS1 \| -1.99 \| 8.19E-45 \| 7.51E-44 \| \| ENSG00000113742 \| CPEB4 \| 1.10 \| 8.23E-45 \| 7.54E-44 \| \| ENSG00000159259 \| CHAF1B \| -1.47 \| 8.35E-45 \| 7.64E-44 \| \| ENSG00000148344 \| PTGES \| 6.96 \| 9.02E-45 \| 8.25E-44 \| \| ENSG00000108039 \| XPNPEP1 \| -0.80 \| 9.36E-45 \| 8.55E-44 \| \| ENSG00000242258 \| LINC00996 \| 2.50 \| 9.72E-45 \| 8.87E-44 \| \| ENSG00000130164 \| LDLR \| 1.14 \| 9.76E-45 \| 8.90E-44 \| \| ENSG00000015171 \| ZMYND11 \| -1.14 \| 9.94E-45 \| 9.06E-44 \| \| ENSG00000109099 \| PMP22 \| 3.09 \| 1.10E-44 \| 1.01E-43 \| \| ENSG00000196843 \| ARID5A \| -0.84 \| 1.15E-44 \| 1.04E-43 \| \| ENSG00000091039 \| OSBPL8 \| 0.66 \| 1.19E-44 \| 1.08E-43 \| \| ENSG00000076003 \| MCM6 \| -0.91 \| 1.70E-44 \| 1.55E-43 \| \| ENSG00000147459 \| DOCK5 \| 0.64 \| 2.41E-44 \| 2.19E-43 \| \| ENSG00000134815 \| DHX34 \| 0.78 \| 2.42E-44 \| 2.20E-43 \| \| ENSG00000138069 \| RAB1A \| 0.62 \| 2.42E-44 \| 2.20E-43 \| \| ENSG00000164463 \| CREBRF \| 1.01 \| 2.53E-44 \| 2.29E-43 \| \| ENSG00000120802 \| TMPO \| -0.75 \| 2.93E-44 \| 2.65E-43 \| \| ENSG00000239713 \| APOBEC3G \| -1.20 \| 2.94E-44 \| 2.66E-43 \| \| ENSG00000137812 \| KNL1 \| -1.69 \| 3.09E-44 \| 2.80E-43 \| \| ENSG00000099194 \| SCD \| -1.42 \| 3.46E-44 \| 3.13E-43 \| \| ENSG00000198355 \| PIM3 \| 1.27 \| 4.04E-44 \| 3.66E-43 \| \| ENSG00000167549 \| CORO6 \| -2.16 \| 4.10E-44 \| 3.70E-43 \| \| ENSG00000118193 \| KIF14 \| -2.49 \| 4.85E-44 \| 4.38E-43 \| \| ENSG00000165023 \| DIRAS2 \| -2.09 \| 5.58E-44 \| 5.03E-43 \| \| ENSG00000162924 \| REL \| 0.94 \| 6.38E-44 \| 5.74E-43 \| \| ENSG00000120725 \| SIL1 \| -0.81 \| 6.60E-44 \| 5.94E-43 \| \| ENSG00000092853 \| CLSPN \| -1.52 \| 7.55E-44 \| 6.78E-43 \| \| ENSG00000085063 \| CD59 \| 0.62 \| 1.03E-43 \| 9.26E-43 \| \| ENSG00000157510 \| AFAP1L1 \| -2.27 \| 1.24E-43 \| 1.11E-42 \| \| ENSG00000085377 \| PREP \| -0.72 \| 1.25E-43 \| 1.12E-42 \| \| ENSG00000149115 \| TNKS1BP1 \| 0.61 \| 1.30E-43 \| 1.16E-42 \| \| ENSG00000119682 \| AREL1 \| 0.64 \| 1.56E-43 \| 1.39E-42 \| \| ENSG00000111452 \| ADGRD1 \| -4.42 \| 1.68E-43 \| 1.50E-42 \| \| ENSG00000100526 \| CDKN3 \| -3.13 \| 1.88E-43 \| 1.68E-42 \| \| ENSG00000184730 \| APOBR \| -0.62 \| 2.00E-43 \| 1.79E-42 \| \| ENSG00000129993 \| CBFA2T3 \| -1.69 \| 2.17E-43 \| 1.93E-42 \| \| ENSG00000125841 \| NRSN2 \| -1.47 \| 2.34E-43 \| 2.09E-42 \| \| ENSG00000043462 \| LCP2 \| -0.67 \| 2.40E-43 \| 2.14E-42 \| \| ENSG00000060982 \| BCAT1 \| 0.75 \| 2.47E-43 \| 2.20E-42 \| \| ENSG00000205476 \| CCDC85C \| -2.06 \| 2.67E-43 \| 2.38E-42 \| \| ENSG00000189057 \| FAM111B \| -2.07 \| 2.95E-43 \| 2.62E-42 \| \| ENSG00000107438 \| PDLIM1 \| -3.22 \| 3.26E-43 \| 2.90E-42 \| \| ENSG00000076641 \| PAG1 \| 0.66 \| 4.12E-43 \| 3.67E-42 \| \| ENSG00000005379 \| TSPOAP1 \| -1.95 \| 4.19E-43 \| 3.72E-42 \| \| ENSG00000177830 \| CHID1 \| -0.84 \| 4.59E-43 \| 4.07E-42 \| \| ENSG00000163629 \| PTPN13 \| 2.63 \| 4.65E-43 \| 4.12E-42 \| \| ENSG00000112139 \| MDGA1 \| -1.55 \| 6.30E-43 \| 5.57E-42 \| \| ENSG00000197780 \| TAF13 \| 0.99 \| 6.35E-43 \| 5.61E-42 \| \| ENSG00000178175 \| ZNF366 \| -2.09 \| 6.98E-43 \| 6.17E-42 \| \| ENSG00000052795 \| FNIP2 \| 0.78 \| 7.29E-43 \| 6.44E-42 \| \| ENSG00000119514 \| GALNT12 \| -3.13 \| 7.33E-43 \| 6.47E-42 \| \| ENSG00000164109 \| MAD2L1 \| -1.66 \| 9.48E-43 \| 8.36E-42 \| \| ENSG00000134247 \| PTGFRN \| -2.08 \| 9.57E-43 \| 8.44E-42 \| \| ENSG00000145833 \| DDX46 \| -0.69 \| 1.01E-42 \| 8.90E-42 \| \| ENSG00000107738 \| VSIR \| -0.96 \| 1.08E-42 \| 9.55E-42 \| \| ENSG00000135439 \| AGAP2 \| -1.19 \| 1.10E-42 \| 9.68E-42 \| \| ENSG00000095397 \| WHRN \| -1.32 \| 1.10E-42 \| 9.71E-42 \| \| ENSG00000233922 \| LINC01694 \| -3.85 \| 1.16E-42 \| 1.02E-41 \| \| ENSG00000168172 \| HOOK3 \| 0.79 \| 1.25E-42 \| 1.10E-41 \| \| ENSG00000271383 \| NOTCH2NLC \| 1.54 \| 1.26E-42 \| 1.10E-41 \| \| ENSG00000271383 \| NBPF19 \| 1.54 \| 1.26E-42 \| 1.10E-41 \| \| ENSG00000130311 \| DDA1 \| 0.62 \| 1.28E-42 \| 1.12E-41 \| \| ENSG00000236104 \| ZBTB22 \| 0.80 \| 1.35E-42 \| 1.18E-41 \| \| ENSG00000127884 \| ECHS1 \| -1.00 \| 1.38E-42 \| 1.21E-41 \| \| ENSG00000156052 \| GNAQ \| -0.68 \| 1.40E-42 \| 1.23E-41 \| \| ENSG00000100299 \| ARSA \| -0.72 \| 1.46E-42 \| 1.27E-41 \| \| ENSG00000139192 \| TAPBPL \| -1.52 \| 1.47E-42 \| 1.28E-41 \| \| ENSG00000152413 \| HOMER1 \| 1.18 \| 1.48E-42 \| 1.29E-41 \| \| ENSG00000167081 \| PBX3 \| -0.67 \| 1.49E-42 \| 1.30E-41 \| \| ENSG00000120051 \| CFAP58 \| 1.54 \| 1.51E-42 \| 1.31E-41 \| \| ENSG00000139734 \| DIAPH3 \| -2.18 \| 1.70E-42 \| 1.48E-41 \| \| ENSG00000113448 \| PDE4D \| 1.54 \| 1.73E-42 \| 1.51E-41 \| \| ENSG00000065911 \| MTHFD2 \| 0.66 \| 1.73E-42 \| 1.51E-41 \| \| ENSG00000130787 \| HIP1R \| -1.11 \| 1.75E-42 \| 1.53E-41 \| \| ENSG00000080839 \| RBL1 \| -1.25 \| 2.19E-42 \| 1.90E-41 \| \| ENSG00000276045 \| ORAI1 \| -1.11 \| 2.21E-42 \| 1.92E-41 \| \| ENSG00000100065 \| CARD10 \| 2.08 \| 2.60E-42 \| 2.26E-41 \| \| ENSG00000103111 \| MON1B \| 0.62 \| 2.92E-42 \| 2.54E-41 \| \| ENSG00000062822 \| POLD1 \| -0.92 \| 3.09E-42 \| 2.68E-41 \| \| ENSG00000157456 \| CCNB2 \| -1.71 \| 3.25E-42 \| 2.82E-41 \| \| ENSG00000129250 \| KIF1C \| -0.71 \| 4.06E-42 \| 3.52E-41 \| \| ENSG00000144228 \| SPOPL \| 1.29 \| 4.58E-42 \| 3.97E-41 \| \| ENSG00000170989 \| S1PR1 \| -6.69 \| 4.97E-42 \| 4.30E-41 \| \| ENSG00000106829 \| TLE4 \| -0.70 \| 5.32E-42 \| 4.60E-41 \| \| ENSG00000118292 \| C1orf54 \| -1.23 \| 5.83E-42 \| 5.04E-41 \| \| ENSG00000150403 \| TMCO3 \| 0.72 \| 6.00E-42 \| 5.18E-41 \| \| ENSG00000171608 \| PIK3CD \| -0.93 \| 6.43E-42 \| 5.55E-41 \| \| ENSG00000196998 \| WDR45 \| 0.74 \| 6.93E-42 \| 5.98E-41 \| \| ENSG00000171236 \| LRG1 \| -3.57 \| 7.53E-42 \| 6.49E-41 \| \| ENSG00000276600 \| RAB7B \| -1.53 \| 7.77E-42 \| 6.69E-41 \| \| ENSG00000173451 \| THAP2 \| 1.38 \| 8.42E-42 \| 7.25E-41 \| \| ENSG00000154447 \| SH3RF1 \| 0.72 \| 8.71E-42 \| 7.49E-41 \| \| ENSG00000119699 \| TGFB3 \| 1.16 \| 9.13E-42 \| 7.85E-41 \| \| ENSG00000196850 \| PPTC7 \| 0.74 \| 9.70E-42 \| 8.33E-41 \| \| ENSG00000181577 \| C6orf223 \| 2.36 \| 1.04E-41 \| 8.92E-41 \| \| ENSG00000099942 \| CRKL \| 0.65 \| 1.10E-41 \| 9.40E-41 \| \| ENSG00000143486 \| EIF2D \| -0.92 \| 1.22E-41 \| 1.05E-40 \| \| ENSG00000125868 \| DSTN \| -0.71 \| 1.32E-41 \| 1.13E-40 \| \| ENSG00000152284 \| TCF7L1 \| -2.33 \| 1.35E-41 \| 1.16E-40 \| \| ENSG00000116032 \| GRIN3B \| -2.15 \| 1.47E-41 \| 1.26E-40 \| \| ENSG00000169607 \| CKAP2L \| -1.55 \| 1.48E-41 \| 1.26E-40 \| \| ENSG00000105792 \| CFAP69 \| 2.89 \| 1.52E-41 \| 1.30E-40 \| \| ENSG00000112837 \| TBX18 \| -1.14 \| 1.79E-41 \| 1.53E-40 \| \| ENSG00000254415 \| SIGLEC14 \| -2.25 \| 2.29E-41 \| 1.95E-40 \| \| ENSG00000101955 \| SRPX \| 2.05 \| 2.58E-41 \| 2.20E-40 \| \| ENSG00000167645 \| YIF1B \| -1.04 \| 2.84E-41 \| 2.42E-40 \| \| ENSG00000160094 \| ZNF362 \| -0.97 \| 2.88E-41 \| 2.45E-40 \| \| ENSG00000174943 \| KCTD13 \| 0.78 \| 2.98E-41 \| 2.54E-40 \| \| ENSG00000181409 \| AATK \| -2.71 \| 3.04E-41 \| 2.58E-40 \| \| ENSG00000115457 \| IGFBP2 \| -2.83 \| 3.12E-41 \| 2.65E-40 \| \| ENSG00000186439 \| TRDN \| -2.73 \| 3.43E-41 \| 2.91E-40 \| \| ENSG00000117228 \| GBP1 \| -4.21 \| 3.53E-41 \| 2.99E-40 \| \| ENSG00000133169 \| BEX1 \| -3.71 \| 3.59E-41 \| 3.04E-40 \| \| ENSG00000143322 \| ABL2 \| 1.29 \| 3.62E-41 \| 3.07E-40 \| \| ENSG00000142327 \| RNPEPL1 \| -0.59 \| 3.98E-41 \| 3.37E-40 \| \| ENSG00000168078 \| PBK \| -2.35 \| 4.03E-41 \| 3.41E-40 \| \| ENSG00000126773 \| PCNX4 \| 0.88 \| 4.32E-41 \| 3.65E-40 \| \| ENSG00000108798 \| ABI3 \| -2.57 \| 4.63E-41 \| 3.91E-40 \| \| ENSG00000184557 \| SOCS3 \| 3.13 \| 4.97E-41 \| 4.20E-40 \| \| ENSG00000130208 \| APOC1 \| -1.50 \| 5.62E-41 \| 4.74E-40 \| \| ENSG00000253123 \|  \| -1.25 \| 6.21E-41 \| 5.23E-40 \| \| ENSG00000090661 \| CERS4 \| -1.97 \| 6.32E-41 \| 5.32E-40 \| \| ENSG00000096996 \| IL12RB1 \| -2.59 \| 6.32E-41 \| 5.32E-40 \| \| ENSG00000179750 \| APOBEC3B \| -2.59 \| 6.57E-41 \| 5.53E-40 \| \| ENSG00000148908 \| RGS10 \| -0.66 \| 7.55E-41 \| 6.35E-40 \| \| ENSG00000013364 \| MVP \| -0.73 \| 9.55E-41 \| 8.03E-40 \| \| ENSG00000104687 \| GSR \| -0.60 \| 1.48E-40 \| 1.24E-39 \| \| ENSG00000138448 \| ITGAV \| 0.67 \| 1.57E-40 \| 1.32E-39 \| \| ENSG00000146834 \| MEPCE \| 0.62 \| 1.67E-40 \| 1.40E-39 \| \| ENSG00000055211 \| GINM1 \| 0.74 \| 1.76E-40 \| 1.47E-39 \| \| ENSG00000103319 \| EEF2K \| -0.90 \| 1.78E-40 \| 1.49E-39 \| \| ENSG00000117650 \| NEK2 \| -3.04 \| 1.88E-40 \| 1.57E-39 \| \| ENSG00000119906 \| SLF2 \| -1.31 \| 2.02E-40 \| 1.69E-39 \| \| ENSG00000130338 \| TULP4 \| 0.91 \| 2.16E-40 \| 1.80E-39 \| \| ENSG00000111266 \| DUSP16 \| 1.49 \| 2.25E-40 \| 1.88E-39 \| \| ENSG00000156711 \| MAPK13 \| -0.94 \| 2.42E-40 \| 2.02E-39 \| \| ENSG00000145014 \| TMEM44 \| -1.25 \| 2.74E-40 \| 2.28E-39 \| \| ENSG00000132646 \| PCNA \| -1.14 \| 3.00E-40 \| 2.50E-39 \| \| ENSG00000188130 \| MAPK12 \| -0.95 \| 3.05E-40 \| 2.54E-39 \| \| ENSG00000085999 \| RAD54L \| -1.78 \| 3.57E-40 \| 2.97E-39 \| \| ENSG00000188549 \| CCDC9B \| -2.05 \| 3.57E-40 \| 2.97E-39 \| \| ENSG00000164418 \| GRIK2 \| 1.60 \| 3.92E-40 \| 3.26E-39 \| \| ENSG00000100485 \| SOS2 \| 0.66 \| 4.51E-40 \| 3.75E-39 \| \| ENSG00000107745 \| MICU1 \| -0.93 \| 5.26E-40 \| 4.36E-39 \| \| ENSG00000163320 \| CGGBP1 \| 0.61 \| 6.23E-40 \| 5.16E-39 \| \| ENSG00000076604 \| TRAF4 \| 0.94 \| 6.34E-40 \| 5.25E-39 \| \| ENSG00000100504 \| PYGL \| -0.76 \| 7.08E-40 \| 5.86E-39 \| \| ENSG00000155265 \| GOLGA7B \| -1.83 \| 7.37E-40 \| 6.10E-39 \| \| ENSG00000100413 \| POLR3H \| 0.69 \| 7.40E-40 \| 6.12E-39 \| \| ENSG00000165140 \| FBP1 \| -5.93 \| 7.91E-40 \| 6.54E-39 \| \| ENSG00000143847 \| PPFIA4 \| -2.16 \| 8.96E-40 \| 7.40E-39 \| \| ENSG00000179348 \| GATA2 \| -2.00 \| 1.00E-39 \| 8.26E-39 \| \| ENSG00000132383 \| RPA1 \| -0.88 \| 1.07E-39 \| 8.82E-39 \| \| ENSG00000250771 \| LOC100419170 \| 2.39 \| 1.08E-39 \| 8.92E-39 \| \| ENSG00000160058 \| BSDC1 \| 0.65 \| 1.12E-39 \| 9.25E-39 \| \| ENSG00000130300 \| PLVAP \| 1.21 \| 1.13E-39 \| 9.28E-39 \| \| ENSG00000168397 \| ATG4B \| -0.87 \| 1.20E-39 \| 9.86E-39 \| \| ENSG00000061273 \| HDAC7 \| 0.67 \| 1.22E-39 \| 1.01E-38 \| \| ENSG00000188747 \| NOXA1 \| -2.37 \| 1.36E-39 \| 1.12E-38 \| \| ENSG00000161888 \| SPC24 \| -2.80 \| 1.38E-39 \| 1.14E-38 \| \| ENSG00000163220 \| S100A9 \| -1.48 \| 1.60E-39 \| 1.32E-38 \| \| ENSG00000178761 \| FAM219B \| -0.71 \| 1.73E-39 \| 1.42E-38 \| \| ENSG00000162066 \| AMDHD2 \| -1.21 \| 2.04E-39 \| 1.67E-38 \| \| ENSG00000235106 \| BRD3OS \| 0.74 \| 2.05E-39 \| 1.68E-38 \| \| ENSG00000119866 \| BCL11A \| 2.35 \| 2.08E-39 \| 1.70E-38 \| \| ENSG00000177508 \| IRX3 \| 0.82 \| 2.80E-39 \| 2.29E-38 \| \| ENSG00000175489 \| LRRC25 \| -0.86 \| 2.93E-39 \| 2.39E-38 \| \| ENSG00000196476 \| C20orf96 \| -1.56 \| 2.93E-39 \| 2.39E-38 \| \| ENSG00000108813 \| DLX4 \| -2.24 \| 2.94E-39 \| 2.40E-38 \| \| ENSG00000124145 \| SDC4 \| 0.88 \| 3.50E-39 \| 2.86E-38 \| \| ENSG00000051341 \| POLQ \| -2.37 \| 5.06E-39 \| 4.13E-38 \| \| ENSG00000189060 \| H1F0 \| 0.63 \| 5.93E-39 \| 4.83E-38 \| \| ENSG00000160145 \| KALRN \| -3.26 \| 6.45E-39 \| 5.25E-38 \| \| ENSG00000115464 \| USP34 \| 0.72 \| 6.86E-39 \| 5.58E-38 \| \| ENSG00000165637 \| VDAC2 \| -0.85 \| 7.33E-39 \| 5.96E-38 \| \| ENSG00000140853 \| NLRC5 \| 0.64 \| 7.37E-39 \| 5.99E-38 \| \| ENSG00000187840 \| EIF4EBP1 \| -0.65 \| 7.48E-39 \| 6.08E-38 \| \| ENSG00000182022 \| CHST15 \| -0.68 \| 7.56E-39 \| 6.14E-38 \| \| ENSG00000071539 \| TRIP13 \| -1.58 \| 8.17E-39 \| 6.63E-38 \| \| ENSG00000137474 \| MYO7A \| -2.69 \| 9.26E-39 \| 7.51E-38 \| \| ENSG00000105767 \| CADM4 \| 1.85 \| 9.70E-39 \| 7.86E-38 \| \| ENSG00000129625 \| REEP5 \| -0.77 \| 1.01E-38 \| 8.22E-38 \| \| ENSG00000275302 \| CCL4 \| 2.54 \| 1.04E-38 \| 8.40E-38 \| \| ENSG00000138613 \| APH1B \| 0.98 \| 1.15E-38 \| 9.28E-38 \| \| ENSG00000086289 \| EPDR1 \| -1.72 \| 1.26E-38 \| 1.02E-37 \| \| ENSG00000198892 \| SHISA4 \| -0.87 \| 1.26E-38 \| 1.02E-37 \| \| ENSG00000087903 \| RFX2 \| 0.71 \| 1.42E-38 \| 1.15E-37 \| \| ENSG00000075884 \| ARHGAP15 \| -1.38 \| 1.42E-38 \| 1.15E-37 \| \| ENSG00000154640 \| BTG3 \| 0.96 \| 1.55E-38 \| 1.25E-37 \| \| ENSG00000164045 \| CDC25A \| -1.51 \| 1.58E-38 \| 1.27E-37 \| \| ENSG00000146263 \| MMS22L \| -1.96 \| 2.26E-38 \| 1.82E-37 \| \| ENSG00000143398 \| PIP5K1A \| 0.73 \| 2.41E-38 \| 1.94E-37 \| \| ENSG00000198520 \| ARMH1 \| -1.14 \| 2.65E-38 \| 2.13E-37 \| \| ENSG00000141232 \| TOB1 \| -0.83 \| 2.68E-38 \| 2.16E-37 \| \| ENSG00000113494 \| PRLR \| 3.34 \| 3.00E-38 \| 2.41E-37 \| \| ENSG00000175857 \| GAPT \| -5.58 \| 3.11E-38 \| 2.50E-37 \| \| ENSG00000140749 \| IGSF6 \| -3.19 \| 3.17E-38 \| 2.54E-37 \| \| ENSG00000118946 \| PCDH17 \| 3.48 \| 3.24E-38 \| 2.60E-37 \| \| ENSG00000127526 \| SLC35E1 \| 0.63 \| 3.44E-38 \| 2.76E-37 \| \| ENSG00000121743 \| GJA3 \| -1.92 \| 3.47E-38 \| 2.78E-37 \| \| ENSG00000102445 \| RUBCNL \| -0.98 \| 3.56E-38 \| 2.85E-37 \| \| ENSG00000198026 \| ZNF335 \| 0.67 \| 4.06E-38 \| 3.25E-37 \| \| ENSG00000117280 \| RAB29 \| -0.64 \| 5.09E-38 \| 4.07E-37 \| \| ENSG00000014138 \| POLA2 \| -1.06 \| 5.30E-38 \| 4.24E-37 \| \| ENSG00000183621 \| ZNF438 \| -0.96 \| 5.45E-38 \| 4.35E-37 \| \| ENSG00000109667 \| SLC2A9 \| -1.56 \| 5.87E-38 \| 4.69E-37 \| \| ENSG00000070190 \| DAPP1 \| -2.44 \| 5.88E-38 \| 4.69E-37 \| \| ENSG00000129116 \| PALLD \| -1.36 \| 6.39E-38 \| 5.10E-37 \| \| ENSG00000145107 \| TM4SF19 \| -1.34 \| 6.68E-38 \| 5.32E-37 \| \| ENSG00000145685 \| LHFPL2 \| 0.89 \| 8.16E-38 \| 6.49E-37 \| \| ENSG00000072274 \| TFRC \| 0.67 \| 8.22E-38 \| 6.54E-37 \| \| ENSG00000246695 \| RASSF8-AS1 \| 1.65 \| 8.63E-38 \| 6.86E-37 \| \| ENSG00000206190 \| ATP10A \| -1.20 \| 8.87E-38 \| 7.05E-37 \| \| ENSG00000141337 \| ARSG \| 2.08 \| 9.41E-38 \| 7.47E-37 \| \| ENSG00000108439 \| PNPO \| -0.82 \| 1.01E-37 \| 8.03E-37 \| \| ENSG00000215039 \| CD27-AS1 \| -1.85 \| 1.05E-37 \| 8.32E-37 \| \| ENSG00000203685 \| STUM \| -3.16 \| 1.10E-37 \| 8.71E-37 \| \| ENSG00000123610 \| TNFAIP6 \| 5.03 \| 1.20E-37 \| 9.49E-37 \| \| ENSG00000136169 \| SETDB2 \| -0.96 \| 1.24E-37 \| 9.84E-37 \| \| ENSG00000166073 \| GPR176 \| 2.54 \| 1.24E-37 \| 9.84E-37 \| \| ENSG00000164746 \| C7orf57 \| 3.99 \| 1.28E-37 \| 1.01E-36 \| \| ENSG00000184979 \| USP18 \| -4.09 \| 1.29E-37 \| 1.02E-36 \| \| ENSG00000137509 \| PRCP \| -0.86 \| 1.35E-37 \| 1.07E-36 \| \| ENSG00000017483 \| SLC38A5 \| -1.63 \| 1.55E-37 \| 1.23E-36 \| \| ENSG00000174945 \| AMZ1 \| 2.70 \| 1.63E-37 \| 1.29E-36 \| \| ENSG00000182557 \| SPNS3 \| -3.01 \| 1.74E-37 \| 1.37E-36 \| \| ENSG00000101425 \| BPI \| 1.96 \| 1.75E-37 \| 1.38E-36 \| \| ENSG00000153147 \| SMARCA5 \| 0.60 \| 1.81E-37 \| 1.43E-36 \| \| ENSG00000229644 \|  \| 2.19 \| 2.04E-37 \| 1.61E-36 \| \| ENSG00000136869 \| TLR4 \| -0.79 \| 2.07E-37 \| 1.63E-36 \| \| ENSG00000153993 \| SEMA3D \| 1.04 \| 2.61E-37 \| 2.05E-36 \| \| ENSG00000162434 \| JAK1 \| 0.60 \| 2.71E-37 \| 2.13E-36 \| \| ENSG00000164611 \| PTTG1 \| -1.77 \| 2.73E-37 \| 2.14E-36 \| \| ENSG00000065154 \| OAT \| -1.00 \| 2.80E-37 \| 2.19E-36 \| \| ENSG00000125968 \| ID1 \| -1.37 \| 3.19E-37 \| 2.50E-36 \| \| ENSG00000203668 \| CHML \| 0.99 \| 3.55E-37 \| 2.79E-36 \| \| ENSG00000105723 \| GSK3A \| 0.62 \| 3.63E-37 \| 2.85E-36 \| \| ENSG00000076554 \| TPD52 \| -3.40 \| 3.65E-37 \| 2.86E-36 \| \| ENSG00000186591 \| UBE2H \| 0.60 \| 3.72E-37 \| 2.91E-36 \| \| ENSG00000213626 \| LBH \| -0.64 \| 4.67E-37 \| 3.65E-36 \| \| ENSG00000259436 \|  \| 1.72 \| 4.78E-37 \| 3.74E-36 \| \| ENSG00000128585 \| MKLN1 \| 0.77 \| 5.14E-37 \| 4.01E-36 \| \| ENSG00000152952 \| PLOD2 \| 2.22 \| 5.25E-37 \| 4.10E-36 \| \| ENSG00000100427 \| MLC1 \| -4.53 \| 5.65E-37 \| 4.40E-36 \| \| ENSG00000172466 \| ZNF24 \| 0.64 \| 5.86E-37 \| 4.56E-36 \| \| ENSG00000172594 \| SMPDL3A \| 0.83 \| 6.78E-37 \| 5.28E-36 \| \| ENSG00000131697 \| NPHP4 \| 0.87 \| 7.15E-37 \| 5.56E-36 \| \| ENSG00000135451 \| TROAP \| -3.10 \| 7.39E-37 \| 5.74E-36 \| \| ENSG00000165672 \| PRDX3 \| -0.59 \| 7.62E-37 \| 5.92E-36 \| \| ENSG00000179715 \| PCED1B \| -2.39 \| 8.11E-37 \| 6.30E-36 \| \| ENSG00000118473 \| SGIP1 \| 1.32 \| 8.22E-37 \| 6.39E-36 \| \| ENSG00000109466 \| KLHL2 \| 0.73 \| 8.53E-37 \| 6.62E-36 \| \| ENSG00000133678 \| TMEM254 \| -1.20 \| 1.03E-36 \| 8.00E-36 \| \| ENSG00000206052 \| DOK6 \| 2.63 \| 1.07E-36 \| 8.28E-36 \| \| ENSG00000116128 \| BCL9 \| 1.19 \| 1.19E-36 \| 9.25E-36 \| \| ENSG00000005073 \| HOXA11 \| -1.76 \| 1.35E-36 \| 1.04E-35 \| \| ENSG00000135631 \| RAB11FIP5 \| 0.66 \| 1.38E-36 \| 1.07E-35 \| \| ENSG00000079616 \| KIF22 \| -0.76 \| 1.50E-36 \| 1.16E-35 \| \| ENSG00000026751 \| SLAMF7 \| -1.21 \| 1.53E-36 \| 1.18E-35 \| \| ENSG00000163956 \| LRPAP1 \| -0.60 \| 1.69E-36 \| 1.31E-35 \| \| ENSG00000134909 \| ARHGAP32 \| 0.90 \| 1.90E-36 \| 1.46E-35 \| \| ENSG00000173391 \| OLR1 \| 0.92 \| 2.10E-36 \| 1.62E-35 \| \| ENSG00000133019 \| CHRM3 \| -0.82 \| 2.47E-36 \| 1.90E-35 \| \| ENSG00000187801 \| ZFP69B \| 0.65 \| 2.61E-36 \| 2.00E-35 \| \| ENSG00000118200 \| CAMSAP2 \| 0.69 \| 2.63E-36 \| 2.02E-35 \| \| ENSG00000100281 \| HMGXB4 \| 0.66 \| 2.73E-36 \| 2.09E-35 \| \| ENSG00000214194 \| SMIM30 \| -1.86 \| 2.87E-36 \| 2.20E-35 \| \| ENSG00000061337 \| LZTS1 \| -1.81 \| 2.89E-36 \| 2.22E-35 \| \| ENSG00000176597 \| B3GNT5 \| -1.80 \| 2.91E-36 \| 2.23E-35 \| \| ENSG00000126947 \| ARMCX1 \| -1.43 \| 2.94E-36 \| 2.25E-35 \| \| ENSG00000148411 \| NACC2 \| -0.80 \| 3.03E-36 \| 2.32E-35 \| \| ENSG00000112667 \| DNPH1 \| -0.72 \| 3.06E-36 \| 2.34E-35 \| \| ENSG00000138279 \| ANXA7 \| -0.79 \| 3.12E-36 \| 2.38E-35 \| \| ENSG00000164933 \| SLC25A32 \| 0.66 \| 3.15E-36 \| 2.41E-35 \| \| ENSG00000029993 \| HMGB3 \| 0.65 \| 3.23E-36 \| 2.47E-35 \| \| ENSG00000140534 \| TICRR \| -1.91 \| 3.24E-36 \| 2.48E-35 \| \| ENSG00000119912 \| IDE \| -1.21 \| 3.28E-36 \| 2.50E-35 \| \| ENSG00000118523 \| CTGF \| 2.08 \| 3.35E-36 \| 2.56E-35 \| \| ENSG00000205086 \| C2orf91 \| -3.92 \| 3.60E-36 \| 2.75E-35 \| \| ENSG00000130304 \| SLC27A1 \| 0.71 \| 3.69E-36 \| 2.81E-35 \| \| ENSG00000197343 \| ZNF655 \| 0.71 \| 3.89E-36 \| 2.97E-35 \| \| ENSG00000100614 \| PPM1A \| 0.69 \| 4.04E-36 \| 3.08E-35 \| \| ENSG00000058056 \| USP13 \| 0.87 \| 4.35E-36 \| 3.31E-35 \| \| ENSG00000136378 \| ADAMTS7 \| 1.54 \| 5.08E-36 \| 3.86E-35 \| \| ENSG00000124181 \| PLCG1 \| -0.94 \| 5.49E-36 \| 4.17E-35 \| \| ENSG00000116991 \| SIPA1L2 \| 0.88 \| 5.80E-36 \| 4.40E-35 \| \| ENSG00000132965 \| ALOX5AP \| -0.60 \| 5.85E-36 \| 4.44E-35 \| \| ENSG00000095002 \| MSH2 \| -1.17 \| 6.55E-36 \| 4.97E-35 \| \| ENSG00000157873 \| TNFRSF14 \| -0.97 \| 6.70E-36 \| 5.08E-35 \| \| ENSG00000152253 \| SPC25 \| -2.38 \| 6.86E-36 \| 5.19E-35 \| \| ENSG00000116957 \|  \| -0.85 \| 7.36E-36 \| 5.57E-35 \| \| ENSG00000171320 \| ESCO2 \| -1.56 \| 7.80E-36 \| 5.91E-35 \| \| ENSG00000198879 \| SFMBT2 \| -0.82 \| 8.08E-36 \| 6.11E-35 \| \| ENSG00000147679 \| UTP23 \| 0.73 \| 8.17E-36 \| 6.18E-35 \| \| ENSG00000164405 \| UQCRQ \| -0.99 \| 1.09E-35 \| 8.20E-35 \| \| ENSG00000163041 \| H3F3A \| -0.78 \| 1.16E-35 \| 8.73E-35 \| \| ENSG00000173264 \| GPR137 \| 0.76 \| 1.33E-35 \| 1.00E-34 \| \| ENSG00000127663 \| KDM4B \| 0.70 \| 1.34E-35 \| 1.01E-34 \| \| ENSG00000257335 \| MGAM \| 2.53 \| 1.45E-35 \| 1.09E-34 \| \| ENSG00000004799 \| PDK4 \| -2.07 \| 1.50E-35 \| 1.13E-34 \| \| ENSG00000168672 \| FAM84B \| -2.16 \| 1.50E-35 \| 1.13E-34 \| \| ENSG00000173915 \| ATP5MD \| -0.95 \| 1.51E-35 \| 1.14E-34 \| \| ENSG00000126262 \| FFAR2 \| -2.67 \| 1.54E-35 \| 1.16E-34 \| \| ENSG00000131067 \| GGT7 \| 1.19 \| 1.77E-35 \| 1.33E-34 \| \| ENSG00000119446 \| RBM18 \| 0.82 \| 1.84E-35 \| 1.38E-34 \| \| ENSG00000196535 \| MYO18A \| -0.84 \| 1.89E-35 \| 1.42E-34 \| \| ENSG00000213563 \| C8orf82 \| 0.74 \| 1.96E-35 \| 1.47E-34 \| \| ENSG00000162607 \| USP1 \| -0.88 \| 2.01E-35 \| 1.50E-34 \| \| ENSG00000118762 \| PKD2 \| 0.68 \| 2.10E-35 \| 1.57E-34 \| \| ENSG00000131899 \| LLGL1 \| 0.96 \| 2.15E-35 \| 1.60E-34 \| \| ENSG00000172939 \| OXSR1 \| 0.72 \| 2.70E-35 \| 2.01E-34 \| \| ENSG00000064300 \| NGFR \| -4.17 \| 2.74E-35 \| 2.05E-34 \| \| ENSG00000150967 \| ABCB9 \| -1.35 \| 2.90E-35 \| 2.16E-34 \| \| ENSG00000173207 \| CKS1B \| -1.08 \| 3.17E-35 \| 2.37E-34 \| \| ENSG00000135336 \| ORC3 \| -1.01 \| 3.25E-35 \| 2.43E-34 \| \| ENSG00000101849 \| TBL1X \| -1.90 \| 3.31E-35 \| 2.47E-34 \| \| ENSG00000174405 \| LIG4 \| 0.96 \| 3.37E-35 \| 2.51E-34 \| \| ENSG00000116750 \| UCHL5 \| -0.76 \| 3.72E-35 \| 2.77E-34 \| \| ENSG00000184635 \| ZNF93 \| -1.11 \| 3.85E-35 \| 2.87E-34 \| \| ENSG00000176842 \| IRX5 \| 1.06 \| 4.25E-35 \| 3.16E-34 \| \| ENSG00000173418 \| NAA20 \| -0.66 \| 4.44E-35 \| 3.30E-34 \| \| ENSG00000138111 \| MFSD13A \| -1.38 \| 4.84E-35 \| 3.59E-34 \| \| ENSG00000270069 \|  \| 1.39 \| 4.88E-35 \| 3.62E-34 \| \| ENSG00000147155 \| EBP \| -0.97 \| 5.10E-35 \| 3.78E-34 \| \| ENSG00000107338 \| SHB \| 1.25 \| 5.41E-35 \| 4.02E-34 \| \| ENSG00000128274 \| A4GALT \| 0.78 \| 5.46E-35 \| 4.05E-34 \| \| ENSG00000116906 \| GNPAT \| -0.77 \| 5.56E-35 \| 4.12E-34 \| \| ENSG00000164032 \| H2AFZ \| -0.69 \| 6.53E-35 \| 4.83E-34 \| \| ENSG00000056972 \| TRAF3IP2 \| 0.76 \| 7.31E-35 \| 5.41E-34 \| \| ENSG00000118454 \| ANKRD13C \| 0.75 \| 7.71E-35 \| 5.70E-34 \| \| ENSG00000213523 \| SRA1 \| 0.63 \| 8.85E-35 \| 6.54E-34 \| \| ENSG00000226562 \|  \| 4.85 \| 9.01E-35 \| 6.65E-34 \| \| ENSG00000110492 \| MDK \| -2.13 \| 9.60E-35 \| 7.08E-34 \| \| ENSG00000178999 \| AURKB \| -2.53 \| 9.71E-35 \| 7.16E-34 \| \| ENSG00000147457 \| CHMP7 \| 0.62 \| 1.04E-34 \| 7.65E-34 \| \| ENSG00000119242 \| CCDC92 \| -0.70 \| 1.12E-34 \| 8.22E-34 \| \| ENSG00000124766 \| SOX4 \| 0.60 \| 1.20E-34 \| 8.79E-34 \| \| ENSG00000156127 \| BATF \| -1.29 \| 1.20E-34 \| 8.83E-34 \| \| ENSG00000162063 \| CCNF \| -1.13 \| 1.23E-34 \| 9.06E-34 \| \| ENSG00000146192 \| FGD2 \| -4.60 \| 1.74E-34 \| 1.27E-33 \| \| ENSG00000134779 \| TPGS2 \| -0.61 \| 1.83E-34 \| 1.34E-33 \| \| ENSG00000101493 \| ZNF516 \| -0.62 \| 2.11E-34 \| 1.54E-33 \| \| ENSG00000116830 \| TTF2 \| -1.13 \| 2.26E-34 \| 1.65E-33 \| \| ENSG00000118898 \| PPL \| 2.75 \| 2.53E-34 \| 1.85E-33 \| \| ENSG00000168246 \| UBTD2 \| 0.87 \| 2.59E-34 \| 1.89E-33 \| \| ENSG00000227671 \|  \| -0.77 \| 2.76E-34 \| 2.01E-33 \| \| ENSG00000138600 \| SPPL2A \| 0.62 \| 2.78E-34 \| 2.03E-33 \| \| ENSG00000155011 \| DKK2 \| 1.36 \| 2.90E-34 \| 2.12E-33 \| \| ENSG00000162542 \| TMCO4 \| -1.63 \| 2.96E-34 \| 2.16E-33 \| \| ENSG00000162946 \| DISC1 \| -0.89 \| 3.03E-34 \| 2.20E-33 \| \| ENSG00000114812 \| VIPR1 \| -4.49 \| 3.13E-34 \| 2.28E-33 \| \| ENSG00000165209 \| STRBP \| -0.99 \| 3.44E-34 \| 2.50E-33 \| \| ENSG00000140538 \| NTRK3 \| 2.51 \| 3.60E-34 \| 2.61E-33 \| \| ENSG00000049247 \| UTS2 \| -2.15 \| 3.67E-34 \| 2.67E-33 \| \| ENSG00000187678 \| SPRY4 \| 0.84 \| 3.80E-34 \| 2.76E-33 \| \| ENSG00000196914 \| ARHGEF12 \| 0.73 \| 3.87E-34 \| 2.81E-33 \| \| ENSG00000105939 \| ZC3HAV1 \| -0.61 \| 4.02E-34 \| 2.91E-33 \| \| ENSG00000158882 \| TOMM40L \| -1.01 \| 4.12E-34 \| 2.99E-33 \| \| ENSG00000119403 \| PHF19 \| -0.75 \| 4.42E-34 \| 3.20E-33 \| \| ENSG00000172831 \| CES2 \| 0.59 \| 4.94E-34 \| 3.57E-33 \| \| ENSG00000113739 \| STC2 \| 1.48 \| 5.07E-34 \| 3.66E-33 \| \| ENSG00000178226 \| PRSS36 \| -1.66 \| 5.32E-34 \| 3.84E-33 \| \| ENSG00000143643 \| TTC13 \| -0.85 \| 5.96E-34 \| 4.30E-33 \| \| ENSG00000185112 \| FAM43A \| 1.18 \| 5.97E-34 \| 4.31E-33 \| \| ENSG00000161640 \| SIGLEC11 \| -1.70 \| 6.05E-34 \| 4.37E-33 \| \| ENSG00000178585 \| CTNNBIP1 \| -1.33 \| 6.33E-34 \| 4.57E-33 \| \| ENSG00000180182 \| MED14 \| 0.59 \| 6.62E-34 \| 4.77E-33 \| \| ENSG00000271503 \| CCL5 \| -1.46 \| 7.16E-34 \| 5.16E-33 \| \| ENSG00000144354 \| CDCA7 \| -2.49 \| 7.20E-34 \| 5.18E-33 \| \| ENSG00000136877 \| FPGS \| 0.59 \| 7.42E-34 \| 5.34E-33 \| \| ENSG00000165752 \| STK32C \| -1.24 \| 7.67E-34 \| 5.52E-33 \| \| ENSG00000178199 \| ZC3H12D \| -0.76 \| 7.75E-34 \| 5.57E-33 \| \| ENSG00000283652 \|  \| 0.83 \| 8.43E-34 \| 6.05E-33 \| \| ENSG00000184164 \| CRELD2 \| -0.78 \| 9.65E-34 \| 6.92E-33 \| \| ENSG00000178568 \| ERBB4 \| 2.17 \| 1.00E-33 \| 7.19E-33 \| \| ENSG00000173581 \| CCDC106 \| -1.10 \| 1.08E-33 \| 7.72E-33 \| \| ENSG00000173083 \| HPSE \| -1.21 \| 1.09E-33 \| 7.83E-33 \| \| ENSG00000069248 \| NUP133 \| -0.68 \| 1.14E-33 \| 8.19E-33 \| \| ENSG00000104375 \| STK3 \| 0.89 \| 1.21E-33 \| 8.62E-33 \| \| ENSG00000108406 \| DHX40 \| 0.80 \| 1.44E-33 \| 1.03E-32 \| \| ENSG00000165322 \| ARHGAP12 \| -1.03 \| 1.53E-33 \| 1.09E-32 \| \| ENSG00000180539 \| C9orf139 \| -1.37 \| 1.57E-33 \| 1.12E-32 \| \| ENSG00000163032 \| VSNL1 \| 2.99 \| 1.62E-33 \| 1.16E-32 \| \| ENSG00000124782 \| RREB1 \| -0.70 \| 1.72E-33 \| 1.22E-32 \| \| ENSG00000198945 \| L3MBTL3 \| 1.01 \| 1.78E-33 \| 1.27E-32 \| \| ENSG00000148677 \| ANKRD1 \| 3.69 \| 1.80E-33 \| 1.29E-32 \| \| ENSG00000038002 \| AGA \| -1.96 \| 1.90E-33 \| 1.35E-32 \| \| ENSG00000110711 \| AIP \| 0.69 \| 1.90E-33 \| 1.35E-32 \| \| ENSG00000106605 \| BLVRA \| -2.28 \| 1.98E-33 \| 1.41E-32 \| \| ENSG00000165757 \| JCAD \| -1.64 \| 1.98E-33 \| 1.41E-32 \| \| ENSG00000122223 \| CD244 \| -1.57 \| 2.05E-33 \| 1.46E-32 \| \| ENSG00000181938 \| GINS3 \| -1.37 \| 2.07E-33 \| 1.47E-32 \| \| ENSG00000268734 \|  \| 1.49 \| 2.47E-33 \| 1.75E-32 \| \| ENSG00000173218 \| VANGL1 \| -0.87 \| 2.50E-33 \| 1.77E-32 \| \| ENSG00000070808 \| CAMK2A \| 2.09 \| 2.70E-33 \| 1.91E-32 \| \| ENSG00000178623 \| GPR35 \| 0.89 \| 2.74E-33 \| 1.94E-32 \| \| ENSG00000187951 \| LOC100288637 \| -2.72 \| 3.08E-33 \| 2.18E-32 \| \| ENSG00000067208 \| EVI5 \| 0.90 \| 3.12E-33 \| 2.21E-32 \| \| ENSG00000024526 \| DEPDC1 \| -3.06 \| 3.22E-33 \| 2.28E-32 \| \| ENSG00000144747 \| TMF1 \| 0.69 \| 3.23E-33 \| 2.28E-32 \| \| ENSG00000140443 \| IGF1R \| 2.18 \| 3.42E-33 \| 2.41E-32 \| \| ENSG00000184988 \| TMEM106A \| -0.59 \| 3.50E-33 \| 2.47E-32 \| \| ENSG00000181467 \| RAP2B \| -0.69 \| 3.62E-33 \| 2.55E-32 \| \| ENSG00000008300 \| CELSR3 \| 1.49 \| 3.76E-33 \| 2.65E-32 \| \| ENSG00000113558 \| SKP1 \| -0.71 \| 3.76E-33 \| 2.65E-32 \| \| ENSG00000235092 \| ID2-AS1 \| -2.53 \| 3.95E-33 \| 2.78E-32 \| \| ENSG00000169299 \| PGM2 \| -0.59 \| 3.99E-33 \| 2.81E-32 \| \| ENSG00000179954 \| SSC5D \| 0.95 \| 4.46E-33 \| 3.14E-32 \| \| ENSG00000147027 \| TMEM47 \| 2.45 \| 5.66E-33 \| 3.98E-32 \| \| ENSG00000148700 \| ADD3 \| -1.66 \| 5.68E-33 \| 3.99E-32 \| \| ENSG00000087586 \| AURKA \| -1.03 \| 6.41E-33 \| 4.49E-32 \| \| ENSG00000157064 \| NMNAT2 \| 1.48 \| 6.85E-33 \| 4.80E-32 \| \| ENSG00000155876 \| RRAGA \| 0.62 \| 7.28E-33 \| 5.10E-32 \| \| ENSG00000168884 \| TNIP2 \| 0.78 \| 8.34E-33 \| 5.84E-32 \| \| ENSG00000104936 \| DMPK \| 0.93 \| 8.55E-33 \| 5.98E-32 \| \| ENSG00000171451 \| DSEL \| 4.37 \| 8.56E-33 \| 5.98E-32 \| \| ENSG00000101265 \| RASSF2 \| -1.10 \| 8.62E-33 \| 6.02E-32 \| \| ENSG00000136273 \| HUS1 \| 0.77 \| 9.06E-33 \| 6.33E-32 \| \| ENSG00000116809 \| ZBTB17 \| 0.66 \| 9.36E-33 \| 6.53E-32 \| \| ENSG00000092964 \| DPYSL2 \| -0.70 \| 9.53E-33 \| 6.65E-32 \| \| ENSG00000104472 \| CHRAC1 \| 0.64 \| 9.55E-33 \| 6.66E-32 \| \| ENSG00000180543 \| TSPYL5 \| 0.60 \| 1.05E-32 \| 7.34E-32 \| \| ENSG00000197299 \| BLM \| -1.64 \| 1.13E-32 \| 7.90E-32 \| \| ENSG00000172243 \| CLEC7A \| -3.18 \| 1.30E-32 \| 9.02E-32 \| \| ENSG00000120337 \| TNFSF18 \| -2.42 \| 1.30E-32 \| 9.05E-32 \| \| ENSG00000108018 \| SORCS1 \| -1.59 \| 1.39E-32 \| 9.64E-32 \| \| ENSG00000116857 \| TMEM9 \| -1.07 \| 1.41E-32 \| 9.76E-32 \| \| ENSG00000144597 \| EAF1 \| 0.67 \| 1.42E-32 \| 9.84E-32 \| \| ENSG00000107882 \| SUFU \| -0.95 \| 1.45E-32 \| 1.01E-31 \| \| ENSG00000142700 \| DMRTA2 \| -1.00 \| 1.57E-32 \| 1.09E-31 \| \| ENSG00000099958 \| DERL3 \| -2.01 \| 1.64E-32 \| 1.13E-31 \| \| ENSG00000278600 \|  \| 1.75 \| 1.65E-32 \| 1.14E-31 \| \| ENSG00000123500 \| COL10A1 \| 3.49 \| 1.68E-32 \| 1.16E-31 \| \| ENSG00000167363 \| FN3K \| -3.69 \| 1.68E-32 \| 1.16E-31 \| \| ENSG00000237181 \|  \| 0.77 \| 1.73E-32 \| 1.20E-31 \| \| ENSG00000124209 \| RAB22A \| 0.60 \| 1.82E-32 \| 1.26E-31 \| \| ENSG00000114019 \| AMOTL2 \| 1.52 \| 2.14E-32 \| 1.48E-31 \| \| ENSG00000213047 \| DENND1B \| -1.07 \| 2.23E-32 \| 1.54E-31 \| \| ENSG00000146063 \| TRIM41 \| 0.65 \| 2.41E-32 \| 1.66E-31 \| \| ENSG00000110987 \| BCL7A \| -0.96 \| 2.45E-32 \| 1.69E-31 \| \| ENSG00000174749 \| FAM241A \| 1.19 \| 2.75E-32 \| 1.89E-31 \| \| ENSG00000170234 \| PWWP2A \| 0.79 \| 2.78E-32 \| 1.91E-31 \| \| ENSG00000186862 \| PDZD7 \| -2.62 \| 2.91E-32 \| 2.00E-31 \| \| ENSG00000107020 \| PLGRKT \| 0.96 \| 3.02E-32 \| 2.08E-31 \| \| ENSG00000143344 \| RGL1 \| -0.96 \| 3.14E-32 \| 2.16E-31 \| \| ENSG00000265148 \| TSPOAP1-AS1 \| -1.80 \| 3.36E-32 \| 2.31E-31 \| \| ENSG00000248187 \|  \| 1.06 \| 3.47E-32 \| 2.38E-31 \| \| ENSG00000168118 \| RAB4A \| -0.70 \| 3.54E-32 \| 2.43E-31 \| \| ENSG00000125740 \| FOSB \| 2.73 \| 3.68E-32 \| 2.52E-31 \| \| ENSG00000085733 \| CTTN \| 0.82 \| 4.32E-32 \| 2.96E-31 \| \| ENSG00000097046 \| CDC7 \| -1.72 \| 4.75E-32 \| 3.25E-31 \| \| ENSG00000112394 \| SLC16A10 \| 2.42 \| 4.87E-32 \| 3.33E-31 \| \| ENSG00000112078 \| KCTD20 \| -0.61 \| 5.32E-32 \| 3.64E-31 \| \| ENSG00000180914 \| OXTR \| -3.89 \| 5.49E-32 \| 3.75E-31 \| \| ENSG00000100368 \| CSF2RB \| -0.67 \| 5.78E-32 \| 3.95E-31 \| \| ENSG00000134453 \| RBM17 \| -0.77 \| 5.91E-32 \| 4.03E-31 \| \| ENSG00000119596 \| YLPM1 \| 0.61 \| 5.95E-32 \| 4.06E-31 \| \| ENSG00000080822 \| CLDND1 \| 0.86 \| 6.22E-32 \| 4.24E-31 \| \| ENSG00000144668 \| ITGA9 \| -2.76 \| 6.59E-32 \| 4.49E-31 \| \| ENSG00000116171 \| SCP2 \| -0.64 \| 6.88E-32 \| 4.69E-31 \| \| ENSG00000112799 \| LY86 \| -4.33 \| 7.38E-32 \| 5.02E-31 \| \| ENSG00000173638 \| SLC19A1 \| -1.27 \| 7.62E-32 \| 5.18E-31 \| \| ENSG00000006534 \| ALDH3B1 \| 0.59 \| 7.70E-32 \| 5.24E-31 \| \| ENSG00000150782 \| IL18 \| -2.61 \| 7.80E-32 \| 5.30E-31 \| \| ENSG00000122862 \| SRGN \| -0.61 \| 9.01E-32 \| 6.12E-31 \| \| ENSG00000110400 \| NECTIN1 \| -1.26 \| 9.06E-32 \| 6.15E-31 \| \| ENSG00000260314 \| MRC1 \| -5.17 \| 9.13E-32 \| 6.19E-31 \| \| ENSG00000167925 \| GHDC \| -1.02 \| 9.30E-32 \| 6.31E-31 \| \| ENSG00000006282 \| SPATA20 \| -0.61 \| 9.32E-32 \| 6.31E-31 \| \| ENSG00000073464 \| CLCN4 \| -2.96 \| 9.86E-32 \| 6.67E-31 \| \| ENSG00000221829 \| FANCG \| -1.24 \| 1.08E-31 \| 7.30E-31 \| \| ENSG00000012232 \| EXTL3 \| 0.61 \| 1.13E-31 \| 7.61E-31 \| \| ENSG00000275342 \| PRAG1 \| -0.64 \| 1.14E-31 \| 7.68E-31 \| \| ENSG00000249898 \| MCPH1-AS1 \| 1.76 \| 1.21E-31 \| 8.20E-31 \| \| ENSG00000255587 \| RAB44 \| -1.96 \| 1.23E-31 \| 8.33E-31 \| \| ENSG00000182180 \| MRPS16 \| -0.87 \| 1.25E-31 \| 8.45E-31 \| \| ENSG00000147535 \| PLPP5 \| 0.79 \| 1.34E-31 \| 9.03E-31 \| \| ENSG00000237686 \| LOC101929705 \| 3.66 \| 1.37E-31 \| 9.26E-31 \| \| ENSG00000075275 \| CELSR1 \| 0.60 \| 1.45E-31 \| 9.77E-31 \| \| ENSG00000166578 \| IQCD \| 1.76 \| 1.48E-31 \| 9.93E-31 \| \| ENSG00000197712 \| FAM114A1 \| 1.04 \| 1.49E-31 \| 1.00E-30 \| \| ENSG00000183735 \| TBK1 \| 0.67 \| 1.59E-31 \| 1.07E-30 \| \| ENSG00000153162 \| BMP6 \| 1.94 \| 1.63E-31 \| 1.09E-30 \| \| ENSG00000276900 \|  \| 1.18 \| 1.69E-31 \| 1.13E-30 \| \| ENSG00000265354 \| TIMM23 \| -0.81 \| 2.03E-31 \| 1.36E-30 \| \| ENSG00000166928 \| MS4A14 \| -1.51 \| 2.05E-31 \| 1.37E-30 \| \| ENSG00000197070 \| ARRDC1 \| -0.77 \| 2.18E-31 \| 1.46E-30 \| \| ENSG00000154839 \| SKA1 \| -2.38 \| 2.21E-31 \| 1.48E-30 \| \| ENSG00000131668 \| BARX1 \| -1.49 \| 2.23E-31 \| 1.49E-30 \| \| ENSG00000154639 \| CXADR \| -2.04 \| 2.47E-31 \| 1.65E-30 \| \| ENSG00000124107 \| SLPI \| -5.80 \| 2.49E-31 \| 1.67E-30 \| \| ENSG00000010327 \| STAB1 \| -0.90 \| 2.58E-31 \| 1.73E-30 \| \| ENSG00000178252 \| WDR6 \| -0.59 \| 2.64E-31 \| 1.76E-30 \| \| ENSG00000176171 \| BNIP3 \| -1.23 \| 2.90E-31 \| 1.93E-30 \| \| ENSG00000047230 \| CTPS2 \| -1.97 \| 2.93E-31 \| 1.95E-30 \| \| ENSG00000085224 \| ATRX \| 0.63 \| 2.95E-31 \| 1.96E-30 \| \| ENSG00000205726 \| ITSN1 \| -0.67 \| 2.99E-31 \| 1.99E-30 \| \| ENSG00000035687 \| ADSS \| 0.61 \| 3.21E-31 \| 2.13E-30 \| \| ENSG00000198502 \| HLA-DRB5 \| -3.63 \| 3.26E-31 \| 2.17E-30 \| \| ENSG00000015413 \| DPEP1 \| -4.91 \| 3.29E-31 \| 2.19E-30 \| \| ENSG00000140968 \| IRF8 \| -0.92 \| 3.38E-31 \| 2.24E-30 \| \| ENSG00000095951 \| HIVEP1 \| 0.80 \| 3.75E-31 \| 2.49E-30 \| \| ENSG00000163625 \| WDFY3 \| 0.78 \| 3.96E-31 \| 2.62E-30 \| \| ENSG00000198719 \| DLL1 \| 2.36 \| 3.98E-31 \| 2.64E-30 \| \| ENSG00000100626 \| GALNT16 \| -2.98 \| 4.14E-31 \| 2.74E-30 \| \| ENSG00000196968 \| FUT11 \| -1.98 \| 4.66E-31 \| 3.09E-30 \| \| ENSG00000196189 \| SEMA4A \| 0.96 \| 4.73E-31 \| 3.13E-30 \| \| ENSG00000214063 \| TSPAN4 \| -1.71 \| 5.15E-31 \| 3.41E-30 \| \| ENSG00000100448 \| CTSG \| -6.45 \| 5.80E-31 \| 3.84E-30 \| \| ENSG00000076685 \| NT5C2 \| -0.64 \| 6.18E-31 \| 4.08E-30 \| \| ENSG00000020129 \| NCDN \| 0.62 \| 6.41E-31 \| 4.23E-30 \| \| ENSG00000047578 \| KIAA0556 \| 0.72 \| 6.55E-31 \| 4.32E-30 \| \| ENSG00000105778 \| AVL9 \| 0.64 \| 7.74E-31 \| 5.10E-30 \| \| ENSG00000183741 \| CBX6 \| -0.59 \| 7.95E-31 \| 5.23E-30 \| \| ENSG00000154359 \| LONRF1 \| 0.87 \| 7.98E-31 \| 5.25E-30 \| \| ENSG00000078246 \| TULP3 \| 1.12 \| 8.88E-31 \| 5.83E-30 \| \| ENSG00000178038 \| ALS2CL \| -1.82 \| 8.91E-31 \| 5.85E-30 \| \| ENSG00000014914 \| MTMR11 \| -0.83 \| 9.48E-31 \| 6.22E-30 \| \| ENSG00000171130 \| ATP6V0E2 \| -1.41 \| 9.85E-31 \| 6.46E-30 \| \| ENSG00000051180 \| RAD51 \| -1.95 \| 1.16E-30 \| 7.62E-30 \| \| ENSG00000183018 \| SPNS2 \| -1.18 \| 1.22E-30 \| 8.02E-30 \| \| ENSG00000130520 \| LSM4 \| -0.59 \| 1.29E-30 \| 8.43E-30 \| \| ENSG00000035499 \| DEPDC1B \| -2.06 \| 1.33E-30 \| 8.70E-30 \| \| ENSG00000117697 \| NSL1 \| -0.99 \| 1.36E-30 \| 8.91E-30 \| \| ENSG00000132109 \| TRIM21 \| -0.70 \| 1.40E-30 \| 9.13E-30 \| \| ENSG00000137710 \| RDX \| -0.68 \| 1.41E-30 \| 9.21E-30 \| \| ENSG00000147202 \| DIAPH2 \| 0.61 \| 1.50E-30 \| 9.77E-30 \| \| ENSG00000143226 \| FCGR2A \| -1.77 \| 1.63E-30 \| 1.07E-29 \| \| ENSG00000143226 \| FCGR2C \| -1.77 \| 1.63E-30 \| 1.07E-29 \| \| ENSG00000111665 \| CDCA3 \| -2.85 \| 1.64E-30 \| 1.07E-29 \| \| ENSG00000104884 \| ERCC2 \| 0.63 \| 1.69E-30 \| 1.10E-29 \| \| ENSG00000162878 \| PKDCC \| -0.80 \| 1.78E-30 \| 1.16E-29 \| \| ENSG00000125247 \| TMTC4 \| 0.98 \| 1.80E-30 \| 1.17E-29 \| \| ENSG00000004809 \| SLC22A16 \| -1.02 \| 1.85E-30 \| 1.20E-29 \| \| ENSG00000143198 \| MGST3 \| -0.74 \| 1.91E-30 \| 1.24E-29 \| \| ENSG00000274605 \| LOC105370333 \| 1.28 \| 1.92E-30 \| 1.25E-29 \| \| ENSG00000114166 \| KAT2B \| -0.90 \| 2.00E-30 \| 1.30E-29 \| \| ENSG00000148450 \| MSRB2 \| -1.53 \| 2.03E-30 \| 1.32E-29 \| \| ENSG00000107566 \| ERLIN1 \| -1.25 \| 2.09E-30 \| 1.36E-29 \| \| ENSG00000100364 \| KIAA0930 \| -0.61 \| 2.10E-30 \| 1.36E-29 \| \| ENSG00000091651 \| ORC6 \| -1.63 \| 2.18E-30 \| 1.42E-29 \| \| ENSG00000156256 \| USP16 \| 0.67 \| 2.57E-30 \| 1.66E-29 \| \| ENSG00000128512 \| DOCK4 \| 0.75 \| 2.63E-30 \| 1.71E-29 \| \| ENSG00000172331 \| BPGM \| 1.02 \| 2.64E-30 \| 1.71E-29 \| \| ENSG00000249115 \| HAUS5 \| -1.26 \| 2.76E-30 \| 1.79E-29 \| \| ENSG00000122882 \| ECD \| -0.74 \| 2.91E-30 \| 1.89E-29 \| \| ENSG00000196547 \| MAN2A2 \| -0.64 \| 2.92E-30 \| 1.89E-29 \| \| ENSG00000163803 \| PLB1 \| -2.15 \| 2.94E-30 \| 1.91E-29 \| \| ENSG00000205336 \| ADGRG1 \| 1.26 \| 3.00E-30 \| 1.94E-29 \| \| ENSG00000126003 \| PLAGL2 \| 0.80 \| 3.07E-30 \| 1.98E-29 \| \| ENSG00000151461 \| UPF2 \| -0.75 \| 3.31E-30 \| 2.14E-29 \| \| ENSG00000154736 \| ADAMTS5 \| 3.84 \| 3.44E-30 \| 2.22E-29 \| \| ENSG00000105426 \| PTPRS \| 1.28 \| 3.48E-30 \| 2.24E-29 \| \| ENSG00000233038 \| LOC100506585 \| -3.07 \| 3.96E-30 \| 2.55E-29 \| \| ENSG00000232679 \|  \| 4.84 \| 3.96E-30 \| 2.55E-29 \| \| ENSG00000121297 \| TSHZ3 \| 1.80 \| 4.42E-30 \| 2.85E-29 \| \| ENSG00000116701 \| NCF2 \| -0.95 \| 4.59E-30 \| 2.95E-29 \| \| ENSG00000128335 \| APOL2 \| -0.97 \| 4.73E-30 \| 3.04E-29 \| \| ENSG00000104490 \| NCALD \| -2.40 \| 4.89E-30 \| 3.15E-29 \| \| ENSG00000107863 \| ARHGAP21 \| -0.81 \| 5.11E-30 \| 3.29E-29 \| \| ENSG00000165178 \| NCF1C \| 1.31 \| 5.43E-30 \| 3.49E-29 \| \| ENSG00000113161 \| HMGCR \| 0.60 \| 5.80E-30 \| 3.72E-29 \| \| ENSG00000172819 \| RARG \| 0.61 \| 6.08E-30 \| 3.90E-29 \| \| ENSG00000198056 \| PRIM1 \| -1.87 \| 6.63E-30 \| 4.25E-29 \| \| ENSG00000120334 \| CENPL \| -1.45 \| 6.82E-30 \| 4.37E-29 \| \| ENSG00000149150 \| SLC43A1 \| -0.82 \| 7.22E-30 \| 4.63E-29 \| \| ENSG00000013573 \| DDX11 \| -0.78 \| 7.37E-30 \| 4.72E-29 \| \| ENSG00000128309 \| MPST \| -1.04 \| 7.60E-30 \| 4.86E-29 \| \| ENSG00000167670 \| CHAF1A \| -0.79 \| 9.71E-30 \| 6.21E-29 \| \| ENSG00000108010 \| GLRX3 \| -0.70 \| 9.83E-30 \| 6.28E-29 \| \| ENSG00000163661 \| PTX3 \| 1.41 \| 9.95E-30 \| 6.36E-29 \| \| ENSG00000204397 \| CARD16 \| -2.16 \| 1.00E-29 \| 6.42E-29 \| \| ENSG00000111647 \| UHRF1BP1L \| 0.62 \| 1.02E-29 \| 6.51E-29 \| \| ENSG00000013725 \| CD6 \| -1.58 \| 1.12E-29 \| 7.18E-29 \| \| ENSG00000109519 \| GRPEL1 \| 0.63 \| 1.15E-29 \| 7.34E-29 \| \| ENSG00000159788 \| RGS12 \| -1.29 \| 1.16E-29 \| 7.37E-29 \| \| ENSG00000112584 \| FAM120B \| 0.62 \| 1.27E-29 \| 8.08E-29 \| \| ENSG00000107672 \| NSMCE4A \| -1.19 \| 1.33E-29 \| 8.49E-29 \| \| ENSG00000169031 \| COL4A3 \| 3.05 \| 1.37E-29 \| 8.75E-29 \| \| ENSG00000163918 \| RFC4 \| -1.27 \| 1.38E-29 \| 8.78E-29 \| \| ENSG00000072071 \| ADGRL1 \| -1.77 \| 1.46E-29 \| 9.32E-29 \| \| ENSG00000155093 \| PTPRN2 \| -1.29 \| 1.57E-29 \| 1.00E-28 \| \| ENSG00000101974 \| ATP11C \| 0.76 \| 1.80E-29 \| 1.14E-28 \| \| ENSG00000149503 \| INCENP \| -0.90 \| 1.85E-29 \| 1.17E-28 \| \| ENSG00000151117 \| TMEM86A \| -0.76 \| 2.11E-29 \| 1.34E-28 \| \| ENSG00000163874 \| ZC3H12A \| 1.29 \| 2.13E-29 \| 1.35E-28 \| \| ENSG00000099840 \| IZUMO4 \| 1.82 \| 2.14E-29 \| 1.36E-28 \| \| ENSG00000153531 \| ADPRHL1 \| 1.05 \| 2.40E-29 \| 1.52E-28 \| \| ENSG00000150938 \| CRIM1 \| 0.94 \| 2.41E-29 \| 1.53E-28 \| \| ENSG00000198924 \| DCLRE1A \| -1.76 \| 2.75E-29 \| 1.74E-28 \| \| ENSG00000109743 \| BST1 \| -1.37 \| 2.81E-29 \| 1.78E-28 \| \| ENSG00000138778 \| CENPE \| -1.31 \| 3.26E-29 \| 2.06E-28 \| \| ENSG00000131153 \| GINS2 \| -1.71 \| 3.30E-29 \| 2.09E-28 \| \| ENSG00000155307 \| SAMSN1 \| -4.26 \| 3.45E-29 \| 2.18E-28 \| \| ENSG00000155307 \| LOC388813 \| -4.26 \| 3.45E-29 \| 2.18E-28 \| \| ENSG00000049249 \| TNFRSF9 \| -2.02 \| 3.45E-29 \| 2.18E-28 \| \| ENSG00000196155 \| PLEKHG4 \| -1.77 \| 3.92E-29 \| 2.48E-28 \| \| ENSG00000149483 \| TMEM138 \| 0.76 \| 3.97E-29 \| 2.50E-28 \| \| ENSG00000140474 \| ULK3 \| -0.61 \| 4.17E-29 \| 2.63E-28 \| \| ENSG00000009950 \| MLXIPL \| -0.79 \| 4.23E-29 \| 2.67E-28 \| \| ENSG00000154920 \| EME1 \| -1.89 \| 4.26E-29 \| 2.68E-28 \| \| ENSG00000185621 \| LMLN \| 0.95 \| 4.86E-29 \| 3.06E-28 \| \| ENSG00000130477 \| UNC13A \| 1.02 \| 5.50E-29 \| 3.45E-28 \| \| ENSG00000183814 \| LIN9 \| -1.46 \| 6.19E-29 \| 3.88E-28 \| \| ENSG00000059758 \| CDK17 \| 0.66 \| 6.31E-29 \| 3.96E-28 \| \| ENSG00000188070 \| C11orf95 \| -0.87 \| 6.37E-29 \| 3.99E-28 \| \| ENSG00000101596 \| SMCHD1 \| 0.61 \| 6.49E-29 \| 4.06E-28 \| \| ENSG00000266714 \| MYO15B \| -0.61 \| 6.53E-29 \| 4.09E-28 \| \| ENSG00000261269 \|  \| -2.20 \| 6.77E-29 \| 4.24E-28 \| \| ENSG00000198963 \| RORB \| 3.13 \| 7.98E-29 \| 4.99E-28 \| \| ENSG00000187164 \| SHTN1 \| -1.30 \| 8.45E-29 \| 5.28E-28 \| \| ENSG00000276116 \| FUT8-AS1 \| 1.45 \| 8.57E-29 \| 5.35E-28 \| \| ENSG00000023445 \| BIRC3 \| 1.51 \| 9.80E-29 \| 6.11E-28 \| \| ENSG00000157796 \| WDR19 \| 0.99 \| 9.97E-29 \| 6.22E-28 \| \| ENSG00000162390 \| ACOT11 \| -0.88 \| 1.00E-28 \| 6.26E-28 \| \| ENSG00000115687 \| PASK \| -1.68 \| 1.12E-28 \| 6.99E-28 \| \| ENSG00000135144 \| DTX1 \| -3.70 \| 1.12E-28 \| 6.99E-28 \| \| ENSG00000091262 \| ABCC6 \| -1.16 \| 1.13E-28 \| 7.01E-28 \| \| ENSG00000277449 \| CEBPB-AS1 \| 1.88 \| 1.17E-28 \| 7.27E-28 \| \| ENSG00000137135 \| ARHGEF39 \| -2.46 \| 1.21E-28 \| 7.52E-28 \| \| ENSG00000139132 \| FGD4 \| -1.83 \| 1.28E-28 \| 7.92E-28 \| \| ENSG00000180767 \| CHST13 \| -1.57 \| 1.47E-28 \| 9.12E-28 \| \| ENSG00000108055 \| SMC3 \| -0.71 \| 1.65E-28 \| 1.02E-27 \| \| ENSG00000138650 \| PCDH10 \| 2.92 \| 1.87E-28 \| 1.16E-27 \| \| ENSG00000223764 \| LINC02593 \| -3.22 \| 1.97E-28 \| 1.22E-27 \| \| ENSG00000144810 \| COL8A1 \| 3.05 \| 2.05E-28 \| 1.27E-27 \| \| ENSG00000196712 \| NF1 \| 0.66 \| 2.15E-28 \| 1.33E-27 \| \| ENSG00000116761 \| CTH \| 2.45 \| 2.17E-28 \| 1.34E-27 \| \| ENSG00000153933 \| DGKE \| 1.23 \| 2.21E-28 \| 1.37E-27 \| \| ENSG00000124198 \| ARFGEF2 \| 0.61 \| 2.36E-28 \| 1.45E-27 \| \| ENSG00000144843 \| ADPRH \| -1.17 \| 2.46E-28 \| 1.52E-27 \| \| ENSG00000035928 \| RFC1 \| -0.70 \| 2.46E-28 \| 1.52E-27 \| \| ENSG00000174233 \| ADCY6 \| 0.97 \| 2.61E-28 \| 1.61E-27 \| \| ENSG00000127419 \| TMEM175 \| -0.78 \| 2.70E-28 \| 1.66E-27 \| \| ENSG00000133874 \| RNF122 \| -0.85 \| 2.71E-28 \| 1.67E-27 \| \| ENSG00000034152 \| MAP2K3 \| 0.64 \| 2.78E-28 \| 1.71E-27 \| \| ENSG00000164506 \| STXBP5 \| 0.73 \| 2.87E-28 \| 1.77E-27 \| \| ENSG00000064490 \| RFXANK \| -0.68 \| 3.16E-28 \| 1.94E-27 \| \| ENSG00000110675 \| ELMOD1 \| 3.62 \| 3.24E-28 \| 1.99E-27 \| \| ENSG00000196372 \| ASB13 \| -1.21 \| 3.31E-28 \| 2.03E-27 \| \| ENSG00000221869 \| CEBPD \| 0.70 \| 3.48E-28 \| 2.13E-27 \| \| ENSG00000141562 \| NARF \| 0.65 \| 3.61E-28 \| 2.21E-27 \| \| ENSG00000143858 \| SYT2 \| -4.86 \| 3.70E-28 \| 2.27E-27 \| \| ENSG00000115194 \| SLC30A3 \| -1.34 \| 3.79E-28 \| 2.32E-27 \| \| ENSG00000172943 \| PHF8 \| 0.61 \| 3.95E-28 \| 2.42E-27 \| \| ENSG00000086758 \| HUWE1 \| 0.64 \| 4.16E-28 \| 2.54E-27 \| \| ENSG00000131409 \| LRRC4B \| -2.82 \| 4.60E-28 \| 2.81E-27 \| \| ENSG00000124275 \| MTRR \| 0.65 \| 5.34E-28 \| 3.26E-27 \| \| ENSG00000136492 \| BRIP1 \| -2.35 \| 5.35E-28 \| 3.26E-27 \| \| ENSG00000197930 \| ERO1A \| 0.60 \| 5.41E-28 \| 3.30E-27 \| \| ENSG00000169155 \| ZBTB43 \| 0.64 \| 5.56E-28 \| 3.39E-27 \| \| ENSG00000117525 \| F3 \| 1.32 \| 5.82E-28 \| 3.55E-27 \| \| ENSG00000130822 \| PNCK \| -3.98 \| 5.94E-28 \| 3.62E-27 \| \| ENSG00000175745 \| NR2F1 \| 1.07 \| 6.14E-28 \| 3.74E-27 \| \| ENSG00000113504 \| SLC12A7 \| -5.78 \| 6.25E-28 \| 3.80E-27 \| \| ENSG00000117152 \| RGS4 \| -3.66 \| 6.28E-28 \| 3.82E-27 \| \| ENSG00000121210 \| TMEM131L \| -1.07 \| 7.06E-28 \| 4.28E-27 \| \| ENSG00000099282 \| TSPAN15 \| -1.52 \| 7.83E-28 \| 4.75E-27 \| \| ENSG00000109689 \| STIM2 \| 0.67 \| 9.41E-28 \| 5.70E-27 \| \| ENSG00000003147 \| ICA1 \| -1.12 \| 9.84E-28 \| 5.96E-27 \| \| ENSG00000114446 \| IFT57 \| 0.88 \| 1.01E-27 \| 6.13E-27 \| \| ENSG00000167207 \| NOD2 \| 1.49 \| 1.09E-27 \| 6.60E-27 \| \| ENSG00000093167 \| LRRFIP2 \| 0.69 \| 1.09E-27 \| 6.62E-27 \| \| ENSG00000118620 \| ZNF430 \| 0.80 \| 1.15E-27 \| 6.96E-27 \| \| ENSG00000115163 \| CENPA \| -2.85 \| 1.18E-27 \| 7.15E-27 \| \| ENSG00000130766 \| SESN2 \| 0.89 \| 1.20E-27 \| 7.27E-27 \| \| ENSG00000105245 \| NUMBL \| -1.17 \| 1.24E-27 \| 7.49E-27 \| \| ENSG00000178607 \| ERN1 \| 0.74 \| 1.24E-27 \| 7.49E-27 \| \| ENSG00000106780 \| MEGF9 \| 0.92 \| 1.25E-27 \| 7.52E-27 \| \| ENSG00000119969 \| HELLS \| -2.21 \| 1.33E-27 \| 8.00E-27 \| \| ENSG00000172315 \| TP53RK \| 0.80 \| 1.40E-27 \| 8.42E-27 \| \| ENSG00000153922 \| CHD1 \| 0.68 \| 1.43E-27 \| 8.59E-27 \| \| ENSG00000172059 \| KLF11 \| -0.64 \| 1.51E-27 \| 9.07E-27 \| \| ENSG00000075340 \| ADD2 \| -3.18 \| 1.53E-27 \| 9.21E-27 \| \| ENSG00000188242 \| PP7080 \| 1.01 \| 1.54E-27 \| 9.27E-27 \| \| ENSG00000177000 \| MTHFR \| -0.65 \| 1.62E-27 \| 9.76E-27 \| \| ENSG00000166189 \| HPS6 \| -0.76 \| 1.66E-27 \| 9.98E-27 \| \| ENSG00000138678 \| GPAT3 \| -1.50 \| 1.70E-27 \| 1.02E-26 \| \| ENSG00000145362 \| ANK2 \| -2.93 \| 1.84E-27 \| 1.10E-26 \| \| ENSG00000163840 \| DTX3L \| -0.73 \| 1.85E-27 \| 1.11E-26 \| \| ENSG00000137968 \| SLC44A5 \| -1.88 \| 1.91E-27 \| 1.14E-26 \| \| ENSG00000121316 \| PLBD1 \| -2.42 \| 2.07E-27 \| 1.24E-26 \| \| ENSG00000170537 \| TMC7 \| -2.40 \| 2.10E-27 \| 1.26E-26 \| \| ENSG00000225614 \| ZNF469 \| -0.73 \| 2.20E-27 \| 1.31E-26 \| \| ENSG00000253293 \| HOXA10 \| -1.05 \| 2.23E-27 \| 1.33E-26 \| \| ENSG00000040933 \| INPP4A \| -0.76 \| 2.36E-27 \| 1.41E-26 \| \| ENSG00000184221 \| OLIG1 \| 1.58 \| 2.47E-27 \| 1.47E-26 \| \| ENSG00000109062 \| SLC9A3R1 \| -1.06 \| 2.48E-27 \| 1.48E-26 \| \| ENSG00000143499 \| SMYD2 \| -1.42 \| 2.49E-27 \| 1.48E-26 \| \| ENSG00000142961 \| MOB3C \| -1.01 \| 2.56E-27 \| 1.52E-26 \| \| ENSG00000155229 \| MMS19 \| -0.70 \| 2.56E-27 \| 1.52E-26 \| \| ENSG00000279198 \|  \| 1.66 \| 2.87E-27 \| 1.71E-26 \| \| ENSG00000132535 \| DLG4 \| 0.62 \| 2.92E-27 \| 1.74E-26 \| \| ENSG00000106868 \| SUSD1 \| -0.80 \| 2.98E-27 \| 1.77E-26 \| \| ENSG00000156136 \| DCK \| -0.80 \| 3.33E-27 \| 1.98E-26 \| \| ENSG00000120889 \| TNFRSF10B \| 0.64 \| 3.61E-27 \| 2.14E-26 \| \| ENSG00000189159 \| JPT1 \| -0.72 \| 3.90E-27 \| 2.31E-26 \| \| ENSG00000100290 \| BIK \| -2.51 \| 4.06E-27 \| 2.41E-26 \| \| ENSG00000170545 \| SMAGP \| 0.80 \| 4.62E-27 \| 2.73E-26 \| \| ENSG00000122884 \| P4HA1 \| -0.77 \| 5.41E-27 \| 3.20E-26 \| \| ENSG00000088827 \| SIGLEC1 \| -4.76 \| 6.10E-27 \| 3.60E-26 \| \| ENSG00000162654 \| GBP4 \| -4.45 \| 6.56E-27 \| 3.87E-26 \| \| ENSG00000163453 \| IGFBP7 \| -1.02 \| 6.60E-27 \| 3.89E-26 \| \| ENSG00000145779 \| TNFAIP8 \| 0.78 \| 7.00E-27 \| 4.12E-26 \| \| ENSG00000170468 \| RIOX1 \| 0.73 \| 7.74E-27 \| 4.55E-26 \| \| ENSG00000099377 \| HSD3B7 \| -1.14 \| 9.23E-27 \| 5.43E-26 \| \| ENSG00000013375 \| PGM3 \| 0.83 \| 1.02E-26 \| 5.99E-26 \| \| ENSG00000075790 \| BCAP29 \| 0.65 \| 1.06E-26 \| 6.25E-26 \| \| ENSG00000123384 \| LRP1 \| -1.14 \| 1.07E-26 \| 6.26E-26 \| \| ENSG00000080709 \| KCNN2 \| -1.46 \| 1.18E-26 \| 6.90E-26 \| \| ENSG00000101079 \| NDRG3 \| -0.81 \| 1.21E-26 \| 7.09E-26 \| \| ENSG00000051382 \| PIK3CB \| 0.61 \| 1.27E-26 \| 7.44E-26 \| \| ENSG00000166004 \| CEP295 \| -1.07 \| 1.29E-26 \| 7.57E-26 \| \| ENSG00000130758 \| MAP3K10 \| 0.72 \| 1.39E-26 \| 8.14E-26 \| \| ENSG00000131013 \| PPIL4 \| 0.70 \| 1.58E-26 \| 9.24E-26 \| \| ENSG00000119917 \| IFIT3 \| -2.53 \| 1.62E-26 \| 9.47E-26 \| \| ENSG00000140691 \| ARMC5 \| 0.76 \| 1.72E-26 \| 1.01E-25 \| \| ENSG00000136153 \| LMO7 \| 1.03 \| 1.80E-26 \| 1.05E-25 \| \| ENSG00000122707 \| RECK \| -0.82 \| 1.86E-26 \| 1.08E-25 \| \| ENSG00000126215 \| XRCC3 \| -1.09 \| 1.90E-26 \| 1.11E-25 \| \| ENSG00000182782 \| HCAR2 \| 4.10 \| 1.95E-26 \| 1.13E-25 \| \| ENSG00000242193 \| CRYZL2P \| -1.27 \| 1.95E-26 \| 1.13E-25 \| \| ENSG00000135272 \| MDFIC \| 0.61 \| 2.12E-26 \| 1.23E-25 \| \| ENSG00000145431 \| PDGFC \| 0.70 \| 2.18E-26 \| 1.27E-25 \| \| ENSG00000158985 \| CDC42SE2 \| -0.87 \| 2.30E-26 \| 1.34E-25 \| \| ENSG00000272886 \| DCP1A \| 0.61 \| 2.31E-26 \| 1.34E-25 \| \| ENSG00000001561 \| ENPP4 \| 0.77 \| 2.32E-26 \| 1.35E-25 \| \| ENSG00000228536 \|  \| 1.63 \| 2.88E-26 \| 1.67E-25 \| \| ENSG00000139112 \| GABARAPL1 \| 0.81 \| 2.99E-26 \| 1.73E-25 \| \| ENSG00000175602 \| CCDC85B \| -0.91 \| 3.03E-26 \| 1.75E-25 \| \| ENSG00000117020 \| AKT3 \| 0.77 \| 3.03E-26 \| 1.76E-25 \| \| ENSG00000203727 \| SAMD5 \| 3.09 \| 3.05E-26 \| 1.77E-25 \| \| ENSG00000058085 \| LAMC2 \| 2.00 \| 3.07E-26 \| 1.77E-25 \| \| ENSG00000169379 \| ARL13B \| 1.01 \| 3.11E-26 \| 1.80E-25 \| \| ENSG00000117877 \| CD3EAP \| 0.81 \| 3.22E-26 \| 1.86E-25 \| \| ENSG00000137868 \| STRA6 \| 2.18 \| 3.22E-26 \| 1.86E-25 \| \| ENSG00000148331 \| ASB6 \| 0.70 \| 3.44E-26 \| 1.99E-25 \| \| ENSG00000154310 \| TNIK \| 0.78 \| 3.74E-26 \| 2.16E-25 \| \| ENSG00000164649 \| CDCA7L \| -1.07 \| 3.86E-26 \| 2.23E-25 \| \| ENSG00000168701 \| TMEM208 \| 0.79 \| 4.06E-26 \| 2.34E-25 \| \| ENSG00000165029 \| ABCA1 \| 1.61 \| 4.25E-26 \| 2.45E-25 \| \| ENSG00000137393 \| RNF144B \| 3.33 \| 4.41E-26 \| 2.54E-25 \| \| ENSG00000177732 \| SOX12 \| -0.66 \| 4.45E-26 \| 2.56E-25 \| \| ENSG00000128917 \| DLL4 \| 2.22 \| 4.64E-26 \| 2.67E-25 \| \| ENSG00000182463 \| TSHZ2 \| 5.44 \| 4.71E-26 \| 2.71E-25 \| \| ENSG00000135547 \| HEY2 \| -2.16 \| 4.91E-26 \| 2.82E-25 \| \| ENSG00000272734 \|  \| -1.13 \| 4.92E-26 \| 2.83E-25 \| \| ENSG00000196507 \| TCEAL3 \| -1.06 \| 5.24E-26 \| 3.01E-25 \| \| ENSG00000231007 \|  \| -3.00 \| 5.61E-26 \| 3.22E-25 \| \| ENSG00000100162 \| CENPM \| -2.30 \| 5.62E-26 \| 3.22E-25 \| \| ENSG00000233539 \| LOC730338 \| 3.77 \| 6.45E-26 \| 3.69E-25 \| \| ENSG00000102218 \| RP2 \| 0.66 \| 6.68E-26 \| 3.83E-25 \| \| ENSG00000154767 \| XPC \| 0.69 \| 7.42E-26 \| 4.25E-25 \| \| ENSG00000137752 \| CASP1 \| -1.32 \| 7.44E-26 \| 4.26E-25 \| \| ENSG00000105352 \| CEACAM4 \| 2.19 \| 7.80E-26 \| 4.46E-25 \| \| ENSG00000166135 \| HIF1AN \| -0.71 \| 7.94E-26 \| 4.54E-25 \| \| ENSG00000163563 \| MNDA \| -6.32 \| 8.49E-26 \| 4.85E-25 \| \| ENSG00000164620 \| RELL2 \| -0.77 \| 8.50E-26 \| 4.85E-25 \| \| ENSG00000204282 \| TNRC6C-AS1 \| -2.24 \| 8.55E-26 \| 4.88E-25 \| \| ENSG00000157214 \| STEAP2 \| 2.12 \| 8.60E-26 \| 4.90E-25 \| \| ENSG00000179361 \| ARID3B \| 0.79 \| 9.13E-26 \| 5.20E-25 \| \| ENSG00000135476 \| ESPL1 \| -1.18 \| 9.47E-26 \| 5.39E-25 \| \| ENSG00000187522 \| HSPA14 \| -1.02 \| 9.65E-26 \| 5.49E-25 \| \| ENSG00000151208 \| DLG5 \| -0.64 \| 9.67E-26 \| 5.50E-25 \| \| ENSG00000022556 \| NLRP2 \| 1.37 \| 1.04E-25 \| 5.91E-25 \| \| ENSG00000145817 \| YIPF5 \| 0.62 \| 1.18E-25 \| 6.72E-25 \| \| ENSG00000186001 \| LRCH3 \| 0.63 \| 1.31E-25 \| 7.42E-25 \| \| ENSG00000264364 \| DYNLL2 \| 0.60 \| 1.37E-25 \| 7.75E-25 \| \| ENSG00000172840 \| PDP2 \| 1.00 \| 1.37E-25 \| 7.79E-25 \| \| ENSG00000138771 \| SHROOM3 \| 2.25 \| 1.38E-25 \| 7.82E-25 \| \| ENSG00000128487 \| SPECC1 \| -1.27 \| 1.38E-25 \| 7.82E-25 \| \| ENSG00000204580 \| DDR1 \| 1.58 \| 1.53E-25 \| 8.64E-25 \| \| ENSG00000137177 \| KIF13A \| 1.34 \| 1.73E-25 \| 9.79E-25 \| \| ENSG00000182400 \| TRAPPC6B \| 0.63 \| 1.79E-25 \| 1.01E-24 \| \| ENSG00000188677 \| PARVB \| 0.76 \| 1.85E-25 \| 1.05E-24 \| \| ENSG00000154127 \| UBASH3B \| -1.15 \| 1.91E-25 \| 1.08E-24 \| \| ENSG00000108622 \| ICAM2 \| -2.87 \| 1.92E-25 \| 1.08E-24 \| \| ENSG00000197582 \|  \| -0.89 \| 2.04E-25 \| 1.15E-24 \| \| ENSG00000132793 \| LPIN3 \| -0.76 \| 2.09E-25 \| 1.18E-24 \| \| ENSG00000153048 \| CARHSP1 \| -0.67 \| 2.09E-25 \| 1.18E-24 \| \| ENSG00000086475 \| SEPHS1 \| -0.85 \| 2.15E-25 \| 1.21E-24 \| \| ENSG00000152818 \| UTRN \| -0.62 \| 2.16E-25 \| 1.21E-24 \| \| ENSG00000175197 \| DDIT3 \| 0.86 \| 2.18E-25 \| 1.23E-24 \| \| ENSG00000136305 \| CIDEB \| -1.04 \| 2.19E-25 \| 1.23E-24 \| \| ENSG00000118412 \| CASP8AP2 \| -0.85 \| 2.64E-25 \| 1.49E-24 \| \| ENSG00000123395 \| ATG101 \| 0.87 \| 2.85E-25 \| 1.60E-24 \| \| ENSG00000156876 \| SASS6 \| -1.47 \| 3.00E-25 \| 1.69E-24 \| \| ENSG00000100592 \| DAAM1 \| 1.08 \| 3.04E-25 \| 1.71E-24 \| \| ENSG00000106603 \| COA1 \| -0.89 \| 3.07E-25 \| 1.73E-24 \| \| ENSG00000169181 \| GSG1L \| 1.33 \| 3.19E-25 \| 1.79E-24 \| \| ENSG00000171492 \| LRRC8D \| -0.88 \| 3.28E-25 \| 1.84E-24 \| \| ENSG00000125148 \| MT2A \| 0.95 \| 3.32E-25 \| 1.86E-24 \| \| ENSG00000136404 \| TM6SF1 \| -1.79 \| 3.36E-25 \| 1.88E-24 \| \| ENSG00000146373 \| RNF217 \| -0.98 \| 3.83E-25 \| 2.14E-24 \| \| ENSG00000101230 \| ISM1 \| 1.31 \| 4.12E-25 \| 2.31E-24 \| \| ENSG00000197632 \| SERPINB2 \| 3.31 \| 4.22E-25 \| 2.36E-24 \| \| ENSG00000163536 \| SERPINI1 \| -1.46 \| 4.28E-25 \| 2.40E-24 \| \| ENSG00000078177 \| N4BP2 \| 0.75 \| 4.61E-25 \| 2.58E-24 \| \| ENSG00000138642 \| HERC6 \| -1.68 \| 4.65E-25 \| 2.60E-24 \| \| ENSG00000079308 \| TNS1 \| -0.68 \| 4.65E-25 \| 2.60E-24 \| \| ENSG00000196247 \| ZNF107 \| -1.41 \| 5.28E-25 \| 2.95E-24 \| \| ENSG00000104067 \| TJP1 \| 2.24 \| 6.01E-25 \| 3.35E-24 \| \| ENSG00000058866 \| DGKG \| -2.48 \| 6.06E-25 \| 3.38E-24 \| \| ENSG00000186792 \| HYAL3 \| -1.40 \| 6.09E-25 \| 3.39E-24 \| \| ENSG00000129810 \| SGO1 \| -2.34 \| 6.59E-25 \| 3.67E-24 \| \| ENSG00000073737 \| DHRS9 \| -4.44 \| 6.69E-25 \| 3.72E-24 \| \| ENSG00000268043 \| NBPF12 \| 0.79 \| 6.90E-25 \| 3.83E-24 \| \| ENSG00000213390 \| ARHGAP19 \| -1.29 \| 7.19E-25 \| 3.99E-24 \| \| ENSG00000063660 \| GPC1 \| -0.96 \| 7.25E-25 \| 4.02E-24 \| \| ENSG00000182263 \| FIGN \| -3.11 \| 7.71E-25 \| 4.28E-24 \| \| ENSG00000116704 \| SLC35D1 \| 0.69 \| 7.78E-25 \| 4.32E-24 \| \| ENSG00000185090 \| MANEAL \| 0.73 \| 7.85E-25 \| 4.36E-24 \| \| ENSG00000138386 \| NAB1 \| 0.67 \| 7.98E-25 \| 4.42E-24 \| \| ENSG00000038219 \| BOD1L1 \| 0.64 \| 8.58E-25 \| 4.75E-24 \| \| ENSG00000183691 \| NOG \| 1.96 \| 8.86E-25 \| 4.91E-24 \| \| ENSG00000112208 \| BAG2 \| -1.36 \| 8.92E-25 \| 4.94E-24 \| \| ENSG00000178209 \| PLEC \| 0.73 \| 9.67E-25 \| 5.34E-24 \| \| ENSG00000151882 \| CCL28 \| -5.19 \| 9.71E-25 \| 5.36E-24 \| \| ENSG00000187486 \| KCNJ11 \| 2.46 \| 9.78E-25 \| 5.40E-24 \| \| ENSG00000139946 \| PELI2 \| 2.00 \| 9.93E-25 \| 5.48E-24 \| \| ENSG00000276085 \| CCL3L1 \| 2.22 \| 1.04E-24 \| 5.75E-24 \| \| ENSG00000276085 \| CCL3L3 \| 2.22 \| 1.04E-24 \| 5.75E-24 \| \| ENSG00000136824 \| SMC2 \| -0.80 \| 1.12E-24 \| 6.17E-24 \| \| ENSG00000137077 \| CCL21 \| -1.69 \| 1.15E-24 \| 6.37E-24 \| \| ENSG00000282851 \| BISPR \| -2.35 \| 1.19E-24 \| 6.58E-24 \| \| ENSG00000112294 \| ALDH5A1 \| -1.27 \| 1.23E-24 \| 6.76E-24 \| \| ENSG00000049323 \| LTBP1 \| -1.56 \| 1.26E-24 \| 6.92E-24 \| \| ENSG00000111801 \| BTN3A3 \| -2.43 \| 1.29E-24 \| 7.08E-24 \| \| ENSG00000160570 \| DEDD2 \| 0.64 \| 1.31E-24 \| 7.22E-24 \| \| ENSG00000261295 \|  \| 1.86 \| 1.41E-24 \| 7.77E-24 \| \| ENSG00000185567 \| AHNAK2 \| -1.10 \| 1.46E-24 \| 8.04E-24 \| \| ENSG00000101236 \| RNF24 \| 0.67 \| 1.51E-24 \| 8.30E-24 \| \| ENSG00000143401 \| ANP32E \| -0.59 \| 1.58E-24 \| 8.66E-24 \| \| ENSG00000233621 \| LINC01137 \| 1.03 \| 1.59E-24 \| 8.70E-24 \| \| ENSG00000170430 \| MGMT \| -1.42 \| 1.68E-24 \| 9.23E-24 \| \| ENSG00000249001 \|  \| -1.00 \| 1.70E-24 \| 9.33E-24 \| \| ENSG00000132530 \| XAF1 \| -3.64 \| 1.94E-24 \| 1.06E-23 \| \| ENSG00000181192 \| DHTKD1 \| -0.98 \| 2.12E-24 \| 1.16E-23 \| \| ENSG00000197558 \| SSPO \| -2.29 \| 2.16E-24 \| 1.18E-23 \| \| ENSG00000176148 \| TCP11L1 \| 0.85 \| 2.20E-24 \| 1.20E-23 \| \| ENSG00000198691 \| ABCA4 \| 2.58 \| 2.36E-24 \| 1.29E-23 \| \| ENSG00000204178 \| MACO1 \| 0.59 \| 2.57E-24 \| 1.40E-23 \| \| ENSG00000168386 \| FILIP1L \| -0.67 \| 2.63E-24 \| 1.43E-23 \| \| ENSG00000140030 \| GPR65 \| -1.31 \| 2.70E-24 \| 1.47E-23 \| \| ENSG00000107829 \| FBXW4 \| -1.04 \| 2.96E-24 \| 1.61E-23 \| \| ENSG00000105173 \| CCNE1 \| -1.00 \| 3.00E-24 \| 1.63E-23 \| \| ENSG00000152784 \| PRDM8 \| 1.11 \| 3.23E-24 \| 1.76E-23 \| \| ENSG00000187791 \| FAM205C \| -0.81 \| 3.28E-24 \| 1.79E-23 \| \| ENSG00000145041 \| DCAF1 \| 0.59 \| 3.35E-24 \| 1.82E-23 \| \| ENSG00000088387 \| DOCK9 \| 1.94 \| 3.47E-24 \| 1.89E-23 \| \| ENSG00000120008 \| WDR11 \| -0.78 \| 3.69E-24 \| 2.01E-23 \| \| ENSG00000180817 \| PPA1 \| -0.77 \| 4.51E-24 \| 2.45E-23 \| \| ENSG00000107874 \| CUEDC2 \| -0.74 \| 4.77E-24 \| 2.59E-23 \| \| ENSG00000139567 \| ACVRL1 \| -1.33 \| 4.82E-24 \| 2.61E-23 \| \| ENSG00000134970 \| TMED7 \| -0.76 \| 4.86E-24 \| 2.63E-23 \| \| ENSG00000177556 \| ATOX1 \| 0.69 \| 5.08E-24 \| 2.75E-23 \| \| ENSG00000133063 \| CHIT1 \| 1.78 \| 5.28E-24 \| 2.86E-23 \| \| ENSG00000135749 \| PCNX2 \| 0.65 \| 5.51E-24 \| 2.98E-23 \| \| ENSG00000124225 \| PMEPA1 \| 1.14 \| 5.61E-24 \| 3.03E-23 \| \| ENSG00000116711 \| PLA2G4A \| -1.21 \| 5.71E-24 \| 3.08E-23 \| \| ENSG00000121274 \| TENT4B \| 0.77 \| 5.72E-24 \| 3.09E-23 \| \| ENSG00000206337 \| HCP5 \| -3.63 \| 5.83E-24 \| 3.15E-23 \| \| ENSG00000122863 \| CHST3 \| -2.52 \| 5.89E-24 \| 3.18E-23 \| \| ENSG00000142733 \| MAP3K6 \| -0.89 \| 5.89E-24 \| 3.18E-23 \| \| ENSG00000173210 \| ABLIM3 \| -0.65 \| 6.01E-24 \| 3.24E-23 \| \| ENSG00000147130 \| ZMYM3 \| -0.68 \| 6.39E-24 \| 3.45E-23 \| \| ENSG00000163597 \| SNHG16 \| 0.59 \| 6.45E-24 \| 3.47E-23 \| \| ENSG00000101745 \| ANKRD12 \| 0.59 \| 6.95E-24 \| 3.74E-23 \| \| ENSG00000282608 \| ADORA3 \| -2.50 \| 6.99E-24 \| 3.76E-23 \| \| ENSG00000102962 \| CCL22 \| -2.89 \| 7.08E-24 \| 3.81E-23 \| \| ENSG00000007171 \| NOS2 \| 1.51 \| 8.35E-24 \| 4.49E-23 \| \| ENSG00000180953 \| ST20 \| 1.74 \| 9.45E-24 \| 5.08E-23 \| \| ENSG00000206418 \| RAB12 \| 0.64 \| 9.67E-24 \| 5.20E-23 \| \| ENSG00000100376 \| FAM118A \| -1.03 \| 9.83E-24 \| 5.28E-23 \| \| ENSG00000163293 \| NIPAL1 \| 2.39 \| 1.03E-23 \| 5.55E-23 \| \| ENSG00000183569 \| SERHL2 \| -2.36 \| 1.05E-23 \| 5.61E-23 \| \| ENSG00000137871 \| ZNF280D \| -0.60 \| 1.23E-23 \| 6.61E-23 \| \| ENSG00000152082 \| MZT2B \| -0.78 \| 1.24E-23 \| 6.65E-23 \| \| ENSG00000125166 \| GOT2 \| -0.62 \| 1.26E-23 \| 6.76E-23 \| \| ENSG00000077157 \| PPP1R12B \| -1.08 \| 1.26E-23 \| 6.77E-23 \| \| ENSG00000196865 \| NHLRC2 \| -1.08 \| 1.40E-23 \| 7.47E-23 \| \| ENSG00000228477 \|  \| 1.62 \| 1.47E-23 \| 7.88E-23 \| \| ENSG00000170325 \| PRDM10 \| 0.69 \| 1.48E-23 \| 7.93E-23 \| \| ENSG00000109323 \| MANBA \| 0.60 \| 1.49E-23 \| 7.96E-23 \| \| ENSG00000164327 \| RICTOR \| 0.63 \| 1.50E-23 \| 7.98E-23 \| \| ENSG00000198960 \| ARMCX6 \| -0.90 \| 1.52E-23 \| 8.11E-23 \| \| ENSG00000196639 \| HRH1 \| 0.72 \| 1.52E-23 \| 8.12E-23 \| \| ENSG00000173517 \| PEAK1 \| 0.94 \| 1.66E-23 \| 8.83E-23 \| \| ENSG00000143224 \| PPOX \| -1.30 \| 1.66E-23 \| 8.84E-23 \| \| ENSG00000099139 \| PCSK5 \| -1.45 \| 1.78E-23 \| 9.46E-23 \| \| ENSG00000047648 \| ARHGAP6 \| -1.18 \| 1.80E-23 \| 9.57E-23 \| \| ENSG00000118308 \| LRMP \| -0.91 \| 1.80E-23 \| 9.58E-23 \| \| ENSG00000135070 \| ISCA1 \| 0.72 \| 1.98E-23 \| 1.05E-22 \| \| ENSG00000122378 \| PRXL2A \| -0.95 \| 2.08E-23 \| 1.10E-22 \| \| ENSG00000186417 \| GLDN \| -6.03 \| 2.25E-23 \| 1.19E-22 \| \| ENSG00000108387 \| 04-Sep \| -6.55 \| 2.40E-23 \| 1.27E-22 \| \| ENSG00000163507 \| CIP2A \| -1.54 \| 2.52E-23 \| 1.33E-22 \| \| ENSG00000075142 \| SRI \| 0.66 \| 2.82E-23 \| 1.49E-22 \| \| ENSG00000133119 \| RFC3 \| -1.24 \| 2.98E-23 \| 1.57E-22 \| \| ENSG00000081041 \| CXCL2 \| 2.84 \| 3.19E-23 \| 1.68E-22 \| \| ENSG00000163909 \| HEYL \| -2.50 \| 3.95E-23 \| 2.08E-22 \| \| ENSG00000154760 \| SLFN13 \| -3.55 \| 4.32E-23 \| 2.28E-22 \| \| ENSG00000189369 \| GSPT2 \| -3.02 \| 4.72E-23 \| 2.49E-22 \| \| ENSG00000100302 \| RASD2 \| -1.19 \| 5.10E-23 \| 2.68E-22 \| \| ENSG00000165312 \| OTUD1 \| 0.79 \| 5.21E-23 \| 2.74E-22 \| \| ENSG00000139826 \| ABHD13 \| 0.82 \| 5.71E-23 \| 3.00E-22 \| \| ENSG00000126522 \| ASL \| 0.68 \| 6.13E-23 \| 3.22E-22 \| \| ENSG00000108771 \| DHX58 \| -1.24 \| 6.94E-23 \| 3.64E-22 \| \| ENSG00000125746 \| EML2 \| 0.64 \| 7.05E-23 \| 3.70E-22 \| \| ENSG00000005189 \| REXO5 \| -2.57 \| 7.51E-23 \| 3.93E-22 \| \| ENSG00000113621 \| TXNDC15 \| -1.08 \| 7.70E-23 \| 4.03E-22 \| \| ENSG00000169252 \| ADRB2 \| -1.38 \| 8.62E-23 \| 4.51E-22 \| \| ENSG00000110315 \| RNF141 \| -0.76 \| 9.17E-23 \| 4.79E-22 \| \| ENSG00000090686 \| USP48 \| -0.76 \| 9.87E-23 \| 5.15E-22 \| \| ENSG00000143376 \| SNX27 \| 0.63 \| 1.01E-22 \| 5.28E-22 \| \| ENSG00000196792 \| STRN3 \| 0.77 \| 1.04E-22 \| 5.41E-22 \| \| ENSG00000196663 \| TECPR2 \| 0.82 \| 1.04E-22 \| 5.42E-22 \| \| ENSG00000111906 \| HDDC2 \| -0.73 \| 1.07E-22 \| 5.56E-22 \| \| ENSG00000141682 \| PMAIP1 \| -1.43 \| 1.07E-22 \| 5.59E-22 \| \| ENSG00000131711 \| MAP1B \| 1.17 \| 1.08E-22 \| 5.61E-22 \| \| ENSG00000130812 \| ANGPTL6 \| -2.49 \| 1.10E-22 \| 5.72E-22 \| \| ENSG00000100697 \| DICER1 \| 0.64 \| 1.20E-22 \| 6.22E-22 \| \| ENSG00000149212 \| SESN3 \| -1.39 \| 1.22E-22 \| 6.32E-22 \| \| ENSG00000142303 \| ADAMTS10 \| -3.92 \| 1.24E-22 \| 6.43E-22 \| \| ENSG00000179820 \| MYADM \| -0.66 \| 1.39E-22 \| 7.21E-22 \| \| ENSG00000184497 \| TMEM255B \| -1.88 \| 1.41E-22 \| 7.30E-22 \| \| ENSG00000106031 \| HOXA13 \| -2.82 \| 1.42E-22 \| 7.35E-22 \| \| ENSG00000172638 \| EFEMP2 \| -0.94 \| 1.65E-22 \| 8.55E-22 \| \| ENSG00000112029 \| FBXO5 \| -0.92 \| 1.69E-22 \| 8.73E-22 \| \| ENSG00000168502 \| MTCL1 \| 0.98 \| 1.70E-22 \| 8.79E-22 \| \| ENSG00000104064 \| GABPB1 \| 0.60 \| 1.79E-22 \| 9.26E-22 \| \| ENSG00000161267 \| BDH1 \| 1.24 \| 1.89E-22 \| 9.78E-22 \| \| ENSG00000115129 \| TP53I3 \| -1.61 \| 2.00E-22 \| 1.04E-21 \| \| ENSG00000120262 \| CCDC170 \| -1.25 \| 2.15E-22 \| 1.11E-21 \| \| ENSG00000161036 \| LRWD1 \| 0.59 \| 2.43E-22 \| 1.26E-21 \| \| ENSG00000092621 \| PHGDH \| -1.78 \| 2.46E-22 \| 1.27E-21 \| \| ENSG00000188243 \| COMMD6 \| -0.71 \| 2.58E-22 \| 1.33E-21 \| \| ENSG00000105971 \| CAV2 \| 1.33 \| 2.61E-22 \| 1.35E-21 \| \| ENSG00000109674 \| NEIL3 \| -4.11 \| 3.02E-22 \| 1.55E-21 \| \| ENSG00000174672 \| BRSK2 \| 2.17 \| 3.47E-22 \| 1.78E-21 \| \| ENSG00000173894 \| CBX2 \| -2.33 \| 3.54E-22 \| 1.82E-21 \| \| ENSG00000166295 \| ANAPC16 \| -0.73 \| 3.57E-22 \| 1.83E-21 \| \| ENSG00000156966 \| B3GNT7 \| -1.74 \| 3.92E-22 \| 2.01E-21 \| \| ENSG00000134222 \| PSRC1 \| -1.10 \| 3.96E-22 \| 2.03E-21 \| \| ENSG00000236345 \|  \| 1.47 \| 4.36E-22 \| 2.23E-21 \| \| ENSG00000184319 \| RPL23AP82 \| 0.90 \| 4.44E-22 \| 2.27E-21 \| \| ENSG00000119771 \| KLHL29 \| 1.90 \| 4.62E-22 \| 2.36E-21 \| \| ENSG00000105321 \| CCDC9 \| 0.74 \| 4.64E-22 \| 2.37E-21 \| \| ENSG00000168765 \| GSTM4 \| -1.09 \| 4.78E-22 \| 2.44E-21 \| \| ENSG00000186687 \| LYRM7 \| -1.34 \| 4.98E-22 \| 2.55E-21 \| \| ENSG00000140995 \| DEF8 \| -0.66 \| 5.10E-22 \| 2.61E-21 \| \| ENSG00000148082 \| SHC3 \| 1.98 \| 5.16E-22 \| 2.63E-21 \| \| ENSG00000068383 \| INPP5A \| -1.17 \| 5.27E-22 \| 2.69E-21 \| \| ENSG00000154930 \| ACSS1 \| -1.92 \| 6.62E-22 \| 3.37E-21 \| \| ENSG00000178896 \| EXOSC4 \| 0.63 \| 8.33E-22 \| 4.23E-21 \| \| ENSG00000104889 \| RNASEH2A \| -0.94 \| 8.42E-22 \| 4.28E-21 \| \| ENSG00000111271 \| ACAD10 \| -0.79 \| 9.13E-22 \| 4.63E-21 \| \| ENSG00000261888 \|  \| 1.25 \| 9.24E-22 \| 4.68E-21 \| \| ENSG00000144339 \| TMEFF2 \| 2.73 \| 9.80E-22 \| 4.96E-21 \| \| ENSG00000001461 \| NIPAL3 \| -0.85 \| 9.81E-22 \| 4.96E-21 \| \| ENSG00000162645 \| GBP2 \| -1.42 \| 1.01E-21 \| 5.09E-21 \| \| ENSG00000161921 \| CXCL16 \| -0.81 \| 1.16E-21 \| 5.89E-21 \| \| ENSG00000092036 \| HAUS4 \| -1.41 \| 1.25E-21 \| 6.31E-21 \| \| ENSG00000006468 \| ETV1 \| 1.46 \| 1.31E-21 \| 6.60E-21 \| \| ENSG00000166579 \| NDEL1 \| 0.73 \| 1.31E-21 \| 6.63E-21 \| \| ENSG00000128791 \| TWSG1 \| 0.62 \| 1.34E-21 \| 6.74E-21 \| \| ENSG00000235655 \|  \| -0.91 \| 1.44E-21 \| 7.23E-21 \| \| ENSG00000122986 \| HVCN1 \| -0.80 \| 1.44E-21 \| 7.26E-21 \| \| ENSG00000198954 \| KIF1BP \| -1.00 \| 1.49E-21 \| 7.48E-21 \| \| ENSG00000023041 \| ZDHHC6 \| -0.64 \| 1.50E-21 \| 7.57E-21 \| \| ENSG00000145247 \| OCIAD2 \| -0.97 \| 1.51E-21 \| 7.59E-21 \| \| ENSG00000165806 \| CASP7 \| -0.87 \| 1.55E-21 \| 7.81E-21 \| \| ENSG00000168528 \| SERINC2 \| -2.09 \| 1.58E-21 \| 7.93E-21 \| \| ENSG00000213983 \| AP1G2 \| -0.67 \| 1.60E-21 \| 8.02E-21 \| \| ENSG00000071794 \| HLTF \| -1.35 \| 1.65E-21 \| 8.28E-21 \| \| ENSG00000009830 \| POMT2 \| 0.78 \| 1.69E-21 \| 8.46E-21 \| \| ENSG00000271122 \| LOC101930085 \| 0.68 \| 1.69E-21 \| 8.46E-21 \| \| ENSG00000000003 \| TSPAN6 \| 1.59 \| 1.74E-21 \| 8.73E-21 \| \| ENSG00000169783 \| LINGO1 \| -1.64 \| 1.79E-21 \| 8.98E-21 \| \| ENSG00000171914 \| TLN2 \| -1.25 \| 1.81E-21 \| 9.08E-21 \| \| ENSG00000129946 \| SHC2 \| -1.10 \| 1.85E-21 \| 9.24E-21 \| \| ENSG00000176390 \| CRLF3 \| 0.61 \| 1.85E-21 \| 9.24E-21 \| \| ENSG00000072682 \| P4HA2 \| -1.29 \| 1.86E-21 \| 9.30E-21 \| \| ENSG00000057657 \| PRDM1 \| 0.67 \| 1.91E-21 \| 9.53E-21 \| \| ENSG00000138346 \| DNA2 \| -2.19 \| 2.00E-21 \| 1.00E-20 \| \| ENSG00000160293 \| VAV2 \| -0.82 \| 2.01E-21 \| 1.00E-20 \| \| ENSG00000172175 \| MALT1 \| 0.65 \| 2.03E-21 \| 1.01E-20 \| \| ENSG00000238243 \| OR2W3 \| -2.66 \| 2.04E-21 \| 1.02E-20 \| \| ENSG00000076067 \| RBMS2 \| -2.09 \| 2.05E-21 \| 1.02E-20 \| \| ENSG00000130270 \| ATP8B3 \| -1.47 \| 2.18E-21 \| 1.09E-20 \| \| ENSG00000104888 \| SLC17A7 \| 0.67 \| 2.60E-21 \| 1.30E-20 \| \| ENSG00000216775 \| LOC730101 \| -1.40 \| 2.62E-21 \| 1.30E-20 \| \| ENSG00000102100 \| SLC35A2 \| 0.63 \| 2.80E-21 \| 1.39E-20 \| \| ENSG00000129538 \| RNASE1 \| -2.88 \| 2.82E-21 \| 1.40E-20 \| \| ENSG00000132906 \| CASP9 \| 0.87 \| 2.99E-21 \| 1.48E-20 \| \| ENSG00000177839 \| PCDHB9 \| -4.16 \| 2.99E-21 \| 1.49E-20 \| \| ENSG00000164850 \| GPER1 \| -2.49 \| 3.14E-21 \| 1.56E-20 \| \| ENSG00000108306 \| FBXL20 \| 0.76 \| 3.45E-21 \| 1.71E-20 \| \| ENSG00000119431 \| HDHD3 \| -1.90 \| 3.45E-21 \| 1.71E-20 \| \| ENSG00000165948 \| IFI27L1 \| -2.40 \| 3.47E-21 \| 1.72E-20 \| \| ENSG00000198860 \| TSEN15 \| -1.04 \| 3.71E-21 \| 1.84E-20 \| \| ENSG00000116729 \| WLS \| 0.82 \| 3.93E-21 \| 1.94E-20 \| \| ENSG00000097021 \| ACOT7 \| -0.71 \| 4.04E-21 \| 2.00E-20 \| \| ENSG00000055950 \| MRPL43 \| -0.72 \| 4.16E-21 \| 2.06E-20 \| \| ENSG00000082805 \| ERC1 \| 0.74 \| 4.22E-21 \| 2.08E-20 \| \| ENSG00000158457 \| TSPAN33 \| -1.45 \| 4.36E-21 \| 2.15E-20 \| \| ENSG00000187098 \| MITF \| -0.66 \| 4.39E-21 \| 2.17E-20 \| \| ENSG00000244165 \| P2RY11 \| -1.18 \| 4.65E-21 \| 2.29E-20 \| \| ENSG00000101695 \| RNF125 \| -1.27 \| 4.66E-21 \| 2.30E-20 \| \| ENSG00000168404 \| MLKL \| -1.59 \| 4.73E-21 \| 2.33E-20 \| \| ENSG00000171227 \| TMEM37 \| -3.96 \| 5.12E-21 \| 2.52E-20 \| \| ENSG00000146416 \| AIG1 \| -1.06 \| 5.24E-21 \| 2.58E-20 \| \| ENSG00000280303 \|  \| 0.89 \| 5.29E-21 \| 2.61E-20 \| \| ENSG00000183307 \| TMEM121B \| -1.08 \| 5.34E-21 \| 2.63E-20 \| \| ENSG00000175470 \| PPP2R2D \| -0.76 \| 5.36E-21 \| 2.64E-20 \| \| ENSG00000148688 \| RPP30 \| -0.99 \| 5.38E-21 \| 2.65E-20 \| \| ENSG00000153094 \| BCL2L11 \| -0.68 \| 5.86E-21 \| 2.88E-20 \| \| ENSG00000140563 \| MCTP2 \| 3.50 \| 5.87E-21 \| 2.88E-20 \| \| ENSG00000226979 \| LTA \| 2.80 \| 6.28E-21 \| 3.08E-20 \| \| ENSG00000181704 \| YIPF6 \| 0.60 \| 6.81E-21 \| 3.34E-20 \| \| ENSG00000186470 \| BTN3A2 \| -0.96 \| 7.22E-21 \| 3.54E-20 \| \| ENSG00000188419 \| CHM \| 0.68 \| 7.50E-21 \| 3.68E-20 \| \| ENSG00000138795 \| LEF1 \| -1.46 \| 7.73E-21 \| 3.78E-20 \| \| ENSG00000253557 \| LOC100128993 \| -2.27 \| 7.90E-21 \| 3.87E-20 \| \| ENSG00000167969 \| ECI1 \| -1.03 \| 8.80E-21 \| 4.30E-20 \| \| ENSG00000117625 \| RCOR3 \| -0.71 \| 9.37E-21 \| 4.58E-20 \| \| ENSG00000180902 \| D2HGDH \| -1.42 \| 9.61E-21 \| 4.69E-20 \| \| ENSG00000091136 \| LAMB1 \| 2.66 \| 9.86E-21 \| 4.81E-20 \| \| ENSG00000111711 \| GOLT1B \| 0.63 \| 1.04E-20 \| 5.09E-20 \| \| ENSG00000248323 \| LUCAT1 \| 1.01 \| 1.23E-20 \| 5.99E-20 \| \| ENSG00000149218 \| ENDOD1 \| -0.83 \| 1.27E-20 \| 6.19E-20 \| \| ENSG00000254703 \| SENCR \| -1.64 \| 1.28E-20 \| 6.24E-20 \| \| ENSG00000163491 \| NEK10 \| 0.96 \| 1.32E-20 \| 6.39E-20 \| \| ENSG00000276043 \| UHRF1 \| -0.74 \| 1.33E-20 \| 6.47E-20 \| \| ENSG00000164867 \| NOS3 \| -2.37 \| 1.35E-20 \| 6.54E-20 \| \| ENSG00000184349 \| EFNA5 \| 2.99 \| 1.40E-20 \| 6.79E-20 \| \| ENSG00000159873 \| CCDC117 \| 0.79 \| 1.57E-20 \| 7.58E-20 \| \| ENSG00000214021 \| TTLL3 \| -0.76 \| 1.60E-20 \| 7.76E-20 \| \| ENSG00000143493 \| INTS7 \| -0.91 \| 1.70E-20 \| 8.22E-20 \| \| ENSG00000139163 \| ETNK1 \| 0.66 \| 1.74E-20 \| 8.39E-20 \| \| ENSG00000278727 \|  \| 1.21 \| 1.76E-20 \| 8.51E-20 \| \| ENSG00000143195 \| ILDR2 \| -2.43 \| 1.78E-20 \| 8.62E-20 \| \| ENSG00000011347 \| SYT7 \| -1.99 \| 1.81E-20 \| 8.75E-20 \| \| ENSG00000014919 \| COX15 \| -1.01 \| 1.84E-20 \| 8.89E-20 \| \| ENSG00000125356 \| NDUFA1 \| -0.60 \| 1.88E-20 \| 9.05E-20 \| \| ENSG00000184916 \| JAG2 \| -0.78 \| 1.88E-20 \| 9.07E-20 \| \| ENSG00000166974 \| MAPRE2 \| -0.67 \| 1.91E-20 \| 9.20E-20 \| \| ENSG00000159239 \|  \| 1.26 \| 1.93E-20 \| 9.31E-20 \| \| ENSG00000131871 \| SELENOS \| 0.59 \| 1.95E-20 \| 9.42E-20 \| \| ENSG00000152944 \| MED21 \| 0.75 \| 2.02E-20 \| 9.75E-20 \| \| ENSG00000280721 \|  \| 1.19 \| 2.14E-20 \| 1.03E-19 \| \| ENSG00000104921 \| FCER2 \| -4.20 \| 2.26E-20 \| 1.09E-19 \| \| ENSG00000198556 \| ZNF789 \| -0.98 \| 2.27E-20 \| 1.09E-19 \| \| ENSG00000240809 \|  \| -0.76 \| 2.32E-20 \| 1.11E-19 \| \| ENSG00000101868 \| POLA1 \| -1.00 \| 2.37E-20 \| 1.14E-19 \| \| ENSG00000137628 \| DDX60 \| -1.39 \| 2.43E-20 \| 1.17E-19 \| \| ENSG00000055732 \| MCOLN3 \| -3.94 \| 2.52E-20 \| 1.21E-19 \| \| ENSG00000106993 \| CDC37L1 \| 0.77 \| 2.53E-20 \| 1.21E-19 \| \| ENSG00000145284 \| SCD5 \| 1.51 \| 2.57E-20 \| 1.23E-19 \| \| ENSG00000089195 \| TRMT6 \| 0.94 \| 2.60E-20 \| 1.25E-19 \| \| ENSG00000166803 \| PCLAF \| -2.54 \| 3.03E-20 \| 1.45E-19 \| \| ENSG00000103241 \| FOXF1 \| 1.57 \| 3.08E-20 \| 1.48E-19 \| \| ENSG00000078043 \| PIAS2 \| -0.77 \| 3.23E-20 \| 1.55E-19 \| \| ENSG00000138190 \| EXOC6 \| -1.34 \| 3.29E-20 \| 1.58E-19 \| \| ENSG00000196782 \| MAML3 \| -0.68 \| 3.30E-20 \| 1.58E-19 \| \| ENSG00000157240 \| FZD1 \| 0.62 \| 3.35E-20 \| 1.60E-19 \| \| ENSG00000148655 \| LRMDA \| -3.61 \| 3.41E-20 \| 1.63E-19 \| \| ENSG00000120539 \| MASTL \| -0.86 \| 3.61E-20 \| 1.72E-19 \| \| ENSG00000163736 \| PPBP \| -2.34 \| 3.79E-20 \| 1.81E-19 \| \| ENSG00000134982 \| APC \| -0.71 \| 3.85E-20 \| 1.84E-19 \| \| ENSG00000133739 \| LRRCC1 \| -1.39 \| 3.96E-20 \| 1.89E-19 \| \| ENSG00000118965 \| WDR35 \| 1.10 \| 4.01E-20 \| 1.91E-19 \| \| ENSG00000119333 \| WDR34 \| -0.70 \| 4.07E-20 \| 1.94E-19 \| \| ENSG00000089006 \| SNX5 \| -0.59 \| 4.49E-20 \| 2.14E-19 \| \| ENSG00000057019 \| DCBLD2 \| 0.71 \| 4.62E-20 \| 2.20E-19 \| \| ENSG00000131368 \| MRPS25 \| -0.66 \| 4.70E-20 \| 2.24E-19 \| \| ENSG00000078098 \| FAP \| 1.05 \| 4.77E-20 \| 2.27E-19 \| \| ENSG00000109854 \| HTATIP2 \| -0.76 \| 4.81E-20 \| 2.29E-19 \| \| ENSG00000120658 \| ENOX1 \| 1.01 \| 4.97E-20 \| 2.36E-19 \| \| ENSG00000158352 \| SHROOM4 \| 1.75 \| 5.19E-20 \| 2.46E-19 \| \| ENSG00000164124 \| TMEM144 \| -1.91 \| 5.30E-20 \| 2.51E-19 \| \| ENSG00000227825 \| SLC9A7P1 \| 1.04 \| 5.37E-20 \| 2.55E-19 \| \| ENSG00000147536 \| GINS4 \| -0.97 \| 5.49E-20 \| 2.60E-19 \| \| ENSG00000134452 \| FBH1 \| -0.65 \| 5.99E-20 \| 2.84E-19 \| \| ENSG00000112237 \| CCNC \| -0.72 \| 6.05E-20 \| 2.87E-19 \| \| ENSG00000132359 \| RAP1GAP2 \| -1.13 \| 6.55E-20 \| 3.10E-19 \| \| ENSG00000177721 \| ANXA2R \| -3.21 \| 6.58E-20 \| 3.11E-19 \| \| ENSG00000110660 \| SLC35F2 \| 0.81 \| 7.01E-20 \| 3.31E-19 \| \| ENSG00000259803 \| SLC22A31 \| -1.06 \| 7.54E-20 \| 3.56E-19 \| \| ENSG00000169683 \| LRRC45 \| -1.08 \| 8.06E-20 \| 3.80E-19 \| \| ENSG00000227467 \| LINC01537 \| -5.61 \| 8.21E-20 \| 3.87E-19 \| \| ENSG00000100060 \| MFNG \| 0.71 \| 8.41E-20 \| 3.96E-19 \| \| ENSG00000158747 \| NBL1 \| 0.92 \| 9.77E-20 \| 4.60E-19 \| \| ENSG00000158747 \| MINOS1-NBL1 \| 0.92 \| 9.77E-20 \| 4.60E-19 \| \| ENSG00000137070 \| IL11RA \| -1.33 \| 9.80E-20 \| 4.61E-19 \| \| ENSG00000004468 \| CD38 \| 1.79 \| 1.01E-19 \| 4.76E-19 \| \| ENSG00000076248 \| UNG \| -0.71 \| 1.03E-19 \| 4.84E-19 \| \| ENSG00000159733 \| ZFYVE28 \| 0.97 \| 1.05E-19 \| 4.95E-19 \| \| ENSG00000164619 \| BMPER \| 3.70 \| 1.09E-19 \| 5.12E-19 \| \| ENSG00000150995 \| ITPR1 \| 0.78 \| 1.09E-19 \| 5.13E-19 \| \| ENSG00000226453 \|  \| -5.55 \| 1.10E-19 \| 5.16E-19 \| \| ENSG00000176142 \| TMEM39A \| 0.73 \| 1.10E-19 \| 5.18E-19 \| \| ENSG00000165240 \| ATP7A \| 0.73 \| 1.11E-19 \| 5.21E-19 \| \| ENSG00000261795 \|  \| -2.24 \| 1.11E-19 \| 5.23E-19 \| \| ENSG00000128923 \| MINDY2 \| 0.59 \| 1.14E-19 \| 5.32E-19 \| \| ENSG00000196295 \| GARS-DT \| -0.66 \| 1.15E-19 \| 5.40E-19 \| \| ENSG00000137413 \| TAF8 \| 0.66 \| 1.20E-19 \| 5.59E-19 \| \| ENSG00000128951 \| DUT \| -0.86 \| 1.24E-19 \| 5.78E-19 \| \| ENSG00000213722 \| DDAH2 \| -1.00 \| 1.33E-19 \| 6.20E-19 \| \| ENSG00000141582 \| CBX4 \| -0.85 \| 1.37E-19 \| 6.41E-19 \| \| ENSG00000166169 \| POLL \| -1.03 \| 1.42E-19 \| 6.61E-19 \| \| ENSG00000188610 \| FAM72B \| -1.70 \| 1.46E-19 \| 6.83E-19 \| \| ENSG00000089558 \| KCNH4 \| -0.96 \| 1.48E-19 \| 6.89E-19 \| \| ENSG00000134030 \| CTIF \| 0.73 \| 1.55E-19 \| 7.22E-19 \| \| ENSG00000162739 \| SLAMF6 \| -4.86 \| 1.81E-19 \| 8.41E-19 \| \| ENSG00000175029 \| CTBP2 \| -0.85 \| 1.93E-19 \| 8.99E-19 \| \| ENSG00000211584 \| SLC48A1 \| -1.06 \| 2.02E-19 \| 9.38E-19 \| \| ENSG00000176490 \| DIRAS1 \| -0.73 \| 2.08E-19 \| 9.66E-19 \| \| ENSG00000167208 \| SNX20 \| -0.78 \| 2.38E-19 \| 1.10E-18 \| \| ENSG00000065600 \| TMEM206 \| -0.65 \| 2.41E-19 \| 1.12E-18 \| \| ENSG00000234311 \|  \| -2.98 \| 2.41E-19 \| 1.12E-18 \| \| ENSG00000106608 \| URGCP \| 0.67 \| 2.61E-19 \| 1.21E-18 \| \| ENSG00000158402 \| CDC25C \| -2.73 \| 2.64E-19 \| 1.22E-18 \| \| ENSG00000165521 \| EML5 \| 2.07 \| 2.69E-19 \| 1.24E-18 \| \| ENSG00000134138 \| MEIS2 \| 0.66 \| 2.70E-19 \| 1.25E-18 \| \| ENSG00000121957 \| GPSM2 \| -1.28 \| 2.84E-19 \| 1.31E-18 \| \| ENSG00000184574 \| LPAR5 \| -0.97 \| 2.86E-19 \| 1.32E-18 \| \| ENSG00000105499 \| PLA2G4C \| -0.62 \| 3.00E-19 \| 1.38E-18 \| \| ENSG00000121552 \| CSTA \| -1.26 \| 3.02E-19 \| 1.39E-18 \| \| ENSG00000154016 \| GRAP \| -2.21 \| 3.35E-19 \| 1.54E-18 \| \| ENSG00000173890 \| GPR160 \| -1.67 \| 3.43E-19 \| 1.58E-18 \| \| ENSG00000088808 \| PPP1R13B \| -0.81 \| 3.56E-19 \| 1.64E-18 \| \| ENSG00000122224 \| LY9 \| -3.00 \| 3.67E-19 \| 1.69E-18 \| \| ENSG00000116663 \| FBXO6 \| -1.52 \| 3.98E-19 \| 1.83E-18 \| \| ENSG00000108960 \| MMD \| 0.75 \| 4.03E-19 \| 1.85E-18 \| \| ENSG00000269713 \| NBPF9 \| 1.17 \| 4.15E-19 \| 1.91E-18 \| \| ENSG00000162522 \| KIAA1522 \| -0.68 \| 4.40E-19 \| 2.02E-18 \| \| ENSG00000140545 \| MFGE8 \| -0.64 \| 4.46E-19 \| 2.05E-18 \| \| ENSG00000254858 \| MPV17L2 \| 0.61 \| 4.79E-19 \| 2.20E-18 \| \| ENSG00000114784 \| EIF1B \| 0.65 \| 5.02E-19 \| 2.30E-18 \| \| ENSG00000188643 \| S100A16 \| -0.62 \| 5.17E-19 \| 2.37E-18 \| \| ENSG00000025434 \| NR1H3 \| -0.95 \| 5.26E-19 \| 2.41E-18 \| \| ENSG00000175155 \| YPEL2 \| 0.67 \| 5.69E-19 \| 2.60E-18 \| \| ENSG00000109158 \| GABRA4 \| 1.36 \| 6.08E-19 \| 2.78E-18 \| \| ENSG00000167107 \| ACSF2 \| -2.15 \| 6.27E-19 \| 2.87E-18 \| \| ENSG00000176046 \| NUPR1 \| -2.68 \| 7.19E-19 \| 3.28E-18 \| \| ENSG00000123411 \| IKZF4 \| 0.73 \| 7.44E-19 \| 3.40E-18 \| \| ENSG00000004864 \| SLC25A13 \| 0.76 \| 7.93E-19 \| 3.62E-18 \| \| ENSG00000255921 \|  \| 1.52 \| 8.01E-19 \| 3.66E-18 \| \| ENSG00000171311 \| EXOSC1 \| -1.05 \| 8.08E-19 \| 3.69E-18 \| \| ENSG00000186063 \| AIDA \| -0.62 \| 8.62E-19 \| 3.93E-18 \| \| ENSG00000129951 \| PLPPR3 \| -5.44 \| 8.68E-19 \| 3.95E-18 \| \| ENSG00000154889 \| MPPE1 \| -0.93 \| 8.92E-19 \| 4.06E-18 \| \| ENSG00000174130 \| TLR6 \| -0.61 \| 9.41E-19 \| 4.28E-18 \| \| ENSG00000184207 \| PGP \| -0.65 \| 1.05E-18 \| 4.77E-18 \| \| ENSG00000171860 \| C3AR1 \| -0.85 \| 1.06E-18 \| 4.81E-18 \| \| ENSG00000101447 \| FAM83D \| -2.41 \| 1.10E-18 \| 4.99E-18 \| \| ENSG00000100558 \| PLEK2 \| -3.94 \| 1.12E-18 \| 5.08E-18 \| \| ENSG00000130193 \| THEM6 \| -1.56 \| 1.14E-18 \| 5.18E-18 \| \| ENSG00000165655 \| ZNF503 \| -1.86 \| 1.16E-18 \| 5.24E-18 \| \| ENSG00000163395 \| IGFN1 \| 1.28 \| 1.34E-18 \| 6.05E-18 \| \| ENSG00000165355 \| FBXO33 \| 0.65 \| 1.34E-18 \| 6.05E-18 \| \| ENSG00000169330 \| MINAR1 \| 1.79 \| 1.43E-18 \| 6.48E-18 \| \| ENSG00000137501 \| SYTL2 \| -2.69 \| 1.44E-18 \| 6.50E-18 \| \| ENSG00000008056 \| SYN1 \| 2.10 \| 1.47E-18 \| 6.64E-18 \| \| ENSG00000146540 \| C7orf50 \| -0.61 \| 1.52E-18 \| 6.89E-18 \| \| ENSG00000187950 \| OVCH1 \| 1.34 \| 1.54E-18 \| 6.97E-18 \| \| ENSG00000152620 \| NADK2 \| 0.75 \| 1.60E-18 \| 7.22E-18 \| \| ENSG00000196678 \| ERI2 \| -0.91 \| 1.63E-18 \| 7.35E-18 \| \| ENSG00000128394 \| APOBEC3F \| -2.73 \| 1.66E-18 \| 7.47E-18 \| \| ENSG00000148690 \| FRA10AC1 \| -1.64 \| 1.75E-18 \| 7.91E-18 \| \| ENSG00000136152 \| COG3 \| 0.59 \| 1.86E-18 \| 8.38E-18 \| \| ENSG00000125144 \| MT1G \| 3.68 \| 1.87E-18 \| 8.44E-18 \| \| ENSG00000132823 \| OSER1 \| 0.62 \| 1.96E-18 \| 8.83E-18 \| \| ENSG00000135604 \| STX11 \| 0.83 \| 2.02E-18 \| 9.10E-18 \| \| ENSG00000174370 \| C11orf45 \| -0.80 \| 2.11E-18 \| 9.50E-18 \| \| ENSG00000145423 \| SFRP2 \| 3.64 \| 2.13E-18 \| 9.59E-18 \| \| ENSG00000182676 \| PPP1R27 \| -3.49 \| 2.22E-18 \| 9.96E-18 \| \| ENSG00000158825 \| CDA \| 1.69 \| 2.28E-18 \| 1.02E-17 \| \| ENSG00000142875 \| PRKACB \| -1.00 \| 2.28E-18 \| 1.02E-17 \| \| ENSG00000172349 \| IL16 \| -1.08 \| 2.43E-18 \| 1.09E-17 \| \| ENSG00000265798 \|  \| 1.66 \| 2.60E-18 \| 1.16E-17 \| \| ENSG00000104611 \| SH2D4A \| -1.18 \| 2.76E-18 \| 1.23E-17 \| \| ENSG00000168621 \| GDNF \| 4.28 \| 2.85E-18 \| 1.28E-17 \| \| ENSG00000101916 \| TLR8 \| -2.03 \| 3.08E-18 \| 1.38E-17 \| \| ENSG00000115425 \| PECR \| -2.04 \| 3.19E-18 \| 1.42E-17 \| \| ENSG00000068079 \| IFI35 \| -0.84 \| 3.20E-18 \| 1.43E-17 \| \| ENSG00000180549 \| FUT7 \| -1.31 \| 3.39E-18 \| 1.51E-17 \| \| ENSG00000105497 \| ZNF175 \| 0.88 \| 3.52E-18 \| 1.57E-17 \| \| ENSG00000150510 \| FAM124A \| 1.13 \| 3.52E-18 \| 1.57E-17 \| \| ENSG00000164048 \| ZNF589 \| -1.24 \| 3.65E-18 \| 1.63E-17 \| \| ENSG00000068354 \| TBC1D25 \| 0.59 \| 4.01E-18 \| 1.79E-17 \| \| ENSG00000157404 \| KIT \| -2.81 \| 4.03E-18 \| 1.79E-17 \| \| ENSG00000164099 \| PRSS12 \| -1.29 \| 4.09E-18 \| 1.82E-17 \| \| ENSG00000133574 \| GIMAP4 \| -2.79 \| 4.40E-18 \| 1.95E-17 \| \| ENSG00000065833 \| ME1 \| -0.66 \| 4.83E-18 \| 2.14E-17 \| \| ENSG00000008405 \| CRY1 \| 1.12 \| 4.90E-18 \| 2.18E-17 \| \| ENSG00000139410 \| SDSL \| -1.64 \| 5.33E-18 \| 2.36E-17 \| \| ENSG00000067177 \| PHKA1 \| -2.49 \| 5.34E-18 \| 2.36E-17 \| \| ENSG00000168811 \| IL12A \| -4.72 \| 5.68E-18 \| 2.52E-17 \| \| ENSG00000176438 \| SYNE3 \| -0.72 \| 5.89E-18 \| 2.61E-17 \| \| ENSG00000157617 \| C2CD2 \| -0.85 \| 5.90E-18 \| 2.61E-17 \| \| ENSG00000146410 \| MTFR2 \| -1.65 \| 5.94E-18 \| 2.63E-17 \| \| ENSG00000176720 \| BOK \| -2.34 \| 6.28E-18 \| 2.77E-17 \| \| ENSG00000185379 \| RAD51D \| -1.63 \| 6.63E-18 \| 2.92E-17 \| \| ENSG00000231528 \| FAM225A \| 1.35 \| 6.89E-18 \| 3.04E-17 \| \| ENSG00000118596 \| SLC16A7 \| 0.75 \| 7.26E-18 \| 3.20E-17 \| \| ENSG00000163288 \| GABRB1 \| 4.41 \| 7.35E-18 \| 3.24E-17 \| \| ENSG00000231711 \| LINC00899 \| -3.24 \| 7.43E-18 \| 3.27E-17 \| \| ENSG00000165300 \| SLITRK5 \| 0.63 \| 7.68E-18 \| 3.38E-17 \| \| ENSG00000087076 \| HSD17B14 \| -2.13 \| 7.90E-18 \| 3.48E-17 \| \| ENSG00000175182 \| FAM131A \| 0.97 \| 8.14E-18 \| 3.58E-17 \| \| ENSG00000104047 \| DTWD1 \| 0.90 \| 8.31E-18 \| 3.65E-17 \| \| ENSG00000123975 \| CKS2 \| -1.15 \| 8.46E-18 \| 3.72E-17 \| \| ENSG00000213190 \| MLLT11 \| 1.07 \| 8.94E-18 \| 3.93E-17 \| \| ENSG00000169255 \| B3GALNT1 \| -1.15 \| 9.88E-18 \| 4.33E-17 \| \| ENSG00000117602 \| RCAN3 \| -0.92 \| 9.91E-18 \| 4.35E-17 \| \| ENSG00000188807 \| TMEM201 \| -0.72 \| 9.96E-18 \| 4.36E-17 \| \| ENSG00000070759 \| TESK2 \| 0.94 \| 1.01E-17 \| 4.43E-17 \| \| ENSG00000173200 \| PARP15 \| -2.26 \| 1.02E-17 \| 4.45E-17 \| \| ENSG00000170745 \| KCNS3 \| 1.94 \| 1.13E-17 \| 4.95E-17 \| \| ENSG00000280798 \| LINC00294 \| 1.06 \| 1.35E-17 \| 5.89E-17 \| \| ENSG00000133104 \| SPART \| -0.62 \| 1.37E-17 \| 5.98E-17 \| \| ENSG00000099337 \| KCNK6 \| -0.91 \| 1.47E-17 \| 6.41E-17 \| \| ENSG00000178177 \| LCORL \| 0.73 \| 1.47E-17 \| 6.42E-17 \| \| ENSG00000164949 \| GEM \| 1.24 \| 1.50E-17 \| 6.56E-17 \| \| ENSG00000133121 \| STARD13 \| -0.71 \| 1.51E-17 \| 6.60E-17 \| \| ENSG00000157693 \| TMEM268 \| -0.76 \| 1.58E-17 \| 6.88E-17 \| \| ENSG00000258168 \| LOC105369827 \| 0.60 \| 1.60E-17 \| 6.98E-17 \| \| ENSG00000106299 \| WASL \| 0.65 \| 1.67E-17 \| 7.26E-17 \| \| ENSG00000148459 \| PDSS1 \| -1.36 \| 1.70E-17 \| 7.42E-17 \| \| ENSG00000186810 \| CXCR3 \| -2.47 \| 1.71E-17 \| 7.43E-17 \| \| ENSG00000230024 \|  \| 2.29 \| 1.75E-17 \| 7.62E-17 \| \| ENSG00000247982 \| LINC00926 \| 0.89 \| 1.91E-17 \| 8.27E-17 \| \| ENSG00000116670 \| MAD2L2 \| 0.61 \| 1.97E-17 \| 8.54E-17 \| \| ENSG00000198771 \| RCSD1 \| -1.03 \| 2.04E-17 \| 8.85E-17 \| \| ENSG00000275131 \| LOC100996724 \| 2.34 \| 2.04E-17 \| 8.86E-17 \| \| ENSG00000238164 \| TNFRSF14-AS1 \| -2.12 \| 2.06E-17 \| 8.91E-17 \| \| ENSG00000188811 \| NHLRC3 \| -1.31 \| 2.12E-17 \| 9.20E-17 \| \| ENSG00000138138 \| ATAD1 \| -0.65 \| 2.15E-17 \| 9.29E-17 \| \| ENSG00000011566 \| MAP4K3 \| 0.75 \| 2.20E-17 \| 9.50E-17 \| \| ENSG00000157764 \| BRAF \| 0.61 \| 2.38E-17 \| 1.03E-16 \| \| ENSG00000080823 \| MOK \| 1.49 \| 2.78E-17 \| 1.20E-16 \| \| ENSG00000186998 \| EMID1 \| 2.32 \| 2.87E-17 \| 1.24E-16 \| \| ENSG00000158769 \| F11R \| -0.94 \| 3.14E-17 \| 1.35E-16 \| \| ENSG00000231074 \| HCG18 \| 1.01 \| 3.21E-17 \| 1.38E-16 \| \| ENSG00000176208 \| ATAD5 \| -1.44 \| 3.44E-17 \| 1.48E-16 \| \| ENSG00000214106 \| PAXIP1-AS2 \| -2.43 \| 3.47E-17 \| 1.50E-16 \| \| ENSG00000089876 \| DHX32 \| 1.13 \| 3.56E-17 \| 1.53E-16 \| \| ENSG00000107897 \| ACBD5 \| -0.67 \| 3.66E-17 \| 1.57E-16 \| \| ENSG00000197183 \| NOL4L \| 0.90 \| 3.73E-17 \| 1.61E-16 \| \| ENSG00000120029 \| ARMH3 \| -0.63 \| 3.87E-17 \| 1.67E-16 \| \| ENSG00000116983 \| HPCAL4 \| -3.04 \| 4.02E-17 \| 1.73E-16 \| \| ENSG00000131470 \| PSMC3IP \| -1.11 \| 4.08E-17 \| 1.75E-16 \| \| ENSG00000133056 \| PIK3C2B \| -2.43 \| 4.20E-17 \| 1.80E-16 \| \| ENSG00000145332 \| KLHL8 \| 0.68 \| 4.24E-17 \| 1.82E-16 \| \| ENSG00000177406 \| LOC100049716 \| -1.09 \| 4.29E-17 \| 1.84E-16 \| \| ENSG00000132846 \| ZBED3 \| -2.31 \| 4.30E-17 \| 1.84E-16 \| \| ENSG00000091106 \| NLRC4 \| -1.80 \| 4.36E-17 \| 1.87E-16 \| \| ENSG00000101017 \| CD40 \| 0.71 \| 4.44E-17 \| 1.90E-16 \| \| ENSG00000154134 \| ROBO3 \| 0.71 \| 4.51E-17 \| 1.93E-16 \| \| ENSG00000174080 \| CTSF \| -1.13 \| 4.61E-17 \| 1.97E-16 \| \| ENSG00000238113 \| LINC01410 \| -1.31 \| 4.62E-17 \| 1.98E-16 \| \| ENSG00000109390 \| NDUFC1 \| -0.69 \| 4.65E-17 \| 1.99E-16 \| \| ENSG00000119686 \| FLVCR2 \| -1.81 \| 4.66E-17 \| 1.99E-16 \| \| ENSG00000120509 \| PDZD11 \| -0.69 \| 4.89E-17 \| 2.09E-16 \| \| ENSG00000106018 \| VIPR2 \| 5.30 \| 4.98E-17 \| 2.13E-16 \| \| ENSG00000254635 \| WAC-AS1 \| -0.69 \| 5.27E-17 \| 2.25E-16 \| \| ENSG00000172071 \| EIF2AK3 \| 0.76 \| 5.28E-17 \| 2.25E-16 \| \| ENSG00000134256 \| CD101 \| -1.70 \| 5.29E-17 \| 2.26E-16 \| \| ENSG00000138100 \| TRIM54 \| -1.31 \| 5.29E-17 \| 2.26E-16 \| \| ENSG00000183386 \| FHL3 \| -0.90 \| 5.44E-17 \| 2.32E-16 \| \| ENSG00000155846 \| PPARGC1B \| -1.03 \| 5.65E-17 \| 2.41E-16 \| \| ENSG00000254887 \| LOC100505622 \| 1.44 \| 5.79E-17 \| 2.47E-16 \| \| ENSG00000115008 \| IL1A \| 2.63 \| 5.93E-17 \| 2.53E-16 \| \| ENSG00000167202 \| TBC1D2B \| -1.01 \| 5.94E-17 \| 2.53E-16 \| \| ENSG00000176105 \| YES1 \| 0.72 \| 5.97E-17 \| 2.54E-16 \| \| ENSG00000163430 \| FSTL1 \| -1.69 \| 6.43E-17 \| 2.74E-16 \| \| ENSG00000177303 \| CASKIN2 \| -0.63 \| 6.65E-17 \| 2.83E-16 \| \| ENSG00000138622 \| HCN4 \| 5.69 \| 6.79E-17 \| 2.89E-16 \| \| ENSG00000258727 \| LOC102724814 \| -0.67 \| 6.88E-17 \| 2.92E-16 \| \| ENSG00000137364 \| TPMT \| 0.77 \| 7.13E-17 \| 3.02E-16 \| \| ENSG00000149289 \| ZC3H12C \| 0.73 \| 7.34E-17 \| 3.11E-16 \| \| ENSG00000115604 \| IL18R1 \| 1.68 \| 7.41E-17 \| 3.14E-16 \| \| ENSG00000238083 \| LRRC37A2 \| 1.02 \| 7.65E-17 \| 3.24E-16 \| \| ENSG00000164144 \| ARFIP1 \| 0.67 \| 7.69E-17 \| 3.25E-16 \| \| ENSG00000139998 \| RAB15 \| -0.97 \| 7.70E-17 \| 3.26E-16 \| \| ENSG00000187957 \| DNER \| 2.49 \| 7.71E-17 \| 3.26E-16 \| \| ENSG00000161911 \| TREML1 \| -0.69 \| 7.83E-17 \| 3.31E-16 \| \| ENSG00000134326 \| CMPK2 \| -2.83 \| 8.28E-17 \| 3.50E-16 \| \| ENSG00000176946 \| THAP4 \| -0.64 \| 8.41E-17 \| 3.55E-16 \| \| ENSG00000175662 \| TOM1L2 \| 0.71 \| 8.46E-17 \| 3.57E-16 \| \| ENSG00000114346 \| ECT2 \| -1.08 \| 8.53E-17 \| 3.60E-16 \| \| ENSG00000067646 \| ZFY \| 0.71 \| 8.72E-17 \| 3.68E-16 \| \| ENSG00000107949 \| BCCIP \| -0.65 \| 8.80E-17 \| 3.71E-16 \| \| ENSG00000154451 \| GBP5 \| -4.81 \| 9.68E-17 \| 4.08E-16 \| \| ENSG00000184702 \| 05-Sep \| -1.04 \| 1.12E-16 \| 4.73E-16 \| \| ENSG00000023909 \| GCLM \| 0.68 \| 1.17E-16 \| 4.92E-16 \| \| ENSG00000141452 \| RMC1 \| 0.66 \| 1.18E-16 \| 4.95E-16 \| \| ENSG00000266208 \|  \| -0.83 \| 1.18E-16 \| 4.95E-16 \| \| ENSG00000229271 \|  \| 3.54 \| 1.18E-16 \| 4.97E-16 \| \| ENSG00000138376 \| BARD1 \| -0.94 \| 1.20E-16 \| 5.04E-16 \| \| ENSG00000225138 \| SLC9A3-AS1 \| 0.60 \| 1.30E-16 \| 5.47E-16 \| \| ENSG00000178789 \| CD300LB \| -1.65 \| 1.35E-16 \| 5.65E-16 \| \| ENSG00000196072 \| BLOC1S2 \| -0.62 \| 1.35E-16 \| 5.65E-16 \| \| ENSG00000143341 \| HMCN1 \| -1.72 \| 1.36E-16 \| 5.70E-16 \| \| ENSG00000165669 \| FAM204A \| -0.85 \| 1.36E-16 \| 5.71E-16 \| \| ENSG00000095564 \| BTAF1 \| -0.62 \| 1.37E-16 \| 5.72E-16 \| \| ENSG00000215883 \| CYB5RL \| -1.04 \| 1.39E-16 \| 5.81E-16 \| \| ENSG00000117308 \| GALE \| -0.78 \| 1.44E-16 \| 6.04E-16 \| \| ENSG00000230844 \| ZNF674-AS1 \| 1.37 \| 1.50E-16 \| 6.26E-16 \| \| ENSG00000114948 \| ADAM23 \| 3.26 \| 1.50E-16 \| 6.29E-16 \| \| ENSG00000138658 \| ZGRF1 \| -1.48 \| 1.51E-16 \| 6.32E-16 \| \| ENSG00000184205 \| TSPYL2 \| 1.05 \| 1.53E-16 \| 6.40E-16 \| \| ENSG00000253414 \|  \| -1.53 \| 1.57E-16 \| 6.57E-16 \| \| ENSG00000125648 \| SLC25A23 \| -0.66 \| 1.58E-16 \| 6.62E-16 \| \| ENSG00000224397 \| SMIM25 \| 1.36 \| 1.61E-16 \| 6.73E-16 \| \| ENSG00000010292 \| NCAPD2 \| -0.63 \| 1.65E-16 \| 6.90E-16 \| \| ENSG00000108379 \| WNT3 \| 1.53 \| 1.66E-16 \| 6.92E-16 \| \| ENSG00000108379 \| LOC101929777 \| 1.53 \| 1.66E-16 \| 6.92E-16 \| \| ENSG00000139725 \| RHOF \| -0.76 \| 1.70E-16 \| 7.09E-16 \| \| ENSG00000184828 \| ZBTB7C \| -1.55 \| 1.70E-16 \| 7.10E-16 \| \| ENSG00000179085 \| DPM3 \| -0.76 \| 1.74E-16 \| 7.26E-16 \| \| ENSG00000127415 \| IDUA \| -0.60 \| 1.83E-16 \| 7.64E-16 \| \| ENSG00000138018 \| SELENOI \| 0.62 \| 1.88E-16 \| 7.82E-16 \| \| ENSG00000255833 \| TIFAB \| -2.28 \| 1.95E-16 \| 8.12E-16 \| \| ENSG00000157869 \| RAB28 \| 0.73 \| 2.01E-16 \| 8.35E-16 \| \| ENSG00000128973 \| CLN6 \| -0.64 \| 2.09E-16 \| 8.68E-16 \| \| ENSG00000183323 \| CCDC125 \| -1.39 \| 2.13E-16 \| 8.85E-16 \| \| ENSG00000162976 \| PQLC3 \| -0.85 \| 2.19E-16 \| 9.10E-16 \| \| ENSG00000183648 \| NDUFB1 \| -0.72 \| 2.24E-16 \| 9.27E-16 \| \| ENSG00000152778 \| IFIT5 \| -2.61 \| 2.37E-16 \| 9.83E-16 \| \| ENSG00000183765 \| CHEK2 \| -1.34 \| 2.43E-16 \| 1.01E-15 \| \| ENSG00000186654 \| PRR5 \| -1.61 \| 2.45E-16 \| 1.01E-15 \| \| ENSG00000003249 \| DBNDD1 \| -3.16 \| 2.52E-16 \| 1.04E-15 \| \| ENSG00000134470 \| IL15RA \| -3.21 \| 2.55E-16 \| 1.05E-15 \| \| ENSG00000168300 \| PCMTD1 \| 0.65 \| 2.55E-16 \| 1.05E-15 \| \| ENSG00000143816 \| WNT9A \| -1.73 \| 2.59E-16 \| 1.07E-15 \| \| ENSG00000123737 \| EXOSC9 \| -0.72 \| 2.67E-16 \| 1.10E-15 \| \| ENSG00000196550 \| FAM72A \| -1.29 \| 2.74E-16 \| 1.13E-15 \| \| ENSG00000164904 \| ALDH7A1 \| -4.78 \| 2.74E-16 \| 1.13E-15 \| \| ENSG00000182218 \| HHIPL1 \| -0.77 \| 2.77E-16 \| 1.14E-15 \| \| ENSG00000165660 \| ABRAXAS2 \| -0.65 \| 2.88E-16 \| 1.18E-15 \| \| ENSG00000130962 \| PRRG1 \| 0.83 \| 2.88E-16 \| 1.19E-15 \| \| ENSG00000168453 \| HR \| -4.27 \| 2.95E-16 \| 1.21E-15 \| \| ENSG00000138639 \| ARHGAP24 \| 3.35 \| 3.02E-16 \| 1.24E-15 \| \| ENSG00000267493 \| CIRBP-AS1 \| 0.90 \| 3.08E-16 \| 1.27E-15 \| \| ENSG00000123219 \| CENPK \| -2.05 \| 3.13E-16 \| 1.29E-15 \| \| ENSG00000144455 \| SUMF1 \| -0.59 \| 3.17E-16 \| 1.30E-15 \| \| ENSG00000144455 \| LOC100130207 \| -0.59 \| 3.17E-16 \| 1.30E-15 \| \| ENSG00000165633 \| VSTM4 \| 1.66 \| 3.34E-16 \| 1.37E-15 \| \| ENSG00000163735 \| CXCL5 \| 1.98 \| 3.41E-16 \| 1.40E-15 \| \| ENSG00000187372 \| PCDHB13 \| -3.11 \| 3.42E-16 \| 1.40E-15 \| \| ENSG00000152402 \| GUCY1A2 \| 1.01 \| 3.49E-16 \| 1.43E-15 \| \| ENSG00000137563 \| GGH \| -1.21 \| 3.68E-16 \| 1.51E-15 \| \| ENSG00000136982 \| DSCC1 \| -1.65 \| 4.21E-16 \| 1.73E-15 \| \| ENSG00000161981 \| SNRNP25 \| -0.87 \| 4.35E-16 \| 1.78E-15 \| \| ENSG00000169313 \| P2RY12 \| -6.89 \| 4.47E-16 \| 1.83E-15 \| \| ENSG00000109458 \| GAB1 \| 1.06 \| 4.60E-16 \| 1.88E-15 \| \| ENSG00000085365 \| SCAMP1 \| 0.60 \| 4.79E-16 \| 1.96E-15 \| \| ENSG00000135253 \| KCP \| -1.38 \| 4.90E-16 \| 2.01E-15 \| \| ENSG00000253831 \| ETV3L \| 1.24 \| 4.90E-16 \| 2.01E-15 \| \| ENSG00000104213 \| PDGFRL \| -4.46 \| 5.23E-16 \| 2.14E-15 \| \| ENSG00000047662 \| FAM184B \| -2.09 \| 5.28E-16 \| 2.16E-15 \| \| ENSG00000121900 \| TMEM54 \| -1.26 \| 5.48E-16 \| 2.24E-15 \| \| ENSG00000146281 \| PM20D2 \| -1.05 \| 5.66E-16 \| 2.31E-15 \| \| ENSG00000129355 \| CDKN2D \| 1.13 \| 5.67E-16 \| 2.31E-15 \| \| ENSG00000196584 \| XRCC2 \| -1.31 \| 5.68E-16 \| 2.32E-15 \| \| ENSG00000065057 \| NTHL1 \| -1.20 \| 5.84E-16 \| 2.38E-15 \| \| ENSG00000181274 \| FRAT2 \| -0.63 \| 5.87E-16 \| 2.39E-15 \| \| ENSG00000196814 \| MVB12B \| -0.72 \| 5.91E-16 \| 2.41E-15 \| \| ENSG00000115318 \| LOXL3 \| -0.81 \| 5.98E-16 \| 2.43E-15 \| \| ENSG00000133935 \| ERG28 \| -0.70 \| 6.18E-16 \| 2.51E-15 \| \| ENSG00000056998 \| GYG2 \| -1.99 \| 6.40E-16 \| 2.60E-15 \| \| ENSG00000133398 \| MED10 \| 0.62 \| 6.87E-16 \| 2.79E-15 \| \| ENSG00000142856 \| ITGB3BP \| -1.60 \| 6.92E-16 \| 2.81E-15 \| \| ENSG00000231877 \|  \| -1.20 \| 7.69E-16 \| 3.12E-15 \| \| ENSG00000104154 \| SLC30A4 \| 2.97 \| 7.77E-16 \| 3.15E-15 \| \| ENSG00000260231 \| KDM7A-DT \| 1.79 \| 7.88E-16 \| 3.20E-15 \| \| ENSG00000139211 \| AMIGO2 \| -1.45 \| 8.07E-16 \| 3.27E-15 \| \| ENSG00000163535 \| SGO2 \| -1.18 \| 8.77E-16 \| 3.55E-15 \| \| ENSG00000266976 \| LOC102724908 \| -9.51 \| 8.81E-16 \| 3.57E-15 \| \| ENSG00000173598 \| NUDT4 \| 0.60 \| 8.83E-16 \| 3.58E-15 \| \| ENSG00000173598 \| NUDT4B \| 0.60 \| 8.83E-16 \| 3.58E-15 \| \| ENSG00000120129 \| DUSP1 \| 0.80 \| 8.99E-16 \| 3.64E-15 \| \| ENSG00000144802 \| NFKBIZ \| 1.33 \| 8.99E-16 \| 3.64E-15 \| \| ENSG00000122126 \| OCRL \| -0.90 \| 9.56E-16 \| 3.87E-15 \| \| ENSG00000196664 \| TLR7 \| -1.35 \| 9.93E-16 \| 4.01E-15 \| \| ENSG00000110042 \| DTX4 \| 0.65 \| 9.98E-16 \| 4.03E-15 \| \| ENSG00000132256 \| TRIM5 \| -1.70 \| 1.02E-15 \| 4.12E-15 \| \| ENSG00000164885 \| CDK5 \| -0.65 \| 1.02E-15 \| 4.12E-15 \| \| ENSG00000185909 \| KLHDC8B \| -1.94 \| 1.06E-15 \| 4.26E-15 \| \| ENSG00000106617 \| PRKAG2 \| -0.85 \| 1.12E-15 \| 4.51E-15 \| \| ENSG00000152242 \| C18orf25 \| 0.60 \| 1.12E-15 \| 4.51E-15 \| \| ENSG00000125871 \| MGME1 \| -1.08 \| 1.14E-15 \| 4.58E-15 \| \| ENSG00000226067 \|  \| 0.68 \| 1.14E-15 \| 4.60E-15 \| \| ENSG00000138092 \| CENPO \| -0.70 \| 1.20E-15 \| 4.81E-15 \| \| ENSG00000177613 \| CSTF2T \| -0.73 \| 1.20E-15 \| 4.81E-15 \| \| ENSG00000186205 \| 01-Mar \| -1.99 \| 1.22E-15 \| 4.91E-15 \| \| ENSG00000243927 \| MRPS6 \| 0.69 \| 1.25E-15 \| 5.01E-15 \| \| ENSG00000168944 \| CEP120 \| -1.23 \| 1.25E-15 \| 5.01E-15 \| \| ENSG00000185187 \| SIGIRR \| -0.78 \| 1.32E-15 \| 5.29E-15 \| \| ENSG00000160766 \| GBAP1 \| -1.04 \| 1.34E-15 \| 5.36E-15 \| \| ENSG00000095485 \| CWF19L1 \| -0.97 \| 1.34E-15 \| 5.38E-15 \| \| ENSG00000061676 \| NCKAP1 \| 0.61 \| 1.36E-15 \| 5.46E-15 \| \| ENSG00000140807 \| NKD1 \| 1.04 \| 1.43E-15 \| 5.73E-15 \| \| ENSG00000248334 \| WHAMMP2 \| -2.64 \| 1.45E-15 \| 5.81E-15 \| \| ENSG00000177548 \| RABEP2 \| 0.60 \| 1.50E-15 \| 5.99E-15 \| \| ENSG00000179431 \| FJX1 \| -1.06 \| 1.51E-15 \| 6.03E-15 \| \| ENSG00000183579 \| ZNRF3 \| 0.78 \| 1.51E-15 \| 6.04E-15 \| \| ENSG00000183486 \| MX2 \| -1.02 \| 1.52E-15 \| 6.10E-15 \| \| ENSG00000164430 \| CGAS \| -1.16 \| 1.56E-15 \| 6.22E-15 \| \| ENSG00000277247 \|  \| 1.79 \| 1.57E-15 \| 6.26E-15 \| \| ENSG00000182175 \| RGMA \| 1.76 \| 1.60E-15 \| 6.39E-15 \| \| ENSG00000149636 \| DSN1 \| -1.04 \| 1.61E-15 \| 6.43E-15 \| \| ENSG00000147894 \| C9orf72 \| 1.18 \| 1.62E-15 \| 6.49E-15 \| \| ENSG00000142765 \| SYTL1 \| -4.06 \| 1.63E-15 \| 6.51E-15 \| \| ENSG00000128228 \| SDF2L1 \| -0.75 \| 1.69E-15 \| 6.73E-15 \| \| ENSG00000244045 \| TMEM199 \| 0.59 \| 1.78E-15 \| 7.10E-15 \| \| ENSG00000130653 \| PNPLA7 \| -1.25 \| 1.81E-15 \| 7.22E-15 \| \| ENSG00000104312 \| RIPK2 \| 0.60 \| 1.83E-15 \| 7.31E-15 \| \| ENSG00000232653 \| GOLGA8N \| -3.01 \| 1.95E-15 \| 7.74E-15 \| \| ENSG00000140987 \| ZSCAN32 \| 0.65 \| 1.97E-15 \| 7.82E-15 \| \| ENSG00000162433 \| AK4 \| 1.54 \| 2.02E-15 \| 8.02E-15 \| \| ENSG00000180573 \| HIST1H2AC \| 0.86 \| 2.15E-15 \| 8.53E-15 \| \| ENSG00000106804 \| C5 \| -1.33 \| 2.38E-15 \| 9.45E-15 \| \| ENSG00000158092 \| NCK1 \| -0.71 \| 2.54E-15 \| 1.01E-14 \| \| ENSG00000007129 \| CEACAM21 \| -1.96 \| 2.63E-15 \| 1.04E-14 \| \| ENSG00000147166 \| ITGB1BP2 \| -2.55 \| 2.63E-15 \| 1.04E-14 \| \| ENSG00000115896 \| PLCL1 \| 1.55 \| 2.79E-15 \| 1.11E-14 \| \| ENSG00000166478 \| ZNF143 \| 0.62 \| 2.80E-15 \| 1.11E-14 \| \| ENSG00000267080 \| ASB16-AS1 \| -0.89 \| 2.86E-15 \| 1.13E-14 \| \| ENSG00000129195 \| PIMREG \| -4.19 \| 2.88E-15 \| 1.14E-14 \| \| ENSG00000056291 \| NPFFR2 \| 2.69 \| 3.14E-15 \| 1.24E-14 \| \| ENSG00000174799 \| CEP135 \| 0.63 \| 3.14E-15 \| 1.24E-14 \| \| ENSG00000198720 \| ANKRD13B \| -1.31 \| 3.19E-15 \| 1.26E-14 \| \| ENSG00000124615 \| MOCS1 \| -4.37 \| 3.41E-15 \| 1.35E-14 \| \| ENSG00000154102 \| C16orf74 \| -2.63 \| 3.43E-15 \| 1.35E-14 \| \| ENSG00000138709 \| LARP1B \| 0.62 \| 3.44E-15 \| 1.36E-14 \| \| ENSG00000145476 \| CYP4V2 \| -0.93 \| 3.49E-15 \| 1.38E-14 \| \| ENSG00000171596 \| NMUR1 \| -2.97 \| 3.50E-15 \| 1.38E-14 \| \| ENSG00000134265 \| NAPG \| 0.64 \| 3.55E-15 \| 1.40E-14 \| \| ENSG00000259877 \|  \| 0.91 \| 3.58E-15 \| 1.41E-14 \| \| ENSG00000165410 \| CFL2 \| -0.99 \| 4.10E-15 \| 1.61E-14 \| \| ENSG00000090376 \| IRAK3 \| -0.88 \| 4.15E-15 \| 1.63E-14 \| \| ENSG00000004777 \| ARHGAP33 \| -0.80 \| 4.19E-15 \| 1.65E-14 \| \| ENSG00000175643 \| RMI2 \| -2.28 \| 4.37E-15 \| 1.72E-14 \| \| ENSG00000147119 \| CHST7 \| 0.73 \| 4.60E-15 \| 1.81E-14 \| \| ENSG00000198121 \| LPAR1 \| 0.64 \| 4.69E-15 \| 1.84E-14 \| \| ENSG00000149970 \| CNKSR2 \| 2.24 \| 4.74E-15 \| 1.86E-14 \| \| ENSG00000196511 \| TPK1 \| -0.79 \| 4.97E-15 \| 1.95E-14 \| \| ENSG00000163738 \| MTHFD2L \| -0.70 \| 4.99E-15 \| 1.96E-14 \| \| ENSG00000124570 \| SERPINB6 \| -0.77 \| 5.01E-15 \| 1.96E-14 \| \| ENSG00000143603 \| KCNN3 \| 4.43 \| 5.06E-15 \| 1.98E-14 \| \| ENSG00000143797 \| MBOAT2 \| -1.86 \| 5.09E-15 \| 1.99E-14 \| \| ENSG00000165072 \| MAMDC2 \| -9.29 \| 5.35E-15 \| 2.09E-14 \| \| ENSG00000185338 \| SOCS1 \| -2.10 \| 5.39E-15 \| 2.11E-14 \| \| ENSG00000213903 \| LTB4R \| -1.09 \| 5.42E-15 \| 2.12E-14 \| \| ENSG00000128284 \| APOL3 \| -3.21 \| 5.48E-15 \| 2.14E-14 \| \| ENSG00000159023 \| EPB41 \| -0.89 \| 5.55E-15 \| 2.17E-14 \| \| ENSG00000151422 \| FER \| 0.95 \| 5.60E-15 \| 2.19E-14 \| \| ENSG00000234420 \| ZNF37BP \| -1.05 \| 5.74E-15 \| 2.24E-14 \| \| ENSG00000124772 \| CPNE5 \| -2.96 \| 5.77E-15 \| 2.25E-14 \| \| ENSG00000128590 \| DNAJB9 \| 0.73 \| 5.90E-15 \| 2.30E-14 \| \| ENSG00000235823 \| OLMALINC \| -1.39 \| 5.94E-15 \| 2.31E-14 \| \| ENSG00000124731 \| TREM1 \| 1.20 \| 6.13E-15 \| 2.39E-14 \| \| ENSG00000243742 \| RPLP0P2 \| -1.40 \| 6.22E-15 \| 2.42E-14 \| \| ENSG00000164221 \| CCDC112 \| -1.62 \| 6.24E-15 \| 2.43E-14 \| \| ENSG00000010539 \| ZNF200 \| 0.71 \| 6.48E-15 \| 2.52E-14 \| \| ENSG00000136856 \| SLC2A8 \| -1.06 \| 6.81E-15 \| 2.65E-14 \| \| ENSG00000196878 \| LAMB3 \| -0.69 \| 6.84E-15 \| 2.66E-14 \| \| ENSG00000224032 \| EPB41L4A-AS1 \| 0.64 \| 6.93E-15 \| 2.69E-14 \| \| ENSG00000106333 \| PCOLCE \| 1.61 \| 7.26E-15 \| 2.82E-14 \| \| ENSG00000111602 \| TIMELESS \| -0.61 \| 7.38E-15 \| 2.86E-14 \| \| ENSG00000138382 \| METTL5 \| -0.78 \| 7.56E-15 \| 2.93E-14 \| \| ENSG00000175105 \| ZNF654 \| 0.80 \| 7.85E-15 \| 3.04E-14 \| \| ENSG00000183666 \| GUSBP1 \| 1.02 \| 8.06E-15 \| 3.12E-14 \| \| ENSG00000167216 \| KATNAL2 \| -1.78 \| 8.49E-15 \| 3.28E-14 \| \| ENSG00000231574 \| LINC02015 \| 5.38 \| 8.60E-15 \| 3.33E-14 \| \| ENSG00000159231 \| CBR3 \| -1.79 \| 8.66E-15 \| 3.35E-14 \| \| ENSG00000119979 \| FAM45A \| -0.81 \| 8.68E-15 \| 3.35E-14 \| \| ENSG00000068831 \| RASGRP2 \| -1.35 \| 8.71E-15 \| 3.36E-14 \| \| ENSG00000106100 \| NOD1 \| -1.65 \| 8.79E-15 \| 3.40E-14 \| \| ENSG00000213930 \| GALT \| -0.93 \| 8.83E-15 \| 3.41E-14 \| \| ENSG00000099849 \| RASSF7 \| -0.85 \| 8.86E-15 \| 3.42E-14 \| \| ENSG00000000460 \| C1orf112 \| -1.51 \| 8.90E-15 \| 3.44E-14 \| \| ENSG00000178150 \| ZNF114 \| -2.18 \| 9.22E-15 \| 3.56E-14 \| \| ENSG00000213186 \| TRIM59 \| -1.51 \| 9.90E-15 \| 3.82E-14 \| \| ENSG00000073417 \| PDE8A \| 0.65 \| 1.01E-14 \| 3.90E-14 \| \| ENSG00000164535 \| DAGLB \| -0.64 \| 1.03E-14 \| 3.98E-14 \| \| ENSG00000112379 \| ARFGEF3 \| 0.96 \| 1.05E-14 \| 4.05E-14 \| \| ENSG00000228956 \|  \| -2.67 \| 1.06E-14 \| 4.07E-14 \| \| ENSG00000260941 \| LINC00622 \| 2.38 \| 1.11E-14 \| 4.27E-14 \| \| ENSG00000180592 \| SKIDA1 \| -3.80 \| 1.13E-14 \| 4.33E-14 \| \| ENSG00000135094 \| SDS \| -2.59 \| 1.17E-14 \| 4.51E-14 \| \| ENSG00000185418 \| TARSL2 \| 1.03 \| 1.21E-14 \| 4.64E-14 \| \| ENSG00000161692 \| DBF4B \| -1.01 \| 1.23E-14 \| 4.72E-14 \| \| ENSG00000165195 \| PIGA \| 0.74 \| 1.25E-14 \| 4.80E-14 \| \| ENSG00000120053 \| GOT1 \| -1.19 \| 1.33E-14 \| 5.12E-14 \| \| ENSG00000224870 \| MRPL20-AS1 \| 0.66 \| 1.34E-14 \| 5.15E-14 \| \| ENSG00000173267 \| SNCG \| -0.99 \| 1.40E-14 \| 5.36E-14 \| \| ENSG00000159228 \| CBR1 \| -0.67 \| 1.42E-14 \| 5.45E-14 \| \| ENSG00000166016 \| ABTB2 \| 0.91 \| 1.43E-14 \| 5.49E-14 \| \| ENSG00000165457 \| FOLR2 \| -3.78 \| 1.45E-14 \| 5.56E-14 \| \| ENSG00000181195 \| PENK \| -2.88 \| 1.51E-14 \| 5.76E-14 \| \| ENSG00000104974 \| LILRA1 \| -3.50 \| 1.52E-14 \| 5.80E-14 \| \| ENSG00000061918 \| GUCY1B1 \| 1.21 \| 1.54E-14 \| 5.87E-14 \| \| ENSG00000141295 \| SCRN2 \| -0.78 \| 1.57E-14 \| 6.00E-14 \| \| ENSG00000090339 \| ICAM1 \| 0.86 \| 1.60E-14 \| 6.11E-14 \| \| ENSG00000132000 \| PODNL1 \| -1.73 \| 1.64E-14 \| 6.25E-14 \| \| ENSG00000129493 \| HEATR5A \| 1.09 \| 1.71E-14 \| 6.52E-14 \| \| ENSG00000154864 \| PIEZO2 \| -3.37 \| 1.72E-14 \| 6.55E-14 \| \| ENSG00000120798 \| NR2C1 \| -0.68 \| 1.92E-14 \| 7.33E-14 \| \| ENSG00000230124 \| ACBD6 \| -0.65 \| 2.08E-14 \| 7.90E-14 \| \| ENSG00000230124 \| LHX4-AS1 \| -0.65 \| 2.08E-14 \| 7.90E-14 \| \| ENSG00000064651 \| SLC12A2 \| 0.70 \| 2.09E-14 \| 7.94E-14 \| \| ENSG00000113211 \| PCDHB6 \| -1.60 \| 2.10E-14 \| 7.99E-14 \| \| ENSG00000150787 \| PTS \| 1.02 \| 2.14E-14 \| 8.13E-14 \| \| ENSG00000260918 \|  \| 4.42 \| 2.18E-14 \| 8.27E-14 \| \| ENSG00000135541 \| AHI1 \| 0.67 \| 2.25E-14 \| 8.55E-14 \| \| ENSG00000122376 \| SHLD2 \| -0.60 \| 2.27E-14 \| 8.62E-14 \| \| ENSG00000135063 \| FAM189A2 \| -3.08 \| 2.30E-14 \| 8.73E-14 \| \| ENSG00000198554 \| WDHD1 \| -1.01 \| 2.35E-14 \| 8.91E-14 \| \| ENSG00000069869 \| NEDD4 \| 0.70 \| 2.40E-14 \| 9.09E-14 \| \| ENSG00000100906 \| NFKBIA \| 1.07 \| 2.43E-14 \| 9.20E-14 \| \| ENSG00000241399 \| CD302 \| -0.90 \| 2.62E-14 \| 9.91E-14 \| \| ENSG00000183773 \| AIFM3 \| -1.44 \| 2.64E-14 \| 1.00E-13 \| \| ENSG00000237036 \| ZEB1-AS1 \| -1.77 \| 2.76E-14 \| 1.04E-13 \| \| ENSG00000115365 \| LANCL1 \| -0.84 \| 2.77E-14 \| 1.05E-13 \| \| ENSG00000263961 \| RHEX \| -4.75 \| 2.86E-14 \| 1.08E-13 \| \| ENSG00000236546 \| LOC105378668 \| 1.42 \| 2.87E-14 \| 1.08E-13 \| \| ENSG00000171223 \| JUNB \| 0.66 \| 2.88E-14 \| 1.09E-13 \| \| ENSG00000111912 \| NCOA7 \| -0.59 \| 2.99E-14 \| 1.13E-13 \| \| ENSG00000170684 \| ZNF296 \| -0.95 \| 3.00E-14 \| 1.13E-13 \| \| ENSG00000188626 \| GOLGA8M \| -3.43 \| 3.10E-14 \| 1.17E-13 \| \| ENSG00000187601 \| MAGEH1 \| 0.87 \| 3.18E-14 \| 1.20E-13 \| \| ENSG00000257743 \| MGAM2 \| 2.70 \| 3.27E-14 \| 1.23E-13 \| \| ENSG00000143457 \| GOLPH3L \| -0.64 \| 3.43E-14 \| 1.29E-13 \| \| ENSG00000112149 \| CD83 \| 1.14 \| 3.45E-14 \| 1.30E-13 \| \| ENSG00000198624 \| CCDC69 \| -0.81 \| 3.47E-14 \| 1.31E-13 \| \| ENSG00000234883 \| MIR155HG \| -1.03 \| 3.47E-14 \| 1.31E-13 \| \| ENSG00000111249 \| CUX2 \| -1.10 \| 3.77E-14 \| 1.42E-13 \| \| ENSG00000235770 \| LINC00607 \| 2.83 \| 3.79E-14 \| 1.42E-13 \| \| ENSG00000214113 \| LYRM4 \| 0.73 \| 3.87E-14 \| 1.46E-13 \| \| ENSG00000171502 \| COL24A1 \| -1.33 \| 3.93E-14 \| 1.48E-13 \| \| ENSG00000188763 \| FZD9 \| 1.27 \| 3.99E-14 \| 1.50E-13 \| \| ENSG00000171792 \| RHNO1 \| -0.95 \| 4.06E-14 \| 1.52E-13 \| \| ENSG00000267123 \| SCAT1 \| 1.08 \| 4.41E-14 \| 1.66E-13 \| \| ENSG00000215068 \| LOC153684 \| -2.33 \| 4.67E-14 \| 1.75E-13 \| \| ENSG00000162929 \| KIAA1841 \| -1.08 \| 4.85E-14 \| 1.82E-13 \| \| ENSG00000127951 \| FGL2 \| -3.68 \| 4.92E-14 \| 1.84E-13 \| \| ENSG00000168813 \| ZNF507 \| 0.63 \| 5.22E-14 \| 1.96E-13 \| \| ENSG00000258634 \|  \| 1.14 \| 5.27E-14 \| 1.97E-13 \| \| ENSG00000037897 \| METTL1 \| 0.60 \| 5.44E-14 \| 2.04E-13 \| \| ENSG00000111581 \| NUP107 \| -0.60 \| 5.45E-14 \| 2.04E-13 \| \| ENSG00000115641 \| FHL2 \| -1.10 \| 5.52E-14 \| 2.07E-13 \| \| ENSG00000138356 \| AOX1 \| 2.54 \| 5.56E-14 \| 2.08E-13 \| \| ENSG00000183763 \| TRAIP \| -1.42 \| 5.87E-14 \| 2.19E-13 \| \| ENSG00000100347 \| SAMM50 \| -0.59 \| 6.01E-14 \| 2.24E-13 \| \| ENSG00000203814 \| HIST2H2BF \| 1.83 \| 6.03E-14 \| 2.25E-13 \| \| ENSG00000067606 \| PRKCZ \| -1.59 \| 6.07E-14 \| 2.26E-13 \| \| ENSG00000065485 \| PDIA5 \| -0.70 \| 6.10E-14 \| 2.27E-13 \| \| ENSG00000135929 \| CYP27A1 \| -0.99 \| 6.13E-14 \| 2.29E-13 \| \| ENSG00000137965 \| IFI44 \| -4.42 \| 6.32E-14 \| 2.36E-13 \| \| ENSG00000066056 \| TIE1 \| -0.99 \| 6.67E-14 \| 2.49E-13 \| \| ENSG00000197093 \| GAL3ST4 \| -1.80 \| 6.75E-14 \| 2.52E-13 \| \| ENSG00000138303 \| ASCC1 \| -0.80 \| 6.83E-14 \| 2.54E-13 \| \| ENSG00000157570 \| TSPAN18 \| -2.08 \| 6.93E-14 \| 2.58E-13 \| \| ENSG00000152595 \| MEPE \| -2.38 \| 7.03E-14 \| 2.62E-13 \| \| ENSG00000076662 \| ICAM3 \| -0.67 \| 7.46E-14 \| 2.77E-13 \| \| ENSG00000234380 \| LINC01426 \| 1.12 \| 7.51E-14 \| 2.79E-13 \| \| ENSG00000091127 \| PUS7 \| 0.61 \| 7.74E-14 \| 2.87E-13 \| \| ENSG00000133059 \| DSTYK \| -0.94 \| 7.87E-14 \| 2.92E-13 \| \| ENSG00000271216 \| LINC01050 \| 1.88 \| 7.96E-14 \| 2.96E-13 \| \| ENSG00000185345 \| PRKN \| 1.71 \| 8.51E-14 \| 3.16E-13 \| \| ENSG00000268996 \| MAN1B1-DT \| 1.10 \| 8.77E-14 \| 3.25E-13 \| \| ENSG00000174516 \| PELI3 \| 0.73 \| 8.98E-14 \| 3.33E-13 \| \| ENSG00000111666 \| CHPT1 \| -1.17 \| 9.15E-14 \| 3.39E-13 \| \| ENSG00000145780 \| FEM1C \| -0.65 \| 9.24E-14 \| 3.42E-13 \| \| ENSG00000236915 \|  \| 2.10 \| 9.25E-14 \| 3.42E-13 \| \| ENSG00000189180 \| ZNF33A \| -0.78 \| 9.54E-14 \| 3.53E-13 \| \| ENSG00000162592 \| CCDC27 \| -1.57 \| 1.01E-13 \| 3.74E-13 \| \| ENSG00000186638 \| KIF24 \| -1.27 \| 1.04E-13 \| 3.85E-13 \| \| ENSG00000116095 \| PLEKHA3 \| 0.66 \| 1.13E-13 \| 4.16E-13 \| \| ENSG00000149131 \| SERPING1 \| -3.53 \| 1.13E-13 \| 4.18E-13 \| \| ENSG00000164603 \| BMT2 \| 1.16 \| 1.15E-13 \| 4.24E-13 \| \| ENSG00000165272 \| AQP3 \| 0.88 \| 1.19E-13 \| 4.41E-13 \| \| ENSG00000162062 \| TEDC2 \| -1.31 \| 1.20E-13 \| 4.41E-13 \| \| ENSG00000152192 \| POU4F1 \| 1.48 \| 1.25E-13 \| 4.59E-13 \| \| ENSG00000129465 \| RIPK3 \| -1.06 \| 1.28E-13 \| 4.73E-13 \| \| ENSG00000141696 \| P3H4 \| -2.11 \| 1.34E-13 \| 4.93E-13 \| \| ENSG00000257038 \| LOC100287837 \| 1.67 \| 1.45E-13 \| 5.33E-13 \| \| ENSG00000169131 \| ZNF354A \| 0.65 \| 1.53E-13 \| 5.63E-13 \| \| ENSG00000114739 \| ACVR2B \| 0.74 \| 1.61E-13 \| 5.92E-13 \| \| ENSG00000072163 \| LIMS2 \| -2.65 \| 1.65E-13 \| 6.05E-13 \| \| ENSG00000101574 \| METTL4 \| 0.84 \| 1.67E-13 \| 6.14E-13 \| \| ENSG00000186532 \| SMYD4 \| -1.16 \| 1.74E-13 \| 6.40E-13 \| \| ENSG00000123353 \| ORMDL2 \| 0.62 \| 1.80E-13 \| 6.61E-13 \| \| ENSG00000204301 \| NOTCH4 \| 1.67 \| 1.81E-13 \| 6.62E-13 \| \| ENSG00000076321 \| KLHL20 \| -0.67 \| 1.91E-13 \| 7.00E-13 \| \| ENSG00000180871 \| CXCR2 \| -2.94 \| 1.95E-13 \| 7.15E-13 \| \| ENSG00000132749 \| TESMIN \| -1.27 \| 1.99E-13 \| 7.26E-13 \| \| ENSG00000155966 \| AFF2 \| 1.07 \| 2.00E-13 \| 7.31E-13 \| \| ENSG00000186908 \| ZDHHC17 \| 0.66 \| 2.10E-13 \| 7.66E-13 \| \| ENSG00000164306 \| PRIMPOL \| -1.43 \| 2.12E-13 \| 7.74E-13 \| \| ENSG00000175595 \| ERCC4 \| 0.70 \| 2.19E-13 \| 8.01E-13 \| \| ENSG00000051596 \| THOC3 \| -0.94 \| 2.27E-13 \| 8.27E-13 \| \| ENSG00000259517 \| LINC02169 \| -3.64 \| 2.29E-13 \| 8.36E-13 \| \| ENSG00000146143 \| PRIM2 \| -0.93 \| 2.34E-13 \| 8.52E-13 \| \| ENSG00000164087 \| POC1A \| -0.85 \| 2.53E-13 \| 9.21E-13 \| \| ENSG00000213465 \| ARL2 \| -0.75 \| 2.57E-13 \| 9.36E-13 \| \| ENSG00000188312 \| CENPP \| -1.17 \| 2.70E-13 \| 9.82E-13 \| \| ENSG00000164532 \| TBX20 \| 1.60 \| 2.75E-13 \| 1.00E-12 \| \| ENSG00000166452 \| AKIP1 \| -0.61 \| 2.76E-13 \| 1.00E-12 \| \| ENSG00000170412 \| GPRC5C \| -1.41 \| 2.88E-13 \| 1.05E-12 \| \| ENSG00000231346 \| LINC01160 \| -1.20 \| 2.96E-13 \| 1.07E-12 \| \| ENSG00000129235 \| TXNDC17 \| -0.77 \| 3.16E-13 \| 1.15E-12 \| \| ENSG00000119661 \| DNAL1 \| 0.94 \| 3.20E-13 \| 1.16E-12 \| \| ENSG00000136144 \| RCBTB1 \| -0.64 \| 3.26E-13 \| 1.18E-12 \| \| ENSG00000168061 \| SAC3D1 \| -1.01 \| 3.27E-13 \| 1.18E-12 \| \| ENSG00000121769 \| FABP3 \| -3.62 \| 3.38E-13 \| 1.23E-12 \| \| ENSG00000144161 \| ZC3H8 \| 0.76 \| 3.54E-13 \| 1.28E-12 \| \| ENSG00000120458 \| MSANTD2 \| 1.10 \| 3.54E-13 \| 1.28E-12 \| \| ENSG00000170962 \| PDGFD \| -1.80 \| 3.61E-13 \| 1.31E-12 \| \| ENSG00000130054 \| FAM155B \| -2.45 \| 3.86E-13 \| 1.40E-12 \| \| ENSG00000162650 \| ATXN7L2 \| 0.61 \| 3.98E-13 \| 1.43E-12 \| \| ENSG00000228158 \|  \| 2.52 \| 4.02E-13 \| 1.45E-12 \| \| ENSG00000152382 \| TADA1 \| -0.96 \| 4.07E-13 \| 1.47E-12 \| \| ENSG00000134864 \| GGACT \| -0.77 \| 4.25E-13 \| 1.53E-12 \| \| ENSG00000214357 \| NEURL1B \| -3.80 \| 4.39E-13 \| 1.58E-12 \| \| ENSG00000089041 \| P2RX7 \| -2.31 \| 4.47E-13 \| 1.61E-12 \| \| ENSG00000197520 \| FAM177B \| 3.25 \| 4.64E-13 \| 1.67E-12 \| \| ENSG00000100228 \| RAB36 \| -5.46 \| 4.66E-13 \| 1.68E-12 \| \| ENSG00000107201 \| DDX58 \| -1.27 \| 4.83E-13 \| 1.74E-12 \| \| ENSG00000165832 \| TRUB1 \| -1.08 \| 4.84E-13 \| 1.74E-12 \| \| ENSG00000257167 \| TMPO-AS1 \| -1.03 \| 4.84E-13 \| 1.74E-12 \| \| ENSG00000163923 \| RPL39L \| -1.18 \| 5.03E-13 \| 1.80E-12 \| \| ENSG00000226688 \| ENTPD1-AS1 \| -1.24 \| 5.10E-13 \| 1.83E-12 \| \| ENSG00000171843 \| MLLT3 \| 1.14 \| 5.13E-13 \| 1.84E-12 \| \| ENSG00000026950 \| BTN3A1 \| -1.21 \| 5.73E-13 \| 2.05E-12 \| \| ENSG00000231233 \| CFAP58-DT \| 2.77 \| 5.73E-13 \| 2.05E-12 \| \| ENSG00000111445 \| RFC5 \| -0.69 \| 5.78E-13 \| 2.07E-12 \| \| ENSG00000231752 \| EMBP1 \| -2.22 \| 5.96E-13 \| 2.13E-12 \| \| ENSG00000148468 \| FAM171A1 \| -2.58 \| 6.06E-13 \| 2.17E-12 \| \| ENSG00000198937 \| CCDC167 \| -1.04 \| 6.09E-13 \| 2.17E-12 \| \| ENSG00000111247 \| RAD51AP1 \| -2.30 \| 6.16E-13 \| 2.20E-12 \| \| ENSG00000152457 \| DCLRE1C \| -0.90 \| 6.35E-13 \| 2.26E-12 \| \| ENSG00000122733 \| PHF24 \| 2.56 \| 6.65E-13 \| 2.37E-12 \| \| ENSG00000144115 \| THNSL2 \| 1.22 \| 6.68E-13 \| 2.38E-12 \| \| ENSG00000168280 \| KIF5C \| 1.79 \| 6.70E-13 \| 2.39E-12 \| \| ENSG00000103404 \| USP31 \| 0.64 \| 6.73E-13 \| 2.40E-12 \| \| ENSG00000135617 \| PRADC1 \| -0.84 \| 6.91E-13 \| 2.46E-12 \| \| ENSG00000146243 \| IRAK1BP1 \| -1.85 \| 6.93E-13 \| 2.47E-12 \| \| ENSG00000079462 \| PAFAH1B3 \| -0.86 \| 7.11E-13 \| 2.53E-12 \| \| ENSG00000175262 \| C1orf127 \| -3.83 \| 7.14E-13 \| 2.54E-12 \| \| ENSG00000172731 \| LRRC20 \| -2.54 \| 7.21E-13 \| 2.56E-12 \| \| ENSG00000115540 \| MOB4 \| 0.62 \| 7.22E-13 \| 2.57E-12 \| \| ENSG00000245060 \| LINC00847 \| -0.97 \| 7.33E-13 \| 2.60E-12 \| \| ENSG00000149743 \| TRPT1 \| 0.85 \| 7.34E-13 \| 2.61E-12 \| \| ENSG00000156521 \| TYSND1 \| -1.11 \| 7.38E-13 \| 2.62E-12 \| \| ENSG00000103089 \| FA2H \| -2.12 \| 7.63E-13 \| 2.71E-12 \| \| ENSG00000131848 \| ZSCAN5A \| 1.19 \| 7.91E-13 \| 2.81E-12 \| \| ENSG00000182168 \| UNC5C \| 3.65 \| 8.33E-13 \| 2.95E-12 \| \| ENSG00000179922 \| ZNF784 \| 0.87 \| 8.78E-13 \| 3.11E-12 \| \| ENSG00000151500 \| THYN1 \| -0.74 \| 8.99E-13 \| 3.18E-12 \| \| ENSG00000270629 \| NBPF14 \| 1.01 \| 9.10E-13 \| 3.22E-12 \| \| ENSG00000270629 \| NOTCH2NLB \| 1.01 \| 9.10E-13 \| 3.22E-12 \| \| ENSG00000170892 \| TSEN34 \| 0.65 \| 9.24E-13 \| 3.26E-12 \| \| ENSG00000153064 \| BANK1 \| -2.02 \| 9.56E-13 \| 3.38E-12 \| \| ENSG00000127328 \| RAB3IP \| 1.25 \| 9.97E-13 \| 3.52E-12 \| \| ENSG00000103174 \| NAGPA \| -0.74 \| 1.05E-12 \| 3.69E-12 \| \| ENSG00000165899 \| OTOGL \| 1.79 \| 1.09E-12 \| 3.84E-12 \| \| ENSG00000147255 \| IGSF1 \| -3.15 \| 1.10E-12 \| 3.88E-12 \| \| ENSG00000075651 \| PLD1 \| -1.43 \| 1.13E-12 \| 3.97E-12 \| \| ENSG00000230069 \|  \| 1.55 \| 1.19E-12 \| 4.17E-12 \| \| ENSG00000161905 \| ALOX15 \| -3.11 \| 1.19E-12 \| 4.18E-12 \| \| ENSG00000123836 \| PFKFB2 \| -2.22 \| 1.22E-12 \| 4.30E-12 \| \| ENSG00000157601 \| MX1 \| -2.10 \| 1.26E-12 \| 4.43E-12 \| \| ENSG00000198729 \| PPP1R14C \| 0.84 \| 1.28E-12 \| 4.50E-12 \| \| ENSG00000029534 \| ANK1 \| -1.34 \| 1.32E-12 \| 4.63E-12 \| \| ENSG00000174600 \| CMKLR1 \| -2.40 \| 1.39E-12 \| 4.87E-12 \| \| ENSG00000141447 \| OSBPL1A \| -0.62 \| 1.57E-12 \| 5.49E-12 \| \| ENSG00000235316 \|  \| -0.90 \| 1.69E-12 \| 5.90E-12 \| \| ENSG00000186364 \| NUDT17 \| -1.39 \| 1.71E-12 \| 5.98E-12 \| \| ENSG00000185361 \| TNFAIP8L1 \| 0.69 \| 1.76E-12 \| 6.15E-12 \| \| ENSG00000115504 \| EHBP1 \| 0.59 \| 1.81E-12 \| 6.32E-12 \| \| ENSG00000058335 \| RASGRF1 \| -0.64 \| 1.85E-12 \| 6.45E-12 \| \| ENSG00000150907 \| FOXO1 \| -1.12 \| 1.88E-12 \| 6.55E-12 \| \| ENSG00000124224 \| PPP4R1L \| 0.77 \| 2.02E-12 \| 7.03E-12 \| \| ENSG00000121454 \| LHX4 \| 3.22 \| 2.05E-12 \| 7.13E-12 \| \| ENSG00000107864 \| CPEB3 \| -1.79 \| 2.08E-12 \| 7.25E-12 \| \| ENSG00000234191 \|  \| 0.96 \| 2.09E-12 \| 7.28E-12 \| \| ENSG00000008517 \| IL32 \| -1.33 \| 2.10E-12 \| 7.33E-12 \| \| ENSG00000103313 \| MEFV \| -1.40 \| 2.11E-12 \| 7.36E-12 \| \| ENSG00000111490 \| TBC1D30 \| 1.11 \| 2.13E-12 \| 7.40E-12 \| \| ENSG00000140104 \| CLBA1 \| 0.62 \| 2.13E-12 \| 7.40E-12 \| \| ENSG00000213347 \| MXD3 \| -0.95 \| 2.27E-12 \| 7.90E-12 \| \| ENSG00000114988 \| LMAN2L \| -0.68 \| 2.33E-12 \| 8.10E-12 \| \| ENSG00000145604 \| SKP2 \| -0.96 \| 2.73E-12 \| 9.46E-12 \| \| ENSG00000008283 \| CYB561 \| -0.99 \| 2.77E-12 \| 9.61E-12 \| \| ENSG00000120332 \| TNN \| -2.28 \| 2.80E-12 \| 9.71E-12 \| \| ENSG00000189223 \| PAX8-AS1 \| -0.99 \| 2.96E-12 \| 1.02E-11 \| \| ENSG00000121075 \| TBX4 \| -1.90 \| 3.06E-12 \| 1.06E-11 \| \| ENSG00000232559 \|  \| 0.80 \| 3.12E-12 \| 1.08E-11 \| \| ENSG00000184465 \| WDR27 \| -0.62 \| 3.13E-12 \| 1.08E-11 \| \| ENSG00000272114 \|  \| 3.15 \| 3.21E-12 \| 1.11E-11 \| \| ENSG00000171840 \| NINJ2 \| -1.29 \| 3.28E-12 \| 1.13E-11 \| \| ENSG00000153044 \| CENPH \| -1.41 \| 3.44E-12 \| 1.19E-11 \| \| ENSG00000225434 \| LINC01504 \| -1.60 \| 3.52E-12 \| 1.21E-11 \| \| ENSG00000187764 \| SEMA4D \| -0.70 \| 3.52E-12 \| 1.21E-11 \| \| ENSG00000139625 \| MAP3K12 \| 0.73 \| 3.58E-12 \| 1.23E-11 \| \| ENSG00000107807 \| TLX1 \| -8.34 \| 3.62E-12 \| 1.25E-11 \| \| ENSG00000155287 \| SLC25A28 \| -0.60 \| 3.71E-12 \| 1.28E-11 \| \| ENSG00000008441 \| NFIX \| -0.81 \| 3.77E-12 \| 1.30E-11 \| \| ENSG00000198842 \| DUSP27 \| -6.00 \| 3.79E-12 \| 1.30E-11 \| \| ENSG00000090971 \| NAT14 \| 0.75 \| 3.84E-12 \| 1.32E-11 \| \| ENSG00000148444 \| COMMD3 \| -1.94 \| 3.88E-12 \| 1.33E-11 \| \| ENSG00000146038 \| DCDC2 \| 2.04 \| 3.89E-12 \| 1.33E-11 \| \| ENSG00000238042 \| LINC02257 \| 2.49 \| 3.90E-12 \| 1.34E-11 \| \| ENSG00000130244 \| FAM98C \| -0.86 \| 3.96E-12 \| 1.36E-11 \| \| ENSG00000102221 \| JADE3 \| 1.06 \| 4.00E-12 \| 1.37E-11 \| \| ENSG00000189420 \| ZFP92 \| 1.21 \| 4.11E-12 \| 1.41E-11 \| \| ENSG00000101445 \| PPP1R16B \| -0.71 \| 4.20E-12 \| 1.44E-11 \| \| ENSG00000116774 \| OLFML3 \| -3.95 \| 4.31E-12 \| 1.47E-11 \| \| ENSG00000066248 \| NGEF \| -0.91 \| 4.33E-12 \| 1.48E-11 \| \| ENSG00000231721 \| LINC-PINT \| 1.19 \| 4.40E-12 \| 1.50E-11 \| \| ENSG00000230107 \|  \| -2.72 \| 4.57E-12 \| 1.56E-11 \| \| ENSG00000083857 \| FAT1 \| -0.75 \| 4.97E-12 \| 1.69E-11 \| \| ENSG00000128408 \| RIBC2 \| -2.50 \| 5.00E-12 \| 1.71E-11 \| \| ENSG00000146374 \| RSPO3 \| 3.28 \| 5.04E-12 \| 1.72E-11 \| \| ENSG00000151743 \| AMN1 \| 0.84 \| 5.13E-12 \| 1.75E-11 \| \| ENSG00000103145 \| HCFC1R1 \| -0.60 \| 5.41E-12 \| 1.84E-11 \| \| ENSG00000121005 \| CRISPLD1 \| -2.66 \| 5.55E-12 \| 1.89E-11 \| \| ENSG00000161558 \| TMEM143 \| -1.01 \| 5.70E-12 \| 1.94E-11 \| \| ENSG00000262246 \| CORO7 \| -0.74 \| 6.07E-12 \| 2.06E-11 \| \| ENSG00000026559 \| KCNG1 \| 0.82 \| 6.71E-12 \| 2.27E-11 \| \| ENSG00000174442 \| ZWILCH \| -0.81 \| 6.82E-12 \| 2.31E-11 \| \| ENSG00000141655 \| TNFRSF11A \| 1.05 \| 6.82E-12 \| 2.31E-11 \| \| ENSG00000248487 \| ABHD14A \| -1.20 \| 6.96E-12 \| 2.36E-11 \| \| ENSG00000120498 \| TEX11 \| -8.21 \| 7.05E-12 \| 2.39E-11 \| \| ENSG00000132376 \| INPP5K \| 0.63 \| 7.76E-12 \| 2.63E-11 \| \| ENSG00000197380 \| DACT3 \| 1.58 \| 7.79E-12 \| 2.64E-11 \| \| ENSG00000203760 \| CENPW \| -1.44 \| 8.00E-12 \| 2.70E-11 \| \| ENSG00000253187 \| HOXA10-AS \| -1.21 \| 8.05E-12 \| 2.72E-11 \| \| ENSG00000074660 \| SCARF1 \| -1.41 \| 8.37E-12 \| 2.83E-11 \| \| ENSG00000187244 \| BCAM \| -1.25 \| 8.52E-12 \| 2.88E-11 \| \| ENSG00000134545 \| KLRC1 \| -5.88 \| 8.54E-12 \| 2.88E-11 \| \| ENSG00000111275 \| ALDH2 \| -3.17 \| 8.82E-12 \| 2.97E-11 \| \| ENSG00000244879 \|  \| 0.62 \| 8.82E-12 \| 2.97E-11 \| \| ENSG00000122585 \| NPY \| -3.85 \| 8.86E-12 \| 2.99E-11 \| \| ENSG00000172062 \| SMN1 \| 0.96 \| 9.13E-12 \| 3.07E-11 \| \| ENSG00000075131 \| TIPIN \| -1.01 \| 9.18E-12 \| 3.09E-11 \| \| ENSG00000138459 \| SLC35A5 \| 0.70 \| 9.19E-12 \| 3.09E-11 \| \| ENSG00000156787 \| TBC1D31 \| -0.89 \| 9.24E-12 \| 3.11E-11 \| \| ENSG00000226328 \| NUP50-DT \| 0.98 \| 9.39E-12 \| 3.16E-11 \| \| ENSG00000188603 \| CLN3 \| -0.62 \| 9.59E-12 \| 3.22E-11 \| \| ENSG00000102763 \| VWA8 \| -0.59 \| 9.78E-12 \| 3.28E-11 \| \| ENSG00000144445 \| KANSL1L \| 0.85 \| 9.93E-12 \| 3.33E-11 \| \| ENSG00000164100 \| NDST3 \| 2.87 \| 1.00E-11 \| 3.36E-11 \| \| ENSG00000123268 \| ATF1 \| 0.71 \| 1.04E-11 \| 3.48E-11 \| \| ENSG00000138670 \| RASGEF1B \| 0.61 \| 1.14E-11 \| 3.80E-11 \| \| ENSG00000070159 \| PTPN3 \| -2.05 \| 1.14E-11 \| 3.81E-11 \| \| ENSG00000154917 \| RAB6B \| -0.99 \| 1.16E-11 \| 3.86E-11 \| \| ENSG00000174028 \|  \| 1.03 \| 1.17E-11 \| 3.92E-11 \| \| ENSG00000164309 \| CMYA5 \| 0.88 \| 1.23E-11 \| 4.09E-11 \| \| ENSG00000020922 \| MRE11 \| -0.61 \| 1.28E-11 \| 4.27E-11 \| \| ENSG00000078053 \| AMPH \| 4.43 \| 1.34E-11 \| 4.47E-11 \| \| ENSG00000040608 \| RTN4R \| -1.71 \| 1.38E-11 \| 4.59E-11 \| \| ENSG00000141570 \| CBX8 \| -1.07 \| 1.38E-11 \| 4.60E-11 \| \| ENSG00000137745 \| MMP13 \| -3.06 \| 1.40E-11 \| 4.66E-11 \| \| ENSG00000106268 \| NUDT1 \| -0.82 \| 1.48E-11 \| 4.91E-11 \| \| ENSG00000106565 \| TMEM176B \| -2.54 \| 1.49E-11 \| 4.93E-11 \| \| ENSG00000121350 \| PYROXD1 \| 0.59 \| 1.50E-11 \| 4.97E-11 \| \| ENSG00000100479 \| POLE2 \| -1.24 \| 1.51E-11 \| 5.00E-11 \| \| ENSG00000162897 \| FCAMR \| -2.54 \| 1.54E-11 \| 5.11E-11 \| \| ENSG00000125629 \| INSIG2 \| 0.99 \| 1.55E-11 \| 5.15E-11 \| \| ENSG00000107872 \| FBXL15 \| -0.98 \| 1.60E-11 \| 5.31E-11 \| \| ENSG00000124587 \| PEX6 \| -0.60 \| 1.65E-11 \| 5.46E-11 \| \| ENSG00000243156 \| MICAL3 \| -0.61 \| 1.66E-11 \| 5.48E-11 \| \| ENSG00000260727 \|  \| 1.53 \| 1.68E-11 \| 5.55E-11 \| \| ENSG00000149927 \| DOC2A \| -1.84 \| 1.72E-11 \| 5.70E-11 \| \| ENSG00000254054 \|  \| -3.51 \| 1.82E-11 \| 6.02E-11 \| \| ENSG00000125375 \| DMAC2L \| 0.84 \| 1.84E-11 \| 6.08E-11 \| \| ENSG00000276644 \| DACH1 \| -1.81 \| 1.90E-11 \| 6.28E-11 \| \| ENSG00000163170 \| BOLA3 \| -0.64 \| 1.98E-11 \| 6.53E-11 \| \| ENSG00000118733 \| OLFM3 \| -8.06 \| 1.98E-11 \| 6.53E-11 \| \| ENSG00000244040 \| IL12A-AS1 \| -2.20 \| 2.02E-11 \| 6.65E-11 \| \| ENSG00000164308 \| ERAP2 \| -1.28 \| 2.03E-11 \| 6.69E-11 \| \| ENSG00000154710 \| RABGEF1 \| 1.55 \| 2.09E-11 \| 6.86E-11 \| \| ENSG00000161513 \| FDXR \| -2.09 \| 2.09E-11 \| 6.89E-11 \| \| ENSG00000134627 \| PIWIL4 \| -0.83 \| 2.14E-11 \| 7.02E-11 \| \| ENSG00000120915 \| EPHX2 \| -2.40 \| 2.14E-11 \| 7.03E-11 \| \| ENSG00000137944 \| KYAT3 \| 0.65 \| 2.17E-11 \| 7.12E-11 \| \| ENSG00000127329 \| PTPRB \| -2.63 \| 2.22E-11 \| 7.27E-11 \| \| ENSG00000127955 \| GNAI1 \| 0.84 \| 2.23E-11 \| 7.31E-11 \| \| ENSG00000121858 \| TNFSF10 \| -3.80 \| 2.27E-11 \| 7.45E-11 \| \| ENSG00000182010 \| RTKN2 \| -3.18 \| 2.29E-11 \| 7.49E-11 \| \| ENSG00000174628 \| IQCK \| 0.88 \| 2.29E-11 \| 7.50E-11 \| \| ENSG00000177425 \| PAWR \| -1.97 \| 2.29E-11 \| 7.51E-11 \| \| ENSG00000275395 \| FCGBP \| -0.60 \| 2.31E-11 \| 7.58E-11 \| \| ENSG00000135698 \| MPHOSPH6 \| 0.64 \| 2.38E-11 \| 7.78E-11 \| \| ENSG00000251442 \| LINC01094 \| -0.98 \| 2.38E-11 \| 7.78E-11 \| \| ENSG00000151729 \| SLC25A4 \| -2.24 \| 2.40E-11 \| 7.86E-11 \| \| ENSG00000196418 \| ZNF124 \| -0.69 \| 2.42E-11 \| 7.91E-11 \| \| ENSG00000144815 \| NXPE3 \| 0.83 \| 2.46E-11 \| 8.06E-11 \| \| ENSG00000137267 \| TUBB2A \| -1.29 \| 2.47E-11 \| 8.08E-11 \| \| ENSG00000143434 \| SEMA6C \| 0.93 \| 2.48E-11 \| 8.11E-11 \| \| ENSG00000177602 \| HASPIN \| -1.53 \| 2.51E-11 \| 8.19E-11 \| \| ENSG00000126603 \| GLIS2 \| 0.60 \| 2.64E-11 \| 8.63E-11 \| \| ENSG00000165644 \| COMTD1 \| -1.04 \| 2.78E-11 \| 9.08E-11 \| \| ENSG00000134574 \| DDB2 \| -0.80 \| 2.80E-11 \| 9.13E-11 \| \| ENSG00000181634 \| TNFSF15 \| 0.86 \| 2.84E-11 \| 9.27E-11 \| \| ENSG00000168334 \| XIRP1 \| 1.04 \| 2.91E-11 \| 9.48E-11 \| \| ENSG00000260868 \| LINC01960 \| 3.36 \| 2.92E-11 \| 9.51E-11 \| \| ENSG00000080603 \| SRCAP \| 0.70 \| 2.93E-11 \| 9.55E-11 \| \| ENSG00000112195 \| TREML2 \| -2.50 \| 2.96E-11 \| 9.63E-11 \| \| ENSG00000125124 \| BBS2 \| -0.96 \| 3.00E-11 \| 9.77E-11 \| \| ENSG00000155893 \| PXYLP1 \| 1.01 \| 3.00E-11 \| 9.78E-11 \| \| ENSG00000108342 \| CSF3 \| 5.78 \| 3.06E-11 \| 9.94E-11 \| \| ENSG00000100116 \| GCAT \| 1.44 \| 3.10E-11 \| 1.01E-10 \| \| ENSG00000116525 \| TRIM62 \| 0.81 \| 3.25E-11 \| 1.05E-10 \| \| ENSG00000197483 \| ZNF628 \| 0.66 \| 3.33E-11 \| 1.08E-10 \| \| ENSG00000182890 \| GLUD2 \| -1.09 \| 3.37E-11 \| 1.09E-10 \| \| ENSG00000186399 \| GOLGA8R \| -3.31 \| 3.39E-11 \| 1.10E-10 \| \| ENSG00000213462 \| ERV3-1 \| -0.64 \| 3.40E-11 \| 1.10E-10 \| \| ENSG00000156017 \| CARNMT1 \| 0.68 \| 3.45E-11 \| 1.12E-10 \| \| ENSG00000103522 \| IL21R \| 0.61 \| 3.57E-11 \| 1.16E-10 \| \| ENSG00000125885 \| MCM8 \| -1.08 \| 3.58E-11 \| 1.16E-10 \| \| ENSG00000126822 \| PLEKHG3 \| -1.13 \| 3.58E-11 \| 1.16E-10 \| \| ENSG00000154589 \| LY96 \| -1.06 \| 3.59E-11 \| 1.16E-10 \| \| ENSG00000261609 \| GAN \| 0.97 \| 3.60E-11 \| 1.16E-10 \| \| ENSG00000205856 \| C22orf42 \| 1.44 \| 3.63E-11 \| 1.17E-10 \| \| ENSG00000141448 \| GATA6 \| 2.62 \| 3.68E-11 \| 1.19E-10 \| \| ENSG00000132821 \| VSTM2L \| -1.24 \| 3.68E-11 \| 1.19E-10 \| \| ENSG00000163508 \| EOMES \| -1.23 \| 3.90E-11 \| 1.26E-10 \| \| ENSG00000196218 \| RYR1 \| -2.40 \| 4.04E-11 \| 1.30E-10 \| \| ENSG00000106976 \| DNM1 \| -0.71 \| 4.23E-11 \| 1.36E-10 \| \| ENSG00000128815 \| WDFY4 \| 0.74 \| 4.28E-11 \| 1.38E-10 \| \| ENSG00000242516 \| LINC00960 \| 0.90 \| 4.45E-11 \| 1.43E-10 \| \| ENSG00000164056 \| SPRY1 \| 2.13 \| 4.69E-11 \| 1.51E-10 \| \| ENSG00000166394 \| CYB5R2 \| 0.67 \| 4.74E-11 \| 1.52E-10 \| \| ENSG00000083454 \| P2RX5 \| -3.45 \| 4.75E-11 \| 1.53E-10 \| \| ENSG00000108187 \| PBLD \| -1.45 \| 4.81E-11 \| 1.55E-10 \| \| ENSG00000172985 \| SH3RF3 \| 2.80 \| 4.83E-11 \| 1.55E-10 \| \| ENSG00000267100 \| ILF3-DT \| 0.69 \| 4.95E-11 \| 1.59E-10 \| \| ENSG00000156869 \| FRRS1 \| -1.90 \| 5.18E-11 \| 1.66E-10 \| \| ENSG00000185614 \| INKA1 \| -1.12 \| 5.23E-11 \| 1.68E-10 \| \| ENSG00000267060 \| PTGES3L \| -1.49 \| 5.23E-11 \| 1.68E-10 \| \| ENSG00000164440 \| TXLNB \| -0.60 \| 5.33E-11 \| 1.71E-10 \| \| ENSG00000167034 \| NKX3-1 \| 1.95 \| 5.54E-11 \| 1.77E-10 \| \| ENSG00000114251 \| WNT5A \| 1.95 \| 5.56E-11 \| 1.78E-10 \| \| ENSG00000188211 \| NCR3LG1 \| -0.81 \| 5.56E-11 \| 1.78E-10 \| \| ENSG00000105255 \| FSD1 \| 6.85 \| 5.63E-11 \| 1.80E-10 \| \| ENSG00000171928 \| TVP23B \| 0.79 \| 5.89E-11 \| 1.88E-10 \| \| ENSG00000157741 \| UBN2 \| 0.69 \| 5.92E-11 \| 1.89E-10 \| \| ENSG00000112186 \| CAP2 \| -1.26 \| 6.25E-11 \| 1.99E-10 \| \| ENSG00000185499 \| MUC1 \| -1.06 \| 6.32E-11 \| 2.01E-10 \| \| ENSG00000136514 \| RTP4 \| -2.46 \| 6.50E-11 \| 2.07E-10 \| \| ENSG00000231672 \| DIRC3 \| -2.17 \| 7.20E-11 \| 2.29E-10 \| \| ENSG00000129467 \| ADCY4 \| -1.95 \| 7.23E-11 \| 2.30E-10 \| \| ENSG00000236438 \| FAM157A \| 3.44 \| 7.26E-11 \| 2.30E-10 \| \| ENSG00000239887 \| C1orf226 \| 0.78 \| 7.40E-11 \| 2.35E-10 \| \| ENSG00000157087 \| ATP2B2 \| 1.26 \| 7.95E-11 \| 2.52E-10 \| \| ENSG00000164182 \| NDUFAF2 \| 0.73 \| 7.97E-11 \| 2.52E-10 \| \| ENSG00000168427 \| KLHL30 \| -2.96 \| 7.97E-11 \| 2.52E-10 \| \| ENSG00000106560 \| GIMAP2 \| -1.07 \| 8.22E-11 \| 2.60E-10 \| \| ENSG00000103742 \| IGDCC4 \| 1.89 \| 8.25E-11 \| 2.61E-10 \| \| ENSG00000166582 \| CENPV \| -1.13 \| 8.26E-11 \| 2.61E-10 \| \| ENSG00000133818 \| RRAS2 \| 0.86 \| 8.54E-11 \| 2.70E-10 \| \| ENSG00000169291 \| SHE \| -1.35 \| 8.61E-11 \| 2.72E-10 \| \| ENSG00000166897 \| ELFN2 \| 2.42 \| 8.74E-11 \| 2.76E-10 \| \| ENSG00000197779 \| ZNF81 \| 0.76 \| 8.97E-11 \| 2.83E-10 \| \| ENSG00000116885 \| OSCP1 \| 1.44 \| 8.98E-11 \| 2.83E-10 \| \| ENSG00000169016 \| E2F6 \| 0.68 \| 8.99E-11 \| 2.84E-10 \| \| ENSG00000136243 \| NUPL2 \| -0.60 \| 9.37E-11 \| 2.95E-10 \| \| ENSG00000170909 \| OSCAR \| -0.85 \| 9.70E-11 \| 3.05E-10 \| \| ENSG00000183570 \| PCBP3 \| 1.24 \| 9.74E-11 \| 3.06E-10 \| \| ENSG00000227345 \| PARG \| -0.79 \| 9.78E-11 \| 3.08E-10 \| \| ENSG00000228709 \|  \| -4.14 \| 9.87E-11 \| 3.11E-10 \| \| ENSG00000119632 \| IFI27L2 \| -0.76 \| 9.98E-11 \| 3.14E-10 \| \| ENSG00000182050 \| MGAT4C \| 3.45 \| 1.00E-10 \| 3.15E-10 \| \| ENSG00000106571 \| GLI3 \| 3.48 \| 1.10E-10 \| 3.44E-10 \| \| ENSG00000095794 \| CREM \| -0.85 \| 1.10E-10 \| 3.46E-10 \| \| ENSG00000235890 \| TSPEAR-AS1 \| -2.81 \| 1.11E-10 \| 3.47E-10 \| \| ENSG00000135297 \| MTO1 \| -0.61 \| 1.14E-10 \| 3.57E-10 \| \| ENSG00000162910 \| MRPL55 \| -0.62 \| 1.17E-10 \| 3.66E-10 \| \| ENSG00000132016 \| C19orf57 \| -2.08 \| 1.17E-10 \| 3.67E-10 \| \| ENSG00000161653 \| NAGS \| 1.68 \| 1.19E-10 \| 3.71E-10 \| \| ENSG00000161647 \| MPP3 \| 0.94 \| 1.24E-10 \| 3.87E-10 \| \| ENSG00000123989 \| CHPF \| -0.60 \| 1.27E-10 \| 3.96E-10 \| \| ENSG00000163545 \| NUAK2 \| -2.76 \| 1.27E-10 \| 3.97E-10 \| \| ENSG00000167904 \| TMEM68 \| 0.62 \| 1.31E-10 \| 4.09E-10 \| \| ENSG00000134532 \| SOX5 \| 0.83 \| 1.35E-10 \| 4.20E-10 \| \| ENSG00000107954 \| NEURL1 \| -1.65 \| 1.35E-10 \| 4.22E-10 \| \| ENSG00000124217 \| MOCS3 \| 0.70 \| 1.36E-10 \| 4.23E-10 \| \| ENSG00000144647 \| POMGNT2 \| -0.77 \| 1.54E-10 \| 4.80E-10 \| \| ENSG00000160219 \| GAB3 \| -0.98 \| 1.54E-10 \| 4.80E-10 \| \| ENSG00000107105 \| ELAVL2 \| 1.03 \| 1.54E-10 \| 4.81E-10 \| \| ENSG00000281103 \| TRG-AS1 \| -2.22 \| 1.55E-10 \| 4.83E-10 \| \| ENSG00000137473 \| TTC29 \| 1.74 \| 1.57E-10 \| 4.90E-10 \| \| ENSG00000248971 \|  \| 1.28 \| 1.60E-10 \| 4.97E-10 \| \| ENSG00000247626 \| MARS2 \| 0.74 \| 1.63E-10 \| 5.05E-10 \| \| ENSG00000182685 \| BRICD5 \| -1.34 \| 1.63E-10 \| 5.06E-10 \| \| ENSG00000232004 \|  \| -0.76 \| 1.64E-10 \| 5.08E-10 \| \| ENSG00000114735 \| HEMK1 \| -0.87 \| 1.64E-10 \| 5.10E-10 \| \| ENSG00000138944 \| SHISAL1 \| 2.12 \| 1.65E-10 \| 5.14E-10 \| \| ENSG00000095917 \| TPSD1 \| -3.50 \| 1.68E-10 \| 5.23E-10 \| \| ENSG00000141384 \| TAF4B \| 0.72 \| 1.69E-10 \| 5.25E-10 \| \| ENSG00000122592 \| HOXA7 \| -1.32 \| 1.78E-10 \| 5.51E-10 \| \| ENSG00000205213 \| LGR4 \| 1.02 \| 1.89E-10 \| 5.86E-10 \| \| ENSG00000128944 \| KNSTRN \| -0.68 \| 1.91E-10 \| 5.91E-10 \| \| ENSG00000122025 \| FLT3 \| -0.93 \| 1.95E-10 \| 6.03E-10 \| \| ENSG00000145864 \| GABRB2 \| -2.19 \| 2.02E-10 \| 6.23E-10 \| \| ENSG00000167578 \| RAB4B \| 0.78 \| 2.03E-10 \| 6.27E-10 \| \| ENSG00000113231 \| PDE8B \| 2.26 \| 2.07E-10 \| 6.40E-10 \| \| ENSG00000279010 \|  \| 1.30 \| 2.18E-10 \| 6.71E-10 \| \| ENSG00000188848 \| BEND4 \| -2.71 \| 2.29E-10 \| 7.04E-10 \| \| ENSG00000116141 \| MARK1 \| 2.01 \| 2.29E-10 \| 7.05E-10 \| \| ENSG00000267751 \| LOC105372233 \| -1.55 \| 2.37E-10 \| 7.28E-10 \| \| ENSG00000100916 \| BRMS1L \| 0.88 \| 2.37E-10 \| 7.29E-10 \| \| ENSG00000066926 \| FECH \| -0.62 \| 2.41E-10 \| 7.40E-10 \| \| ENSG00000174607 \| UGT8 \| -1.43 \| 2.43E-10 \| 7.47E-10 \| \| ENSG00000282508 \| LINC01002 \| 1.60 \| 2.45E-10 \| 7.53E-10 \| \| ENSG00000082512 \| TRAF5 \| -3.02 \| 2.50E-10 \| 7.68E-10 \| \| ENSG00000180155 \| LYNX1 \| -1.98 \| 2.55E-10 \| 7.82E-10 \| \| ENSG00000113594 \| LIFR \| 4.97 \| 2.57E-10 \| 7.88E-10 \| \| ENSG00000173926 \| 03-Mar \| 1.19 \| 2.58E-10 \| 7.90E-10 \| \| ENSG00000245248 \| USP2-AS1 \| 0.99 \| 2.59E-10 \| 7.93E-10 \| \| ENSG00000116833 \| NR5A2 \| -0.81 \| 2.61E-10 \| 7.99E-10 \| \| ENSG00000237190 \| CDKN2AIPNL \| -0.92 \| 2.66E-10 \| 8.15E-10 \| \| ENSG00000233030 \|  \| 2.49 \| 2.67E-10 \| 8.19E-10 \| \| ENSG00000135482 \| ZC3H10 \| 0.70 \| 2.74E-10 \| 8.38E-10 \| \| ENSG00000187720 \| THSD4 \| 0.83 \| 2.76E-10 \| 8.44E-10 \| \| ENSG00000106789 \| CORO2A \| -0.68 \| 2.82E-10 \| 8.64E-10 \| \| ENSG00000186871 \| ERCC6L \| -2.50 \| 2.99E-10 \| 9.15E-10 \| \| ENSG00000102384 \| CENPI \| -1.73 \| 3.01E-10 \| 9.20E-10 \| \| ENSG00000272502 \|  \| -0.82 \| 3.03E-10 \| 9.26E-10 \| \| ENSG00000148019 \| CEP78 \| -0.74 \| 3.07E-10 \| 9.37E-10 \| \| ENSG00000103995 \| CEP152 \| -0.86 \| 3.15E-10 \| 9.62E-10 \| \| ENSG00000135069 \| PSAT1 \| -1.37 \| 3.16E-10 \| 9.65E-10 \| \| ENSG00000131037 \| EPS8L1 \| 0.64 \| 3.18E-10 \| 9.69E-10 \| \| ENSG00000117543 \| DPH5 \| -0.67 \| 3.21E-10 \| 9.80E-10 \| \| ENSG00000183098 \| GPC6 \| 0.64 \| 3.23E-10 \| 9.85E-10 \| \| ENSG00000134253 \| TRIM45 \| -3.29 \| 3.24E-10 \| 9.86E-10 \| \| ENSG00000143315 \| PIGM \| -0.59 \| 3.27E-10 \| 9.96E-10 \| \| ENSG00000169218 \| RSPO1 \| 1.34 \| 3.37E-10 \| 1.03E-09 \| \| ENSG00000121807 \| CCR2 \| -5.43 \| 3.41E-10 \| 1.04E-09 \| \| ENSG00000013619 \| MAMLD1 \| 0.71 \| 3.45E-10 \| 1.05E-09 \| \| ENSG00000168077 \| SCARA3 \| -4.81 \| 3.57E-10 \| 1.08E-09 \| \| ENSG00000197893 \| NRAP \| 0.81 \| 3.59E-10 \| 1.09E-09 \| \| ENSG00000182379 \| NXPH4 \| -1.91 \| 3.67E-10 \| 1.12E-09 \| \| ENSG00000163347 \| CLDN1 \| 1.00 \| 3.67E-10 \| 1.12E-09 \| \| ENSG00000104450 \| SPAG1 \| -0.75 \| 3.69E-10 \| 1.12E-09 \| \| ENSG00000273669 \|  \| -3.30 \| 3.69E-10 \| 1.12E-09 \| \| ENSG00000243943 \| ZNF512 \| -0.72 \| 3.74E-10 \| 1.14E-09 \| \| ENSG00000187730 \| GABRD \| -4.81 \| 3.84E-10 \| 1.16E-09 \| \| ENSG00000115255 \| REEP6 \| -0.85 \| 3.93E-10 \| 1.19E-09 \| \| ENSG00000118507 \| AKAP7 \| -1.61 \| 3.97E-10 \| 1.20E-09 \| \| ENSG00000184939 \| ZFP90 \| -0.70 \| 4.02E-10 \| 1.22E-09 \| \| ENSG00000225684 \| FAM225B \| 1.29 \| 4.08E-10 \| 1.24E-09 \| \| ENSG00000158859 \| ADAMTS4 \| -2.11 \| 4.09E-10 \| 1.24E-09 \| \| ENSG00000109084 \| TMEM97 \| -0.65 \| 4.20E-10 \| 1.27E-09 \| \| ENSG00000158856 \| DMTN \| -4.04 \| 4.28E-10 \| 1.29E-09 \| \| ENSG00000272767 \| JMJD1C-AS1 \| -0.94 \| 4.36E-10 \| 1.32E-09 \| \| ENSG00000077935 \| SMC1B \| -3.04 \| 4.39E-10 \| 1.33E-09 \| \| ENSG00000140451 \| PIF1 \| -4.03 \| 4.39E-10 \| 1.33E-09 \| \| ENSG00000157470 \| FAM81A \| 0.68 \| 4.68E-10 \| 1.41E-09 \| \| ENSG00000234964 \|  \| -1.39 \| 4.69E-10 \| 1.42E-09 \| \| ENSG00000169860 \| P2RY1 \| -1.30 \| 4.70E-10 \| 1.42E-09 \| \| ENSG00000243364 \| EFNA4 \| -1.23 \| 4.81E-10 \| 1.45E-09 \| \| ENSG00000154079 \| SDHAF4 \| -0.62 \| 4.85E-10 \| 1.46E-09 \| \| ENSG00000153956 \| CACNA2D1 \| 1.44 \| 4.86E-10 \| 1.46E-09 \| \| ENSG00000104881 \| PPP1R13L \| 0.88 \| 4.88E-10 \| 1.47E-09 \| \| ENSG00000198774 \| RASSF9 \| 1.98 \| 4.99E-10 \| 1.50E-09 \| \| ENSG00000148426 \| PROSER2 \| -1.28 \| 5.04E-10 \| 1.52E-09 \| \| ENSG00000151470 \| C4orf33 \| -1.10 \| 5.06E-10 \| 1.52E-09 \| \| ENSG00000075188 \| NUP37 \| -0.82 \| 5.09E-10 \| 1.53E-09 \| \| ENSG00000140470 \| ADAMTS17 \| -0.73 \| 5.09E-10 \| 1.53E-09 \| \| ENSG00000141622 \| RNF165 \| 2.13 \| 5.11E-10 \| 1.54E-09 \| \| ENSG00000213366 \| GSTM2 \| -2.39 \| 5.15E-10 \| 1.55E-09 \| \| ENSG00000239213 \| NCK1-DT \| -1.08 \| 5.21E-10 \| 1.57E-09 \| \| ENSG00000106524 \| ANKMY2 \| -0.76 \| 5.27E-10 \| 1.58E-09 \| \| ENSG00000180694 \| TMEM64 \| 0.75 \| 5.27E-10 \| 1.58E-09 \| \| ENSG00000163701 \| IL17RE \| -1.74 \| 5.32E-10 \| 1.60E-09 \| \| ENSG00000170915 \| PAQR8 \| 0.98 \| 5.47E-10 \| 1.64E-09 \| \| ENSG00000099715 \| PCDH11Y \| 3.01 \| 5.76E-10 \| 1.73E-09 \| \| ENSG00000141542 \| RAB40B \| -0.85 \| 5.86E-10 \| 1.76E-09 \| \| ENSG00000111058 \| ACSS3 \| -1.48 \| 5.89E-10 \| 1.77E-09 \| \| ENSG00000167393 \| PPP2R3B \| -0.90 \| 5.91E-10 \| 1.77E-09 \| \| ENSG00000164694 \| FNDC1 \| 2.56 \| 6.09E-10 \| 1.82E-09 \| \| ENSG00000241749 \| RPSAP52 \| 3.42 \| 6.19E-10 \| 1.85E-09 \| \| ENSG00000102678 \| FGF9 \| 2.20 \| 6.29E-10 \| 1.88E-09 \| \| ENSG00000185633 \| NDUFA4L2 \| -1.53 \| 6.33E-10 \| 1.89E-09 \| \| ENSG00000274736 \| CCL23 \| -2.28 \| 6.46E-10 \| 1.93E-09 \| \| ENSG00000110002 \| VWA5A \| -0.82 \| 6.86E-10 \| 2.05E-09 \| \| ENSG00000166002 \| SMCO4 \| -0.65 \| 7.04E-10 \| 2.10E-09 \| \| ENSG00000213225 \|  \| -2.44 \| 7.08E-10 \| 2.11E-09 \| \| ENSG00000137225 \| CAPN11 \| -3.15 \| 7.29E-10 \| 2.17E-09 \| \| ENSG00000175874 \| CREG2 \| -1.33 \| 7.29E-10 \| 2.18E-09 \| \| ENSG00000188015 \| S100A3 \| -1.17 \| 7.32E-10 \| 2.18E-09 \| \| ENSG00000107951 \| MTPAP \| -0.80 \| 7.66E-10 \| 2.28E-09 \| \| ENSG00000138621 \| PPCDC \| -0.64 \| 7.84E-10 \| 2.34E-09 \| \| ENSG00000099617 \| EFNA2 \| -1.77 \| 8.03E-10 \| 2.39E-09 \| \| ENSG00000127481 \| UBR4 \| 0.67 \| 8.12E-10 \| 2.42E-09 \| \| ENSG00000183137 \| CEP57L1 \| -0.80 \| 8.38E-10 \| 2.49E-09 \| \| ENSG00000134363 \| FST \| 2.20 \| 8.60E-10 \| 2.56E-09 \| \| ENSG00000240771 \| ARHGEF25 \| 1.01 \| 8.62E-10 \| 2.56E-09 \| \| ENSG00000171612 \| SLC25A33 \| 0.65 \| 8.74E-10 \| 2.60E-09 \| \| ENSG00000159433 \| STARD9 \| -0.77 \| 8.75E-10 \| 2.60E-09 \| \| ENSG00000232931 \|  \| -3.38 \| 8.80E-10 \| 2.61E-09 \| \| ENSG00000100749 \| VRK1 \| -0.66 \| 8.81E-10 \| 2.62E-09 \| \| ENSG00000276070 \| CCL4L2 \| 1.46 \| 9.05E-10 \| 2.69E-09 \| \| ENSG00000276070 \| CCL4L1 \| 1.46 \| 9.05E-10 \| 2.69E-09 \| \| ENSG00000060656 \| PTPRU \| 1.28 \| 9.19E-10 \| 2.73E-09 \| \| ENSG00000166923 \| GREM1 \| 2.56 \| 9.38E-10 \| 2.78E-09 \| \| ENSG00000139178 \| C1RL \| -0.99 \| 9.98E-10 \| 2.95E-09 \| \| ENSG00000119865 \| CNRIP1 \| 0.59 \| 1.03E-09 \| 3.05E-09 \| \| ENSG00000224531 \| SMIM13 \| 0.66 \| 1.06E-09 \| 3.14E-09 \| \| ENSG00000229334 \|  \| -1.32 \| 1.09E-09 \| 3.23E-09 \| \| ENSG00000169548 \| ZNF280A \| -4.77 \| 1.10E-09 \| 3.25E-09 \| \| ENSG00000130762 \| ARHGEF16 \| -1.47 \| 1.13E-09 \| 3.34E-09 \| \| ENSG00000196329 \| GIMAP5 \| 1.34 \| 1.14E-09 \| 3.37E-09 \| \| ENSG00000101639 \| CEP192 \| -0.59 \| 1.15E-09 \| 3.39E-09 \| \| ENSG00000161544 \| CYGB \| 3.20 \| 1.15E-09 \| 3.39E-09 \| \| ENSG00000255197 \|  \| 0.63 \| 1.16E-09 \| 3.41E-09 \| \| ENSG00000103710 \| RASL12 \| 3.30 \| 1.18E-09 \| 3.48E-09 \| \| ENSG00000141401 \| IMPA2 \| 0.66 \| 1.19E-09 \| 3.51E-09 \| \| ENSG00000205517 \| RGL3 \| -2.56 \| 1.20E-09 \| 3.53E-09 \| \| ENSG00000182809 \| CRIP2 \| -1.13 \| 1.23E-09 \| 3.62E-09 \| \| ENSG00000276975 \|  \| -2.62 \| 1.26E-09 \| 3.71E-09 \| \| ENSG00000104814 \| MAP4K1 \| -2.26 \| 1.26E-09 \| 3.72E-09 \| \| ENSG00000198417 \| MT1F \| 1.51 \| 1.33E-09 \| 3.90E-09 \| \| ENSG00000162745 \| OLFML2B \| -0.85 \| 1.33E-09 \| 3.90E-09 \| \| ENSG00000185760 \| KCNQ5 \| -2.77 \| 1.36E-09 \| 4.01E-09 \| \| ENSG00000099284 \| H2AFY2 \| -1.03 \| 1.37E-09 \| 4.01E-09 \| \| ENSG00000105516 \| DBP \| -1.40 \| 1.37E-09 \| 4.03E-09 \| \| ENSG00000187391 \| MAGI2 \| 1.13 \| 1.39E-09 \| 4.08E-09 \| \| ENSG00000205220 \| PSMB10 \| -0.66 \| 1.42E-09 \| 4.15E-09 \| \| ENSG00000107554 \| DNMBP \| -0.80 \| 1.42E-09 \| 4.17E-09 \| \| ENSG00000136695 \| IL36RN \| 3.94 \| 1.44E-09 \| 4.22E-09 \| \| ENSG00000185480 \| PARPBP \| -1.45 \| 1.45E-09 \| 4.24E-09 \| \| ENSG00000083290 \| ULK2 \| -1.29 \| 1.48E-09 \| 4.33E-09 \| \| ENSG00000070501 \| POLB \| -0.86 \| 1.49E-09 \| 4.36E-09 \| \| ENSG00000110799 \| VWF \| -1.24 \| 1.49E-09 \| 4.36E-09 \| \| ENSG00000113749 \| HRH2 \| 0.68 \| 1.55E-09 \| 4.53E-09 \| \| ENSG00000135074 \| ADAM19 \| -0.69 \| 1.56E-09 \| 4.54E-09 \| \| ENSG00000136114 \| THSD1 \| -2.35 \| 1.56E-09 \| 4.57E-09 \| \| ENSG00000124875 \| CXCL6 \| 1.69 \| 1.57E-09 \| 4.59E-09 \| \| ENSG00000255737 \| AGAP2-AS1 \| 0.64 \| 1.60E-09 \| 4.67E-09 \| \| ENSG00000250959 \| GLUD1P3 \| -0.92 \| 1.61E-09 \| 4.70E-09 \| \| ENSG00000273136 \| NBPF26 \| 0.83 \| 1.62E-09 \| 4.72E-09 \| \| ENSG00000273136 \| NOTCH2NLR \| 0.83 \| 1.62E-09 \| 4.72E-09 \| \| ENSG00000231609 \| LOC100132215 \| -1.45 \| 1.63E-09 \| 4.74E-09 \| \| ENSG00000154217 \| PITPNC1 \| -0.80 \| 1.63E-09 \| 4.76E-09 \| \| ENSG00000136250 \| AOAH \| -2.27 \| 1.67E-09 \| 4.85E-09 \| \| ENSG00000185716 \| MOSMO \| 0.70 \| 1.69E-09 \| 4.92E-09 \| \| ENSG00000126106 \| TMEM53 \| 0.72 \| 1.72E-09 \| 5.01E-09 \| \| ENSG00000223508 \|  \| 1.19 \| 1.76E-09 \| 5.12E-09 \| \| ENSG00000167914 \| GSDMA \| -3.07 \| 1.78E-09 \| 5.17E-09 \| \| ENSG00000197077 \| KIAA1671 \| -2.11 \| 1.78E-09 \| 5.17E-09 \| \| ENSG00000218565 \|  \| 1.23 \| 1.79E-09 \| 5.21E-09 \| \| ENSG00000174206 \| C12orf66 \| 0.77 \| 1.92E-09 \| 5.58E-09 \| \| ENSG00000172167 \| MTBP \| -1.13 \| 1.93E-09 \| 5.60E-09 \| \| ENSG00000126070 \| AGO3 \| 0.62 \| 1.94E-09 \| 5.62E-09 \| \| ENSG00000100036 \| SLC35E4 \| 0.62 \| 1.96E-09 \| 5.69E-09 \| \| ENSG00000099937 \| SERPIND1 \| 3.20 \| 1.97E-09 \| 5.70E-09 \| \| ENSG00000250067 \| YJEFN3 \| -0.86 \| 1.99E-09 \| 5.76E-09 \| \| ENSG00000125434 \| SLC25A35 \| -1.59 \| 2.06E-09 \| 5.97E-09 \| \| ENSG00000162882 \| HAAO \| -0.82 \| 2.08E-09 \| 6.02E-09 \| \| ENSG00000181790 \| ADGRB1 \| -0.82 \| 2.10E-09 \| 6.07E-09 \| \| ENSG00000166352 \| C11orf74 \| -1.48 \| 2.15E-09 \| 6.23E-09 \| \| ENSG00000116544 \| DLGAP3 \| 2.13 \| 2.16E-09 \| 6.24E-09 \| \| ENSG00000073861 \| TBX21 \| 1.52 \| 2.20E-09 \| 6.38E-09 \| \| ENSG00000177519 \| RPRM \| -6.24 \| 2.23E-09 \| 6.44E-09 \| \| ENSG00000170955 \| CAVIN3 \| 1.00 \| 2.23E-09 \| 6.44E-09 \| \| ENSG00000187091 \| PLCD1 \| -1.57 \| 2.27E-09 \| 6.54E-09 \| \| ENSG00000183784 \| C9orf66 \| -1.85 \| 2.31E-09 \| 6.66E-09 \| \| ENSG00000113790 \| EHHADH \| -1.18 \| 2.50E-09 \| 7.22E-09 \| \| ENSG00000177738 \| LOC648987 \| -1.07 \| 2.51E-09 \| 7.25E-09 \| \| ENSG00000108515 \| ENO3 \| -1.02 \| 2.60E-09 \| 7.50E-09 \| \| ENSG00000139618 \| BRCA2 \| -0.85 \| 2.62E-09 \| 7.53E-09 \| \| ENSG00000167748 \| KLK1 \| -2.58 \| 2.62E-09 \| 7.53E-09 \| \| ENSG00000233308 \| OSTN-AS1 \| 4.72 \| 2.66E-09 \| 7.65E-09 \| \| ENSG00000185052 \| SLC24A3 \| 1.39 \| 2.66E-09 \| 7.66E-09 \| \| ENSG00000182048 \| TRPC2 \| -1.63 \| 2.72E-09 \| 7.82E-09 \| \| ENSG00000181004 \| BBS12 \| 1.27 \| 2.74E-09 \| 7.88E-09 \| \| ENSG00000180071 \| ANKRD18A \| -1.66 \| 2.75E-09 \| 7.89E-09 \| \| ENSG00000186831 \| KRT17P2 \| -7.23 \| 2.89E-09 \| 8.30E-09 \| \| ENSG00000143674 \| MAP3K21 \| 0.75 \| 2.90E-09 \| 8.32E-09 \| \| ENSG00000128311 \| TST \| -0.81 \| 2.92E-09 \| 8.39E-09 \| \| ENSG00000250722 \| SELENOP \| -5.20 \| 2.98E-09 \| 8.54E-09 \| \| ENSG00000148120 \| C9orf3 \| -1.34 \| 2.98E-09 \| 8.54E-09 \| \| ENSG00000157927 \| RADIL \| -1.24 \| 3.09E-09 \| 8.85E-09 \| \| ENSG00000185842 \| DNAH14 \| -1.37 \| 3.10E-09 \| 8.88E-09 \| \| ENSG00000132514 \| CLEC10A \| -2.63 \| 3.10E-09 \| 8.88E-09 \| \| ENSG00000261416 \|  \| -1.08 \| 3.11E-09 \| 8.91E-09 \| \| ENSG00000132481 \| TRIM47 \| -0.65 \| 3.30E-09 \| 9.42E-09 \| \| ENSG00000077063 \| CTTNBP2 \| 1.68 \| 3.33E-09 \| 9.51E-09 \| \| ENSG00000120327 \| PCDHB14 \| -3.66 \| 3.33E-09 \| 9.52E-09 \| \| ENSG00000152240 \| HAUS1 \| -0.75 \| 3.38E-09 \| 9.63E-09 \| \| ENSG00000131885 \|  \| -6.19 \| 3.45E-09 \| 9.84E-09 \| \| ENSG00000234771 \| SLC25A25-AS1 \| -0.91 \| 3.46E-09 \| 9.86E-09 \| \| ENSG00000205208 \| C4orf46 \| -0.71 \| 3.62E-09 \| 1.03E-08 \| \| ENSG00000246100 \| LINC00900 \| -1.07 \| 3.67E-09 \| 1.05E-08 \| \| ENSG00000197301 \| LOC100129940 \| -2.60 \| 3.71E-09 \| 1.06E-08 \| \| ENSG00000156398 \| SFXN2 \| -1.00 \| 3.72E-09 \| 1.06E-08 \| \| ENSG00000072952 \| MRVI1 \| -3.63 \| 3.88E-09 \| 1.10E-08 \| \| ENSG00000175175 \| PPM1E \| -0.76 \| 3.92E-09 \| 1.11E-08 \| \| ENSG00000042317 \| SPATA7 \| -1.32 \| 4.05E-09 \| 1.15E-08 \| \| ENSG00000118600 \| RXYLT1 \| 0.62 \| 4.11E-09 \| 1.17E-08 \| \| ENSG00000272106 \|  \| 0.83 \| 4.21E-09 \| 1.20E-08 \| \| ENSG00000151151 \| IPMK \| 0.59 \| 4.28E-09 \| 1.21E-08 \| \| ENSG00000268518 \|  \| -0.88 \| 4.36E-09 \| 1.24E-08 \| \| ENSG00000181690 \| PLAG1 \| 0.96 \| 4.37E-09 \| 1.24E-08 \| \| ENSG00000182489 \| XKRX \| -1.33 \| 4.49E-09 \| 1.27E-08 \| \| ENSG00000197128 \| ZNF772 \| -1.26 \| 4.75E-09 \| 1.35E-08 \| \| ENSG00000228109 \| MELTF-AS1 \| -2.04 \| 4.81E-09 \| 1.36E-08 \| \| ENSG00000069011 \| PITX1 \| -1.56 \| 5.06E-09 \| 1.43E-08 \| \| ENSG00000105290 \| APLP1 \| 0.93 \| 5.13E-09 \| 1.45E-08 \| \| ENSG00000230724 \|  \| 1.67 \| 5.22E-09 \| 1.48E-08 \| \| ENSG00000167771 \| RCOR2 \| -2.28 \| 5.29E-09 \| 1.50E-08 \| \| ENSG00000157353 \| FUK \| -0.70 \| 5.36E-09 \| 1.52E-08 \| \| ENSG00000105137 \| SYDE1 \| 0.99 \| 5.41E-09 \| 1.53E-08 \| \| ENSG00000182132 \| KCNIP1 \| -2.02 \| 5.44E-09 \| 1.54E-08 \| \| ENSG00000177181 \| RIMKLA \| -2.27 \| 5.51E-09 \| 1.56E-08 \| \| ENSG00000129151 \| BBOX1 \| -6.09 \| 5.54E-09 \| 1.56E-08 \| \| ENSG00000063601 \| MTMR1 \| -0.60 \| 5.65E-09 \| 1.60E-08 \| \| ENSG00000254681 \| PKD1P5-LOC105376752 \| 0.60 \| 5.69E-09 \| 1.61E-08 \| \| ENSG00000226125 \| LINC01907 \| -3.39 \| 5.80E-09 \| 1.64E-08 \| \| ENSG00000182575 \| NXPH3 \| -1.33 \| 5.81E-09 \| 1.64E-08 \| \| ENSG00000105072 \| C19orf44 \| 0.84 \| 6.05E-09 \| 1.70E-08 \| \| ENSG00000031691 \| CENPQ \| -0.89 \| 6.06E-09 \| 1.71E-08 \| \| ENSG00000152749 \| GPR180 \| 0.64 \| 6.09E-09 \| 1.71E-08 \| \| ENSG00000179388 \| EGR3 \| 1.20 \| 6.11E-09 \| 1.72E-08 \| \| ENSG00000162139 \| NEU3 \| -0.92 \| 6.26E-09 \| 1.76E-08 \| \| ENSG00000106236 \| NPTX2 \| -3.13 \| 6.27E-09 \| 1.76E-08 \| \| ENSG00000142405 \| NLRP12 \| -2.07 \| 6.29E-09 \| 1.77E-08 \| \| ENSG00000214595 \| EML6 \| 1.99 \| 6.77E-09 \| 1.90E-08 \| \| ENSG00000231811 \|  \| 2.42 \| 6.97E-09 \| 1.96E-08 \| \| ENSG00000145217 \| SLC26A1 \| 1.75 \| 7.21E-09 \| 2.02E-08 \| \| ENSG00000137714 \| FDX1 \| -0.71 \| 7.36E-09 \| 2.06E-08 \| \| ENSG00000011083 \| SLC6A7 \| 2.75 \| 7.48E-09 \| 2.09E-08 \| \| ENSG00000143167 \| GPA33 \| -2.52 \| 7.52E-09 \| 2.10E-08 \| \| ENSG00000261504 \| LINC01686 \| 1.03 \| 7.63E-09 \| 2.13E-08 \| \| ENSG00000186106 \| ANKRD46 \| -0.74 \| 7.73E-09 \| 2.16E-08 \| \| ENSG00000112276 \| BVES \| -0.68 \| 7.83E-09 \| 2.19E-08 \| \| ENSG00000215447 \|  \| -0.86 \| 8.12E-09 \| 2.27E-08 \| \| ENSG00000066651 \| TRMT11 \| -0.75 \| 8.14E-09 \| 2.27E-08 \| \| ENSG00000185522 \| LMNTD2 \| -0.77 \| 8.19E-09 \| 2.28E-08 \| \| ENSG00000112559 \| MDFI \| -3.42 \| 8.34E-09 \| 2.33E-08 \| \| ENSG00000099889 \| ARVCF \| -1.32 \| 8.58E-09 \| 2.39E-08 \| \| ENSG00000197905 \| TEAD4 \| 0.83 \| 8.69E-09 \| 2.42E-08 \| \| ENSG00000137142 \| IGFBPL1 \| -3.09 \| 8.71E-09 \| 2.43E-08 \| \| ENSG00000182552 \| RWDD4 \| -0.63 \| 8.77E-09 \| 2.44E-08 \| \| ENSG00000167604 \| NFKBID \| 0.73 \| 8.83E-09 \| 2.46E-08 \| \| ENSG00000163702 \| IL17RC \| -2.76 \| 8.86E-09 \| 2.47E-08 \| \| ENSG00000101311 \| FERMT1 \| 1.25 \| 8.91E-09 \| 2.48E-08 \| \| ENSG00000140323 \| DISP2 \| -1.47 \| 9.03E-09 \| 2.51E-08 \| \| ENSG00000245904 \| LOC101928617 \| 0.64 \| 9.19E-09 \| 2.55E-08 \| \| ENSG00000121621 \| KIF18A \| -1.12 \| 9.24E-09 \| 2.57E-08 \| \| ENSG00000262580 \|  \| -1.52 \| 9.29E-09 \| 2.58E-08 \| \| ENSG00000116981 \| NT5C1A \| -5.04 \| 9.41E-09 \| 2.61E-08 \| \| ENSG00000169413 \| RNASE6 \| -1.29 \| 9.45E-09 \| 2.62E-08 \| \| ENSG00000255422 \| LOC105369519 \| -3.81 \| 9.51E-09 \| 2.64E-08 \| \| ENSG00000088305 \| DNMT3B \| -1.43 \| 9.62E-09 \| 2.67E-08 \| \| ENSG00000176659 \| C20orf197 \| -2.08 \| 9.75E-09 \| 2.71E-08 \| \| ENSG00000274536 \|  \| -1.40 \| 9.91E-09 \| 2.75E-08 \| \| ENSG00000183340 \| JRKL \| 0.68 \| 9.92E-09 \| 2.75E-08 \| \| ENSG00000183605 \| SFXN4 \| -0.79 \| 9.93E-09 \| 2.75E-08 \| \| ENSG00000260139 \|  \| 0.63 \| 1.04E-08 \| 2.88E-08 \| \| ENSG00000185347 \| TEDC1 \| -0.72 \| 1.05E-08 \| 2.90E-08 \| \| ENSG00000272899 \| ATP6V1FNB \| 1.28 \| 1.06E-08 \| 2.94E-08 \| \| ENSG00000154188 \| ANGPT1 \| -7.00 \| 1.08E-08 \| 2.99E-08 \| \| ENSG00000003436 \| TFPI \| -1.46 \| 1.12E-08 \| 3.09E-08 \| \| ENSG00000099953 \| MMP11 \| -3.81 \| 1.12E-08 \| 3.10E-08 \| \| ENSG00000087237 \| CETP \| 3.84 \| 1.13E-08 \| 3.11E-08 \| \| ENSG00000046647 \| GEMIN8 \| 0.69 \| 1.13E-08 \| 3.11E-08 \| \| ENSG00000109944 \| JHY \| -1.49 \| 1.17E-08 \| 3.23E-08 \| \| ENSG00000076351 \| SLC46A1 \| -0.72 \| 1.22E-08 \| 3.35E-08 \| \| ENSG00000205485 \| LOC100133091 \| 0.95 \| 1.22E-08 \| 3.36E-08 \| \| ENSG00000188620 \| HMX3 \| -1.54 \| 1.24E-08 \| 3.40E-08 \| \| ENSG00000249816 \| LINC00964 \| 1.45 \| 1.25E-08 \| 3.45E-08 \| \| ENSG00000197603 \| CPLANE1 \| -0.84 \| 1.26E-08 \| 3.45E-08 \| \| ENSG00000223855 \| HRAT92 \| -0.84 \| 1.26E-08 \| 3.46E-08 \| \| ENSG00000242282 \|  \| -1.38 \| 1.26E-08 \| 3.48E-08 \| \| ENSG00000107960 \| STN1 \| -0.77 \| 1.27E-08 \| 3.49E-08 \| \| ENSG00000164038 \| SLC9B2 \| 0.61 \| 1.29E-08 \| 3.54E-08 \| \| ENSG00000179344 \| HLA-DQB1 \| -2.47 \| 1.34E-08 \| 3.67E-08 \| \| ENSG00000268686 \| LOC101928295 \| -1.17 \| 1.34E-08 \| 3.68E-08 \| \| ENSG00000137078 \| SIT1 \| -1.18 \| 1.36E-08 \| 3.74E-08 \| \| ENSG00000196693 \| ZNF33B \| -1.29 \| 1.37E-08 \| 3.75E-08 \| \| ENSG00000110077 \| MS4A6A \| -6.84 \| 1.37E-08 \| 3.75E-08 \| \| ENSG00000105697 \| HAMP \| -2.09 \| 1.37E-08 \| 3.76E-08 \| \| ENSG00000151617 \| EDNRA \| -1.30 \| 1.38E-08 \| 3.78E-08 \| \| ENSG00000154358 \| OBSCN \| -0.90 \| 1.40E-08 \| 3.83E-08 \| \| ENSG00000185168 \| LINC00482 \| -1.39 \| 1.43E-08 \| 3.90E-08 \| \| ENSG00000226742 \| HSBP1L1 \| -1.64 \| 1.43E-08 \| 3.91E-08 \| \| ENSG00000184785 \| SMIM10 \| -1.38 \| 1.43E-08 \| 3.92E-08 \| \| ENSG00000197989 \| SNHG12 \| -0.63 \| 1.44E-08 \| 3.94E-08 \| \| ENSG00000226822 \|  \| 1.02 \| 1.52E-08 \| 4.16E-08 \| \| ENSG00000136848 \| DAB2IP \| -1.85 \| 1.53E-08 \| 4.17E-08 \| \| ENSG00000104497 \| SNX16 \| 1.25 \| 1.57E-08 \| 4.29E-08 \| \| ENSG00000087842 \| PIR \| -1.13 \| 1.58E-08 \| 4.31E-08 \| \| ENSG00000281332 \|  \| 1.00 \| 1.61E-08 \| 4.40E-08 \| \| ENSG00000109881 \| CCDC34 \| -1.46 \| 1.64E-08 \| 4.46E-08 \| \| ENSG00000100505 \| TRIM9 \| 1.72 \| 1.64E-08 \| 4.47E-08 \| \| ENSG00000204257 \| HLA-DMA \| -1.64 \| 1.64E-08 \| 4.47E-08 \| \| ENSG00000114933 \| INO80D \| 0.60 \| 1.65E-08 \| 4.51E-08 \| \| ENSG00000168546 \| GFRA2 \| -5.94 \| 1.67E-08 \| 4.54E-08 \| \| ENSG00000124491 \| F13A1 \| -3.06 \| 1.71E-08 \| 4.65E-08 \| \| ENSG00000100784 \| RPS6KA5 \| 1.00 \| 1.72E-08 \| 4.69E-08 \| \| ENSG00000253535 \|  \| 3.67 \| 1.73E-08 \| 4.70E-08 \| \| ENSG00000164853 \| UNCX \| -2.93 \| 1.74E-08 \| 4.74E-08 \| \| ENSG00000102003 \| SYP \| -1.03 \| 1.75E-08 \| 4.75E-08 \| \| ENSG00000172586 \| CHCHD1 \| -0.77 \| 1.76E-08 \| 4.79E-08 \| \| ENSG00000141750 \| STAC2 \| 0.86 \| 1.77E-08 \| 4.81E-08 \| \| ENSG00000137766 \| UNC13C \| 1.24 \| 1.79E-08 \| 4.88E-08 \| \| ENSG00000185158 \| LRRC37B \| -1.00 \| 1.85E-08 \| 5.01E-08 \| \| ENSG00000254827 \| SLC22A18AS \| -1.21 \| 1.85E-08 \| 5.04E-08 \| \| ENSG00000100852 \| ARHGAP5 \| 1.65 \| 1.88E-08 \| 5.10E-08 \| \| ENSG00000102575 \| ACP5 \| -1.46 \| 1.90E-08 \| 5.15E-08 \| \| ENSG00000226380 \| LINC-PINT \| 1.43 \| 1.90E-08 \| 5.16E-08 \| \| ENSG00000104760 \| FGL1 \| -3.13 \| 1.93E-08 \| 5.24E-08 \| \| ENSG00000269609 \| RPARP-AS1 \| -1.25 \| 1.94E-08 \| 5.26E-08 \| \| ENSG00000112312 \| GMNN \| -1.23 \| 1.95E-08 \| 5.28E-08 \| \| ENSG00000076555 \| ACACB \| -1.19 \| 1.95E-08 \| 5.29E-08 \| \| ENSG00000171714 \| ANO5 \| 0.93 \| 1.97E-08 \| 5.32E-08 \| \| ENSG00000171444 \| MCC \| 1.13 \| 1.99E-08 \| 5.39E-08 \| \| ENSG00000174501 \| ANKRD36C \| -2.91 \| 2.00E-08 \| 5.40E-08 \| \| ENSG00000253200 \|  \| 0.64 \| 2.00E-08 \| 5.40E-08 \| \| ENSG00000119929 \| CUTC \| -0.86 \| 2.03E-08 \| 5.50E-08 \| \| ENSG00000198223 \| CSF2RA \| -0.92 \| 2.06E-08 \| 5.57E-08 \| \| ENSG00000180667 \| YOD1 \| -0.66 \| 2.07E-08 \| 5.59E-08 \| \| ENSG00000139055 \| ERP27 \| -1.68 \| 2.28E-08 \| 6.14E-08 \| \| ENSG00000081177 \| EXD2 \| -0.67 \| 2.38E-08 \| 6.42E-08 \| \| ENSG00000189007 \| ADAT2 \| -1.04 \| 2.39E-08 \| 6.45E-08 \| \| ENSG00000137441 \| FGFBP2 \| 6.85 \| 2.44E-08 \| 6.56E-08 \| \| ENSG00000148143 \| ZNF462 \| -2.89 \| 2.55E-08 \| 6.86E-08 \| \| ENSG00000204025 \| TRPC5OS \| -4.35 \| 2.62E-08 \| 7.04E-08 \| \| ENSG00000106123 \| EPHB6 \| -0.88 \| 2.63E-08 \| 7.07E-08 \| \| ENSG00000158163 \| DZIP1L \| -1.56 \| 2.68E-08 \| 7.20E-08 \| \| ENSG00000167210 \| LOXHD1 \| 0.72 \| 2.68E-08 \| 7.21E-08 \| \| ENSG00000006625 \| GGCT \| -0.96 \| 2.69E-08 \| 7.22E-08 \| \| ENSG00000108384 \| RAD51C \| -0.69 \| 2.76E-08 \| 7.41E-08 \| \| ENSG00000167785 \| ZNF558 \| 0.74 \| 2.77E-08 \| 7.43E-08 \| \| ENSG00000241015 \| TPM3P9 \| 0.60 \| 2.88E-08 \| 7.72E-08 \| \| ENSG00000073331 \| ALPK1 \| -0.72 \| 2.88E-08 \| 7.73E-08 \| \| ENSG00000154822 \| PLCL2 \| 0.63 \| 2.89E-08 \| 7.76E-08 \| \| ENSG00000173372 \| C1QA \| -6.80 \| 2.90E-08 \| 7.77E-08 \| \| ENSG00000121895 \| TMEM156 \| -2.12 \| 2.96E-08 \| 7.92E-08 \| \| ENSG00000152822 \| GRM1 \| 2.64 \| 3.03E-08 \| 8.11E-08 \| \| ENSG00000152217 \| SETBP1 \| 0.62 \| 3.03E-08 \| 8.12E-08 \| \| ENSG00000127423 \| AUNIP \| -1.35 \| 3.04E-08 \| 8.14E-08 \| \| ENSG00000283199 \| C13orf46 \| 1.51 \| 3.14E-08 \| 8.40E-08 \| \| ENSG00000223551 \|  \| -1.64 \| 3.17E-08 \| 8.49E-08 \| \| ENSG00000158050 \| DUSP2 \| -1.48 \| 3.22E-08 \| 8.62E-08 \| \| ENSG00000276136 \|  \| 2.09 \| 3.26E-08 \| 8.72E-08 \| \| ENSG00000151553 \| FAM160B1 \| -0.66 \| 3.34E-08 \| 8.91E-08 \| \| ENSG00000107537 \| PHYH \| -1.04 \| 3.38E-08 \| 9.03E-08 \| \| ENSG00000253953 \| PCDHGB4 \| -0.64 \| 3.39E-08 \| 9.06E-08 \| \| ENSG00000272674 \| PCDHB16 \| -1.95 \| 3.41E-08 \| 9.11E-08 \| \| ENSG00000118503 \| TNFAIP3 \| 0.99 \| 3.42E-08 \| 9.13E-08 \| \| ENSG00000162892 \| IL24 \| 4.66 \| 3.43E-08 \| 9.14E-08 \| \| ENSG00000163517 \| HDAC11 \| -1.18 \| 3.45E-08 \| 9.20E-08 \| \| ENSG00000072210 \| ALDH3A2 \| -0.63 \| 3.62E-08 \| 9.66E-08 \| \| ENSG00000105642 \| KCNN1 \| -3.45 \| 3.73E-08 \| 9.93E-08 \| \| ENSG00000153714 \| LURAP1L \| 1.28 \| 3.83E-08 \| 1.02E-07 \| \| ENSG00000188215 \| DCUN1D3 \| 0.93 \| 3.91E-08 \| 1.04E-07 \| \| ENSG00000177283 \| FZD8 \| 1.15 \| 3.93E-08 \| 1.04E-07 \| \| ENSG00000260188 \|  \| -1.80 \| 4.00E-08 \| 1.06E-07 \| \| ENSG00000151150 \| ANK3 \| -2.97 \| 4.00E-08 \| 1.06E-07 \| \| ENSG00000176386 \| CDC26 \| 0.66 \| 4.05E-08 \| 1.08E-07 \| \| ENSG00000125510 \| OPRL1 \| -1.92 \| 4.13E-08 \| 1.10E-07 \| \| ENSG00000188133 \| TMEM215 \| -2.10 \| 4.16E-08 \| 1.10E-07 \| \| ENSG00000264456 \|  \| -1.41 \| 4.36E-08 \| 1.15E-07 \| \| ENSG00000156298 \| TSPAN7 \| 1.95 \| 4.50E-08 \| 1.19E-07 \| \| ENSG00000120278 \| PLEKHG1 \| -4.28 \| 4.50E-08 \| 1.19E-07 \| \| ENSG00000159208 \| CIART \| -1.64 \| 4.53E-08 \| 1.20E-07 \| \| ENSG00000144306 \| SCRN3 \| -0.59 \| 4.60E-08 \| 1.22E-07 \| \| ENSG00000104147 \| OIP5 \| -2.03 \| 4.61E-08 \| 1.22E-07 \| \| ENSG00000081760 \| AACS \| -0.62 \| 4.67E-08 \| 1.24E-07 \| \| ENSG00000166396 \| SERPINB7 \| 6.72 \| 4.68E-08 \| 1.24E-07 \| \| ENSG00000158481 \| CD1C \| 0.79 \| 4.85E-08 \| 1.28E-07 \| \| ENSG00000175066 \| GK5 \| 0.64 \| 4.90E-08 \| 1.30E-07 \| \| ENSG00000104689 \| TNFRSF10A \| -0.83 \| 4.93E-08 \| 1.30E-07 \| \| ENSG00000197496 \| SLC2A10 \| -1.82 \| 4.94E-08 \| 1.30E-07 \| \| ENSG00000158477 \| CD1A \| 1.09 \| 5.19E-08 \| 1.37E-07 \| \| ENSG00000069966 \| GNB5 \| 0.70 \| 5.30E-08 \| 1.40E-07 \| \| ENSG00000054598 \| FOXC1 \| 1.23 \| 5.31E-08 \| 1.40E-07 \| \| ENSG00000115041 \| KCNIP3 \| -1.52 \| 5.38E-08 \| 1.42E-07 \| \| ENSG00000121211 \| MND1 \| -0.70 \| 5.42E-08 \| 1.43E-07 \| \| ENSG00000171777 \| RASGRP4 \| -6.56 \| 5.44E-08 \| 1.43E-07 \| \| ENSG00000122420 \| PTGFR \| 4.07 \| 5.46E-08 \| 1.44E-07 \| \| ENSG00000250644 \|  \| 1.72 \| 5.48E-08 \| 1.44E-07 \| \| ENSG00000261594 \| TPBGL \| -1.20 \| 5.56E-08 \| 1.46E-07 \| \| ENSG00000277895 \|  \| 2.45 \| 5.60E-08 \| 1.47E-07 \| \| ENSG00000243232 \| PCDHAC2 \| -1.38 \| 5.64E-08 \| 1.48E-07 \| \| ENSG00000111788 \|  \| -1.35 \| 5.83E-08 \| 1.53E-07 \| \| ENSG00000123810 \| B9D2 \| 0.66 \| 6.06E-08 \| 1.59E-07 \| \| ENSG00000107890 \| ANKRD26 \| -1.18 \| 6.11E-08 \| 1.60E-07 \| \| ENSG00000124444 \| ZNF576 \| 0.60 \| 6.38E-08 \| 1.67E-07 \| \| ENSG00000172954 \| LCLAT1 \| -0.59 \| 6.50E-08 \| 1.70E-07 \| \| ENSG00000120322 \| PCDHB8 \| -1.59 \| 6.83E-08 \| 1.79E-07 \| \| ENSG00000202474 \|  \| 1.15 \| 7.01E-08 \| 1.83E-07 \| \| ENSG00000086205 \| FOLH1 \| -1.22 \| 7.04E-08 \| 1.84E-07 \| \| ENSG00000104967 \| NOVA2 \| -3.13 \| 7.05E-08 \| 1.84E-07 \| \| ENSG00000092445 \| TYRO3 \| -0.74 \| 7.11E-08 \| 1.86E-07 \| \| ENSG00000001617 \| SEMA3F \| 0.81 \| 7.17E-08 \| 1.87E-07 \| \| ENSG00000136696 \| IL36B \| 2.27 \| 7.20E-08 \| 1.88E-07 \| \| ENSG00000116285 \| ERRFI1 \| 0.70 \| 7.25E-08 \| 1.89E-07 \| \| ENSG00000165490 \| DDIAS \| -1.14 \| 7.64E-08 \| 1.99E-07 \| \| ENSG00000182257 \| PRR34 \| -2.71 \| 7.65E-08 \| 1.99E-07 \| \| ENSG00000041982 \| TNC \| -3.66 \| 7.67E-08 \| 2.00E-07 \| \| ENSG00000145781 \| COMMD10 \| -0.71 \| 7.95E-08 \| 2.07E-07 \| \| ENSG00000172236 \| TPSAB1 \| -3.93 \| 7.98E-08 \| 2.08E-07 \| \| ENSG00000028839 \| TBPL1 \| 0.65 \| 8.04E-08 \| 2.09E-07 \| \| ENSG00000137460 \| FHDC1 \| 1.39 \| 8.08E-08 \| 2.10E-07 \| \| ENSG00000127152 \| BCL11B \| 2.01 \| 8.18E-08 \| 2.13E-07 \| \| ENSG00000115266 \| APC2 \| 1.32 \| 8.30E-08 \| 2.16E-07 \| \| ENSG00000228327 \|  \| 0.84 \| 8.37E-08 \| 2.17E-07 \| \| ENSG00000181458 \| TMEM45A \| 1.05 \| 8.47E-08 \| 2.20E-07 \| \| ENSG00000279400 \|  \| 2.56 \| 8.74E-08 \| 2.27E-07 \| \| ENSG00000141934 \| PLPP2 \| -3.87 \| 8.88E-08 \| 2.31E-07 \| \| ENSG00000049130 \| KITLG \| 0.63 \| 8.95E-08 \| 2.32E-07 \| \| ENSG00000279088 \|  \| -1.02 \| 9.07E-08 \| 2.35E-07 \| \| ENSG00000279667 \|  \| 0.87 \| 9.22E-08 \| 2.39E-07 \| \| ENSG00000134874 \| DZIP1 \| -1.03 \| 9.30E-08 \| 2.41E-07 \| \| ENSG00000254281 \|  \| 1.66 \| 9.34E-08 \| 2.42E-07 \| \| ENSG00000113638 \| TTC33 \| 0.87 \| 9.47E-08 \| 2.45E-07 \| \| ENSG00000133636 \| NTS \| -6.57 \| 9.67E-08 \| 2.50E-07 \| \| ENSG00000269743 \| SLC25A53 \| 0.83 \| 9.85E-08 \| 2.55E-07 \| \| ENSG00000062582 \| MRPS24 \| 2.28 \| 9.87E-08 \| 2.55E-07 \| \| ENSG00000124785 \| NRN1 \| -0.74 \| 9.87E-08 \| 2.55E-07 \| \| ENSG00000101307 \| SIRPB1 \| -1.02 \| 9.99E-08 \| 2.58E-07 \| \| ENSG00000245694 \| CRNDE \| 0.61 \| 1.00E-07 \| 2.59E-07 \| \| ENSG00000245694 \| LOC101927480 \| 0.61 \| 1.00E-07 \| 2.59E-07 \| \| ENSG00000185215 \| TNFAIP2 \| 0.99 \| 1.02E-07 \| 2.62E-07 \| \| ENSG00000119986 \| AVPI1 \| -1.09 \| 1.02E-07 \| 2.62E-07 \| \| ENSG00000187837 \| HIST1H1C \| 0.93 \| 1.02E-07 \| 2.63E-07 \| \| ENSG00000215458 \| AATBC \| -2.82 \| 1.02E-07 \| 2.63E-07 \| \| ENSG00000267296 \| CEBPA-DT \| -1.29 \| 1.02E-07 \| 2.64E-07 \| \| ENSG00000035664 \| DAPK2 \| -0.72 \| 1.06E-07 \| 2.73E-07 \| \| ENSG00000196427 \| NBPF4 \| 1.39 \| 1.06E-07 \| 2.74E-07 \| \| ENSG00000163704 \| PRRT3 \| 0.66 \| 1.10E-07 \| 2.84E-07 \| \| ENSG00000125319 \| C17orf53 \| -0.78 \| 1.11E-07 \| 2.85E-07 \| \| ENSG00000185219 \| ZNF445 \| -0.65 \| 1.17E-07 \| 3.01E-07 \| \| ENSG00000164796 \| CSMD3 \| 1.78 \| 1.18E-07 \| 3.03E-07 \| \| ENSG00000207561 \| MIR635 \| 3.01 \| 1.19E-07 \| 3.05E-07 \| \| ENSG00000231584 \| FAHD2CP \| -1.68 \| 1.20E-07 \| 3.09E-07 \| \| ENSG00000169684 \| CHRNA5 \| -1.57 \| 1.22E-07 \| 3.13E-07 \| \| ENSG00000254777 \|  \| -2.49 \| 1.23E-07 \| 3.15E-07 \| \| ENSG00000110427 \| KIAA1549L \| 1.70 \| 1.23E-07 \| 3.15E-07 \| \| ENSG00000138587 \| MNS1 \| -1.94 \| 1.25E-07 \| 3.22E-07 \| \| ENSG00000085831 \| TTC39A \| -2.27 \| 1.28E-07 \| 3.28E-07 \| \| ENSG00000249740 \| LINC01265 \| 2.70 \| 1.28E-07 \| 3.29E-07 \| \| ENSG00000164749 \| HNF4G \| -1.22 \| 1.30E-07 \| 3.33E-07 \| \| ENSG00000083750 \| RRAGB \| -0.78 \| 1.30E-07 \| 3.35E-07 \| \| ENSG00000232859 \| LYRM9 \| -1.03 \| 1.31E-07 \| 3.37E-07 \| \| ENSG00000135976 \| ANKRD36 \| -2.00 \| 1.32E-07 \| 3.38E-07 \| \| ENSG00000240764 \| PCDHGC5 \| -1.90 \| 1.32E-07 \| 3.39E-07 \| \| ENSG00000260303 \|  \| -5.57 \| 1.33E-07 \| 3.40E-07 \| \| ENSG00000226435 \| ANKRD18DP \| 1.43 \| 1.35E-07 \| 3.45E-07 \| \| ENSG00000215788 \| TNFRSF25 \| -4.16 \| 1.39E-07 \| 3.57E-07 \| \| ENSG00000198483 \| ANKRD35 \| -1.20 \| 1.40E-07 \| 3.57E-07 \| \| ENSG00000130829 \| DUSP9 \| -1.68 \| 1.44E-07 \| 3.67E-07 \| \| ENSG00000178966 \| RMI1 \| -0.65 \| 1.44E-07 \| 3.68E-07 \| \| ENSG00000145824 \| CXCL14 \| 2.59 \| 1.44E-07 \| 3.69E-07 \| \| ENSG00000152661 \| GJA1 \| -1.35 \| 1.44E-07 \| 3.69E-07 \| \| ENSG00000157613 \| CREB3L1 \| -1.05 \| 1.51E-07 \| 3.86E-07 \| \| ENSG00000257704 \| INAFM1 \| 0.59 \| 1.52E-07 \| 3.87E-07 \| \| ENSG00000117009 \| KMO \| 0.81 \| 1.53E-07 \| 3.90E-07 \| \| ENSG00000223804 \|  \| 1.05 \| 1.53E-07 \| 3.90E-07 \| \| ENSG00000265531 \| FCGR1CP \| -3.04 \| 1.55E-07 \| 3.95E-07 \| \| ENSG00000259865 \|  \| -1.07 \| 1.57E-07 \| 4.01E-07 \| \| ENSG00000255108 \|  \| 1.92 \| 1.59E-07 \| 4.06E-07 \| \| ENSG00000166938 \| DIS3L \| -0.59 \| 1.64E-07 \| 4.18E-07 \| \| ENSG00000176358 \| TAC4 \| -5.52 \| 1.65E-07 \| 4.19E-07 \| \| ENSG00000052850 \| ALX4 \| -1.88 \| 1.65E-07 \| 4.20E-07 \| \| ENSG00000164953 \| TMEM67 \| 0.88 \| 1.67E-07 \| 4.25E-07 \| \| ENSG00000264538 \|  \| -0.77 \| 1.68E-07 \| 4.27E-07 \| \| ENSG00000214826 \| DDX12P \| -1.54 \| 1.70E-07 \| 4.32E-07 \| \| ENSG00000140398 \| NEIL1 \| -1.21 \| 1.73E-07 \| 4.39E-07 \| \| ENSG00000272255 \|  \| 1.65 \| 1.77E-07 \| 4.50E-07 \| \| ENSG00000233155 \|  \| 1.37 \| 1.78E-07 \| 4.52E-07 \| \| ENSG00000006432 \| MAP3K9 \| 2.63 \| 1.80E-07 \| 4.56E-07 \| \| ENSG00000121851 \| POLR3GL \| -0.78 \| 1.81E-07 \| 4.58E-07 \| \| ENSG00000137210 \| TMEM14B \| -0.60 \| 1.82E-07 \| 4.60E-07 \| \| ENSG00000166033 \| HTRA1 \| -6.37 \| 1.82E-07 \| 4.61E-07 \| \| ENSG00000165630 \| PRPF18 \| -0.68 \| 1.85E-07 \| 4.69E-07 \| \| ENSG00000086548 \| CEACAM6 \| -6.43 \| 1.90E-07 \| 4.82E-07 \| \| ENSG00000197375 \| SLC22A5 \| -1.37 \| 2.01E-07 \| 5.07E-07 \| \| ENSG00000183770 \| FOXL2 \| -0.98 \| 2.02E-07 \| 5.10E-07 \| \| ENSG00000131044 \| TTLL9 \| 2.67 \| 2.07E-07 \| 5.22E-07 \| \| ENSG00000136367 \| ZFHX2 \| 0.92 \| 2.08E-07 \| 5.26E-07 \| \| ENSG00000165046 \| LETM2 \| 0.73 \| 2.09E-07 \| 5.27E-07 \| \| ENSG00000171680 \| PLEKHG5 \| -1.63 \| 2.09E-07 \| 5.28E-07 \| \| ENSG00000141068 \| KSR1 \| -0.98 \| 2.10E-07 \| 5.31E-07 \| \| ENSG00000168994 \| PXDC1 \| -0.67 \| 2.14E-07 \| 5.40E-07 \| \| ENSG00000196205 \|  \| -0.59 \| 2.15E-07 \| 5.42E-07 \| \| ENSG00000168778 \| TCTN2 \| -0.65 \| 2.19E-07 \| 5.52E-07 \| \| ENSG00000197629 \| MPEG1 \| -3.33 \| 2.23E-07 \| 5.62E-07 \| \| ENSG00000100490 \| CDKL1 \| -1.60 \| 2.30E-07 \| 5.80E-07 \| \| ENSG00000275764 \|  \| 0.99 \| 2.36E-07 \| 5.94E-07 \| \| ENSG00000077942 \| FBLN1 \| -6.46 \| 2.37E-07 \| 5.97E-07 \| \| ENSG00000254139 \| LOC286178 \| 2.72 \| 2.39E-07 \| 6.00E-07 \| \| ENSG00000010704 \| HFE \| -6.42 \| 2.41E-07 \| 6.05E-07 \| \| ENSG00000109501 \| WFS1 \| -6.39 \| 2.44E-07 \| 6.14E-07 \| \| ENSG00000218336 \| TENM3 \| 0.97 \| 2.47E-07 \| 6.19E-07 \| \| ENSG00000182487 \| NCF1B \| 0.62 \| 2.54E-07 \| 6.38E-07 \| \| ENSG00000280088 \|  \| 0.81 \| 2.56E-07 \| 6.42E-07 \| \| ENSG00000240990 \| HOXA11-AS \| -2.18 \| 2.57E-07 \| 6.45E-07 \| \| ENSG00000046653 \| GPM6B \| -2.65 \| 2.65E-07 \| 6.63E-07 \| \| ENSG00000100601 \| ALKBH1 \| 0.60 \| 2.65E-07 \| 6.64E-07 \| \| ENSG00000135333 \| EPHA7 \| 1.28 \| 2.65E-07 \| 6.65E-07 \| \| ENSG00000178385 \| PLEKHM3 \| 0.65 \| 2.78E-07 \| 6.96E-07 \| \| ENSG00000182704 \| TSKU \| -2.12 \| 2.79E-07 \| 6.98E-07 \| \| ENSG00000186812 \| ZNF397 \| 0.64 \| 2.81E-07 \| 7.02E-07 \| \| ENSG00000204044 \| SLC12A5-AS1 \| -1.38 \| 2.89E-07 \| 7.22E-07 \| \| ENSG00000113966 \| ARL6 \| 1.11 \| 2.91E-07 \| 7.26E-07 \| \| ENSG00000188185 \| LINC00265 \| 0.90 \| 2.96E-07 \| 7.40E-07 \| \| ENSG00000120885 \| CLU \| -0.94 \| 2.98E-07 \| 7.44E-07 \| \| ENSG00000137831 \| UACA \| -0.92 \| 3.06E-07 \| 7.65E-07 \| \| ENSG00000262097 \| LINC02185 \| 0.96 \| 3.13E-07 \| 7.81E-07 \| \| ENSG00000162723 \| SLAMF9 \| -1.99 \| 3.14E-07 \| 7.83E-07 \| \| ENSG00000114737 \| CISH \| -2.90 \| 3.15E-07 \| 7.86E-07 \| \| ENSG00000183793 \| NPIPA5 \| 0.63 \| 3.20E-07 \| 7.96E-07 \| \| ENSG00000104290 \| FZD3 \| 0.68 \| 3.20E-07 \| 7.98E-07 \| \| ENSG00000184408 \| KCND2 \| 1.95 \| 3.31E-07 \| 8.23E-07 \| \| ENSG00000170374 \| SP7 \| -3.77 \| 3.48E-07 \| 8.67E-07 \| \| ENSG00000122873 \| CISD1 \| -0.60 \| 3.62E-07 \| 9.00E-07 \| \| ENSG00000265728 \|  \| -1.95 \| 3.66E-07 \| 9.09E-07 \| \| ENSG00000185668 \| POU3F1 \| -1.96 \| 3.67E-07 \| 9.11E-07 \| \| ENSG00000229563 \| LINC01204 \| 2.85 \| 3.72E-07 \| 9.23E-07 \| \| ENSG00000184602 \| SNN \| -1.00 \| 3.79E-07 \| 9.41E-07 \| \| ENSG00000214189 \|  \| -1.60 \| 3.80E-07 \| 9.44E-07 \| \| ENSG00000234362 \| LINC01914 \| -4.06 \| 3.85E-07 \| 9.56E-07 \| \| ENSG00000168955 \| TM4SF20 \| 3.42 \| 3.99E-07 \| 9.89E-07 \| \| ENSG00000171551 \| ECEL1 \| -5.36 \| 4.10E-07 \| 1.02E-06 \| \| ENSG00000135127 \| BICDL1 \| -0.81 \| 4.11E-07 \| 1.02E-06 \| \| ENSG00000133321 \| RARRES3 \| -6.28 \| 4.12E-07 \| 1.02E-06 \| \| ENSG00000169992 \| NLGN2 \| 0.90 \| 4.13E-07 \| 1.02E-06 \| \| ENSG00000103528 \| SYT17 \| 0.90 \| 4.13E-07 \| 1.02E-06 \| \| ENSG00000221909 \| FAM200A \| 0.79 \| 4.14E-07 \| 1.02E-06 \| \| ENSG00000258944 \|  \| 1.58 \| 4.23E-07 \| 1.04E-06 \| \| ENSG00000173727 \|  \| 1.66 \| 4.34E-07 \| 1.07E-06 \| \| ENSG00000206262 \| FOXL2NB \| -2.39 \| 4.34E-07 \| 1.07E-06 \| \| ENSG00000153898 \| MCOLN2 \| -1.49 \| 4.37E-07 \| 1.08E-06 \| \| ENSG00000162444 \| RBP7 \| -6.15 \| 4.37E-07 \| 1.08E-06 \| \| ENSG00000279631 \|  \| -1.34 \| 4.37E-07 \| 1.08E-06 \| \| ENSG00000281398 \| SNHG4 \| -1.10 \| 4.43E-07 \| 1.09E-06 \| \| ENSG00000189337 \| KAZN \| 0.73 \| 4.55E-07 \| 1.12E-06 \| \| ENSG00000198873 \| GRK5 \| 0.77 \| 4.57E-07 \| 1.13E-06 \| \| ENSG00000268112 \|  \| 1.22 \| 4.69E-07 \| 1.16E-06 \| \| ENSG00000074935 \| TUBE1 \| 0.68 \| 4.76E-07 \| 1.17E-06 \| \| ENSG00000151014 \| NOCT \| 0.95 \| 4.88E-07 \| 1.20E-06 \| \| ENSG00000184588 \| PDE4B \| 1.16 \| 4.91E-07 \| 1.21E-06 \| \| ENSG00000151612 \| ZNF827 \| -0.84 \| 5.14E-07 \| 1.26E-06 \| \| ENSG00000181513 \| ACBD4 \| -0.76 \| 5.21E-07 \| 1.28E-06 \| \| ENSG00000165694 \| FRMD7 \| 4.01 \| 5.22E-07 \| 1.28E-06 \| \| ENSG00000214425 \|  \| -1.44 \| 5.23E-07 \| 1.29E-06 \| \| ENSG00000279415 \|  \| 1.00 \| 5.31E-07 \| 1.30E-06 \| \| ENSG00000176225 \| RTTN \| -0.80 \| 5.31E-07 \| 1.30E-06 \| \| ENSG00000067842 \| ATP2B3 \| -2.14 \| 5.37E-07 \| 1.32E-06 \| \| ENSG00000242732 \| RTL5 \| -0.97 \| 5.46E-07 \| 1.34E-06 \| \| ENSG00000203666 \| EFCAB2 \| -0.94 \| 5.67E-07 \| 1.39E-06 \| \| ENSG00000198286 \| CARD11 \| -6.27 \| 5.73E-07 \| 1.40E-06 \| \| ENSG00000271425 \| NBPF10 \| 1.10 \| 5.84E-07 \| 1.43E-06 \| \| ENSG00000171224 \| FAM241B \| -3.38 \| 5.88E-07 \| 1.44E-06 \| \| ENSG00000149243 \| KLHL35 \| 0.86 \| 5.92E-07 \| 1.45E-06 \| \| ENSG00000131401 \| NAPSB \| -6.10 \| 5.97E-07 \| 1.46E-06 \| \| ENSG00000185252 \| ZNF74 \| -1.41 \| 5.97E-07 \| 1.46E-06 \| \| ENSG00000153363 \| LINC00467 \| -0.77 \| 5.97E-07 \| 1.46E-06 \| \| ENSG00000182700 \| IGIP \| 1.22 \| 6.03E-07 \| 1.47E-06 \| \| ENSG00000158806 \| NPM2 \| -2.89 \| 6.10E-07 \| 1.49E-06 \| \| ENSG00000182912 \| TSPEAR-AS2 \| -3.36 \| 6.19E-07 \| 1.51E-06 \| \| ENSG00000196420 \| S100A5 \| -2.65 \| 6.31E-07 \| 1.54E-06 \| \| ENSG00000153291 \| SLC25A27 \| 1.83 \| 6.34E-07 \| 1.55E-06 \| \| ENSG00000197557 \| TTC30A \| 0.95 \| 6.41E-07 \| 1.56E-06 \| \| ENSG00000130349 \| C6orf203 \| -0.62 \| 6.41E-07 \| 1.56E-06 \| \| ENSG00000180061 \| TMEM150B \| -1.47 \| 6.44E-07 \| 1.57E-06 \| \| ENSG00000269890 \|  \| -1.21 \| 6.44E-07 \| 1.57E-06 \| \| ENSG00000196912 \| ANKRD36B \| -2.23 \| 6.61E-07 \| 1.61E-06 \| \| ENSG00000135324 \| MRAP2 \| 1.76 \| 6.65E-07 \| 1.62E-06 \| \| ENSG00000059804 \| SLC2A3 \| -0.60 \| 6.65E-07 \| 1.62E-06 \| \| ENSG00000170425 \| ADORA2B \| 0.72 \| 6.69E-07 \| 1.63E-06 \| \| ENSG00000224167 \| HP08777 \| -4.49 \| 6.70E-07 \| 1.63E-06 \| \| ENSG00000184058 \| TBX1 \| -0.95 \| 6.72E-07 \| 1.64E-06 \| \| ENSG00000105514 \| RAB3D \| -0.97 \| 6.85E-07 \| 1.67E-06 \| \| ENSG00000266733 \| TBC1D29P \| -2.04 \| 6.87E-07 \| 1.67E-06 \| \| ENSG00000119698 \| PPP4R4 \| 2.28 \| 6.98E-07 \| 1.70E-06 \| \| ENSG00000270959 \| LPP-AS2 \| 0.73 \| 6.99E-07 \| 1.70E-06 \| \| ENSG00000170365 \| SMAD1 \| -0.98 \| 7.52E-07 \| 1.83E-06 \| \| ENSG00000183077 \| AFMID \| -0.59 \| 7.65E-07 \| 1.86E-06 \| \| ENSG00000111341 \| MGP \| -2.57 \| 7.75E-07 \| 1.88E-06 \| \| ENSG00000153132 \| CLGN \| -1.82 \| 7.77E-07 \| 1.88E-06 \| \| ENSG00000135378 \| PRRG4 \| -0.93 \| 7.97E-07 \| 1.93E-06 \| \| ENSG00000243766 \|  \| -6.04 \| 8.07E-07 \| 1.96E-06 \| \| ENSG00000165813 \| CCDC186 \| -0.71 \| 8.10E-07 \| 1.96E-06 \| \| ENSG00000119862 \| LGALSL \| -1.46 \| 8.21E-07 \| 1.99E-06 \| \| ENSG00000149926 \| FAM57B \| -1.86 \| 8.22E-07 \| 1.99E-06 \| \| ENSG00000205189 \| ZBTB10 \| -0.69 \| 8.31E-07 \| 2.01E-06 \| \| ENSG00000053747 \| LAMA3 \| -1.02 \| 8.37E-07 \| 2.03E-06 \| \| ENSG00000104055 \| TGM5 \| -2.50 \| 8.52E-07 \| 2.06E-06 \| \| ENSG00000138399 \| FASTKD1 \| -0.71 \| 8.57E-07 \| 2.07E-06 \| \| ENSG00000124882 \| EREG \| -2.08 \| 8.58E-07 \| 2.07E-06 \| \| ENSG00000223509 \|  \| -1.79 \| 8.60E-07 \| 2.08E-06 \| \| ENSG00000274818 \|  \| 1.32 \| 8.85E-07 \| 2.14E-06 \| \| ENSG00000182183 \| SHISAL2A \| -3.96 \| 8.86E-07 \| 2.14E-06 \| \| ENSG00000265975 \|  \| 0.97 \| 8.91E-07 \| 2.15E-06 \| \| ENSG00000101224 \| CDC25B \| -0.60 \| 8.92E-07 \| 2.15E-06 \| \| ENSG00000112246 \| SIM1 \| 3.47 \| 9.41E-07 \| 2.27E-06 \| \| ENSG00000213145 \| CRIP1 \| -1.19 \| 9.70E-07 \| 2.34E-06 \| \| ENSG00000137726 \| FXYD6 \| -2.96 \| 9.82E-07 \| 2.36E-06 \| \| ENSG00000267247 \|  \| -3.89 \| 1.00E-06 \| 2.41E-06 \| \| ENSG00000061455 \| PRDM6 \| -5.25 \| 1.03E-06 \| 2.47E-06 \| \| ENSG00000172671 \| ZFAND4 \| 0.71 \| 1.04E-06 \| 2.49E-06 \| \| ENSG00000198270 \| TMEM116 \| -0.64 \| 1.04E-06 \| 2.50E-06 \| \| ENSG00000141034 \| GID4 \| 0.61 \| 1.05E-06 \| 2.52E-06 \| \| ENSG00000159618 \| ADGRG5 \| -3.93 \| 1.06E-06 \| 2.53E-06 \| \| ENSG00000102996 \| MMP15 \| -0.79 \| 1.08E-06 \| 2.58E-06 \| \| ENSG00000231808 \|  \| -0.78 \| 1.08E-06 \| 2.59E-06 \| \| ENSG00000233901 \| LINC01503 \| -1.44 \| 1.09E-06 \| 2.60E-06 \| \| ENSG00000226334 \|  \| 1.20 \| 1.09E-06 \| 2.62E-06 \| \| ENSG00000139832 \| RAB20 \| -3.31 \| 1.10E-06 \| 2.62E-06 \| \| ENSG00000257181 \|  \| 2.84 \| 1.10E-06 \| 2.65E-06 \| \| ENSG00000271643 \|  \| 0.74 \| 1.12E-06 \| 2.67E-06 \| \| ENSG00000146090 \| RASGEF1C \| 0.85 \| 1.12E-06 \| 2.68E-06 \| \| ENSG00000105963 \| ADAP1 \| 0.72 \| 1.13E-06 \| 2.71E-06 \| \| ENSG00000268027 \|  \| -6.13 \| 1.13E-06 \| 2.71E-06 \| \| ENSG00000111799 \| COL12A1 \| 0.78 \| 1.13E-06 \| 2.71E-06 \| \| ENSG00000256525 \| POLG2 \| -0.77 \| 1.16E-06 \| 2.77E-06 \| \| ENSG00000256628 \| ZBTB11-AS1 \| 0.62 \| 1.16E-06 \| 2.78E-06 \| \| ENSG00000151657 \| KIN \| -0.65 \| 1.18E-06 \| 2.83E-06 \| \| ENSG00000046604 \| DSG2 \| -1.50 \| 1.19E-06 \| 2.84E-06 \| \| ENSG00000119227 \| PIGZ \| -0.73 \| 1.21E-06 \| 2.88E-06 \| \| ENSG00000162975 \| KCNF1 \| 1.35 \| 1.22E-06 \| 2.91E-06 \| \| ENSG00000274265 \|  \| 0.70 \| 1.22E-06 \| 2.92E-06 \| \| ENSG00000229180 \| GS1-124K5.11 \| 0.81 \| 1.24E-06 \| 2.97E-06 \| \| ENSG00000138185 \| ENTPD1 \| -3.88 \| 1.25E-06 \| 2.97E-06 \| \| ENSG00000205277 \| MUC12 \| 2.71 \| 1.25E-06 \| 2.97E-06 \| \| ENSG00000170577 \| SIX2 \| -2.58 \| 1.26E-06 \| 3.01E-06 \| \| ENSG00000124493 \| GRM4 \| -2.47 \| 1.27E-06 \| 3.03E-06 \| \| ENSG00000162194 \| LBHD1 \| -0.79 \| 1.27E-06 \| 3.03E-06 \| \| ENSG00000133135 \| RNF128 \| -5.94 \| 1.28E-06 \| 3.06E-06 \| \| ENSG00000187653 \|  \| -1.23 \| 1.28E-06 \| 3.06E-06 \| \| ENSG00000261737 \| CLCA4-AS1 \| 0.81 \| 1.32E-06 \| 3.15E-06 \| \| ENSG00000180822 \| PSMG4 \| -0.86 \| 1.33E-06 \| 3.18E-06 \| \| ENSG00000198142 \| SOWAHC \| 0.70 \| 1.36E-06 \| 3.24E-06 \| \| ENSG00000073670 \| ADAM11 \| -2.01 \| 1.37E-06 \| 3.26E-06 \| \| ENSG00000186642 \| PDE2A \| -1.33 \| 1.38E-06 \| 3.29E-06 \| \| ENSG00000159147 \| DONSON \| -0.61 \| 1.39E-06 \| 3.31E-06 \| \| ENSG00000128165 \| ADM2 \| -1.01 \| 1.41E-06 \| 3.34E-06 \| \| ENSG00000168454 \| TXNDC2 \| 2.27 \| 1.43E-06 \| 3.40E-06 \| \| ENSG00000154721 \| JAM2 \| 1.10 \| 1.44E-06 \| 3.43E-06 \| \| ENSG00000011638 \| TMEM159 \| -1.23 \| 1.46E-06 \| 3.46E-06 \| \| ENSG00000185332 \| TMEM105 \| -2.98 \| 1.46E-06 \| 3.46E-06 \| \| ENSG00000160602 \| NEK8 \| 0.61 \| 1.49E-06 \| 3.53E-06 \| \| ENSG00000147145 \| LPAR4 \| 1.19 \| 1.55E-06 \| 3.67E-06 \| \| ENSG00000163623 \| NKX6-1 \| 0.65 \| 1.55E-06 \| 3.67E-06 \| \| ENSG00000067113 \| PLPP1 \| 0.95 \| 1.58E-06 \| 3.73E-06 \| \| ENSG00000175395 \| ZNF25 \| -0.66 \| 1.59E-06 \| 3.76E-06 \| \| ENSG00000120055 \| C10orf95 \| -1.69 \| 1.59E-06 \| 3.77E-06 \| \| ENSG00000197885 \| NKIRAS1 \| 0.71 \| 1.60E-06 \| 3.79E-06 \| \| ENSG00000138083 \| SIX3 \| -1.08 \| 1.62E-06 \| 3.84E-06 \| \| ENSG00000270231 \| NBPF8 \| 0.79 \| 1.64E-06 \| 3.88E-06 \| \| ENSG00000196092 \| PAX5 \| -1.40 \| 1.65E-06 \| 3.90E-06 \| \| ENSG00000111846 \| GCNT2 \| -6.00 \| 1.66E-06 \| 3.94E-06 \| \| ENSG00000269446 \|  \| -1.11 \| 1.68E-06 \| 3.97E-06 \| \| ENSG00000225774 \|  \| 0.75 \| 1.69E-06 \| 3.99E-06 \| \| ENSG00000214050 \| FBXO16 \| -2.02 \| 1.70E-06 \| 4.01E-06 \| \| ENSG00000280407 \|  \| 0.70 \| 1.70E-06 \| 4.01E-06 \| \| ENSG00000100100 \| PIK3IP1 \| -0.82 \| 1.75E-06 \| 4.12E-06 \| \| ENSG00000100167 \| 03-Sep \| -0.75 \| 1.76E-06 \| 4.16E-06 \| \| ENSG00000163449 \| TMEM169 \| -1.69 \| 1.76E-06 \| 4.16E-06 \| \| ENSG00000186314 \| PRELID2 \| -1.10 \| 1.77E-06 \| 4.18E-06 \| \| ENSG00000267702 \|  \| -2.17 \| 1.77E-06 \| 4.18E-06 \| \| ENSG00000150681 \| RGS18 \| -3.54 \| 1.80E-06 \| 4.24E-06 \| \| ENSG00000206113 \| CFAP99 \| 1.99 \| 1.82E-06 \| 4.28E-06 \| \| ENSG00000116191 \| RALGPS2 \| 0.81 \| 1.82E-06 \| 4.30E-06 \| \| ENSG00000163596 \| ICA1L \| -1.33 \| 1.83E-06 \| 4.30E-06 \| \| ENSG00000168350 \| DEGS2 \| -3.51 \| 1.83E-06 \| 4.30E-06 \| \| ENSG00000251273 \| LINC02228 \| 3.05 \| 1.84E-06 \| 4.32E-06 \| \| ENSG00000171016 \| PYGO1 \| -0.82 \| 1.84E-06 \| 4.34E-06 \| \| ENSG00000152455 \| SUV39H2 \| -0.89 \| 1.87E-06 \| 4.40E-06 \| \| ENSG00000138101 \| DTNB \| -0.90 \| 1.90E-06 \| 4.48E-06 \| \| ENSG00000236901 \| MIR600HG \| -1.46 \| 1.92E-06 \| 4.50E-06 \| \| ENSG00000111879 \| FAM184A \| 1.04 \| 1.92E-06 \| 4.51E-06 \| \| ENSG00000070404 \| FSTL3 \| 0.83 \| 1.92E-06 \| 4.51E-06 \| \| ENSG00000123179 \| EBPL \| -0.66 \| 1.96E-06 \| 4.60E-06 \| \| ENSG00000169247 \| SH3TC2 \| -1.61 \| 1.99E-06 \| 4.66E-06 \| \| ENSG00000103044 \| HAS3 \| -1.61 \| 2.05E-06 \| 4.82E-06 \| \| ENSG00000242861 \|  \| -1.13 \| 2.07E-06 \| 4.86E-06 \| \| ENSG00000183508 \| TENT5C \| -1.41 \| 2.09E-06 \| 4.89E-06 \| \| ENSG00000127946 \| HIP1 \| -0.78 \| 2.10E-06 \| 4.93E-06 \| \| ENSG00000059769 \| DNAJC25 \| 0.66 \| 2.11E-06 \| 4.95E-06 \| \| ENSG00000116675 \| DNAJC6 \| 1.01 \| 2.12E-06 \| 4.96E-06 \| \| ENSG00000103569 \| AQP9 \| 2.54 \| 2.15E-06 \| 5.04E-06 \| \| ENSG00000198157 \| HMGN5 \| 0.62 \| 2.15E-06 \| 5.04E-06 \| \| ENSG00000221817 \| PPP3CB-AS1 \| -0.67 \| 2.21E-06 \| 5.17E-06 \| \| ENSG00000152642 \| GPD1L \| -0.74 \| 2.24E-06 \| 5.25E-06 \| \| ENSG00000130921 \| C12orf65 \| -0.66 \| 2.25E-06 \| 5.26E-06 \| \| ENSG00000050555 \| LAMC3 \| -5.81 \| 2.34E-06 \| 5.47E-06 \| \| ENSG00000187775 \| DNAH17 \| 1.62 \| 2.41E-06 \| 5.62E-06 \| \| ENSG00000114529 \| C3orf52 \| 2.74 \| 2.41E-06 \| 5.62E-06 \| \| ENSG00000196368 \| NUDT11 \| 1.76 \| 2.47E-06 \| 5.76E-06 \| \| ENSG00000234072 \| LOC105374363 \| 0.63 \| 2.49E-06 \| 5.82E-06 \| \| ENSG00000198919 \| DZIP3 \| -1.16 \| 2.52E-06 \| 5.87E-06 \| \| ENSG00000279204 \|  \| 2.61 \| 2.54E-06 \| 5.92E-06 \| \| ENSG00000182348 \| ZNF804B \| 4.45 \| 2.54E-06 \| 5.93E-06 \| \| ENSG00000023608 \| SNAPC1 \| 0.85 \| 2.56E-06 \| 5.96E-06 \| \| ENSG00000148835 \| TAF5 \| -0.61 \| 2.60E-06 \| 6.06E-06 \| \| ENSG00000083123 \| BCKDHB \| -0.71 \| 2.61E-06 \| 6.07E-06 \| \| ENSG00000179082 \|  \| -2.21 \| 2.64E-06 \| 6.13E-06 \| \| ENSG00000263513 \| FAM72C \| -2.38 \| 2.69E-06 \| 6.26E-06 \| \| ENSG00000263513 \| FAM72D \| -2.38 \| 2.69E-06 \| 6.26E-06 \| \| ENSG00000184508 \| HDDC3 \| -0.67 \| 2.70E-06 \| 6.27E-06 \| \| ENSG00000167397 \| VKORC1 \| -0.70 \| 2.70E-06 \| 6.27E-06 \| \| ENSG00000156463 \| SH3RF2 \| -5.89 \| 2.74E-06 \| 6.37E-06 \| \| ENSG00000147144 \| CCDC120 \| 0.90 \| 2.75E-06 \| 6.38E-06 \| \| ENSG00000111305 \| GSG1 \| -2.24 \| 2.79E-06 \| 6.47E-06 \| \| ENSG00000162927 \| PUS10 \| -0.60 \| 2.87E-06 \| 6.65E-06 \| \| ENSG00000102539 \| MLNR \| -1.22 \| 2.88E-06 \| 6.69E-06 \| \| ENSG00000188906 \| LRRK2 \| -0.79 \| 2.89E-06 \| 6.70E-06 \| \| ENSG00000275183 \| LENG9 \| 1.00 \| 2.89E-06 \| 6.70E-06 \| \| ENSG00000280161 \|  \| 1.48 \| 2.90E-06 \| 6.72E-06 \| \| ENSG00000119922 \| IFIT2 \| -2.55 \| 2.90E-06 \| 6.73E-06 \| \| ENSG00000116497 \| S100PBP \| -0.68 \| 2.91E-06 \| 6.75E-06 \| \| ENSG00000120324 \| PCDHB10 \| -3.73 \| 2.94E-06 \| 6.81E-06 \| \| ENSG00000170629 \| DPY19L2P2 \| -2.15 \| 3.03E-06 \| 7.01E-06 \| \| ENSG00000131773 \| KHDRBS3 \| 1.33 \| 3.04E-06 \| 7.03E-06 \| \| ENSG00000144935 \| TRPC1 \| 2.41 \| 3.04E-06 \| 7.03E-06 \| \| ENSG00000166171 \| DPCD \| -0.65 \| 3.10E-06 \| 7.17E-06 \| \| ENSG00000277228 \|  \| -2.64 \| 3.11E-06 \| 7.19E-06 \| \| ENSG00000159450 \| TCHH \| -1.55 \| 3.12E-06 \| 7.22E-06 \| \| ENSG00000234572 \| LINC01800 \| -3.25 \| 3.15E-06 \| 7.29E-06 \| \| ENSG00000240356 \| RPL23AP7 \| 0.63 \| 3.15E-06 \| 7.29E-06 \| \| ENSG00000255301 \|  \| 2.14 \| 3.20E-06 \| 7.39E-06 \| \| ENSG00000135740 \| SLC9A5 \| 1.16 \| 3.21E-06 \| 7.42E-06 \| \| ENSG00000004766 \| VPS50 \| -0.61 \| 3.23E-06 \| 7.47E-06 \| \| ENSG00000241859 \|  \| -1.30 \| 3.30E-06 \| 7.62E-06 \| \| ENSG00000135709 \| KIAA0513 \| -0.88 \| 3.34E-06 \| 7.70E-06 \| \| ENSG00000099256 \| PRTFDC1 \| -3.78 \| 3.36E-06 \| 7.76E-06 \| \| ENSG00000176641 \| RNF152 \| 1.50 \| 3.39E-06 \| 7.82E-06 \| \| ENSG00000187556 \| NANOS3 \| 3.06 \| 3.39E-06 \| 7.82E-06 \| \| ENSG00000172123 \| SLFN12 \| -1.13 \| 3.43E-06 \| 7.90E-06 \| \| ENSG00000272760 \|  \| -3.48 \| 3.43E-06 \| 7.92E-06 \| \| ENSG00000222043 \|  \| 1.26 \| 3.50E-06 \| 8.08E-06 \| \| ENSG00000134242 \| PTPN22 \| -1.42 \| 3.57E-06 \| 8.21E-06 \| \| ENSG00000196867 \| ZFP28 \| 0.64 \| 3.58E-06 \| 8.23E-06 \| \| ENSG00000186510 \| CLCNKA \| -1.53 \| 3.61E-06 \| 8.30E-06 \| \| ENSG00000018869 \| ZNF582 \| 1.36 \| 3.64E-06 \| 8.39E-06 \| \| ENSG00000243323 \| PTPRVP \| -2.45 \| 3.66E-06 \| 8.41E-06 \| \| ENSG00000134321 \| RSAD2 \| -3.07 \| 3.73E-06 \| 8.57E-06 \| \| ENSG00000265494 \|  \| 3.87 \| 3.73E-06 \| 8.58E-06 \| \| ENSG00000166455 \| C16orf46 \| 1.37 \| 3.79E-06 \| 8.70E-06 \| \| ENSG00000114646 \| CSPG5 \| 1.09 \| 3.92E-06 \| 9.01E-06 \| \| ENSG00000183873 \| SCN5A \| 2.56 \| 4.02E-06 \| 9.23E-06 \| \| ENSG00000187608 \| ISG15 \| -0.62 \| 4.10E-06 \| 9.42E-06 \| \| ENSG00000166220 \| TBATA \| -5.88 \| 4.13E-06 \| 9.46E-06 \| \| ENSG00000101349 \| PAK5 \| -1.99 \| 4.17E-06 \| 9.55E-06 \| \| ENSG00000260852 \| FBXL19-AS1 \| 0.79 \| 4.26E-06 \| 9.75E-06 \| \| ENSG00000271743 \|  \| 1.98 \| 4.36E-06 \| 9.98E-06 \| \| ENSG00000107562 \| CXCL12 \| -1.67 \| 4.43E-06 \| 1.01E-05 \| \| ENSG00000183785 \| TUBA8 \| -3.68 \| 4.44E-06 \| 1.01E-05 \| \| ENSG00000246898 \| LINC00920 \| -3.44 \| 4.44E-06 \| 1.01E-05 \| \| ENSG00000233680 \|  \| -3.03 \| 4.46E-06 \| 1.02E-05 \| \| ENSG00000180336 \| MEIOC \| -1.30 \| 4.48E-06 \| 1.02E-05 \| \| ENSG00000198521 \| ZNF43 \| -0.82 \| 4.50E-06 \| 1.03E-05 \| \| ENSG00000107282 \| APBA1 \| -1.09 \| 4.51E-06 \| 1.03E-05 \| \| ENSG00000112655 \| PTK7 \| -1.52 \| 4.55E-06 \| 1.04E-05 \| \| ENSG00000163673 \| DCLK3 \| -3.68 \| 4.55E-06 \| 1.04E-05 \| \| ENSG00000247134 \|  \| -0.80 \| 4.60E-06 \| 1.05E-05 \| \| ENSG00000147799 \| ARHGAP39 \| 0.69 \| 4.70E-06 \| 1.07E-05 \| \| ENSG00000109775 \| UFSP2 \| -0.75 \| 4.88E-06 \| 1.11E-05 \| \| ENSG00000115363 \| EVA1A \| 2.50 \| 4.93E-06 \| 1.12E-05 \| \| ENSG00000169857 \| AVEN \| 0.61 \| 5.17E-06 \| 1.17E-05 \| \| ENSG00000136943 \| CTSV \| -2.73 \| 5.27E-06 \| 1.20E-05 \| \| ENSG00000100271 \| TTLL1 \| -0.68 \| 5.30E-06 \| 1.20E-05 \| \| ENSG00000063180 \| CA11 \| -0.81 \| 5.30E-06 \| 1.20E-05 \| \| ENSG00000271857 \|  \| 0.68 \| 5.33E-06 \| 1.21E-05 \| \| ENSG00000257453 \|  \| 0.64 \| 5.35E-06 \| 1.21E-05 \| \| ENSG00000133460 \| SLC2A11 \| -1.16 \| 5.43E-06 \| 1.23E-05 \| \| ENSG00000080561 \| MID2 \| -0.83 \| 5.44E-06 \| 1.23E-05 \| \| ENSG00000152465 \| NMT2 \| -0.64 \| 5.45E-06 \| 1.23E-05 \| \| ENSG00000126970 \| ZC4H2 \| -1.14 \| 5.60E-06 \| 1.27E-05 \| \| ENSG00000260898 \| ADPGK-AS1 \| 0.91 \| 5.72E-06 \| 1.29E-05 \| \| ENSG00000179532 \| DNHD1 \| -0.84 \| 5.73E-06 \| 1.30E-05 \| \| ENSG00000198846 \| TOX \| -2.55 \| 5.81E-06 \| 1.31E-05 \| \| ENSG00000178075 \| GRAMD1C \| 1.81 \| 5.87E-06 \| 1.33E-05 \| \| ENSG00000180834 \| MAP6D1 \| -1.47 \| 5.88E-06 \| 1.33E-05 \| \| ENSG00000165060 \| FXN \| -0.64 \| 5.90E-06 \| 1.33E-05 \| \| ENSG00000168062 \| BATF2 \| -1.25 \| 6.15E-06 \| 1.39E-05 \| \| ENSG00000029639 \| TFB1M \| 0.59 \| 6.18E-06 \| 1.39E-05 \| \| ENSG00000134824 \| FADS2 \| -0.70 \| 6.22E-06 \| 1.40E-05 \| \| ENSG00000129990 \| SYT5 \| 2.41 \| 6.36E-06 \| 1.43E-05 \| \| ENSG00000166816 \| LDHD \| -2.24 \| 6.38E-06 \| 1.44E-05 \| \| ENSG00000228065 \|  \| -2.34 \| 6.40E-06 \| 1.44E-05 \| \| ENSG00000166321 \| NUDT13 \| -1.24 \| 6.51E-06 \| 1.46E-05 \| \| ENSG00000182185 \| RAD51B \| -1.06 \| 6.51E-06 \| 1.46E-05 \| \| ENSG00000261061 \|  \| -0.68 \| 6.69E-06 \| 1.50E-05 \| \| ENSG00000211829 \|  \| 2.58 \| 6.81E-06 \| 1.53E-05 \| \| ENSG00000110844 \| PRPF40B \| -1.07 \| 6.84E-06 \| 1.54E-05 \| \| ENSG00000139508 \| SLC46A3 \| -0.90 \| 6.88E-06 \| 1.55E-05 \| \| ENSG00000180611 \| MB21D2 \| 2.52 \| 6.91E-06 \| 1.55E-05 \| \| ENSG00000149516 \| MS4A3 \| -5.74 \| 6.97E-06 \| 1.56E-05 \| \| ENSG00000106511 \| MEOX2 \| 2.30 \| 6.98E-06 \| 1.57E-05 \| \| ENSG00000204228 \| HSD17B8 \| -2.49 \| 6.99E-06 \| 1.57E-05 \| \| ENSG00000126778 \| SIX1 \| 1.15 \| 7.17E-06 \| 1.61E-05 \| \| ENSG00000104970 \| KIR3DX1 \| -1.74 \| 7.32E-06 \| 1.64E-05 \| \| ENSG00000197763 \| TXNRD3 \| -0.85 \| 7.35E-06 \| 1.65E-05 \| \| ENSG00000197763 \| TXNRD3NB \| -0.85 \| 7.35E-06 \| 1.65E-05 \| \| ENSG00000108309 \| RUNDC3A \| -1.94 \| 7.36E-06 \| 1.65E-05 \| \| ENSG00000215146 \| LOC441666 \| -1.92 \| 7.38E-06 \| 1.65E-05 \| \| ENSG00000251393 \|  \| 1.25 \| 7.40E-06 \| 1.66E-05 \| \| ENSG00000179058 \| C9orf50 \| 2.18 \| 7.49E-06 \| 1.67E-05 \| \| ENSG00000253389 \|  \| -2.48 \| 7.54E-06 \| 1.69E-05 \| \| ENSG00000237773 \|  \| 1.43 \| 7.58E-06 \| 1.69E-05 \| \| ENSG00000258738 \|  \| 0.64 \| 7.60E-06 \| 1.70E-05 \| \| ENSG00000169288 \| MRPL1 \| -0.64 \| 7.61E-06 \| 1.70E-05 \| \| ENSG00000121903 \| ZSCAN20 \| 0.63 \| 7.62E-06 \| 1.70E-05 \| \| ENSG00000117519 \| CNN3 \| -1.34 \| 7.65E-06 \| 1.71E-05 \| \| ENSG00000253326 \|  \| 2.52 \| 7.69E-06 \| 1.72E-05 \| \| ENSG00000227827 \|  \| 0.76 \| 7.81E-06 \| 1.74E-05 \| \| ENSG00000228486 \| C2orf92 \| -0.60 \| 7.89E-06 \| 1.76E-05 \| \| ENSG00000066923 \| STAG3 \| -4.82 \| 7.98E-06 \| 1.78E-05 \| \| ENSG00000162494 \| LRRC38 \| 2.87 \| 7.99E-06 \| 1.78E-05 \| \| ENSG00000237525 \|  \| 1.62 \| 8.21E-06 \| 1.83E-05 \| \| ENSG00000227051 \| C14orf132 \| -1.74 \| 8.24E-06 \| 1.83E-05 \| \| ENSG00000056050 \| HPF1 \| -0.59 \| 8.26E-06 \| 1.84E-05 \| \| ENSG00000197619 \| ZNF615 \| 0.73 \| 8.40E-06 \| 1.87E-05 \| \| ENSG00000184083 \| FAM120C \| 0.61 \| 8.42E-06 \| 1.87E-05 \| \| ENSG00000172828 \| CES3 \| -3.32 \| 8.43E-06 \| 1.87E-05 \| \| ENSG00000272625 \|  \| 1.36 \| 8.46E-06 \| 1.88E-05 \| \| ENSG00000171533 \| MAP6 \| 1.50 \| 8.70E-06 \| 1.93E-05 \| \| ENSG00000144619 \| CNTN4 \| -1.46 \| 8.99E-06 \| 1.99E-05 \| \| ENSG00000095637 \| SORBS1 \| 1.51 \| 9.06E-06 \| 2.01E-05 \| \| ENSG00000198513 \| ATL1 \| -2.47 \| 9.09E-06 \| 2.02E-05 \| \| ENSG00000229474 \| PATL2 \| -0.76 \| 9.09E-06 \| 2.02E-05 \| \| ENSG00000120696 \| KBTBD7 \| -1.15 \| 9.20E-06 \| 2.04E-05 \| \| ENSG00000134508 \| CABLES1 \| -2.19 \| 9.27E-06 \| 2.05E-05 \| \| ENSG00000125843 \| AP5S1 \| 0.62 \| 9.29E-06 \| 2.06E-05 \| \| ENSG00000245812 \| LINC02202 \| 4.13 \| 9.40E-06 \| 2.08E-05 \| \| ENSG00000182318 \| ZSCAN22 \| 0.72 \| 9.44E-06 \| 2.09E-05 \| \| ENSG00000185985 \| SLITRK2 \| 0.64 \| 9.50E-06 \| 2.10E-05 \| \| ENSG00000042062 \| RIPOR3 \| -2.96 \| 9.65E-06 \| 2.13E-05 \| \| ENSG00000259928 \|  \| -2.58 \| 9.81E-06 \| 2.17E-05 \| \| ENSG00000230453 \| ANKRD18B \| 1.16 \| 9.87E-06 \| 2.18E-05 \| \| ENSG00000164331 \| ANKRA2 \| 0.67 \| 9.90E-06 \| 2.19E-05 \| \| ENSG00000136231 \| IGF2BP3 \| -1.48 \| 1.01E-05 \| 2.23E-05 \| \| ENSG00000068137 \| PLEKHH3 \| -1.04 \| 1.01E-05 \| 2.23E-05 \| \| ENSG00000180855 \| ZNF443 \| -2.08 \| 1.02E-05 \| 2.26E-05 \| \| ENSG00000112964 \| GHR \| 3.04 \| 1.04E-05 \| 2.29E-05 \| \| ENSG00000259985 \|  \| -1.74 \| 1.05E-05 \| 2.31E-05 \| \| ENSG00000197497 \| ZNF665 \| -4.79 \| 1.08E-05 \| 2.38E-05 \| \| ENSG00000166788 \| SAAL1 \| -0.66 \| 1.10E-05 \| 2.42E-05 \| \| ENSG00000249741 \|  \| 2.57 \| 1.10E-05 \| 2.42E-05 \| \| ENSG00000266389 \|  \| 0.90 \| 1.11E-05 \| 2.44E-05 \| \| ENSG00000054690 \| PLEKHH1 \| -1.10 \| 1.11E-05 \| 2.44E-05 \| \| ENSG00000163884 \| KLF15 \| 1.48 \| 1.11E-05 \| 2.45E-05 \| \| ENSG00000279207 \|  \| 1.31 \| 1.12E-05 \| 2.47E-05 \| \| ENSG00000277969 \|  \| 0.68 \| 1.14E-05 \| 2.51E-05 \| \| ENSG00000106080 \| FKBP14 \| -0.66 \| 1.15E-05 \| 2.52E-05 \| \| ENSG00000214870 \| LOC441204 \| -3.99 \| 1.15E-05 \| 2.53E-05 \| \| ENSG00000236871 \| LINC00106 \| -0.78 \| 1.15E-05 \| 2.53E-05 \| \| ENSG00000241058 \| NSUN6 \| -1.11 \| 1.16E-05 \| 2.54E-05 \| \| ENSG00000166813 \| KIF7 \| -2.77 \| 1.16E-05 \| 2.55E-05 \| \| ENSG00000169704 \| GP9 \| -2.27 \| 1.17E-05 \| 2.56E-05 \| \| ENSG00000163607 \| GTPBP8 \| -0.69 \| 1.17E-05 \| 2.57E-05 \| \| ENSG00000162836 \| ACP6 \| -1.54 \| 1.18E-05 \| 2.59E-05 \| \| ENSG00000203797 \| DDO \| -2.24 \| 1.19E-05 \| 2.61E-05 \| \| ENSG00000173404 \| INSM1 \| 2.11 \| 1.23E-05 \| 2.69E-05 \| \| ENSG00000185697 \| MYBL1 \| -0.85 \| 1.24E-05 \| 2.71E-05 \| \| ENSG00000074219 \| TEAD2 \| -1.24 \| 1.26E-05 \| 2.76E-05 \| \| ENSG00000270021 \|  \| 1.12 \| 1.28E-05 \| 2.81E-05 \| \| ENSG00000167562 \| ZNF701 \| -0.68 \| 1.32E-05 \| 2.89E-05 \| \| ENSG00000182621 \| PLCB1 \| 1.36 \| 1.35E-05 \| 2.94E-05 \| \| ENSG00000182621 \| LOC105372522 \| 1.36 \| 1.35E-05 \| 2.94E-05 \| \| ENSG00000120328 \| PCDHB12 \| -2.35 \| 1.37E-05 \| 2.99E-05 \| \| ENSG00000111181 \| SLC6A12 \| -2.74 \| 1.45E-05 \| 3.16E-05 \| \| ENSG00000124257 \| NEURL2 \| -1.02 \| 1.46E-05 \| 3.18E-05 \| \| ENSG00000260876 \| LINC01229 \| -3.21 \| 1.46E-05 \| 3.18E-05 \| \| ENSG00000112137 \| PHACTR1 \| -1.18 \| 1.46E-05 \| 3.19E-05 \| \| ENSG00000169918 \| OTUD7A \| 1.93 \| 1.46E-05 \| 3.19E-05 \| \| ENSG00000283633 \|  \| -3.34 \| 1.47E-05 \| 3.21E-05 \| \| ENSG00000178636 \| LOC101929762 \| 4.80 \| 1.48E-05 \| 3.23E-05 \| \| ENSG00000135899 \| SP110 \| -0.90 \| 1.50E-05 \| 3.26E-05 \| \| ENSG00000159871 \| LYPD5 \| -2.83 \| 1.50E-05 \| 3.27E-05 \| \| ENSG00000165912 \| PACSIN3 \| -0.60 \| 1.51E-05 \| 3.30E-05 \| \| ENSG00000280987 \| MATR3 \| -1.13 \| 1.53E-05 \| 3.33E-05 \| \| ENSG00000269386 \| RAB11B-AS1 \| 0.75 \| 1.54E-05 \| 3.34E-05 \| \| ENSG00000177469 \| CAVIN1 \| -0.88 \| 1.54E-05 \| 3.34E-05 \| \| ENSG00000231106 \| LINC01436 \| -1.19 \| 1.56E-05 \| 3.40E-05 \| \| ENSG00000144199 \| FAHD2B \| -1.61 \| 1.56E-05 \| 3.40E-05 \| \| ENSG00000136010 \| ALDH1L2 \| -0.64 \| 1.61E-05 \| 3.51E-05 \| \| ENSG00000206538 \| VGLL3 \| 2.42 \| 1.62E-05 \| 3.52E-05 \| \| ENSG00000261512 \|  \| 0.61 \| 1.65E-05 \| 3.58E-05 \| \| ENSG00000197182 \| MIRLET7BHG \| -0.77 \| 1.65E-05 \| 3.59E-05 \| \| ENSG00000163331 \| DAPL1 \| -5.57 \| 1.66E-05 \| 3.60E-05 \| \| ENSG00000167874 \| TMEM88 \| 1.79 \| 1.68E-05 \| 3.65E-05 \| \| ENSG00000253671 \|  \| -3.50 \| 1.69E-05 \| 3.66E-05 \| \| ENSG00000150637 \| CD226 \| -0.74 \| 1.70E-05 \| 3.69E-05 \| \| ENSG00000273486 \|  \| -0.88 \| 1.71E-05 \| 3.70E-05 \| \| ENSG00000181544 \| FANCB \| -2.08 \| 1.72E-05 \| 3.72E-05 \| \| ENSG00000105427 \| CNFN \| -1.97 \| 1.74E-05 \| 3.77E-05 \| \| ENSG00000267287 \|  \| -0.95 \| 1.74E-05 \| 3.78E-05 \| \| ENSG00000088836 \| SLC4A11 \| -0.71 \| 1.77E-05 \| 3.84E-05 \| \| ENSG00000158079 \| PTPDC1 \| -0.62 \| 1.77E-05 \| 3.84E-05 \| \| ENSG00000145850 \| TIMD4 \| -2.53 \| 1.77E-05 \| 3.84E-05 \| \| ENSG00000159055 \| MIS18A \| -0.70 \| 1.78E-05 \| 3.85E-05 \| \| ENSG00000247746 \| USP51 \| -0.75 \| 1.83E-05 \| 3.97E-05 \| \| ENSG00000228058 \| LINC01736 \| -2.72 \| 1.84E-05 \| 3.97E-05 \| \| ENSG00000164976 \| MYORG \| -1.41 \| 1.89E-05 \| 4.07E-05 \| \| ENSG00000189136 \| UBE2Q2P1 \| -1.84 \| 1.90E-05 \| 4.11E-05 \| \| ENSG00000160588 \| MPZL3 \| -1.17 \| 1.92E-05 \| 4.14E-05 \| \| ENSG00000258077 \|  \| -1.77 \| 1.93E-05 \| 4.17E-05 \| \| ENSG00000125285 \| SOX21 \| 3.23 \| 1.94E-05 \| 4.19E-05 \| \| ENSG00000105227 \| PRX \| 0.83 \| 1.95E-05 \| 4.20E-05 \| \| ENSG00000177398 \| UMODL1 \| -3.51 \| 1.95E-05 \| 4.20E-05 \| \| ENSG00000271882 \|  \| 1.12 \| 1.96E-05 \| 4.22E-05 \| \| ENSG00000156509 \| FBXO43 \| -2.12 \| 1.97E-05 \| 4.25E-05 \| \| ENSG00000109686 \| SH3D19 \| -0.68 \| 2.02E-05 \| 4.35E-05 \| \| ENSG00000102794 \| ACOD1 \| 2.08 \| 2.04E-05 \| 4.38E-05 \| \| ENSG00000215784 \| FAM72D \| -1.61 \| 2.04E-05 \| 4.39E-05 \| \| ENSG00000173013 \| CCDC96 \| 0.88 \| 2.09E-05 \| 4.49E-05 \| \| ENSG00000101417 \| PXMP4 \| -0.67 \| 2.09E-05 \| 4.50E-05 \| \| ENSG00000154529 \| CNTNAP3B \| -1.13 \| 2.13E-05 \| 4.58E-05 \| \| ENSG00000198929 \| NOS1AP \| 0.67 \| 2.14E-05 \| 4.60E-05 \| \| ENSG00000185100 \| ADSSL1 \| -1.26 \| 2.14E-05 \| 4.60E-05 \| \| ENSG00000253968 \|  \| -1.36 \| 2.17E-05 \| 4.67E-05 \| \| ENSG00000120457 \| KCNJ5 \| -1.93 \| 2.18E-05 \| 4.69E-05 \| \| ENSG00000110079 \| MS4A4A \| -5.32 \| 2.19E-05 \| 4.69E-05 \| \| ENSG00000075213 \| SEMA3A \| 0.93 \| 2.19E-05 \| 4.70E-05 \| \| ENSG00000173253 \| DMRT2 \| 0.68 \| 2.22E-05 \| 4.76E-05 \| \| ENSG00000255299 \|  \| 2.51 \| 2.31E-05 \| 4.94E-05 \| \| ENSG00000231535 \| LINC00278 \| -1.14 \| 2.31E-05 \| 4.95E-05 \| \| ENSG00000257093 \| KIAA1147 \| 0.59 \| 2.33E-05 \| 4.99E-05 \| \| ENSG00000227082 \| LOC107985194 \| -1.74 \| 2.38E-05 \| 5.10E-05 \| \| ENSG00000261662 \|  \| 1.59 \| 2.39E-05 \| 5.12E-05 \| \| ENSG00000154096 \| THY1 \| -1.13 \| 2.40E-05 \| 5.13E-05 \| \| ENSG00000162383 \| SLC1A7 \| -5.30 \| 2.47E-05 \| 5.28E-05 \| \| ENSG00000160439 \| RDH13 \| -0.92 \| 2.50E-05 \| 5.34E-05 \| \| ENSG00000167555 \| ZNF528 \| -0.67 \| 2.52E-05 \| 5.38E-05 \| \| ENSG00000100181 \| TPTEP1 \| -2.54 \| 2.53E-05 \| 5.40E-05 \| \| ENSG00000100181 \| LOC112268292 \| -2.54 \| 2.53E-05 \| 5.40E-05 \| \| ENSG00000203688 \| LINC02487 \| -5.24 \| 2.53E-05 \| 5.41E-05 \| \| ENSG00000272686 \|  \| 0.82 \| 2.64E-05 \| 5.62E-05 \| \| ENSG00000174403 \| MIR1-1HG-AS1 \| -5.28 \| 2.66E-05 \| 5.67E-05 \| \| ENSG00000213160 \| KLHL23 \| -1.05 \| 2.67E-05 \| 5.69E-05 \| \| ENSG00000213160 \| PHOSPHO2-KLHL23 \| -1.05 \| 2.67E-05 \| 5.69E-05 \| \| ENSG00000215302 \|  \| -1.92 \| 2.69E-05 \| 5.73E-05 \| \| ENSG00000111261 \| MANSC1 \| -2.06 \| 2.70E-05 \| 5.75E-05 \| \| ENSG00000260086 \|  \| -1.42 \| 2.72E-05 \| 5.79E-05 \| \| ENSG00000122870 \| BICC1 \| 1.10 \| 2.72E-05 \| 5.79E-05 \| \| ENSG00000128602 \| SMO \| -2.94 \| 2.79E-05 \| 5.93E-05 \| \| ENSG00000256546 \| LOC100506691 \| -1.99 \| 2.79E-05 \| 5.93E-05 \| \| ENSG00000188820 \| CALHM6 \| -1.94 \| 2.80E-05 \| 5.95E-05 \| \| ENSG00000156453 \| PCDH1 \| -0.78 \| 2.80E-05 \| 5.95E-05 \| \| ENSG00000273855 \|  \| -1.58 \| 2.85E-05 \| 6.06E-05 \| \| ENSG00000111644 \| ACRBP \| -1.18 \| 2.90E-05 \| 6.16E-05 \| \| ENSG00000273893 \|  \| 1.66 \| 2.92E-05 \| 6.22E-05 \| \| ENSG00000112414 \| ADGRG6 \| -2.43 \| 2.94E-05 \| 6.24E-05 \| \| ENSG00000137198 \| GMPR \| -1.16 \| 2.94E-05 \| 6.25E-05 \| \| ENSG00000115392 \| FANCL \| -0.86 \| 2.98E-05 \| 6.33E-05 \| \| ENSG00000037280 \| FLT4 \| -4.56 \| 3.06E-05 \| 6.49E-05 \| \| ENSG00000231123 \|  \| 3.99 \| 3.09E-05 \| 6.56E-05 \| \| ENSG00000185745 \| IFIT1 \| -2.25 \| 3.10E-05 \| 6.57E-05 \| \| ENSG00000267980 \|  \| 1.92 \| 3.15E-05 \| 6.68E-05 \| \| ENSG00000222022 \|  \| -3.85 \| 3.20E-05 \| 6.78E-05 \| \| ENSG00000230778 \| ANKRD63 \| -1.00 \| 3.25E-05 \| 6.88E-05 \| \| ENSG00000203747 \| FCGR3A \| -2.36 \| 3.26E-05 \| 6.91E-05 \| \| ENSG00000198039 \| ZNF273 \| -0.69 \| 3.30E-05 \| 6.99E-05 \| \| ENSG00000126803 \| HSPA2 \| -2.02 \| 3.32E-05 \| 7.03E-05 \| \| ENSG00000151287 \| TEX30 \| -0.69 \| 3.34E-05 \| 7.07E-05 \| \| ENSG00000082175 \| PGR \| -1.14 \| 3.36E-05 \| 7.10E-05 \| \| ENSG00000133027 \| PEMT \| 0.62 \| 3.36E-05 \| 7.12E-05 \| \| ENSG00000135314 \| KHDC1 \| -1.59 \| 3.37E-05 \| 7.12E-05 \| \| ENSG00000002016 \| RAD52 \| 0.89 \| 3.41E-05 \| 7.21E-05 \| \| ENSG00000183242 \| WT1-AS \| 0.99 \| 3.44E-05 \| 7.26E-05 \| \| ENSG00000157554 \| ERG \| 1.70 \| 3.54E-05 \| 7.46E-05 \| \| ENSG00000187122 \| SLIT1 \| -2.28 \| 3.57E-05 \| 7.53E-05 \| \| ENSG00000121753 \| ADGRB2 \| -2.74 \| 3.59E-05 \| 7.57E-05 \| \| ENSG00000196711 \| ALKAL1 \| 1.08 \| 3.59E-05 \| 7.57E-05 \| \| ENSG00000241163 \| LINC00877 \| -1.40 \| 3.62E-05 \| 7.63E-05 \| \| ENSG00000106688 \| SLC1A1 \| 1.19 \| 3.63E-05 \| 7.65E-05 \| \| ENSG00000281162 \|  \| 1.46 \| 3.63E-05 \| 7.65E-05 \| \| ENSG00000249395 \| CASC9 \| -1.46 \| 3.65E-05 \| 7.69E-05 \| \| ENSG00000125814 \| NAPB \| 0.67 \| 3.72E-05 \| 7.84E-05 \| \| ENSG00000130222 \| GADD45G \| -0.98 \| 3.74E-05 \| 7.88E-05 \| \| ENSG00000155719 \| OTOA \| -2.42 \| 3.77E-05 \| 7.94E-05 \| \| ENSG00000245688 \|  \| 1.98 \| 3.78E-05 \| 7.96E-05 \| \| ENSG00000160323 \| ADAMTS13 \| -0.88 \| 3.82E-05 \| 8.03E-05 \| \| ENSG00000167680 \| SEMA6B \| 1.01 \| 3.84E-05 \| 8.08E-05 \| \| ENSG00000164638 \| SLC29A4 \| -0.78 \| 3.86E-05 \| 8.11E-05 \| \| ENSG00000228903 \|  \| -0.88 \| 3.90E-05 \| 8.20E-05 \| \| ENSG00000092529 \| CAPN3 \| -1.70 \| 3.96E-05 \| 8.32E-05 \| \| ENSG00000056558 \| TRAF1 \| 0.77 \| 4.20E-05 \| 8.81E-05 \| \| ENSG00000182566 \| CLEC4G \| -3.78 \| 4.21E-05 \| 8.81E-05 \| \| ENSG00000204262 \| COL5A2 \| -1.69 \| 4.23E-05 \| 8.85E-05 \| \| ENSG00000151692 \| RNF144A \| -0.87 \| 4.25E-05 \| 8.90E-05 \| \| ENSG00000203999 \| LINC01270 \| 1.52 \| 4.28E-05 \| 8.95E-05 \| \| ENSG00000102057 \| KCND1 \| -1.22 \| 4.29E-05 \| 8.97E-05 \| \| ENSG00000141198 \| TOM1L1 \| 0.63 \| 4.30E-05 \| 8.99E-05 \| \| ENSG00000140092 \| FBLN5 \| -2.78 \| 4.33E-05 \| 9.06E-05 \| \| ENSG00000183798 \| EMILIN3 \| -3.37 \| 4.35E-05 \| 9.10E-05 \| \| ENSG00000177335 \| C8orf31 \| -3.40 \| 4.37E-05 \| 9.14E-05 \| \| ENSG00000271856 \| LINC01215 \| 1.26 \| 4.38E-05 \| 9.16E-05 \| \| ENSG00000144395 \| CCDC150 \| -1.31 \| 4.47E-05 \| 9.34E-05 \| \| ENSG00000272931 \|  \| -2.12 \| 4.48E-05 \| 9.36E-05 \| \| ENSG00000278330 \|  \| 0.85 \| 4.51E-05 \| 9.42E-05 \| \| ENSG00000168917 \| SLC35G2 \| -1.23 \| 4.53E-05 \| 9.46E-05 \| \| ENSG00000204599 \| TRIM39 \| 0.62 \| 4.60E-05 \| 9.60E-05 \| \| ENSG00000197062 \| ZSCAN26 \| -1.17 \| 4.62E-05 \| 9.65E-05 \| \| ENSG00000254528 \|  \| -4.46 \| 4.64E-05 \| 9.68E-05 \| \| ENSG00000006016 \| CRLF1 \| 1.05 \| 4.65E-05 \| 9.69E-05 \| \| ENSG00000135469 \| COQ10A \| 0.66 \| 4.70E-05 \| 9.80E-05 \| \| ENSG00000224687 \| RASAL2-AS1 \| 0.84 \| 4.70E-05 \| 9.80E-05 \| \| ENSG00000272269 \| LOC105374952 \| 0.71 \| 4.72E-05 \| 9.83E-05 \| \| ENSG00000161860 \| SYCE2 \| -2.76 \| 4.79E-05 \| 9.97E-05 \| \| ENSG00000230489 \| VAV3-AS1 \| 1.05 \| 4.82E-05 \| 1.00E-04 \| \| ENSG00000187193 \| MT1X \| 1.25 \| 4.83E-05 \| 1.01E-04 \| \| ENSG00000182612 \| TSPAN10 \| -1.41 \| 4.87E-05 \| 1.01E-04 \| \| ENSG00000227066 \|  \| -1.60 \| 4.89E-05 \| 1.02E-04 \| \| ENSG00000236154 \|  \| -3.38 \| 4.92E-05 \| 1.02E-04 \| \| ENSG00000183092 \| BEGAIN \| -1.31 \| 4.97E-05 \| 1.03E-04 \| \| ENSG00000225177 \| FLJ46906 \| -1.50 \| 5.10E-05 \| 1.06E-04 \| \| ENSG00000224413 \| LOC105372842 \| 1.72 \| 5.11E-05 \| 1.06E-04 \| \| ENSG00000135898 \| GPR55 \| -2.38 \| 5.12E-05 \| 1.06E-04 \| \| ENSG00000165716 \| DIPK1B \| -2.03 \| 5.13E-05 \| 1.06E-04 \| \| ENSG00000214575 \| CPEB1 \| -1.84 \| 5.22E-05 \| 1.08E-04 \| \| ENSG00000176349 \|  \| 1.24 \| 5.26E-05 \| 1.09E-04 \| \| ENSG00000082482 \| KCNK2 \| 2.60 \| 5.26E-05 \| 1.09E-04 \| \| ENSG00000100147 \| CCDC134 \| -0.87 \| 5.32E-05 \| 1.10E-04 \| \| ENSG00000149548 \| CCDC15 \| -0.82 \| 5.32E-05 \| 1.10E-04 \| \| ENSG00000248211 \|  \| 2.38 \| 5.32E-05 \| 1.10E-04 \| \| ENSG00000136098 \| NEK3 \| -0.72 \| 5.34E-05 \| 1.10E-04 \| \| ENSG00000197927 \| C2orf27A \| 0.84 \| 5.48E-05 \| 1.13E-04 \| \| ENSG00000111319 \| SCNN1A \| -2.18 \| 5.50E-05 \| 1.13E-04 \| \| ENSG00000172399 \| MYOZ2 \| -0.91 \| 5.53E-05 \| 1.14E-04 \| \| ENSG00000105492 \| SIGLEC6 \| -2.40 \| 5.54E-05 \| 1.14E-04 \| \| ENSG00000169715 \| MT1E \| 0.76 \| 5.55E-05 \| 1.15E-04 \| \| ENSG00000158296 \| SLC13A3 \| 1.48 \| 5.58E-05 \| 1.15E-04 \| \| ENSG00000204682 \| CASC10 \| -3.39 \| 5.71E-05 \| 1.18E-04 \| \| ENSG00000152582 \| SPEF2 \| -1.38 \| 5.76E-05 \| 1.19E-04 \| \| ENSG00000166432 \| ZMAT1 \| -0.64 \| 5.76E-05 \| 1.19E-04 \| \| ENSG00000133808 \|  \| 0.60 \| 5.85E-05 \| 1.20E-04 \| \| ENSG00000234373 \|  \| 2.11 \| 5.91E-05 \| 1.22E-04 \| \| ENSG00000214402 \| LCNL1 \| -1.82 \| 6.02E-05 \| 1.24E-04 \| \| ENSG00000271981 \|  \| -2.88 \| 6.17E-05 \| 1.27E-04 \| \| ENSG00000196267 \| ZNF836 \| -1.13 \| 6.34E-05 \| 1.30E-04 \| \| ENSG00000100629 \| CEP128 \| -0.75 \| 6.34E-05 \| 1.30E-04 \| \| ENSG00000131042 \| LILRB2 \| -2.22 \| 6.44E-05 \| 1.32E-04 \| \| ENSG00000185666 \| SYN3 \| -2.12 \| 6.56E-05 \| 1.35E-04 \| \| ENSG00000101104 \| PABPC1L \| -0.59 \| 6.60E-05 \| 1.35E-04 \| \| ENSG00000169908 \| TM4SF1 \| 1.32 \| 6.76E-05 \| 1.38E-04 \| \| ENSG00000130528 \| HRC \| -3.32 \| 6.82E-05 \| 1.40E-04 \| \| ENSG00000180423 \| HARBI1 \| 0.87 \| 6.84E-05 \| 1.40E-04 \| \| ENSG00000170542 \| SERPINB9 \| -1.74 \| 6.86E-05 \| 1.40E-04 \| \| ENSG00000236747 \| LINC01282 \| 1.51 \| 6.86E-05 \| 1.40E-04 \| \| ENSG00000151413 \| NUBPL \| -0.87 \| 6.89E-05 \| 1.41E-04 \| \| ENSG00000272655 \|  \| 0.59 \| 7.03E-05 \| 1.44E-04 \| \| ENSG00000141510 \| TP53 \| -0.72 \| 7.04E-05 \| 1.44E-04 \| \| ENSG00000196378 \| ZNF34 \| 0.68 \| 7.11E-05 \| 1.45E-04 \| \| ENSG00000168916 \| ZNF608 \| -1.13 \| 7.21E-05 \| 1.47E-04 \| \| ENSG00000130590 \| SAMD10 \| -0.68 \| 7.22E-05 \| 1.48E-04 \| \| ENSG00000138468 \| SENP7 \| -0.62 \| 7.27E-05 \| 1.48E-04 \| \| ENSG00000213023 \| SYT3 \| -0.60 \| 7.33E-05 \| 1.50E-04 \| \| ENSG00000148832 \| PAOX \| -1.97 \| 7.39E-05 \| 1.51E-04 \| \| ENSG00000138316 \| ADAMTS14 \| -1.19 \| 7.55E-05 \| 1.54E-04 \| \| ENSG00000151967 \| SCHIP1 \| 1.06 \| 7.56E-05 \| 1.54E-04 \| \| ENSG00000246273 \| SBF2-AS1 \| -0.83 \| 7.60E-05 \| 1.55E-04 \| \| ENSG00000212694 \| LINC01089 \| -0.60 \| 7.65E-05 \| 1.56E-04 \| \| ENSG00000277561 \| GOLGA8IP \| -4.35 \| 7.66E-05 \| 1.56E-04 \| \| ENSG00000256633 \|  \| -2.29 \| 7.74E-05 \| 1.58E-04 \| \| ENSG00000138772 \| ANXA3 \| -5.00 \| 7.76E-05 \| 1.58E-04 \| \| ENSG00000198825 \| INPP5F \| -0.59 \| 7.76E-05 \| 1.58E-04 \| \| ENSG00000100433 \| KCNK10 \| -3.67 \| 7.97E-05 \| 1.62E-04 \| \| ENSG00000118402 \| ELOVL4 \| 1.48 \| 8.22E-05 \| 1.67E-04 \| \| ENSG00000168405 \| CMAHP \| -3.07 \| 8.22E-05 \| 1.67E-04 \| \| ENSG00000168405 \| LOC101928663 \| -3.07 \| 8.22E-05 \| 1.67E-04 \| \| ENSG00000105136 \| ZNF419 \| -0.83 \| 8.27E-05 \| 1.68E-04 \| \| ENSG00000259674 \|  \| -2.83 \| 8.44E-05 \| 1.71E-04 \| \| ENSG00000128040 \| SPINK2 \| 1.95 \| 8.46E-05 \| 1.72E-04 \| \| ENSG00000258920 \| FOXN3-AS1 \| -1.78 \| 8.65E-05 \| 1.75E-04 \| \| ENSG00000266835 \| GAPLINC \| -2.00 \| 8.66E-05 \| 1.76E-04 \| \| ENSG00000106399 \| RPA3 \| -0.70 \| 8.67E-05 \| 1.76E-04 \| \| ENSG00000259162 \|  \| -2.01 \| 8.73E-05 \| 1.77E-04 \| \| ENSG00000177340 \| FLJ13224 \| 1.09 \| 8.79E-05 \| 1.78E-04 \| \| ENSG00000175426 \| PCSK1 \| 2.37 \| 9.88E-05 \| 2.00E-04 \| \| ENSG00000111674 \| ENO2 \| 0.71 \| 1.00E-04 \| 2.02E-04 \| \| ENSG00000138030 \| KHK \| -1.60 \| 1.03E-04 \| 2.08E-04 \| \| ENSG00000091622 \| PITPNM3 \| -0.79 \| 1.03E-04 \| 2.08E-04 \| \| ENSG00000114107 \| CEP70 \| -1.46 \| 1.04E-04 \| 2.09E-04 \| \| ENSG00000129514 \| FOXA1 \| 2.17 \| 1.05E-04 \| 2.12E-04 \| \| ENSG00000181965 \| NEUROG1 \| 1.90 \| 1.06E-04 \| 2.14E-04 \| \| ENSG00000183208 \| GDPGP1 \| -0.94 \| 1.07E-04 \| 2.15E-04 \| \| ENSG00000144596 \| GRIP2 \| -1.29 \| 1.07E-04 \| 2.16E-04 \| \| ENSG00000135373 \| EHF \| 2.63 \| 1.08E-04 \| 2.17E-04 \| \| ENSG00000160179 \| ABCG1 \| -0.72 \| 1.09E-04 \| 2.19E-04 \| \| ENSG00000270372 \|  \| -1.93 \| 1.09E-04 \| 2.20E-04 \| \| ENSG00000111907 \| TPD52L1 \| -3.63 \| 1.09E-04 \| 2.20E-04 \| \| ENSG00000254469 \|  \| -1.47 \| 1.10E-04 \| 2.20E-04 \| \| ENSG00000118690 \| ARMC2 \| -0.81 \| 1.10E-04 \| 2.22E-04 \| \| ENSG00000129535 \| NRL \| 1.09 \| 1.11E-04 \| 2.23E-04 \| \| ENSG00000276980 \|  \| 2.76 \| 1.13E-04 \| 2.26E-04 \| \| ENSG00000225101 \|  \| -1.86 \| 1.13E-04 \| 2.27E-04 \| \| ENSG00000082293 \| COL19A1 \| 1.91 \| 1.13E-04 \| 2.27E-04 \| \| ENSG00000164116 \| GUCY1A1 \| 1.24 \| 1.14E-04 \| 2.28E-04 \| \| ENSG00000223960 \| PRKRA-AS1 \| -0.69 \| 1.14E-04 \| 2.29E-04 \| \| ENSG00000280128 \|  \| 1.03 \| 1.15E-04 \| 2.31E-04 \| \| ENSG00000020426 \| MNAT1 \| -0.73 \| 1.17E-04 \| 2.35E-04 \| \| ENSG00000278535 \| DHRS11 \| -0.66 \| 1.17E-04 \| 2.35E-04 \| \| ENSG00000236591 \|  \| 0.79 \| 1.21E-04 \| 2.41E-04 \| \| ENSG00000170089 \| LOC728554 \| -1.02 \| 1.21E-04 \| 2.43E-04 \| \| ENSG00000226200 \| SGMS1-AS1 \| -1.30 \| 1.26E-04 \| 2.52E-04 \| \| ENSG00000188707 \| ZBED6CL \| -3.63 \| 1.26E-04 \| 2.52E-04 \| \| ENSG00000248905 \| FMN1 \| 0.96 \| 1.27E-04 \| 2.53E-04 \| \| ENSG00000126010 \| GRPR \| 1.40 \| 1.27E-04 \| 2.54E-04 \| \| ENSG00000068885 \| IFT80 \| -0.75 \| 1.29E-04 \| 2.57E-04 \| \| ENSG00000173698 \| ADGRG2 \| 3.02 \| 1.30E-04 \| 2.61E-04 \| \| ENSG00000273117 \|  \| 0.80 \| 1.32E-04 \| 2.64E-04 \| \| ENSG00000181031 \| RPH3AL \| -1.62 \| 1.36E-04 \| 2.72E-04 \| \| ENSG00000189212 \|  \| -1.69 \| 1.36E-04 \| 2.72E-04 \| \| ENSG00000198400 \| NTRK1 \| -1.73 \| 1.37E-04 \| 2.74E-04 \| \| ENSG00000170293 \| CMTM8 \| -2.24 \| 1.39E-04 \| 2.77E-04 \| \| ENSG00000281912 \| LINC01144 \| 1.17 \| 1.41E-04 \| 2.81E-04 \| \| ENSG00000133069 \| TMCC2 \| -2.36 \| 1.45E-04 \| 2.88E-04 \| \| ENSG00000160678 \| S100A1 \| -2.46 \| 1.45E-04 \| 2.88E-04 \| \| ENSG00000235961 \| PNMA6A \| -1.35 \| 1.46E-04 \| 2.90E-04 \| \| ENSG00000240024 \| LINC00888 \| -0.67 \| 1.46E-04 \| 2.90E-04 \| \| ENSG00000237513 \|  \| 0.59 \| 1.47E-04 \| 2.92E-04 \| \| ENSG00000271824 \| SMIM32 \| -2.97 \| 1.47E-04 \| 2.93E-04 \| \| ENSG00000150477 \| KIAA1328 \| -0.67 \| 1.49E-04 \| 2.95E-04 \| \| ENSG00000160188 \| RSPH1 \| 1.47 \| 1.49E-04 \| 2.96E-04 \| \| ENSG00000196917 \| HCAR1 \| -2.83 \| 1.51E-04 \| 2.99E-04 \| \| ENSG00000188599 \|  \| 0.66 \| 1.51E-04 \| 2.99E-04 \| \| ENSG00000133026 \| MYH10 \| -1.29 \| 1.51E-04 \| 2.99E-04 \| \| ENSG00000279355 \| AGPAT4-IT1 \| 0.85 \| 1.52E-04 \| 3.01E-04 \| \| ENSG00000269934 \|  \| -1.29 \| 1.52E-04 \| 3.01E-04 \| \| ENSG00000136371 \| MTHFS \| 1.63 \| 1.52E-04 \| 3.02E-04 \| \| ENSG00000197008 \| ZNF138 \| -0.81 \| 1.54E-04 \| 3.06E-04 \| \| ENSG00000155016 \| CYP2U1 \| 0.62 \| 1.55E-04 \| 3.06E-04 \| \| ENSG00000127528 \| KLF2 \| 0.75 \| 1.57E-04 \| 3.10E-04 \| \| ENSG00000275400 \|  \| 0.59 \| 1.60E-04 \| 3.17E-04 \| \| ENSG00000088179 \| PTPN4 \| -0.74 \| 1.62E-04 \| 3.20E-04 \| \| ENSG00000260877 \|  \| 1.75 \| 1.63E-04 \| 3.22E-04 \| \| ENSG00000217555 \| CKLF \| -1.19 \| 1.66E-04 \| 3.28E-04 \| \| ENSG00000158220 \| ESYT3 \| -2.31 \| 1.66E-04 \| 3.28E-04 \| \| ENSG00000050344 \| NFE2L3 \| -0.59 \| 1.69E-04 \| 3.33E-04 \| \| ENSG00000144485 \| HES6 \| -0.64 \| 1.70E-04 \| 3.35E-04 \| \| ENSG00000184925 \| LCN12 \| -1.55 \| 1.71E-04 \| 3.37E-04 \| \| ENSG00000152556 \| PFKM \| -0.86 \| 1.71E-04 \| 3.37E-04 \| \| ENSG00000279227 \|  \| 1.07 \| 1.71E-04 \| 3.38E-04 \| \| ENSG00000132274 \| TRIM22 \| -2.27 \| 1.74E-04 \| 3.43E-04 \| \| ENSG00000163888 \| CAMK2N2 \| 0.64 \| 1.75E-04 \| 3.44E-04 \| \| ENSG00000164125 \| FAM198B \| -1.68 \| 1.75E-04 \| 3.45E-04 \| \| ENSG00000146648 \| EGFR \| -1.44 \| 1.81E-04 \| 3.56E-04 \| \| ENSG00000148950 \| IMMP1L \| -1.11 \| 1.81E-04 \| 3.56E-04 \| \| ENSG00000134594 \| RAB33A \| -1.57 \| 1.81E-04 \| 3.57E-04 \| \| ENSG00000166173 \| LARP6 \| -1.05 \| 1.83E-04 \| 3.60E-04 \| \| ENSG00000197857 \| ZNF44 \| -0.98 \| 1.84E-04 \| 3.62E-04 \| \| ENSG00000273026 \|  \| 1.18 \| 1.84E-04 \| 3.62E-04 \| \| ENSG00000229191 \|  \| 1.08 \| 1.88E-04 \| 3.69E-04 \| \| ENSG00000139899 \| CBLN3 \| -0.61 \| 1.92E-04 \| 3.77E-04 \| \| ENSG00000204860 \| FAM201A \| -1.17 \| 1.94E-04 \| 3.81E-04 \| \| ENSG00000227917 \|  \| -2.65 \| 1.95E-04 \| 3.82E-04 \| \| ENSG00000269553 \|  \| -2.91 \| 1.98E-04 \| 3.89E-04 \| \| ENSG00000138759 \| FRAS1 \| -1.74 \| 1.99E-04 \| 3.90E-04 \| \| ENSG00000139971 \| ARMH4 \| 2.36 \| 2.03E-04 \| 3.97E-04 \| \| ENSG00000148814 \| LRRC27 \| -0.73 \| 2.03E-04 \| 3.97E-04 \| \| ENSG00000255864 \|  \| 1.86 \| 2.04E-04 \| 3.99E-04 \| \| ENSG00000103154 \| NECAB2 \| -1.97 \| 2.05E-04 \| 4.00E-04 \| \| ENSG00000280347 \|  \| 0.76 \| 2.06E-04 \| 4.03E-04 \| \| ENSG00000081803 \| CADPS2 \| -3.12 \| 2.06E-04 \| 4.03E-04 \| \| ENSG00000100926 \| TM9SF1 \| 0.75 \| 2.07E-04 \| 4.05E-04 \| \| ENSG00000166845 \| C18orf54 \| -0.86 \| 2.09E-04 \| 4.08E-04 \| \| ENSG00000180596 \| HIST1H2BC \| 1.09 \| 2.18E-04 \| 4.26E-04 \| \| ENSG00000087085 \| ACHE \| 1.28 \| 2.19E-04 \| 4.27E-04 \| \| ENSG00000147570 \| DNAJC5B \| -2.19 \| 2.19E-04 \| 4.28E-04 \| \| ENSG00000221995 \| TIAF1 \| -1.83 \| 2.19E-04 \| 4.29E-04 \| \| ENSG00000197134 \| ZNF257 \| -1.18 \| 2.20E-04 \| 4.30E-04 \| \| ENSG00000231645 \|  \| -4.10 \| 2.24E-04 \| 4.36E-04 \| \| ENSG00000232325 \|  \| 0.99 \| 2.24E-04 \| 4.37E-04 \| \| ENSG00000265190 \| ANXA8 \| -3.47 \| 2.26E-04 \| 4.41E-04 \| \| ENSG00000205181 \| LINC00654 \| -4.74 \| 2.26E-04 \| 4.41E-04 \| \| ENSG00000105374 \| NKG7 \| 0.80 \| 2.28E-04 \| 4.43E-04 \| \| ENSG00000273706 \| LHX1 \| -1.46 \| 2.29E-04 \| 4.45E-04 \| \| ENSG00000167083 \| GNGT2 \| -3.52 \| 2.33E-04 \| 4.53E-04 \| \| ENSG00000197275 \| RAD54B \| -1.69 \| 2.36E-04 \| 4.59E-04 \| \| ENSG00000107611 \| CUBN \| 1.25 \| 2.38E-04 \| 4.62E-04 \| \| ENSG00000128016 \| ZFP36 \| -0.74 \| 2.38E-04 \| 4.63E-04 \| \| ENSG00000147912 \| FBXO10 \| -0.62 \| 2.39E-04 \| 4.64E-04 \| \| ENSG00000250492 \|  \| 2.15 \| 2.39E-04 \| 4.65E-04 \| \| ENSG00000105717 \| PBX4 \| 0.69 \| 2.43E-04 \| 4.72E-04 \| \| ENSG00000184261 \| KCNK12 \| -2.39 \| 2.45E-04 \| 4.75E-04 \| \| ENSG00000132692 \| BCAN \| -4.10 \| 2.45E-04 \| 4.77E-04 \| \| ENSG00000065675 \| PRKCQ \| -2.57 \| 2.46E-04 \| 4.78E-04 \| \| ENSG00000279879 \|  \| -1.98 \| 2.56E-04 \| 4.96E-04 \| \| ENSG00000123453 \| SARDH \| -2.89 \| 2.56E-04 \| 4.96E-04 \| \| ENSG00000124233 \| SEMG1 \| -4.70 \| 2.58E-04 \| 5.00E-04 \| \| ENSG00000171051 \| FPR1 \| -1.65 \| 2.60E-04 \| 5.03E-04 \| \| ENSG00000156050 \| FAM161B \| 0.82 \| 2.63E-04 \| 5.08E-04 \| \| ENSG00000250234 \|  \| 2.09 \| 2.66E-04 \| 5.14E-04 \| \| ENSG00000237732 \| LOC440934 \| 1.20 \| 2.66E-04 \| 5.15E-04 \| \| ENSG00000047634 \| SCML1 \| 0.73 \| 2.68E-04 \| 5.19E-04 \| \| ENSG00000182648 \| LINC01006 \| -0.78 \| 2.69E-04 \| 5.19E-04 \| \| ENSG00000135312 \| HTR1B \| -0.90 \| 2.71E-04 \| 5.25E-04 \| \| ENSG00000237484 \| LINC01684 \| -1.21 \| 2.74E-04 \| 5.29E-04 \| \| ENSG00000176928 \| GCNT4 \| 1.65 \| 2.78E-04 \| 5.37E-04 \| \| ENSG00000172244 \| C5orf34 \| -0.94 \| 2.86E-04 \| 5.53E-04 \| \| ENSG00000144645 \| OSBPL10 \| -1.32 \| 2.87E-04 \| 5.54E-04 \| \| ENSG00000205628 \| LINC01446 \| -4.65 \| 2.90E-04 \| 5.60E-04 \| \| ENSG00000245571 \| FAM111A-DT \| -0.89 \| 2.96E-04 \| 5.70E-04 \| \| ENSG00000172322 \| CLEC12A \| -2.33 \| 2.96E-04 \| 5.70E-04 \| \| ENSG00000175147 \| TMEM51-AS1 \| -0.93 \| 2.97E-04 \| 5.73E-04 \| \| ENSG00000282393 \|  \| 1.56 \| 2.99E-04 \| 5.75E-04 \| \| ENSG00000134285 \| FKBP11 \| -0.89 \| 2.99E-04 \| 5.75E-04 \| \| ENSG00000255135 \|  \| -1.32 \| 3.01E-04 \| 5.80E-04 \| \| ENSG00000167642 \| SPINT2 \| -3.52 \| 3.05E-04 \| 5.87E-04 \| \| ENSG00000153904 \| DDAH1 \| -0.81 \| 3.06E-04 \| 5.88E-04 \| \| ENSG00000158987 \| RAPGEF6 \| -0.74 \| 3.07E-04 \| 5.91E-04 \| \| ENSG00000262468 \| LINC01569 \| -1.06 \| 3.07E-04 \| 5.91E-04 \| \| ENSG00000125249 \| RAP2A \| -0.77 \| 3.08E-04 \| 5.92E-04 \| \| ENSG00000259071 \|  \| -1.90 \| 3.09E-04 \| 5.93E-04 \| \| ENSG00000239445 \| ST3GAL6-AS1 \| -0.96 \| 3.12E-04 \| 6.00E-04 \| \| ENSG00000255666 \|  \| -1.02 \| 3.15E-04 \| 6.06E-04 \| \| ENSG00000260285 \|  \| 1.04 \| 3.18E-04 \| 6.11E-04 \| \| ENSG00000167535 \| CACNB3 \| -0.67 \| 3.28E-04 \| 6.30E-04 \| \| ENSG00000173275 \| ZNF449 \| -0.67 \| 3.31E-04 \| 6.35E-04 \| \| ENSG00000113645 \| WWC1 \| -0.76 \| 3.32E-04 \| 6.36E-04 \| \| ENSG00000230701 \| FBXW4P1 \| 1.22 \| 3.32E-04 \| 6.37E-04 \| \| ENSG00000274370 \|  \| 1.02 \| 3.33E-04 \| 6.37E-04 \| \| ENSG00000261884 \|  \| -1.10 \| 3.33E-04 \| 6.38E-04 \| \| ENSG00000162825 \| NBPF20 \| 0.93 \| 3.36E-04 \| 6.42E-04 \| \| ENSG00000196208 \| GREB1 \| -1.25 \| 3.38E-04 \| 6.46E-04 \| \| ENSG00000095585 \| BLNK \| -3.01 \| 3.43E-04 \| 6.56E-04 \| \| ENSG00000237499 \| LOC100130476 \| -0.97 \| 3.45E-04 \| 6.59E-04 \| \| ENSG00000092051 \| JPH4 \| -3.13 \| 3.46E-04 \| 6.61E-04 \| \| ENSG00000280852 \| LOC653653 \| 0.96 \| 3.51E-04 \| 6.70E-04 \| \| ENSG00000087299 \| L2HGDH \| -0.79 \| 3.54E-04 \| 6.76E-04 \| \| ENSG00000169750 \| RAC3 \| -0.80 \| 3.61E-04 \| 6.89E-04 \| \| ENSG00000100206 \| DMC1 \| -3.36 \| 3.63E-04 \| 6.92E-04 \| \| ENSG00000150667 \| FSIP1 \| 2.24 \| 3.65E-04 \| 6.95E-04 \| \| ENSG00000122507 \| BBS9 \| 0.86 \| 3.65E-04 \| 6.95E-04 \| \| ENSG00000280239 \|  \| -1.88 \| 3.65E-04 \| 6.96E-04 \| \| ENSG00000172572 \| PDE3A \| 1.14 \| 3.66E-04 \| 6.97E-04 \| \| ENSG00000169193 \| CCDC126 \| 0.59 \| 3.73E-04 \| 7.11E-04 \| \| ENSG00000125170 \| DOK4 \| -1.05 \| 3.74E-04 \| 7.12E-04 \| \| ENSG00000176273 \| SLC35G1 \| -1.04 \| 3.75E-04 \| 7.14E-04 \| \| ENSG00000241634 \|  \| -2.46 \| 3.78E-04 \| 7.20E-04 \| \| ENSG00000273084 \|  \| -2.00 \| 3.80E-04 \| 7.23E-04 \| \| ENSG00000260910 \| LINC00565 \| 1.21 \| 3.80E-04 \| 7.23E-04 \| \| ENSG00000172037 \| LAMB2 \| -3.97 \| 3.80E-04 \| 7.23E-04 \| \| ENSG00000271270 \| TMCC1-AS1 \| -0.80 \| 3.82E-04 \| 7.26E-04 \| \| ENSG00000236008 \| LINC01814 \| -1.51 \| 3.84E-04 \| 7.30E-04 \| \| ENSG00000277496 \|  \| -1.27 \| 3.87E-04 \| 7.35E-04 \| \| ENSG00000132570 \| PCBD2 \| -0.60 \| 3.87E-04 \| 7.36E-04 \| \| ENSG00000124523 \| SIRT5 \| -0.73 \| 3.92E-04 \| 7.44E-04 \| \| ENSG00000162174 \| ASRGL1 \| -1.34 \| 3.93E-04 \| 7.46E-04 \| \| ENSG00000181350 \| LRRC75A \| -1.12 \| 3.98E-04 \| 7.56E-04 \| \| ENSG00000170775 \| GPR37 \| 1.95 \| 3.99E-04 \| 7.57E-04 \| \| ENSG00000232973 \| CYP1B1-AS1 \| 1.00 \| 4.04E-04 \| 7.67E-04 \| \| ENSG00000230373 \| GOLGA6L5P \| -1.99 \| 4.09E-04 \| 7.75E-04 \| \| ENSG00000113205 \| PCDHB3 \| -2.00 \| 4.09E-04 \| 7.76E-04 \| \| ENSG00000125245 \| GPR18 \| -0.88 \| 4.14E-04 \| 7.84E-04 \| \| ENSG00000279407 \|  \| -1.50 \| 4.14E-04 \| 7.84E-04 \| \| ENSG00000254102 \| LOC401463 \| -0.89 \| 4.17E-04 \| 7.90E-04 \| \| ENSG00000133302 \| SLF1 \| -0.85 \| 4.19E-04 \| 7.93E-04 \| \| ENSG00000276141 \| WHAMMP3 \| -2.11 \| 4.27E-04 \| 8.09E-04 \| \| ENSG00000270055 \|  \| -1.53 \| 4.32E-04 \| 8.18E-04 \| \| ENSG00000123352 \| SPATS2 \| -0.75 \| 4.34E-04 \| 8.22E-04 \| \| ENSG00000101213 \| PTK6 \| -0.91 \| 4.35E-04 \| 8.22E-04 \| \| ENSG00000185885 \| IFITM1 \| -4.62 \| 4.36E-04 \| 8.24E-04 \| \| ENSG00000125816 \| NKX2-4 \| -0.68 \| 4.36E-04 \| 8.25E-04 \| \| ENSG00000258976 \|  \| 1.28 \| 4.40E-04 \| 8.31E-04 \| \| ENSG00000105371 \| ICAM4 \| 1.13 \| 4.42E-04 \| 8.35E-04 \| \| ENSG00000149260 \| CAPN5 \| -0.86 \| 4.46E-04 \| 8.41E-04 \| \| ENSG00000167723 \| TRPV3 \| 1.63 \| 4.46E-04 \| 8.42E-04 \| \| ENSG00000137274 \| BPHL \| -1.00 \| 4.47E-04 \| 8.43E-04 \| \| ENSG00000179029 \| TMEM107 \| -0.67 \| 4.48E-04 \| 8.46E-04 \| \| ENSG00000110848 \| CD69 \| -4.68 \| 4.52E-04 \| 8.53E-04 \| \| ENSG00000253981 \|  \| -0.84 \| 4.53E-04 \| 8.54E-04 \| \| ENSG00000227640 \| SOX21-AS1 \| 2.00 \| 4.55E-04 \| 8.59E-04 \| \| ENSG00000154330 \| PGM5 \| -3.97 \| 4.57E-04 \| 8.62E-04 \| \| ENSG00000255366 \|  \| -1.72 \| 4.62E-04 \| 8.72E-04 \| \| ENSG00000166104 \|  \| -0.97 \| 4.70E-04 \| 8.85E-04 \| \| ENSG00000198019 \| FCGR1B \| -0.92 \| 4.71E-04 \| 8.87E-04 \| \| ENSG00000146859 \| TMEM140 \| -1.46 \| 4.73E-04 \| 8.91E-04 \| \| ENSG00000177468 \| OLIG3 \| -3.99 \| 4.75E-04 \| 8.94E-04 \| \| ENSG00000166793 \| YPEL4 \| 1.58 \| 4.77E-04 \| 8.98E-04 \| \| ENSG00000135824 \| RGS8 \| -1.96 \| 4.78E-04 \| 8.99E-04 \| \| ENSG00000092421 \| SEMA6A \| -1.06 \| 4.78E-04 \| 9.01E-04 \| \| ENSG00000079393 \| DUSP13 \| -3.00 \| 4.85E-04 \| 9.13E-04 \| \| ENSG00000272720 \|  \| -0.98 \| 4.88E-04 \| 9.17E-04 \| \| ENSG00000136999 \| NOV \| -1.44 \| 4.89E-04 \| 9.19E-04 \| \| ENSG00000272908 \|  \| -0.77 \| 4.99E-04 \| 9.37E-04 \| \| ENSG00000181652 \| ATG9B \| -2.10 \| 4.99E-04 \| 9.38E-04 \| \| ENSG00000144712 \| CAND2 \| -4.55 \| 5.02E-04 \| 9.42E-04 \| \| ENSG00000279118 \|  \| 1.39 \| 5.02E-04 \| 9.43E-04 \| \| ENSG00000246582 \| LOC389641 \| -1.20 \| 5.13E-04 \| 9.62E-04 \| \| ENSG00000183850 \| ZNF730 \| -2.28 \| 5.14E-04 \| 9.64E-04 \| \| ENSG00000123358 \| NR4A1 \| -1.92 \| 5.14E-04 \| 9.65E-04 \| \| ENSG00000100583 \| SAMD15 \| 1.01 \| 5.15E-04 \| 9.66E-04 \| \| ENSG00000267221 \| ZNF385C \| -1.71 \| 5.24E-04 \| 9.82E-04 \| \| ENSG00000267221 \| C17orf113 \| -1.71 \| 5.24E-04 \| 9.82E-04 \| \| ENSG00000228672 \| PROB1 \| 0.74 \| 5.29E-04 \| 9.90E-04 \| \| ENSG00000124140 \| SLC12A5 \| -0.81 \| 5.40E-04 \| 1.01E-03 \| \| ENSG00000169184 \| MN1 \| 1.71 \| 5.40E-04 \| 1.01E-03 \| \| ENSG00000123685 \| BATF3 \| -1.94 \| 5.43E-04 \| 1.02E-03 \| \| ENSG00000179965 \| ZNF771 \| 0.63 \| 5.43E-04 \| 1.02E-03 \| \| ENSG00000185924 \| RTN4RL1 \| -2.96 \| 5.54E-04 \| 1.04E-03 \| \| ENSG00000254389 \| RHPN1-AS1 \| -0.98 \| 5.54E-04 \| 1.04E-03 \| \| ENSG00000145506 \| NKD2 \| -1.58 \| 5.58E-04 \| 1.04E-03 \| \| ENSG00000198948 \| MFAP3L \| 2.40 \| 5.59E-04 \| 1.05E-03 \| \| ENSG00000188511 \| C22orf34 \| -1.41 \| 5.74E-04 \| 1.07E-03 \| \| ENSG00000182963 \| GJC1 \| -0.72 \| 5.75E-04 \| 1.07E-03 \| \| ENSG00000236449 \|  \| 0.82 \| 5.81E-04 \| 1.09E-03 \| \| ENSG00000081059 \| TCF7 \| 0.70 \| 5.82E-04 \| 1.09E-03 \| \| ENSG00000163053 \| SLC16A14 \| -2.32 \| 5.83E-04 \| 1.09E-03 \| \| ENSG00000236830 \| CBR3-AS1 \| -0.85 \| 5.84E-04 \| 1.09E-03 \| \| ENSG00000118849 \| RARRES1 \| -2.76 \| 5.88E-04 \| 1.10E-03 \| \| ENSG00000198753 \| PLXNB3 \| -0.63 \| 5.94E-04 \| 1.11E-03 \| \| ENSG00000180720 \| CHRM4 \| 1.73 \| 5.95E-04 \| 1.11E-03 \| \| ENSG00000253520 \|  \| -3.92 \| 5.98E-04 \| 1.11E-03 \| \| ENSG00000276672 \|  \| -1.32 \| 5.98E-04 \| 1.12E-03 \| \| ENSG00000214837 \| LINC01347 \| 1.06 \| 6.09E-04 \| 1.13E-03 \| \| ENSG00000225062 \| CATIP-AS1 \| 1.36 \| 6.09E-04 \| 1.14E-03 \| \| ENSG00000197465 \| GYPE \| 2.16 \| 6.10E-04 \| 1.14E-03 \| \| ENSG00000204186 \| ZDBF2 \| 1.44 \| 6.13E-04 \| 1.14E-03 \| \| ENSG00000132561 \| MATN2 \| -0.68 \| 6.14E-04 \| 1.14E-03 \| \| ENSG00000177337 \| DLGAP1-AS1 \| -0.72 \| 6.16E-04 \| 1.15E-03 \| \| ENSG00000239569 \|  \| 1.17 \| 6.17E-04 \| 1.15E-03 \| \| ENSG00000261423 \| TMEM202-AS1 \| 0.83 \| 6.24E-04 \| 1.16E-03 \| \| ENSG00000227502 \| LINC01268 \| -2.22 \| 6.25E-04 \| 1.16E-03 \| \| ENSG00000222011 \| FAM185A \| -0.99 \| 6.27E-04 \| 1.17E-03 \| \| ENSG00000182950 \| ODF3L1 \| -1.01 \| 6.36E-04 \| 1.18E-03 \| \| ENSG00000255045 \|  \| -1.22 \| 6.43E-04 \| 1.19E-03 \| \| ENSG00000171757 \| LRRC34 \| -0.88 \| 6.53E-04 \| 1.21E-03 \| \| ENSG00000052126 \| PLEKHA5 \| -0.60 \| 6.59E-04 \| 1.22E-03 \| \| ENSG00000124019 \| FAM124B \| -1.85 \| 6.60E-04 \| 1.23E-03 \| \| ENSG00000227766 \|  \| -0.81 \| 6.63E-04 \| 1.23E-03 \| \| ENSG00000131480 \| AOC2 \| 1.93 \| 6.68E-04 \| 1.24E-03 \| \| ENSG00000236088 \| COX10-AS1 \| 0.62 \| 6.68E-04 \| 1.24E-03 \| \| ENSG00000160200 \| CBS \| -0.67 \| 6.70E-04 \| 1.24E-03 \| \| ENSG00000160200 \| CBSL \| -0.67 \| 6.70E-04 \| 1.24E-03 \| \| ENSG00000130810 \| PPAN \| -0.65 \| 6.71E-04 \| 1.24E-03 \| \| ENSG00000063015 \| SEZ6 \| -1.19 \| 6.81E-04 \| 1.26E-03 \| \| ENSG00000198515 \| CNGA1 \| 1.96 \| 6.86E-04 \| 1.27E-03 \| \| ENSG00000223768 \| LOC642852 \| -0.73 \| 6.86E-04 \| 1.27E-03 \| \| ENSG00000248208 \|  \| 0.97 \| 6.89E-04 \| 1.27E-03 \| \| ENSG00000250635 \| CXXC5-AS1 \| 0.85 \| 6.91E-04 \| 1.28E-03 \| \| ENSG00000274565 \|  \| -0.75 \| 7.09E-04 \| 1.31E-03 \| \| ENSG00000237836 \| PHKA2-AS1 \| -2.90 \| 7.16E-04 \| 1.32E-03 \| \| ENSG00000246095 \| LINC01096 \| -1.72 \| 7.21E-04 \| 1.33E-03 \| \| ENSG00000138794 \| CASP6 \| -0.65 \| 7.25E-04 \| 1.34E-03 \| \| ENSG00000272914 \|  \| -2.31 \| 7.26E-04 \| 1.34E-03 \| \| ENSG00000088538 \| DOCK3 \| -0.96 \| 7.45E-04 \| 1.37E-03 \| \| ENSG00000254614 \| LOC728975 \| -0.69 \| 7.45E-04 \| 1.37E-03 \| \| ENSG00000002933 \| TMEM176A \| -2.67 \| 7.47E-04 \| 1.38E-03 \| \| ENSG00000155974 \| GRIP1 \| -0.94 \| 7.47E-04 \| 1.38E-03 \| \| ENSG00000188013 \|  \| -2.12 \| 7.60E-04 \| 1.40E-03 \| \| ENSG00000158023 \| WDR66 \| -0.82 \| 7.68E-04 \| 1.41E-03 \| \| ENSG00000124249 \| KCNK15 \| 2.24 \| 7.81E-04 \| 1.43E-03 \| \| ENSG00000275993 \| SIK1B \| 0.76 \| 7.91E-04 \| 1.45E-03 \| \| ENSG00000275055 \|  \| 1.99 \| 7.93E-04 \| 1.46E-03 \| \| ENSG00000274712 \|  \| 0.97 \| 7.98E-04 \| 1.47E-03 \| \| ENSG00000174502 \| SLC26A9 \| 2.05 \| 8.00E-04 \| 1.47E-03 \| \| ENSG00000204116 \| CHIC1 \| -0.61 \| 8.03E-04 \| 1.47E-03 \| \| ENSG00000196391 \| ZNF774 \| -1.34 \| 8.11E-04 \| 1.49E-03 \| \| ENSG00000251867 \|  \| -1.12 \| 8.13E-04 \| 1.49E-03 \| \| ENSG00000160606 \| TLCD1 \| 1.09 \| 8.19E-04 \| 1.50E-03 \| \| ENSG00000113083 \| LOX \| 0.94 \| 8.21E-04 \| 1.50E-03 \| \| ENSG00000253522 \| MIR3142HG \| 0.82 \| 8.32E-04 \| 1.52E-03 \| \| ENSG00000142794 \| NBPF3 \| -0.64 \| 8.38E-04 \| 1.53E-03 \| \| ENSG00000196972 \| SMIM10L2B \| 1.38 \| 8.57E-04 \| 1.57E-03 \| \| ENSG00000259030 \| FPGT-TNNI3K \| -0.90 \| 8.57E-04 \| 1.57E-03 \| \| ENSG00000233895 \|  \| -2.31 \| 8.71E-04 \| 1.59E-03 \| \| ENSG00000237187 \| NR2F1-AS1 \| 0.72 \| 8.78E-04 \| 1.60E-03 \| \| ENSG00000119411 \| BSPRY \| -1.38 \| 9.03E-04 \| 1.65E-03 \| \| ENSG00000258839 \| MC1R \| 0.80 \| 9.06E-04 \| 1.65E-03 \| \| ENSG00000142959 \| BEST4 \| 1.68 \| 9.11E-04 \| 1.66E-03 \| \| ENSG00000147465 \| STAR \| -1.54 \| 9.14E-04 \| 1.67E-03 \| \| ENSG00000267601 \|  \| -2.17 \| 9.17E-04 \| 1.67E-03 \| \| ENSG00000250995 \|  \| 0.91 \| 9.19E-04 \| 1.67E-03 \| \| ENSG00000279821 \|  \| -1.19 \| 9.22E-04 \| 1.68E-03 \| \| ENSG00000161609 \| CCDC155 \| -1.30 \| 9.33E-04 \| 1.70E-03 \| \| ENSG00000108511 \| HOXB6 \| -1.92 \| 9.40E-04 \| 1.71E-03 \| \| ENSG00000070087 \| PFN2 \| 1.38 \| 9.43E-04 \| 1.72E-03 \| \| ENSG00000150773 \| PIH1D2 \| 1.28 \| 9.43E-04 \| 1.72E-03 \| \| ENSG00000260401 \|  \| -0.60 \| 9.47E-04 \| 1.72E-03 \| \| ENSG00000281183 \| NPTN-IT1 \| 0.61 \| 9.71E-04 \| 1.77E-03 \| \| ENSG00000197472 \| ZNF695 \| -1.10 \| 9.73E-04 \| 1.77E-03 \| \| ENSG00000261359 \| PYCARD-AS1 \| -1.17 \| 9.82E-04 \| 1.78E-03 \| \| ENSG00000128536 \| CDHR3 \| -4.32 \| 9.91E-04 \| 1.80E-03 \| \| ENSG00000187123 \| LYPD6 \| -1.87 \| 1.00E-03 \| 1.82E-03 \| \| ENSG00000224914 \| LINC00863 \| -0.73 \| 1.01E-03 \| 1.83E-03 \| \| ENSG00000241990 \|  \| -2.67 \| 1.01E-03 \| 1.84E-03 \| \| ENSG00000124391 \| IL17C \| 1.75 \| 1.02E-03 \| 1.85E-03 \| \| ENSG00000272768 \|  \| 0.76 \| 1.02E-03 \| 1.86E-03 \| \| ENSG00000186496 \| ZNF396 \| 1.04 \| 1.03E-03 \| 1.86E-03 \| \| ENSG00000277342 \|  \| 1.53 \| 1.03E-03 \| 1.87E-03 \| \| ENSG00000249042 \| LOC105379049 \| -1.77 \| 1.03E-03 \| 1.87E-03 \| \| ENSG00000280832 \| GSEC \| -0.93 \| 1.04E-03 \| 1.87E-03 \| \| ENSG00000172403 \| SYNPO2 \| -1.49 \| 1.04E-03 \| 1.88E-03 \| \| ENSG00000182749 \| PAQR7 \| -1.32 \| 1.06E-03 \| 1.91E-03 \| \| ENSG00000166831 \| RBPMS2 \| -1.12 \| 1.06E-03 \| 1.92E-03 \| \| ENSG00000106484 \| MEST \| -1.36 \| 1.08E-03 \| 1.95E-03 \| \| ENSG00000279519 \|  \| 1.05 \| 1.08E-03 \| 1.96E-03 \| \| ENSG00000172260 \| NEGR1 \| 2.16 \| 1.09E-03 \| 1.98E-03 \| \| ENSG00000277462 \| ZNF670 \| -0.72 \| 1.10E-03 \| 1.98E-03 \| \| ENSG00000215817 \| ZC3H11B \| 1.15 \| 1.10E-03 \| 1.98E-03 \| \| ENSG00000166997 \| CNPY4 \| -0.63 \| 1.10E-03 \| 1.99E-03 \| \| ENSG00000198597 \| ZNF536 \| -0.86 \| 1.11E-03 \| 2.01E-03 \| \| ENSG00000257108 \| NHLRC4 \| -1.52 \| 1.12E-03 \| 2.03E-03 \| \| ENSG00000172007 \| RAB33B \| 0.59 \| 1.13E-03 \| 2.05E-03 \| \| ENSG00000162849 \| KIF26B \| -1.05 \| 1.13E-03 \| 2.05E-03 \| \| ENSG00000167711 \| SERPINF2 \| -2.00 \| 1.14E-03 \| 2.05E-03 \| \| ENSG00000143850 \| PLEKHA6 \| -2.85 \| 1.14E-03 \| 2.06E-03 \| \| ENSG00000185339 \| TCN2 \| -2.62 \| 1.15E-03 \| 2.08E-03 \| \| ENSG00000080031 \| PTPRH \| 0.68 \| 1.16E-03 \| 2.09E-03 \| \| ENSG00000236914 \| LINC01852 \| -3.73 \| 1.16E-03 \| 2.10E-03 \| \| ENSG00000177106 \| EPS8L2 \| -2.29 \| 1.17E-03 \| 2.10E-03 \| \| ENSG00000138336 \| TET1 \| -3.16 \| 1.17E-03 \| 2.11E-03 \| \| ENSG00000112812 \| PRSS16 \| -1.99 \| 1.19E-03 \| 2.14E-03 \| \| ENSG00000163017 \| ACTG2 \| -3.18 \| 1.20E-03 \| 2.15E-03 \| \| ENSG00000214455 \|  \| 0.84 \| 1.20E-03 \| 2.16E-03 \| \| ENSG00000159674 \| SPON2 \| -0.94 \| 1.21E-03 \| 2.17E-03 \| \| ENSG00000159674 \| LOC100130872 \| -0.94 \| 1.21E-03 \| 2.17E-03 \| \| ENSG00000268157 \|  \| -1.64 \| 1.23E-03 \| 2.22E-03 \| \| ENSG00000153982 \| GDPD1 \| -0.73 \| 1.24E-03 \| 2.24E-03 \| \| ENSG00000177238 \| TRIM72 \| -2.57 \| 1.24E-03 \| 2.24E-03 \| \| ENSG00000270504 \|  \| -1.10 \| 1.25E-03 \| 2.24E-03 \| \| ENSG00000180881 \| CAPS2 \| -1.39 \| 1.25E-03 \| 2.25E-03 \| \| ENSG00000256340 \| ABCC6P1 \| -3.74 \| 1.25E-03 \| 2.26E-03 \| \| ENSG00000196696 \| PDXDC2P-NPIPB14P \| 0.90 \| 1.25E-03 \| 2.26E-03 \| \| ENSG00000231312 \| MAP4K3-DT \| 0.85 \| 1.26E-03 \| 2.27E-03 \| \| ENSG00000126500 \| FLRT1 \| -1.76 \| 1.27E-03 \| 2.29E-03 \| \| ENSG00000268061 \| NAPA-AS1 \| -1.44 \| 1.28E-03 \| 2.30E-03 \| \| ENSG00000259291 \| ZNF710-AS1 \| -0.68 \| 1.29E-03 \| 2.32E-03 \| \| ENSG00000267469 \|  \| -1.49 \| 1.30E-03 \| 2.33E-03 \| \| ENSG00000130347 \| RTN4IP1 \| -0.77 \| 1.30E-03 \| 2.33E-03 \| \| ENSG00000234636 \| MED14OS \| 1.66 \| 1.30E-03 \| 2.33E-03 \| \| ENSG00000271976 \|  \| 1.00 \| 1.30E-03 \| 2.33E-03 \| \| ENSG00000123870 \|  \| -2.30 \| 1.30E-03 \| 2.33E-03 \| \| ENSG00000213199 \| ASIC3 \| -0.80 \| 1.30E-03 \| 2.33E-03 \| \| ENSG00000234719 \| NPIPB2 \| 0.73 \| 1.32E-03 \| 2.36E-03 \| \| ENSG00000187068 \| C3orf70 \| 0.70 \| 1.32E-03 \| 2.37E-03 \| \| ENSG00000162543 \| UBXN10 \| -1.49 \| 1.32E-03 \| 2.37E-03 \| \| ENSG00000263432 \|  \| -3.66 \| 1.33E-03 \| 2.37E-03 \| \| ENSG00000154027 \| AK5 \| -1.74 \| 1.33E-03 \| 2.38E-03 \| \| ENSG00000205571 \| SMN1 \| 0.69 \| 1.34E-03 \| 2.40E-03 \| \| ENSG00000205571 \| SMN2 \| 0.69 \| 1.34E-03 \| 2.40E-03 \| \| ENSG00000124749 \| COL21A1 \| -3.66 \| 1.35E-03 \| 2.42E-03 \| \| ENSG00000174721 \| FGFBP3 \| -1.18 \| 1.36E-03 \| 2.42E-03 \| \| ENSG00000026103 \| FAS \| 0.74 \| 1.36E-03 \| 2.43E-03 \| \| ENSG00000275713 \| HIST1H2BH \| -1.96 \| 1.37E-03 \| 2.45E-03 \| \| ENSG00000142512 \| SIGLEC10 \| -1.33 \| 1.38E-03 \| 2.46E-03 \| \| ENSG00000079691 \| CARMIL1 \| -0.80 \| 1.39E-03 \| 2.48E-03 \| \| ENSG00000253141 \|  \| -0.94 \| 1.39E-03 \| 2.48E-03 \| \| ENSG00000279138 \|  \| 1.46 \| 1.44E-03 \| 2.56E-03 \| \| ENSG00000270175 \|  \| 1.21 \| 1.45E-03 \| 2.59E-03 \| \| ENSG00000115271 \| GCA \| -0.68 \| 1.47E-03 \| 2.62E-03 \| \| ENSG00000179913 \| B3GNT3 \| -2.08 \| 1.50E-03 \| 2.67E-03 \| \| ENSG00000233058 \|  \| 0.87 \| 1.51E-03 \| 2.68E-03 \| \| ENSG00000135480 \| KRT7 \| 1.07 \| 1.51E-03 \| 2.68E-03 \| \| ENSG00000187808 \| SOWAHD \| -0.79 \| 1.53E-03 \| 2.73E-03 \| \| ENSG00000124613 \| ZNF391 \| -1.69 \| 1.54E-03 \| 2.74E-03 \| \| ENSG00000264242 \|  \| -1.95 \| 1.56E-03 \| 2.77E-03 \| \| ENSG00000171132 \| PRKCE \| -1.02 \| 1.56E-03 \| 2.77E-03 \| \| ENSG00000198576 \| ARC \| -0.81 \| 1.57E-03 \| 2.79E-03 \| \| ENSG00000227507 \| LTB \| 0.69 \| 1.58E-03 \| 2.81E-03 \| \| ENSG00000180035 \| ZNF48 \| -1.13 \| 1.59E-03 \| 2.81E-03 \| \| ENSG00000129654 \| FOXJ1 \| -1.77 \| 1.59E-03 \| 2.82E-03 \| \| ENSG00000196436 \| NPIPB15 \| 0.83 \| 1.60E-03 \| 2.83E-03 \| \| ENSG00000153902 \| LGI4 \| 1.48 \| 1.62E-03 \| 2.87E-03 \| \| ENSG00000183496 \| MEX3B \| 0.93 \| 1.63E-03 \| 2.88E-03 \| \| ENSG00000257698 \| GIHCG \| -1.10 \| 1.63E-03 \| 2.89E-03 \| \| ENSG00000102349 \| KLF8 \| -0.68 \| 1.63E-03 \| 2.89E-03 \| \| ENSG00000261840 \| LOC730183 \| 1.07 \| 1.63E-03 \| 2.89E-03 \| \| ENSG00000176894 \| PXMP2 \| -1.13 \| 1.64E-03 \| 2.91E-03 \| \| ENSG00000268581 \|  \| 1.69 \| 1.65E-03 \| 2.91E-03 \| \| ENSG00000254027 \|  \| -0.81 \| 1.65E-03 \| 2.92E-03 \| \| ENSG00000128564 \| VGF \| -1.02 \| 1.65E-03 \| 2.92E-03 \| \| ENSG00000204177 \| BMS1P1 \| -1.48 \| 1.66E-03 \| 2.94E-03 \| \| ENSG00000219665 \| ZNF433-AS1 \| -0.78 \| 1.67E-03 \| 2.96E-03 \| \| ENSG00000205978 \| NYNRIN \| -3.58 \| 1.68E-03 \| 2.97E-03 \| \| ENSG00000134202 \| GSTM3 \| -0.66 \| 1.68E-03 \| 2.98E-03 \| \| ENSG00000164683 \| HEY1 \| 1.26 \| 1.70E-03 \| 3.00E-03 \| \| ENSG00000272839 \|  \| -2.27 \| 1.70E-03 \| 3.00E-03 \| \| ENSG00000213906 \| LTB4R2 \| -0.92 \| 1.70E-03 \| 3.01E-03 \| \| ENSG00000161944 \| ASGR2 \| -0.77 \| 1.72E-03 \| 3.04E-03 \| \| ENSG00000267414 \| LOC101927943 \| 0.89 \| 1.73E-03 \| 3.05E-03 \| \| ENSG00000117400 \| MPL \| -3.70 \| 1.74E-03 \| 3.07E-03 \| \| ENSG00000089723 \| OTUB2 \| -0.68 \| 1.78E-03 \| 3.14E-03 \| \| ENSG00000173041 \| ZNF680 \| -0.66 \| 1.78E-03 \| 3.14E-03 \| \| ENSG00000166046 \| TCP11L2 \| 0.64 \| 1.78E-03 \| 3.14E-03 \| \| ENSG00000173531 \| MST1 \| -0.59 \| 1.79E-03 \| 3.15E-03 \| \| ENSG00000177453 \| NIM1K \| 1.16 \| 1.80E-03 \| 3.16E-03 \| \| ENSG00000156886 \| ITGAD \| -1.83 \| 1.81E-03 \| 3.19E-03 \| \| ENSG00000250182 \|  \| -0.87 \| 1.81E-03 \| 3.19E-03 \| \| ENSG00000185670 \| ZBTB3 \| 0.61 \| 1.84E-03 \| 3.24E-03 \| \| ENSG00000165171 \| METTL27 \| -2.07 \| 1.84E-03 \| 3.24E-03 \| \| ENSG00000213742 \| ZNF337-AS1 \| -0.81 \| 1.85E-03 \| 3.25E-03 \| \| ENSG00000278058 \|  \| -1.72 \| 1.86E-03 \| 3.27E-03 \| \| ENSG00000229914 \|  \| 1.95 \| 1.90E-03 \| 3.34E-03 \| \| ENSG00000231435 \|  \| -1.59 \| 1.91E-03 \| 3.35E-03 \| \| ENSG00000101400 \| SNTA1 \| -0.80 \| 1.91E-03 \| 3.35E-03 \| \| ENSG00000108001 \| EBF3 \| -1.12 \| 1.91E-03 \| 3.36E-03 \| \| ENSG00000102796 \| DHRS12 \| 0.59 \| 1.92E-03 \| 3.38E-03 \| \| ENSG00000258461 \|  \| -1.30 \| 1.98E-03 \| 3.48E-03 \| \| ENSG00000268941 \| LINC01711 \| 1.73 \| 1.99E-03 \| 3.48E-03 \| \| ENSG00000242574 \| HLA-DMB \| -3.56 \| 2.05E-03 \| 3.58E-03 \| \| ENSG00000146205 \| ANO7 \| -0.70 \| 2.07E-03 \| 3.62E-03 \| \| ENSG00000189430 \| NCR1 \| 1.48 \| 2.08E-03 \| 3.64E-03 \| \| ENSG00000099954 \| CECR2 \| 0.81 \| 2.09E-03 \| 3.65E-03 \| \| ENSG00000204815 \| TTC25 \| 1.23 \| 2.09E-03 \| 3.65E-03 \| \| ENSG00000126266 \| FFAR1 \| -2.15 \| 2.09E-03 \| 3.66E-03 \| \| ENSG00000180318 \| ALX1 \| 0.63 \| 2.12E-03 \| 3.70E-03 \| \| ENSG00000165071 \| TMEM71 \| -2.50 \| 2.12E-03 \| 3.70E-03 \| \| ENSG00000213967 \| ZNF726 \| -2.12 \| 2.14E-03 \| 3.74E-03 \| \| ENSG00000241472 \| PTPRG-AS1 \| -2.09 \| 2.15E-03 \| 3.76E-03 \| \| ENSG00000039523 \| RIPOR1 \| -0.66 \| 2.17E-03 \| 3.78E-03 \| \| ENSG00000182508 \| LHFPL1 \| 1.11 \| 2.20E-03 \| 3.83E-03 \| \| ENSG00000138449 \| SLC40A1 \| -2.10 \| 2.21E-03 \| 3.86E-03 \| \| ENSG00000164039 \| BDH2 \| -1.33 \| 2.25E-03 \| 3.92E-03 \| \| ENSG00000225611 \| LINC02158 \| 1.14 \| 2.25E-03 \| 3.92E-03 \| \| ENSG00000112782 \| CLIC5 \| -0.76 \| 2.28E-03 \| 3.96E-03 \| \| ENSG00000111837 \| MAK \| 1.82 \| 2.34E-03 \| 4.07E-03 \| \| ENSG00000258352 \|  \| -1.61 \| 2.35E-03 \| 4.08E-03 \| \| ENSG00000106853 \| PTGR1 \| -0.69 \| 2.36E-03 \| 4.09E-03 \| \| ENSG00000230555 \|  \| -0.97 \| 2.36E-03 \| 4.10E-03 \| \| ENSG00000115718 \| PROC \| -1.68 \| 2.38E-03 \| 4.14E-03 \| \| ENSG00000156049 \| GNA14 \| -1.76 \| 2.40E-03 \| 4.17E-03 \| \| ENSG00000240350 \| LOC105373557 \| 1.92 \| 2.41E-03 \| 4.18E-03 \| \| ENSG00000267458 \|  \| -1.12 \| 2.42E-03 \| 4.19E-03 \| \| ENSG00000251136 \| LOC101929709 \| 0.61 \| 2.44E-03 \| 4.23E-03 \| \| ENSG00000271888 \|  \| 1.40 \| 2.49E-03 \| 4.31E-03 \| \| ENSG00000227543 \| SPAG5-AS1 \| 1.49 \| 2.53E-03 \| 4.38E-03 \| \| ENSG00000164294 \| GPX8 \| -1.42 \| 2.56E-03 \| 4.43E-03 \| \| ENSG00000183929 \|  \| -1.99 \| 2.57E-03 \| 4.44E-03 \| \| ENSG00000100890 \| KIAA0391 \| -0.61 \| 2.58E-03 \| 4.45E-03 \| \| ENSG00000072694 \| FCGR2B \| -0.96 \| 2.59E-03 \| 4.48E-03 \| \| ENSG00000229512 \|  \| 0.99 \| 2.64E-03 \| 4.56E-03 \| \| ENSG00000169397 \| RNASE3 \| -1.40 \| 2.65E-03 \| 4.58E-03 \| \| ENSG00000100027 \| YPEL1 \| 0.86 \| 2.68E-03 \| 4.63E-03 \| \| ENSG00000280693 \| SH3PXD2A-AS1 \| -2.23 \| 2.71E-03 \| 4.68E-03 \| \| ENSG00000149054 \| ZNF215 \| -1.23 \| 2.75E-03 \| 4.74E-03 \| \| ENSG00000176714 \| CCDC121 \| -0.69 \| 2.76E-03 \| 4.75E-03 \| \| ENSG00000238072 \|  \| 1.42 \| 2.77E-03 \| 4.76E-03 \| \| ENSG00000162631 \| NTNG1 \| -1.20 \| 2.79E-03 \| 4.79E-03 \| \| ENSG00000167524 \| SGK494 \| -0.63 \| 2.81E-03 \| 4.83E-03 \| \| ENSG00000228340 \| MIR646HG \| -2.18 \| 2.81E-03 \| 4.83E-03 \| \| ENSG00000228340 \| LOC729296 \| -2.18 \| 2.81E-03 \| 4.83E-03 \| \| ENSG00000107821 \| KAZALD1 \| -1.32 \| 2.83E-03 \| 4.86E-03 \| \| ENSG00000125675 \| GRIA3 \| -1.85 \| 2.86E-03 \| 4.90E-03 \| \| ENSG00000214226 \| C17orf67 \| 0.70 \| 2.86E-03 \| 4.91E-03 \| \| ENSG00000148671 \| ADIRF \| -2.08 \| 2.87E-03 \| 4.92E-03 \| \| ENSG00000237943 \| PRKCQ-AS1 \| -2.25 \| 2.87E-03 \| 4.93E-03 \| \| ENSG00000214029 \| ZNF891 \| 0.70 \| 2.90E-03 \| 4.97E-03 \| \| ENSG00000184343 \| SRPK3 \| 1.07 \| 2.94E-03 \| 5.04E-03 \| \| ENSG00000280213 \| UCKL1-AS1 \| 1.14 \| 2.97E-03 \| 5.08E-03 \| \| ENSG00000272398 \| CD24 \| -1.99 \| 2.97E-03 \| 5.10E-03 \| \| ENSG00000114013 \| CD86 \| -0.84 \| 2.98E-03 \| 5.10E-03 \| \| ENSG00000253177 \|  \| -2.37 \| 2.98E-03 \| 5.10E-03 \| \| ENSG00000125869 \| LAMP5 \| -1.59 \| 2.98E-03 \| 5.10E-03 \| \| ENSG00000010361 \| FUZ \| 0.78 \| 2.98E-03 \| 5.10E-03 \| \| ENSG00000261490 \|  \| -0.99 \| 2.98E-03 \| 5.11E-03 \| \| ENSG00000183186 \| C2CD4C \| -1.34 \| 2.99E-03 \| 5.12E-03 \| \| ENSG00000197933 \| ZNF823 \| -0.81 \| 3.00E-03 \| 5.13E-03 \| \| ENSG00000154783 \| FGD5 \| -2.12 \| 3.01E-03 \| 5.15E-03 \| \| ENSG00000235034 \| C19orf81 \| -0.78 \| 3.03E-03 \| 5.18E-03 \| \| ENSG00000260367 \|  \| 0.78 \| 3.03E-03 \| 5.18E-03 \| \| ENSG00000276231 \| PIK3R6 \| 1.37 \| 3.07E-03 \| 5.24E-03 \| \| ENSG00000092758 \| COL9A3 \| -1.83 \| 3.07E-03 \| 5.25E-03 \| \| ENSG00000255224 \|  \| 0.74 \| 3.09E-03 \| 5.28E-03 \| \| ENSG00000198420 \| TCAF1 \| -0.64 \| 3.09E-03 \| 5.28E-03 \| \| ENSG00000278177 \|  \| 1.17 \| 3.11E-03 \| 5.30E-03 \| \| ENSG00000178498 \| DTX3 \| 0.91 \| 3.11E-03 \| 5.31E-03 \| \| ENSG00000178445 \| GLDC \| -1.18 \| 3.14E-03 \| 5.36E-03 \| \| ENSG00000186765 \| FSCN2 \| -2.65 \| 3.18E-03 \| 5.42E-03 \| \| ENSG00000162881 \| OXER1 \| -0.70 \| 3.21E-03 \| 5.48E-03 \| \| ENSG00000266256 \| LINC00683 \| -2.05 \| 3.24E-03 \| 5.52E-03 \| \| ENSG00000248476 \|  \| 1.38 \| 3.25E-03 \| 5.53E-03 \| \| ENSG00000076826 \| CAMSAP3 \| -0.67 \| 3.26E-03 \| 5.55E-03 \| \| ENSG00000236753 \| MKLN1-AS \| 0.67 \| 3.26E-03 \| 5.56E-03 \| \| ENSG00000253140 \|  \| -0.91 \| 3.28E-03 \| 5.59E-03 \| \| ENSG00000184599 \| FAM19A3 \| -1.89 \| 3.29E-03 \| 5.60E-03 \| \| ENSG00000159164 \| SV2A \| -1.01 \| 3.31E-03 \| 5.64E-03 \| \| ENSG00000233608 \| TWIST2 \| -2.33 \| 3.35E-03 \| 5.71E-03 \| \| ENSG00000272142 \| LYRM4-AS1 \| 0.66 \| 3.38E-03 \| 5.75E-03 \| \| ENSG00000164778 \| EN2 \| 0.90 \| 3.42E-03 \| 5.81E-03 \| \| ENSG00000283317 \|  \| -1.83 \| 3.42E-03 \| 5.81E-03 \| \| ENSG00000279619 \|  \| 1.61 \| 3.43E-03 \| 5.83E-03 \| \| ENSG00000095739 \| BAMBI \| -0.86 \| 3.44E-03 \| 5.84E-03 \| \| ENSG00000106069 \| CHN2 \| -1.63 \| 3.44E-03 \| 5.84E-03 \| \| ENSG00000236683 \|  \| 1.30 \| 3.45E-03 \| 5.86E-03 \| \| ENSG00000183631 \| PRR32 \| -0.70 \| 3.50E-03 \| 5.95E-03 \| \| ENSG00000142235 \| LMTK3 \| -0.84 \| 3.54E-03 \| 6.01E-03 \| \| ENSG00000271797 \|  \| -0.97 \| 3.56E-03 \| 6.05E-03 \| \| ENSG00000158286 \| RNF207 \| -1.89 \| 3.59E-03 \| 6.10E-03 \| \| ENSG00000261556 \| SMG1P7 \| -0.88 \| 3.65E-03 \| 6.18E-03 \| \| ENSG00000205045 \| SLFN12L \| -1.54 \| 3.67E-03 \| 6.22E-03 \| \| ENSG00000249685 \|  \| 1.00 \| 3.76E-03 \| 6.37E-03 \| \| ENSG00000235609 \|  \| 0.88 \| 3.77E-03 \| 6.38E-03 \| \| ENSG00000197977 \| ELOVL2 \| -1.87 \| 3.78E-03 \| 6.41E-03 \| \| ENSG00000197536 \| C5orf56 \| -1.15 \| 3.80E-03 \| 6.44E-03 \| \| ENSG00000129009 \| ISLR \| -0.95 \| 3.80E-03 \| 6.44E-03 \| \| ENSG00000203739 \| LOC101928673 \| 0.85 \| 3.83E-03 \| 6.48E-03 \| \| ENSG00000232671 \| ZNF687-AS1 \| 1.36 \| 3.88E-03 \| 6.56E-03 \| \| ENSG00000230910 \| LOC101928307 \| -1.62 \| 3.90E-03 \| 6.59E-03 \| \| ENSG00000198780 \| FAM169A \| -1.96 \| 3.92E-03 \| 6.63E-03 \| \| ENSG00000237276 \|  \| -1.39 \| 3.93E-03 \| 6.63E-03 \| \| ENSG00000214212 \| C19orf38 \| -1.66 \| 3.94E-03 \| 6.65E-03 \| \| ENSG00000258704 \|  \| -1.30 \| 3.96E-03 \| 6.68E-03 \| \| ENSG00000173611 \| SCAI \| -0.61 \| 3.96E-03 \| 6.69E-03 \| \| ENSG00000257151 \| PWAR6 \| -1.58 \| 3.98E-03 \| 6.71E-03 \| \| ENSG00000237152 \| DLEU7-AS1 \| 1.09 \| 3.98E-03 \| 6.72E-03 \| \| ENSG00000102174 \| PHEX \| -1.84 \| 3.99E-03 \| 6.74E-03 \| \| ENSG00000174236 \| REP15 \| 0.84 \| 4.00E-03 \| 6.75E-03 \| \| ENSG00000152527 \| PLEKHH2 \| -1.29 \| 4.07E-03 \| 6.87E-03 \| \| ENSG00000174564 \| IL20RB \| -1.21 \| 4.08E-03 \| 6.87E-03 \| \| ENSG00000268205 \|  \| 0.59 \| 4.08E-03 \| 6.88E-03 \| \| ENSG00000260196 \|  \| -0.90 \| 4.08E-03 \| 6.88E-03 \| \| ENSG00000276101 \|  \| -1.10 \| 4.11E-03 \| 6.92E-03 \| \| ENSG00000141497 \| ZMYND15 \| -0.87 \| 4.12E-03 \| 6.94E-03 \| \| ENSG00000187185 \| LOC388282 \| -0.94 \| 4.13E-03 \| 6.96E-03 \| \| ENSG00000269069 \|  \| -0.95 \| 4.14E-03 \| 6.97E-03 \| \| ENSG00000260912 \|  \| 0.69 \| 4.17E-03 \| 7.02E-03 \| \| ENSG00000186056 \| MATN1-AS1 \| 0.86 \| 4.18E-03 \| 7.03E-03 \| \| ENSG00000182584 \| ACTL10 \| 0.93 \| 4.21E-03 \| 7.08E-03 \| \| ENSG00000237476 \| LINC01637 \| -0.80 \| 4.29E-03 \| 7.20E-03 \| \| ENSG00000260083 \| MIR762HG \| 0.65 \| 4.30E-03 \| 7.23E-03 \| \| ENSG00000196872 \| KIAA1211L \| -0.85 \| 4.34E-03 \| 7.29E-03 \| \| ENSG00000233493 \| TMEM238 \| -0.90 \| 4.36E-03 \| 7.32E-03 \| \| ENSG00000230606 \|  \| -2.55 \| 4.45E-03 \| 7.45E-03 \| \| ENSG00000213185 \| FAM24B \| -1.45 \| 4.46E-03 \| 7.47E-03 \| \| ENSG00000225345 \|  \| -2.34 \| 4.53E-03 \| 7.59E-03 \| \| ENSG00000248980 \|  \| 0.80 \| 4.59E-03 \| 7.67E-03 \| \| ENSG00000243422 \|  \| -1.65 \| 4.59E-03 \| 7.68E-03 \| \| ENSG00000260793 \|  \| -0.70 \| 4.67E-03 \| 7.81E-03 \| \| ENSG00000259891 \|  \| 0.65 \| 4.69E-03 \| 7.84E-03 \| \| ENSG00000131019 \| ULBP3 \| -0.76 \| 4.70E-03 \| 7.86E-03 \| \| ENSG00000144061 \| NPHP1 \| 1.15 \| 4.75E-03 \| 7.94E-03 \| \| ENSG00000129451 \| KLK10 \| 1.30 \| 4.77E-03 \| 7.97E-03 \| \| ENSG00000204410 \| MSH5 \| -0.64 \| 4.79E-03 \| 8.00E-03 \| \| ENSG00000204410 \| MSH5-SAPCD1 \| -0.64 \| 4.79E-03 \| 8.00E-03 \| \| ENSG00000107614 \| TRDMT1 \| -0.59 \| 4.83E-03 \| 8.06E-03 \| \| ENSG00000174808 \| BTC \| -1.68 \| 4.88E-03 \| 8.14E-03 \| \| ENSG00000182873 \| PRKCZ-AS1 \| 1.01 \| 4.89E-03 \| 8.16E-03 \| \| ENSG00000255182 \|  \| -0.60 \| 4.90E-03 \| 8.18E-03 \| \| ENSG00000129757 \| CDKN1C \| -1.09 \| 4.93E-03 \| 8.22E-03 \| \| ENSG00000136449 \| MYCBPAP \| -1.34 \| 4.94E-03 \| 8.24E-03 \| \| ENSG00000132122 \| SPATA6 \| 0.88 \| 4.96E-03 \| 8.27E-03 \| \| ENSG00000147437 \| GNRH1 \| -1.39 \| 4.96E-03 \| 8.27E-03 \| \| ENSG00000260942 \| CAPN10-DT \| 0.79 \| 4.96E-03 \| 8.27E-03 \| \| ENSG00000185008 \| ROBO2 \| -1.06 \| 4.97E-03 \| 8.29E-03 \| \| ENSG00000169403 \| PTAFR \| -0.77 \| 5.02E-03 \| 8.37E-03 \| \| ENSG00000267598 \|  \| 0.62 \| 5.03E-03 \| 8.38E-03 \| \| ENSG00000177465 \| ACOT4 \| -0.59 \| 5.06E-03 \| 8.42E-03 \| \| ENSG00000205002 \| AARD \| 0.96 \| 5.06E-03 \| 8.42E-03 \| \| ENSG00000184719 \| RNLS \| -0.88 \| 5.07E-03 \| 8.43E-03 \| \| ENSG00000204524 \| ZNF805 \| 0.66 \| 5.10E-03 \| 8.48E-03 \| \| ENSG00000159625 \| DRC7 \| 0.67 \| 5.15E-03 \| 8.57E-03 \| \| ENSG00000255441 \| LOC100129083 \| -2.10 \| 5.18E-03 \| 8.61E-03 \| \| ENSG00000188985 \|  \| -1.73 \| 5.22E-03 \| 8.68E-03 \| \| ENSG00000260572 \|  \| -2.08 \| 5.23E-03 \| 8.68E-03 \| \| ENSG00000172460 \| PRSS30P \| -1.13 \| 5.23E-03 \| 8.69E-03 \| \| ENSG00000177301 \| KCNA2 \| -1.42 \| 5.28E-03 \| 8.77E-03 \| \| ENSG00000196549 \| MME \| -1.31 \| 5.28E-03 \| 8.77E-03 \| \| ENSG00000273329 \|  \| -0.69 \| 5.34E-03 \| 8.86E-03 \| \| ENSG00000279369 \| LOC729683 \| 0.64 \| 5.35E-03 \| 8.88E-03 \| \| ENSG00000153558 \| FBXL2 \| -1.22 \| 5.36E-03 \| 8.89E-03 \| \| ENSG00000172780 \| RAB43 \| 0.59 \| 5.36E-03 \| 8.89E-03 \| \| ENSG00000166896 \| ATP23 \| -0.81 \| 5.37E-03 \| 8.90E-03 \| \| ENSG00000273199 \|  \| 1.28 \| 5.45E-03 \| 9.03E-03 \| \| ENSG00000183722 \| LHFPL6 \| 1.58 \| 5.47E-03 \| 9.06E-03 \| \| ENSG00000175322 \| ZNF519 \| -0.86 \| 5.50E-03 \| 9.10E-03 \| \| ENSG00000248015 \|  \| -1.11 \| 5.51E-03 \| 9.12E-03 \| \| ENSG00000105997 \| HOXA3 \| -1.38 \| 5.52E-03 \| 9.14E-03 \| \| ENSG00000277954 \|  \| -1.52 \| 5.58E-03 \| 9.23E-03 \| \| ENSG00000251364 \| LOC100506258 \| -1.31 \| 5.60E-03 \| 9.26E-03 \| \| ENSG00000214562 \| NUTM2D \| -1.30 \| 5.61E-03 \| 9.27E-03 \| \| ENSG00000270189 \|  \| 1.54 \| 5.61E-03 \| 9.28E-03 \| \| ENSG00000230753 \| ZNF341-AS1 \| 0.97 \| 5.63E-03 \| 9.30E-03 \| \| ENSG00000214199 \|  \| -0.96 \| 5.64E-03 \| 9.32E-03 \| \| ENSG00000273748 \|  \| 0.71 \| 5.68E-03 \| 9.39E-03 \| \| ENSG00000135447 \| PPP1R1A \| -1.35 \| 5.69E-03 \| 9.39E-03 \| \| ENSG00000182983 \| ZNF662 \| -1.11 \| 5.69E-03 \| 9.40E-03 \| \| ENSG00000169962 \| TAS1R3 \| -1.92 \| 5.77E-03 \| 9.52E-03 \| \| ENSG00000237819 \| LOC101927497 \| -0.73 \| 5.78E-03 \| 9.54E-03 \| \| ENSG00000162817 \| C1orf115 \| -0.74 \| 5.83E-03 \| 9.62E-03 \| \| ENSG00000238105 \| GOLGA2P5 \| -1.78 \| 5.87E-03 \| 9.68E-03 \| \| ENSG00000226752 \| CUTALP \| -1.20 \| 5.94E-03 \| 9.79E-03 \| \| ENSG00000280414 \|  \| 0.85 \| 5.96E-03 \| 9.82E-03 \| \| ENSG00000279689 \|  \| -1.26 \| 5.97E-03 \| 9.83E-03 \| \| ENSG00000165238 \| WNK2 \| -1.40 \| 5.97E-03 \| 9.83E-03 \| \| ENSG00000260966 \|  \| 1.02 \| 5.99E-03 \| 9.85E-03 \| \| ENSG00000120093 \| HOXB3 \| -0.84 \| 6.01E-03 \| 9.88E-03 \| \| ENSG00000225335 \|  \| -1.31 \| 6.02E-03 \| 9.90E-03 \| \| ENSG00000182165 \| TP53TG1 \| -1.17 \| 6.05E-03 \| 9.95E-03 \| \| ENSG00000213203 \| GIMAP1 \| -0.91 \| 6.07E-03 \| 9.98E-03 \| | | | | |
[truncated: 156,049 more chars]
