# Supplementary material for: A Genome-Wide Knockout Screen in Human Macrophages Identified Host Factors Modulating Salmonella Infection
Source: mBio. 2019 Oct 8;10(5):e02169-19. doi: 10.1128/mBio.02169-19 (PMC6786873; doi:10.1128/mBio.02169-19)
Supplement: FIG S4 [file mBio.02169-19-sf004.pdf]

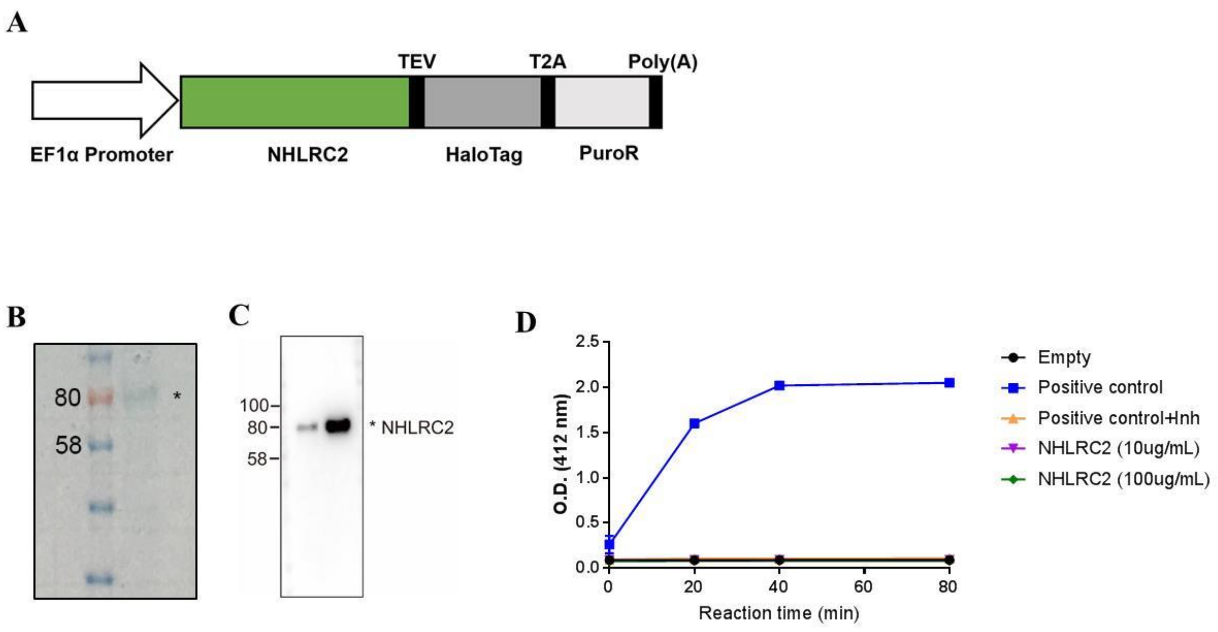

Fig. S4. Purified NHLRC2 proteins did not exhibit thioredoxin activity in a thioredoxin reductase assay.
