# Supplementary material for: A Genome-Wide Knockout Screen in Human Macrophages Identified Host Factors Modulating Salmonella Infection
Source: mBio. 2019 Oct 8;10(5):e02169-19. doi: 10.1128/mBio.02169-19 (PMC6786873; doi:10.1128/mBio.02169-19)
Supplement: TABLE S5 [file mBio.02169-19-st005.docx]

| \| **Supplementary Table 5: List of proteins co-precipitated with NHLRC2 as detected by mass spectrometry.** Results are presented as normalized quantitative values as determined by Scaffold proteome software. The following parameters were used for quantitative analysis: Protein Threshold: 99.0%, Min # Peptides: 2, Peptide Threshold: 95%. \| \| \| \| \| \| \| \| --- \| --- \| --- \| --- \| --- \| --- \| --- \| \|  \|  \|  \|  \|  \|  \| \| \|  \|  \|  \| \| **Column** \| **Description** \|  \|  \|  \|  \| \| \|  \|  \|  \| \| WT IgG s1 \| WT THP-1 macrophages lysates incubated with IgG isotype control antibody replicate 1 \| \| \| \| \| \| \| WT N s1 \| WT THP-1 macrophages lysates incubated with anti-NHLRC2 antibody replicate 1 \| \| \| \| \|  \| \| MUT IgG s1 \| *NHLRC2* macrophages lysates incubated with IgG isotype control antibody replicate 1 \| \| \| \| \|  \| \| MUT N s1 \| *NHLRC2* macrophages lysates incubated with anti-NHLRC2 antibody replicate 1 \| \| \| \| \|  \| \| WT IgG s2 \| WT THP-1 macrophages lysates incubated with IgG isotype control antibody replicate 2 \| \| \| \| \|  \| \| WT N s2 \| WT THP-1 macrophages lysates incubated with anti-NHLRC2 antibody replicate 2 \| \| \| \| \|  \| \| MUT IgG s2 \| *NHLRC2* macrophages lysates incubated with IgG isotype control antibody replicate 2 \| \| \| \| \|  \| \| MUT N s2 \| *NHLRC2* macrophages lysates incubated with anti-NHLRC2 antibody replicate 2 \| \| \| \| \|  \| | | | | | | | | |
| --- | --- | --- | --- | --- | --- | --- | --- | --- | --- | --- | --- | --- | --- | --- | --- | --- | --- | --- | --- | --- | --- | --- | --- | --- | --- | --- | --- | --- | --- | --- | --- | --- | --- | --- | --- | --- | --- | --- | --- | --- | --- | --- | --- | --- | --- | --- | --- | --- | --- | --- | --- | --- | --- | --- | --- | --- | --- | --- | --- | --- | --- | --- | --- | --- | --- | --- | --- | --- | --- | --- | --- | --- | --- | --- | --- | --- | --- | --- | --- | --- | --- | --- | --- | --- | --- | --- | --- | --- | --- | --- | --- | --- | --- |
|  |  |  |  |  |  |  |  |  |
| **Protein Name** | **WT IgG s1** | **WT N s1** | **MUT IgG s1** | **MUT N s1** | **WT IgG s2** | **WT N s2** | **MUT IgG s2** | **MUT N s2** |
| NHLRC2 | 0.00 | 165.07 | 0.00 | 34.08 | 0.00 | 77.90 | 0.00 | 15.33 |
| FRYL | 0.00 | 65.19 | 0.00 | 24.70 | 0.00 | 117.31 | 0.00 | 86.20 |
| HOMER3 | 0.00 | 57.83 | 0.00 | 57.08 | 0.00 | 47.66 | 0.00 | 37.36 |
| KRT79 | 0.00 | 35.75 | 0.00 | 0.00 | 0.00 | 0.00 | 0.00 | 0.00 |
| CEP131 | 0.00 | 23.13 | 0.00 | 24.70 | 0.00 | 15.58 | 0.00 | 24.90 |
| EIF2AK2 | 0.00 | 23.13 | 0.00 | 2.56 | 0.00 | 11.00 | 0.00 | 0.00 |
| IGKV1 | 0.00 | 15.77 | 0.00 | 16.19 | 0.00 | 10.08 | 0.00 | 0.00 |
| STRN3 | 0.00 | 14.72 | 0.00 | 5.11 | 0.00 | 12.83 | 0.00 | 10.54 |
| MIB1 | 0.00 | 11.57 | 0.00 | 11.93 | 0.00 | 8.25 | 0.00 | 9.58 |
| PRDX3 | 0.00 | 11.57 | 0.00 | 4.26 | 0.00 | 5.50 | 0.00 | 0.00 |
| SNAP29 | 0.00 | 10.51 | 0.00 | 8.52 | 0.00 | 3.67 | 0.00 | 3.83 |
| KRT86 | 0.00 | 10.51 | 0.00 | 0.00 | 0.00 | 0.00 | 0.00 | 0.00 |
| FRYL | 0.00 | 9.46 | 0.00 | 0.00 | 0.00 | 39.41 | 0.00 | 34.48 |
| KRT84 | 0.00 | 8.41 | 0.00 | 0.00 | 0.00 | 0.00 | 0.00 | 0.00 |
| EDC4 | 0.00 | 7.36 | 0.00 | 0.00 | 0.00 | 5.50 | 0.00 | 0.00 |
| STAU2 | 0.00 | 7.36 | 0.00 | 0.00 | 0.00 | 2.75 | 0.00 | 0.00 |
| NSF | 0.00 | 6.31 | 0.00 | 9.37 | 0.00 | 0.00 | 0.00 | 0.00 |
| PRKRA | 0.00 | 6.31 | 0.00 | 0.00 | 0.00 | 0.00 | 0.00 | 0.00 |
| HOMER1 | 0.00 | 5.26 | 0.00 | 3.41 | 0.00 | 10.08 | 0.00 | 4.79 |
| KLHL13 | 0.00 | 5.26 | 0.00 | 0.00 | 0.00 | 1.83 | 0.00 | 0.00 |
| VIM | 0.00 | 5.26 | 0.00 | 0.00 | 0.00 | 0.00 | 0.00 | 0.00 |
| ARFGEF1 | 0.00 | 4.21 | 0.00 | 0.00 | 0.00 | 2.75 | 0.00 | 12.45 |
| HMMR | 0.00 | 4.21 | 0.00 | 11.07 | 0.00 | 0.00 | 0.00 | 0.00 |
| ILF2 | 0.00 | 4.21 | 0.00 | 0.00 | 0.00 | 4.58 | 0.00 | 0.00 |
| ALK | 0.00 | 3.15 | 0.00 | 0.00 | 0.00 | 0.00 | 0.00 | 0.00 |
| ACSL3 | 0.00 | 3.15 | 0.00 | 5.11 | 0.00 | 0.00 | 0.00 | 0.00 |
| PIBF1 | 0.00 | 3.15 | 0.00 | 1.70 | 0.00 | 0.00 | 0.00 | 0.00 |
| PPP2R1A | 0.00 | 3.15 | 0.00 | 0.00 | 0.00 | 0.00 | 0.00 | 0.00 |
| PPP2CA | 0.00 | 3.15 | 0.00 | 0.00 | 0.00 | 0.00 | 0.00 | 0.00 |
| FAM83D | 0.00 | 3.15 | 0.00 | 0.00 | 0.00 | 0.00 | 0.00 | 0.00 |
| SCLT1 | 0.00 | 3.15 | 0.00 | 0.00 | 0.00 | 0.00 | 0.00 | 0.00 |
| RAB27A | 0.00 | 3.15 | 0.00 | 0.00 | 0.00 | 0.00 | 0.00 | 0.00 |
| CEP72 | 0.00 | 3.15 | 0.00 | 0.00 | 0.00 | 0.00 | 0.00 | 0.00 |
| VAC14 | 0.00 | 2.10 | 0.00 | 1.70 | 0.00 | 0.00 | 0.00 | 2.87 |
| HERC2 | 0.00 | 2.10 | 0.00 | 2.56 | 0.00 | 0.00 | 0.00 | 2.87 |
| IKPNB1 | 0.00 | 2.10 | 0.00 | 0.00 | 0.00 | 0.00 | 0.00 | 0.00 |
| HSPE1-MOB4 | 0.00 | 2.10 | 0.00 | 0.00 | 0.00 | 1.83 | 0.00 | 0.00 |
| OFD1 | 0.00 | 2.10 | 0.00 | 0.00 | 0.00 | 0.00 | 0.00 | 0.00 |
| C4A | 0.00 | 2.10 | 0.00 | 2.56 | 0.00 | 0.00 | 0.00 | 0.00 |
| TNPO1 | 0.00 | 2.10 | 0.00 | 0.00 | 0.00 | 0.00 | 0.00 | 0.00 |
| PHGDH | 0.00 | 2.10 | 0.00 | 0.00 | 0.00 | 0.00 | 0.00 | 0.00 |
| ARHGAP26 | 0.00 | 2.10 | 0.00 | 1.70 | 0.00 | 0.00 | 0.00 | 3.83 |
| CCDC61 | 0.00 | 2.10 | 0.00 | 0.00 | 0.00 | 0.00 | 0.00 | 0.00 |
| ZNF451 | 0.00 | 2.10 | 0.00 | 3.41 | 0.00 | 0.00 | 0.00 | 0.00 |
| HSD17B11 | 0.00 | 2.10 | 0.00 | 1.70 | 0.00 | 0.00 | 0.00 | 0.00 |
| HAUS1 | 0.00 | 2.10 | 0.00 | 2.56 | 0.00 | 0.00 | 0.00 | 0.00 |
| TSR1 | 0.00 | 2.10 | 0.00 | 0.00 | 0.00 | 0.00 | 0.00 | 0.00 |
| STRIP1 | 0.00 | 2.10 | 0.00 | 0.00 | 0.00 | 0.00 | 0.00 | 0.00 |
| VAMP8 | 0.00 | 2.10 | 0.00 | 1.70 | 0.00 | 0.00 | 0.00 | 0.00 |
| CTTNBP2NL | 0.00 | 2.10 | 0.00 | 0.00 | 0.00 | 0.00 | 0.00 | 0.00 |
| IGHG1 | 0.00 | 0.00 | 0.00 | 15.33 | 0.00 | 0.00 | 0.00 | 0.00 |
| CSN2 | 0.00 | 0.00 | 0.00 | 5.96 | 0.00 | 0.00 | 0.00 | 0.00 |
| PPL | 0.00 | 0.00 | 0.00 | 0.00 | 0.00 | 1.83 | 0.00 | 0.00 |
| SRRM2 | 0.00 | 0.00 | 0.00 | 0.00 | 0.00 | 16.50 | 0.00 | 6.70 |
| CEP290 | 0.00 | 0.00 | 0.00 | 1.70 | 0.00 | 0.00 | 0.00 | 0.00 |
| POF1B | 0.00 | 0.00 | 0.00 | 0.00 | 0.00 | 13.75 | 0.00 | 8.62 |
| EPPK1 | 0.00 | 0.00 | 0.00 | 0.00 | 0.00 | 17.41 | 0.00 | 11.49 |
| C3 | 0.00 | 0.00 | 0.00 | 2.56 | 0.00 | 0.00 | 0.00 | 0.00 |
| IDE | 0.00 | 0.00 | 0.00 | 0.00 | 0.00 | 14.66 | 0.00 | 15.33 |
| CSN1S1 | 0.00 | 0.00 | 0.00 | 12.78 | 0.00 | 0.00 | 0.00 | 1.92 |
| PRPF8 | 0.00 | 0.00 | 0.00 | 1.70 | 0.00 | 4.58 | 0.00 | 0.00 |
| PNP | 0.00 | 0.00 | 0.00 | 0.00 | 0.00 | 12.83 | 0.00 | 11.49 |
| SRSF3 | 0.00 | 0.00 | 0.00 | 2.56 | 0.00 | 2.75 | 0.00 | 0.00 |
| PGK1 | 0.00 | 0.00 | 0.00 | 0.00 | 0.00 | 2.75 | 0.00 | 3.83 |
| SERPINA12 | 0.00 | 0.00 | 0.00 | 0.00 | 0.00 | 2.75 | 0.00 | 5.75 |
| KRT3 | 0.00 | 0.00 | 0.00 | 0.00 | 0.00 | 0.00 | 0.00 | 121.64 |
| NUDC | 0.00 | 0.00 | 0.00 | 1.70 | 0.00 | 0.00 | 0.00 | 0.00 |
| SMC2 | 0.00 | 0.00 | 0.00 | 1.70 | 0.00 | 0.00 | 0.00 | 0.00 |
| DYNLL1 | 0.00 | 0.00 | 0.00 | 2.56 | 0.00 | 3.67 | 0.00 | 2.87 |
| SNRPD3 | 0.00 | 0.00 | 0.00 | 1.70 | 0.00 | 0.00 | 0.00 | 0.00 |
| A2ML1 | 0.00 | 0.00 | 0.00 | 0.00 | 0.00 | 4.58 | 0.00 | 0.00 |
| CHMP2A | 0.00 | 0.00 | 0.00 | 2.56 | 0.00 | 0.00 | 0.00 | 0.00 |
| KRT23 | 0.00 | 0.00 | 0.00 | 0.00 | 0.00 | 13.75 | 0.00 | 7.66 |
| TKT | 0.00 | 0.00 | 0.00 | 0.00 | 0.00 | 6.42 | 0.00 | 9.58 |
| LRRC59 | 0.00 | 0.00 | 0.00 | 2.56 | 0.00 | 0.00 | 0.00 | 0.00 |
| DMKN | 0.00 | 0.00 | 0.00 | 0.00 | 0.00 | 6.42 | 0.00 | 3.83 |
| TXLNA | 0.00 | 0.00 | 0.00 | 4.26 | 0.00 | 0.00 | 0.00 | 0.00 |
| SLC25A11 | 0.00 | 0.00 | 0.00 | 3.41 | 0.00 | 0.00 | 0.00 | 0.00 |
| DPYSL2 | 0.00 | 0.00 | 0.00 | 3.41 | 0.00 | 0.00 | 0.00 | 4.79 |
| S100A11 | 0.00 | 0.00 | 0.00 | 1.70 | 0.00 | 1.83 | 0.00 | 1.92 |
| GN=SF3B3 | 0.00 | 0.00 | 0.00 | 0.00 | 0.00 | 2.75 | 0.00 | 0.00 |
| PRPF6 | 0.00 | 0.00 | 0.00 | 2.56 | 0.00 | 0.00 | 0.00 | 0.00 |
| TCOF1 | 0.00 | 0.00 | 0.00 | 1.70 | 0.00 | 0.00 | 0.00 | 0.00 |
| TPI1 | 0.00 | 0.00 | 0.00 | 1.70 | 0.00 | 6.42 | 0.00 | 0.00 |
| CPA4 | 0.00 | 0.00 | 0.00 | 3.41 | 0.00 | 4.58 | 0.00 | 1.92 |
| GSTP1 | 0.00 | 0.00 | 0.00 | 0.00 | 0.00 | 6.42 | 0.00 | 4.79 |
| ECM1 | 0.00 | 0.00 | 0.00 | 0.00 | 0.00 | 3.67 | 0.00 | 3.83 |
| PSMA5 | 0.00 | 0.00 | 0.00 | 0.00 | 0.00 | 5.50 | 0.00 | 3.83 |
| SPRR1A | 0.00 | 0.00 | 0.00 | 1.70 | 0.00 | 0.00 | 0.00 | 0.00 |
| CAPN1 | 0.00 | 0.00 | 0.00 | 2.56 | 0.00 | 1.83 | 0.00 | 2.87 |
| SPTBN4 | 0.00 | 0.00 | 0.00 | 3.41 | 0.00 | 0.00 | 0.00 | 0.00 |
| SYNCRIP | 0.00 | 0.00 | 0.00 | 0.00 | 0.00 | 1.83 | 0.00 | 0.00 |
| EIF2S2 | 0.00 | 0.00 | 0.00 | 1.70 | 0.00 | 0.00 | 0.00 | 0.00 |
| PTBP1 | 0.00 | 0.00 | 0.00 | 0.00 | 0.00 | 2.75 | 0.00 | 0.00 |
| GN=RAN | 0.00 | 0.00 | 0.00 | 1.70 | 0.00 | 0.00 | 0.00 | 0.00 |
| RAB7A | 0.00 | 0.00 | 0.00 | 0.00 | 0.00 | 3.67 | 0.00 | 1.92 |
| PSMB4 | 0.00 | 0.00 | 0.00 | 0.00 | 0.00 | 4.58 | 0.00 | 2.87 |
| PGM2 | 0.00 | 0.00 | 0.00 | 0.00 | 0.00 | 2.75 | 0.00 | 0.00 |
| LGALS3 | 0.00 | 0.00 | 0.00 | 0.00 | 0.00 | 3.67 | 0.00 | 2.87 |
| ZYX | 0.00 | 0.00 | 0.00 | 1.70 | 0.00 | 0.00 | 0.00 | 0.00 |
| TGM5 | 0.00 | 0.00 | 0.00 | 0.00 | 0.00 | 3.67 | 0.00 | 4.79 |
| ALDH9A1 | 0.00 | 0.00 | 0.00 | 0.00 | 0.00 | 5.50 | 0.00 | 4.79 |
| SNRPD2 | 0.00 | 0.00 | 0.00 | 1.70 | 0.00 | 0.00 | 0.00 | 0.00 |
| RAB14 | 0.00 | 0.00 | 0.00 | 2.56 | 0.00 | 0.00 | 0.00 | 3.83 |
| PSMA1 | 0.00 | 0.00 | 0.00 | 0.00 | 0.00 | 0.00 | 0.00 | 1.92 |
| COPG1 | 0.00 | 0.00 | 0.00 | 1.70 | 0.00 | 0.00 | 0.00 | 0.00 |
| RRBP1 | 0.00 | 0.00 | 0.00 | 1.70 | 0.00 | 0.00 | 0.00 | 0.00 |
| HAUS3 | 0.00 | 0.00 | 0.00 | 5.11 | 0.00 | 0.00 | 0.00 | 0.00 |
| ANXA7 | 0.00 | 0.00 | 0.00 | 0.00 | 0.00 | 6.42 | 0.00 | 0.00 |
| CALML3 | 0.00 | 0.00 | 0.00 | 0.00 | 0.00 | 3.67 | 0.00 | 0.00 |
| PSMB6 | 0.00 | 0.00 | 0.00 | 0.00 | 0.00 | 1.83 | 0.00 | 1.92 |
| HNRNPF | 0.00 | 0.00 | 0.00 | 0.00 | 0.00 | 1.83 | 0.00 | 0.00 |
| P4HB | 0.00 | 0.00 | 0.00 | 3.41 | 0.00 | 0.00 | 0.00 | 0.00 |
| SUN2 | 0.00 | 0.00 | 0.00 | 1.70 | 0.00 | 0.00 | 0.00 | 0.00 |
| PEBP | 0.00 | 0.00 | 0.00 | 0.00 | 0.00 | 3.67 | 0.00 | 1.92 |
| VCL | 0.00 | 0.00 | 0.00 | 0.00 | 0.00 | 3.67 | 0.00 | 1.92 |
| NPEPPS | 0.00 | 0.00 | 0.00 | 0.00 | 0.00 | 4.58 | 0.00 | 2.87 |
| SERPINB5 | 0.00 | 0.00 | 0.00 | 0.00 | 0.00 | 5.50 | 0.00 | 1.92 |
| SLC25A19 | 0.00 | 0.00 | 0.00 | 3.41 | 0.00 | 0.00 | 0.00 | 0.00 |
| RAB32 | 0.00 | 0.00 | 0.00 | 5.11 | 0.00 | 0.00 | 0.00 | 0.00 |
| KRT73 | 0.00 | 0.00 | 0.00 | 0.00 | 0.00 | 0.00 | 0.00 | 17.24 |
| KEAP1 | 0.00 | 0.00 | 0.00 | 0.00 | 0.00 | 0.00 | 0.00 | 2.87 |
| TBC1D15 | 0.00 | 0.00 | 0.00 | 1.70 | 0.00 | 0.00 | 0.00 | 0.00 |
| MAGOHB | 0.00 | 0.00 | 0.00 | 0.00 | 0.00 | 0.00 | 0.00 | 1.92 |
| PARK7 | 0.00 | 0.00 | 0.00 | 0.00 | 0.00 | 2.75 | 0.00 | 1.92 |
| KIF4A | 0.00 | 0.00 | 0.00 | 0.00 | 0.00 | 1.83 | 0.00 | 0.00 |
| DSC2 | 0.00 | 0.00 | 0.00 | 0.00 | 0.00 | 5.50 | 0.00 | 0.00 |
| HARS | 0.00 | 0.00 | 0.00 | 0.00 | 0.00 | 1.83 | 0.00 | 2.87 |
| RALY | 0.00 | 0.00 | 0.00 | 0.00 | 0.00 | 1.83 | 0.00 | 0.00 |
| TLN1 | 0.00 | 0.00 | 0.00 | 1.70 | 0.00 | 0.00 | 0.00 | 0.00 |
| PSMB1 | 0.00 | 0.00 | 0.00 | 0.00 | 0.00 | 1.83 | 0.00 | 2.87 |
| GDI2 | 0.00 | 0.00 | 0.00 | 0.00 | 0.00 | 1.83 | 0.00 | 1.92 |
| RASGRP2 | 0.00 | 0.00 | 0.00 | 1.70 | 0.00 | 0.00 | 0.00 | 0.00 |
| PSMA7 | 0.00 | 0.00 | 0.00 | 0.00 | 0.00 | 2.75 | 0.00 | 1.92 |
| AHCY | 0.00 | 0.00 | 0.00 | 0.00 | 0.00 | 2.75 | 0.00 | 2.87 |
| PRKAR2A | 0.00 | 0.00 | 0.00 | 3.41 | 0.00 | 0.00 | 0.00 | 0.00 |
| NUDT21 | 0.00 | 0.00 | 0.00 | 1.70 | 0.00 | 0.00 | 0.00 | 0.00 |
| GGH | 0.00 | 0.00 | 0.00 | 0.00 | 0.00 | 1.83 | 0.00 | 0.00 |
| RCSD1 | 0.00 | 0.00 | 0.00 | 3.41 | 0.00 | 0.00 | 0.00 | 0.00 |
| DNAJC7 | 0.00 | 0.00 | 0.00 | 1.70 | 0.00 | 0.00 | 0.00 | 0.00 |
| PFN1 | 0.00 | 0.00 | 0.00 | 2.56 | 0.00 | 0.00 | 0.00 | 0.00 |
| SF3B2 | 0.00 | 0.00 | 0.00 | 0.00 | 0.00 | 1.83 | 0.00 | 0.00 |
| PIGR | 0.00 | 0.00 | 0.00 | 4.26 | 0.00 | 0.00 | 0.00 | 0.00 |
| ARCN1 | 0.00 | 0.00 | 0.00 | 2.56 | 0.00 | 0.00 | 0.00 | 0.00 |
| GANAB | 0.00 | 0.00 | 0.00 | 2.56 | 0.00 | 0.00 | 0.00 | 0.00 |
| DNM1L | 0.00 | 0.00 | 0.00 | 1.70 | 0.00 | 0.00 | 0.00 | 0.00 |
| NF2 | 0.00 | 0.00 | 0.00 | 1.70 | 0.00 | 0.00 | 0.00 | 0.00 |
| TPP1 | 0.00 | 0.00 | 0.00 | 0.00 | 0.00 | 1.83 | 0.00 | 1.92 |
| AIMP2 | 0.00 | 0.00 | 0.00 | 1.70 | 0.00 | 0.00 | 0.00 | 0.00 |
| IMPA2 | 0.00 | 0.00 | 0.00 | 0.00 | 0.00 | 1.83 | 0.00 | 0.00 |
| PSMD2 | 0.00 | 0.00 | 0.00 | 1.70 | 0.00 | 0.00 | 0.00 | 0.00 |
| PSMA3 | 0.00 | 0.00 | 0.00 | 0.00 | 0.00 | 3.67 | 0.00 | 1.92 |
| IVL | 0.00 | 0.00 | 0.00 | 0.00 | 0.00 | 1.83 | 0.00 | 1.92 |
| BPIFB1 | 0.00 | 0.00 | 0.00 | 3.41 | 0.00 | 0.00 | 0.00 | 0.00 |
| PSMA6 | 0.00 | 0.00 | 0.00 | 0.00 | 0.00 | 1.83 | 0.00 | 1.92 |
| TRA2B | 0.00 | 0.00 | 0.00 | 0.00 | 0.00 | 1.83 | 0.00 | 0.00 |
| KRT85 | 0.00 | 0.00 | 0.00 | 2.56 | 0.00 | 0.00 | 0.00 | 0.00 |
| CTSC | 0.00 | 0.00 | 0.00 | 0.00 | 0.00 | 1.83 | 0.00 | 1.92 |
| YOD1 | 0.00 | 0.00 | 0.00 | 0.00 | 0.00 | 1.83 | 0.00 | 2.87 |
| CTSA | 0.00 | 0.00 | 0.00 | 0.00 | 0.00 | 1.83 | 0.00 | 1.92 |
| GBA | 0.00 | 0.00 | 0.00 | 0.00 | 0.00 | 1.83 | 0.00 | 1.92 |
| SRI | 0.00 | 0.00 | 0.00 | 0.00 | 0.00 | 1.83 | 0.00 | 2.87 |
| ANXA4 | 0.00 | 0.00 | 0.00 | 0.00 | 0.00 | 0.00 | 0.00 | 1.92 |
| PDCD10 | 0.00 | 0.00 | 0.00 | 0.00 | 0.00 | 1.83 | 0.00 | 0.00 |
| TYMP | 0.00 | 0.00 | 0.00 | 0.00 | 0.00 | 1.83 | 0.00 | 0.00 |
| IGKC | 0.00 | 0.00 | 0.00 | 2.56 | 0.00 | 0.00 | 0.00 | 0.00 |
| SAFB2 | 0.00 | 0.00 | 0.00 | 0.00 | 0.00 | 1.83 | 0.00 | 0.00 |
| GLRX | 0.00 | 0.00 | 0.00 | 0.00 | 0.00 | 1.83 | 0.00 | 1.92 |
| SEC13 | 0.00 | 0.00 | 0.00 | 1.70 | 0.00 | 0.00 | 0.00 | 0.00 |
| VPS26A | 0.00 | 0.00 | 0.00 | 0.00 | 0.00 | 0.00 | 0.00 | 1.92 |
| NOP2 | 0.00 | 0.00 | 0.00 | 1.70 | 0.00 | 0.00 | 0.00 | 0.00 |
| DUSP14 | 0.00 | 0.00 | 0.00 | 0.00 | 0.00 | 2.75 | 0.00 | 0.00 |
| PRDX6 | 0.00 | 0.00 | 0.00 | 0.00 | 0.00 | 2.75 | 0.00 | 0.00 |
| CYC1 | 0.00 | 0.00 | 0.00 | 1.70 | 0.00 | 0.00 | 0.00 | 0.00 |
| HSPA2 | 0.00 | 0.00 | 0.00 | 0.00 | 0.00 | 20.16 | 0.00 | 0.00 |
| PDIA3 | 0.00 | 0.00 | 0.00 | 1.70 | 0.00 | 0.00 | 0.00 | 0.00 |
| IL36G | 0.00 | 0.00 | 0.00 | 0.00 | 0.00 | 1.83 | 0.00 | 0.00 |
| MYL1 | 0.00 | 0.00 | 0.00 | 0.00 | 0.00 | 2.75 | 0.00 | 0.00 |
| NAGK | 0.00 | 0.00 | 0.00 | 0.00 | 0.00 | 1.83 | 0.00 | 0.00 |
| LEMD2 | 0.00 | 0.00 | 0.00 | 1.70 | 0.00 | 0.00 | 0.00 | 0.00 |
| PSMB | 0.00 | 0.00 | 0.00 | 0.00 | 0.00 | 0.00 | 0.00 | 1.92 |
| LAMP1 | 0.00 | 0.00 | 0.00 | 0.00 | 0.00 | 0.00 | 0.00 | 1.92 |
| PREB | 0.00 | 0.00 | 0.00 | 1.70 | 0.00 | 0.00 | 0.00 | 0.00 |
| BCLAF1 | 0.00 | 0.00 | 0.00 | 0.00 | 0.00 | 1.83 | 0.00 | 0.00 |
| APEH | 0.00 | 0.00 | 0.00 | 0.00 | 0.00 | 0.00 | 0.00 | 1.92 |
| TRIM29 | 0.00 | 0.00 | 0.00 | 0.00 | 0.00 | 1.83 | 0.00 | 0.00 |
| BPIFA1 | 0.00 | 0.00 | 0.00 | 1.70 | 0.00 | 0.00 | 0.00 | 0.00 |
| CRYAB | 0.00 | 0.00 | 0.00 | 0.00 | 0.00 | 1.83 | 0.00 | 0.00 |
| MRPS7 | 0.00 | 0.00 | 0.00 | 1.70 | 0.00 | 0.00 | 0.00 | 0.00 |
| HAUS4 | 0.00 | 0.00 | 0.00 | 1.70 | 0.00 | 0.00 | 0.00 | 0.00 |
| TUBB4A | 0.00 | 0.00 | 0.00 | 88.60 | 0.00 | 0.00 | 0.00 | 0.00 |
