# Supplementary material for: A Genome-Wide Knockout Screen in Human Macrophages Identified Host Factors Modulating Salmonella Infection
Source: mBio. 2019 Oct 8;10(5):e02169-19. doi: 10.1128/mBio.02169-19 (PMC6786873; doi:10.1128/mBio.02169-19)
Supplement: TEXT S1 [file mBio.02169-19-s0001.docx]

**SUPPLEMENTAL METHODS**

**Transduction of THP-1 cells.** Transduction of THP-1 cells was performed in 6-well plates. Cells and viral supernatants were mixed in medium supplemented with 8 µg/ml polybrene (TR-1003-G, Millipore). The plates were spun for 90 min at 900*xg* and 32^o^C. Subsequently the plates were incubated overnight at 37^o^C, 5% CO_2_. The next day the medium was refreshed and the day after selected drug was added to the refreshed medium to initiate selection.

**Generation of Cas9-9 THP-1 cells.** THP-1 monocytes were transduced with lentivirus carrying Cas9. The Cas9 lentiviral construct was a gift from Konstantinos Tzelepis. In brief, the construct carries the gene for Cas9 upstream of a blastidicin-resistance gene (map and sequence of the Cas9 vector construct can be found in: **1**). Blastidicin selection at 25 µg/ml was initiated 3 days after transduction (A1113903, ThermoFisher). The Cas9 THP-1 cells were assayed for Cas9 activity using the Cas9 functional assay as described previously (**1**). In brief, the cells were transduced with lentivirus produced with vector containing a GFP gRNA expressed under the U6 promoter and a constitutively expressing gene for Blue Fluorescent Protein (BFP) (gift from Konstantinos Tzelepis). The ratio of cells expressing only BFP to GFP-BFP double positive cells was analysed using the LSRFortessa flow cytometer.

**Generation of Genome-wide Mutant Libraries and Screening.** The human GeCKO v2.0 pooled gRNA library from Sanjana et al. 2014 (**2**) was obtained through Addgene. The half library A comprised of 65,383 sgRNAs (3 targeting gRNAs per gene) targeting 19,050 protein coding genes was amplified as described in Sanjana et al. 2014 (**2**) and the library representation was confirmed by next-generation sequencing (NGS). A total of 2.4 x10^7^ Cas9 THP-1 cells were transduced with the GeCKO v2.0 half library A at an MOI of 0.3 (to ensure 1 sgRNA per cell) and selected in puromycin at 1 µg/ml for 14 days Three independent infections were conducted. Two weeks post-infection, 1.0 x10^7^ cells were pelleted for genomic DNA extraction, while 3.0 x10^7^ mutant monocytic cells were differentiated to macrophages using PMA as described above. On the day of the *Salmonella* infection, 1.0 x10^7^ macrophages were pelleted for DNA extraction, and the remaining macrophages were used for infection assay as described above. After gentamicin treatment, the macrophages were pelleted in PBS and GFP-negative macrophages were sorted using the Influx cell sorter (BD) for DNA extraction. Uninfected live Cas9 THP-1 macrophages were used to create the live and GFP-negative gates. For genomic DNA preparation, we re-suspended the cells in lysis buffer (50 mM Tris-Cl, pH 7.4, 100 mM NaCl, 10 mM EDTA, 0.5% SDS) with 1 mg/ml proteinase K and incubated overnight shaking at 50-55^o^C. Following incubation, DNA was recovered by centrifugation at 1700*xg* for 2 min and washed with 70% ethanol. Air dried DNA pellet was resuspended in TE buffer and used for PCR templates. PCR was performed to amplify the gRNA regions and subsequently sequencing adaptors and barcodes were attached to the samples using the conditions described in Shalem et al. 2014 and Sanjana et al. 2014 (**2,3**) with some modifications. Primer sequences for the PCR amplification are listed in **Table S1** For the first PCR with gDNA of each sample, we performed 96 separate 50 µl reactions with 1 µg genomic DNA in each reaction using Kapa Hifi DNA polymerase (Kapa) and then combined the amplicons. After purification with 100 µl of amplicon using PCR purification kit (Qiagen), second PCR was performed to attach Illumina adaptors and barcode samples. 150 ng of purified first PCR amplicon was reacted in 50 µl reaction volume for the second PCR. Samples were purified with Agencourt AMPure XP beads according to manufacturer’s instructions (Beckman Coulter).

**Western blot, immunoprecipitations, and mass spectrometry.** For Western blot experiments, cell lysates from Cas9 THP-1 and NHLRC2 mutant THP-1 macrophages were prepared using RIPA buffer (RO278, Sigma) and protein concentrations were quantified using Pierce BCA Protein Assay Kit (23225, ThermoFisher). Samples were denatured and ran on a Mini-Protean TGX Precast 12% gels (4561043, Bio-rad), followed by wet transfer onto polyvinylidene difluoride (PVDF) membrane. The membrane was blocked in 3% BSA and then incubated with primary antibody overnight. Subsequently the membrane was washed with TBST and incubated with the appropriate HRP-conjugated secondary antibody. The bands were visualized with Clarity Western ECL substrate (170-5060, Bio-rad) and analyzed using the ImageQuant LAS 4000 (GE Healthcare). Anti-GAPDH antibody (ab9485, Abcam) was used as a loading control.

For immunoprecipitation experiments, cell lysates from WT and NHLRC2 mutant THP-1 macrophages were extracted under non-denaturing condition using Cell Lysis Buffer (9803, Cell Signaling) in the presence of protease and phosphatase inhibitors (ab201119, Abcam). Cell lysates were incubated with 25 μl of protein A magnetic beads (S1425, New England Biolabs) for 1 h at 4°C. After removing protein A magnetic beads, lysate was incubated with 5μg of anti-NHLRC2 antibody (HPA038493, Sigma Aldrich) or IgG isotype control antibody (ab171870, Abcam) for 18 h at 4°C. To precipitate NHLRC2 protein, protein A magnetic beads were added to the lysate, following incubation for 1 h at 4°C. Beads were separated by magnetic separator (DynaMag^TM^-2 Magnet, 12321D, Thermofisher) washed with cold immunoprecipitation buffer 3 times. After boiling of bead pellet in 3X SDS sample loading buffer (187.5 mM Tris-HCl (pH 6.8), 6% (w/v) SDS, 30% glycerol, 150mM DTT, 0.03% (w/v) bromophenol blue and 2% β-mercaptoethanol), beads were removed from supernatant. Proteins were separated by SDS-PAGE and transferred to PVDF membrane. To confirm binding proteins through co-immunoprecipitation, anti-FRYL (AB95065, AbCam), anti-PKR (AB32506, Abcam), anti-Peroxiredoxin 3 (AB129206, Abcam), anti-KHLH13 (MA5-15658, Thermo Fisher Scientific), anti-NHLRC2 (HPA038493, Sigma Aldrich) and anti-GAPDH (AB9484, Abcam) antibodies were used for primary reaction and VeriBlot for IP detection reagent (AB131366, Abcam) was used for secondary reaction.

For mass spectrometry, the boiled supernatants were separated on Tris/Glycine gel and bands per lane were cut into 1 mm^2^ pieces, destained, reduced (DTT) and alkylated (iodoacetamide) and subjected to enzymatic digestion with trypsin overnight at 37°C. After digestion, the supernatant was pipetted into a sample vial and loaded onto an autosampler for automated LC-MS/MS analysis. All LC-MS/MS experiments were performed using a Dionex Ultimate 3000 RSLC nanoUPLC (Thermo Fisher Scientific Inc, Waltham, MA, USA) system and a Q Exactive Orbitrap mass spectrometer (Thermo Fisher Scientific Inc, Waltham, MA, USA). Separation of peptides was performed by reverse-phase chromatography at a flow rate of 300 nL/min and a Thermo Scientific reverse-phase nano Easy-spray column (Thermo Scientific PepMap C18, 2 µm particle size, 100 Å pore size, 75 µm inner diameter (i.d.) x 50 cm length). Peptides were loaded onto a pre-column (Thermo Scientific PepMap 100 C18, 5 µm particle size, 100 Å pore size, 300 µm i.d. x 5 mm length) from the Ultimate 3000 autosampler with 0.1% formic acid for 3 min at a flow rate of 10 µL/min. After this period, the column valve was switched to allow elution of peptides from the pre-column onto the analytical column. Solvent A was water + 0.1% formic acid and solvent B was 80% acetonitrile, 20% water + 0.1% formic acid. The linear gradient employed was 2-40% B in 30 min. Further wash and equilibration steps gave a total run time of 60 min.

The LC eluant was sprayed into the mass spectrometer by means of an Easy-Spray source (Thermo Fisher Scientific Inc.). All *m/z* values of eluting ions were measured in an Orbitrap mass analyzer, set at a resolution of 70000 and was scanned between *m/z* 380-1500. Data dependent scans (Top 20) were employed to automatically isolate and generate fragment ions by higher energy collisional dissociation (HCD, NCE:25%) in the HCD collision cell and measurement of the resulting fragment ions was performed in the Orbitrap analyser, set at a resolution of 17500. Singly charged ions and ions with unassigned charge states were excluded from being selected for MS/MS and a dynamic exclusion window of 20 sec was employed.

Post-run, all MS/MS data were converted to mgf files and the files were then submitted to the Mascot search algorithm (Matrix Science, London UK) and searched against the UniProt human database (71898 sequences; 24121858 residues) and common contaminant sequences containing non-specific proteins such as keratin and trypsin (115 sequences, 38274 residues) removed. Variable modifications of oxidation (M) and deamidation (NQ) were applied as well a fixed modification of carbamidomethyl (C). The peptide and fragment mass tolerances were set to 5 ppm and 0.1 Da, respectively. A significance threshold value of p<0.05 and a peptide cut-off score of 20 were also applied. All data was then imported into the Scaffold program (Version_4.5.4, Proteome Software Inc, Portland, OR).

**Complementation of a NHLRC2 mutation in Cas9 THP-1 cells**

Human NHLRC2 ORF clone was purchased from Origene (SC12157, Origene) and NHLC2 ORF was amplified by PCR for sub-cloning into lentiviral plasmid (primer sequences for PCR amplification are listed in **Table S1**). Each PCR fragments including EF1α promoter, T2A-mCherry, PGK promoter, Neomycin resistant gene and PiggyBac-lentiviral backbone were assembled using Gibson Assembly kit (E5510, New England Biolabs). The construct was confirmed by restriction enzyme digests, PCR, and Sanger sequencing (primer sequences listed in **Table S1**). To generate lentivirus for NHLRC2 complementation, 293T cells were transfected with lentiviral NHLRC2 plasmid using Lipofectamine LTX Reagent with Plus Reagent (15338100, ThermoFisher Scientific). After 72 h of transfection, the viral supernatant was harvested. NHLRC2 mutant cells were incubated with viral supernatants and infected cells were selected against 5 µg/ml Geneticin (10131027, Gibco). The expression of NHLRC2 in THP-1 cells was confirmed by immunoblotting using anti-NHLRC2 antibody (HPA038493, Sigma).

**RNA-Seq analysis.** Sequenced quality was assessed using FastQC v0.11.6 and MultiQC v1.6 (**4**). The FASTQ reads were aligned to the reference human hg19 genome (Ensembl GRCh38.86, **5**) using STAR v2.5 (**6**) and mapped to Ensembl GRCh38 transcripts. Read-counts were generated using htseq-count (HTSeq 0.6.1p1, **7**). Genes with very low counts (with less than 10 counts in three samples which correspond to the smallest number of biological replicates within each treatment group) were pre-filtered and removed *in silico*. Differential gene expression analysis was performed using R version 3.5.2 and DESeq2 v1.22.1 (**8**) and differentially expressed genes called at a p-value <0.01 and a fold change of ≥1.5. Pathway analysis was performed using the innate immunity interactome database and analysis platform, InnateDB (www.innatedb.ca; Breuer 2013) and Sigora 3.0.1 (**9**), while network analyses were performed using NetworkAnalyst (**10**).

**Purification of NHLRC2 protein**. NHLRC2 ORF was sub-cloned into HaloTag expressing plasmid backbone using pCDF1-mAtf4-HT-Puro plasmid (101795, Addgene) by Gibson Assembly method (E5510, New England Biolabs). 293T cells (5.0 x10^7^) were transfected with Halo-NHLRC2 expressing plasmid by electroporation (Neon transfection system, Thermo Fisher Scientific) and incubated for 24 h. Cells were suspended in 1ml of protein purification buffer (1x PBS pH 7.5, 1 mM DTT and 0.005% NP-40) and lysed by repeated freezing-thawing cycles. For the purification of protein from the cell lysate, we used HaloTag® Mammalian protein detection and purification systems (G6795, Promega) and followed manufacture’s protocol. Briefly, lysate was incubated with Halolink resin for 16 h at 4°C. Halolink resin was washed three times, followed by HaloTEV protease cleavage for 16 h at 4°C. To ensure resin-free elution, eluent was purified through spin column. Purified protein was analyzed by SDS-PAGE and following protein detection by Coomassie brilliant blue gel staining or NHLRC2 antibody

**Thioredoxin reductase (TrxR) assay.** For thioredoxin reductase assay of NHLRC2 protein, we followed the provide protocol of Thioredoxin reductase assay kit (AB83463, Abcam). Positive control sample or a range of added amounts of NHLRC2 protein were incubated with 5, 5’-dithiobis (2-nitrobenzoic) acid (DTNB) and NADPH. Samples were subsequently incubated at 25°C for indicated time and then TrxR activity was detected by the measurement of OD_412nm_ value.

**SUPPLEMENTAL REFERENCES**

1. Tzelepis K, Koike-Yusa H, De Braekeleer E, Li Y, Metzakopian E, Dovey OM, Mupo A, Grinkevich V, Li M, Mazan M, Gozdecka M, Ohnishi S, Cooper J, Patel M, McKerrell T, Chen B, Domingues AF, Gallopoli P, Teichmann S, Ponstingl H, McDermott U, Saez-Rodriguez J, Huntly BJP, Iorio F, Pina C, Vassiliou GS, Yusa K. 2016. A CRISPR dropout screen identifies genetic vulnerabilitieis and therapeutic targets in acute myeloid leukemia. Cell Rep 17: 1193-1205.
2. Sanjana NE, Shalem O, Zhang F. 2014. Improved vectors and genome-wide libraries for CRISPR screening. Nat Methods 11: 783-4.
3. Shalem O, Sanjana NE, Hartenian E, Shi X, Scott DA, Mikkelson T, Heckl D, Ebert BL, Root DE, Doench JG, Zhang F. 2014. Genome-scale CRISPR-Cas9 knockout screening in human cells. Science 343: 84-7.
4. Ewels P, Magnusson M, Lundin S, Käller M. 2016. MultiQC: summarize analysis results for multiple tools and samples in a single report. Bioinformatics 32: 3047-8.
5. Zerbino DR, Achuthan P, Akanni W, Amode MR, Barrell D, Bhai J, Billis K, Cummins C, Gall A, Giron CG, Gil L, Gordon L, Haggerty L, Haskell E, Hourlier T, Izuogu OG, Janacek SH, Juettemann T, To JK, Laird MR, Lavidas I, Liu Z, Loveland JE, Maurel T, McLaren W, Moore B, Mudge J, Murphy DN, Newman V, Nuhn M, Ogeh D, Ong CK, Parker A, Patricio M, Riat HS, Schuilenburg H, Sheppard D, Sparrow H, Taylor K, Thormann A, Vullo A, Walts B, Zadissa A, Frankish A, Hunt SE, Kostadima M, Langridge N, Martin FJ, Muffato M, Perry E, Ruffier M, Staines DM, Trevanion SJ, Aken BL, Cunningham F, Yates A, Flicek P. 2018. Ensembl 2018. Nucleic Acids Res. 46: D754-61.
6. Dobin A, Davis CA, Schlesinger F, Drenkow J, Zaleski C, Jha S, Batut P, Chaisson M, Gingeras TR. 2013. STAR: ultrafast universal RNA-Seq aligner. Bioinformatics. 29: 15-21.
7. Anders S, Pyl PT, Huber W. 2015. HTSeq- a Python framework to work with high-throughput sequencing data. Bioinformatics. 31: 166-9.
8. Love MI, Huber W, Anders S. 2014. Moderated estimation of fold change and dispersion for RNA-Seq data with DESeq2. Genome Biol. 15: 550.
9. Foroushani AB, Brinkman FS, Lynn DJ. 2013. Pathway-GPS and SIGORA: identifying relevant pathways based on the over-representation and their gene-pair signatures. PeerJ. 1:e229.
10. Xia J, Gill EE, Hancock RE. 2015. NetworkAnalyst for statistical, visual and network-based meta-analysis of gene expression data. Nature protocols. 10: 823-44.
